# A Benchmark Study of Machine-Learning Regressors and Semiempirical Quantum Methods for CO_2_ Capture in Amine-Based Solvents

**DOI:** 10.1021/acsomega.6c06795

**Published:** 2026-08-26

**Authors:** Jonathan B. Brum, Stanislav R. Stoyanov, Jose Walkimar M. Carneiro, Leonardo M. Costa

**Affiliations:** † Programa de Pós-Graduação em Química, Instituto de Química, Universidade Federal Fluminense, Outeiro de São João Batista s/n, 24020-141 Niterói, RJ, Brazil; ‡ Natural Resources Canada, 428248 CanmetENERGY Devon, 1 Oil Patch Drive, T9G 1A8 Devon, Alberta, Canada

## Abstract

The capture of CO_2_ using amine-based solvents is being continuously improved in an ongoing effort to mitigate anthropogenic CO_2_ emissions. In this study, the 488 CO_2_ capture reactions involving amine-based solvents were optimized, and their vibrational frequencies were calculated at the density functional theory (DFT) level by using the CAM-B3LYP/6–311++G­(d,p) method. Amine-based solvents were selected, including primary, secondary, tertiary, cyclic, and aromatic types containing a variety of functional groups. Due to the computational cost associated with this level of theory, a benchmark study of 17 semiempirical methods was conducted to identify an approach that reproduces the trends predicted by the DFT calculations. The performance of these semiempirical methods was evaluated in terms of geometrical, electronic, and thermodynamic descriptors. Among them, the PM7 method showed the highest overall accuracy compared with DFT results. However, none of the tested methods provided satisfactory correlations for thermodynamic properties such as enthalpy and Gibbs free energy variations. Given the limited accuracy of the semiempirical methods in reproducing thermochemical parameters, 31 regression-based machine learning (ML) models were tested using both cross-validation and train/test strategies. Additionally, regression models were supplied by using features derived from electronic and structural descriptors. Five postprocessing techniques were employed to detect and remove outliers, with efficiency assessed as the ratio of data rejection to the improvement in correlation after treatment. Finally, the Extra Tree method, trained on descriptors calculated at the PM7 level and combined with the anomaly detection robust covariance, exhibited the best correlation and a lower mean absolute error (MAE), (1.26 kcal/mol) with the reference results obtained using DFT. The proposed workflow provides a robust approach to reproducing reference method results using a low-level method augmented by machine learning, significantly reducing the computational cost associated with modeling CO_2_ capture systems and thereby enabling a broader and more efficient exploration of potential sorbent materials.

## Graphical abstract

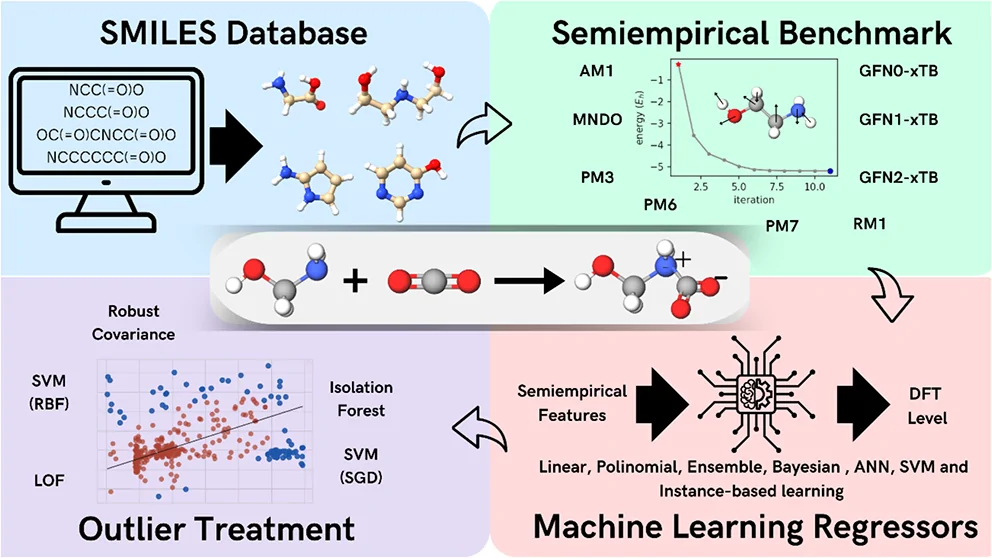

## 1 Introduction

In the last century, increased anthropogenic CO_2_ emissions have been associated in part with the increased use of fossil fuels for energy production.^1–3^ The increased release of CO_2_ into the atmosphere has been linked to rising global temperature, ocean acidification, and even species extinction and respiratory diseases.^4,5^ In the next decades, with the global population projected to increase, the energy demand is expected to grow, and humanity may continue depending on the burning of fossil fuels to meet its energy needs.^6,7^ This scenario can be mitigated by the use of innovative technologies, such as green fuels and carbon capture devices.^8–12^ Traditional precombustion and postcombustion CO_2_ capture processes can contribute to lowering the rate of increase of CO_2_ in the air.^2^ In the past decade, there has been a pronounced increase in the development of chemisorbents related to cyclic adsorption and desorption processes for the capture of CO_2_ from such ultradilute gas streams as the atmospheric air.^11–14^ Advanced materials, such as zeolites, metal–organic frameworks (MOFs), microporous polymers, and amine-based absorption systems, are being extensively explored for CO_2_ sequestration.^11–17^

Numerous computational chemistry studies^18–22^ have examined the interaction between CO_2_ and amines. Zunita et al.^18^ carried out a theoretical study on the interaction between five ionic liquids (ILs) functionalized with amine groups and carbon dioxide (CO_2_), employing the double-ζ B3LYP/6–31+G­(d,p) density functional theory (DFT) method. It was observed that the bond length formed between the IL and CO_2_ constituted the main structural parameter that reflected the intensity of the interaction. This geometric parameter was strongly influenced by the size of the anion present in the ionic liquid. Lemus-Santana^19^ conducted a DFT study using the M06–2X functional with the 6–31+G­(d,p) basis set to investigate the formation of adducts between CO_2_ and heterocycles containing nitrogen atoms with lone pairs (N:). Two orientations of CO_2_ with respect to the plane of the heterocyclic ring were considered: coplanar (in-plane) and perpendicular (top-on). The coplanar complexes are more stable than the perpendicular ones, due to favorable electrostatic and Lewis acid–base interactions. The interaction strength was attributed to a combination of dispersion forces, weak electrostatic interactions, dipole–quadrupole and quadrupole–quadrupole interactions and charge transfer from the heterocyclic ring to the CO_2_ molecule. Higashii et al.^20^ investigated the CO_2_ uptake in aqueous alkanolamine solutions using the triple-ζ B3LYP/6–311++G­(d,p) method in conjunction with the polarizable continuum model (PCM) of solvation. The study evaluated the effect of alkyl chain length and hydroxyl group position in the carbon chain of alkanolamine structures. The results showed that the hydroxyl position significantly influences the reaction products with CO_2_, favoring the formation of intramolecular hydrogen bonds, while variations in alkyl chain length have a minimal impact. These findings indicated that intramolecular hydrogen bonds played a determining role in the CO_2_ capture efficiency in aqueous alkanolamine systems. In a DFT-based CO_2_-amine interaction study with the PBE functional, Yang^21^ demonstrated that the use of primary amines and short carbon chains can improve CO_2_ adsorption performance. Previous analyses conducted by our group^22^ examined the affinity of CO_2_ for a set of 11 amines, revealing that the interaction of the amine N atom with CO_2_ caused a distortion in the geometry of the CO_2_ molecule, changing it from linear to angular in the zwitterionic product. It was also found that the interaction energy was correlated with the basicity of the amine, with more basic amines promoting stronger interactions. The CAM-B3LYP/6–311++G­(d,p) combination of functional and basis set was identified as the best model to approach higher-level bonding and zwitterion optimization calculations.^22^

Recently, machine learning (ML) methods have also been employed in quantum chemistry^23,24^ including to predict the CO_2_-amine interaction efficiency.^25–27^ Shukla et al.^25^ evaluated the performance of aqueous solvents containing amines as potential candidates for CO_2_ capture. Fundamental physicochemical properties, such as density, viscosity, and CO_2_ solubility in each solution, were obtained through data mining techniques using ML algorithms. Models based on Random Forest Regression, Artificial Neural Networks (ANN), and Extreme Gradient Boosting (XGBoost) Regression were developed to predict CO_2_ solubility. The predictive effectiveness of the models was evaluated through statistical analyses, demonstrating that the XGBoost algorithm performed the best. Chen et al.^26^ proposed a physical-chemical coupling ML approach to investigate absorption solvents for carbonyl sulfide (COS) capture. The solubility of 2824 molecules in COS was estimated by integrating physical absorption models, calculated from Henry’s Law, with chemical absorption based on reaction equilibrium calculations, coupled using ML algorithms. Experimental results obtained with four commercial solvents validated the accuracy of the proposed approach in predicting COS solubility in both reactive and nonreactive media. This strategy offers a promising general methodology for the rational design of molecules and materials aiming at enhanced physicochemical synergies in gas capture processes.

The capture of CO_2_ by amine-based solvents has been widely explored due to its efficiency and relatively low cost.^28–34^ On the basis of the studies of Caplow^35^ and Danckwerts,^36^ the CO_2_-amine interaction reaction forms a zwitterionic intermediate. Therefore, the reaction is modulated through the basicity of the amino group (electron density donation) and the acidity of CO_2_, as shown in Figure 1.

**Figure 1:**
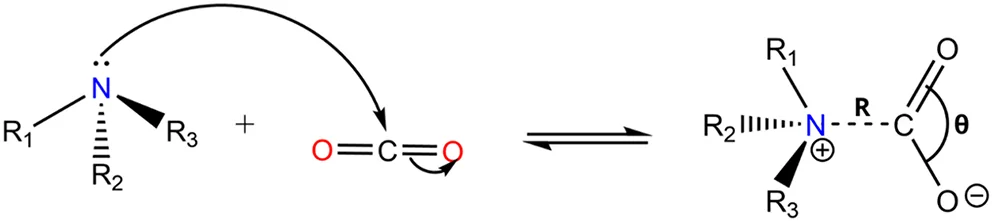
Representation of the CO_2_ capture reaction and acid–base zwitterionic adduct formation.

The stability of the zwitterionic adduct, and consequently the efficiency of CO_2_ capture, is strongly influenced by the amine structure,^36^ by inductive^37^ and steric effects,^38^ and the chain polarizability.^39^ In this way, the efficiency of the reaction can be parametrized by analyzing geometric, thermochemical, quantum, and electronic parameters. In the structure of the acid–base adduct, the bond length (*R*) between the carbon (of CO_2_) and nitrogen atoms (of amines), and the bond angle (θ) of the O=C=O group are considered as descriptors of the degree of interaction. A short distance and a bond angle in the range of [130–145°] suggest that the CO_2_ molecule forms a relatively stable adduct with the amine. The thermochemistry of the reaction is determined by the calculation of the Gibbs free energy (Δ*G*), entropy (Δ*S*), and enthalpy changes (Δ*H*), which allow us to assess, respectively, the spontaneity of the capture process, the impact of reduced degrees of freedom of CO_2_ gas, and the extent to which the adduct is stabilized due to the N–C interaction. Charge transfer (Δ*Q*) allow the evaluation of the effectiveness of electronic donation, proportional to the basicity of the amino group, and the extent of adduct’s polarization, explaining stabilization and destabilization effects caused by alkyl groups and electron-withdrawing or electron-donating substituent in the amine structure.

In the context of carbon capture, zwitterions serve as critical intermediates, whose molecular properties directly influence the efficiency of CO_2_ removal processes. The relationship between structural, thermochemical, and electronic microscopic descriptors with experimental CO_2_ removal can be illustrated through computational studies.^40–43^ Increased branching in a molecular structure significantly enhances the CO_2_ solubility. For instance, branched oligomers can show hundreds of times higher solubility than straight-chain counterparts of the same molecular weight.^40,41^ The presence of specific functional groups, such as primary amine sites, lowers reaction barriers, while substitutions like methyl/ethyl groups or hydroxyl chains can increase them.^42^ The presence of internal hydrogen bonds can substantially reduce CO_2_ absorption rates by hindering the availability of the reactive lone pair.^40–43^ Thermochemical descriptors are also associated with the CO_2_ absorption capacity, particularly through the enthalpy of CO_2_ absorption, which indicates the amount of energy required for the CO_2_ capture and release cycle.^40–42^ Zwitterionic solutions can be engineered to have a lower enthalpy of absorption than traditional solvents, e.g., −38 kJ/mol for some zwitterions versus −53 kJ/mol for the zwitterion of *N*-methyldiethanolamine (MDEA), which reduces the energy needed for regeneration. Additionally, the basicity of the zwitterion precursor, commonly quantified by its p*K*_a_ value, determines the maximum theoretical absorption capacity. Higher p*K*_a_ values generally favor greater CO_2_ uptake, although they may also increase the energetic cost associated with desorption. Electronic descriptors, such as charge transfer, are directly related to reaction kinetics. The first kinetic step involves the nucleophilic attack of the amine lone pair on the electrophilic carbon atom of CO_2_, leading to the formation of a zwitterionic intermediate (Figure 1). Moreover, the overall capture rate is frequently limited by either zwitterion formation or its subsequent deprotonation step. Enhanced intermolecular interactions between the zwitterion and the surrounding solvent molecules (such as water or auxiliary bases) reduce the proton-transfer barrier and consequently accelerate carbamate formation.^43^

In this study, we systematically investigated a broad range of combinations between semiempirical quantum chemical methods and machine learning (ML) regression models to assess their ability to reproduce reference DFT-calculated thermochemical properties. The proposed workflow provides a robust approach for reproducing high-level results using low-level methods augmented with machine learning. The integration of quantum-based descriptors with data-driven models enables a quantitative evaluation of how low-cost electronic structure methods can be efficiently leveraged for predictive thermochemistry. Understanding these relationships provides valuable insights into the transferability and reliability of hybrid semiempirical/ML approaches, potentially paving the way for large-scale screening of amine-based CO_2_ capture agents with reduced computational cost.

## 2 Database Setup and Computational Methodology

Figure 2 presents the workflow and the steps followed in the development of the study, with each step explained in the following sections.

**Figure 2:**
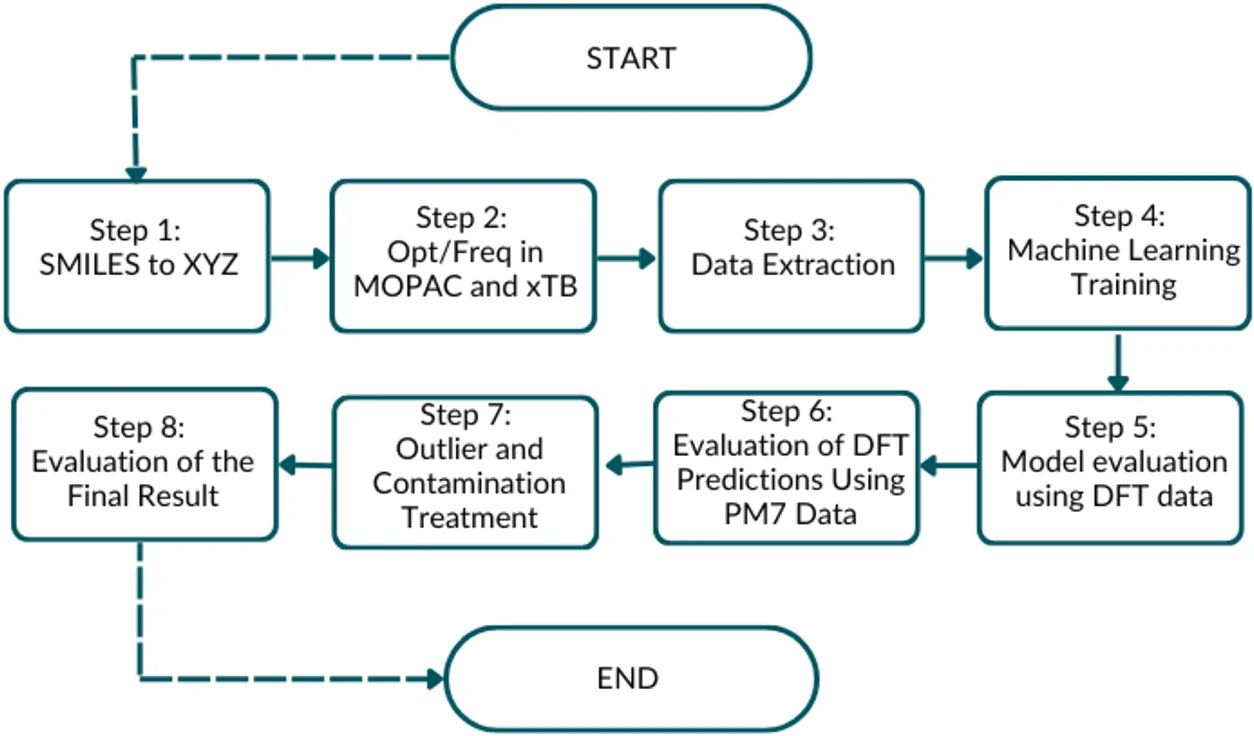
Comprehensive workflow diagram illustrating all steps performed throughout the study.

### 2.1 Structural Data Set

The data set was constructed from 488 amines, generated using Simplified Molecular Input Line Entry System (SMILES), divided into two distinct sets. The first set, comprising 147 amines, includes primary, secondary, and tertiary amines, designed with a second functional group (R) positioned one or two methylene groups (*n* = 1,2; *m* = 1,2) from the reactive Lewis basic nitrogen center. To investigate the effect of the displacement of the functional group (R) from the reactive lone-pair bearing nitrogen site and the inductive influence of the alkyl chain, a functional group R is also positioned from three to nine carbon atoms away (*n* = 3–9) from the N: reactive site of primary amines, totaling 125 molecules. In total, 14 different functional groups (R) were considered, including hydroxyl (−OH), carbonyl (−CO), carboxyl (−COOH), ether (−O−), ester (−COO−), anhydride (−(CO)–O–(CO)–R), amine (−NH_2_), cyan (−CN), nitro (−NO_2_), thiol (−SH), sulfonic acid (−SO_3_H), and halide groups (−F, −Cl, −Br). Figure 3 shows the generic structure of this set. These data are provided in Section S1 of the Supporting Information.

**Figure 3:**
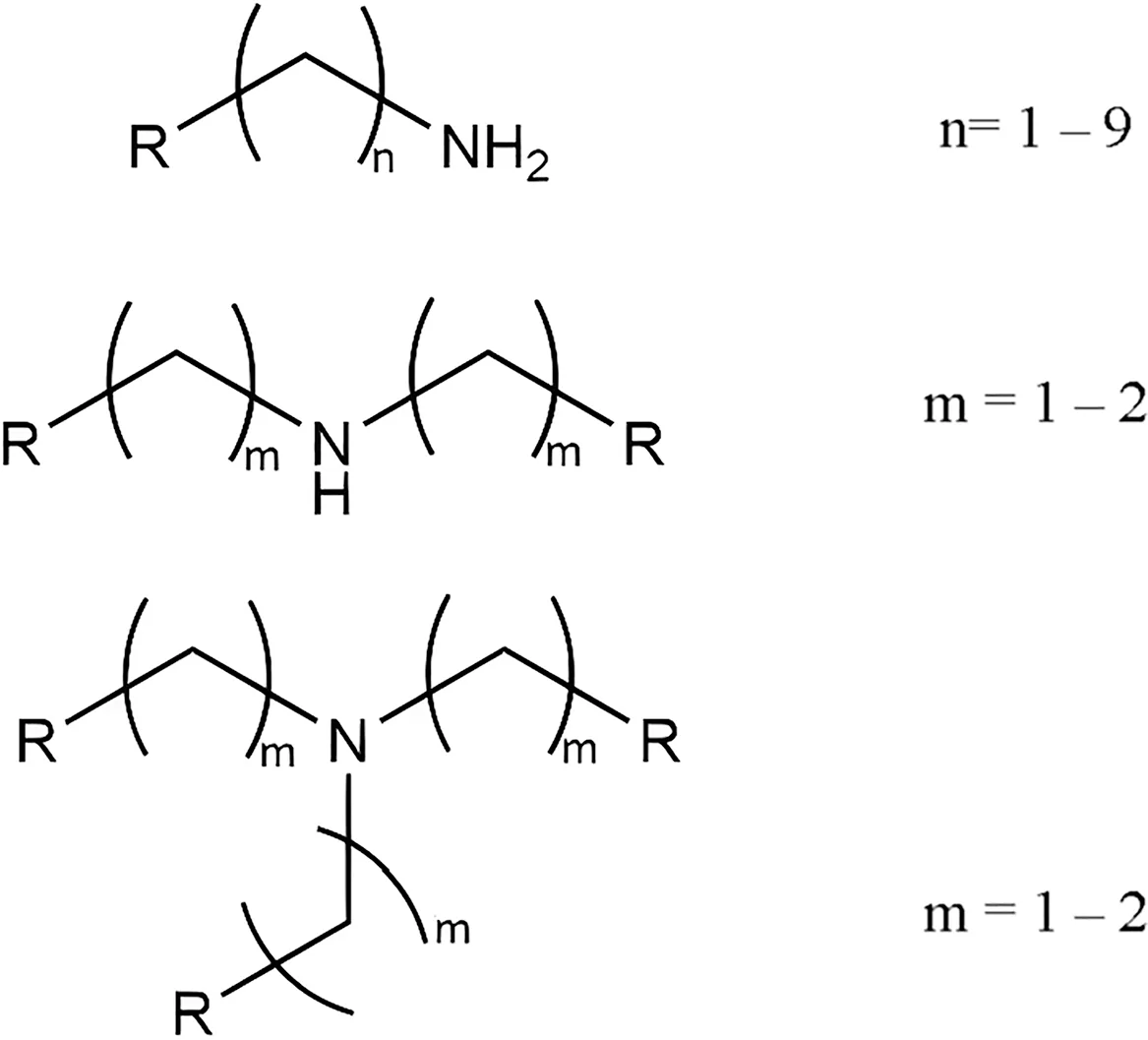
General structures of primary, secondary, and tertiary amines from the first set of the database. The functional groups R are listed in the text.

The second set of compounds is composed of 11 cyclic, aromatic, and saturated amines with different electron-donating and -withdrawing effects (Figure 4). A total of 216 amines were constructed by introducing the functional group R (defined above) at each C atom of the ring. In this way, the study encompasses and explores the widest possible extent of the structural and reactive space of this class of solvents, incorporating a large part of the amine structures that have already been investigated in the literature.^44,45^

**Figure 4:**
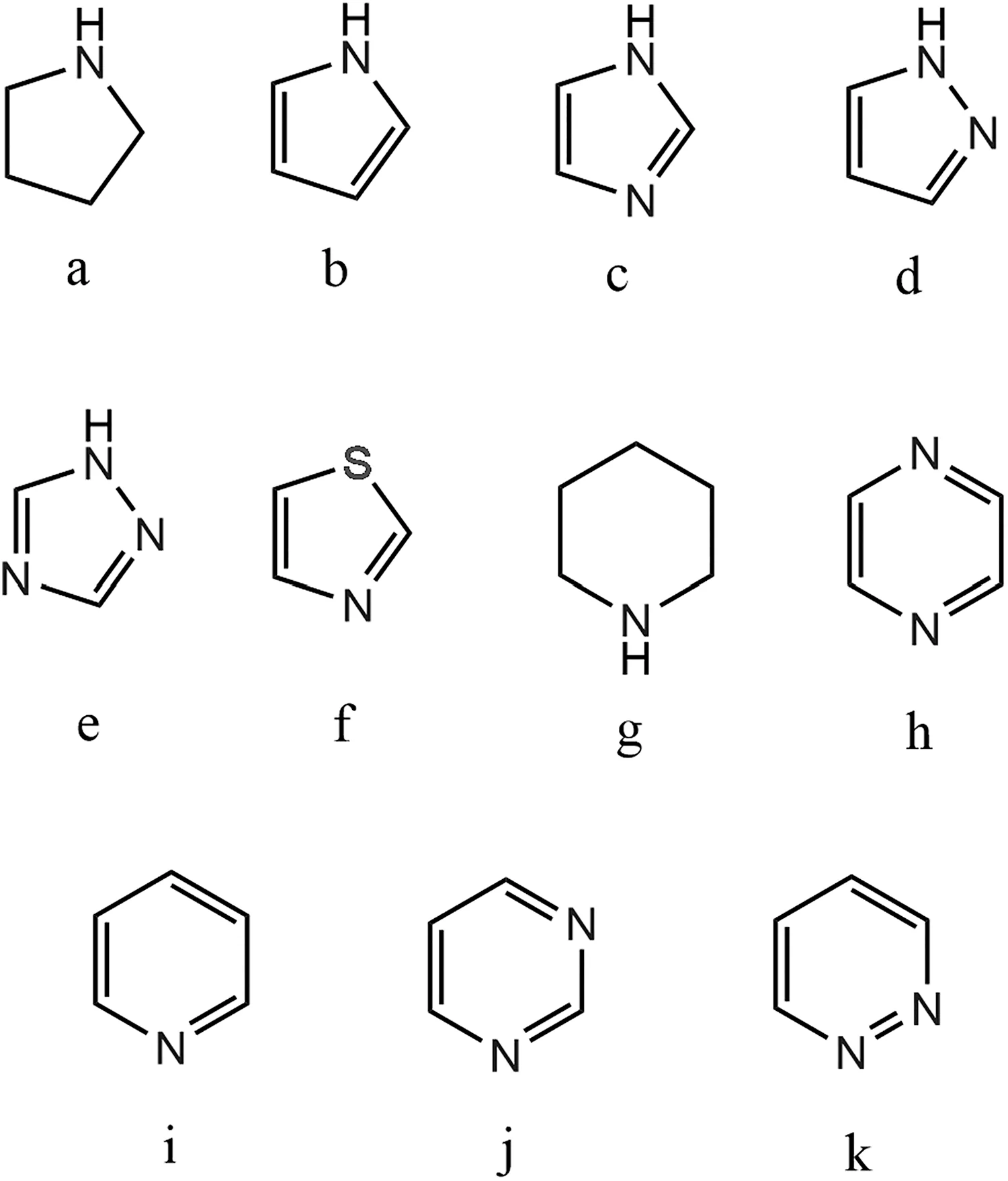
Structures of the second set of amines. (a) Pyrrolidine, (b) pyrrole, (c) imidazole, (d) pyrazole, (e) triazole, (f) thiazole, (g) piperidine, (h) pyrazine, (i) pyridine, (j) pyrimidine, and (k) pyridazine.

Each structure was converted into xyz coordinates using the Open Babel software, employing the Gen3D method.^46^ This approach generates three-dimensional structures based on templates and rules derived from the established structural databases. Initially, a geometry optimization is performed using the MMFF94 force field.^47^ Subsequently, a weighted rotor conformational search was carried out to identify the most stable conformers. Finally, conjugate gradient geometry optimization is performed. The resulting structure is then used as the initial guess for both the DFT and semiempirical calculations.

### 2.2 Computational Technique

The following semiempirical methods were tested: AM1,^48^ MNDO,^49,50^ RM1,^51^ PM3,^52^ PM6,^53^ PM7,^54^ GFN0,^56^ GFN1,^57^ and GFN2.^58^ Geometric, electronic, and thermochemical parameters obtained with these semiempirical methods were compared to those obtained with the DFT CAM-B3LYP/6–311++G­(d,p)^59,60^ method, which was proven to be in agreement with higher levels of theory.^22^ The calculations focused on the geometry optimization of both the reactants (amine and CO_2_) and the zwitterionic adduct as well as on the reaction thermochemistry, calculating the changes in entropy, enthalpy, and Gibbs free energy due to adduct formation. For entropy calculations in solution, ORCA employs the Quasi-RRHO model,^61^ which interpolates between the statistical-mechanical entropy treatments of a harmonic oscillator and a rigid rotor. High-frequency vibrational modes are described as harmonic oscillators, while low-frequency modes are treated as hindered rotational motions. The charge-transfer analysis was carried out based on Mulliken population distribution in the zwitterionic adduct, with particular attention to the net charge gain experienced by the CO_2_ moiety (Figure 1).

The calculations using NDO-based semiempirical methods were performed with the MOPAC software^62^ employing 13 semiempirical methods, including AM1,^48^ MNDO,^49,50^ RM1,^51^ PM3,^52^ PM6,^53^ and PM7 ^54^ and their respective dispersion and hydrogen bonding corrections, such as PM6-D3, PM6-D3H4, PM6-D3H4X, PM6-DH+, and PM6-DH2X. The solvation effect of water was treated using the conductor-like screening model (COSMO).^63^

For calculations using GFN methods, the xTB^55^ software was utilized for geometry optimization and frequency calculations, employing GFN0,^56^ GFN1,^57^ and GFN2 ^58^ with water as an implicit solvent using analytical linearized Poisson–Boltzmann (ALPB).^64^

The molecular structures were generated using Python 3 ^65^ algorithms, leveraging Open Babel^66^ for reading, writing, and manipulating molecular structure files, RDKit^67^ for handling various types of amines and their carboxylation, Pandas^68^ for data processing and management, and Seaborn,^69^ SciPy,^70^ and NumPy^71^ for statistical analysis. For the DFT calculations at the CAM-B3LYP/6–311++G­(d,p)^59,60^ level of theory, the ORCA^72,73^ software was employed, using water as an implicit solvent with the conductor-like polarizable continuum model (CPCM),^74,75^ Chain of Spheres (COSX) algorithm,^76,77^ and advanced integral computation techniques (SHARK).^78^

To evaluate the performance of the distinct semiempirical methods against the DFT results, the statistical metric used was the Pearson Correlation Coefficient (*R*)^79^ (eq 1).

1 $$R=\frac{\sum_{i=1}^{N}\left(x_{i}-\mathrm{x̅}\right)\left(y_{i}-\mathrm{y̅}\right)}{\sqrt{\sum_{i=1}^{N}{\left(x_{i}-\mathrm{x̅}\right)}^{2}}\sqrt{\sum_{i=1}^{N}{\left(y_{i}-\mathrm{y̅}\right)}^{2}}}$$

where *x* and *y* denote the data sets obtained from the DFT calculations and the semiempirical methods, respectively, *x̅* and *y̅* while represent their corresponding mean values, and *N* is the total number of data points.

To evaluate the quantitative accuracy of the employed methodology, the mean absolute error (MAE), the root-mean-square deviation (RMSD), and the 95% confidence interval^80^ (*u*_95%_) were computed, as defined in eqs 2–4, respectively.

2 $$\mathrm{RMSD}=\sqrt{\frac{1}{N}\sum_{i=1}^{N}{\left(y_{i}-\mathrm{y̅}\right)}^{2}}$$

3 $$\mathrm{MAE}=\frac{1}{N}\sum_{i=1}^{N}{|y}_{i}-\overset{®}{\mathrm{y|}}$$

4 $$u_{95\%}={2\sigma }_{\mathrm{rand}+\mathrm{sys}}$$

where *N* denotes the total number of data points, and *y*_*i*_ and *y̅* represent, respectively, the reference DFT values and the mean of the predicted values, and σ_rand+sys_ denotes the combined random and systematic standard deviation of the samples.

### 2.3 ML Regressors, Feature-Based Prediction Methodology and Outlier Treatment

To identify the most appropriate feature regression strategy, the Scikit-Learn library^81^ was employed, and a total of thirty-one regression algorithms were evaluated, covering the full range of supervised learning paradigms implemented in the Python framework. These algorithms were systematically grouped into nine methodological sets. Linear models comprised the Huber regressor, random sample consensus regressor (RANSAC), Theil-Sen estimator, linear support vector regressor (linear SVR), ridge regression, least absolute shrinkage and selection operator (Lasso), elastic net, least angle regression (LARS), stochastic gradient descent regressor (SGDR), passive aggressive regressor (PAR), Tweedie regressor, and transformed target regressor (TTR). Ensemble-based approaches included the histogram-based gradient boosting regressor (Hist-GBR), gradient boosting regressor (GBR), bootstrap aggregation regressor (Bagging), random forest regressor (RF), extra trees regressor (ETR), and decision tree regressor (DTR). Bayesian frameworks encompassed Bayesian ridge regression, automatic relevance determination regression (ARDR), and Gaussian process regressor (GPR). In addition, ANN, polynomial, support vector machine (SVM), and instance-based learning models such as the Radius Neighbors Regressor and *K*-Nearest Neighbors Regressor (KNN) together with Kernel Ridge Regression (KRR) were also considered to ensure broad methodological coverage across diverse learning architectures. Given that the objective is to identify the method that can accurately predict enthalpy variations computed at the DFT level.

In the first stage, the models were trained using distance parameters (*R*), the CO_2_ angle (θ), entropy variation (Δ*S*), and charge transfer (Δ*Q*), all computed at the CAM-B3LYP/6–311++G­(d,p) level, employing a train/test methodology, with 80% of the data used for training and 20% for testing, selected randomly to determine which class of methods is capable of producing routines that yield reliable results. In the second stage, the properties calculated with semiempirical methods were used as input for the previously trained models in order to evaluate which trained routine exhibits the best predictive performance.

Regarding outlier detection and mitigation, five classes of anomaly detection algorithms were employed: robust covariance estimation, one-class support vector machine (SVM) using both radial basis function (RBF) and stochastic gradient descent (SGD) formulations (One-Class SVM, RBF, and SGD variants), isolation forest, and local outlier factor (LOF). Each method requires specifying the contamination level of the data set, that is, the fraction of samples expected to be outliers, to determine the optimal decision boundary between inliers and outliers. Considering this, a broad range of contamination levels (0.10–0.40) was evaluated, and the predictive accuracy of the model was analyzed as a function of this parameter.

## 3 Results

In this section, we compare the results obtained from the 17 semiempirical methods with those from the reference DFT method CAM-B3LYP/6–311++G­(d,p). The following five molecular properties were calculated: geometrical parameters (OCO angle in the CO_2_ moiety and the N···C distance), charge transfer between amine and CO_2_ in the adduct, and changes of entropy (Δ*S*), Gibbs free energy (Δ*G*), and enthalpy (Δ*H*) for the adduct formation reaction (Figure 1). It is noteworthy that these parameters are strongly correlated with the efficiency of CO_2_ capture. When CO_2_ capture is effective, the observed angle typically ranges from 130 to 140°, indicating a geometry close to trigonal.^35–37^ Conversely, when CO_2_ capture does not occur, the angle remains around 180°.

### 3.1 Geometric and Electronic Properties

Figure 5 shows the Pearson correlation coefficients of the computed O=C=O bond angles (θ) and the Nitrogen–Carbon distances (*R*) with those obtained using DFT.

**Figure 5:**
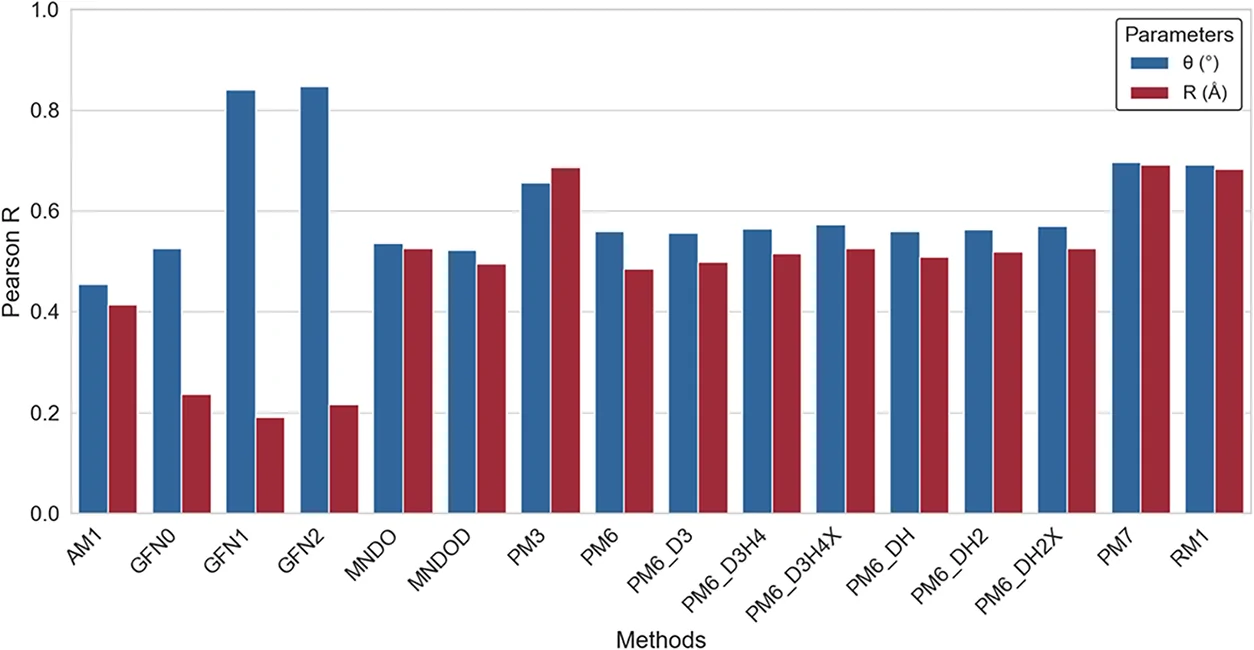
Correlation between semiempirical and DFT-calculated geometric parameters. In blue, OCO bending angles. In red, bond lengths between the N atom of the amine group and the C atom of CO_2_.

The analysis of Figure 5 shows that the GFN1 and GFN2 methods exhibit the largest correlation coefficient (*R*) values of 0.84 and 0.85 for the OCO angle parameter, followed by PM7 (*R* = 0.69) and RM1 (*R* = 0.68), indicating a reasonable ability of these semiempirical methods to reproduce the linear or bent geometries of CO_2_. Correlation coefficient values above 0.9 indicate a very strong degree of correlation, values between 0.9 and 0.7 denote a strong linear correlation, values between 0.7 and 0.5 correspond to an intermediate correlation, values between 0.5 and 0.3 indicate a weak correlation, and values between 0.3 and 0.0 represent a negligible correlation.

The distributions presented in Section S3.5 reveal that GFN2 is able to reproduce the bimodality of linear and bent CO_2_ observed in the DFT data, whereas the PM7 results tend to cluster at around 135°. This duality arises from the efficiency, or lack thereof, of CO_2_ capture by a given amine-based solvent. When the OCO angle is in the range of 175–180° (indicating a linear geometry), the interaction between the amino group and the CO_2_ carbon atom is not indicative of capture. Conversely, when the OCO angle is around 130–140° (indicative of a trigonal geometry), CO_2_ capture is effective.

The results for *R* distances show that PM7 (*R* = 0.70) and RM1 (*R* = 0.69) have the best performance, while the GFN methods showed poor correlations, particularly GFN1 (*R* = 0.19), suggesting their limited ability to describe intermolecular bonding distances. The observed effect is attributed to the large separation range that CO_2_ reaches relative to the amine during the optimization step performed with the GFN1 method. By examining the distribution plots presented in Sections S3.1 and S3.4, it becomes evident that while the DFT-calculated values span a range of approximately 1.5 Å when CO_2_ is captured and between 3 and 4.5 Å when CO_2_ is free, the GFN1 method yields distances between 2 and 3 Å in the captured state and exhibits a much broader distribution, from 4 to 12 Å, in the free state. Consequently, although GFN1 is able to reasonably predict when CO_2_ capture occurs, thereby reproducing the corresponding capture angles, its overall correlation decreases due to the large dispersion in the predicted CO_2_/amine distances.

Figure 6 shows the correlation for the charge transfer between CO_2_ and the amine group calculated by the semiempirical methods (A) and the population distribution of charge transfer values computed using PM7 (B) and DFT (C). Considering that some of the semiempirical methods are capable of describing the structures of the acid–base adducts reasonably well, and that charge derivation methods exhibit significant accuracy, it is not surprising that the charge transfer values are well correlated, as shown in Figure 6A. The PM7 (*R* = 0.77) and RM1 (*R* = 0.75) methods exhibited the largest correlations with DFT-derived atomic charge differences. The GFN1 method also performed satisfactorily well (*R* = 0.74), in contrast to GFN0 (*R* = 0.06), which failed to capture the underlying trend. Population charge transfer distribution plots reveal that the PM7 method (Figure 6B) systematically overestimates the magnitude of negative charges on the CO_2_ moiety, with an accumulation of approximately 300 molecules exhibiting values between −0.6 and −0.5 e^–^. In contrast, the DFT results (Figure 6C) display a broader and less centered distribution, ranging from −0.7 to and 0.4 e^–^.

**Figure 6:**
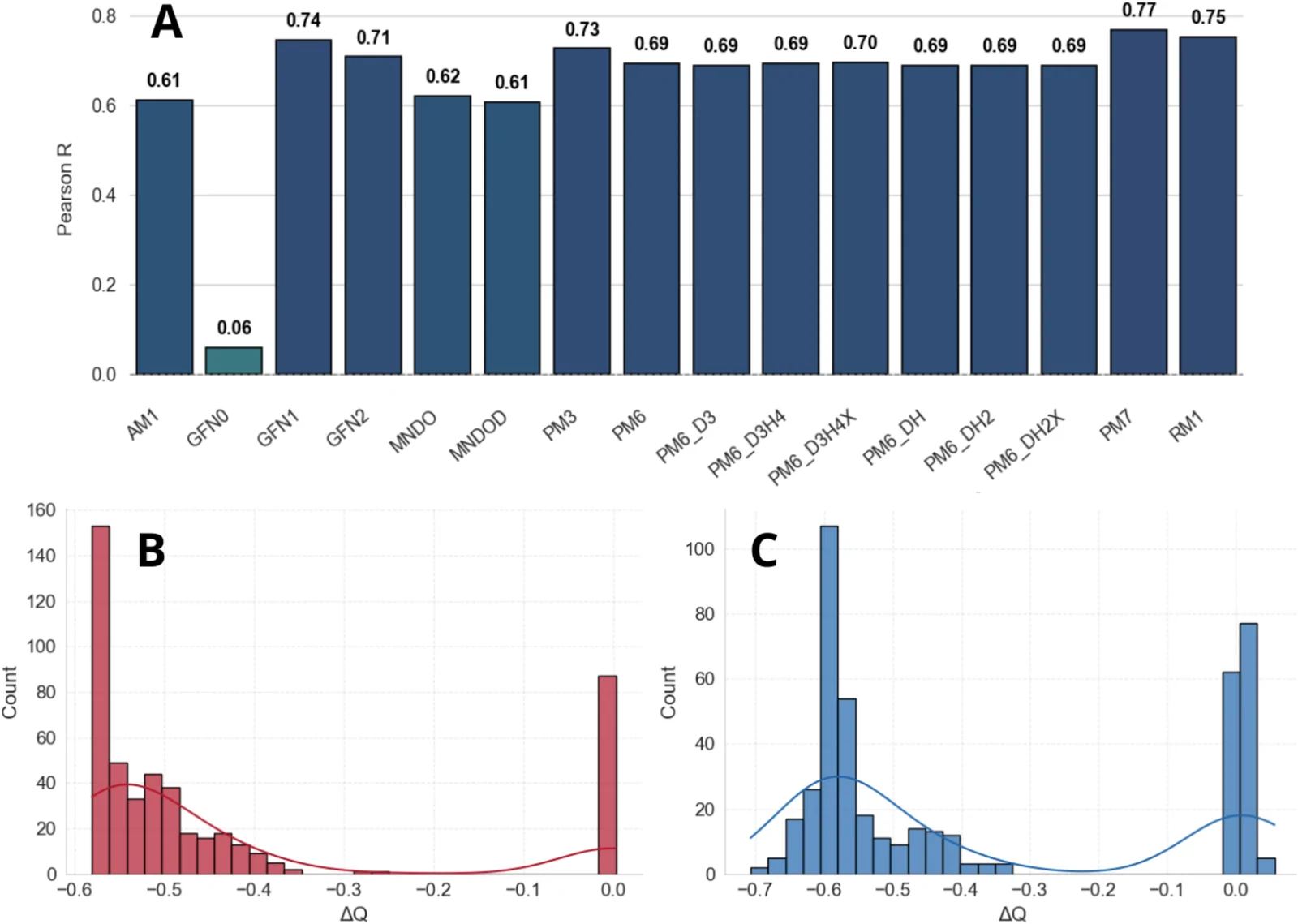
(A) Bar chart containing correlation of charge transfer between CO_2_ and the amine group as calculated by semiempirical methods. Population distribution of charge transfer values computed using the semiempirical PM7 (B) and B3LYP/6–311++G­(d,p) (C).

### 3.2 Thermodynamic Properties

In Figure 7, we present the Pearson *R* values for the thermodynamic properties, entropy, enthalpy, and Gibbs free energy changes. The analysis shows that the variation of entropy is modeled better than the other thermodynamic parameters. This can be attributed to the fact that, in this type of reaction, entropy is intrinsically linked to the loss of degrees of freedom of the whole system, as the amine and CO_2_ molecules form the zwitterionic adduct (Figure 1). Given that semiempirical methods are able to identify the binding event in a manner comparable to that of DFT, entropic effects are consequently incorporated into the calculations. In this context, the PM7 method once again demonstrates superior performance (*R* = 0.69), closely followed by RM1 (*R* = 0.67), indicating a consistent ability to estimate relative entropic variations. The PM6-based approaches exhibited intermediate accuracy (*R* ≃ 0.52–0.57), whereas GFN2 showed a weaker correlation (*R* = 0.39). The distribution plots reveal that both PM7 and RM1 reproduce the DFT reference profile with reasonable fidelity.

**Figure 7:**
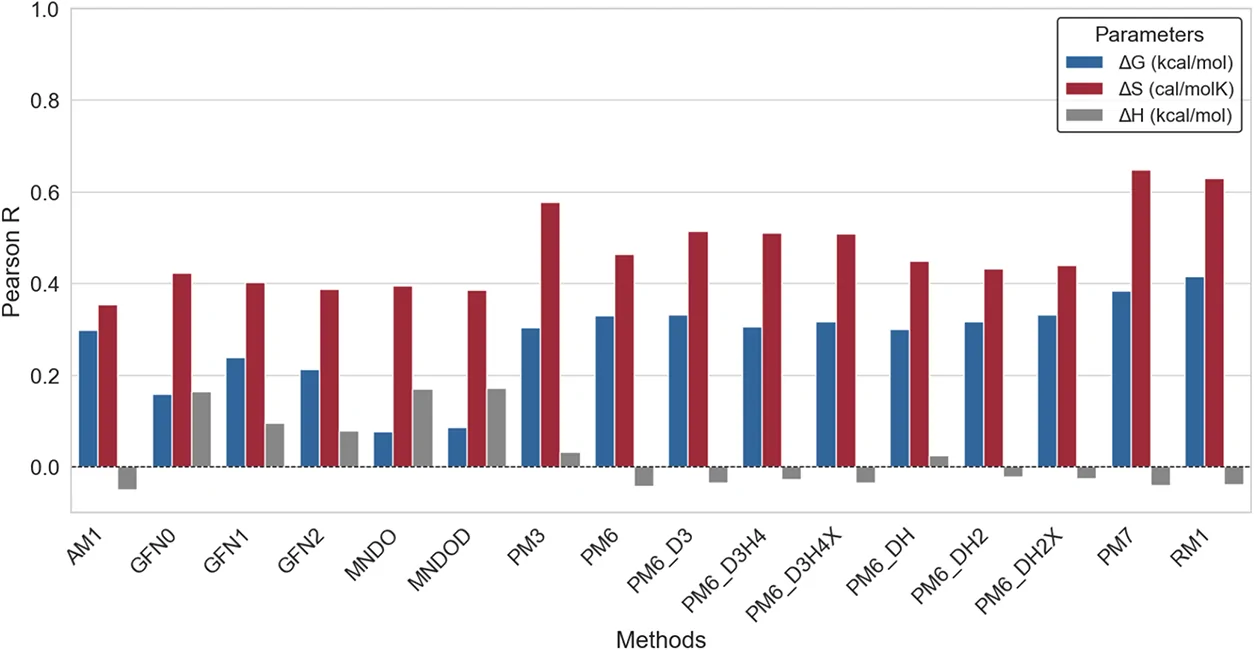
Correlation plots between semiempirical and CAM-B3LYP/6–311++G­(d,p) results for the Gibbs free energy variation, Δ*G* (in blue), variation of entropy, Δ*S* (in red), and enthalpy, Δ*H* (in gray) for the reaction shown in Figure 1.

For enthalpy and Gibbs free energy, none of the evaluated methods provide a significant correlation with DFT values (Figure 7). The Gibbs free energy correlations are generally low across all methods, with PM7 (*R* = 0.37) and RM1 (*R* = 0.40) showing the best, albeit limited, performance. The GFN family performed poorly once again, likely due to the treatment of enthalpic and entropic contributions in their parametrization. For enthalpy values, the highest correlation observed was for GFN0 (*R* = 0.18) with several methods exhibiting negative correlations. This suggests that the description of absolute energetic contributions is particularly sensitive to the choice of method and parametrization, limiting the applicability of these models for accurate thermodynamic assessments. Geometrical, thermodynamic, and charge-transfer data calculated using both the DFT method and the semiempirical methods are provided in Sections S2–S4 of the Supporting Information.

Observing the poor direct correlations between semiempirical methods and the DFT reference data for enthalpy and Gibbs free energy variations (Figure 7), we explored whether supervised ML approaches could identify and learn latent patterns in molecular descriptors derived from PM7 calculations, the semiempirical method that in general performed very well for the evaluated parameters. We propose a feature-based learning strategy using a diverse set of regression algorithms, including linear models (Ridge, Lasso, ElasticNet), kernel-based methods (Support Vector Regressor), ensemble methods (Random Forest Regressor, Gradient Boosting Regressor, Hist Gradient Boosting Regressor, and Extra Trees), nearest neighbors, Gaussian process regressors, and multilayer perceptrons. The strategy consists of incorporating richer geometric and electronic descriptors into the prediction process. Instead of relying solely on enthalpy values, models were trained on feature sets comprising the *R* distance, the θ OCO angle, charge transfer (Δ*Q*), and entropy (Δ*S*) parameters. These molecular descriptors encapsulate latent structure property relationships more effectively than raw energy values alone. The performance of each model is evaluated using Pearson correlation coefficients and cross-validation procedures, with a focus on the ability of the models to reproduce the reference enthalpies obtained from DFT calculations.

### 3.3 Feature-Based Learning for DFT Energy Prediction

For a successful prediction, it is essential that the trained model is capable of accurately inferring DFT values from the input features, while also producing outputs, based on PM7-derived features that are well-correlated with the DFT reference values. Figure 8 provides a comparative overview of model performance, plotting the Pearson correlation between the predictions obtained from training on DFT-derived features and the reference enthalpy values, against the corresponding correlation obtained when the same trained models are applied to PM7-derived descriptors and compared to DFT data. Models located in the upper-right quadrant exhibit the most favorable performance as they combine high internal consistency with strong extrapolative capability across theoretical levels. Residual distributions, parity plots, and additional information are provided in Section S5 of the Supporting Information.

**Figure 8:**
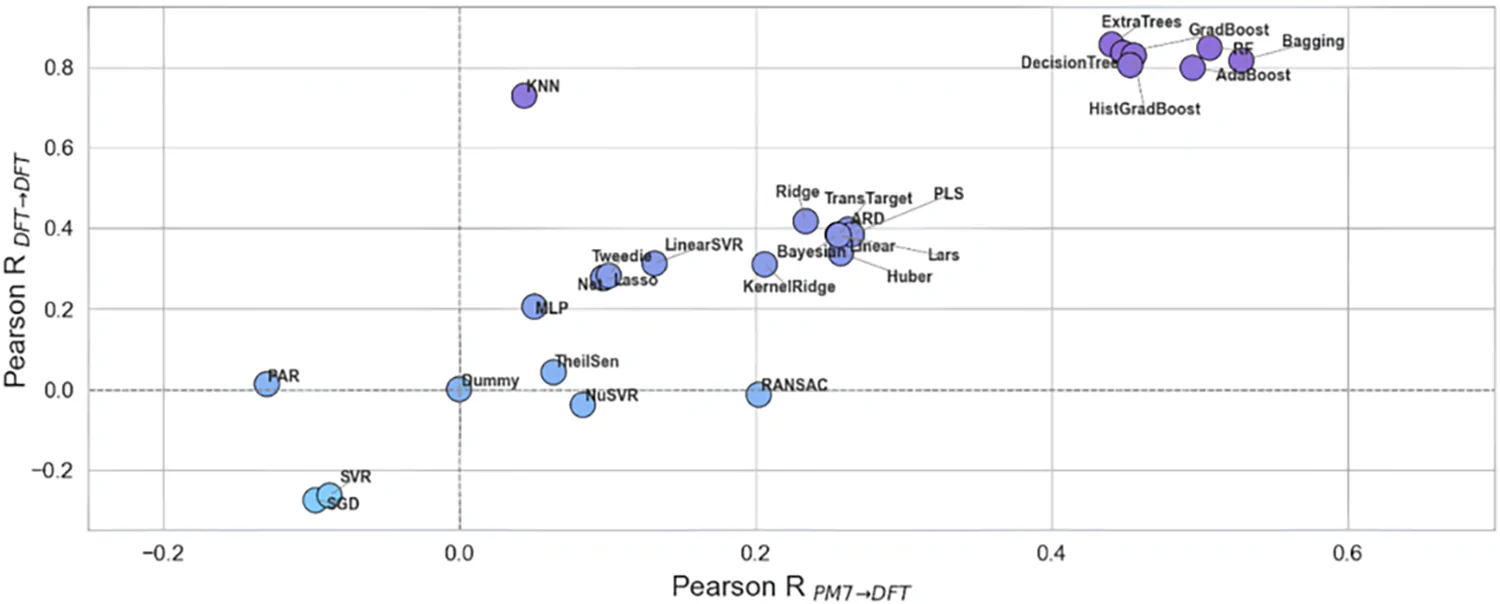
Correlation results between predicted and reference enthalpy variations. The *y*-axis reports the Pearson correlation obtained when the models are trained and evaluated using DFT data (DFT → DFT), whereas the *x*-axis shows the correlation between DFT reference values and the predictions generated by models trained on PM7-derived features (PM7 → DFT).

The ensemble-based regressors (e.g., Gradient Boosting Regressor, Extra Trees Regressor, Random Forest Regressor) clearly outperform other model families, exhibiting strong internal correlation (≥0.8) and intermediate external correlation (0.5–0.6). These models leverage feature interactions and nonlinearities effectively, allowing them to generalize beyond the specific electronic structure method used during training. In contrast, models, such as SVR, SGD Regressor, and RANSAC display near zero or even negative correlation values in both axes, indicating their inadequacy in learning transferable patterns. Interestingly, the *K*-Nearest Neighbors Regressor (KNN) exhibits high internal correlation but fails to generalize externally, suggesting an overreliance on local instance-based learning that does not extrapolate well to differing input domains (PM7 vs DFT).

This systematic benchmarking validates the effectiveness of incorporating structural and electronic features in improving the cross-theory transferability of ML models. Furthermore, it is still possible to improve the external correlation by conducting outlier treatment. A clear clustering of regression methods can be observed, and this grouping is strongly associated with the nature of each method. Three main regions can be identified: an upper-right region characterized by high DFT → DFT correlation values and reasonably good PM7 → DFT correlations; an intermediate region spanning approximately 0.2–0.6 (DFT → DFT) and 0.0–0.4 (PM7 → DFT); and a region comprising negative correlation values. Within the first region, ensemble- and decision tree–based methods are predominant. These approaches yield the best performance because the mapping from PM7 to DFT exhibits pronounced nonlinear deviations, arising from the complex electronic features of the molecules that semiempirical methods often struggle to accurately capture. Consequently, methods that partition the data set and establish decision rules are better suited to reproduce these results than those constrained to a linear error space, such as linear models observed in the intermediate region.

### 3.4 Sensitivity Analysis

To improve the external correlation described in the previous section, a data postprocessing procedure is required. Thus, in this section, we investigate the impact of five distinct outlier filtering techniques, robust covariance, one-class SVM, isolation forest, local outlier factor (LOF), and one-class SVM with stochastic gradient descent (SGD), on the predictive performance of the best performing tree-based ensemble models identified previously. The evaluation is carried out using varying contamination levels to simulate different degrees of data impurity, enabling a comprehensive benchmarking of robustness and model sensitivity. Performance metrics and additional statistical details (Pearson *R*, MAE, and RMSD) are provided in Section S6 of the Supporting Information.

Figure 9 presents the comparative performance of the evaluated regressors across different contamination levels when combined with the robust covariance filter. These models were selected due to their ability to retain a larger fraction of the data set while simultaneously improving the correlation metrics.

**Figure 9:**
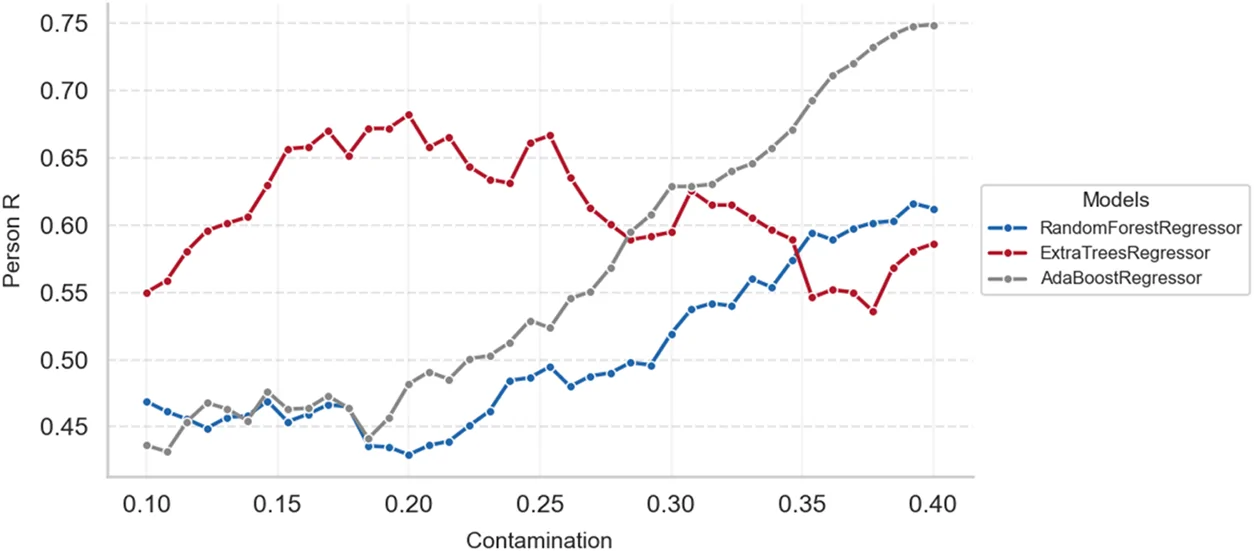
Correlation plots of PM7-predicted values obtained from molecular features, evaluated under different contamination levels. Results after filtering the data set using robust covariance.

The analysis of Figure 9 shows that the Extra Trees Regressor exhibits the most stable and superior performance (*R* ≃ 0.65–0.7), suggesting enhanced resilience to outlier removal at higher contamination levels. Conversely, other models, including Gradient Boosting Regressor and Random Forest Regressor, show pronounced variability and occasional drops in predictive power. Notably, Extra Trees Regressor achieves a steady increase in correlation, peaking at *R* ≃ 0.7 with lower contamination rates. This indicates a high level of robustness to noise and anomalous data, potentially attributable to Extra Trees. However, other models demonstrate substantial volatility in correlation values across the contamination spectrum. This suggests a pronounced dependence on the specific subset of data used for training, which may limit their generalizability in the presence of high outlier prevalence.

Figure 10 presents the combination of extra trees regressor with a robust Covariance using a contamination level of 0.20, which retains approximately three-quarters of the entire data set and achieves a correlation coefficient of 0.68. This reveals that the applied contamination level excludes approximately one-quarter of the data, specifically those lying below the linear fit. This indicates that the robust covariance model filters out values exhibiting a wide range of variation, while the corresponding DFT values remain consistently around ±2.5 kcal/mol. This indicates that the combination of the semiempirical PM7 method as input to the Extra Trees regression algorithm, followed by postprocessing with a robust covariance, enables satisfactory prediction of DFT-calculated Δ*H* values with a significantly reduced computational cost.

**Figure 10:**
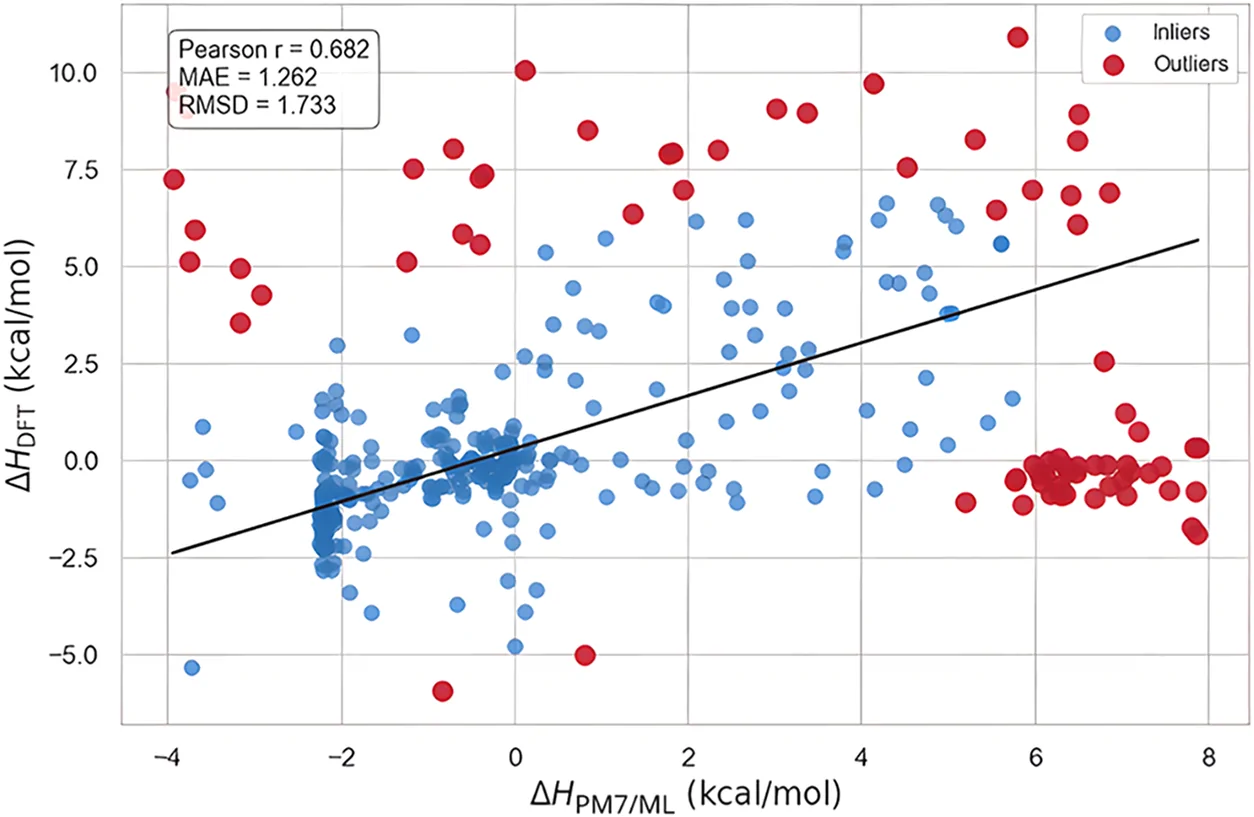
Scatter plot comparing DFT-calculated values with those predicted by the Extra Tree Regressor trained on PM7 descriptors and postprocessed using robust covariance. Inliers retained by the model are shown in blue, while outliers identified and excluded by the algorithm are shown in red.

The error metrics of the PM7/Extra Tree method relative to DFT data reveal a mean absolute error (MAE) of 1.26 kcal/mol and a root-mean-square deviation (RMSD) of 1.73 kcal/mol. Although these values are relatively low, the literature indicates that for thermochemical calculations, deviations around 1 kcal/mol are typically associated with chemical accuracy, implying an offset of approximately 0.26 kcal/mol above the desired threshold. The source of this discrepancy is highlighted by the RMSD value, which being roughly 1.37 times larger than the MAE, suggests a considerable degree of data dispersion. In other words, while most of the data set shows good agreement between PM7/Extra-Tree and DFT results, the presence of a few extreme DFT values contributes significantly to the overall error. The calculated 95% confidence interval of ±3.29 kcal mol^–1^ indicates moderate-to-good predictive accuracy. This deviation is primarily associated with the large structural diversity of the data set, which introduces substantial electronic and thermodynamic variability across the investigated systems. Despite this, the proposed low-cost computational workflow demonstrates useful predictive performance for large-scale molecular screening and trend analysis applications.

Following a detailed analysis of the molecular structures, it was observed that most outliers arise from three main cases: molecules derived from nitrogen-containing heterocycles, particularly thiazole, pyrimidine, and pyrazine; tertiary amines exhibiting significant steric hindrance, which hinders electron density donation during CO_2_ capture; and amines bearing carboxylic acid groups or acyl halides in close proximity to the reactive nitrogen site. In the first case (thiazole, pyrimidine, and pyrazine-derived compounds), it can be noted that for thiazole derivatives the semiempirical method shows very little variation in enthalpy as a function of structural changes. Since the Extra Trees model is trained on these data, its predictions do not accurately reproduce the DFT trend, which exhibits a significantly larger variation. The opposite behavior is observed for pyrimidine and pyrazine derivatives: the DFT-calculated enthalpy variation is close to zero, fluctuating between slightly positive and negative values, whereas the PM7/Extra Trees results display a strongly positive variation. In highly sterically hindered amines, the semiempirical method underestimates Δ*H* values and, consequently, the regressor predictions. Nevertheless, the overall trend is preserved: the pink data points follow a trend parallel to that of the inliers, indicating that although the absolute values are significantly shifted, the structural impact on the energy is consistently captured. Finally, for amines bearing carboxylic groups in close proximity to the reactive nitrogen site, a breakdown of correlation is observed. While DFT predicts significant variations in energy, the semiempirical method yields nearly constant values. This discrepancy arises because carboxylic groups strongly influence the electron density at the nitrogen atom, a feature that semiempirical methods fail to properly describe, thereby rendering these systems as outliers.

## 4 Conclusion

This study demonstrates the viability of employing ML regression models to predict DFT-calculated thermodynamic properties using semiempirical descriptors, particularly those derived from PM7 calculations. The results show that, despite the limited direct correlation between PM7 and DFT values, trained regressors incorporating both geometrical and electronic features were able to capture underlying nonlinear patterns and significantly improve predictive accuracy. Notably, ensemble models such as extra tree, gradient boosting, random forest, and AdaBoost outperformed the linear regressor. Moreover, when combined with anomaly detection methods such as robust covariance, one-class SVM (RBF), and one-class SVM (SGD), these models exhibited strong agreement with DFT results, especially the combination of extra tree and robust covariance, which achieved the highest external correlation.

The accurate description of CO_2_ capture provided by semiempirical methods, particularly PM7 and RM1, was evidenced by the strong correlations observed for geometric and charge transfer descriptors and the interacting energy. This enabled their use in the thermodynamic characterization of the reaction through their implementation as features in the various machine-learning regressors evaluated in this study. This strategy significantly improved the correlation between PM7-predicted and DFT-calculated enthalpy variations, increasing the correlation coefficient from −0.10 for the original PM7 results to 0.68 after ML correction while simultaneously reducing the MAE from 3.08 to 1.26 kcal/mol. The results demonstrate that the proposed hybrid semiempirical/ML workflow provides a reasonably accurate and computationally inexpensive approach to describing the thermodynamic trends associated with CO_2_ capture by amine-based solvents.

These findings underscore the potential of ML-based surrogate models as computationally efficient alternatives for the high-throughput screening of candidate molecules across large chemical spaces. By bypassing the need for exhaustive DFT optimizations in the early stages of molecular evaluation, such models could substantially reduce the computational cost associated with exploratory studies, particularly in the context of the CO_2_ capture. The establishment of a computationally inexpensive yet reasonably accurate workflow paves the way for the construction of reactivity parameter databases, which could facilitate the identification of new materials for efficient CO_2_ capture. Furthermore, the future integration of experimental data, along with the adoption of more robust ML algorithms, such as deep learning, may further enhance the predictive capabilities and generalization of these models.

## Supplementary Material





## Notes

Input files used for all quantum chemical calculations are also available, together with Jupyter Notebook (IPYNB) files containing the scripts used for data analysis, statistical evaluation, and machine-learning workflows (ZIP)
Structural database containing the SMILES notation and molecular structure of each compound; geometrical, thermodynamic, and charge-transfer data calculated using DFT reference method and semiempirical methods; population distributions and histograms of the geometrical, thermodynamic, and charge-transfer descriptors; scatter plots together with Pearson’s *R*, MAE, RMSE, and *R* ^2^ values for the geometrical, thermodynamic, and charge-transfer data obtained with semiempirical methods using DFT calculations as reference; residual distributions and parity plots for the ensemble machine-learning regressors; outlier analyses employing robust covariance, one-class SVM, isolation forest, local outlier factor, and one-class SVM within ensemble frameworks, as well as correlation plots as a function of contamination level and performance metrics (PDF)
