# Supplementary material for: A Benchmark Study of Machine-Learning Regressors and Semiempirical Quantum Methods for CO2 Capture in Amine-Based Solvents
Source: ACS Omega. 2026 Aug 26;11(35):53415–26. doi: 10.1021/acsomega.6c06795 (PMC13564018; doi:10.1021/acsomega.6c06795)
Supplement: Supplementary file 1 [file ao6c06795_si_002.pdf]

Supporting Information:

A Benchmark Study of Machine Learning Regressors and Semi-Empirical Quantum Methods for CO_2 Capture in Amine-Based Solvents

Jonathan B. Brum†, Stanislav R. Stoyanov‡,*, Jose Walkimar M. Carneiro†, Leonardo M. Costa†,*

†Programa de Pós-Graduação em Química, Instituto de Química, Universidade Federal Fluminense. Outeiro de São João Batista s/n 24020-141 Niterói. RJ. Brazil.

‡Natural Resources Canada, CanmetENERGY Devon, 1 Oil Patch Drive, T9G 1A8 Devon, Alberta, Canada

Corresponding author emails: **stanislav.stoyanov@nrcan-rncan.gc.ca**

Contents

S1. Structures DataBase.......4

S2. Geometrical, Thermodynamic, and Charge-Transfer Data calculated using the DFT method and the semiempirical methods.......73

S3. Population distribution analysis and histograms of geometric, thermodynamic, and charge-transfer data calculated using the DFT method and the semiempirical methods.......304

S.1 Population distribution analysis and histograms of geometric, thermodynamic, and charge-transfer data calculated using DFT.......304

S3. 2 Population distribution analysis and histograms of geometric, thermodynamic, and charge-transfer data calculated using AM1 method.......307

S3. 3 Population distribution analysis and histograms of geometric, thermodynamic, and charge-transfer data calculated using GFN0 method.......309

S3. 4 Population distribution analysis and histograms of geometric, thermodynamic, and charge-transfer data calculated using GFN1 method.......312

S3. 5 Population distribution analysis and histograms of geometric, thermodynamic, and charge-transfer data calculated using GFN2 method.......315

S3. 6 Population distribution analysis and histograms of geometric, thermodynamic, and charge-transfer data calculated using MNDO method.......318

S3. 7 Population distribution analysis and histograms of geometric, thermodynamic, and charge-transfer data calculated using MNDOD method 321

S3. 8 Population distribution analysis and histograms of geometric, thermodynamic, and charge-transfer data calculated using PM3 method 324

S3. 9 Population distribution analysis and histograms of geometric, thermodynamic, and charge-transfer data calculated using PM6 method 328

S3. 10 Population distribution analysis and histograms of geometric, thermodynamic, and charge-transfer data calculated using PM7 method 330

S3. 11 Population distribution analysis and histograms of geometric, thermodynamic, and charge-transfer data calculated using RM1 method...333

S4. Scatter plots, Pearson R, MAE, RMSE, and R^2 for geometrical, thermodynamic, and charge-transfer data calculated using the semiempirical methods, employing the DFT method as reference. 336

S4. 1 Correlations between the AM1 method and the CAM-B3LYP/6-311++G(d,p) level of theory 337

S4. 2 Correlations between the GFN0 method and the CAM-B3LYP/6-311++G(d,p) level of theory 339

S4. 3 Correlations between the GFN1 method and the CAM-B3LYP/6-311++G(d,p) level of theory 341

S4. 4 Correlations between the GFN2 method and the CAM-B3LYP/6-311++G(d,p) level of theory 344

S4. 5 Correlations between the MNDO method and the CAM-B3LYP/6-311++G(d,p) level of theory 346

S4. 6 Correlations between the MNDOD method and the CAM-B3LYP/6-311++G(d,p) level of theory 349

S4. 7 Correlations between the PM3 method and the CAM-B3LYP/6-311++G(d,p) level of theory 351

S4. 8 Correlations between the PM6 method and the CAM-B3LYP/6-311++G(d,p) level of theory 354

S4. 9 Correlations between the PM7 method and the CAM-B3LYP/6-311++G(d,p) level of theory 356

S4. 10 Correlations between the RM1 method and the CAM-B3LYP/6-311++G(d,p) level of theory 359

S5. Residual distributions and parity plots in Ensemble ML Regressor Methods. 361

S5.1 Randon Forest Regressor 361

S5.2 Extra Trees Regressor...363

S5.3 Gradient Boosting Regressor 365

S5.4 Ada Boost Regressor 367

S5.5 Hist Gradient Boosting Regressor 369
S5.6 Bagging Regressor 371
S5.7 Decision Tree Regressor 373
S6. Outlier treatment using Robust Covariance, One-Class SVM, Isolation Forest, Local Outlier Factor (LOF), and One-Class SVM within ensemble frameworks. Correlation plots as a function of contamination level and correlation plots with performance metrics (Pearson's r, MAE, RMSD). 375
S6.1 One-Class SVM (RBF) 376
S6.2 One-Class SVM (SGD) 382
S6.3 Robust Covariance 389
S6.4 Isolation Forest 396
S6.5 Local Outlier Factor 404

S1. Structures DataBase

Table S1: Database of amine structures, including the database identifier, SMILES representation, and molecular structure.

File Name	SMILES	Structure
Acid0	NCC(=O)O	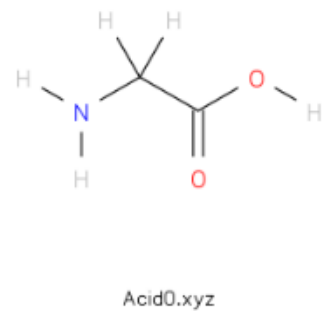
Acid1	NCCC(=O)O	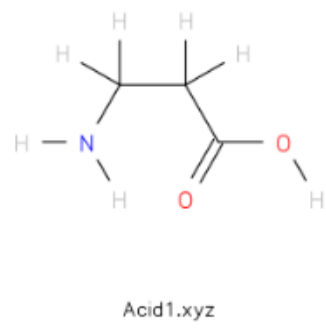
Acid2	NCCCC(=O)O	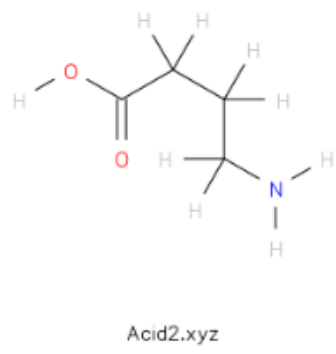
Acid30	OC(=O)CNCC(=O)O	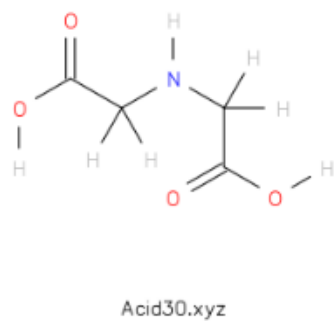
Acid3V2	NCCCCC(=O)O	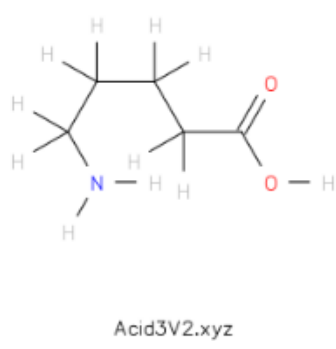
Acid430	OC(=O)CN(CC(=O)O)CC (=O)O	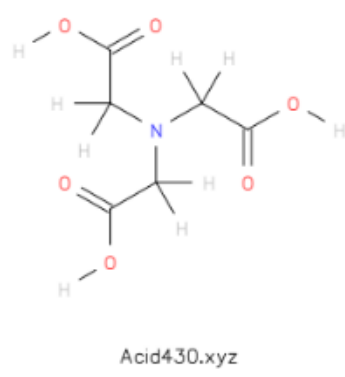

Acid4V2	NCCCCCC(=O)O	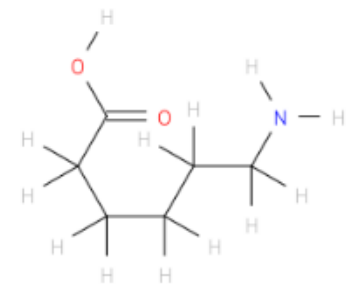 Acid4V2.xyz
Acid50	OC(=O)CNCCC(=O)O	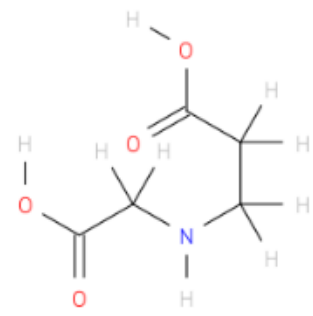 Acid50.xyz
Acid5V2	NCCCCCCC(=O)O	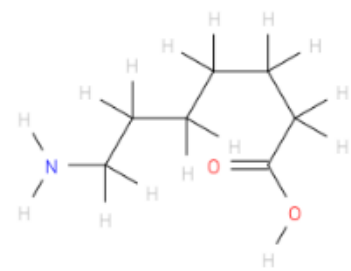 Acid5V2.xyz
Acid671	OC(=O)CCN(CCC(=O)O) CCC(=O)O	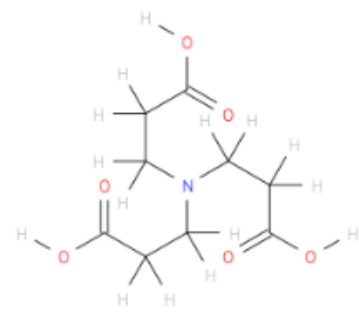 Acid671.xyz
Acid6V2	NCCCCCCCC(=O)O	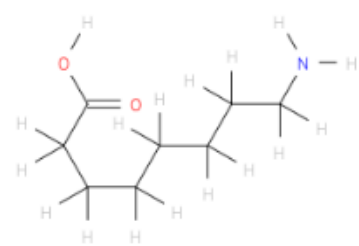 Acid6V2.xyz
Acid7V2	NCCCCCCCCC(=O)O	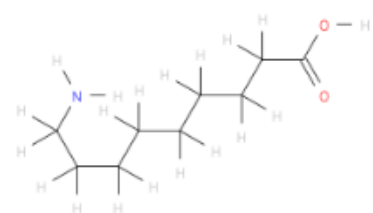 Acid7V2.xyz
Acid8V2	NCCCCCCCCCC(=O)O	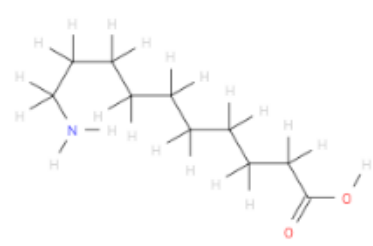 Acid8V2.xyz

AcidBr0	NCC(=O)Br	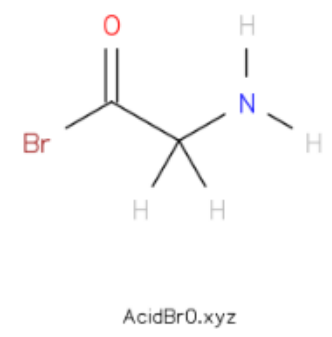
AcidBr1	NCCC(=O)Br	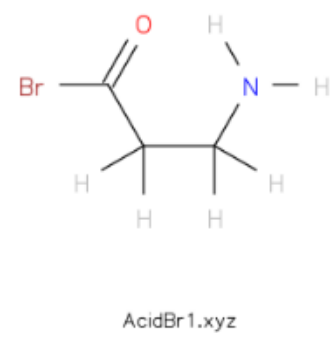
AcidBr2	NCCCC(=O)Br	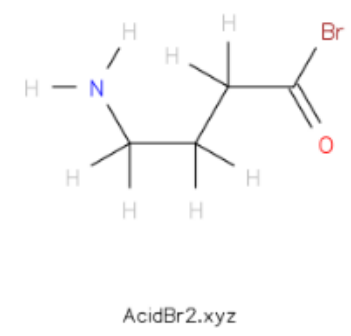
AcidBr3	BrC(=O)CNCC(=O)Br	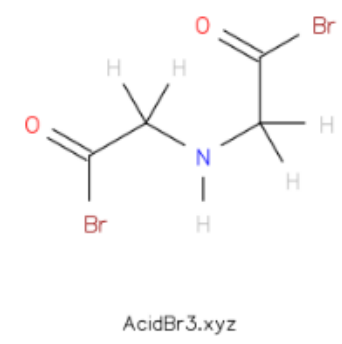
AcidBr4	BrC(=O)CNCCC(=O)Br	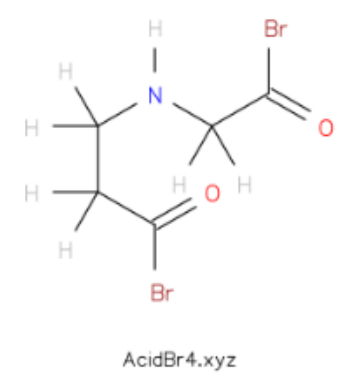
AcidBr5	BrC(=O)CN(CC(=O)Br)CC(=O)Br	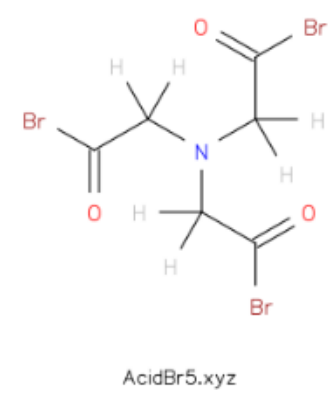
AcidBr6	BrC(=O)CCN(CCC(=O)Br)CCC(=O)Br	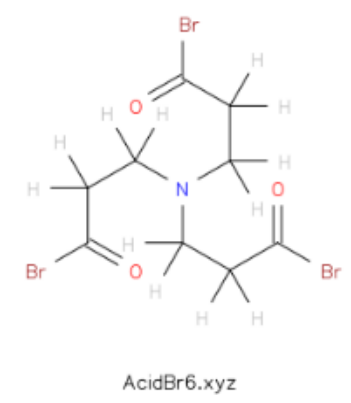

AcidCl0	NCC(=O)Cl	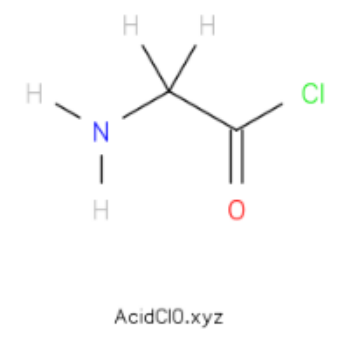
AcidCl1	NCCC(=O)Cl	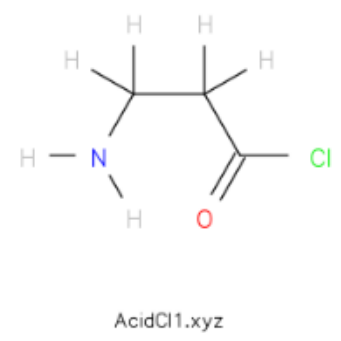
AcidCl2	NCCCC(=O)Cl	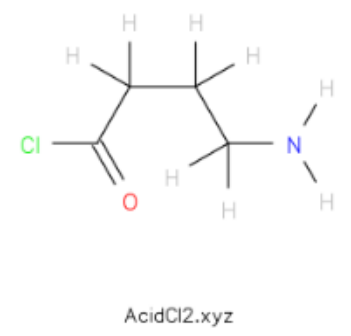
AcidCl3	ClC(=O)CNCC(=O)Cl	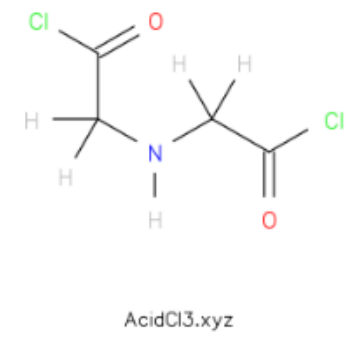
AcidCl4	ClC(=O)CNCCC(=O)Cl	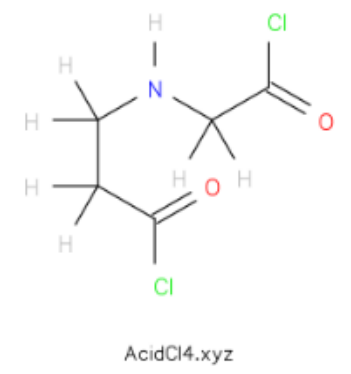
AcidCl5	ClC(=O)CN(CC(=O)Cl)CC(=O)Cl	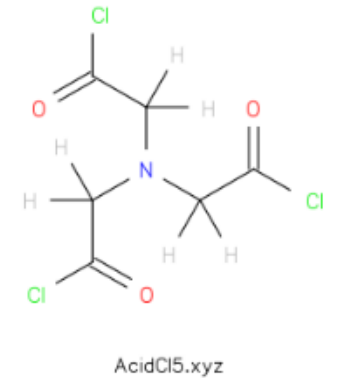
AcidCl6	ClC(=O)CCN(CCC(=O)Cl)CCC(=O)Cl	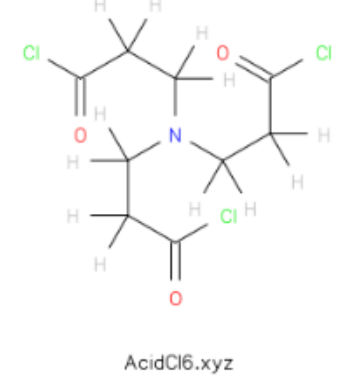

AcidF0	NCC(=O)F	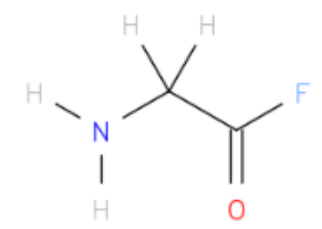<br>AcidF0.xyz
AcidF1	NCCC(=O)F	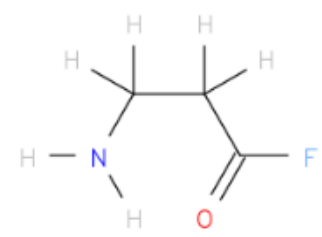<br>AcidF1.xyz
AcidF2	NCCCC(=O)F	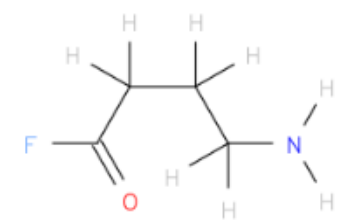<br>AcidF2.xyz
AcidF3	FC(=O)CNCC(=O)F	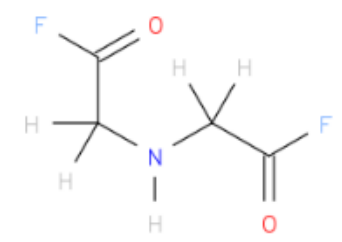<br>AcidF3.xyz
AcidF4	FC(=O)CNCCC(=O)F	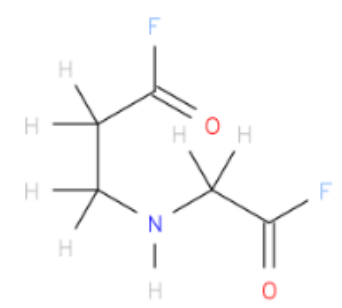<br>AcidF4.xyz
AcidF5	FC(=O)CN(CC(=O)F)CC(=O)F	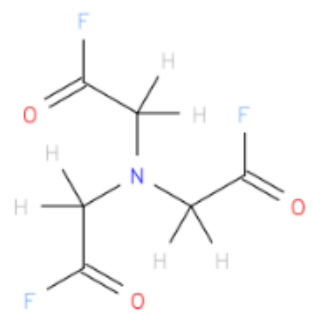<br>AcidF5.xyz
AcidF6	FC(=O)CCN(CCC(=O)F)CCC(=O)F	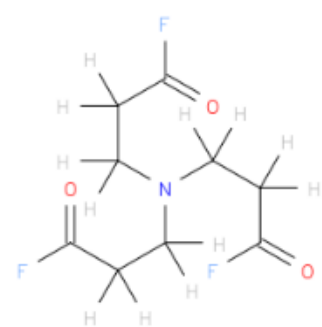<br>AcidF6.xyz

AcidI0	NCC(=O)I	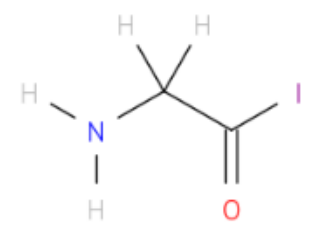 AcidI0.xyz
AcidI1	NCCC(=O)I	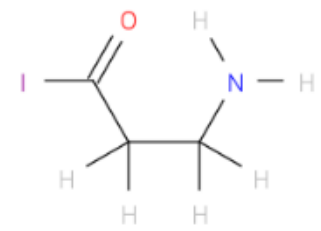 AcidI1.xyz
AcidI2	NCCCC(=O)I	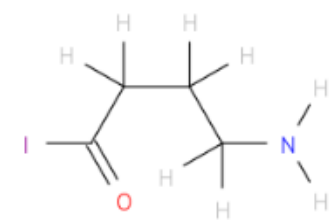 AcidI2.xyz
AcidI3	IC(=O)CNCC(=O)I	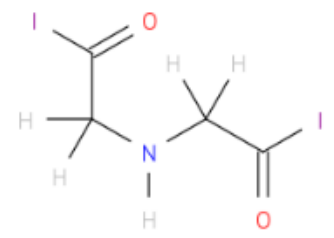 AcidI3.xyz
AcidI4	IC(=O)CNCCC(=O)I	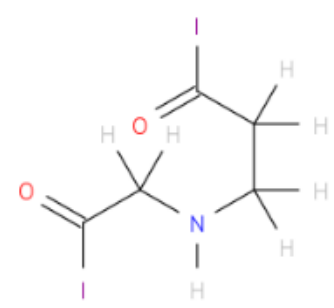 AcidI4.xyz
AcidI5	IC(=O)CN(CC(=O)I)CC(=O)I	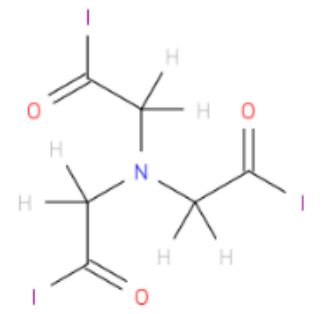 AcidI5.xyz
AcidI6	IC(=O)CCN(CCC(=O)I)CCC(=O)I	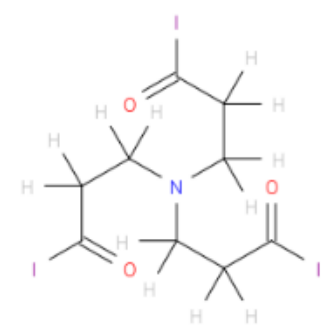 AcidI6.xyz

Acid_Br3V2	NCCCCC(=O)Br	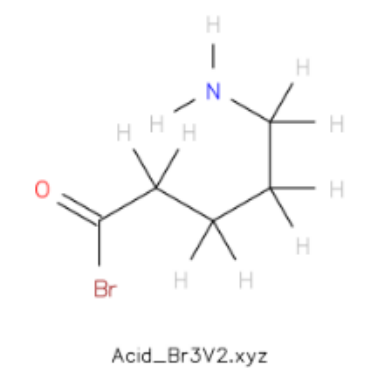
Acid_Br4V2	NCCCCCC(=O)Br	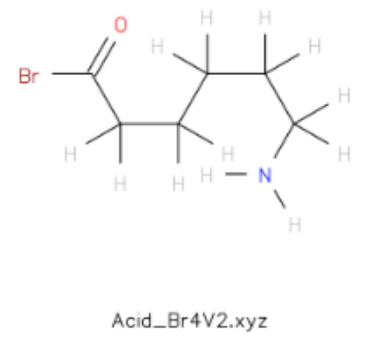
Acid_Br5V2	NCCCCCCC(=O)Br	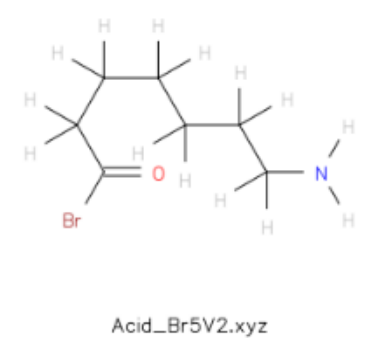
Acid_Br6V2	NCCCCCCCC(=O)Br	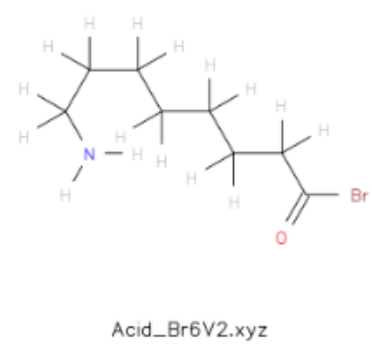
Acid_Br7V2	NCCCCCCCCC(=O)Br	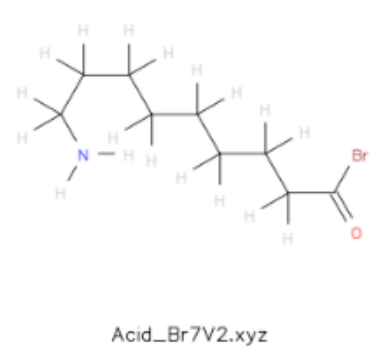
Acid_Br8V2	NCCCCCCCCCC(=O)Br	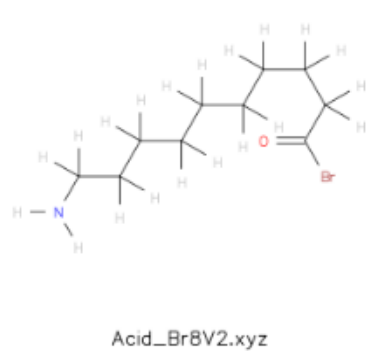
Acid_Cl3V2	NCCCCC(=O)Cl	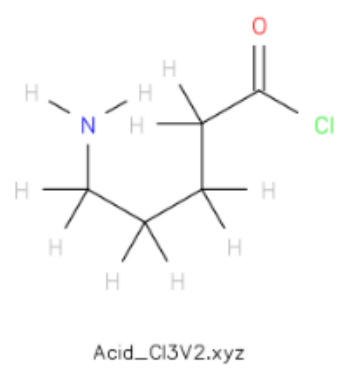

Acid_Cl4V2	NCCCCC(=O)Cl	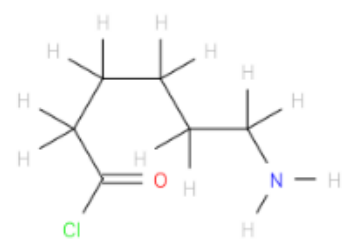 Acid_Cl4V2.xyz
Acid_Cl5V2	NCCCCCC(=O)Cl	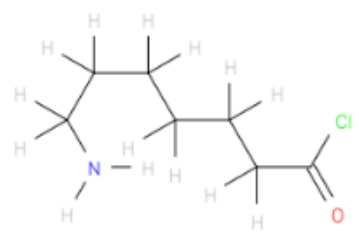 Acid_Cl5V2.xyz
Acid_Cl6V2	NCCCCCCC(=O)Cl	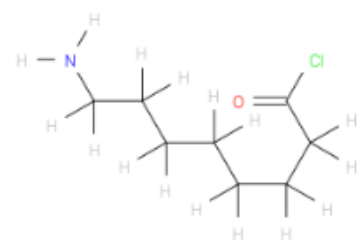 Acid_Cl6V2.xyz
Acid_Cl7V2	NCCCCCCCC(=O)Cl	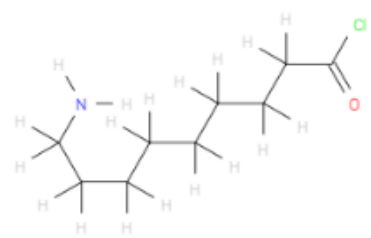 Acid_Cl7V2.xyz
Acid_Cl8V2	NCCCCCCCCC(=O)Cl	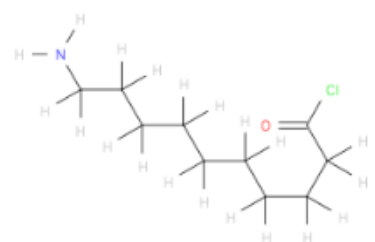 Acid_Cl8V2.xyz
Acid_Halid3V2	NCCCC(=O)F	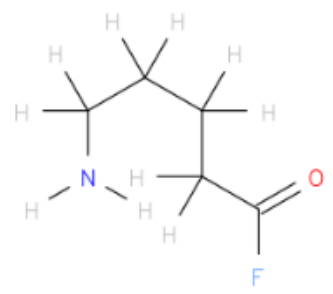 Acid_Halid3V2.xyz
Acid_Halid4V2	NCCCCC(=O)F	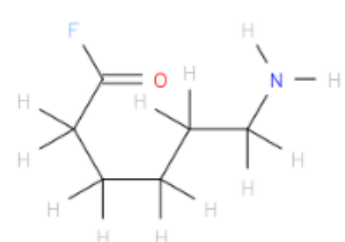 Acid_Halid4V2.xyz

Acid_Halid5V2	NCCCCCCC(=O)F	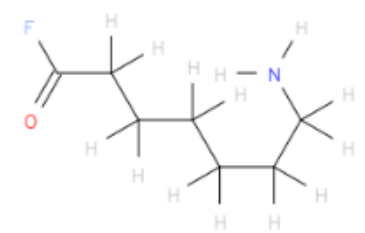 Acid_Halid5V2.xyz
Acid_Halid6V2	NCCCCCCCC(=O)F	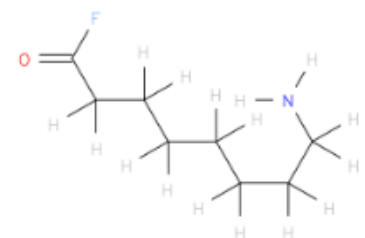 Acid_Halid6V2.xyz
Acid_Halid7V2	NCCCCCCCCC(=O)F	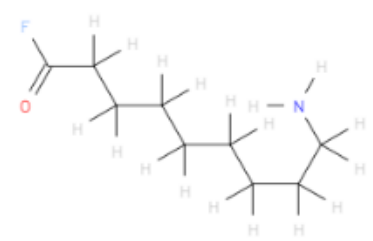 Acid_Halid7V2.xyz
Acid_Halid8V2	NCCCCCCCCCC(=O)F	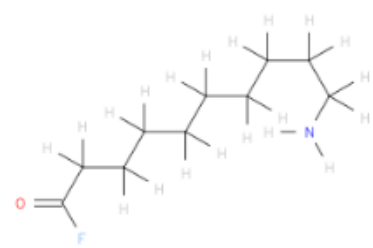 Acid_Halid8V2.xyz
Acid_I3V2	NCCCCC(=O)I	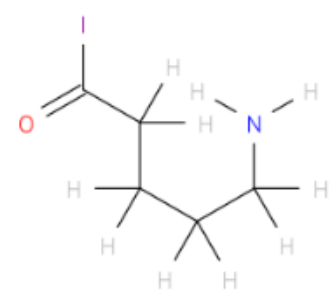 Acid_I3V2.xyz
Acid_I4V2	NCCCCCC(=O)I	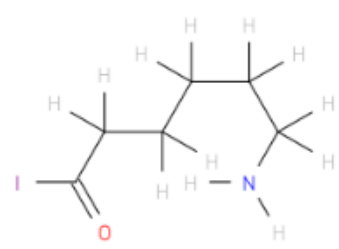 Acid_I4V2.xyz
Acid_I5V2	NCCCCCCC(=O)I	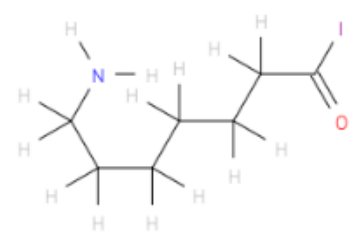 Acid_I5V2.xyz

Acid_I6V2	NCCCCCCCC(=O)I	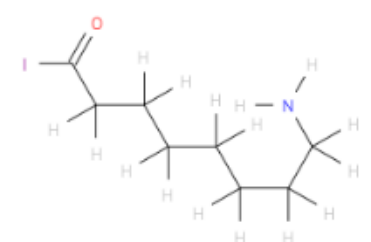 Acid_I6V2.xyz
Acid_I7V2	NCCCCCCCCC(=O)I	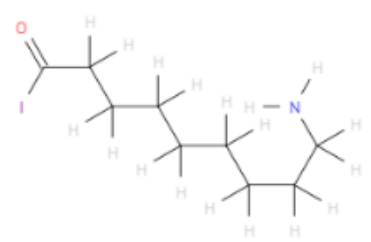 Acid_I7V2.xyz
Acid_I8V2	NCCCCCCCCCC(=O)I	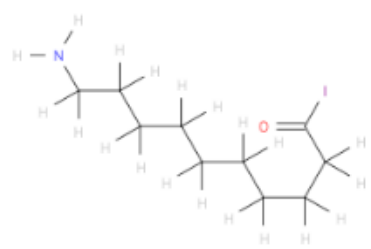 Acid_I8V2.xyz
Acid_Sulf0	NCCS([O])([O])O	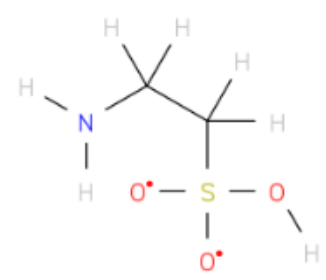 Acid_Sulf0.xyz
Acid_Sulf1	NCCCS([O])([O])O	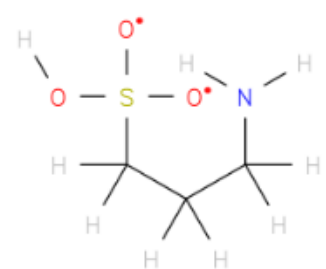 Acid_Sulf1.xyz
Acid_Sulf2	NCCCCS([O])([O])O	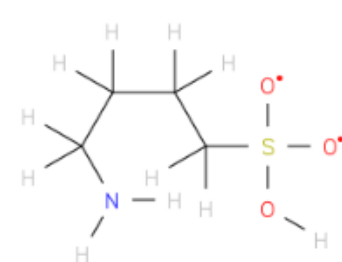 Acid_Sulf2.xyz
Acid_Sulf30	OS([O])([O])CCNCCS([O])([O])O	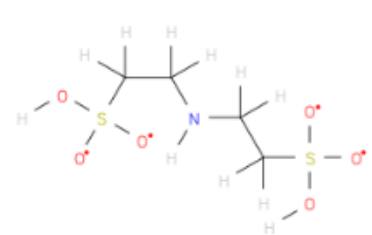 Acid_Sulf30.xyz

Acid_Sulf3V2	NCCCCCS([O])([O])O	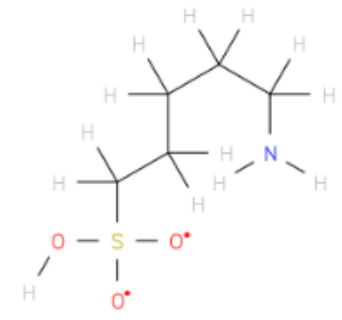<br>Acid_Sulf3V2.xyz
Acid_Sulf430	OS([O])([O])CCN(CCS([O])([O])O)CCS([O])([O])O	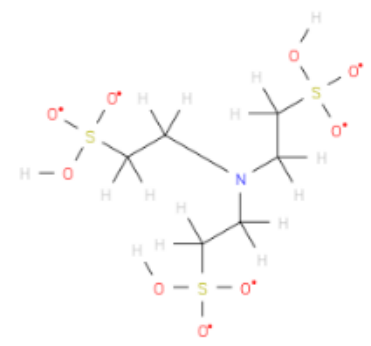<br>Acid_Sulf430.xyz
Acid_Sulf4V2	NCCCCCCS([O])([O])O	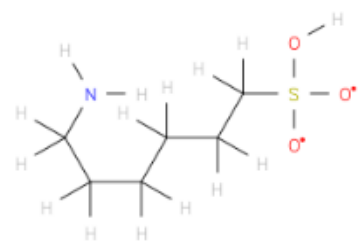<br>Acid_Sulf4V2.xyz
Acid_Sulf50	OS([O])([O])CCNCCCS([O])([O])O	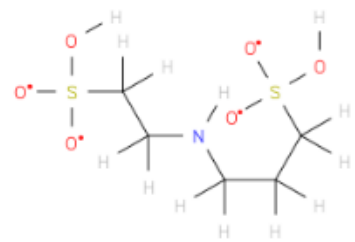<br>Acid_Sulf50.xyz
Acid_Sulf5V2	NCCCCCCCS([O])([O])O	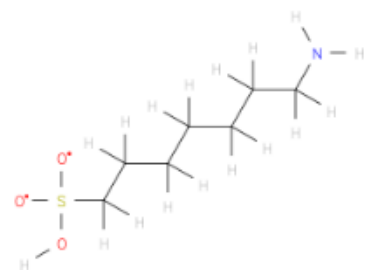<br>Acid_Sulf5V2.xyz
Acid_Sulf671	OS([O])([O])CCCN(CCCS([O])([O])O)CCCS([O])([O])O	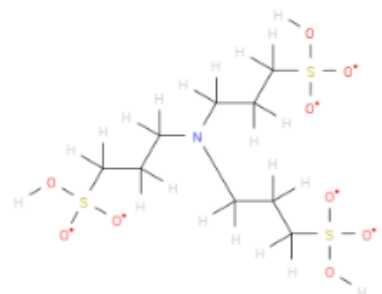<br>Acid_Sulf671.xyz
Acid_Sulf6V2	NCCCCCCCCS([O])([O])O	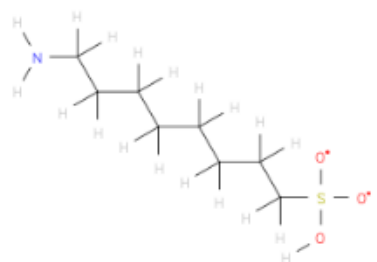<br>Acid_Sulf6V2.xyz

Acid_Sulf7V2	NCCCCCCCCCS([O])([O])O	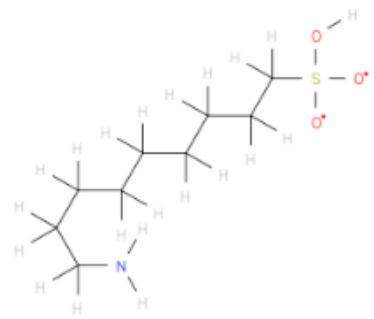 Acid_Sulf7V2.xyz
Acid_Sulf8V2	NCCCCCCCCCCS([O])([O])O	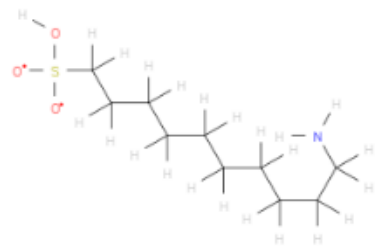 Acid_Sulf8V2.xyz
Acid_Tio0	NCC(=O)S	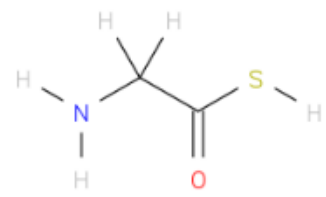 Acid_Tio0.xyz
Acid_Tio1	NCCC(=O)S	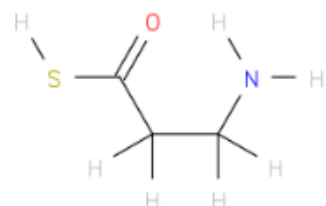 Acid_Tio1.xyz
Acid_Tio2	NCCCC(=O)S	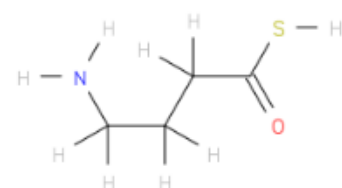 Acid_Tio2.xyz
Acid_Tio30	SC(=O)CNCC(=O)S	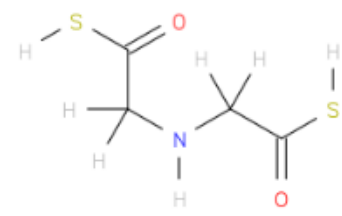 Acid_Tio30.xyz
Acid_Tio3V2	NCCCCC(=O)S	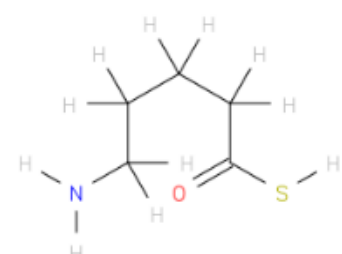 Acid_Tio3V2.xyz

Acid_Tio430	SC(=O)CN(CC(=O)S)CC(=O)S	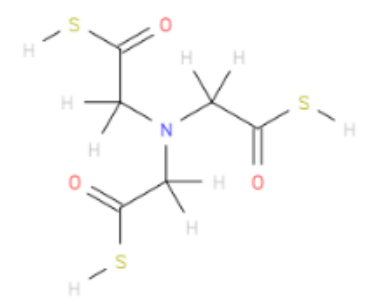 Acid_Tio430.xyz
Acid_Tio4V2	NCCCCCC(=O)S	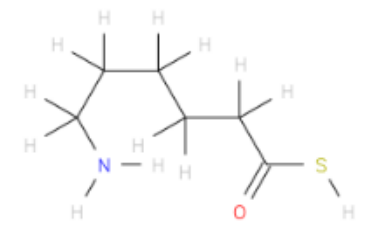 Acid_Tio4V2.xyz
Acid_Tio50	SC(=O)CNCCC(=O)S	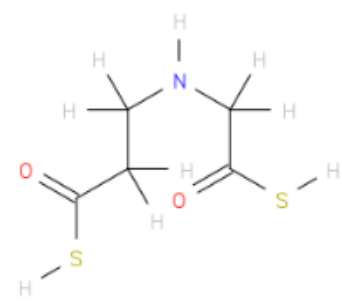 Acid_Tio50.xyz
Acid_Tio5V2	NCCCCCCC(=O)S	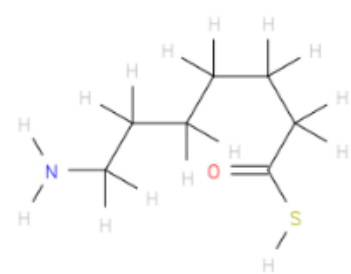 Acid_Tio5V2.xyz
Acid_Tio671	SC(=O)CCN(CCC(=O)S)CCC(=O)S	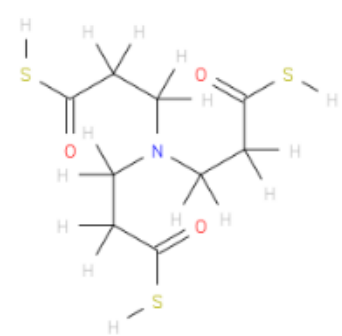 Acid_Tio671.xyz
Acid_Tio6V2	NCCCCCCCC(=O)S	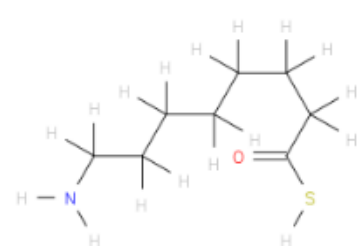 Acid_Tio6V2.xyz
Acid_Tio7V2	NCCCCCCCCC(=O)S	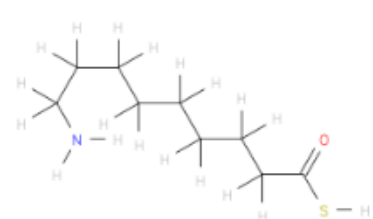 Acid_Tio7V2.xyz

Acid_Tio8V2	NCCCCCCCCCC(=O)S	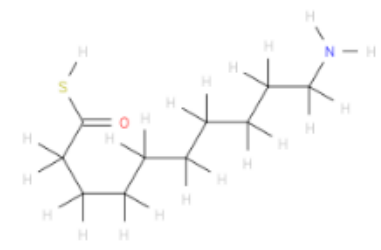 Acid_Tio8V2.xyz
Alcohol0	NCCO	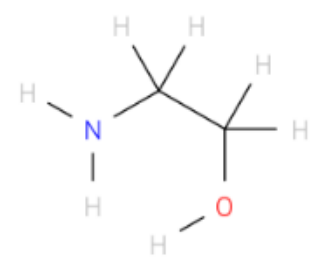 Alcohol0.xyz
Alcohol1	NCCCO	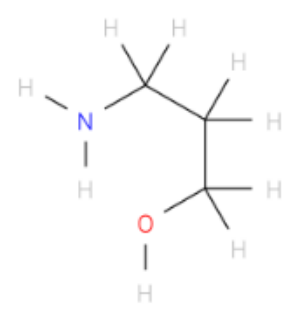 Alcohol1.xyz
Alcohol2	NCCCCO	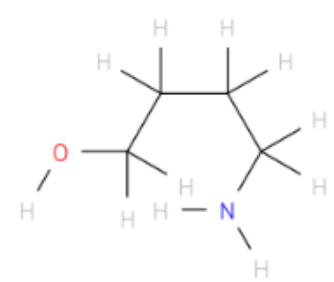 Alcohol2.xyz
Alcohol30	C(O)CNCCO	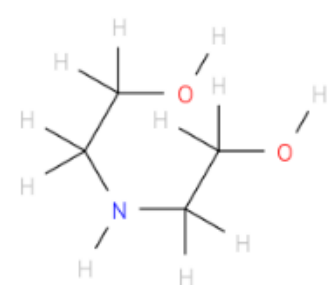 Alcohol30.xyz
Alcohol3V2	NCCCCCO	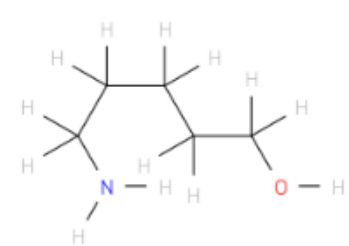 Alcohol3V2.xyz
Alcohol430	C(O)CN(CCO)CCO	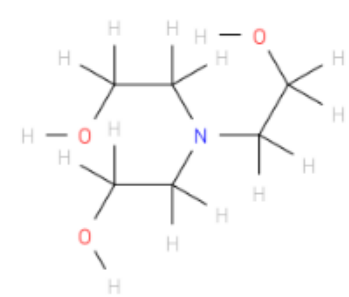 Alcohol430.xyz

Alcohol4V2	NCCCCCCO	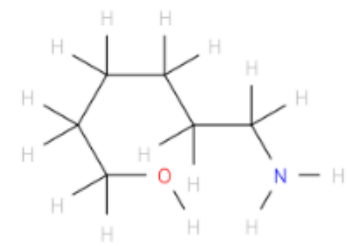 Alcohol4V2.xyz
Alcohol50	C(O)CNCCCO	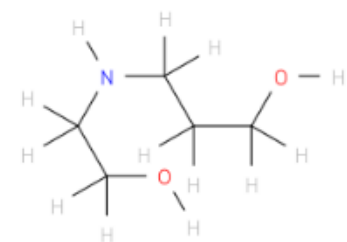 Alcohol50.xyz
Alcohol5V2	NCCCCCCCO	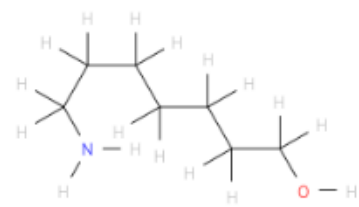 Alcohol5V2.xyz
Alcohol671	C(O)CCN(CCCO)CCCO	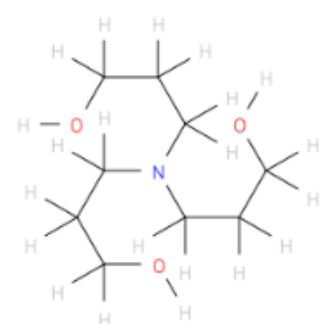 Alcohol671.xyz
Alcohol6V2	NCCCCCCCCO	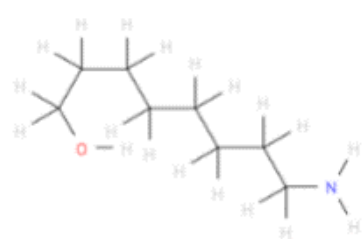 Alcohol6V2.xyz
Alcohol7V2	NCCCCCCCCCO	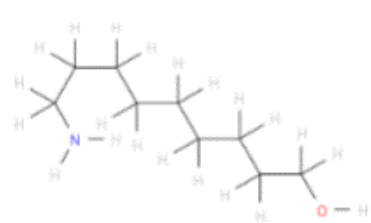 Alcohol7V2.xyz
Alcohol8V2	NCCCCCCCCCCO	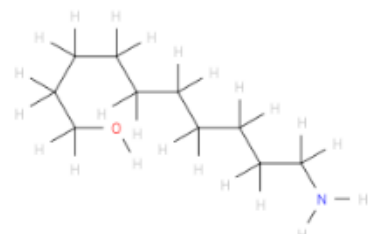 Alcohol8V2.xyz

Aldehyde0	NCC=O	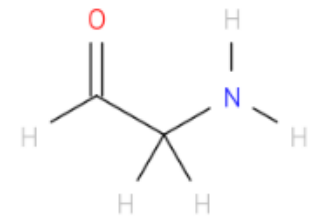 Aldehyde0.xyz
Aldehyde1	NCCC=O	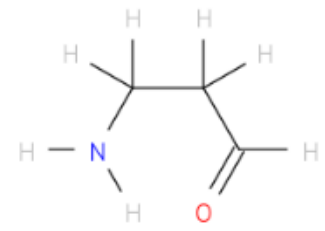 Aldehyde1.xyz
Aldehyde2	NCCCC=O	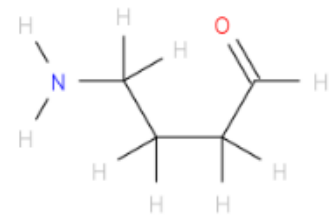 Aldehyde2.xyz
Aldehyde30	O=CCNCC=O	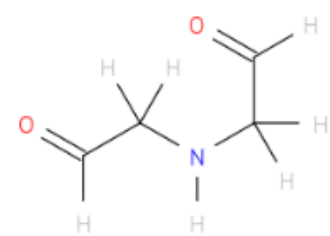 Aldehyde30.xyz
Aldehyde430	O=CCN(CC=O)CC=O	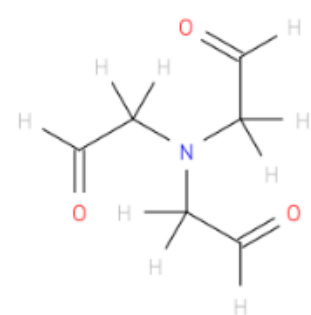 Aldehyde430.xyz
Aldehyde50	O=CCNCCC=O	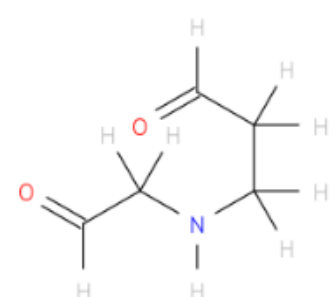 Aldehyde50.xyz
Aldehyde671	O=CCCN(CCC=O)CCC=O	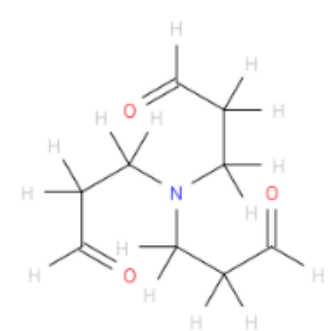 Aldehyde671.xyz

Amide0	NCC(=O)N	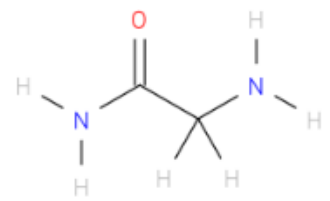 Amide0.xyz
Amide1	NCCC(=O)N	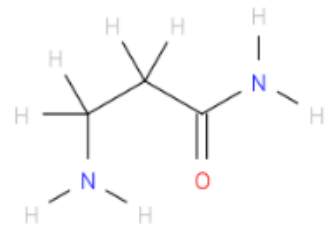 Amide1.xyz
Amide2	NCCCC(=O)N	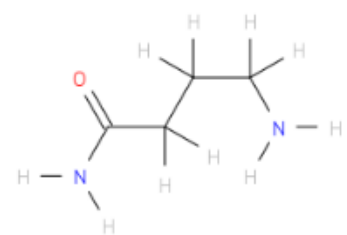 Amide2.xyz
Amide30	NC(=O)CNCC(=O)N	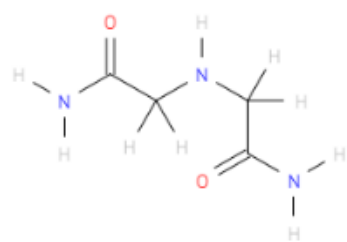 Amide30.xyz
Amide3V2	NCCCCC(=O)N	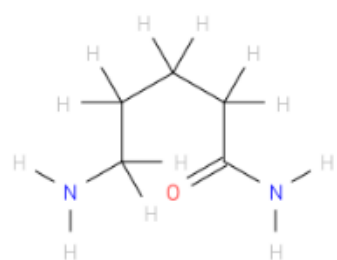 Amide3V2.xyz
Amide430	NC(=O)CN(CC(=O)N)CC(=O)N	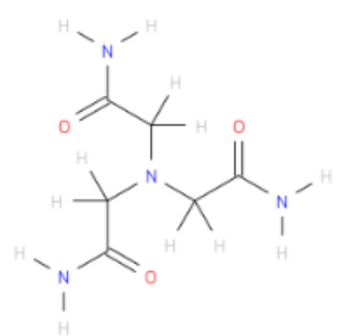 Amide430.xyz
Amide4V2	NCCCCCC(=O)N	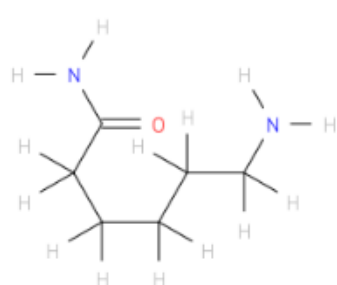 Amide4V2.xyz

Amide50	NC(=O)CNCCC(=O)N	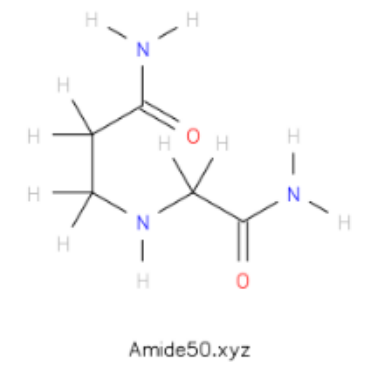
Amide5V2	NCCCCCCC(=O)N	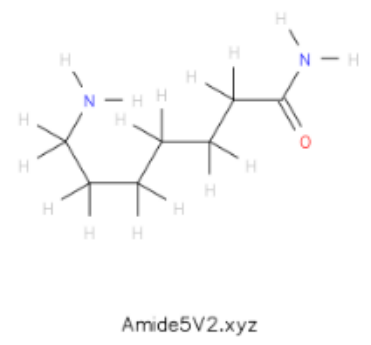
Amide671	NC(=O)CCN(CCC(=O)N)<br>CCC(=O)N	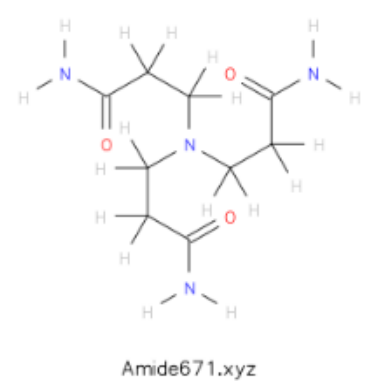
Amide6V2	NCCCCCCCC(=O)N	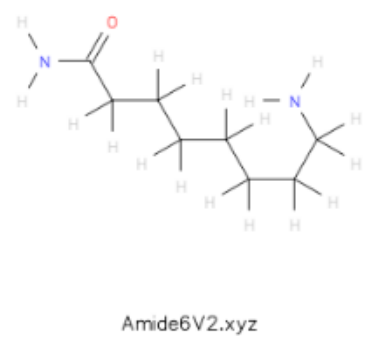
Amide7V2	NCCCCCCCCC(=O)N	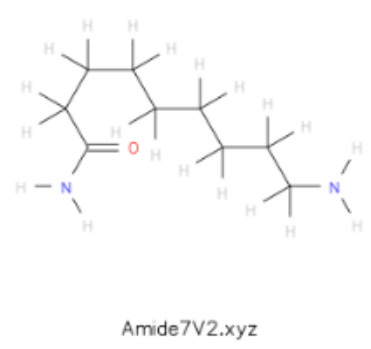
Amide8V2	NCCCCCCCCCC(=O)N	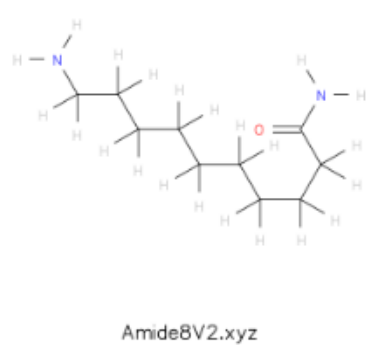
Amine0	NCCN	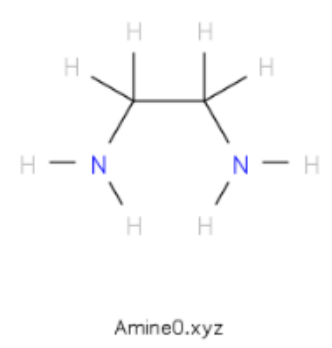

Amine1	NCCCN	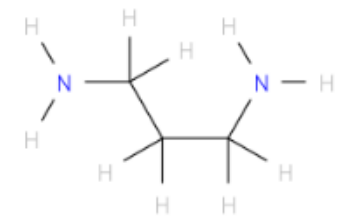 Amine1.xyz
Amine2	NCCCCN	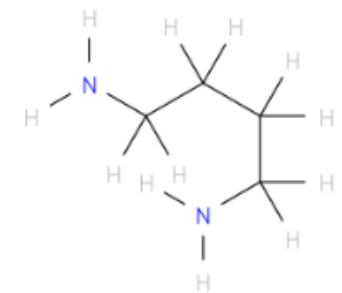 Amine2.xyz
Amine30	C(N)CNCCN	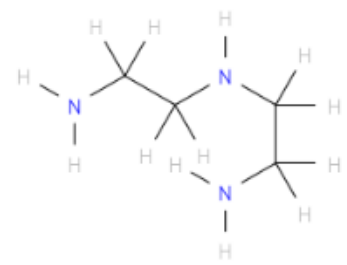 Amine30.xyz
Amine3V2	NCCCCCN	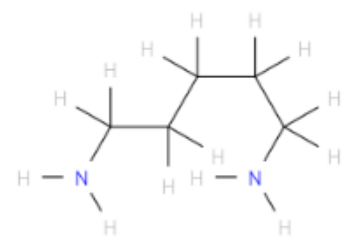 Amine3V2.xyz
Amine430	C(N)CN(CCN)CCN	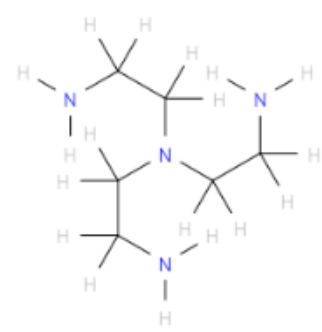 Amine430.xyz
Amine4V2	NCCCCCCN	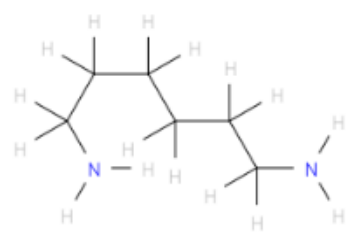 Amine4V2.xyz
Amine50	C(N)CNCCCN	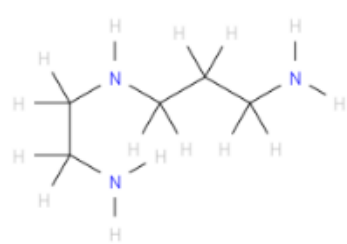 Amine50.xyz

Amine5V2	NCCCCCCCN	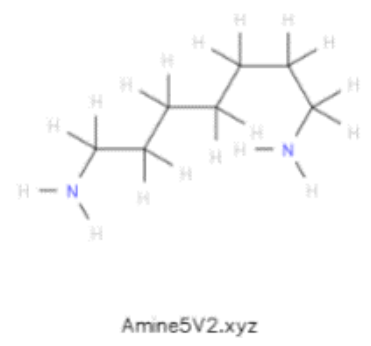
Amine671	C(N)CCN(CCCN)CCCN	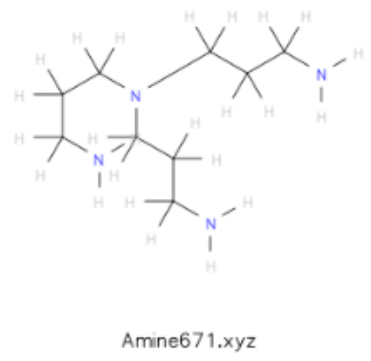
Amine6V2	NCCCCCCCCN	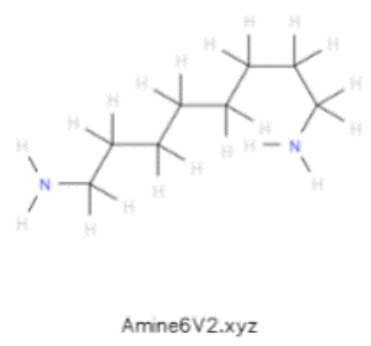
Amine7V2	NCCCCCCCCCN	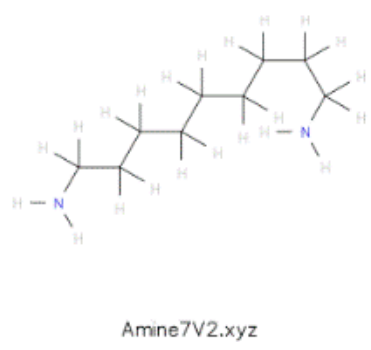
Amine8V2	NCCCCCCCCCCN	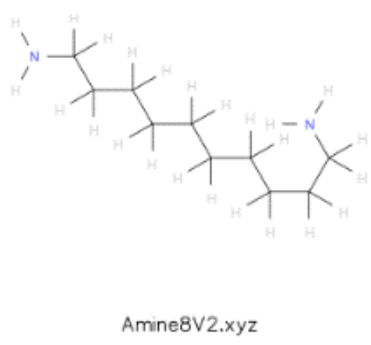
Anhydride0	NCC(=O)OC(=O)C	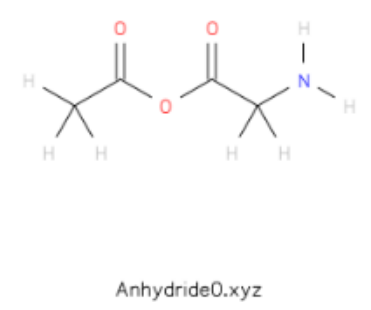
Anhydride1	NCCC(=O)OC(=O)C	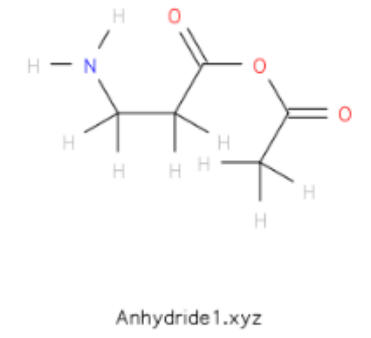

Anhydride2	NCCCC(=O)OC(=O)C	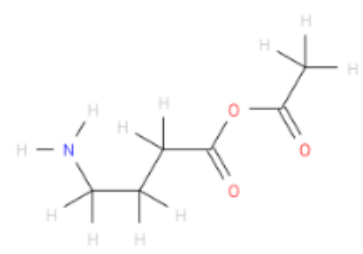 Anhydride2.xyz
Anhydride30	C(=O)OC(=O)CNCC(=O)OC(=O)C	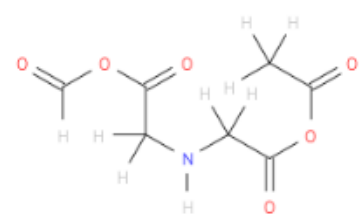 Anhydride30.xyz
Anhydride3V2	NCCCCC(=O)OC(=O)C	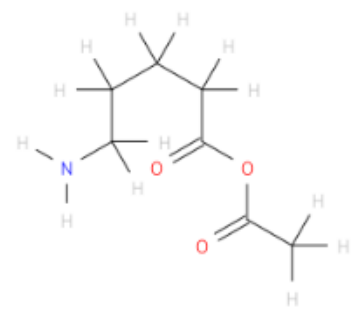 Anhydride3V2.xyz
Anhydride430	C(=O)OC(=O)CN(CC(=O)OC(=O)C)CC(=O)OC(=O)C	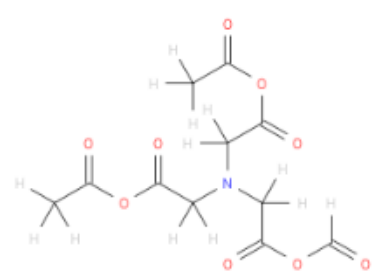 Anhydride430.xyz
Anhydride4V2	NCCCCCC(=O)OC(=O)C	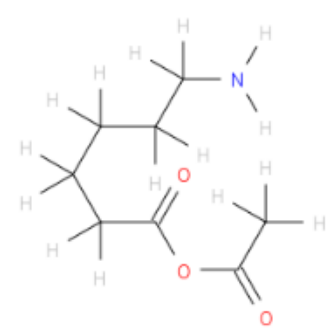 Anhydride4V2.xyz
Anhydride50	C(=O)OC(=O)CNCCC(=O)OC(=O)C	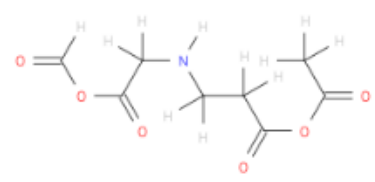 Anhydride50.xyz
Anhydride5V2	NCCCCCCC(=O)OC(=O)C	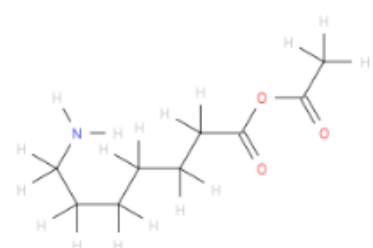 Anhydride5V2.xyz

Anhydride671	C(=O)OC(=O)CCN(CCC(=O)OC(=O)C)CCC(=O)OC(=O)C	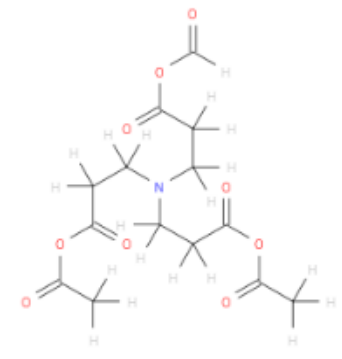 Anhydride671.xyz
Anhydride7V2	NCCCCCCCCC(=O)OC(=O)C	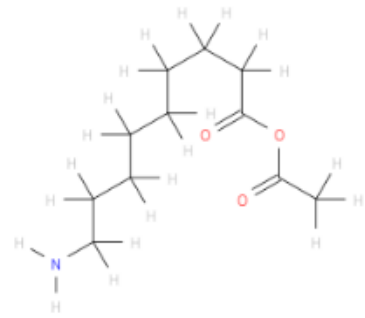 Anhydride7V2.xyz
Anhydride8V2	NCCCCCCCCCC(=O)OC(=O)C	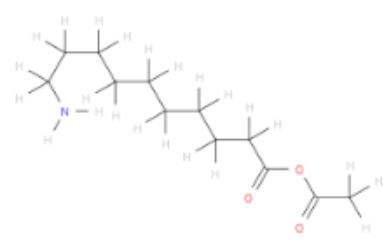 Anhydride8V2.xyz
AzolidineBr1	N1[C@H](CCC1)Br	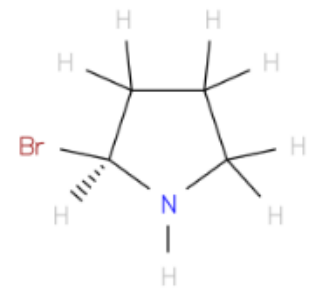 31268
AzolidineBr2	N1C[C@H](CC1)Br	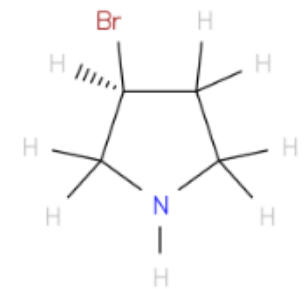 31268
AzolidineC#N1	N1[C@H](CCC1)[C][N]	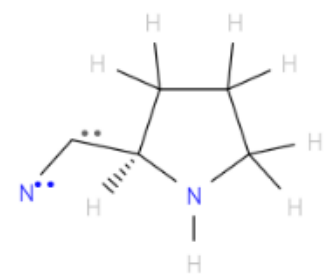 31268
AzolidineC#N2	N1C[C@H](CC1)C#N	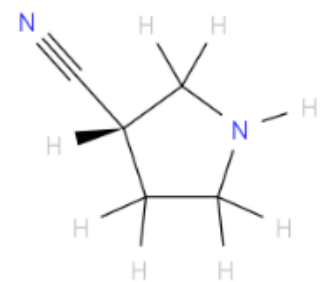 31268

AzolidineCl1	N1[C@H](CCC1)Cl	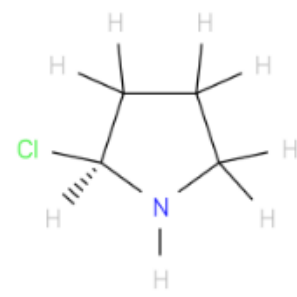 31268
AzolidineCl2	N1C[C@H](CC1)Cl	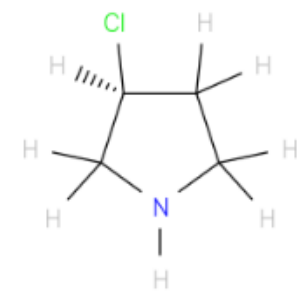 31268
AzolidineF1	N1[C@H](CCC1)F	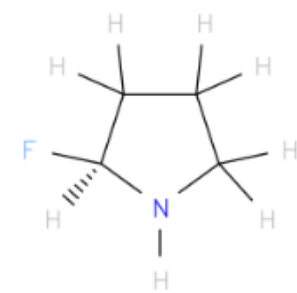 31268
AzolidineF2	N1C[C@H](CC1)F	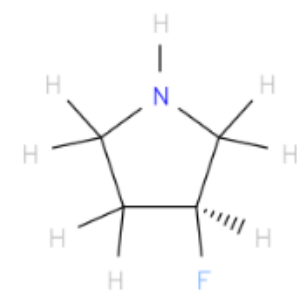 31268
AzolidineI1	N1[C@H](CCC1)I	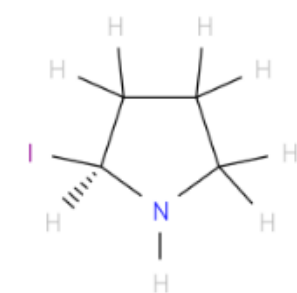 31268
AzolidineI2	N1C[C@H](CC1)I	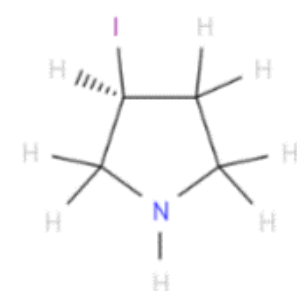 31268
AzolidineN1	N1[C@H](CCC1)N	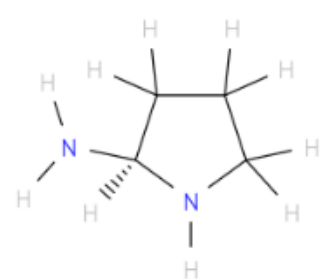 31268

AzolidineN2	N1C[C@H](CC1)N	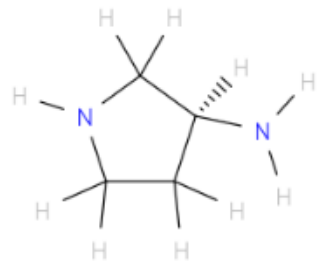 31268
AzolidineNO2_1	N1[C@H](CCC1)N(=O)=O	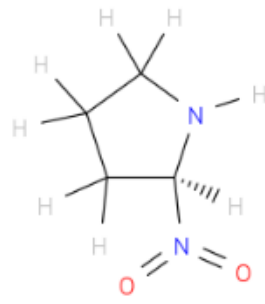 31268
AzolidineNO2_2	N1C[C@H](CC1)N(=O)=O	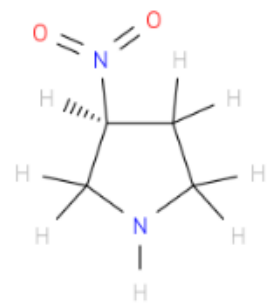 31268
AzolidineO1	N1[C@H](CCC1)O	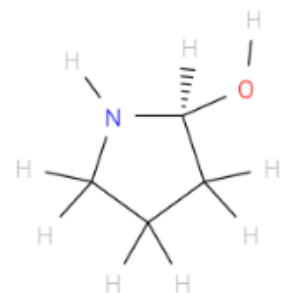 31268
AzolidineO2	N1C[C@H](CC1)O	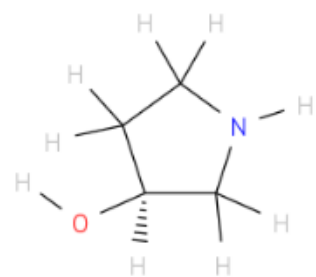 31268
AzolidineS1	N1[C@H](CCC1)S	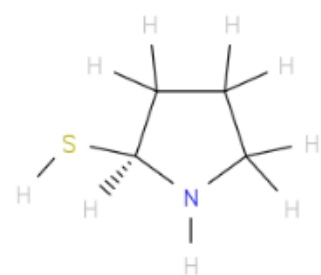 31268
AzolidineS2	N1C[C@H](CC1)S	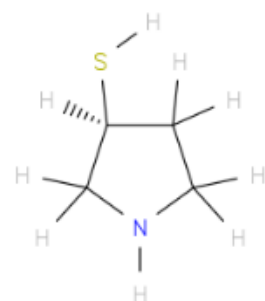 31268

Br0	NCBr	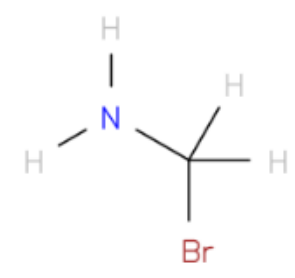 Br0.xyz
Br1	NCCBr	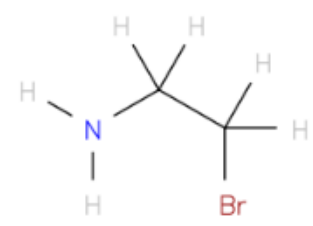 Br1.xyz
Br2	NCCCBr	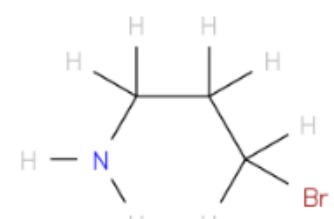 Br2.xyz
Br3	BrCNCBr	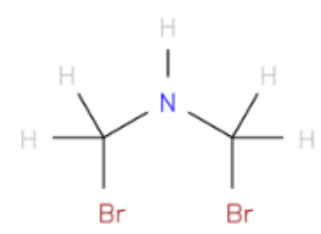 Br3.xyz
Br3V2	NCCCCBr	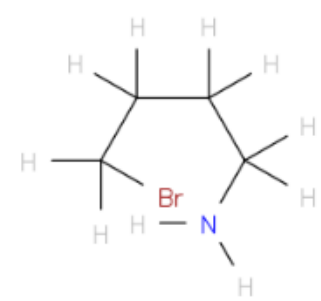 Br3V2.xyz
Br4	BrCNCCBr	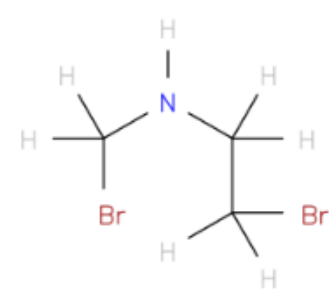 Br4.xyz
Br4V2	NCCCCCBr	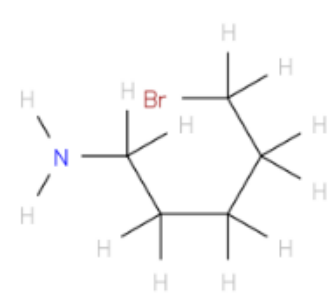 Br4V2.xyz

Br5	BrCN(CBr)CBr	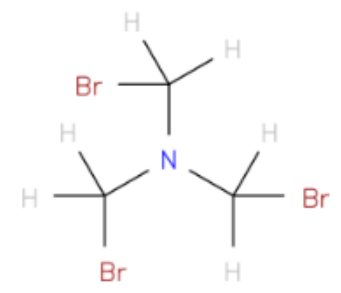 Br5.xyz
Br5V2	NCCCCCCBr	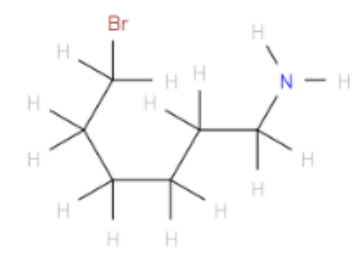 Br5V2.xyz
Br6	BrCCN(CCBr)CCBr	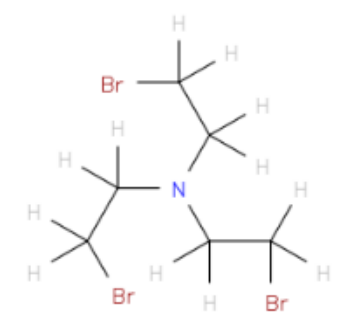 Br6.xyz
Br6V2	NCCCCCCCBr	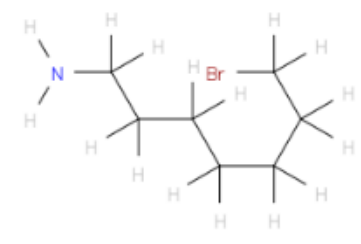 Br6V2.xyz
Br7V2	NCCCCCCCCBr	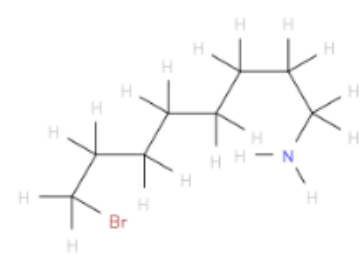 Br7V2.xyz
Br8V2	NCCCCCCCCCBr	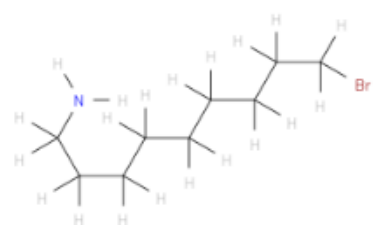 Br8V2.xyz
Cl0	NCCl	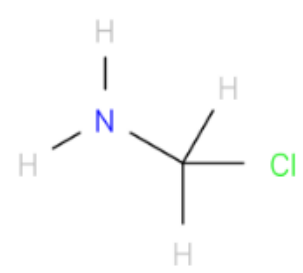 Cl0.xyz

Cl1	NCCCl	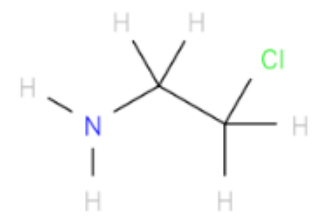 Cl1.xyz
Cl2	NCCCCl	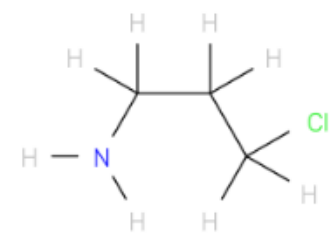 Cl2.xyz
Cl3	ClCNCCl	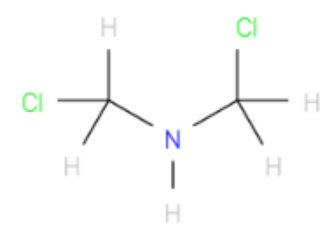 Cl3.xyz
Cl3V2	NCCCCCl	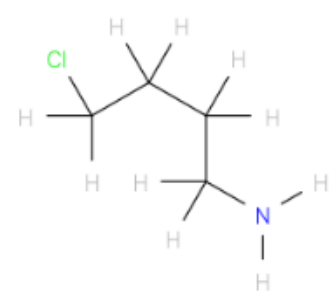 Cl3V2.xyz
Cl4	ClCNCCCl	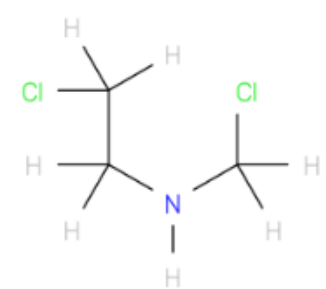 Cl4.xyz
Cl4V2	NCCCCCCl	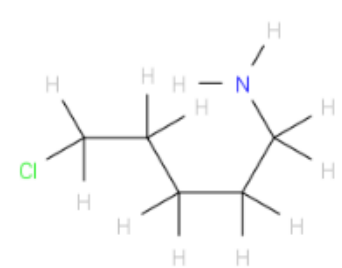 Cl4V2.xyz
Cl5	ClCN(CCl)CCl	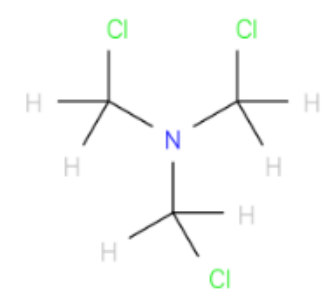 Cl5.xyz

Cl5V2	NCCCCCCCl	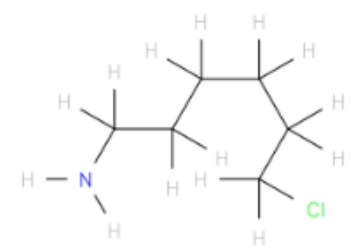<br>Cl5V2.xyz
Cl6	ClCCN(CCCl)CCCl	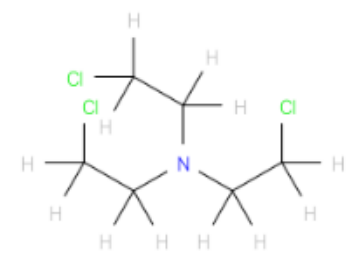<br>Cl6.xyz
Cl6V2	NCCCCCCCCl	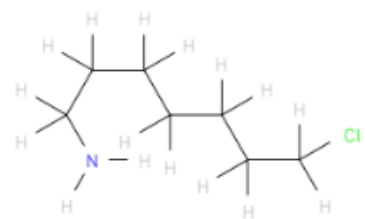<br>Cl6V2.xyz
Cl7V2	NCCCCCCCCCl	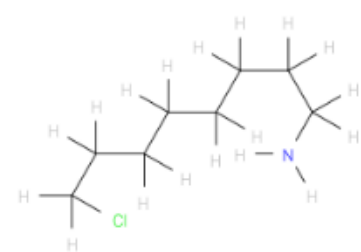<br>Cl7V2.xyz
Cl8V2	NCCCCCCCCCCl	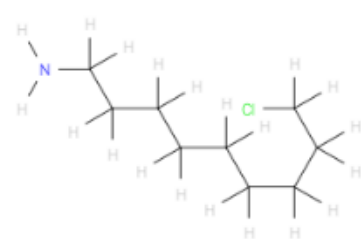<br>Cl8V2.xyz
Cyan0	NC[C][N]	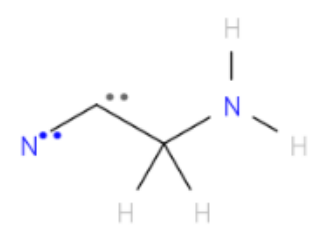<br>Cyan0.xyz
Cyan1	NCC[C][N]	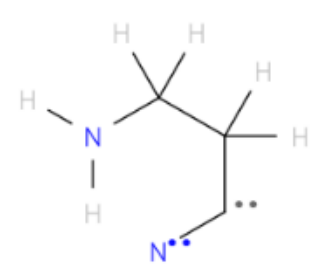<br>Cyan1.xyz

Cyan2	NCCCC#N	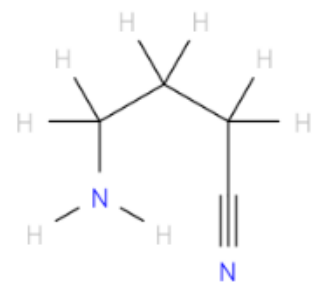 Cyan2.xyz
Cyan30	[N][C]CNC[C][N]	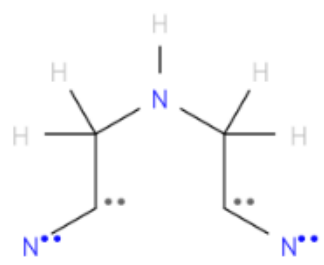 Cyan30.xyz
Cyan3V2	NCCCCC#N	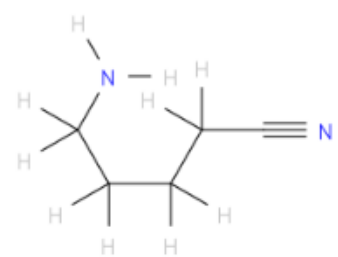 Cyan3V2.xyz
Cyan430	N#CCN(CC#N)CC#N	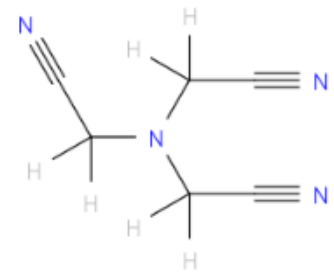 Cyan430.xyz
Cyan4V2	NCCCCCC#N	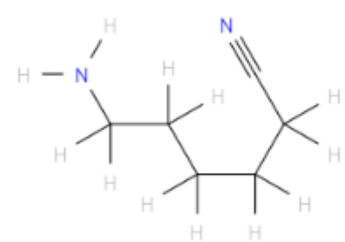 Cyan4V2.xyz
Cyan50	N#CCNCC[C][N]	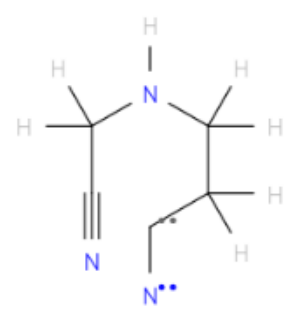 Cyan50.xyz
Cyan5V2	NCCCCCC[C][N]	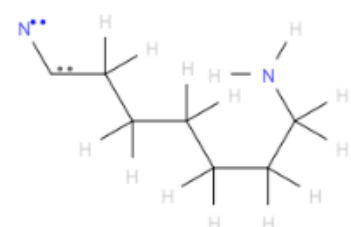 Cyan5V2.xyz

Cyan671	[N][C]CCN(CCC#N)CC[C][N]	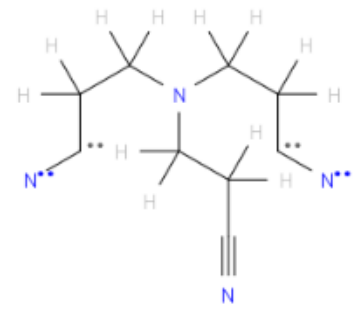 Cyan671.xyz
Cyan6V2	NCCCCCCCC#N	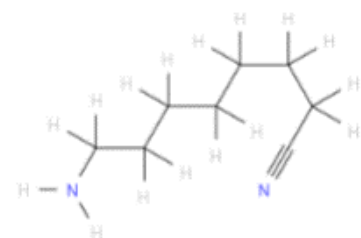 Cyan6V2.xyz
Cyan7V2	NCCCCCCCC[C][N]	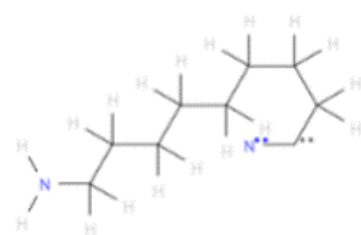 Cyan7V2.xyz
Cyan8V2	NCCCCCCCCC[C][N]	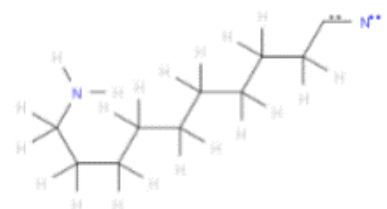 Cyan8V2.xyz
Ester0	NCC(=O)OC	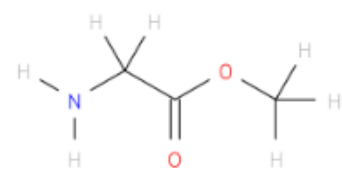 Ester0.xyz
Ester1	NCCC(=O)OC	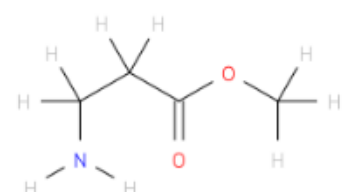 Ester1.xyz
Ester2	NCCCC(=O)OC	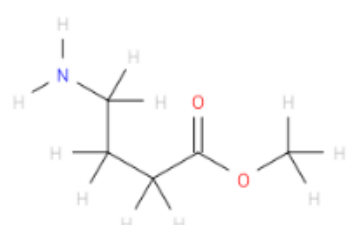 Ester2.xyz

Ester30	C(=O)OCCNCC(=O)OC	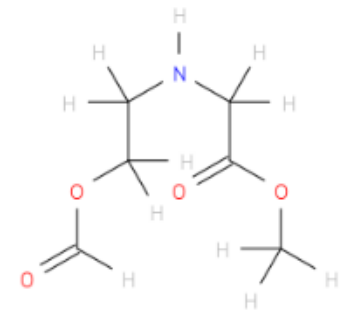 Ester30.xyz
Ester3V2	NCCCCC(=O)OC	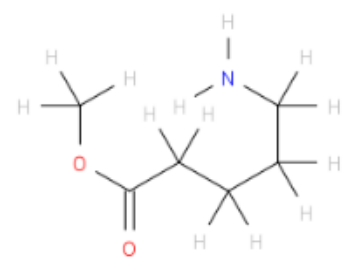 Ester3V2.xyz
Ester430	C(=O)OCCN(CC(=O)OC)CC(=O)OC	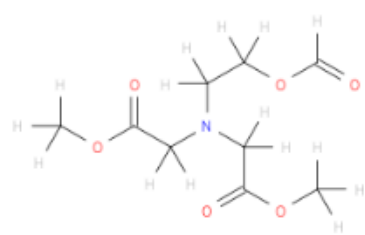 Ester430.xyz
Ester4V2	NCCCCCC(=O)OC	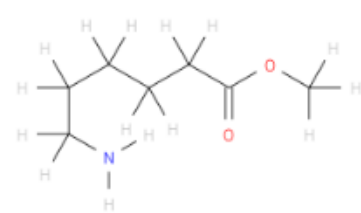 Ester4V2.xyz
Ester50	C(=O)OCCNCCC(=O)OC	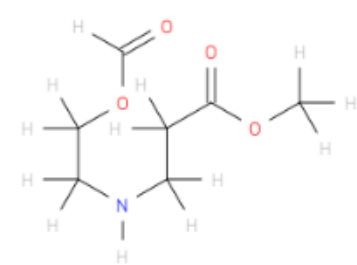 Ester50.xyz
Ester5V2	NCCCCCCC(=O)OC	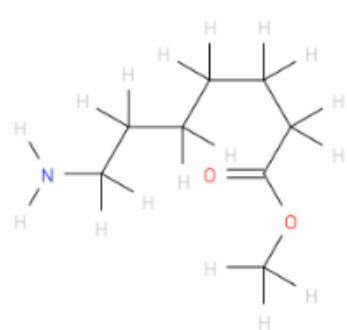 Ester5V2.xyz
Ester671	C(=O)OCCCN(CCC(=O)OC)CCC(=O)OC	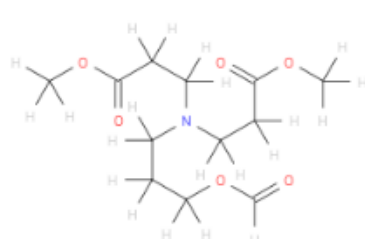 Ester671.xyz

Ester6V2	NCCCCCCCC(=O)OC	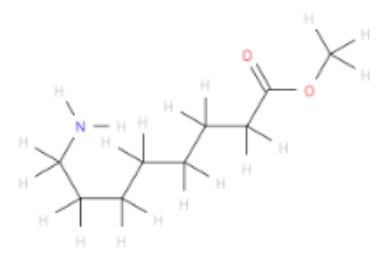 Ester6V2.xyz
Ester7V2	NCCCCCCCCC(=O)OC	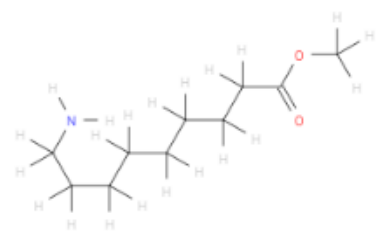 Ester7V2.xyz
Ester8V2	NCCCCCCCCCC(=O)OC	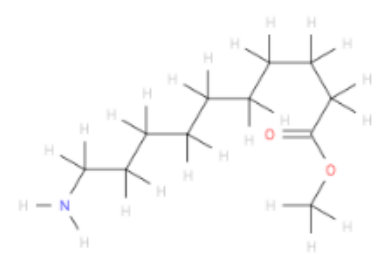 Ester8V2.xyz
Ether0	NCOC	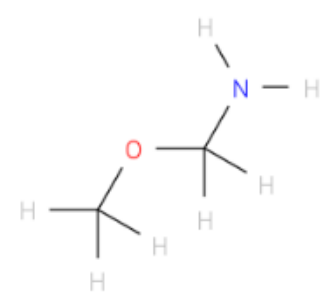 Ether0.xyz
Ether1	NCCOC	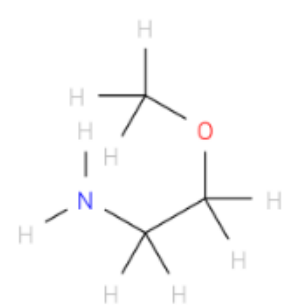 Ether1.xyz
Ether2	NCCCOC	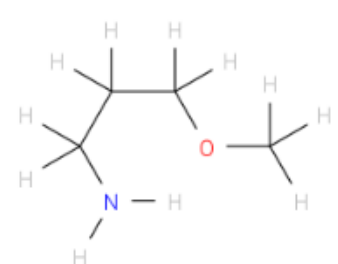 Ether2.xyz
Ether30	COCNCOC	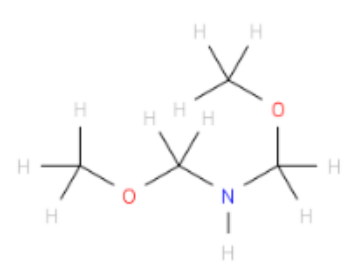 Ether30.xyz

Ether3V2	NCCCCOC	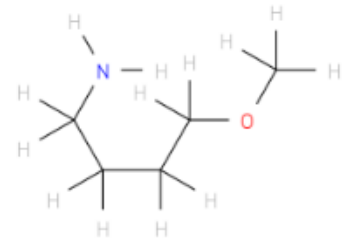 Ether3V2.xyz
Ether430	COCN(COC)COC	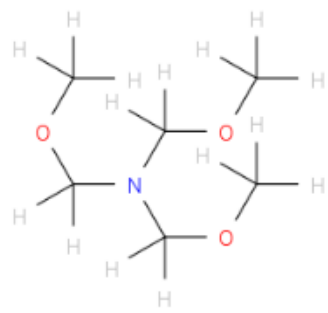 Ether430.xyz
Ether4V2	NCCCCCOC	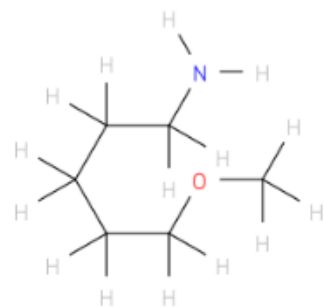 Ether4V2.xyz
Ether50	COCNCCOC	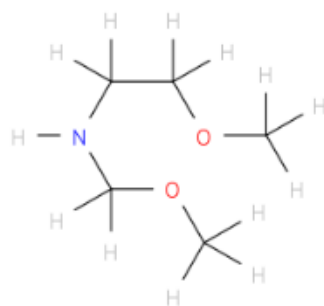 Ether50.xyz
Ether5V2	NCCCCCCOC	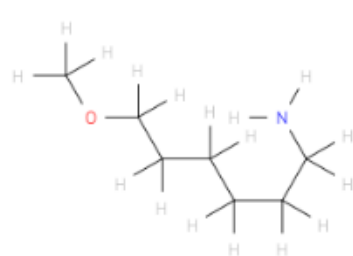 Ether5V2.xyz
Ether671	COCCN(CCOC)CCOC	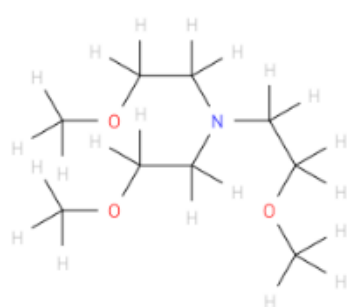 Ether671.xyz
Ether6V2	NCCCCCCCOC	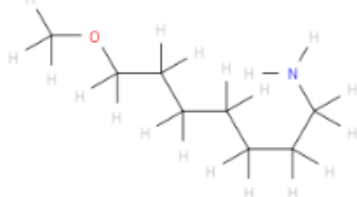 Ether6V2.xyz

Ether7V2	NCCCCCCCCOC	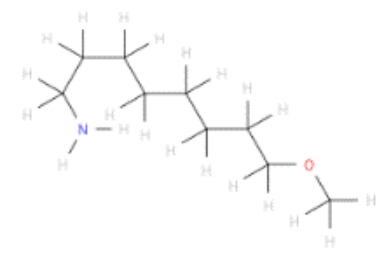 Ether7V2.xyz
Ether8V2	NCCCCCCCCCOC	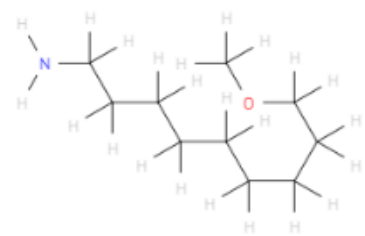 Ether8V2.xyz
F0	NCF	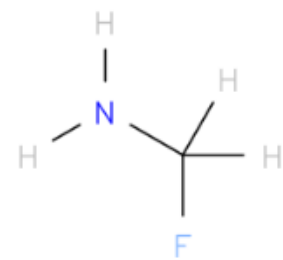 F0.xyz
F1	NCCF	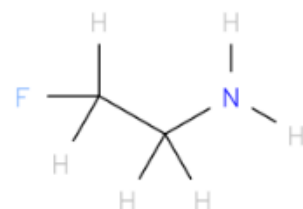 F1.xyz
F2	NCCCF	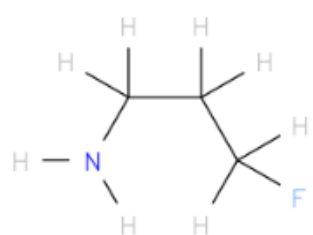 F2.xyz
F3	FCNCF	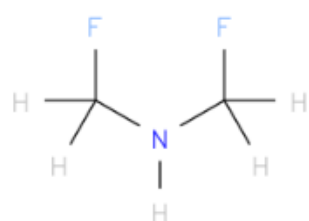 F3.xyz
F4	FCNCCF	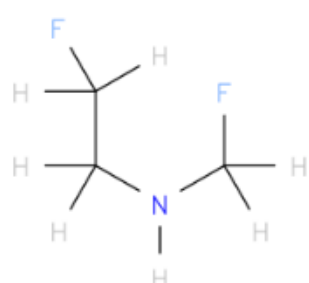 F4.xyz

F5	FCN(CF)CF	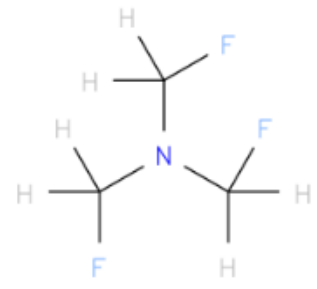 F5.xyz
F6	FCCN(CCF)CCF	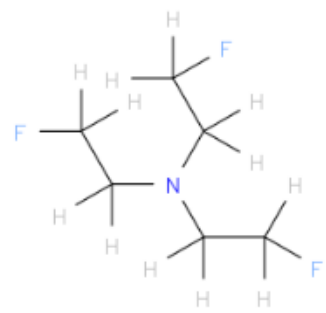 F6.xyz
Halid3V2	NCCCCF	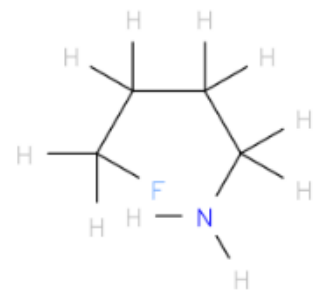 Halid3V2.xyz
Halid4V2	NCCCCCF	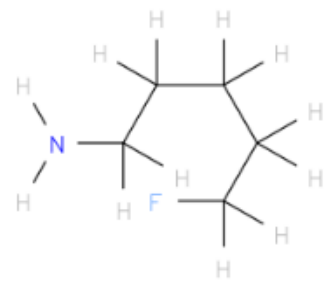 Halid4V2.xyz
Halid5V2	NCCCCCCF	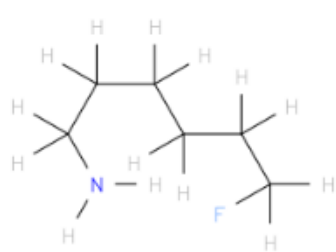 Halid5V2.xyz
Halid6V2	NCCCCCCCF	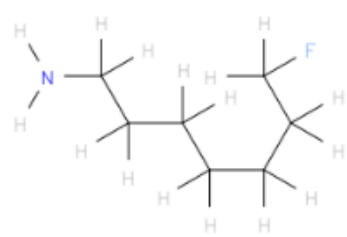 Halid6V2.xyz
Halid7V2	NCCCCCCCCF	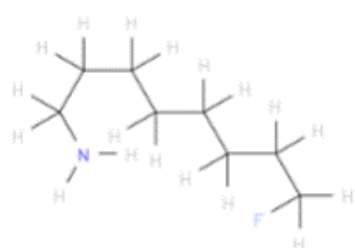 Halid7V2.xyz

Halid8V2	NCCCCCCCCCF	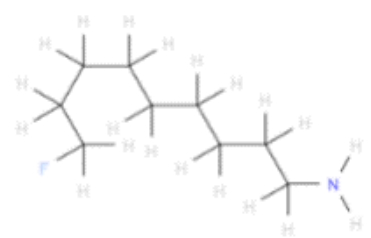<br>Halid8V2.xyz
I0	NCI	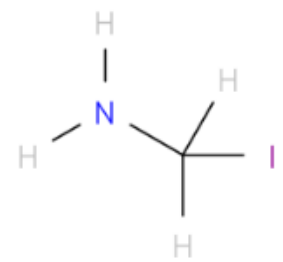<br>I0.xyz
I1	NCCI	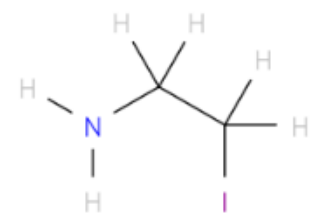<br>I1.xyz
I2	NCCCI	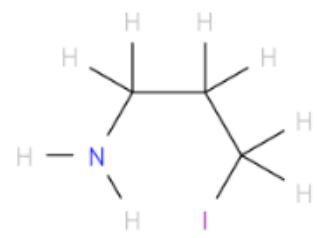<br>I2.xyz
I3	ICNCI	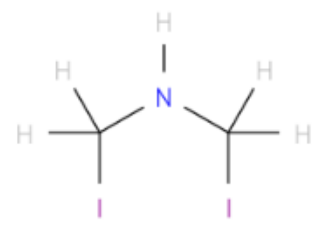<br>I3.xyz
I3V2	NCCCCI	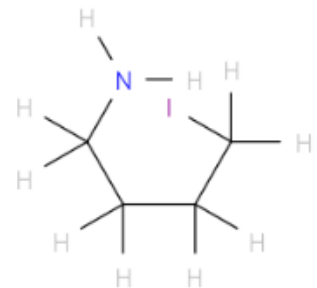<br>I3V2.xyz
I4	ICNCCI	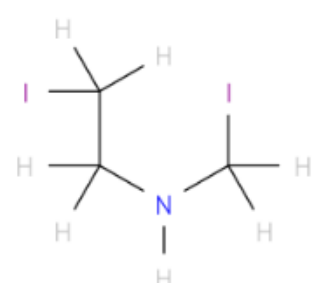<br>I4.xyz

I4V2	NCCCCCI	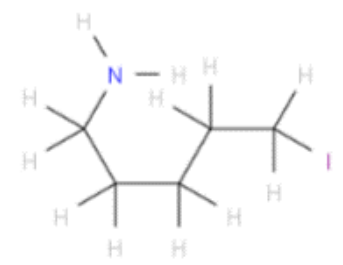<br>I4V2.xyz
I5	ICN(CI)CI	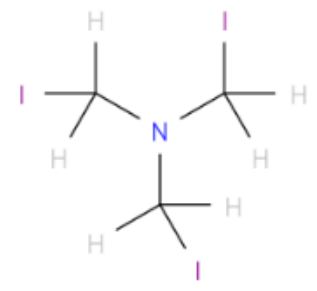<br>I5.xyz
I5V2	NCCCCCCI	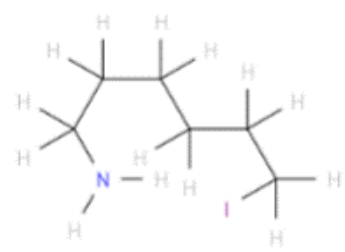<br>I5V2.xyz
I6	ICCN(CCI)CCI	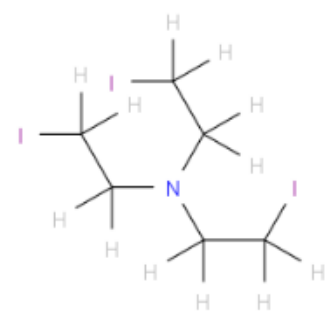<br>I6.xyz
I6V2	NCCCCCCCI	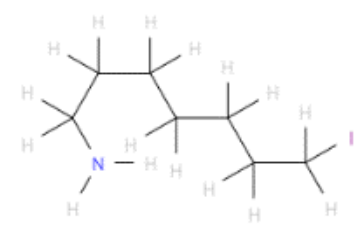<br>I6V2.xyz
I7V2	NCCCCCCCCI	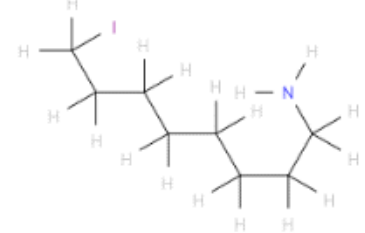<br>I7V2.xyz
I8V2	NCCCCCCCCCI	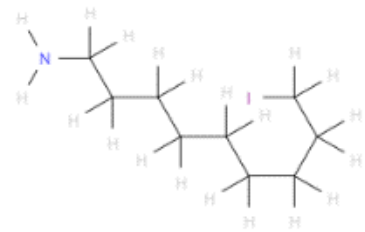<br>I8V2.xyz

ImidazoleBr1	[nH]1ccnc1Br	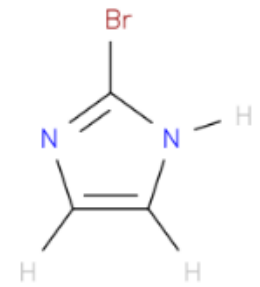 795
ImidazoleBr2	[nH]1c(cnc1)Br	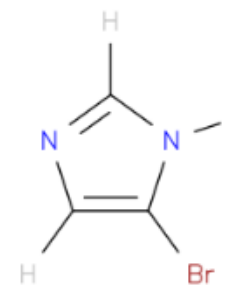 795
ImidazoleC#N1	[nH]1ccnc1C#N	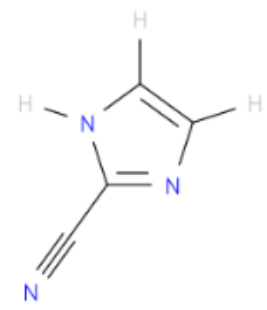 795
ImidazoleC#N2	[nH]1c(cnc1)C#N	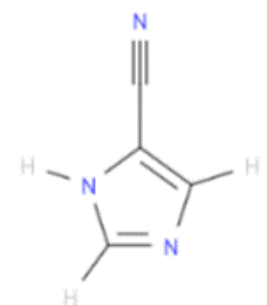 795
ImidazoleCl1	[nH]1ccnc1Cl	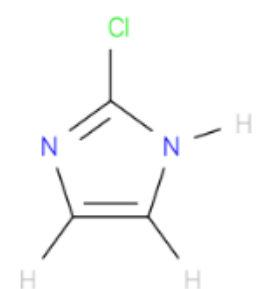 795
ImidazoleCl2	[nH]1c(cnc1)Cl	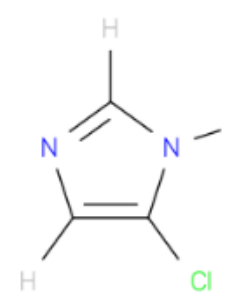 795
ImidazoleF1	[nH]1ccnc1F	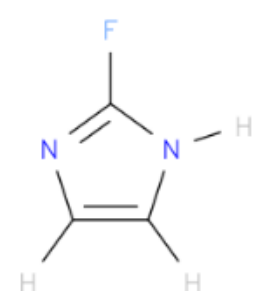 795

ImidazoleF2	[nH]1c(cnc1)F	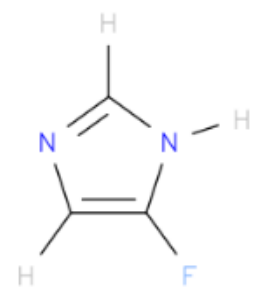 795
ImidazoleI1	[nH]1ccnc1I	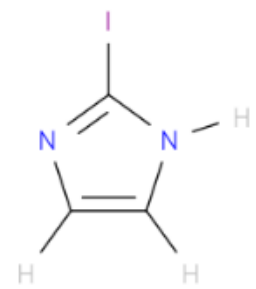 795
ImidazoleI2	[nH]1c(cnc1)I	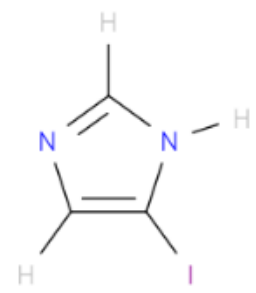 795
ImidazoleN1	[nH]1ccnc1N	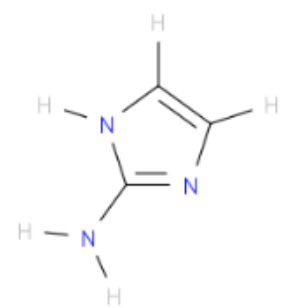 795
ImidazoleN2	[nH]1c(cnc1)N	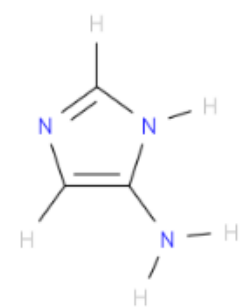 795
ImidazoleNO2_1	[nH]1ccnc1N(=O)=O	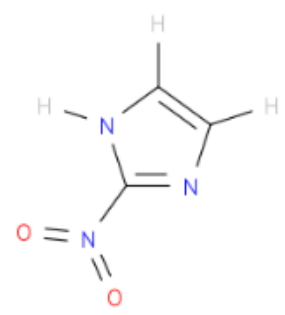 795
ImidazoleNO2_2	[nH]1c(cnc1)N(=O)=O	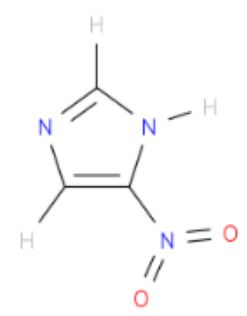 795

ImidazoleO1	[nH]1ccnc1O	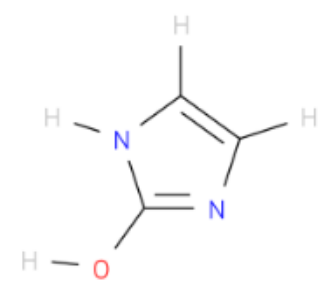 795
ImidazoleO2	[nH]1c(cnc1)O	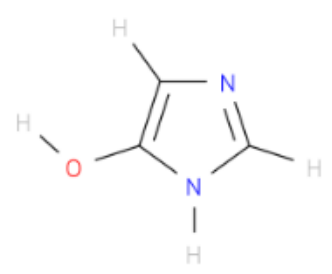 795
ImidazoleS1	[nH]1ccnc1S	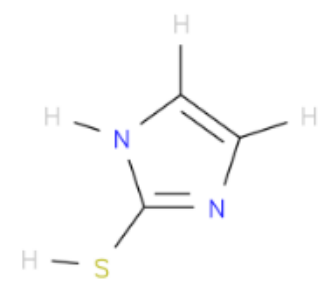 795
ImidazoleS2	[nH]1c(cnc1)S	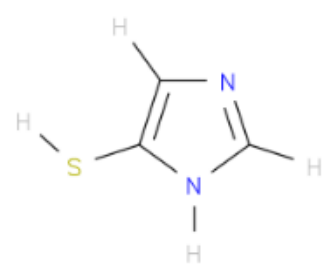 795
Ketone3V2	NCCCCC=O	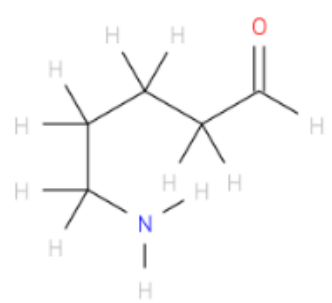 Ketone3V2.xyz
Ketone4V2	NCCCCCC=O	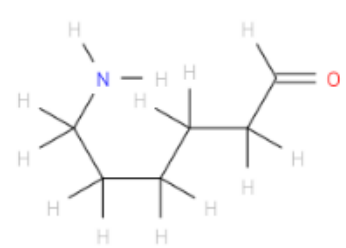 Ketone4V2.xyz
Ketone5V2	NCCCCCCC=O	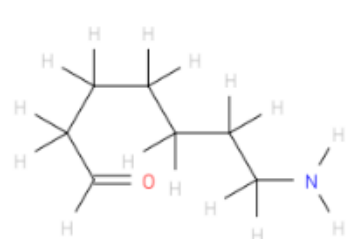 Ketone5V2.xyz

Ketone6V2	NCCCCCCCC=O	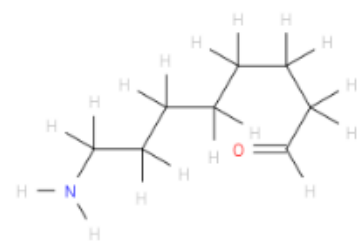 Ketone6V2.xyz
Ketone7V2	NCCCCCCCCC=O	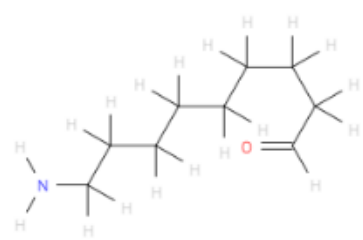 Ketone7V2.xyz
Ketone8V2	NCCCCCCCCCC=O	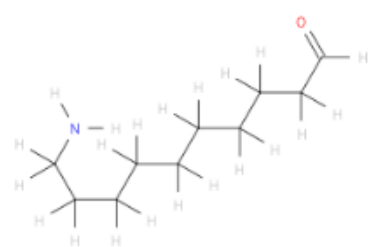 Ketone8V2.xyz
Nitro0	NCCN(=O)=O	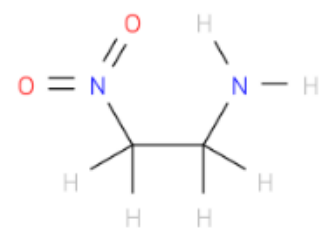 Nitro0.xyz
Nitro1	NCCCN(=O)=O	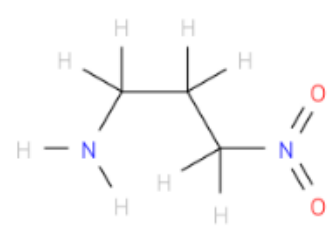 Nitro1.xyz
Nitro3	NCCCCCN(=O)=O	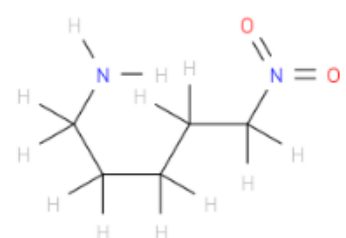 Nitro3.xyz
Nitro30	C(N(=O)=O)CNCCN(=O)=O	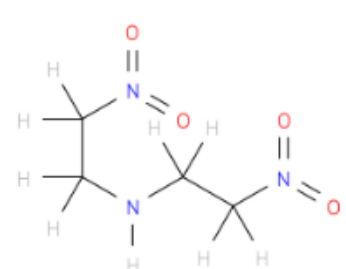 Nitro30.xyz

Nitro430	C(N(=O)=O)CN(CCN(=O)=O)CCN(=O)=O	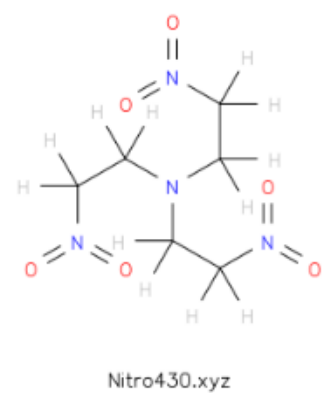
Nitro4V2	NCCCCCCN(=O)=O	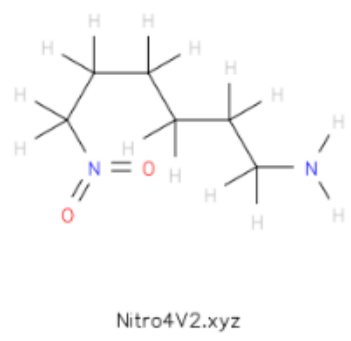
Nitro50	C(N(=O)=O)CNCCCN(=O)=O	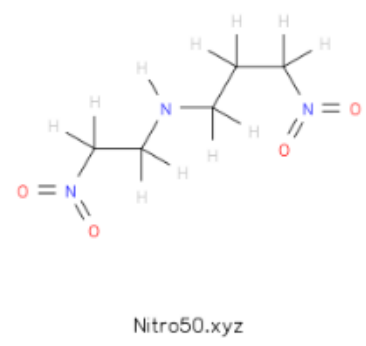
Nitro5V2	NCCCCCCCN(=O)=O	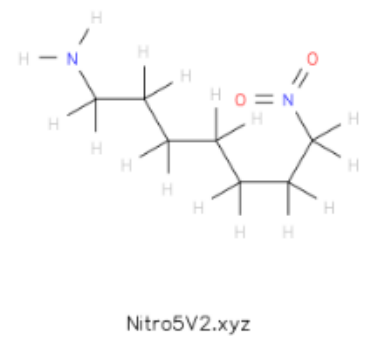
Nitro671	C(N(=O)=O)CCN(CCCN(=O)=O)CCCN(=O)=O	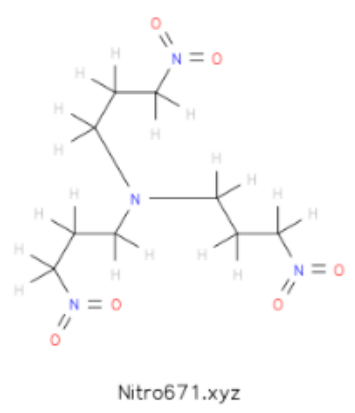
Nitro6V2	NCCCCCCCCN(=O)=O	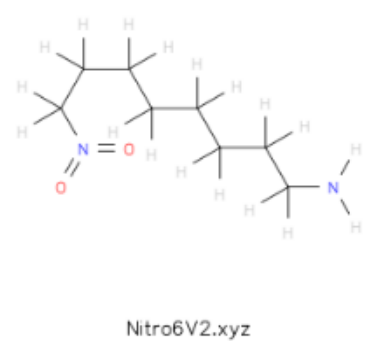
Nitro7V2	NCCCCCCCCCN(=O)=O	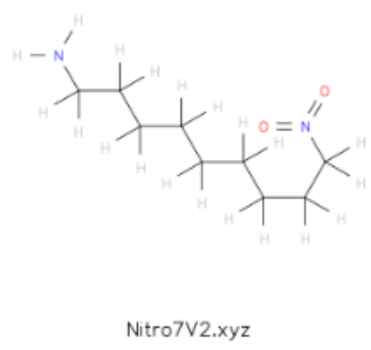

Nitro8V2	NCCCCCCCCCCN(=O)=O	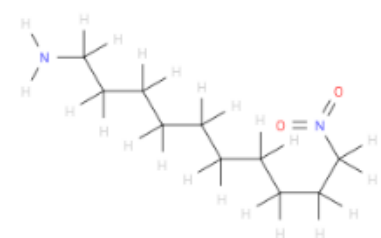 Nitro8V2.xyz
PiperidineBr1	N1[C@@H](CCCC1)Br	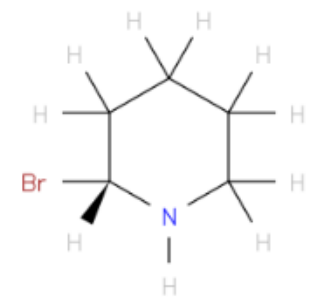 8082
PiperidineBr2	N1C[C@H](CCC1)Br	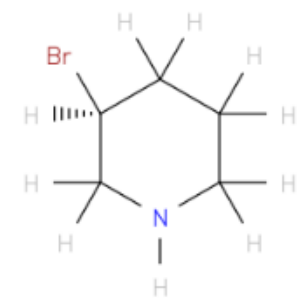 8082
PiperidineBr3	N1CCC(CC1)Br	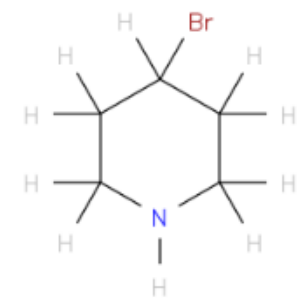 8082
PiperidineC#N1	N1[C@@H](CCCC1)C#N	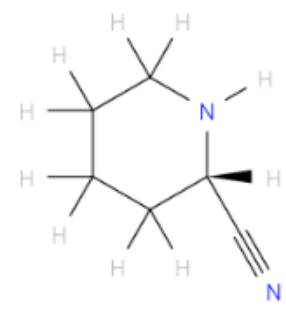 8082
PiperidineC#N2	N1C[C@H](CCC1)[C][N]	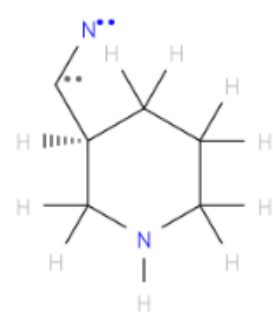 8082
PiperidineC#N3	N1CCC(CC1)C#N	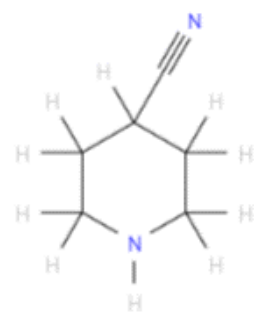 8082

PiperidineCl1	N1[C@@H](CCCC1)Cl	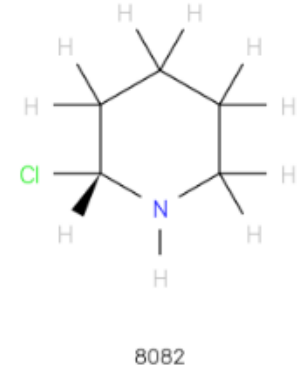
PiperidineCl2	N1C[C@H](CCC1)Cl	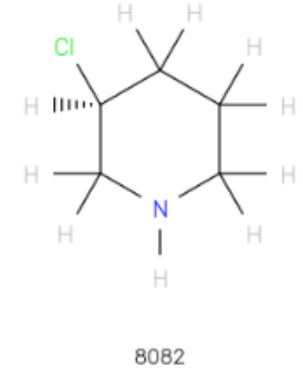
PiperidineCl3	N1CCC(CC1)Cl	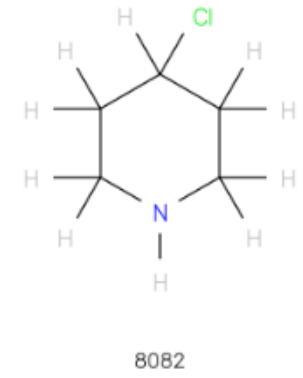
PiperidineF1	N1[C@@H](CCCC1)F	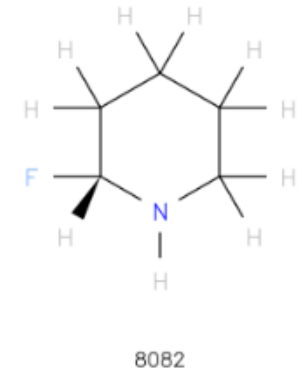
PiperidineF2	N1C[C@H](CCC1)F	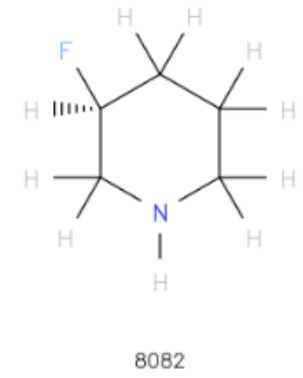
PiperidineF3	N1CCC(CC1)F	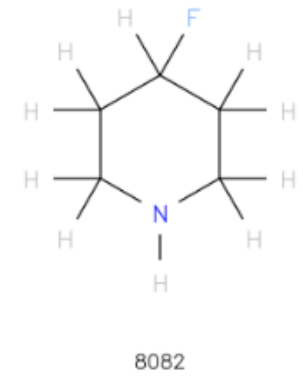
PiperidineI1	N1[C@@H](CCCC1)I	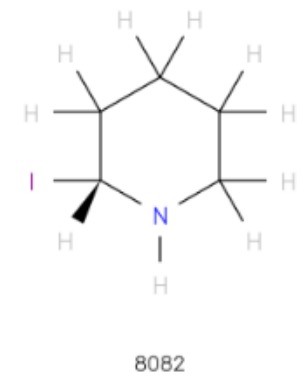

PiperidineI2	N1C[C@H](CCC1)I	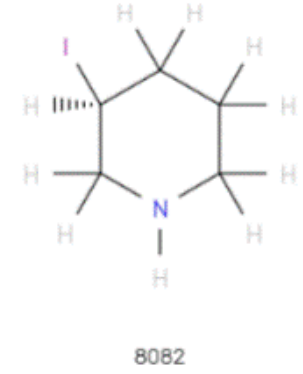
PiperidineI3	N1CCC(CC1)I	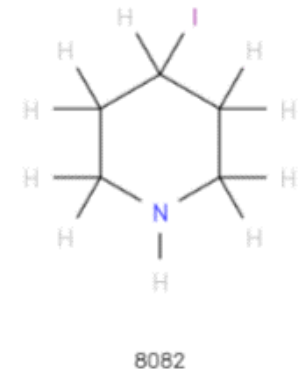
PiperidineN1	N1[C@@H](CCCC1)N	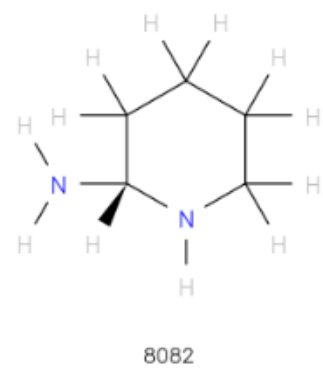
PiperidineN2	N1C[C@H](CCC1)N	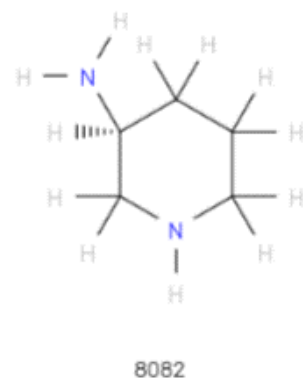
PiperidineN3	N1CCC(CC1)N	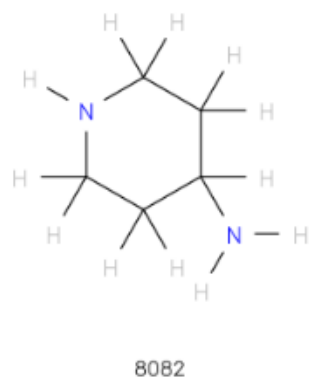
PiperidineNO2_1	N1[C@@H](CCCC1)N(=O)=O	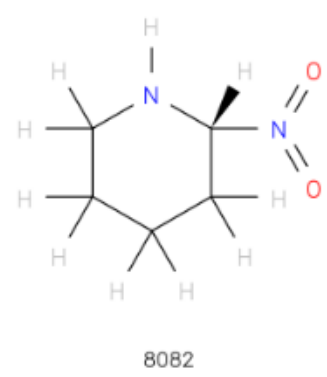
PiperidineNO2_2	N1C[C@H](CCC1)N(=O)=O	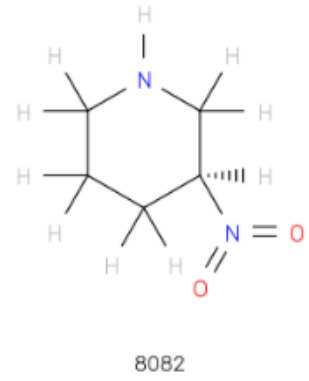

PiperidineNO2_3	N1CCC(CC1)N(=O)=O	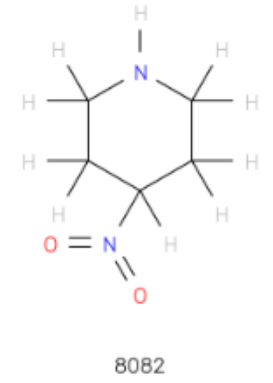
PiperidineO1	N1[C@@H](CCCC1)O	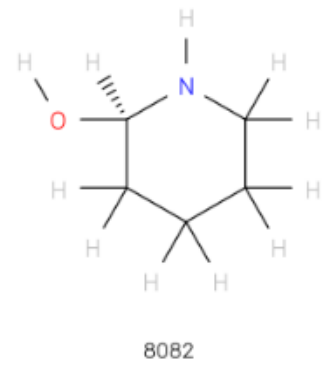
PiperidineO2	N1C[C@H](CCC1)O	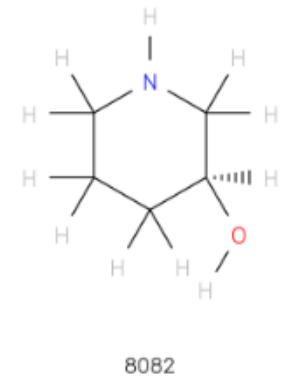
PiperidineO3	N1CCC(CC1)O	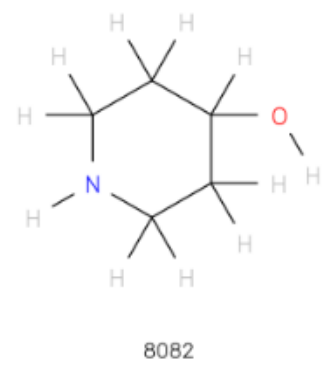
PiperidineS1	N1[C@@H](CCCC1)S	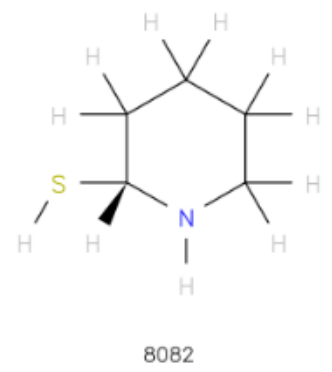
PiperidineS2	N1C[C@H](CCC1)S	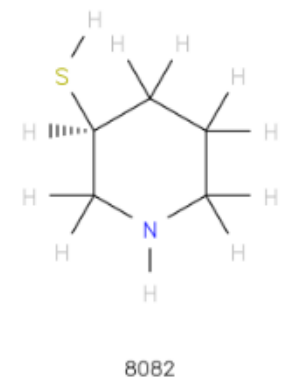
PiperidineS3	N1CCC(CC1)S	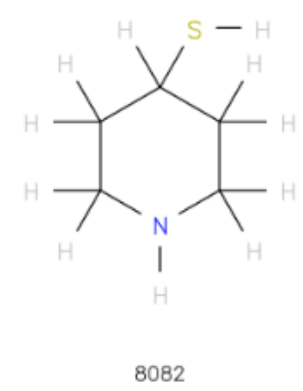

PyrazineBr1	n1c(cncc1)Br	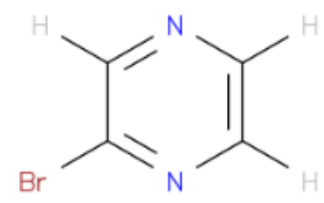 9261
PyrazineBr2	n1ccnc(c1)Br	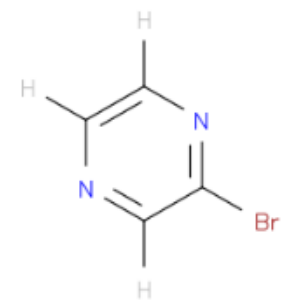 9261
PyrazineC#N1	n1c(cncc1)C#N	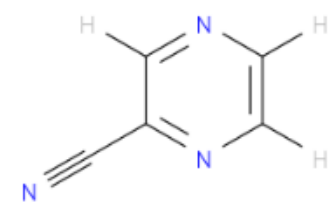 9261
PyrazineC#N2	n1ccnc(c1)C#N	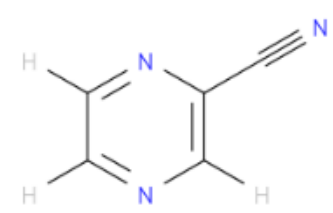 9261
PyrazineCl1	n1c(cncc1)Cl	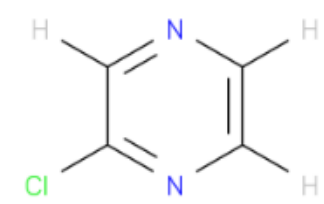 9261
PyrazineCl2	n1ccnc(c1)Cl	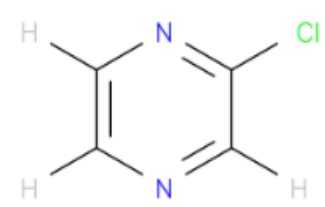 9261
PyrazineF1	n1c(cncc1)F	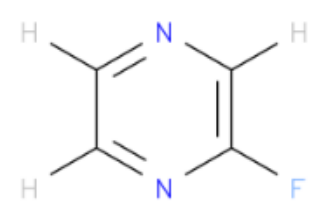 9261

PyrazineF2	n1ccnc(c1)F	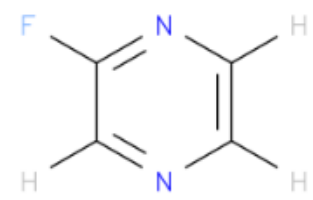 9261
PyrazineI1	n1c(cncc1)I	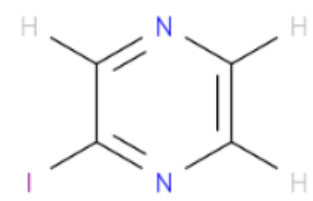 9261
PyrazineI2	n1ccnc(c1)I	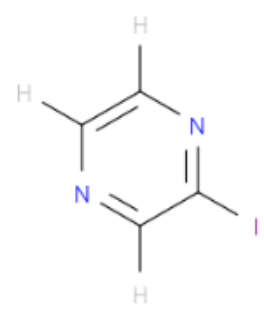 9261
PyrazineN1	n1c(cncc1)N	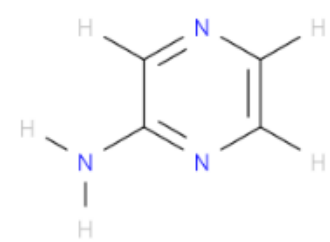 9261
PyrazineN2	n1ccnc(c1)N	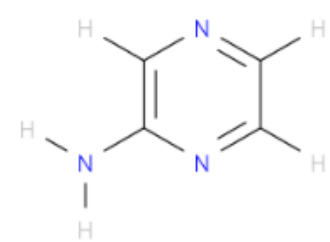 9261
PyrazineNO2_1	n1c(cncc1)N(=O)=O	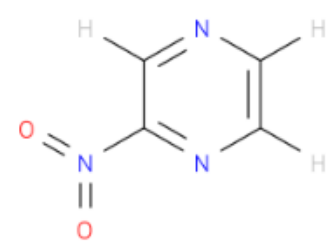 9261
PyrazineNO2_2	n1ccnc(c1)N(=O)=O	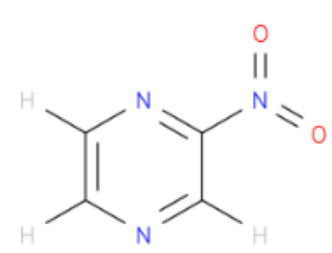 9261

PyrazineO1	N1=C([CH]N=C[CH]1)O	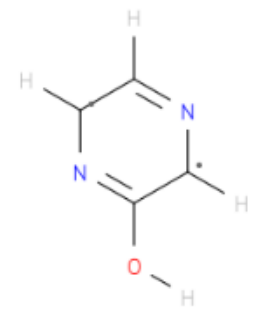 9261
PyrazineO2	N1=C[CH]N=C([CH]1)O	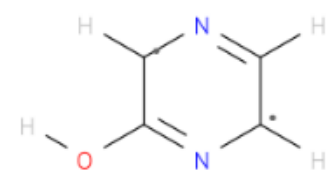 9261
PyrazineS1	n1c(cncc1)S	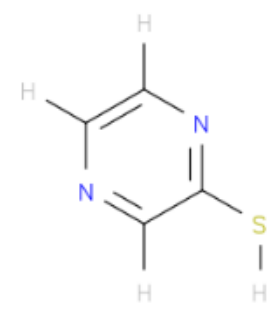 9261
PyrazineS2	n1ccnc(c1)S	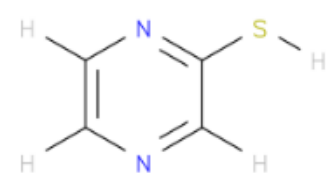 9261
PyrazoleBr1	[nH]1nc(cc1)Br	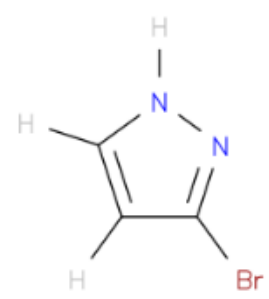 1048
PyrazoleBr2	[nH]1ncc(c1)Br	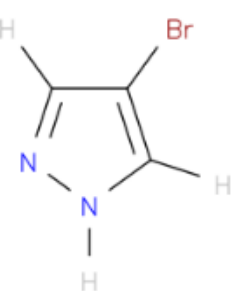 1048
PyrazoleC#N1	[nH]1nc(cc1)C#N	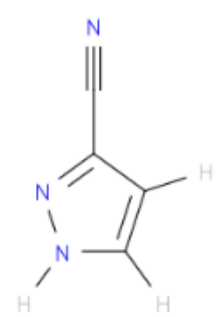 1048

PyrazoleC#N2	[nH]1ncc(c1)C#N	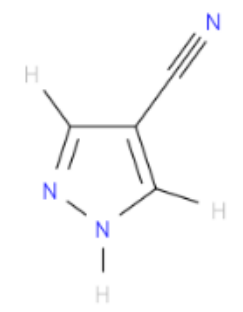 1048
PyrazoleCl1	[nH]1nc(cc1)Cl	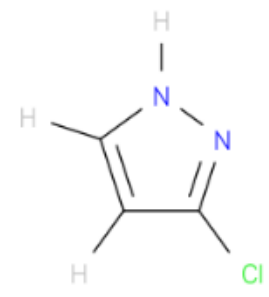 1048
PyrazoleCl2	[nH]1ncc(c1)Cl	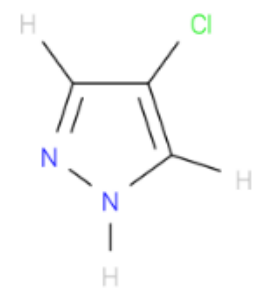 1048
PyrazoleF1	[nH]1nc(cc1)F	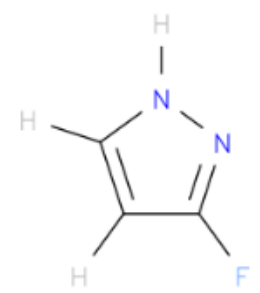 1048
PyrazoleF2	[nH]1ncc(c1)F	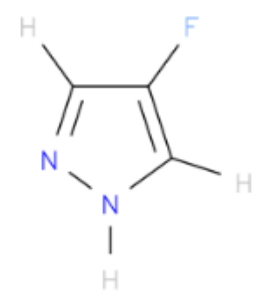 1048
PyrazoleI1	[nH]1nc(cc1)I	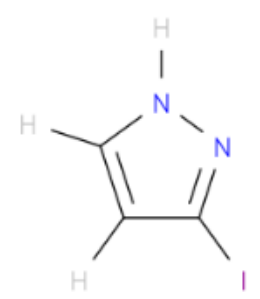 1048
PyrazoleI2	[nH]1ncc(c1)I	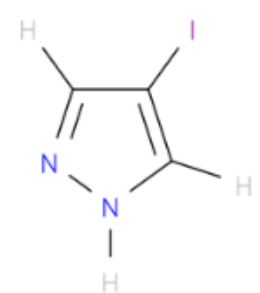 1048

PyrazoleN1	[nH]1nc(cc1)N	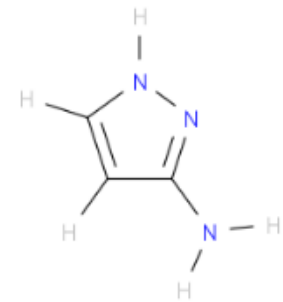 1048
PyrazoleN2	[nH]1ncc(c1)N	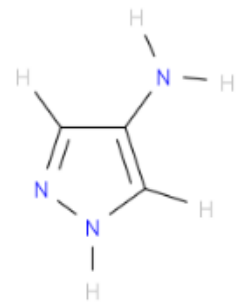 1048
PyrazoleNO2_1	[nH]1nc(cc1)N(=O)=O	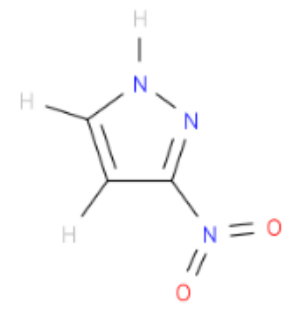 1048
PyrazoleNO2_2	[nH]1ncc(c1)N(=O)=O	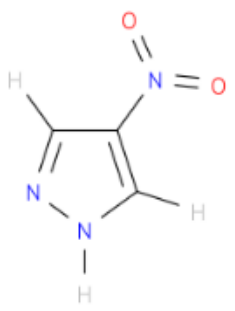 1048
PyrazoleO1	[nH]1nc(cc1)O	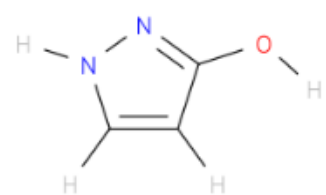 1048
PyrazoleO2	[nH]1ncc(c1)O	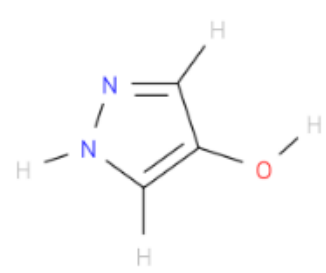 1048
PyrazoleS1	[nH]1nc(cc1)S	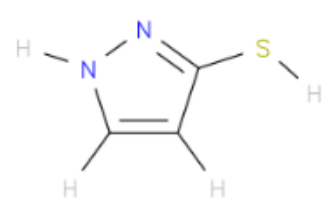 1048

PyrazoleS2	[nH]1ncc(c1)S	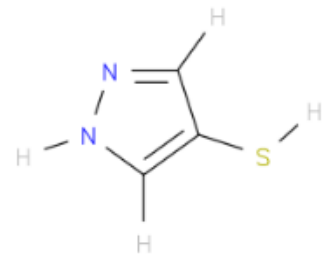 1048
PyridineBr1	n1c(cccc1)Br	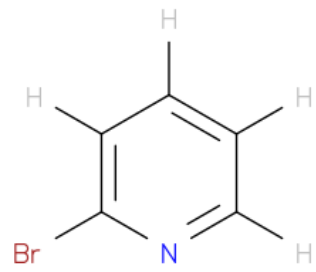 1049
PyridineBr2	n1cc(ccc1)Br	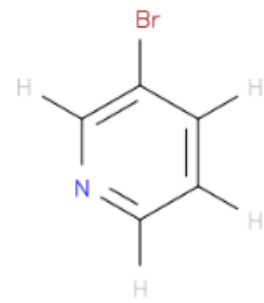 1049
PyridineBr3	n1ccc(cc1)Br	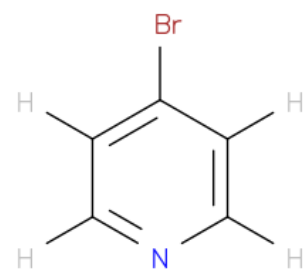 1049
PyridineC#N1	n1c(cccc1)C#N	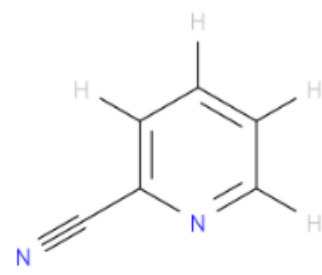 1049
PyridineC#N2	n1cc(ccc1)C#N	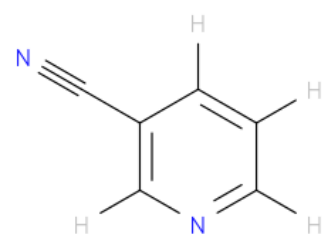 1049
PyridineC#N3	n1ccc(cc1)C#N	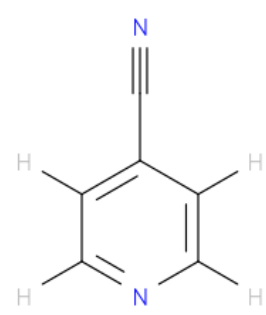 1049

PyridineCl1	n1c(cccc1)Cl	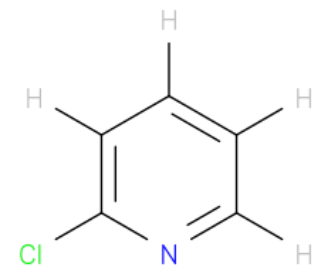 1049
PyridineCl2	n1cc(ccc1)Cl	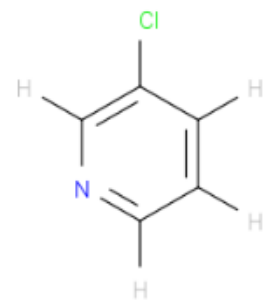 1049
PyridineCl3	n1ccc(cc1)Cl	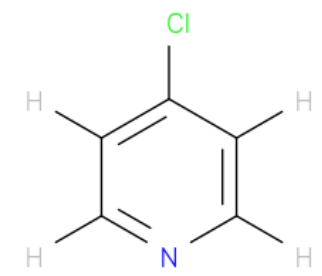 1049
PyridineF1	n1c(cccc1)F	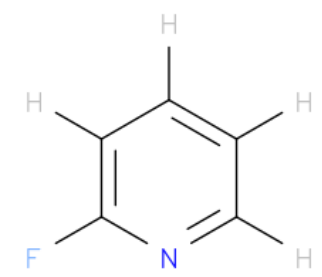 1049
PyridineF2	n1cc(ccc1)F	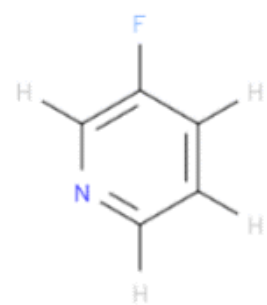 1049
PyridineF3	n1ccc(cc1)F	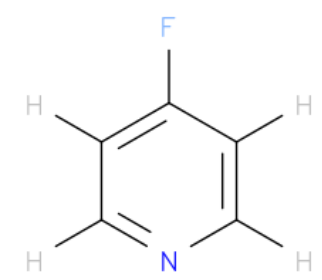 1049
PyridineI1	n1c(cccc1)I	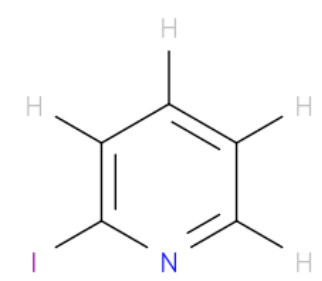 1049

PyridineI2	n1cc(ccc1)I	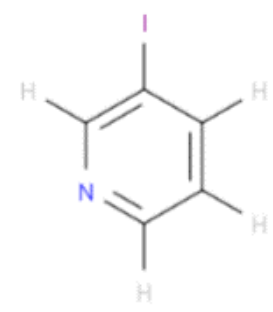 1049
PyridineI3	n1ccc(cc1)I	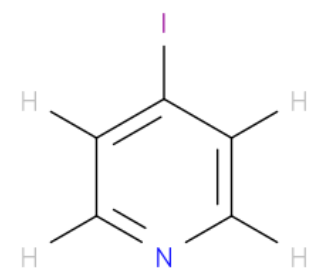 1049
PyridineN1	n1c(cccc1)N	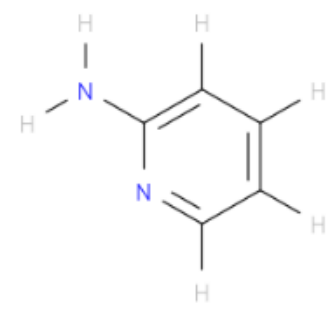 1049
PyridineN2	n1cc(ccc1)N	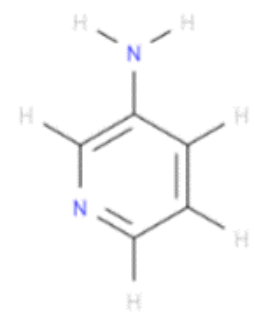 1049
PyridineN3	n1ccc(cc1)N	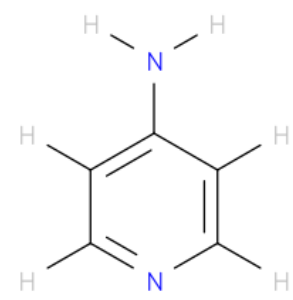 1049
PyridineNO2_1	n1c(cccc1)N(=O)=O	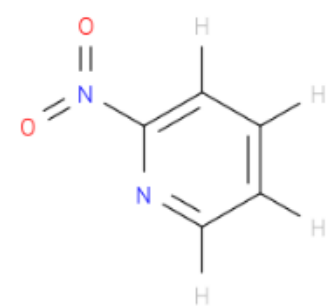 1049
PyridineNO2_2	n1cc(ccc1)N(=O)=O	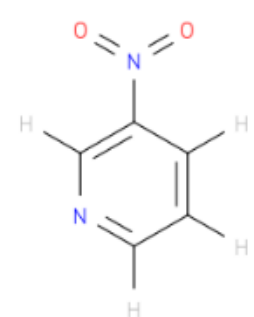 1049

PyridineNO2_3	n1ccc(cc1)N(=O)=O	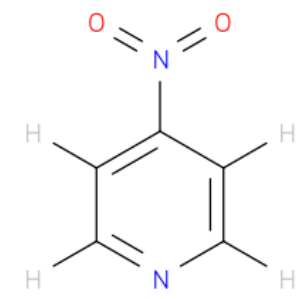 1049
PyridineO1	n1c(cccc1)O	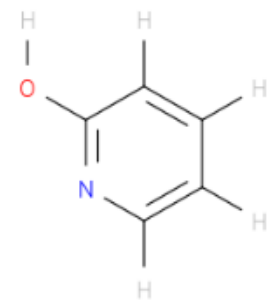 1049
PyridineO2	n1cc(ccc1)O	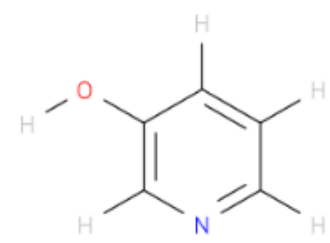 1049
PyridineO3	n1ccc(cc1)O	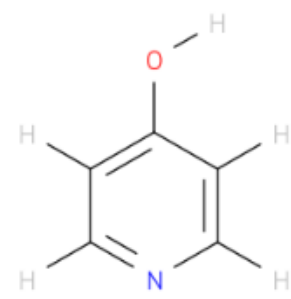 1049
PyridineS1	n1c(cccc1)S	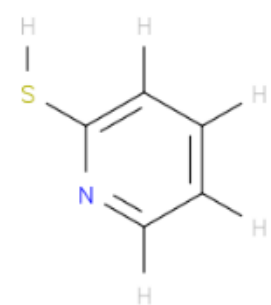 1049
PyridineS2	n1cc(ccc1)S	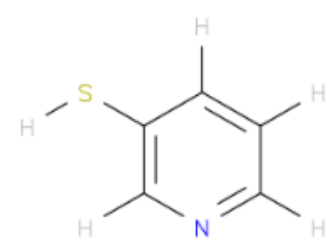 1049
PyridineS3	n1ccc(cc1)S	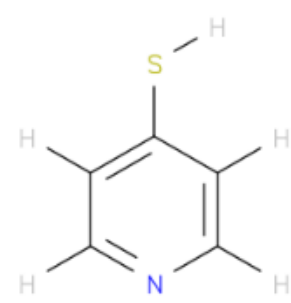 1049

PyrimidineBr1	n1cccnc1Br	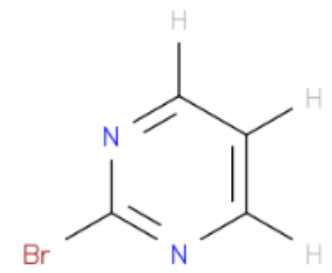 9260
PyrimidineBr2	n1c(ccnc1)Br	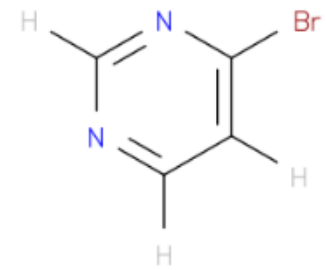 9260
PyrimidineBr3	n1cc(cnc1)Br	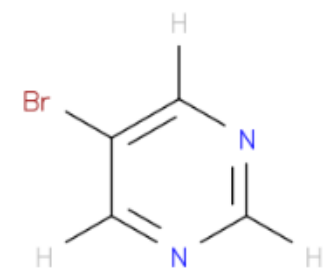 9260
PyrimidineC#N 1	n1cccnc1C#N	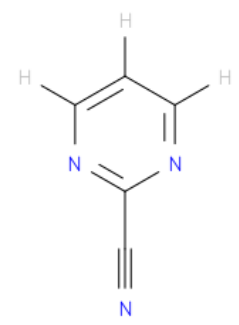 9260
PyrimidineC#N 2	n1c(ccnc1)C#N	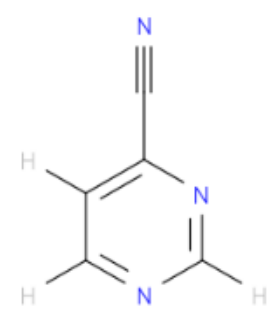 9260
PyrimidineC#N 3	n1cc(cnc1)C#N	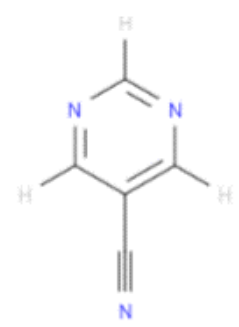 9260
PyrimidineCl1	n1cccnc1Cl	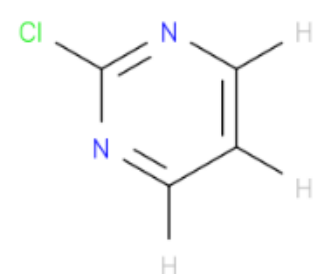 9260

PyrimidineCl2	n1c(ccnc1)Cl	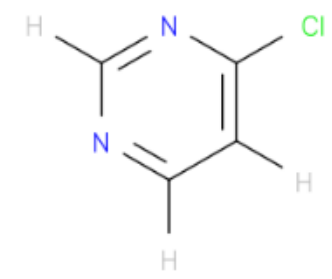 9260
PyrimidineCl3	n1cc(cnc1)Cl	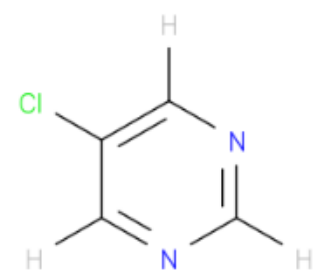 9260
PyrimidineF1	n1cccnc1F	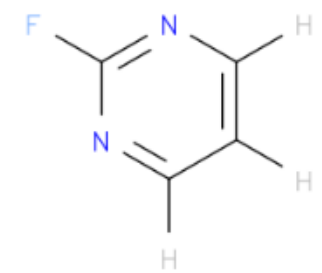 9260
PyrimidineF2	n1c(ccnc1)F	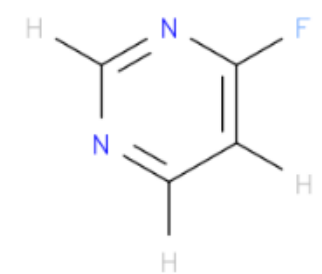 9260
PyrimidineF3	N1=C[N]C=C([CH]1)F	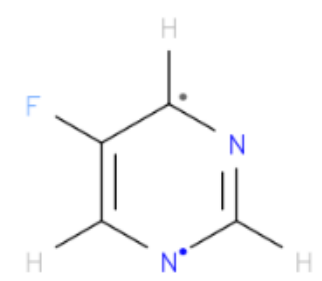 9260
PyrimidineI1	n1cccnc1I	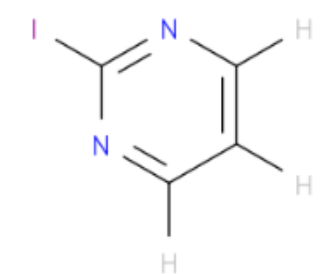 9260
PyrimidineI2	n1c(ccnc1)I	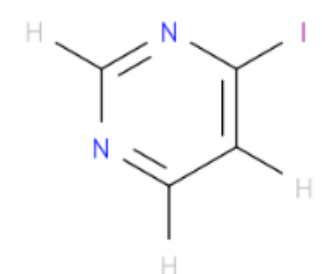 9260

PyrimidineI3	n1cc(cnc1)I	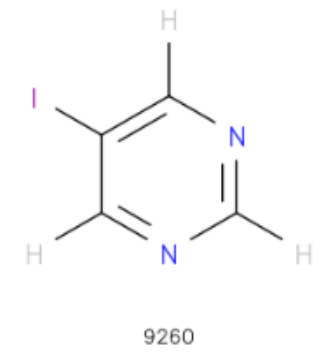
PyrimidineN1	n1cccnc1N	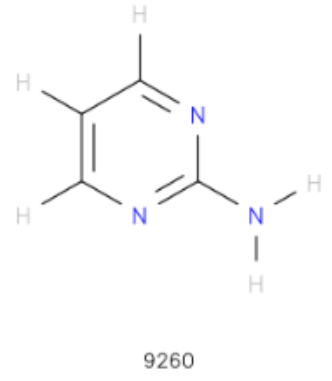
PyrimidineN2	n1c(ccnc1)N	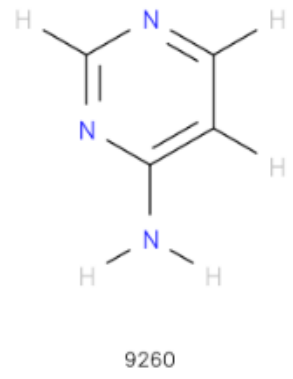
PyrimidineN3	n1cc(cnc1)N	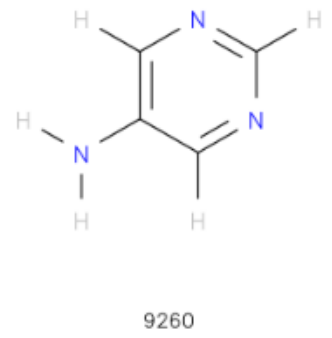
PyrimidineNO2_1	n1cccnc1N(=O)=O	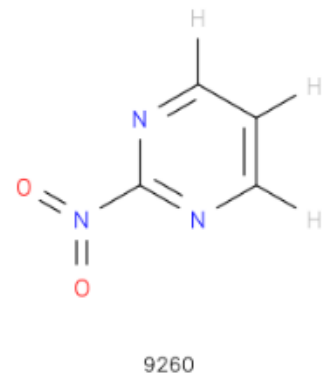
PyrimidineNO2_2	n1c(ccnc1)N(=O)=O	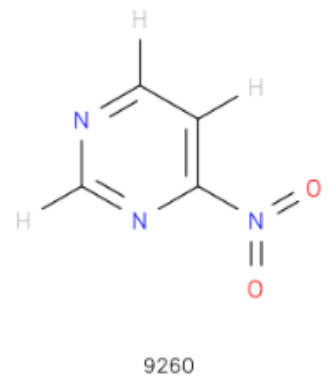
PyrimidineNO2_3	n1cc(cnc1)N(=O)=O	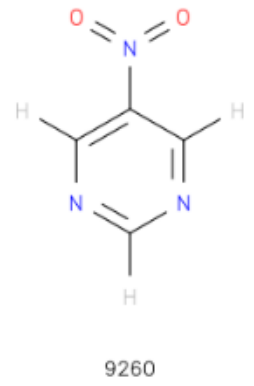

PyrimidineO1	N1=[C](=NC=C[CH]1)O	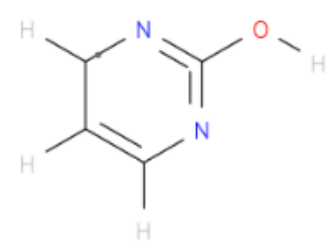<br>9260
PyrimidineO2	n1c(ccnc1)O	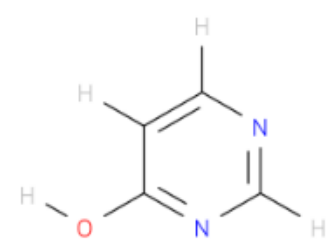<br>9260
PyrimidineO3	n1cc(cnc1)O	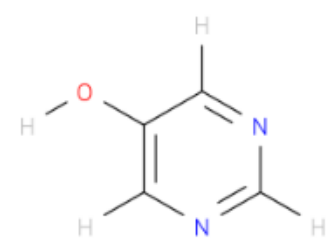<br>9260
PyrimidineS1	n1cccnc1S	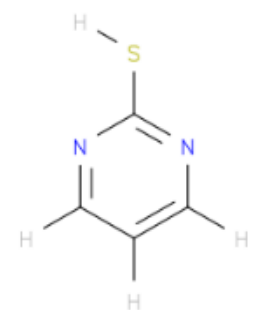<br>9260
PyrimidineS2	n1c(ccnc1)S	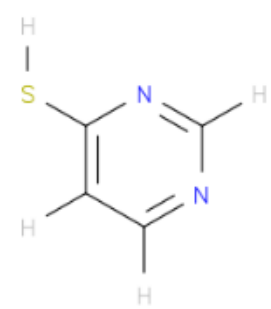<br>9260
PyrimidineS3	n1cc(cnc1)S	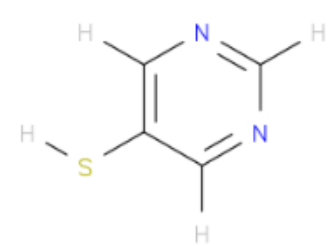<br>9260
PyrroleBr1	[nH]1cccc1Br	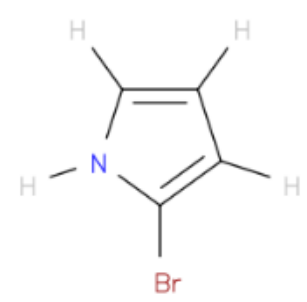<br>8027

PyrroleBr2	[nH]1ccc(c1)Br	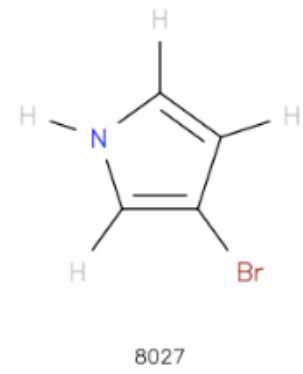
PyrroleC#N1	[nH]1cccc1C#N	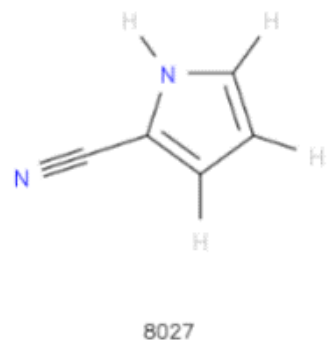
PyrroleC#N2	[nH]1ccc(c1)C#N	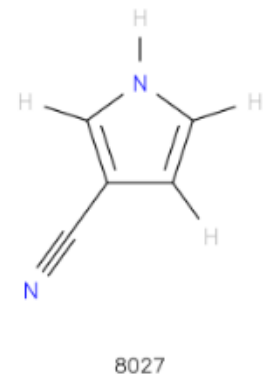
PyrroleCl1	[nH]1cccc1Cl	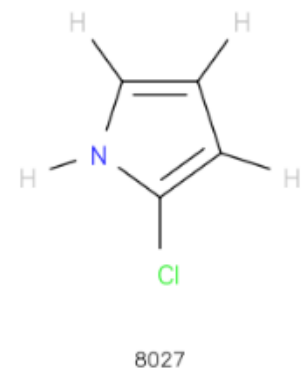
PyrroleCl2	[nH]1ccc(c1)Cl	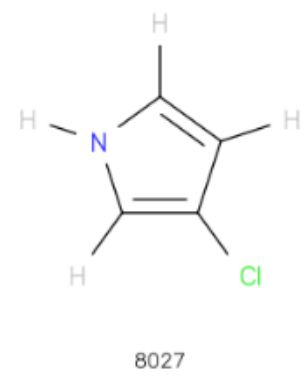
PyrroleF1	[nH]1cccc1F	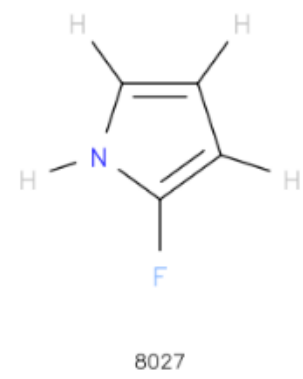
PyrroleF2	[nH]1ccc(c1)F	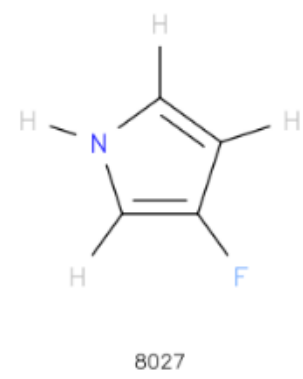

PyrroleI1	[nH]1cccc1I	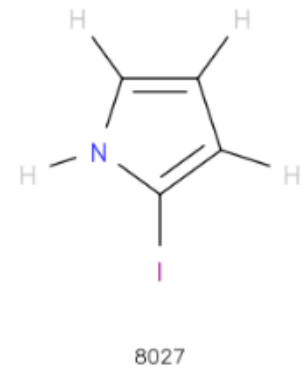
PyrroleI2	[nH]1ccc(c1)I	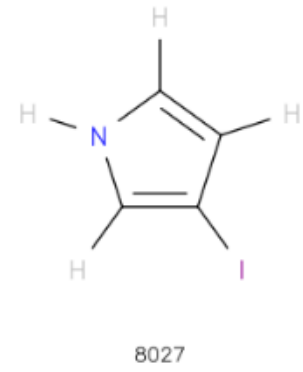
PyrroleN1	[nH]1cccc1N	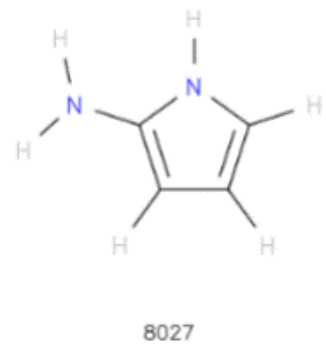
PyrroleN2	[nH]1ccc(c1)N	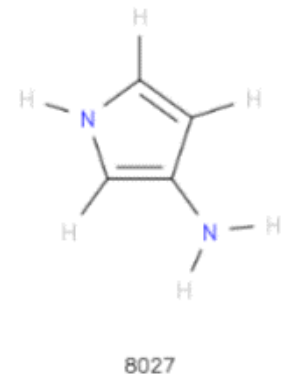
PyrroleNO2_1	[nH]1cccc1N(=O)=O	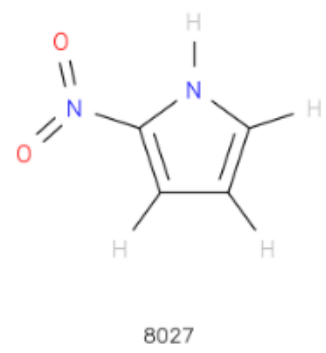
PyrroleNO2_2	[nH]1ccc(c1)N(=O)=O	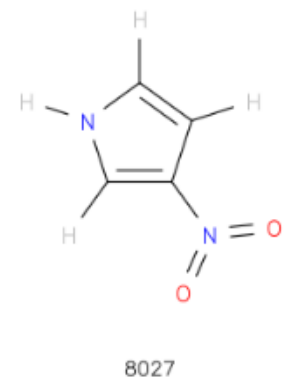
PyrroleO1	[nH]1cccc1O	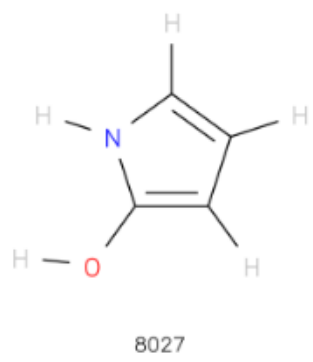

PyrroleO2	[nH]1ccc(c1)O	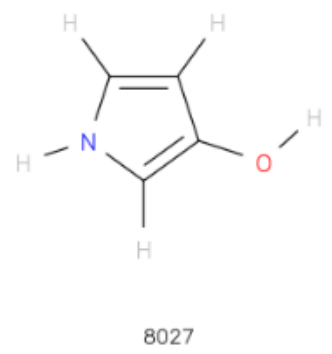
PyrroleS1	[nH]1cccc1S	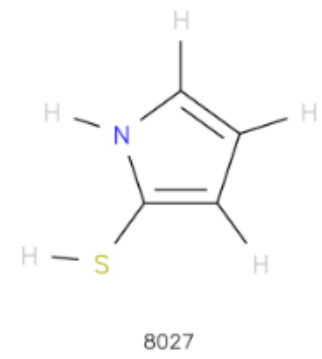
PyrroleS2	[nH]1ccc(c1)S	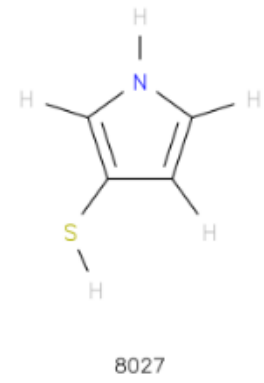
ThiazoleBr1	s1ccnc1Br	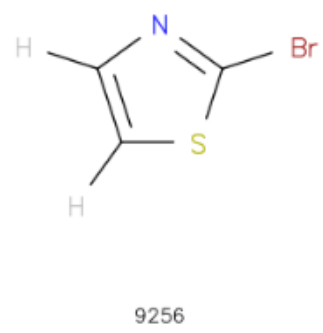
ThiazoleBr2	s1c(cnc1)Br	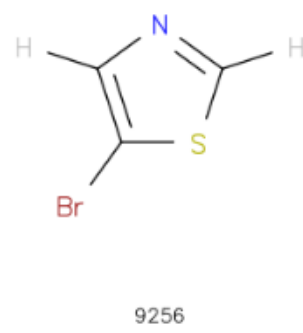
ThiazoleBr3	s1cc(nc1)Br	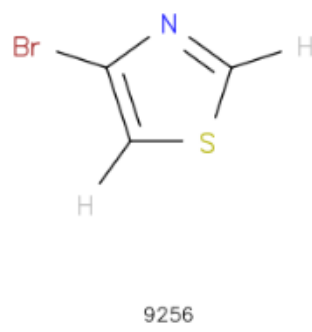
ThiazoleC#N1	s1ccnc1C#N	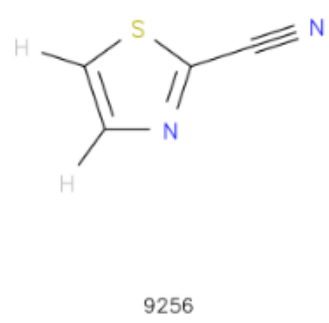

ThiazoleC#N2	s1c(cnc1)C#N	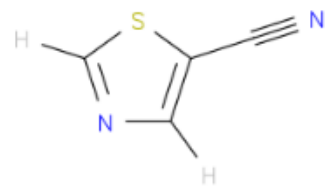 9256
ThiazoleC#N3	s1cc(nc1)C#N	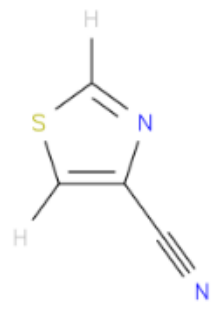 9256
ThiazoleCl1	s1ccnc1Cl	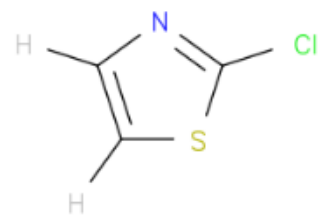 9256
ThiazoleCl2	s1c(cnc1)Cl	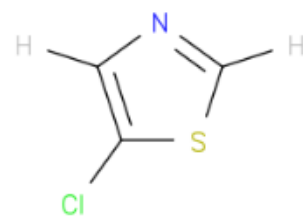 9256
ThiazoleCl3	s1cc(nc1)Cl	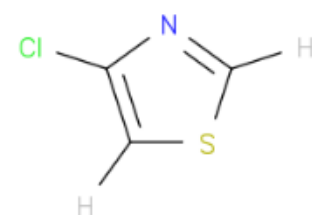 9256
ThiazoleF1	s1ccnc1F	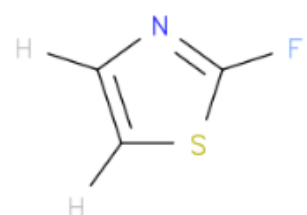 9256
ThiazoleF2	s1c(cnc1)F	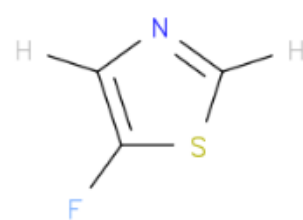 9256

ThiazoleF3	s1cc(nc1)F	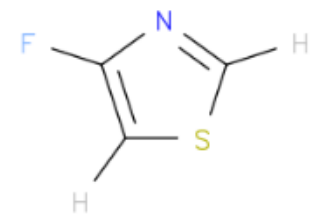 9256
ThiazoleI1	s1ccnc1I	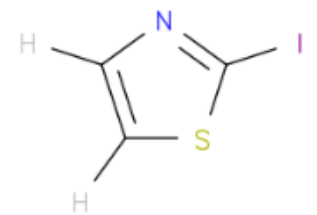 9256
ThiazoleI2	s1c(cnc1)I	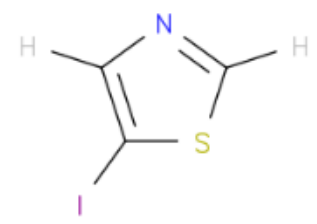 9256
ThiazoleI3	s1cc(nc1)I	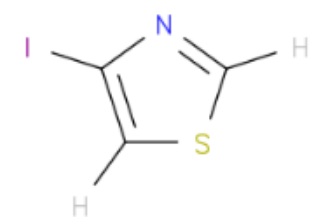 9256
ThiazoleN1	s1ccnc1N	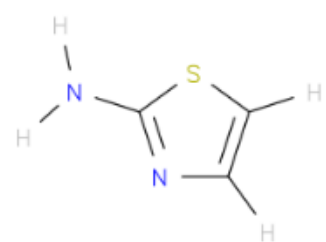 9256
ThiazoleN2	s1c(cnc1)N	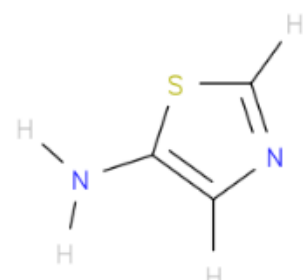 9256
ThiazoleN3	s1cc(nc1)N	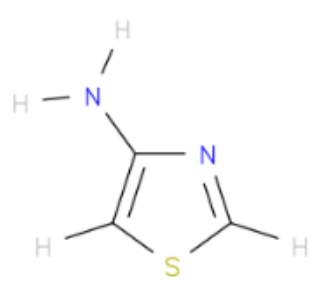 9256

ThiazoleNO2_1	s1ccnc1N(=O)=O	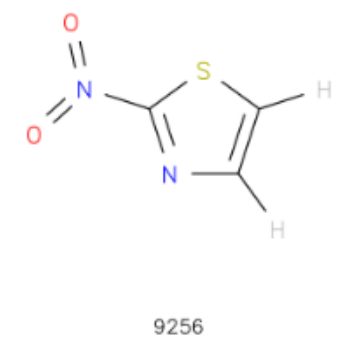
ThiazoleNO2_2	s1c(cnc1)N(=O)=O	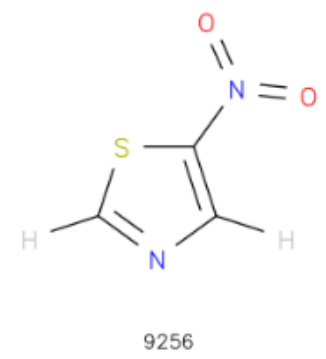
ThiazoleNO2_3	s1cc(nc1)N(=O)=O	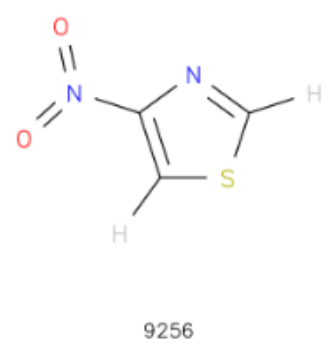
ThiazoleO1	s1ccnc1O	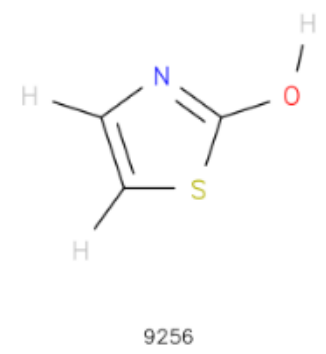
ThiazoleO2	s1c(cnc1)O	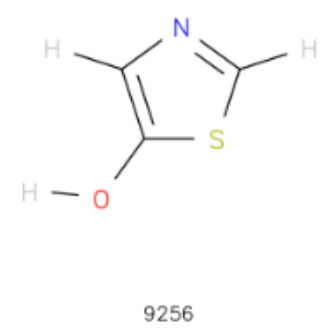
ThiazoleO3	S1[CH]C(=N[CH]1)O	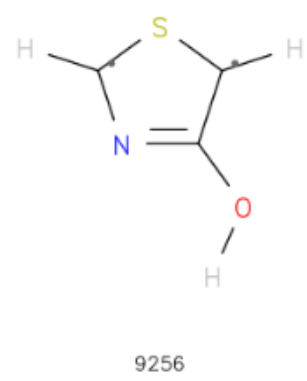
ThiazoleS1	s1ccnc1S	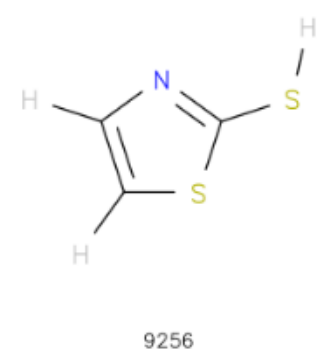

ThiazoleS2	s1c(cnc1)S	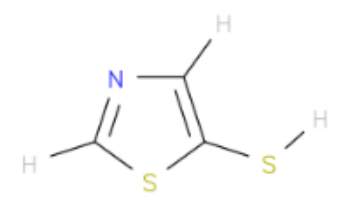 9256
ThiazoleS3	s1cc(nc1)S	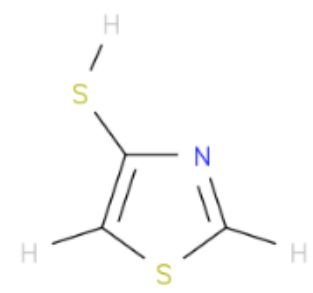 9256
Tio0	NCCS	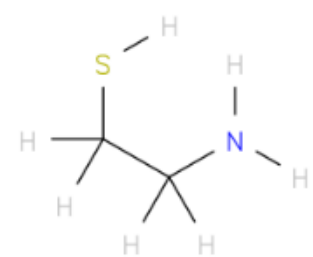 Tio0.xyz
Tio1	NCCCS	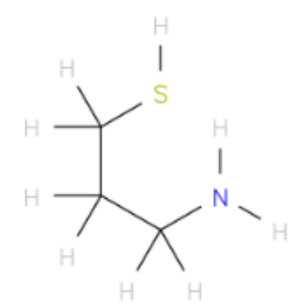 Tio1.xyz
Tio2	NCCCCS	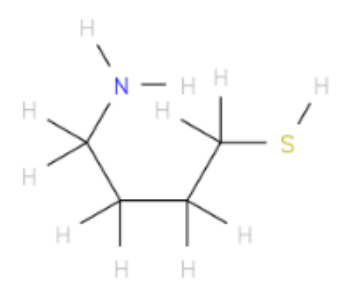 Tio2.xyz
Tio30	C(S)CNCCS	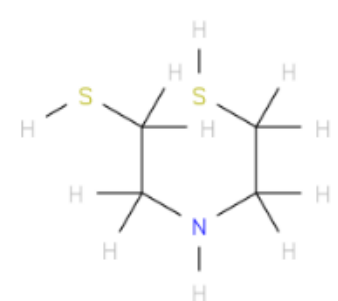 Tio30.xyz
Tio3V2	NCCCCCS	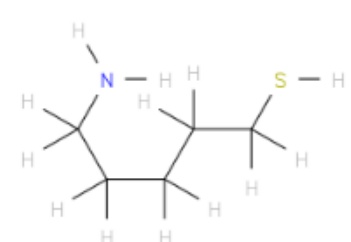 Tio3V2.xyz

Tio430	C(S)CN(CCS)CCS	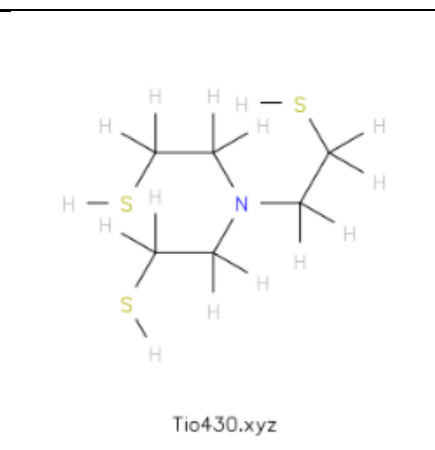
Tio4V2	NCCCCCCS	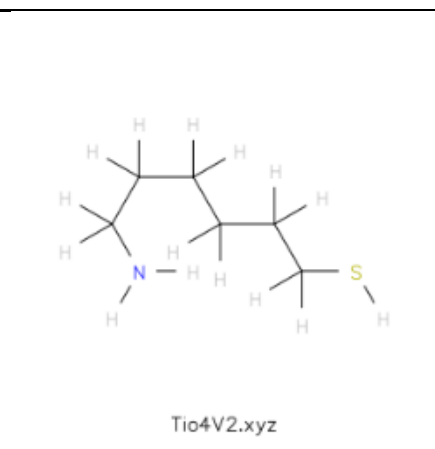
Tio50	C(S)CNCCCS	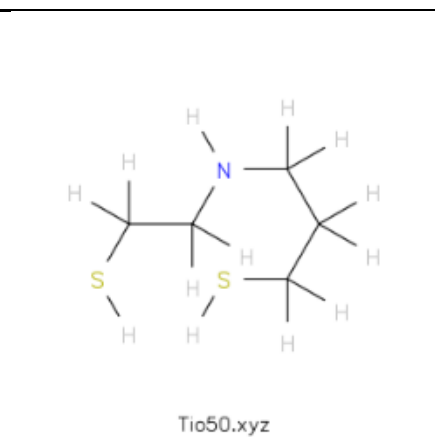
Tio5V2	NCCCCCCCS	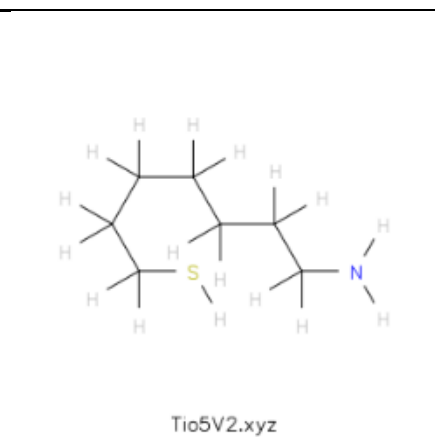
Tio671	C(S)CCN(CCCS)CCCS	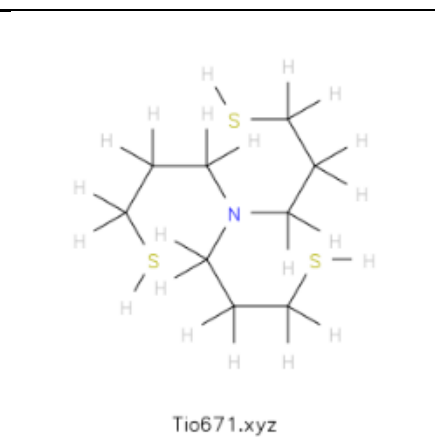
Tio6V2	NCCCCCCCCS	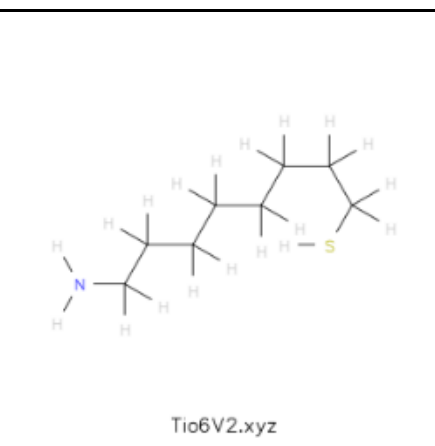
Tio7V2	NCCCCCCCCCS	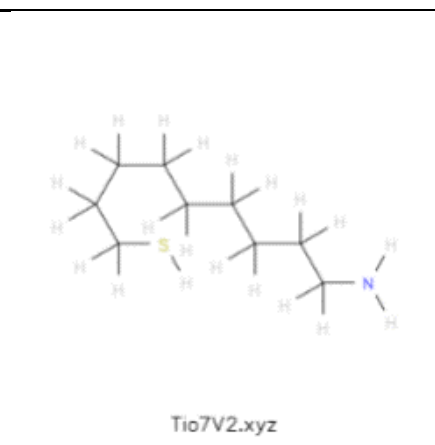

Tio8V2	NCCCCCCCCCCS	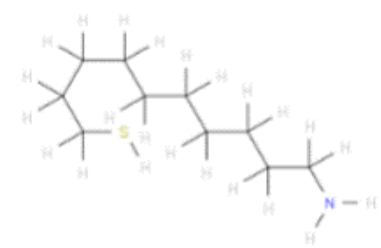 Tio8V2.xyz
TriazoleBr1	N1[N]N=C([CH]1)Br	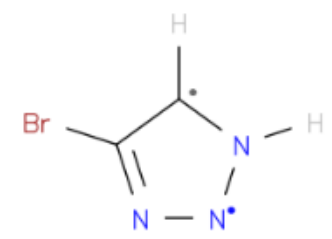 C2H3N3
TriazoleBr2	N1NNC[C@@H]1Br	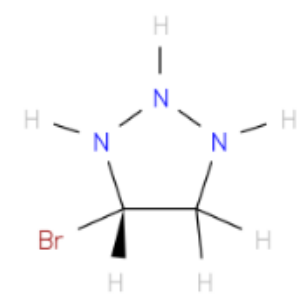 C2H3N3
TriazoleC#N1	N1[N]N=C([CH]1)C#N	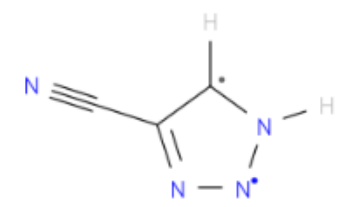 C2H3N3
TriazoleC#N2	N1NNC[C@@H]1C#N	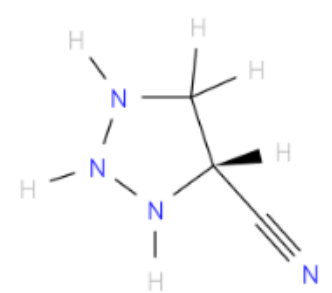 C2H3N3
TriazoleCl1	N1[N]N=C([CH]1)Cl	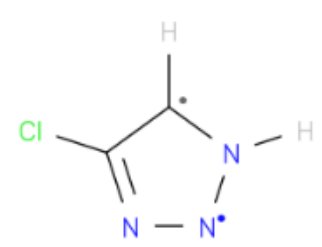 C2H3N3
TriazoleCl2	N1NNC[C@@H]1Cl	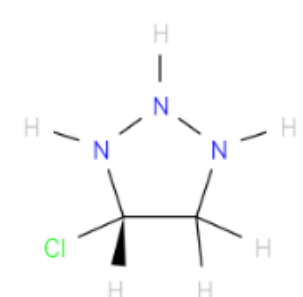 C2H3N3

TriazoleF1	N1[N]N=C([CH]1)F	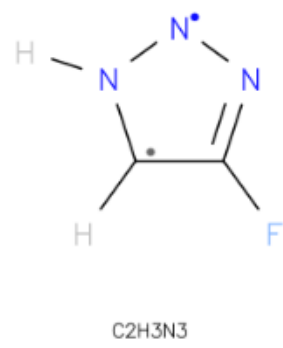
TriazoleF2	N1NNC[C@@H]1F	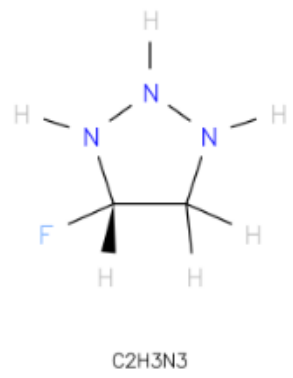
TriazoleI1	N1[N]N=C([CH]1)I	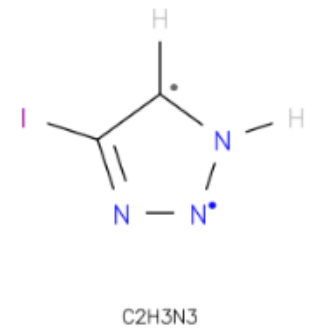
TriazoleI2	N1NNC[C@@H]1I	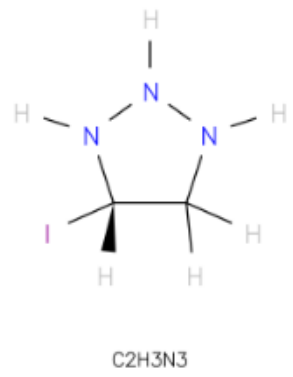
TriazoleN1	N1[N]N=C([CH]1)N	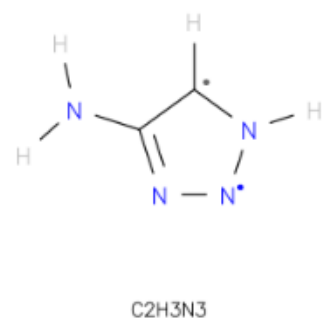
TriazoleN2	N1NNC[C@@H]1N	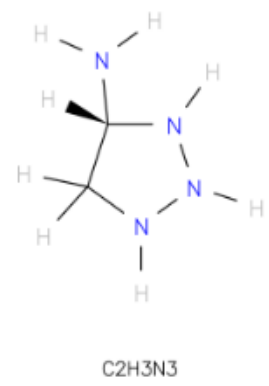
TriazoleNO2_1	N1[N]N=C([CH]1)N(=O)=O	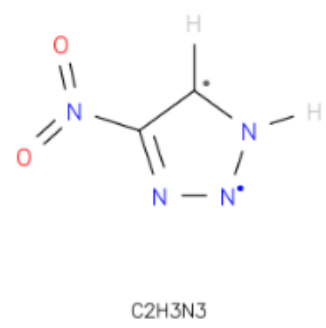

TriazoleNO2_2	N1NNC[C@@H]1N(=O)=O	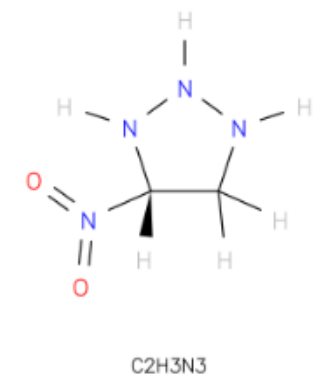
TriazoleO1	N1[N]N=C([CH]1)O	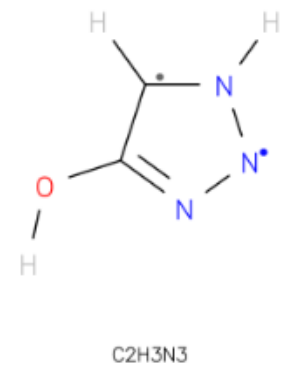
TriazoleO2	[nH]1nncc1O	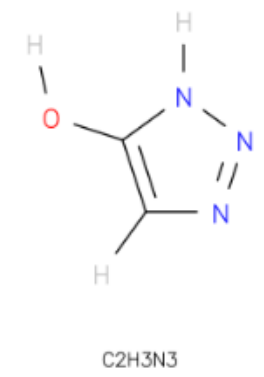
TriazoleS1	N1[N]N=C([CH]1)S	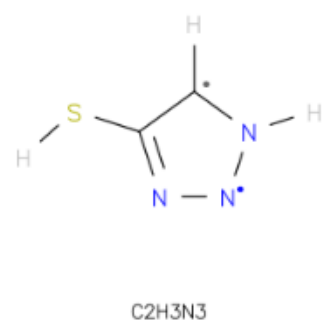
TriazoleS2	N1NNC[C@@H]1S	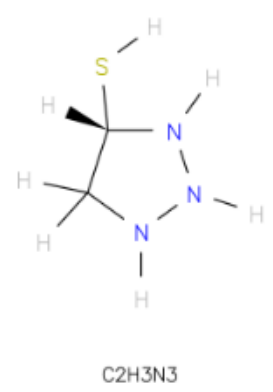

S2. Geometrical, Thermodynamic, and Charge-Transfer Data calculated using the DFT method and the semiempirical methods.

Table S2: Parameters calculated using the CAM-B3LYP/6-311++G(d,p) DFT theory level.

Name	R (Å)	θ (°)	ΔQ	ΔH (kcal/mol)	ΔS (cal/molK)	ΔG (kcal/mol)
Acid0	1.638	138.39	-0.554	8.02	-10.46	18.48
Acid1	1.598	136.41	-0.586	-0.56	-11.24	10.69
Acid2	1.598	136.36	-0.644	-0.57	-11.59	11.02
Acid30	1.658	137.05	-0.424	10.94	-11.47	22.42
Acid430	4.600	179.92	0.007	2.69	-10.03	12.72

Acid50	1.603	135.46	-0.538	8.93	-11.64	20.58
Acid671	1.713	139.09	-0.433	14.16	-10.87	25.03
AcidBr1	1.624	137.63	-0.562	1.65	-10.83	12.48
AcidBr3	3.008	177.79	0.014	-1.90	-9.41	7.51
AcidBr4	1.756	142.61	-0.473	6.93	-11.24	18.17
AcidBr5	4.729	178.94	0.009	-5.95	-9.86	3.92
AcidBr6	3.247	178.86	0.040	0.74	-8.62	9.36
AcidCl0	1.681	140.20	-0.500	4.57	-11.79	16.36
AcidCl1	1.621	137.47	-0.562	1.45	-10.82	12.28
AcidCl2	1.607	136.75	-0.573	-0.92	-12.13	11.22
AcidCl3	2.977	177.66	0.018	-1.70	-8.91	7.22
AcidCl4	1.666	139.42	-0.570	6.09	-12.37	18.47
AcidCl5	4.261	179.24	0.033	-4.80	-9.37	4.57
AcidF0	1.679	140.18	-0.510	4.60	-11.71	16.32
AcidF1	1.620	137.41	-0.572	1.41	-10.68	12.09
AcidF2	1.608	136.83	-0.564	-0.95	-12.25	11.29
AcidF3	2.961	177.56	0.018	-1.13	-8.90	7.77
AcidF4	2.938	176.93	0.015	0.97	-9.14	10.12
AcidF5	3.817	178.57	-0.008	-0.66	-8.83	8.17
AcidF6	1.710	139.70	-0.515	11.24	-12.04	23.28
Acid_Sulf0	1.630	138.04	-0.575	2.97	-10.74	13.71
Acid_Sulf1	1.609	136.94	-0.621	0.58	-11.13	11.72
Acid_Sulf2	1.602	136.51	-0.587	-0.82	-10.75	9.94
Acid_Sulf30	1.668	139.10	-0.507	4.44	-12.06	16.51
Acid_Sulf430	4.051	179.92	0.022	0.52	-9.65	10.17
Acid_Sulf50	1.639	138.14	-0.610	3.23	-11.97	15.21
Acid_Sulf671	1.697	138.46	-0.402	7.54	-12.46	20.00
Acid_Tio0	1.647	138.73	-0.554	3.99	-11.64	15.63
Acid_Tio1	1.616	137.23	-0.559	-0.03	-12.22	12.20
Acid_Tio2	1.598	136.38	-0.640	-0.49	-11.68	11.18
Acid_Tio30	1.753	142.45	-0.434	5.57	-11.04	16.61
Alcohol2	1.601	136.40	-0.584	-1.39	-10.80	9.41
Alcohol30	1.595	135.59	-0.649	0.18	-12.43	12.61
Alcohol430	1.678	137.21	-0.539	8.99	-11.84	20.83
Alcohol50	1.601	136.20	-0.622	-0.80	-11.29	10.48
Alcohol671	1.637	135.50	-0.615	5.86	-12.70	18.56
Aldehyde0	1.632	138.10	-0.567	1.13	-11.85	12.98

Aldehyde1	1.598	136.41	-0.587	-0.73	-11.03	10.30
Aldehyde2	1.602	136.49	-0.587	-1.85	-11.99	10.15
Aldehyde30	1.688	139.87	-0.522	2.13	-11.64	13.77
Aldehyde430	3.222	177.68	0.015	-0.53	-9.52	9.00
Aldehyde50	1.628	137.28	-0.490	3.79	-12.22	16.02
Aldehyde671	1.695	138.35	-0.432	4.08	-11.88	15.97
Amide0	1.621	137.43	-0.584	5.72	-11.10	16.83
Amide1	1.604	136.65	-0.623	-0.22	-10.71	10.49
Amide2	1.600	136.37	-0.606	-0.18	-9.98	9.80
Amide30	1.666	139.13	-0.517	11.72	-11.17	22.89
Amide430	2.983	176.94	0.022	1.22	-9.62	10.84
Amide50	1.623	137.33	-0.643	5.39	-11.70	17.10
Amide671	1.734	139.14	-0.444	9.72	-11.05	20.78
Amine0	1.600	136.50	-0.639	-0.62	-10.76	10.14
Amine1	1.594	136.18	-0.649	-1.28	-11.08	9.81
Amine2	1.593	136.15	-0.646	-1.41	-11.18	9.77
Amine30	1.579	135.52	-0.630	-2.22	-11.24	9.03
Amine430	1.649	136.01	-0.541	5.37	-12.04	17.40
Amine50	1.593	135.76	-0.707	-0.99	-11.70	10.71
Amine671	1.633	135.34	-0.685	8.05	-12.31	20.37
Anhydride0	1.637	138.42	-0.541	2.80	-11.36	14.16
Anhydride1	1.617	137.25	-0.566	-0.98	-12.10	11.13
Anhydride2	1.606	136.70	-0.574	0.08	-10.36	10.45
Anhydride30	2.889	177.11	0.019	0.32	-9.77	10.10
Anhydride430	3.408	177.73	0.033	-1.82	-9.52	7.70
Anhydride50	1.751	141.94	-0.452	8.26	-11.36	19.62
Anhydride671	1.692	138.20	-0.463	9.10	-12.14	21.24
Br0	3.803	179.94	0.003	0.80	-6.99	7.80
Br1	1.619	137.39	-0.575	0.89	-10.91	11.81
Br2	1.608	136.80	-0.589	-0.24	-10.46	10.23
Br3	4.171	179.66	0.003	0.07	-10.01	10.08
Br4	1.687	140.33	-0.543	18.28	-12.61	30.90
Br5	3.727	179.96	0.022	-0.13	-9.11	8.98
Br6	1.709	139.15	-0.462	12.28	-11.92	24.21
Cl0	3.015	178.29	0.010	-1.05	-9.90	8.84
Cl1	1.618	137.35	-0.624	0.68	-11.08	11.76
Cl2	1.609	136.88	-0.568	-0.87	-12.08	11.22

Cl3	3.915	179.55	-0.004	0.64	-8.59	9.23
Cl5	3.813	179.68	0.010	0.41	-7.98	8.39
Cl6	3.013	177.79	0.041	2.57	-8.89	11.47
Cyan0	1.695	140.99	-0.464	5.60	-10.35	15.95
Cyan1	1.625	137.74	-0.590	2.29	-10.91	13.19
Cyan2	1.610	136.92	-0.674	1.43	-11.07	12.50
Cyan30	2.996	178.10	0.008	-0.14	-8.13	7.99
Cyan430	3.214	179.18	0.006	-0.39	-9.98	9.60
Cyan50	1.723	141.91	-0.533	8.31	-10.51	18.82
Cyan671	4.037	179.93	0.029	-0.11	-9.78	9.66
Ester0	1.637	138.27	-0.567	1.83	-11.97	13.81
Ester1	1.611	136.94	-0.576	-0.34	-12.14	11.80
Ester2	1.600	136.45	-0.593	-0.93	-11.01	10.08
Ester30	1.647	138.56	-0.566	6.47	-12.48	18.95
Ester430	3.037	176.83	0.057	-0.20	-9.92	9.72
Ester50	1.638	137.65	-0.557	4.31	-11.26	15.57
Ester671	1.692	138.10	-0.474	6.20	-11.95	18.15
Ether0	1.629	137.91	-0.556	3.33	-10.48	13.82
Ether1	1.602	136.55	-0.590	-1.57	-10.53	8.96
Ether2	1.599	136.37	-0.598	-1.38	-10.77	9.40
Ether30	1.642	138.27	-0.586	7.91	-10.99	18.90
Ether430	3.755	176.84	0.006	-1.09	-8.90	7.81
Ether50	1.648	137.64	-0.521	2.06	-11.40	13.46
Ether671	1.687	137.87	-0.446	7.00	-11.38	18.38
F0	3.004	177.99	0.007	-1.71	-6.96	5.25
F1	1.615	137.22	-0.622	-0.05	-10.94	10.89
F2	1.596	136.26	-0.600	-0.86	-11.03	10.17
F3	3.148	179.14	0.019	-0.79	-8.23	7.44
F4	3.084	178.54	0.009	1.29	-7.86	9.15
F5	3.444	179.90	0.022	-0.68	-7.87	7.18
F6	1.696	138.80	-0.497	7.95	-11.58	19.53
Nitro0	1.618	137.67	-0.612	3.51	-10.87	14.37
Nitro1	1.614	137.04	-0.571	1.11	-10.87	11.99
Nitro3	1.596	136.28	-0.635	-0.75	-9.72	8.98
Nitro30	1.654	138.96	-0.614	6.04	-10.18	16.22
Nitro50	1.628	137.74	-0.657	3.23	-11.78	15.01
Nitro671	1.636	135.99	-0.667	12.23	-13.27	25.51

Tio0	1.601	136.54	-0.599	-0.47	-11.02	10.56
Tio1	1.602	136.51	-0.592	-0.87	-10.76	9.90
Tio2	1.599	136.36	-0.591	-1.33	-10.64	9.32
Tio30	1.610	136.75	-0.676	1.00	-11.55	12.56
Tio430	1.715	138.65	-0.459	11.56	-11.67	23.23
Tio50	1.606	136.46	-0.630	0.01	-11.69	11.70
Tio671	1.658	136.52	-0.498	3.46	-12.21	15.67
Acid3V2	1.599	136.35	-0.588	-1.23	-10.99	9.76
Acid4V2	1.599	136.31	-0.594	-2.69	-13.24	10.56
Acid6V2	1.596	136.18	-0.593	0.34	-11.67	12.02
Acid7V2	1.596	136.20	-0.607	1.27	-11.63	12.90
Acid8V2	1.600	136.34	-0.573	-2.22	-12.82	10.60
Acid_Halid3V2	1.602	136.49	-0.586	-1.10	-10.69	9.59
Acid_Halid4V2	1.599	136.29	-0.591	-2.41	-11.24	8.84
Acid_Halid5V2	1.601	136.40	-0.587	0.19	-12.25	12.44
Acid_Halid6V2	1.598	136.25	-0.587	-3.41	-10.97	7.56
Acid_Halid7V2	1.595	136.16	-0.598	-1.63	-9.34	7.71
Acid_Halid8V2	1.595	136.18	-0.595	-1.61	-11.25	9.65
Acid_Sulf3V2	1.600	136.39	-0.595	-1.62	-12.34	10.73
Acid_Sulf4V2	1.597	136.26	-0.597	-1.93	-10.63	8.71
Acid_Sulf5V2	1.599	136.35	-0.579	-1.56	-12.81	11.26
Acid_Sulf6V2	1.598	136.26	-0.588	-2.19	-12.69	10.50
Acid_Sulf7V2	1.595	136.17	-0.596	1.79	-12.43	14.22
Acid_Sulf8V2	1.599	136.33	-0.573	-2.24	-12.74	10.51
Acid_Tio3V2	1.598	136.33	-0.592	-1.24	-10.86	9.61
Acid_Tio4V2	1.596	136.23	-0.593	-1.46	-11.16	9.70
Acid_Tio5V2	1.597	136.22	-0.602	-0.01	-11.44	11.43
Acid_Tio6V2	1.599	136.32	-0.586	-0.69	-12.52	11.83
Acid_Tio7V2	1.600	136.32	-0.588	0.61	-13.10	13.71
Acid_Tio8V2	1.597	136.27	-0.590	-2.21	-12.46	10.25
Alcohol3V2	1.597	136.26	-0.594	-1.48	-10.95	9.47
Alcohol4V2	1.591	136.04	-0.612	-0.10	-11.18	11.09
Alcohol5V2	1.596	136.20	-0.595	-0.87	-10.91	10.04
Alcohol6V2	1.598	136.26	-0.587	-2.28	-12.27	9.99
Alcohol7V2	1.597	136.22	-0.588	-2.27	-12.40	10.14
Alcohol8V2	1.598	136.27	-0.589	-1.66	-10.39	8.74
Amide3V2	1.602	136.45	-0.579	-2.64	-13.28	10.65

Amide4V2	1.597	136.25	-0.593	-1.61	-10.92	9.32
Amide5V2	1.594	136.11	-0.636	0.74	-10.96	11.70
Amide6V2	1.598	136.31	-0.588	1.18	-10.73	11.91
Amide7V2	1.600	136.34	-0.571	-2.84	-13.41	10.58
Amide8V2	1.597	136.22	-0.593	-2.84	-13.39	10.56
Amine3V2	1.598	136.26	-0.590	-1.60	-10.66	9.06
Amine4V2	1.596	136.18	-0.599	-0.79	-10.57	9.78
Amine5V2	1.596	136.21	-0.595	-1.48	-11.96	10.48
Amine6V2	1.598	136.26	-0.587	-1.66	-10.88	9.23
Amine7V2	1.599	136.33	-0.572	-2.29	-12.25	9.96
Amine8V2	1.597	136.22	-0.591	-2.20	-12.39	10.19
Anhydride3V2	1.601	136.42	-0.589	-1.02	-13.02	12.01
Anhydride4V2	1.598	136.30	-0.594	-1.45	-11.20	9.75
Anhydride5V2	1.597	136.24	-0.598	-1.00	-11.36	10.37
Anhydride7V2	1.598	136.31	-0.587	1.57	-11.75	13.32
Anhydride8V2	1.595	136.13	-0.601	-0.08	-11.02	10.94
Cyan3V2	1.601	136.47	-0.587	-0.83	-10.95	10.12
Cyan4V2	1.600	136.35	-0.587	-1.20	-12.15	10.96
Cyan5V2	1.597	136.22	-0.603	-1.34	-12.33	10.99
Cyan6V2	1.597	136.24	-0.590	-1.63	-10.82	9.19
Cyan7V2	1.592	136.05	-0.607	-1.61	-11.15	9.54
Cyan8V2	1.601	136.37	-0.572	-2.23	-12.15	9.93
Ester3V2	1.599	136.33	-0.596	-0.50	-10.82	10.32
Ester4V2	1.597	136.24	-0.591	-1.54	-11.02	9.49
Ester5V2	1.600	136.38	-0.579	-1.33	-12.55	11.22
Ester6V2	1.598	136.25	-0.588	-2.28	-12.41	10.14
Ester7V2	1.600	136.38	-0.579	-1.40	-12.59	11.19
Ester8V2	1.597	136.23	-0.592	-2.26	-12.53	10.27
Ether3V2	1.600	136.37	-0.588	-1.96	-12.16	10.19
Ether4V2	1.599	136.32	-0.592	-1.26	-12.47	11.22
Ether5V2	1.596	136.19	-0.599	-0.84	-10.75	9.91
Ether6V2	1.599	136.32	-0.577	-2.16	-12.52	10.36
Ether7V2	1.596	136.23	-0.588	-1.63	-11.00	9.38
Ether8V2	1.594	136.13	-0.601	-1.51	-11.45	9.94
Halid2V2	1.593	136.15	-0.603	-0.86	-11.06	10.20
Halid3V2	1.599	136.34	-0.589	-1.48	-10.80	9.33
Halid4V2	1.594	136.19	-0.643	-1.48	-11.12	9.64

Halid5V2	1.600	136.38	-0.587	-1.06	-10.95	9.89
Halid6V2	1.600	136.35	-0.584	-1.37	-12.46	11.10
Halid7V2	1.592	136.07	-0.604	-1.84	-11.27	9.43
Halid8V2	1.596	136.17	-0.591	-1.62	-11.02	9.41
Ketone3V2	1.600	136.36	-0.589	-1.30	-10.64	9.34
Ketone4V2	1.600	136.31	-0.588	-1.50	-10.65	9.15
Ketone5V2	1.591	136.04	-0.649	0.02	-11.49	11.51
Ketone6V2	1.595	136.14	-0.595	-0.12	-11.31	11.20
Ketone7V2	1.599	136.34	-0.570	-2.21	-12.45	10.25
Ketone8V2	1.600	136.35	-0.572	-2.21	-12.66	10.46
Nitro4V2	1.596	136.19	-0.607	0.50	-10.17	10.66
Nitro5V2	1.595	136.19	-0.599	-1.10	-9.47	8.38
Nitro6V2	1.590	135.99	-0.611	-1.64	-11.25	9.62
Nitro7V2	1.596	136.20	-0.595	-1.67	-11.44	9.78
Nitro8V2	1.596	136.18	-0.598	-1.76	-11.45	9.70
Tio3V2	1.597	136.23	-0.591	-1.50	-10.93	9.43
Tio4V2	1.596	136.19	-0.602	-0.83	-10.75	9.92
Tio5V2	1.597	136.20	-0.603	-1.44	-12.25	10.81
Tio6V2	1.599	136.32	-0.572	-2.23	-12.47	10.24
Tio7V2	1.596	136.16	-0.594	-1.68	-10.87	9.20
Tio8V2	1.597	136.21	-0.591	-1.67	-11.18	9.52
AzolidineBr1	1.660	138.85	-0.521	25.04	-13.14	38.19
AzolidineCl1	1.614	137.42	-0.601	20.60	-12.79	33.40
PyrimidineS1	1.659	138.99	-0.409	11.01	-10.87	21.89
ThiazoleO1	1.609	136.97	-0.431	9.07	-12.40	21.47
PyridineS1	1.631	134.85	-0.353	8.53	-12.40	20.93
ThiazoleS3	1.599	137.37	-0.478	9.52	-11.32	20.85
ThiazoleC#N2	1.667	141.05	-0.390	10.09	-10.31	20.41
AzolidineF1	1.643	138.08	-0.545	7.59	-11.98	19.57
ThiazoleS1	1.561	135.81	-0.487	7.26	-11.48	18.74
ThiazoleCl2	1.609	138.32	-0.446	7.39	-10.70	18.09
ThiazoleF2	1.609	138.38	-0.453	7.28	-10.66	17.95
PiperidineNO2_1	1.732	141.33	-0.426	6.63	-11.18	17.81
PyrimidineS3	1.669	140.20	-0.408	6.98	-10.66	17.65
PiperidineBr1	1.647	137.83	-0.589	6.39	-11.07	17.46
PyrimidineO3	1.662	139.83	-0.419	6.59	-10.60	17.20
PiperidineCl1	1.649	137.91	-0.595	6.16	-10.97	17.14

PyridineNO2_2	1.676	140.50	-0.340	6.32	-10.76	17.08
ThiazoleN3	1.572	135.28	-0.496	5.97	-11.10	17.08
PyrazineO2	1.730	142.71	-0.342	6.84	-10.17	17.02
PyrimidineN2	1.577	134.10	-0.531	5.13	-11.87	17.01
PyridineNO2_3	1.675	140.26	-0.345	6.20	-10.78	16.98
ThiazoleS2	1.593	137.51	-0.466	6.20	-10.75	16.96
PyrimidineO2	1.558	133.76	-0.418	5.58	-11.34	16.92
PyridineC#N2	1.657	139.54	-0.359	5.62	-10.78	16.40
PyrazineN1	1.580	134.50	-0.484	4.98	-11.23	16.21
ThiazoleO2	1.585	136.99	-0.477	5.13	-11.04	16.17
PyridineC#N3	1.650	139.13	-0.365	5.14	-10.87	16.01
AzolidineC#N1	1.639	138.41	-0.525	3.78	-11.88	15.66
PyrimidineN1	1.581	134.26	-0.471	4.27	-11.28	15.55
PyrimidineN3	1.632	138.23	-0.452	4.67	-10.66	15.32
PyrazineN2	1.657	139.28	-0.400	4.84	-10.14	14.98
PyridineCl2	1.631	138.21	-0.414	3.95	-10.87	14.83
PyridineBr2	1.631	138.16	-0.422	3.92	-10.90	14.82
PyridineF2	1.631	138.20	-0.419	3.92	-10.83	14.75
ThiazoleN2	1.573	136.24	-0.486	3.58	-10.79	14.37
PyridineBr3	1.610	137.25	-0.437	2.86	-11.04	13.91
PiperidineNO2_2	1.650	137.67	-0.529	2.54	-11.36	13.90
PyridineCl3	1.610	137.26	-0.426	2.74	-11.00	13.74
PiperidineS1	1.621	136.47	-0.642	2.32	-11.31	13.64
PyridineS2	1.614	137.21	-0.403	2.34	-10.97	13.31
PyridineF3	1.607	137.10	-0.449	2.39	-10.92	13.31
PyridineO1	1.547	132.60	-0.401	1.31	-11.56	12.87
AzolidineNO2_2	1.604	136.78	-0.605	1.36	-11.50	12.85
AzolidineS1	1.612	136.72	-0.558	1.60	-11.25	12.85
PiperidineBr2	1.645	137.24	-0.543	1.41	-11.25	12.67
PyridineO2	1.611	136.99	-0.431	1.79	-10.88	12.67
PyridineN1	1.558	132.56	-0.508	0.87	-11.06	11.93
AzolidineO1	1.580	135.58	-0.609	-0.43	-12.18	11.76
PiperidineNO2_3	1.643	137.05	-0.573	-0.06	-11.20	11.14
PyridineS3	1.581	135.84	-0.456	0.08	-11.02	11.11
AzolidineCl2	1.609	136.78	-0.601	-0.95	-11.96	11.01
PiperidineS2	1.634	136.63	-0.557	-0.28	-11.24	10.96
PiperidineCl2	1.606	136.68	-0.594	0.02	-10.84	10.86

PyridineN2	1.600	136.22	-0.445	-0.07	-10.87	10.81
ThiazoleN1	1.517	132.83	-0.535	-0.52	-11.30	10.78
AzolidineC#N2	1.611	136.89	-0.610	0.06	-10.63	10.70
PiperidineBr3	1.640	136.84	-0.573	-0.69	-11.17	10.49
AzolidineF2	1.641	137.20	-0.576	-0.45	-10.92	10.47
PiperidineO3	1.588	135.86	-0.601	-0.82	-10.99	10.17
AzolidineBr2	1.609	136.77	-0.602	-0.70	-10.83	10.13
PyridineO3	1.571	135.32	-0.480	-1.10	-11.04	9.94
PiperidineS3	1.637	136.65	-0.576	-1.31	-11.15	9.85
PiperidineO1	1.603	135.83	-0.550	-1.52	-11.36	9.85
ImidazoleC#N1	3.783	179.99	0.010	-0.56	-9.85	9.29
ImidazoleS1	3.942	179.98	0.020	-0.69	-9.93	9.24
PyrazoleF1	3.421	179.84	0.007	-0.73	-9.96	9.23
PyrazineO1	2.976	178.05	0.019	1.27	-7.91	9.18
PiperidineO2	1.595	136.15	-0.624	-1.77	-10.89	9.12
ThiazoleO3	2.944	177.88	0.005	-0.24	-9.32	9.08
PyridineBr1	3.063	178.36	0.019	-0.58	-9.52	8.94
ThiazoleNO2_3	3.076	178.26	0.002	-0.93	-9.81	8.88
PyrazineCl1	3.012	178.45	0.009	-0.75	-9.59	8.85
PyridineCl1	3.026	178.22	0.017	-0.71	-9.53	8.82
PyrimidineC#N2	2.966	178.29	0.005	-0.67	-9.47	8.81
PyrroleO2	3.281	179.50	0.006	-0.15	-8.95	8.80
ImidazoleS2	3.661	179.98	0.013	-0.15	-8.93	8.79
ThiazoleBr3	2.979	178.21	0.005	-0.70	-9.46	8.76
PyrimidineNO2_2	3.066	178.21	0.005	-0.93	-9.68	8.75
PyrimidineCl2	3.017	178.40	-0.012	-0.57	-9.31	8.75
PyridineNO2_1	3.084	178.10	0.007	-0.96	-9.70	8.73
PyridineC#N1	2.945	177.92	0.025	-0.74	-9.47	8.73
ThiazoleNO2_1	3.060	178.82	0.009	-0.54	-9.25	8.71
TriazoleO2	3.818	179.93	-0.002	0.06	-8.63	8.69
ImidazoleO1	3.513	179.94	0.009	0.03	-8.65	8.69
PyrimidineBr2	3.030	178.43	-0.003	-0.50	-9.19	8.69
ThiazoleBr1	2.977	178.22	0.006	-0.65	-9.31	8.67
ImidazoleC#N2	3.732	179.97	0.007	-0.01	-8.68	8.67
PyrazoleBr2	3.111	178.89	0.000	-0.32	-8.98	8.67
TriazoleS1	3.033	178.86	0.003	-0.47	-9.10	8.64
PyridineF1	2.965	178.02	0.022	-0.74	-9.38	8.64

PyrroleNO2_2	3.511	179.99	0.008	-0.05	-8.68	8.64
PyrimidineF2	2.978	178.35	0.007	-0.64	-9.25	8.62
ImidazoleN2	3.453	179.94	0.008	-0.13	-8.74	8.61
ImidazoleNO2_2	3.898	179.97	0.005	0.05	-8.54	8.60
PyrimidineC#N3	2.886	177.70	0.004	-0.84	-9.40	8.56
PyrazoleNO2_2	3.101	178.86	0.004	-0.40	-8.92	8.53
PyrimidineNO2_3	2.891	177.78	0.016	-0.88	-9.40	8.53
ImidazoleF2	3.652	179.97	0.007	0.03	-8.48	8.51
ThiazoleNO2_2	2.915	177.88	0.007	-0.79	-9.28	8.49
ImidazoleBr1	3.512	179.98	0.010	-0.08	-8.54	8.46
ThiazoleCl1	2.962	178.13	0.003	-0.75	-9.20	8.45
PyrazoleCl2	3.410	179.83	-0.001	-0.18	-8.62	8.45
PyrroleN2	3.287	179.45	0.005	0.40	-8.03	8.43
TriazoleCl1	3.046	178.75	0.003	-0.43	-8.85	8.42
PyrimidineCl3	2.873	177.49	0.003	-0.86	-9.28	8.42
TriazoleF1	3.049	178.78	0.001	-0.43	-8.84	8.41
PyrazoleS1	3.083	178.72	-0.006	-0.53	-8.94	8.41
PyrroleBr2	3.341	179.68	0.008	0.48	-7.92	8.41
PyrroleS1	3.338	179.73	0.007	0.36	-8.01	8.38
PyrazineBr2	2.876	177.56	0.009	-0.91	-9.25	8.35
PyrroleN1	3.324	179.57	0.009	0.35	-7.96	8.32
AzolidineNO2_1	3.038	177.33	0.005	-0.45	-8.73	8.29
PyrroleBr1	3.323	179.74	0.008	0.41	-7.86	8.28
PyrroleF2	3.321	179.65	0.006	0.44	-7.83	8.27
TriazoleC#N1	2.954	178.47	-0.004	-0.67	-8.94	8.27
ThiazoleF1	2.963	178.19	0.009	-0.68	-8.94	8.26
PyrroleCl1	3.317	179.74	0.007	0.36	-7.89	8.26
PyrroleCl2	3.362	179.68	0.008	0.46	-7.79	8.24
PyrimidineNO2_1	3.076	178.18	0.006	-0.36	-8.57	8.22
TriazoleN1	3.016	178.71	0.003	-0.54	-8.75	8.21
PyrroleO1	4.405	179.56	0.000	-0.11	-8.31	8.21
ImidazoleBr2	3.577	179.98	0.003	0.55	-7.56	8.12
PyrimidineBr1	3.034	178.46	0.019	-0.02	-8.11	8.10
PyrazineNO2_1	3.058	178.22	0.008	-0.37	-8.44	8.08
PyrazoleNO2_1	3.096	178.24	-0.002	-0.26	-8.32	8.05
PyrimidineC#N1	2.958	178.26	0.020	-0.11	-8.15	8.04
PyrroleC#N2	3.427	179.88	0.004	0.53	-7.46	8.00

PyrimidineF1	2.954	178.21	0.018	-0.13	-8.12	7.99
ImidazoleCl1	3.536	179.97	0.009	0.49	-7.49	7.99
PyrimidineBr3	2.876	177.51	0.002	-0.85	-8.83	7.99
PyrimidineS2	2.963	178.08	0.023	-0.28	-8.25	7.97
ThiazoleC#N3	2.939	178.13	0.002	-0.11	-8.08	7.96
PyrimidineCl1	3.007	178.35	0.016	-0.11	-8.07	7.96
ThiazoleF3	2.946	178.17	0.007	-0.12	-8.07	7.95
PyrimidineO1	2.953	177.89	0.021	-0.16	-8.10	7.94
TriazoleNO2_1	2.955	178.56	0.001	-0.04	-7.92	7.88
ImidazoleNO2_1	3.683	179.92	0.013	0.59	-7.28	7.87
PyrazoleC#N1	2.932	178.38	-0.002	-0.12	-7.99	7.87
PyrazoleF2	2.906	177.99	-0.007	-0.26	-8.11	7.86
PyrroleF1	3.352	179.71	0.012	0.43	-7.39	7.82
PyrazineBr1	3.035	178.51	0.014	-0.03	-7.83	7.80
ThiazoleCl3	2.961	178.17	0.001	-0.19	-7.98	7.79
AzolidineO2	1.587	135.93	-0.614	-2.12	-9.90	7.78
PyrazoleCl1	3.085	178.86	-0.006	0.05	-7.72	7.77
PyrazineS1	2.961	178.04	0.012	-0.28	-8.04	7.76
TriazoleBr1	3.003	178.58	-0.006	0.06	-7.69	7.76
PyrazoleN1	3.100	178.81	0.004	0.09	-7.66	7.75
PiperidineN2	1.587	135.74	-0.633	-3.11	-10.84	7.74
AzolidineS2	1.587	135.87	-0.648	-3.35	-11.08	7.74
PyrazineF1	2.977	178.36	0.018	-0.11	-7.84	7.73
ImidazoleCl2	3.548	179.97	0.003	0.47	-7.22	7.69
PyrazineC#N1	2.966	178.32	0.015	-0.15	-7.81	7.67
PyrazoleBr1	3.102	178.98	-0.001	0.14	-7.52	7.67
ImidazoleN1	3.516	179.91	0.009	0.63	-7.02	7.65
PyrazoleO1	3.112	178.99	0.004	0.16	-7.46	7.63
PyrroleNO2_1	3.556	179.96	0.008	0.57	-6.99	7.56
ThiazoleC#N1	2.951	178.19	0.007	-0.09	-7.63	7.55
TriazoleO1	2.985	178.40	-0.006	0.00	-7.51	7.52
PyrazoleN2	3.125	178.87	-0.004	0.18	-7.32	7.50
PyrazineS2	2.863	177.32	0.014	-0.32	-7.81	7.49
PyrazineNO2_2	2.888	177.78	0.016	-0.29	-7.70	7.41
ImidazoleF1	3.546	179.95	0.012	0.55	-6.85	7.40
PyrazoleO2	3.769	179.99	0.001	0.72	-6.64	7.36
PiperidineN3	1.583	135.59	-0.603	-3.72	-11.05	7.33

PyrazineC#N2	2.884	177.71	0.010	-0.30	-7.56	7.27
ImidazoleO2	4.797	179.70	0.001	0.37	-6.84	7.22
PyrazineCl2	2.876	177.56	0.008	-0.34	-7.52	7.18
PyrazoleC#N2	2.913	177.93	-0.007	-0.15	-7.32	7.17
PyrimidineF3	2.878	177.52	0.002	-0.27	-7.43	7.16
PyrazineF2	2.874	177.54	0.007	-0.31	-7.41	7.10
AzolidineN1	1.564	134.85	-0.625	-3.93	-10.84	6.91
AzolidineN2	1.584	135.65	-0.640	-3.91	-10.38	6.47
PyrazoleS2	2.921	178.02	-0.004	-1.02	-7.40	6.38
PyrroleS2	4.217	179.87	0.007	-0.50	-6.73	6.23
PyridineN3	1.539	133.63	-0.528	-5.35	-11.12	5.78
PiperidineN1	1.575	135.06	-0.660	-5.02	-10.62	5.61

Table S3: Parameters calculated using the AM1 method.

Name	R (Å)	θ (°)	ΔQ	ΔH (kcal/mol)	ΔS (cal/molK)	ΔG (kcal/mol)
Acid0	1.551	129.64	-0.593	1.41	12.65	10.06
Acid1	1.539	128.56	-0.625	1.01	6.83	11.39
Acid2	1.535	128.10	-0.641	1.27	7.76	11.38
Acid30	1.600	132.56	-0.511	1.32	6.41	11.83
Acid3V2	1.539	128.56	-0.634	1.46	15.93	9.13
Acid430	1.760	140.37	-0.347	2.06	15.12	9.98
Acid4V2	1.533	128.15	-0.639	1.44	14.32	9.59
Acid50	1.583	130.91	-0.537	1.38	7.76	11.49
Acid671	1.613	131.06	-0.534	1.39	3.80	12.68
Acid6V2	1.536	128.44	-0.631	1.05	7.50	11.23
Acid7V2	1.535	128.23	-0.638	1.04	6.52	11.51
Acid8V2	1.536	128.26	-0.637	1.49	14.14	9.70
AcidBr1	1.540	128.72	-0.622	1.37	12.99	9.92
AcidBr2	1.534	128.40	-0.630	1.39	10.31	10.74
AcidBr3	1.600	132.29	-0.521	1.51	11.77	10.42
AcidBr4	1.589	131.41	-0.540	1.52	13.82	9.83
AcidBr5	5.773	179.89	0.000	3.14	33.25	5.64
AcidBr6	1.624	131.06	-0.522	1.52	9.99	10.96
AcidCl0	1.550	129.81	-0.588	1.27	11.54	10.26
AcidCl1	1.539	128.73	-0.622	1.42	13.89	9.70
AcidCl2	1.535	128.37	-0.633	1.34	11.04	10.47

AcidCl3	1.605	132.80	-0.511	1.71	14.87	9.70
AcidCl4	1.587	131.33	-0.544	1.47	10.92	10.64
AcidCl5	4.836	179.76	0.000	3.17	36.56	4.69
AcidCl6	1.600	130.09	-0.545	1.36	7.47	11.55
AcidF0	1.545	130.32	-0.591	1.13	9.10	10.84
AcidF1	1.541	128.79	-0.619	1.45	13.20	9.93
AcidF2	1.537	128.44	-0.631	1.41	10.08	10.83
AcidF3	1.619	133.74	-0.485	1.58	12.45	10.29
AcidF4	1.582	131.87	-0.542	1.53	13.45	9.94
AcidF5	5.500	179.80	0.000	3.09	35.25	5.00
AcidF6	1.601	130.52	-0.545	1.68	17.93	8.75
AcidI0	1.547	129.57	-0.597	1.29	8.92	11.05
AcidI1	1.540	128.67	-0.623	1.42	12.26	10.19
AcidI2	1.529	128.55	-0.633	1.32	11.67	10.26
AcidI3	1.600	131.93	-0.528	1.46	10.92	10.62
AcidI4	1.589	131.61	-0.539	1.53	11.10	10.64
AcidI5	4.375	179.80	0.000	2.97	31.35	6.05
AcidI6	1.595	129.83	-0.554	1.32	5.84	12.00
Acid_Halid3V2	1.535	128.26	-0.638	1.29	11.63	10.24
Acid_Halid4V2	1.533	128.12	-0.638	1.37	13.10	9.89
Acid_Halid5V2	1.527	128.77	-0.641	1.37	16.07	9.00
Acid_Halid6V2	1.533	128.17	-0.640	1.35	10.59	10.61
Acid_Halid7V2	1.533	128.17	-0.640	1.45	13.62	9.81
Acid_Halid8V2	1.536	128.29	-0.637	1.47	13.67	9.81
Acid_Sulf0	1.542	128.58	-0.623	1.38	11.37	10.41
Acid_Sulf1	1.536	128.21	-0.640	1.47	12.78	10.08
Acid_Sulf2	1.534	128.30	-0.635	1.67	14.29	9.83
Acid_Sulf30	1.589	131.12	-0.540	0.86	3.38	12.28
Acid_Sulf3V2	1.534	128.24	-0.636	1.55	12.55	10.22
Acid_Sulf430	1.604	130.59	-0.537	2.15	18.30	9.12
Acid_Sulf4V2	1.535	128.23	-0.637	1.65	12.69	10.29
Acid_Sulf50	1.575	130.13	-0.571	1.53	8.92	11.29
Acid_Sulf5V2	1.534	128.17	-0.638	1.31	9.57	10.87
Acid_Sulf671	1.597	129.54	-0.563	1.25	3.90	12.51
Acid_Sulf6V2	1.535	128.28	-0.637	1.32	6.44	11.82
Acid_Sulf7V2	1.536	128.30	-0.637	0.82	7.85	10.90
Acid_Sulf8V2	1.537	128.20	-0.637	1.37	11.93	10.24

Acid_Tio0	1.538	129.91	-0.607	1.48	13.62	9.84
Acid_Tio1	1.541	128.54	-0.625	1.41	12.55	10.09
Acid_Tio2	1.536	128.07	-0.640	1.28	12.30	10.03
Acid_Tio30	1.588	131.55	-0.539	1.50	12.00	10.34
Acid_Tio3V2	1.534	128.21	-0.638	1.48	12.35	10.22
Acid_Tio430	1.642	132.66	-0.483	1.68	11.24	10.75
Acid_Tio4V2	1.535	128.23	-0.637	1.42	15.24	9.30
Acid_Tio50	1.592	131.68	-0.537	1.47	10.07	10.89
Acid_Tio5V2	1.535	128.22	-0.639	1.19	9.44	10.80
Acid_Tio671	1.595	129.61	-0.559	1.35	3.06	12.85
Acid_Tio6V2	1.536	128.17	-0.637	1.05	10.11	10.46
Acid_Tio7V2	1.536	128.39	-0.638	1.13	10.66	10.38
Acid_Tio8V2	1.535	128.12	-0.639	1.49	14.53	9.58
Alcohol0	1.536	128.35	-0.633	1.41	14.16	9.61
Alcohol1	1.534	128.27	-0.636	1.63	17.95	8.70
Alcohol2	1.536	128.25	-0.636	1.41	14.10	9.63
Alcohol30	1.554	128.75	-0.615	1.64	13.98	9.89
Alcohol3V2	1.534	128.21	-0.638	1.41	10.91	10.58
Alcohol430	1.598	129.39	-0.565	1.64	9.94	11.10
Alcohol4V2	1.534	128.19	-0.638	1.12	12.89	9.70
Alcohol50	1.567	129.56	-0.592	1.66	15.80	9.37
Alcohol5V2	1.535	128.21	-0.638	1.24	13.11	9.76
Alcohol671	1.578	128.12	-0.595	1.54	12.61	10.20
Alcohol6V2	1.534	128.27	-0.638	1.53	17.99	8.58
Alcohol7V2	1.534	128.21	-0.639	1.71	19.63	8.28
Alcohol8V2	1.535	128.26	-0.637	1.38	13.01	9.92
Aldehyde0	1.533	129.53	-0.621	1.07	9.53	10.65
Aldehyde1	1.535	128.31	-0.632	1.18	10.29	10.53
Aldehyde2	1.535	128.27	-0.636	1.35	11.48	10.34
Aldehyde30	1.576	130.47	-0.569	1.14	5.92	11.80
Aldehyde430	1.611	131.28	-0.526	1.35	5.63	12.10
Aldehyde50	1.570	129.99	-0.574	1.32	8.86	11.10
Aldehyde671	1.595	129.67	-0.567	1.39	13.78	9.70
Amide0	1.543	128.90	-0.615	1.10	9.75	10.62
Amide1	1.536	128.17	-0.639	1.35	11.73	10.28
Amide2	1.538	128.54	-0.630	1.34	13.37	9.77
Amide30	1.600	132.68	-0.512	0.95	2.87	12.52

Amide3V2	1.528	128.61	-0.639	1.63	18.96	8.39
Amide430	1.639	132.97	-0.483	1.69	12.81	10.29
Amide4V2	1.534	128.28	-0.636	1.35	10.30	10.70
Amide50	1.576	130.80	-0.570	1.42	11.89	10.29
Amide5V2	1.536	128.09	-0.645	1.08	11.85	9.97
Amide671	1.621	130.51	-0.538	1.35	2.09	13.15
Amide6V2	1.531	128.64	-0.636	0.82	2.15	12.60
Amide7V2	1.535	128.22	-0.637	1.32	12.00	10.16
Amide8V2	1.535	128.19	-0.638	1.35	10.18	10.74
Amine0	1.537	128.57	-0.639	1.69	19.18	8.40
Amine1	1.536	128.12	-0.645	1.48	15.19	9.37
Amine2	1.536	128.09	-0.642	1.47	13.98	9.72
Amine30	1.529	129.17	-0.635	1.07	8.37	11.00
Amine3V2	1.534	128.24	-0.640	1.43	14.99	9.39
Amine430	1.583	128.34	-0.587	1.18	6.81	11.57
Amine4V2	1.534	128.15	-0.639	1.15	10.10	10.56
Amine50	1.552	128.75	-0.619	1.58	14.39	9.71
Amine5V2	1.536	128.21	-0.637	1.23	10.68	10.47
Amine671	1.580	127.69	-0.594	1.49	9.76	11.00
Amine6V2	1.533	128.17	-0.640	1.45	13.23	9.93
Amine7V2	1.537	128.46	-0.637	1.50	15.73	9.23
Amine8V2	1.535	128.21	-0.638	1.48	16.41	9.01
Anhydride0	1.550	129.51	-0.602	1.19	8.63	11.04
Anhydride1	1.540	128.73	-0.621	1.40	10.15	10.80
Anhydride2	1.535	128.41	-0.633	1.47	13.93	9.74
Anhydride30	1.624	133.84	-0.481	1.65	13.12	10.16
Anhydride3V2	1.537	128.40	-0.632	1.30	11.36	10.34
Anhydride430	2.079	155.19	-0.107	1.02	-4.59	14.81
Anhydride4V2	1.536	128.25	-0.637	1.59	14.98	9.54
Anhydride50	1.580	130.58	-0.555	1.73	16.13	9.34
Anhydride5V2	1.535	128.20	-0.638	1.28	9.62	10.83
Anhydride671	1.604	130.28	-0.540	1.31	5.23	12.17
Anhydride7V2	1.535	128.24	-0.637	1.22	14.27	9.39
Anhydride8V2	1.534	128.14	-0.639	1.33	17.57	8.51
AzolidineBr1	1.566	129.53	-0.575	1.57	12.67	10.21
AzolidineBr2	1.554	128.76	-0.609	1.66	14.65	9.71
AzolidineC#N1	1.558	129.81	-0.577	1.50	12.18	10.29

AzolidineC#N2	1.557	129.18	-0.603	1.60	14.97	9.56
AzolidineCl1	1.562	129.90	-0.577	1.59	13.57	9.96
AzolidineCl2	1.553	128.92	-0.602	1.53	12.60	10.20
AzolidineF1	1.555	129.43	-0.589	1.62	14.91	9.60
AzolidineF2	1.554	128.96	-0.604	1.70	15.47	9.51
AzolidineI1	1.563	129.38	-0.577	1.64	14.12	9.85
AzolidineI2	1.551	128.79	-0.610	1.52	11.40	10.54
AzolidineN1	1.534	128.00	-0.635	1.44	11.96	10.29
AzolidineN2	1.549	128.64	-0.612	1.27	5.39	12.08
AzolidineNO2_1	1.581	131.11	-0.538	1.53	14.10	9.75
AzolidineNO2_2	1.563	129.63	-0.586	1.57	13.45	9.98
AzolidineO1	1.546	128.60	-0.614	1.52	11.98	10.37
AzolidineO2	1.552	128.91	-0.606	1.59	13.69	9.92
AzolidineS1	1.558	129.02	-0.597	1.44	10.56	10.71
AzolidineS2	1.552	128.95	-0.604	1.45	7.21	11.72
Br0	1.554	129.76	-0.594	1.54	14.10	9.76
Br1	1.541	128.78	-0.619	1.13	7.84	11.21
Br2	1.535	128.38	-0.633	1.33	12.48	10.03
Br3	1.607	132.67	-0.515	1.72	14.89	9.70
Br4	1.592	131.66	-0.535	1.36	10.56	10.64
Br5	4.685	179.79	0.000	3.10	37.63	4.30
Br6	1.593	129.55	-0.560	1.41	9.58	10.98
Cl0	1.554	130.03	-0.591	1.41	15.56	9.19
Cl1	1.537	128.24	-0.637	1.29	12.05	10.12
Cl2	1.537	128.73	-0.631	1.42	12.41	10.14
Cl3	1.600	132.59	-0.521	1.41	12.43	10.12
Cl4	1.586	131.53	-0.545	1.36	9.06	11.08
Cl5	5.586	179.93	0.000	3.05	37.61	4.26
Cl6	1.608	130.79	-0.540	1.43	7.97	11.47
Cyan0	1.547	129.97	-0.596	1.46	15.30	9.32
Cyan1	1.537	128.16	-0.635	1.34	10.73	10.57
Cyan2	1.536	128.21	-0.638	1.19	9.60	10.75
Cyan30	1.580	131.31	-0.551	1.51	11.66	10.46
Cyan3V2	1.536	128.29	-0.635	1.55	17.13	8.86
Cyan430	1.643	133.70	-0.466	1.65	9.64	11.20
Cyan4V2	1.532	128.18	-0.640	1.29	12.41	10.01
Cyan50	1.574	130.44	-0.573	1.57	13.57	9.95

Cyan5V2	1.534	128.14	-0.639	1.32	13.41	9.74
Cyan671	1.599	129.92	-0.549	1.33	0.72	13.54
Cyan6V2	1.533	128.24	-0.639	1.42	14.17	9.62
Cyan7V2	1.535	128.29	-0.637	1.43	11.60	10.40
Cyan8V2	1.534	128.07	-0.641	1.44	12.03	10.27
Ester0	1.538	130.49	-0.599	1.14	12.17	9.93
Ester1	1.539	128.67	-0.623	1.43	11.59	10.39
Ester2	1.536	128.34	-0.633	1.30	9.53	10.87
Ester30	1.600	131.66	-0.523	1.33	9.32	10.97
Ester3V2	1.535	128.20	-0.637	1.36	13.41	9.78
Ester430	4.589	179.82	0.000	2.96	37.75	4.13
Ester4V2	1.535	128.24	-0.637	1.49	14.38	9.63
Ester50	1.573	130.09	-0.578	1.62	12.93	10.19
Ester5V2	1.533	128.10	-0.639	1.34	15.86	9.04
Ester671	1.608	130.46	-0.543	1.55	6.90	11.91
Ester6V2	1.533	128.17	-0.639	1.38	10.73	10.60
Ester7V2	1.534	128.15	-0.639	1.33	11.84	10.23
Ester8V2	1.535	128.27	-0.637	1.51	13.46	9.91
Ether0	1.543	128.85	-0.611	1.21	10.56	10.49
Ether1	1.535	128.27	-0.633	1.24	11.58	10.21
Ether2	1.534	128.16	-0.638	1.16	9.95	10.61
Ether30	1.569	129.85	-0.586	1.78	14.20	9.97
Ether3V2	1.527	128.70	-0.640	1.33	11.72	10.26
Ether430	1.587	129.50	-0.558	1.68	12.74	10.30
Ether4V2	1.535	128.18	-0.638	1.47	16.02	9.11
Ether50	1.560	129.35	-0.598	1.43	8.52	11.31
Ether5V2	1.534	128.11	-0.640	1.22	8.09	11.23
Ether671	1.596	129.52	-0.567	1.67	10.13	11.07
Ether6V2	1.526	128.56	-0.642	1.34	13.98	9.59
Ether7V2	1.536	128.26	-0.637	1.31	6.95	11.66
Ether8V2	1.536	128.27	-0.637	1.37	11.85	10.26
F0	1.545	129.49	-0.602	1.44	14.76	9.46
F1	1.539	128.39	-0.635	1.23	12.58	9.90
F2	1.537	128.51	-0.629	1.16	8.98	10.91
F3	1.583	131.49	-0.533	1.68	13.95	9.94
F4	1.569	130.19	-0.571	1.50	12.61	10.16
F5	1.652	134.16	-0.448	1.66	7.44	11.87

F6	1.593	129.33	-0.565	1.53	9.11	11.23
Halid2V2	1.537	128.45	-0.629	1.37	12.30	10.12
Halid3V2	1.536	128.26	-0.636	1.42	13.47	9.83
Halid4V2	1.535	127.94	-0.646	1.28	12.66	9.93
Halid5V2	1.536	128.32	-0.637	1.36	13.71	9.70
Halid6V2	1.536	128.20	-0.638	1.43	13.24	9.90
Halid7V2	1.533	128.16	-0.640	1.34	11.79	10.24
Halid8V2	1.535	128.19	-0.638	1.51	12.35	10.25
I0	1.554	129.82	-0.601	1.34	12.64	9.99
I1	1.542	128.97	-0.617	1.12	9.96	10.57
I2	1.535	128.34	-0.634	1.25	10.47	10.55
I3	1.607	132.23	-0.522	1.53	9.91	11.00
I4	1.600	131.41	-0.537	1.15	8.05	11.18
I5	7.168	179.76	0.000	3.10	36.93	4.51
I6	1.599	130.06	-0.558	1.44	9.46	11.04
ImidazoleBr1	1.637	134.58	-0.442	1.91	15.64	9.67
ImidazoleC#N1	1.600	133.67	-0.464	1.68	14.91	9.65
ImidazoleC#N2	1.636	134.76	-0.442	1.91	15.55	9.69
ImidazoleCl1	1.626	134.00	-0.456	1.75	13.22	10.23
ImidazoleF1	1.630	134.41	-0.445	1.85	15.91	9.53
ImidazoleF2	1.600	133.45	-0.474	1.44	11.26	10.51
ImidazoleI1	1.639	134.55	-0.443	1.89	14.63	9.95
ImidazoleI2	1.600	133.44	-0.480	1.52	12.25	10.29
ImidazoleN1	1.572	131.01	-0.538	1.55	11.29	10.61
ImidazoleN2	1.582	131.64	-0.514	1.81	17.32	9.07
ImidazoleNO2_1	4.272	179.51	0.000	3.04	36.42	4.61
ImidazoleNO2_2	2.926	176.92	0.000	2.77	26.54	7.28
ImidazoleO1	1.600	133.37	-0.486	1.01	7.40	11.23
ImidazoleO2	1.586	132.25	-0.505	1.45	11.03	10.59
ImidazoleS1	1.600	132.49	-0.492	1.64	14.32	9.79
ImidazoleS2	1.600	136.26	-0.470	1.23	9.01	10.96
Ketone3V2	1.534	128.25	-0.637	1.36	10.76	10.57
Ketone4V2	1.532	128.16	-0.640	1.43	15.51	9.22
Ketone5V2	1.535	128.09	-0.646	1.26	13.42	9.69
Ketone6V2	1.535	128.24	-0.637	1.29	13.60	9.66
Ketone7V2	1.535	128.18	-0.638	1.43	12.15	10.23
Ketone8V2	1.535	128.25	-0.637	1.49	13.80	9.80

Nitro0	1.549	129.49	-0.600	1.44	12.99	9.99
Nitro1	1.536	129.84	-0.626	1.07	6.04	11.69
Nitro3	1.535	128.09	-0.643	1.34	10.70	10.57
Nitro30	1.574	131.04	-0.559	1.53	9.90	11.00
Nitro430	1.642	133.21	-0.473	1.50	5.70	12.22
Nitro4V2	1.536	128.35	-0.638	1.31	10.80	10.51
Nitro50	1.573	130.34	-0.574	1.55	14.48	9.65
Nitro5V2	1.534	128.21	-0.639	1.35	11.03	10.48
Nitro671	1.588	128.73	-0.576	1.31	9.15	11.00
Nitro6V2	1.532	128.18	-0.638	1.37	9.96	10.82
Nitro7V2	1.536	128.30	-0.636	1.64	15.47	9.45
Nitro8V2	1.535	128.23	-0.638	1.62	18.91	8.40
PiperidineBr1	1.567	129.15	-0.586	1.58	11.39	10.60
PiperidineBr2	1.556	128.56	-0.612	1.52	11.01	10.66
PiperidineBr3	1.554	128.36	-0.618	1.65	13.10	10.16
PiperidineC#N1	1.569	130.34	-0.572	1.69	12.96	10.25
PiperidineC#N2	1.557	128.88	-0.608	1.52	11.10	10.63
PiperidineC#N3	1.549	129.05	-0.601	1.67	13.89	9.95
PiperidineCl1	1.564	129.09	-0.593	1.59	11.86	10.48
PiperidineCl2	1.561	129.79	-0.588	1.65	12.75	10.27
PiperidineCl3	1.554	128.32	-0.619	1.63	14.45	9.74
PiperidineF1	1.562	129.89	-0.587	1.70	13.42	10.12
PiperidineF2	1.555	128.54	-0.611	1.52	11.40	10.55
PiperidineF3	1.558	129.46	-0.598	1.58	12.31	10.34
PiperidineI1	1.575	130.51	-0.567	1.64	11.83	10.53
PiperidineI2	1.560	129.61	-0.592	1.60	11.96	10.46
PiperidineI3	1.558	129.38	-0.597	1.70	13.84	10.00
PiperidineN1	1.545	128.87	-0.609	1.51	11.80	10.41
PiperidineN2	1.557	129.20	-0.603	1.59	12.69	10.23
PiperidineN3	1.549	128.96	-0.610	1.81	14.67	9.86
PiperidineNO2_1	1.575	130.31	-0.563	1.60	12.34	10.34
PiperidineNO2_2	1.563	129.31	-0.592	1.70	14.08	9.92
PiperidineNO2_3	1.557	128.76	-0.609	1.56	10.47	10.86
PiperidineO1	1.555	129.26	-0.612	1.49	12.11	10.31
PiperidineO2	1.547	129.48	-0.601	1.43	11.37	10.46
PiperidineO3	1.551	129.27	-0.601	1.47	12.57	10.15
PiperidineS1	1.562	128.63	-0.605	1.50	10.64	10.75

PiperidineS2	1.553	128.28	-0.618	1.70	13.20	10.18
PiperidineS3	1.555	128.88	-0.613	1.75	15.94	9.42
PyrazineBr1	1.565	128.79	-0.505	1.78	14.13	9.99
PyrazineBr2	1.541	130.36	-0.525	1.79	14.71	9.82
PyrazineC#N1	1.552	130.40	-0.504	1.73	14.77	9.74
PyrazineC#N2	1.547	130.57	-0.516	1.76	14.54	9.85
PyrazineCl1	1.556	131.00	-0.503	1.76	13.25	10.23
PyrazineCl2	1.542	130.34	-0.526	1.76	14.02	10.00
PyrazineF1	1.539	130.19	-0.524	1.82	18.45	8.74
PyrazineF2	1.543	130.56	-0.521	1.77	15.20	9.66
PyrazineN1	1.499	125.54	-0.618	1.63	13.78	9.94
PyrazineN2	1.535	129.17	-0.553	1.78	15.05	9.71
PyrazineNO2_1	1.579	131.85	-0.461	1.87	14.83	9.87
PyrazineNO2_2	1.558	131.54	-0.489	1.74	12.10	10.56
PyrazineO1	1.526	128.71	-0.550	1.77	15.09	9.69
PyrazineO2	1.541	129.82	-0.534	1.78	15.63	9.54
PyrazineS1	1.531	128.36	-0.546	1.64	12.62	10.30
PyrazineS2	1.539	129.76	-0.537	1.72	13.87	10.01
PyrazoleBr1	3.812	179.61	0.001	2.87	33.35	5.35
PyrazoleBr2	3.489	179.49	0.001	2.81	27.77	6.95
PyrazoleC#N1	3.611	179.36	0.000	2.82	28.09	6.86
PyrazoleC#N2	3.452	179.43	0.001	2.74	26.50	7.26
PyrazoleCl1	3.752	179.94	0.002	2.70	26.35	7.27
PyrazoleCl2	1.647	135.56	-0.437	1.82	15.00	9.77
PyrazoleF1	3.463	179.50	0.001	2.83	31.02	6.00
PyrazoleF2	4.262	179.64	0.000	2.98	35.63	4.78
PyrazoleI1	3.858	179.52	0.001	2.89	35.56	4.71
PyrazoleI2	3.067	179.26	0.000	2.93	30.16	6.36
PyrazoleN1	1.608	132.04	-0.525	1.67	14.01	9.91
PyrazoleN2	1.623	133.06	-0.492	1.58	13.00	10.13
PyrazoleNO2_1	5.059	179.94	0.000	2.93	34.41	5.09
PyrazoleNO2_2	3.825	179.91	0.000	2.79	30.30	6.18
PyrazoleO1	1.648	134.66	-0.457	1.58	13.62	9.94
PyrazoleO2	1.695	137.25	-0.402	1.78	14.64	9.83
PyrazoleS1	1.641	134.12	-0.467	1.59	13.96	9.85
PyrazoleS2	1.627	134.01	-0.468	1.66	13.51	10.05
PyridineBr1	1.544	126.83	-0.552	1.69	12.38	10.42

PyridineBr2	1.524	128.57	-0.570	1.77	14.09	9.99
PyridineBr3	1.522	128.42	-0.575	1.83	17.41	9.06
PyridineC#N1	1.530	128.46	-0.553	1.69	13.46	10.10
PyridineC#N2	1.526	128.81	-0.563	1.75	14.08	9.98
PyridineC#N3	1.526	128.66	-0.567	1.75	13.93	10.01
PyridineCl1	1.536	126.89	-0.561	1.65	12.44	10.37
PyridineCl2	1.524	128.50	-0.570	1.76	14.80	9.77
PyridineCl3	1.519	128.19	-0.581	1.71	13.90	9.99
PyridineF1	1.520	128.43	-0.570	1.77	14.30	9.93
PyridineF2	1.527	128.72	-0.565	1.80	15.53	9.59
PyridineF3	1.517	128.09	-0.584	1.74	15.37	9.58
PyridineI1	1.540	127.13	-0.556	1.72	12.61	10.38
PyridineI2	1.522	128.46	-0.574	1.77	14.11	9.98
PyridineI3	1.523	128.43	-0.574	1.83	17.07	9.17
PyridineN1	1.495	125.33	-0.636	1.74	13.38	10.18
PyridineN2	1.520	127.82	-0.590	1.65	13.24	10.13
PyridineN3	1.488	126.06	-0.650	1.80	13.47	10.21
PyridineNO2_1	1.549	130.58	-0.510	1.82	13.55	10.20
PyridineNO2_2	1.537	129.70	-0.537	1.82	14.81	9.83
PyridineNO2_3	1.537	129.55	-0.541	1.86	17.25	9.14
PyridineO1	1.506	126.61	-0.594	1.77	13.98	10.02
PyridineO2	1.522	128.19	-0.580	1.72	14.60	9.79
PyridineO3	1.507	127.37	-0.606	1.68	14.36	9.82
PyridineS1	1.521	126.39	-0.589	1.77	15.24	9.64
PyridineS2	1.522	128.28	-0.579	1.66	12.84	10.25
PyridineS3	1.509	127.10	-0.605	1.60	12.72	10.23
PyrimidineBr1	1.547	127.57	-0.530	1.78	15.83	9.48
PyrimidineBr2	1.536	128.91	-0.532	1.85	14.82	9.85
PyrimidineBr3	1.526	129.40	-0.547	1.78	15.76	9.50
PyrimidineC#N1	1.535	129.35	-0.526	1.62	13.62	9.98
PyrimidineC#N2	1.531	129.48	-0.531	1.72	13.57	10.10
PyrimidineC#N3	1.529	129.65	-0.540	1.76	15.66	9.51
PyrimidineCl1	1.535	127.55	-0.543	1.70	13.90	9.98
PyrimidineCl2	1.538	130.01	-0.529	1.87	15.63	9.63
PyrimidineCl3	1.526	129.33	-0.549	1.76	15.87	9.45
PyrimidineF1	1.523	129.49	-0.543	1.79	17.83	8.90
PyrimidineF2	1.522	129.15	-0.549	1.73	12.94	10.29

PyrimidineF3	1.529	129.52	-0.543	1.77	16.73	9.21
PyrimidineI1	1.540	127.94	-0.537	1.80	14.91	9.78
PyrimidineI2	1.540	128.84	-0.531	1.77	13.40	10.19
PyrimidineI3	1.527	129.28	-0.549	1.80	15.99	9.45
PyrimidineN1	1.496	125.45	-0.619	1.58	12.47	10.29
PyrimidineN2	1.492	125.24	-0.626	1.66	14.60	9.73
PyrimidineN3	1.520	128.58	-0.568	1.91	16.28	9.47
PyrimidineNO2_1	1.555	131.26	-0.486	2.06	17.65	9.22
PyrimidineNO2_2	1.547	130.77	-0.498	1.64	10.63	10.90
PyrimidineNO2_3	1.542	130.47	-0.513	1.76	14.71	9.79
PyrimidineO1	1.514	128.09	-0.565	1.72	14.34	9.86
PyrimidineO2	1.504	127.27	-0.578	1.78	13.70	10.12
PyrimidineO3	1.523	128.99	-0.558	1.71	14.02	9.95
PyrimidineS1	1.514	127.69	-0.567	1.62	12.74	10.24
PyrimidineS2	1.516	127.32	-0.578	1.68	15.01	9.62
PyrimidineS3	1.522	128.96	-0.558	1.73	13.62	10.09
PyrroleBr1	1.600	132.13	-0.507	1.41	10.65	10.66
PyrroleBr2	1.600	131.82	-0.509	1.46	11.35	10.50
PyrroleC#N1	1.600	132.53	-0.501	1.32	10.26	10.68
PyrroleC#N2	1.601	132.30	-0.498	1.34	10.53	10.62
PyrroleCl1	1.593	131.56	-0.517	1.69	13.97	9.94
PyrroleCl2	1.592	131.50	-0.520	1.67	13.52	10.06
PyrroleF1	1.594	131.82	-0.510	1.67	14.26	9.84
PyrroleF2	1.600	131.85	-0.510	1.28	9.89	10.75
PyrroleI1	1.600	131.70	-0.509	1.45	11.37	10.48
PyrroleI2	1.594	131.56	-0.516	1.74	14.47	9.85
PyrroleN1	1.571	130.61	-0.549	1.57	13.90	9.85
PyrroleN2	1.563	129.63	-0.574	1.46	11.27	10.52
PyrroleNO2_1	1.644	135.09	-0.436	1.97	17.22	9.25
PyrroleNO2_2	2.002	154.32	-0.125	2.19	19.16	8.89
PyrroleO1	1.577	131.35	-0.532	1.67	15.05	9.61
PyrroleO2	1.579	130.60	-0.545	1.56	14.49	9.66
PyrroleS1	1.591	131.57	-0.522	1.77	16.01	9.41
PyrroleS2	1.580	130.66	-0.543	1.45	10.90	10.62
ThiazoleBr1	1.507	127.08	-0.577	1.70	12.98	10.25
ThiazoleBr2	1.497	128.09	-0.587	1.74	13.27	10.20
ThiazoleC#N1	1.500	128.05	-0.576	1.80	15.81	9.51

ThiazoleC#N2	1.498	128.13	-0.581	1.74	13.55	10.12
ThiazoleC#N3	1.501	128.23	-0.571	1.73	13.98	9.98
ThiazoleCl1	1.501	126.79	-0.587	1.70	13.24	10.17
ThiazoleCl2	1.495	128.09	-0.588	1.77	16.47	9.28
ThiazoleCl3	1.501	126.97	-0.585	1.72	14.60	9.78
ThiazoleF1	1.495	127.85	-0.587	1.74	13.90	10.01
ThiazoleF2	1.498	128.09	-0.583	1.72	13.95	9.99
ThiazoleF3	1.493	127.97	-0.584	1.72	13.60	10.09
ThiazoleI1	1.503	127.09	-0.583	1.74	14.04	9.98
ThiazoleI2	1.494	127.83	-0.593	1.75	13.62	10.11
ThiazoleI3	1.509	127.07	-0.577	1.78	17.70	8.93
ThiazoleN1	1.471	125.42	-0.648	1.65	13.57	10.03
ThiazoleN2	1.493	127.47	-0.603	1.68	13.43	10.09
ThiazoleN3	1.478	125.80	-0.631	1.66	13.84	9.96
ThiazoleNO2_1	1.505	129.27	-0.554	1.88	16.01	9.53
ThiazoleNO2_2	1.503	129.27	-0.557	1.81	14.99	9.76
ThiazoleNO2_3	1.512	129.17	-0.542	1.81	14.49	9.91
ThiazoleO1	1.488	126.99	-0.604	1.62	13.45	10.03
ThiazoleO2	1.495	127.76	-0.592	1.71	13.80	10.02
ThiazoleO3	1.487	127.06	-0.601	1.58	13.05	10.11
ThiazoleS1	1.491	126.83	-0.600	1.55	11.61	10.51
ThiazoleS2	1.494	127.76	-0.596	1.68	12.56	10.36
ThiazoleS3	1.494	127.00	-0.594	1.58	11.73	10.51
Tio0	1.538	128.48	-0.627	1.28	12.99	9.83
Tio1	1.536	128.28	-0.635	1.25	11.20	10.34
Tio2	1.535	128.25	-0.637	1.62	17.95	8.69
Tio30	1.568	129.91	-0.584	1.65	13.87	9.94
Tio3V2	1.534	128.24	-0.638	1.51	14.02	9.76
Tio430	1.600	129.53	-0.562	1.96	11.73	10.89
Tio4V2	1.534	128.15	-0.640	1.38	14.04	9.61
Tio50	1.569	129.77	-0.587	1.28	6.81	11.67
Tio5V2	1.535	128.14	-0.639	1.29	11.47	10.29
Tio671	1.590	129.03	-0.578	1.37	6.95	11.72
Tio6V2	1.535	128.20	-0.638	1.49	13.63	9.85
Tio7V2	1.535	128.17	-0.639	1.30	8.58	11.16
Tio8V2	1.535	128.23	-0.638	1.34	10.39	10.67
TriazoleC#N1	3.930	179.94	0.001	2.84	30.85	6.06

TriazoleC#N2	3.295	178.48	0.001	2.12	20.09	8.56
TriazoleCl1	3.841	179.95	0.001	2.74	27.26	7.03
TriazoleCl2	3.404	179.81	0.001	2.05	17.71	9.19
TriazoleF1	3.872	179.87	0.001	2.80	31.07	5.96
TriazoleF2	3.769	179.94	0.001	2.27	24.54	7.37
TriazoleI2	3.243	178.81	0.001	2.09	17.83	9.20
TriazoleN1	3.362	179.28	0.001	2.84	29.82	6.37
TriazoleN2	3.768	179.93	0.001	2.34	24.64	7.41
TriazoleNO2_1	3.824	179.96	0.000	2.88	31.58	5.89
TriazoleNO2_2	3.088	177.61	0.000	2.28	25.22	7.18
TriazoleO1	3.817	179.43	0.001	2.81	31.47	5.85
TriazoleO2	3.760	179.58	0.003	2.88	31.63	5.87
TriazoleS1	3.826	179.88	0.001	2.82	32.08	5.67
TriazoleS2	3.316	178.79	0.001	2.28	26.81	6.71

Table S4: Parameters calculated using the GFN0-xTB method.

Name	R (Å)	θ (°)	ΔQ	ΔH (kcal/mol)	ΔS (cal/molK)	ΔG (kcal/mol)
Acid0	1.565	139.62	-0.524	-1.02	-38.34	10.41
Acid1	1.567	139.62	-0.552	-1.28	-40.99	10.94
Acid2	1.565	139.40	-0.581	-1.71	-44.64	11.60
Acid30	1.572	139.67	-0.582	-0.61	-37.47	10.56
Acid3V2	6.259	122.99	-0.858	-1.79	-45.43	11.75
Acid430	1.562	138.37	-0.654	-0.62	-38.87	10.97
Acid4V2	6.815	122.98	-0.866	-1.80	-45.77	11.85
Acid50	1.578	140.89	-0.596	-1.19	-41.62	11.22
Acid671	1.570	137.49	-0.560	-0.47	-37.83	10.81
Acid6V2	6.344	122.66	-0.875	-1.92	-47.39	12.21

Acid7V2	9.353	123.08	-0.883	-1.72	-46.50	12.15
Acid8V2	12.470	123.17	-0.891	-1.73	-45.91	11.96
AcidBr1	4.818	178.73	-0.723	-1.72	-45.09	11.73
AcidBr2	5.147	179.36	-0.800	-1.73	-45.37	11.80
AcidBr3	2.759	173.16	-0.499	-1.21	-42.75	11.54
AcidBr4	4.559	179.74	-0.615	-1.73	-46.93	12.27
AcidBr5	4.378	179.70	-0.463	-0.68	-42.03	11.86
AcidBr6	2.791	172.43	-0.541	-0.83	-41.17	11.44
AcidCl0	5.039	179.16	-0.616	-1.29	-40.57	10.80
AcidCl1	4.784	178.92	-0.653	-1.68	-44.07	11.46
AcidCl2	5.127	179.69	-0.725	-1.72	-44.91	11.67
AcidCl3	2.789	172.05	-0.394	-1.17	-42.34	11.46
AcidCl4	2.929	172.01	-0.487	-1.21	-42.97	11.60
AcidCl5	2.736	171.88	-0.286	-0.60	-39.44	11.16
AcidCl6	2.871	171.89	-0.494	-0.69	-38.08	10.66
AcidF0	1.576	140.28	-0.528	-1.27	-40.21	10.72
AcidF1	1.584	141.06	-0.552	-0.87	-37.69	10.37
AcidF2	1.575	140.35	-0.565	-1.72	-44.42	11.53
AcidF3	1.564	139.07	-0.489	-1.07	-40.69	11.06
AcidF4	1.581	141.35	-0.488	-1.21	-42.18	11.37
AcidF5	1.548	139.00	-0.436	-0.67	-39.48	11.10
AcidF6	1.556	136.99	-0.512	-0.84	-41.77	11.62

AcidI0	5.047	179.02	-0.765	-1.33	-41.99	11.19
AcidI1	4.915	178.61	-0.757	-1.23	-41.27	11.08
AcidI2	4.746	179.41	-0.814	-1.17	-40.77	10.98
AcidI3	5.015	179.56	-0.573	-0.67	-38.95	10.94
AcidI4	4.534	179.64	-0.639	-0.54	-36.04	10.21
AcidI5	4.529	178.90	-0.447	-0.61	-39.69	11.22
AcidI6	2.796	173.73	-0.581	-0.73	-41.31	11.58
Acid_Sulf0	1.571	139.82	-0.432	-1.74	-44.64	11.57
Acid_Sulf1	1.572	140.18	-0.464	-1.70	-44.83	11.66
Acid_Sulf2	1.581	140.78	-0.482	-1.73	-45.21	11.75
Acid_Sulf30	1.551	137.43	-0.384	-1.69	-46.69	12.23
Acid_Sulf430	1.543	136.64	-0.348	-0.87	-42.12	11.69
Acid_Sulf50	1.583	139.73	-0.410	-1.21	-42.89	11.58
Acid_Sulf671	1.548	136.73	-0.445	-0.96	-43.67	12.07
Acid_Tio0	1.563	139.44	-0.565	-1.13	-39.53	10.65
Acid_Tio1	1.579	140.75	-0.584	-1.83	-45.44	11.71
Acid_Tio2	1.565	139.37	-0.613	-1.76	-45.58	11.83
Acid_Tio30	1.568	139.26	-0.557	-1.09	-41.63	11.32
Acid_Tio430	1.563	138.63	-0.567	-0.65	-39.75	11.20
Acid_Tio50	1.601	141.30	-0.569	-1.11	-42.17	11.46
Acid_Tio671	1.549	136.89	-0.580	-0.23	-36.66	10.70
Alcohol0	1.571	140.13	-0.703	-1.17	-38.71	10.37

Alcohol1	1.574	140.24	-0.682	-1.80	-44.17	11.37
Alcohol2	1.570	139.90	-0.691	-1.78	-44.43	11.47
Alcohol30	1.562	138.44	-0.783	-1.70	-45.74	11.94
Alcohol430	1.569	138.44	-0.801	-0.88	-41.74	11.57
Alcohol50	1.577	139.57	-0.747	-1.18	-42.31	11.43
Alcohol671	1.557	136.62	-0.802	-0.94	-41.52	11.44
Aldehyde0	1.566	139.56	-0.583	-1.29	-39.52	10.49
Aldehyde1	1.562	139.28	-0.606	-1.40	-41.34	10.93
Aldehyde2	1.572	140.11	-0.612	-1.74	-44.16	11.42
Aldehyde30	1.564	139.19	-0.572	-1.14	-40.92	11.06
Aldehyde430	1.548	138.97	-0.595	-0.73	-39.66	11.09
Aldehyde50	1.567	138.85	-0.616	-1.19	-41.89	11.30
Aldehyde671	1.555	137.28	-0.643	-0.76	-40.83	11.42
Amide0	1.570	139.83	-0.636	-1.74	-43.69	11.28
Amide1	1.565	139.26	-0.591	-1.84	-44.91	11.55
Amide2	1.557	139.80	-0.594	-1.76	-44.46	11.50
Amide30	1.572	139.22	-0.623	-1.15	-41.41	11.20
Amide430	1.567	139.03	-0.574	-0.84	-41.51	11.53
Amide50	1.572	138.70	-0.632	-1.20	-42.35	11.42
Amide671	1.593	139.09	-0.641	-0.66	-39.97	11.26
Amine0	1.556	139.27	-0.715	-1.82	-43.45	11.13
Amine1	1.565	139.28	-0.705	-1.96	-45.25	11.53

Amine2	1.557	138.95	-0.705	-1.88	-45.08	11.56
Amine30	1.570	139.80	-0.710	-1.92	-45.80	11.74
Amine430	1.561	137.64	-0.766	-1.07	-43.44	11.88
Amine50	1.576	138.46	-0.800	-1.79	-46.91	12.19
Amine671	1.554	136.33	-0.788	-0.89	-41.81	11.58
AzolidineBr1	1.576	139.63	-0.656	-1.19	-41.63	11.23
AzolidineBr2	1.560	138.48	-0.672	-0.70	-37.44	10.47
AzolidineC#N1	1.571	139.51	-0.604	-1.14	-40.71	11.00
AzolidineC#N2	1.570	140.22	-0.593	-1.94	-46.92	12.05
AzolidineCl1	1.571	140.44	-0.728	-1.19	-41.27	11.11
AzolidineCl2	1.564	138.76	-0.698	-1.27	-41.89	11.21
AzolidineF1	1.583	140.85	-0.780	-1.71	-45.20	11.77
AzolidineF2	1.559	139.15	-0.738	-1.70	-44.72	11.63
AzolidineI1	1.590	140.74	-0.727	-1.19	-41.78	11.27
AzolidineI2	1.565	139.52	-0.759	-1.17	-41.27	11.14
AzolidineN1	1.566	139.79	-0.776	-1.18	-41.00	11.04
AzolidineN2	1.564	139.04	-0.826	-1.29	-41.74	11.15
AzolidineNO2_1	1.605	141.31	-0.516	-1.70	-45.96	12.00
AzolidineNO2_2	1.569	139.92	-0.567	-1.67	-45.08	11.77
AzolidineO1	1.578	140.57	-0.790	-1.69	-44.79	11.66
AzolidineO2	1.561	139.89	-0.746	-1.21	-40.83	10.96
AzolidineS1	1.567	139.75	-0.838	-1.67	-45.74	11.96

AzolidineS2	1.563	139.93	-0.788	-1.73	-45.40	11.81
Br0	1.595	141.48	-0.673	-1.76	-42.73	10.98
Br1	1.565	139.50	-0.663	-1.27	-40.36	10.76
Br2	1.573	140.15	-0.650	-1.85	-45.39	11.69
Br3	1.566	139.29	-0.675	-0.73	-38.82	10.84
Br4	1.555	137.94	-0.665	-1.29	-42.99	11.52
Br5	2.687	176.30	-0.818	-0.73	-40.59	11.37
Br6	1.546	136.45	-0.667	-0.76	-40.84	11.42
Cl0	1.614	142.82	-0.665	-1.80	-42.67	10.92
Cl1	1.568	139.76	-0.665	-2.02	-46.24	11.77
Cl2	1.574	140.27	-0.657	-1.84	-45.02	11.58
Cl3	1.610	142.20	-0.643	-0.80	-38.14	10.57
Cl4	1.606	141.60	-0.689	-1.26	-42.11	11.29
Cl5	2.752	175.12	-0.762	-0.31	-34.91	10.10
Cl6	1.572	138.59	-0.670	-1.33	-45.05	12.10
Cyan0	1.582	141.02	-0.470	-1.61	-41.81	10.86
Cyan1	1.570	139.99	-0.512	-1.55	-42.14	11.01
Cyan2	1.574	140.30	-0.547	-1.74	-44.27	11.46
Cyan30	1.566	140.55	-0.364	-0.97	-39.90	10.93
Cyan430	1.563	139.82	-0.307	-0.77	-37.77	10.49
Cyan50	1.573	139.94	-0.433	-0.85	-38.35	10.58
Cyan671	1.546	137.13	-0.423	0.23	-33.29	10.15

Ester0	1.568	139.63	-0.587	-1.79	-44.65	11.52
Ester1	1.574	140.21	-0.597	-1.71	-44.57	11.58
Ester2	1.571	139.79	-0.616	-1.74	-44.90	11.65
Ester30	1.584	139.79	-0.583	-1.62	-45.43	11.93
Ester3V2	5.884	127.62	-0.615	-1.84	-45.81	11.82
Ester430	1.571	138.61	-0.601	-0.79	-41.12	11.47
Ester4V2	6.787	127.65	-0.616	-1.82	-46.04	11.91
Ester50	1.561	138.32	-0.581	-1.12	-41.75	11.32
Ester5V2	8.250	127.79	-0.629	-1.83	-45.90	11.86
Ester671	1.562	137.45	-0.606	-0.68	-40.62	11.43
Ester6V2	9.961	127.76	-0.627	-1.75	-46.09	11.99
Ester7V2	10.322	127.77	-0.630	-1.88	-46.45	11.97
Ester8V2	12.463	127.82	-0.636	-1.78	-46.43	12.06
Ether0	1.587	141.06	-0.706	-1.96	-44.72	11.37
Ether1	1.569	139.83	-0.711	-1.89	-45.08	11.55
Ether2	1.569	139.86	-0.702	-2.11	-47.92	12.17
Ether30	1.563	139.08	-0.665	-1.15	-40.95	11.06
Ether430	1.580	139.36	-0.756	-1.19	-43.17	11.68
Ether50	1.571	138.68	-0.747	-1.73	-46.37	12.09
Ether671	1.564	137.80	-0.768	-0.88	-41.35	11.45
F0	4.687	179.73	-0.937	-1.40	-38.90	10.20
F1	1.567	139.68	-0.697	-1.36	-40.12	10.60

F2	1.573	140.01	-0.702	-1.40	-41.68	11.03
F3	4.496	179.78	-0.868	-1.15	-40.04	10.79
F4	1.583	140.57	-0.724	-1.30	-43.02	11.52
F5	5.062	179.77	-0.839	-0.82	-40.34	11.21
F6	1.554	137.68	-0.737	-0.91	-42.52	11.77
I0	4.123	179.71	-0.892	-1.85	-43.66	11.17
I1	1.567	139.68	-0.699	-1.35	-40.78	10.81
I2	1.573	140.19	-0.688	-1.73	-44.62	11.57
I3	1.589	141.20	-0.702	-1.34	-43.82	11.72
I4	1.594	140.89	-0.771	-1.27	-43.44	11.68
I5	1.596	140.57	-0.761	-0.71	-39.73	11.14
I6	1.558	137.49	-0.728	-0.75	-41.44	11.61
ImidazoleBr1	5.094	179.72	-0.838	-1.36	-42.63	11.35
ImidazoleBr2	3.829	179.65	-0.750	-1.00	-40.15	10.97
ImidazoleC#N1	5.041	179.55	-0.674	-1.30	-42.09	11.25
ImidazoleC#N2	3.661	178.16	-0.643	-1.09	-40.28	10.92
ImidazoleCl1	5.095	179.56	-0.816	-1.36	-42.35	11.27
ImidazoleCl2	3.682	179.92	-0.829	-1.13	-40.74	11.01
ImidazoleF1	5.114	179.83	-0.833	-1.36	-42.08	11.19
ImidazoleF2	3.673	179.89	-0.853	-1.05	-39.97	10.87
ImidazoleI1	5.087	179.53	-0.866	-1.36	-42.67	11.36
ImidazoleI2	3.904	179.56	-0.784	-1.16	-41.31	11.16

ImidazoleN1	3.590	179.76	-0.955	-1.29	-41.63	11.12
ImidazoleN2	3.582	179.96	-0.969	-0.71	-37.85	10.57
ImidazoleNO2_1	3.689	178.51	-0.663	-1.25	-42.26	11.35
ImidazoleNO2_2	4.069	179.10	-0.587	-0.54	-36.54	10.35
ImidazoleO1	3.611	179.39	-0.927	-1.09	-40.32	10.93
ImidazoleO2	3.585	179.70	-0.936	-0.67	-37.28	10.45
ImidazoleS1	3.595	179.30	-0.957	-1.02	-39.47	10.75
ImidazoleS2	2.930	174.01	-0.845	-0.81	-38.73	10.74
Nitro0	1.577	140.58	-0.473	-1.78	-44.25	11.42
Nitro1	1.577	140.55	-0.504	-1.22	-40.62	10.89
Nitro3	1.562	139.59	-0.545	-2.00	-47.47	12.15
Nitro30	1.569	139.32	-0.433	-1.27	-43.12	11.58
Nitro430	1.543	136.82	-0.451	-1.07	-44.61	12.23
Nitro50	1.570	139.31	-0.459	-1.34	-44.17	11.83
Nitro671	1.542	135.64	-0.430	-0.98	-42.65	11.73
PiperidineBr1	1.559	138.07	-0.719	-1.52	-44.04	11.61
PiperidineBr2	1.559	138.42	-0.752	-1.56	-44.32	11.65
PiperidineBr3	1.559	138.39	-0.745	-1.55	-44.34	11.67
PiperidineC#N1	1.572	140.31	-0.612	-1.51	-43.68	11.51
PiperidineC#N2	1.563	138.94	-0.619	-1.54	-43.86	11.54
PiperidineC#N3	1.583	140.52	-0.623	-1.81	-45.84	11.85
PiperidineCl1	1.566	138.78	-0.731	-1.55	-44.17	11.62

PiperidineCl2	1.579	140.17	-0.743	-1.11	-40.43	10.94
PiperidineCl3	1.564	138.71	-0.752	-1.60	-44.39	11.64
PiperidineF1	1.569	139.94	-0.758	-1.57	-44.01	11.55
PiperidineF2	1.561	138.70	-0.768	-1.58	-43.99	11.54
PiperidineF3	1.582	140.18	-0.776	-1.16	-40.95	11.05
PiperidineI1	1.565	139.65	-0.757	-1.59	-45.15	11.87
PiperidineI2	1.576	139.89	-0.772	-1.06	-40.24	10.94
PiperidineI3	1.577	139.77	-0.780	-1.58	-44.59	11.72
PiperidineN1	1.600	140.22	-0.842	-1.20	-41.21	11.08
PiperidineN2	1.571	139.41	-0.765	-1.66	-44.34	11.56
PiperidineN3	1.577	139.82	-0.772	-1.58	-43.87	11.50
PiperidineNO2_1	1.565	138.24	-0.572	-1.47	-43.79	11.58
PiperidineNO2_2	1.559	138.81	-0.600	-1.57	-44.38	11.66
PiperidineNO2_3	1.556	138.25	-0.612	-1.09	-40.61	11.01
PiperidineO1	1.598	140.80	-0.841	-1.26	-42.17	11.31
PiperidineO2	1.562	139.63	-0.750	-1.62	-44.21	11.56
PiperidineO3	1.583	140.17	-0.757	-1.57	-43.86	11.51
PiperidineS1	1.570	138.06	-0.806	-1.33	-42.78	11.43
PiperidineS2	1.563	138.86	-0.788	-1.56	-44.12	11.59
PiperidineS3	1.561	138.55	-0.788	-1.54	-43.94	11.56
PyrazineBr1	1.492	132.46	-0.442	-0.88	-39.75	10.97
PyrazineBr2	1.488	135.14	-0.520	-0.81	-38.76	10.74

PyrazineC#N1	1.487	136.14	-0.375	-0.95	-39.83	10.93
PyrazineC#N2	1.490	135.58	-0.382	-0.80	-38.63	10.72
PyrazineCl1	1.498	133.60	-0.477	-0.86	-39.30	10.86
PyrazineCl2	1.490	135.34	-0.511	-0.83	-38.82	10.75
PyrazineF1	1.490	134.08	-0.487	-0.84	-38.95	10.77
PyrazineF2	1.488	135.14	-0.523	-0.86	-39.03	10.78
PyrazineI1	1.500	132.47	-0.485	-0.96	-40.68	11.17
PyrazineI2	1.490	135.25	-0.547	-0.78	-38.54	10.71
PyrazineN1	1.484	133.83	-0.617	-0.89	-39.11	10.77
PyrazineN2	1.488	134.90	-0.560	-0.83	-38.64	10.69
PyrazineNO2_1	1.490	134.23	-0.304	-0.67	-38.00	10.66
PyrazineNO2_2	1.485	135.10	-0.357	-0.81	-39.20	10.88
PyrazineO1	1.479	132.52	-0.467	-0.69	-38.06	10.66
PyrazineO2	1.489	135.06	-0.535	-0.78	-38.57	10.72
PyrazineS1	1.485	132.42	-0.548	-0.69	-37.92	10.61
PyrazineS2	1.489	135.06	-0.582	-0.78	-38.47	10.69
PyrazoleBr1	6.030	179.58	-0.817	-0.53	-36.16	10.25
PyrazoleBr2	4.412	179.70	-0.728	-0.89	-38.98	10.73
PyrazoleC#N1	4.935	178.89	-0.672	-0.53	-35.88	10.17
PyrazoleC#N2	3.969	178.56	-0.641	-1.12	-40.60	10.98
PyrazoleCl1	5.902	179.57	-0.816	-0.59	-36.27	10.22
PyrazoleCl2	4.456	179.68	-0.772	-0.94	-39.03	10.70

PyrazoleF1	5.922	179.50	-0.841	-0.65	-36.42	10.21
PyrazoleF2	4.663	179.77	-0.803	-1.05	-39.84	10.82
PyrazoleI1	6.078	179.55	-0.857	-1.09	-40.65	11.04
PyrazoleI2	4.370	179.50	-0.777	-1.49	-44.12	11.67
PyrazoleN1	5.423	179.99	-0.879	-0.73	-36.38	10.11
PyrazoleN2	3.629	179.62	-0.932	-1.00	-37.77	10.26
PyrazoleNO2_1	4.444	179.81	-0.623	-0.45	-35.81	10.23
PyrazoleNO2_2	4.178	179.11	-0.600	-0.84	-38.78	10.72
PyrazoleO1	5.989	179.68	-0.838	-1.08	-39.66	10.74
PyrazoleO2	5.142	179.76	-0.919	-0.98	-38.74	10.57
PyrazoleS1	3.651	179.38	-0.933	-1.08	-40.06	10.86
PyrazoleS2	4.957	179.69	-0.917	-0.93	-39.00	10.70
PyridineBr1	1.501	133.04	-0.539	-0.74	-38.71	10.80
PyridineBr2	1.491	135.12	-0.587	-0.66	-37.73	10.59
PyridineBr3	1.487	135.11	-0.583	-0.65	-36.79	10.32
PyridineC#N1	1.490	136.20	-0.458	-0.61	-37.16	10.47
PyridineC#N2	1.494	135.63	-0.458	-0.91	-39.67	10.92
PyridineC#N3	1.493	135.65	-0.460	-0.70	-36.83	10.28
PyridineCl1	1.508	133.88	-0.572	-0.73	-38.26	10.68
PyridineCl2	1.493	135.37	-0.591	-0.67	-37.80	10.60
PyridineCl3	1.490	135.25	-0.593	-0.68	-36.62	10.24
PyridineF1	1.496	134.27	-0.586	-0.62	-37.26	10.49

PyridineF2	1.490	135.12	-0.614	-0.70	-37.86	10.59
PyridineF3	1.490	135.20	-0.618	-0.72	-36.93	10.29
PyridineI1	1.506	133.45	-0.589	-1.33	-43.61	11.68
PyridineI2	1.492	135.22	-0.620	-0.65	-37.73	10.60
PyridineI3	1.489	135.20	-0.621	-1.20	-41.30	11.11
PyridineN1	1.492	133.79	-0.703	-0.61	-37.01	10.43
PyridineN2	1.491	134.93	-0.634	-0.67	-37.69	10.56
PyridineN3	1.482	134.71	-0.634	-0.62	-37.27	10.49
PyridineNO2_1	1.498	134.38	-0.386	-1.06	-41.18	11.22
PyridineNO2_2	1.489	135.17	-0.434	-0.67	-38.31	10.75
PyridineNO2_3	1.491	135.46	-0.442	-0.69	-37.09	10.36
PyridineO1	1.484	134.46	-0.689	-0.87	-38.75	10.68
PyridineO2	1.492	135.09	-0.612	-0.71	-37.96	10.61
PyridineO3	1.481	134.66	-0.613	-0.69	-37.98	10.63
PyridineS1	1.502	133.12	-0.713	-0.73	-38.33	10.70
PyridineS2	1.492	135.12	-0.651	-0.68	-37.76	10.58
PyridineS3	1.484	134.78	-0.650	-0.67	-37.91	10.64
PyrimidineBr1	1.505	133.49	-0.489	-1.22	-41.36	11.11
PyrimidineBr2	1.498	133.30	-0.445	-1.18	-42.10	11.38
PyrimidineBr3	1.490	135.42	-0.482	-0.68	-36.36	10.16
PyrimidineC#N1	1.491	136.78	-0.387	-0.59	-35.75	10.06
PyrimidineC#N2	1.492	136.65	-0.375	-1.09	-40.89	11.10

PyrimidineC#N3	1.496	136.09	-0.370	-0.92	-38.25	10.49
PyrimidineCl1	1.510	134.58	-0.501	-0.63	-36.44	10.23
PyrimidineCl2	1.508	134.24	-0.482	-1.17	-41.79	11.29
PyrimidineCl3	1.494	135.76	-0.496	-0.70	-36.52	10.19
PyrimidineF1	1.499	134.95	-0.496	-0.66	-36.29	10.16
PyrimidineF2	1.498	134.72	-0.488	-1.15	-41.36	11.18
PyrimidineF3	1.492	135.57	-0.514	-0.72	-36.46	10.15
PyrimidineI1	1.512	133.39	-0.528	-1.23	-41.58	11.17
PyrimidineI2	1.506	133.54	-0.493	-1.24	-42.87	11.54
PyrimidineI3	1.492	135.53	-0.516	-0.67	-36.37	10.18
PyrimidineN1	1.495	134.71	-0.627	-0.86	-39.11	10.80
PyrimidineN2	1.495	134.33	-0.621	-1.20	-41.78	11.26
PyrimidineN3	1.493	135.35	-0.548	-0.67	-37.49	10.51
PyrimidineNO2_1	1.498	134.58	-0.316	-0.58	-36.30	10.24
PyrimidineNO2_2	1.498	134.55	-0.306	-1.05	-41.05	11.19
PyrimidineNO2_3	1.490	135.58	-0.345	-0.70	-37.00	10.33
PyrimidineO1	1.489	133.43	-0.485	-0.65	-37.37	10.49
PyrimidineO2	1.488	135.04	-0.601	-1.43	-43.21	11.46
PyrimidineO3	1.493	135.51	-0.522	-0.72	-37.83	10.56
PyrimidineS1	1.495	133.26	-0.581	-1.16	-41.83	11.31
PyrimidineS2	1.503	133.66	-0.632	-1.17	-41.86	11.31
PyrimidineS3	1.493	135.51	-0.564	-0.70	-37.72	10.55

PyrroleBr1	3.691	179.75	-0.932	-1.38	-42.66	11.34
PyrroleBr2	3.663	179.63	-0.938	-1.41	-42.90	11.39
PyrroleC#N1	3.692	178.71	-0.794	-1.37	-42.66	11.35
PyrroleC#N2	3.670	179.83	-0.801	-1.40	-42.83	11.37
PyrroleCl1	3.676	179.85	-0.942	-1.42	-42.76	11.33
PyrroleCl2	3.679	179.92	-0.945	-1.45	-43.03	11.38
PyrroleF1	5.141	179.82	-0.936	-1.39	-42.33	11.23
PyrroleF2	4.768	179.60	-0.902	-1.49	-42.95	11.32
PyrroleI1	5.147	179.94	-0.948	-1.40	-42.87	11.38
PyrroleI2	3.664	179.64	-0.978	-1.43	-43.37	11.50
PyrroleN1	3.577	179.91	-1.064	-1.43	-42.44	11.22
PyrroleN2	3.620	179.63	-1.016	-1.30	-40.57	10.80
PyrroleNO2_1	5.122	179.90	-0.732	-1.31	-42.60	11.39
PyrroleNO2_2	3.665	179.65	-0.777	-1.37	-42.81	11.39
PyrroleO1	3.608	179.55	-1.036	-1.02	-38.99	10.61
PyrroleO2	4.177	179.77	-0.872	-1.54	-43.17	11.33
PyrroleS1	2.914	174.31	-0.941	-1.51	-43.47	11.45
PyrroleS2	6.162	179.35	-1.009	-1.47	-43.22	11.41
ThiazoleBr1	1.481	133.51	-0.529	-0.63	-37.73	10.62
ThiazoleBr2	1.476	135.16	-0.556	-0.62	-37.33	10.51
ThiazoleBr3	1.479	133.77	-0.527	-0.69	-38.49	10.78
ThiazoleC#N1	1.481	136.44	-0.418	-0.53	-36.48	10.34

ThiazoleC#N2	1.481	135.79	-0.418	-0.88	-39.50	10.90
ThiazoleC#N3	1.482	136.49	-0.418	-0.68	-38.10	10.68
ThiazoleCl1	1.487	134.56	-0.558	-0.56	-36.95	10.46
ThiazoleCl2	1.479	135.49	-0.563	-0.65	-37.54	10.54
ThiazoleCl3	1.488	134.87	-0.553	-0.69	-38.29	10.72
ThiazoleF1	1.480	134.55	-0.566	-1.08	-40.73	11.07
ThiazoleF2	1.477	135.28	-0.585	-0.75	-38.27	10.66
ThiazoleF3	1.483	135.03	-0.574	-0.71	-38.16	10.66
ThiazoleI1	1.488	133.63	-0.581	-0.68	-38.40	10.77
ThiazoleI2	1.478	135.33	-0.597	-1.11	-41.22	11.18
ThiazoleI3	1.485	133.78	-0.576	-0.75	-39.11	10.91
ThiazoleN1	1.472	134.57	-0.682	-1.33	-42.35	11.30
ThiazoleN2	1.476	135.04	-0.612	-0.54	-36.15	10.24
ThiazoleN3	1.481	135.37	-0.688	-0.74	-37.84	10.54
ThiazoleNO2_1	1.492	134.21	-0.358	-0.42	-34.93	10.00
ThiazoleNO2_2	1.476	135.27	-0.387	-0.68	-38.40	10.77
ThiazoleNO2_3	1.491	134.26	-0.357	-0.55	-37.39	10.60
ThiazoleO1	1.468	133.13	-0.547	-1.11	-40.87	11.07
ThiazoleO2	1.476	135.17	-0.584	-0.70	-37.78	10.57
ThiazoleO3	1.478	133.87	-0.554	-0.63	-37.74	10.62
ThiazoleS1	1.474	133.54	-0.632	-1.08	-40.98	11.13
ThiazoleS2	1.478	135.19	-0.636	-0.61	-37.04	10.44

ThiazoleS3	1.478	133.91	-0.630	-0.58	-37.05	10.46
Tio0	1.565	139.94	-0.732	-1.30	-40.03	10.64
Tio1	1.572	140.09	-0.719	-1.80	-44.58	11.50
Tio2	1.571	140.03	-0.717	-1.84	-45.51	11.73
Tio30	1.571	139.10	-0.771	-1.61	-45.21	11.87
Tio430	1.573	138.34	-0.842	-0.76	-41.63	11.66
Tio50	1.577	139.54	-0.779	-1.26	-43.28	11.64
Tio671	1.567	137.61	-0.799	-0.77	-41.04	11.47
TriazoleBr1	5.115	179.49	-0.696	-0.86	-38.27	10.55
TriazoleBr2	4.556	179.76	-0.655	-1.17	-42.04	11.36
TriazoleC#N1	3.382	177.61	-0.576	-0.78	-37.28	10.34
TriazoleC#N2	4.178	178.84	-0.570	-1.19	-41.64	11.22
TriazoleCl1	5.112	179.66	-0.716	-0.85	-37.85	10.43
TriazoleCl2	4.584	179.76	-0.697	-0.62	-37.41	10.53
TriazoleF1	5.046	179.34	-0.730	-0.89	-38.19	10.50
TriazoleF2	4.858	179.76	-0.726	-1.15	-40.79	11.01
TriazoleI1	4.785	179.15	-0.721	-1.45	-43.46	11.51
TriazoleI2	4.509	179.72	-0.701	-1.18	-42.22	11.41
TriazoleN1	3.673	179.39	-0.826	-0.82	-37.58	10.39
TriazoleN2	3.652	179.13	-0.817	-1.41	-42.75	11.34
TriazoleNO2_1	4.955	179.05	-0.548	-0.76	-37.48	10.42
TriazoleNO2_2	4.093	179.49	-0.523	-1.11	-41.21	11.17

TriazoleO1	5.113	179.88	-0.736	-0.83	-38.18	10.55
TriazoleO2	3.677	179.80	-0.834	-0.98	-39.76	10.87
TriazoleS1	4.242	179.62	-0.779	-1.37	-42.54	11.31
TriazoleS2	4.106	179.67	-0.765	-1.14	-41.07	11.11

Table S5: Parameters calculated using the GFN1-xTB method.

Name	R (Å)	θ (°)	ΔQ	ΔH (kcal/mol)	ΔS (cal/molK)	ΔG (kcal/mol)
Acid0	1.878	150.00	-0.584	-1.14	-39.09	10.52
Acid1	1.818	146.97	-0.624	-1.38	-42.28	11.22
Acid2	1.792	145.67	-0.649	-1.88	-46.19	11.90
Acid30	1.911	151.35	-0.401	-0.74	-38.48	10.73
Acid3V2	6.280	121.14	-0.906	-1.71	-44.89	11.68
Acid430	3.012	178.55	-0.118	-0.78	-40.27	11.23
Acid4V2	6.844	121.08	-0.905	-1.27	-41.54	11.12
Acid50	1.828	146.96	-0.457	-1.75	-46.37	12.08
Acid671	1.832	146.21	-0.412	-0.75	-41.02	11.48
Acid6V2	6.402	120.84	-0.901	-1.50	-44.32	11.71
Acid7V2	9.419	121.02	-0.905	-2.11	-49.74	12.72
Acid8V2	12.545	120.98	-0.904	-1.83	-46.73	12.10
AcidBr1	1.865	149.26	-0.523	-1.79	-45.35	11.74
AcidBr2	1.831	147.55	-0.553	-1.71	-45.04	11.72
AcidBr3	2.939	179.20	-0.118	-1.27	-43.39	11.67
AcidBr4	1.953	153.22	-0.306	-1.83	-47.78	12.41

AcidBr5	3.062	179.41	-0.022	-0.06	-33.99	10.08
AcidBr6	1.868	148.18	-0.363	-1.08	-44.55	12.20
AcidCl0	1.904	151.15	-0.436	-1.25	-40.37	10.79
AcidCl1	1.861	148.99	-0.474	-1.13	-37.66	10.10
AcidCl2	1.827	147.44	-0.504	-1.73	-44.84	11.64
AcidCl3	3.049	179.42	-0.082	-1.26	-43.15	11.60
AcidCl4	1.854	148.16	-0.372	-1.93	-49.16	12.73
AcidCl5	2.932	178.92	-0.022	-0.66	-40.02	11.28
AcidCl6	1.828	145.71	-0.386	-1.96	-50.92	13.22
AcidF0	2.013	156.23	-0.457	-1.28	-40.38	10.76
AcidF1	1.884	150.06	-0.532	-1.21	-39.54	10.58
AcidF2	1.836	147.85	-0.568	-1.76	-44.89	11.63
AcidF3	3.596	179.72	-0.147	-1.22	-42.20	11.36
AcidF4	4.822	179.31	-0.118	-1.39	-43.79	11.67
AcidF5	3.232	179.82	-0.042	-0.65	-38.49	10.82
AcidF6	1.867	148.33	-0.356	-0.46	-38.85	11.12
AcidI0	1.916	151.76	-0.497	-1.77	-44.84	11.60
AcidI1	1.866	149.26	-0.538	-1.75	-45.24	11.74
AcidI2	1.829	147.50	-0.571	-1.75	-45.76	11.89
AcidI3	2.968	179.18	-0.141	-1.32	-43.84	11.75
AcidI4	1.942	152.62	-0.318	-1.80	-47.57	12.39
AcidI5	2.907	178.69	-0.080	-0.65	-40.56	11.45

AcidI6	1.828	145.74	-0.393	-1.92	-50.28	13.08
Acid_Sulf0	1.863	149.14	-0.616	-1.78	-45.11	11.67
Acid_Sulf1	1.823	147.18	-0.627	-1.81	-45.99	11.90
Acid_Sulf2	1.830	147.51	-0.633	-1.80	-46.02	11.92
Acid_Sulf30	1.875	149.38	-0.527	-1.82	-47.86	12.45
Acid_Sulf430	3.103	178.97	-0.291	-1.43	-46.68	12.49
Acid_Sulf50	1.913	150.89	-0.494	-1.25	-43.54	11.73
Acid_Sulf671	1.844	146.96	-0.512	-0.61	-41.56	11.78
Acid_Tio0	1.851	148.65	-0.535	-1.20	-39.93	10.71
Acid_Tio1	1.834	147.73	-0.559	-1.72	-44.26	11.48
Acid_Tio2	1.786	145.38	-0.593	-1.89	-46.64	12.02
Acid_Tio30	1.861	148.76	-0.387	-1.27	-43.39	11.67
Acid_Tio430	1.903	150.08	-0.357	-1.61	-47.15	12.44
Acid_Tio50	1.870	149.08	-0.380	-1.84	-47.82	12.42
Acid_Tio671	1.793	143.86	-0.423	-2.05	-51.60	13.33
Alcohol0	1.796	145.96	-0.908	-1.32	-40.06	10.62
Alcohol1	1.813	146.73	-0.819	-1.77	-44.16	11.40
Alcohol2	1.804	146.31	-0.831	-1.91	-46.15	11.86
Alcohol30	1.739	142.83	-0.918	-2.00	-49.82	12.85
Alcohol430	1.809	144.61	-0.830	-0.66	-40.12	11.31
Alcohol50	1.776	144.43	-0.852	-2.01	-49.22	12.66
Alcohol671	1.754	141.97	-0.863	-1.17	-44.92	12.22

Aldehyde0	1.852	148.48	-0.517	-1.36	-40.24	10.63
Aldehyde1	1.813	146.73	-0.561	-1.35	-40.88	10.84
Aldehyde2	1.818	146.97	-0.376	-1.73	-43.79	11.33
Aldehyde30	1.877	149.66	-0.376	-1.38	-43.04	11.46
Aldehyde430	1.766	144.23	-0.425	-0.93	-41.72	11.51
Aldehyde50	1.800	146.11	-0.425	-1.41	-44.05	11.72
Aldehyde671	1.823	145.97	-0.407	-1.94	-50.22	13.03
Amide0	1.825	147.63	-0.646	-1.42	-41.18	10.85
Amide1	1.789	145.70	-0.677	-1.84	-45.04	11.59
Amide2	1.813	146.62	-0.672	-1.92	-46.24	11.87
Amide30	1.851	148.16	-0.483	-1.64	-45.31	11.87
Amide430	1.879	149.22	-0.400	-1.05	-42.69	11.68
Amide50	1.798	146.16	-0.531	-1.57	-45.02	11.85
Amide671	1.826	144.95	-0.483	-1.60	-47.92	12.69
Amine0	1.789	145.60	-0.912	-1.40	-40.53	10.68
Amine1	1.785	145.37	-0.880	-1.88	-45.16	11.58
Amine2	1.789	145.48	-0.847	-1.82	-44.78	11.53
Amine30	1.780	145.16	-0.869	-1.72	-44.46	11.53
Amine430	1.770	142.65	-0.855	-0.56	-39.57	11.24
Amine50	1.747	143.52	-0.861	-2.00	-48.97	12.60
Amine671	1.759	141.76	-0.837	-1.08	-43.67	11.94
Anhydride0	1.904	150.96	-0.347	-1.83	-46.04	11.90

Anhydride1	1.864	149.13	-0.387	-1.79	-46.01	11.93
Anhydride2	1.838	147.94	-0.403	-1.69	-44.72	11.64
Anhydride30	4.761	179.94	-0.012	-1.86	-48.41	12.57
Anhydride3V2	4.651	124.36	-0.670	-1.99	-48.50	12.47
Anhydride430	3.127	179.52	-0.002	-0.84	-41.63	11.57
Anhydride4V2	7.468	124.14	-0.666	-1.87	-47.61	12.33
Anhydride50	1.967	153.63	-0.272	-1.33	-43.99	11.78
Anhydride5V2	7.677	124.15	-0.667	-1.24	-42.64	11.47
Anhydride671	1.837	146.17	-0.373	-1.44	-47.64	12.76
Anhydride7V2	8.280	124.11	-0.667	-1.56	-45.74	12.08
Anhydride8V2	10.903	124.19	-0.663	-2.01	-49.14	12.65
AzolidineBr1	1.851	148.34	-0.666	-0.68	-36.96	10.34
AzolidineBr2	1.803	145.50	-0.692	-0.75	-37.91	10.55
AzolidineC#N1	1.855	149.08	-0.607	-1.19	-41.22	11.10
AzolidineC#N2	1.815	146.12	-0.653	-2.14	-48.74	12.40
AzolidineCl1	1.839	148.25	-0.659	-1.24	-41.71	11.19
AzolidineCl2	1.824	146.20	-0.675	-1.27	-41.63	11.14
AzolidineF1	1.898	150.24	-0.847	-1.25	-41.46	11.11
AzolidineF2	1.829	146.70	-0.872	-1.37	-42.55	11.32
AzolidineI1	1.848	148.15	-0.606	-0.69	-37.73	10.56
AzolidineI2	1.810	145.43	-0.661	-1.24	-41.80	11.22
AzolidineN1	1.769	144.74	-0.900	-1.21	-41.13	11.05

AzolidineN2	1.771	143.94	-0.915	-1.34	-42.13	11.22
AzolidineNO2_1	3.140	179.62	-0.160	-1.79	-46.38	12.04
AzolidineNO2_2	1.885	149.44	-0.391	-1.76	-45.95	11.94
AzolidineO1	1.806	145.61	-0.898	-1.31	-42.01	11.22
AzolidineO2	1.801	146.34	-0.866	-1.31	-41.72	11.13
AzolidineS1	1.809	146.22	-0.810	-1.28	-42.08	11.27
AzolidineS2	1.785	145.56	-0.825	-1.81	-45.79	11.85
Br0	1.931	152.25	-0.637	-1.26	-37.39	9.89
Br1	1.823	147.19	-0.692	-1.34	-40.66	10.78
Br2	1.819	146.99	-0.693	-1.79	-45.01	11.63
Br3	2.021	156.49	-0.465	-0.31	-35.21	10.19
Br4	1.838	147.90	-0.524	-1.99	-49.00	12.62
Br5	2.982	179.67	-0.333	-1.38	-46.18	12.38
Br6	1.807	144.62	-0.486	-0.90	-41.97	11.61
Cl0	5.522	179.94	-0.478	-1.30	-38.66	10.22
Cl1	1.800	146.08	-0.693	-1.43	-41.26	10.87
Cl2	1.825	147.31	-0.662	-1.88	-45.68	11.74
Cl3	3.473	179.62	-0.372	-1.47	-44.61	11.83
Cl4	1.912	151.08	-0.550	-1.41	-43.41	11.53
Cl5	2.927	179.64	-0.322	-0.94	-40.07	11.01
Cl6	1.843	147.06	-0.530	-1.02	-43.94	12.08
Cyan0	1.924	152.10	-0.574	-1.58	-41.67	10.84

Cyan1	1.825	147.22	-0.652	-1.49	-41.79	10.97
Cyan2	1.798	145.96	-0.679	-1.31	-41.29	11.00
Cyan30	2.010	156.22	-0.392	-1.05	-40.66	11.07
Cyan430	2.936	179.80	-0.224	-0.77	-37.35	10.37
Cyan50	1.881	150.00	-0.474	-1.52	-44.11	11.63
Cyan671	1.844	146.46	-0.472	0.11	-34.43	10.37
Ester0	1.871	149.60	-0.585	-1.76	-44.43	11.49
Ester1	1.845	148.24	-0.607	-1.77	-45.20	11.70
Ester2	1.813	146.73	-0.628	-1.94	-47.15	12.11
Ester30	1.933	151.60	-0.391	-1.68	-45.97	12.03
Ester3V2	5.918	123.47	-0.809	-1.79	-45.29	11.71
Ester430	2.832	178.27	-0.131	-0.80	-40.98	11.42
Ester4V2	6.832	123.38	-0.810	-1.89	-46.68	12.03
Ester50	1.831	146.96	-0.464	-1.90	-48.39	12.53
Ester5V2	8.271	123.33	-0.808	-1.81	-46.01	11.91
Ester671	1.827	146.00	-0.416	-1.48	-47.41	12.66
Ester6V2	10.029	123.32	-0.809	-1.30	-42.78	11.46
Ester7V2	10.535	123.29	-0.810	-1.82	-46.09	11.92
Ester8V2	12.539	123.29	-0.809	-1.80	-46.95	12.19
Ether0	1.852	148.70	-0.817	-2.29	-47.30	11.81
Ether1	1.803	146.29	-0.857	-1.92	-45.71	11.71
Ether2	1.809	146.52	-0.818	-1.95	-46.08	11.79

Ether30	1.859	148.85	-0.756	-0.28	-34.66	10.05
Ether430	1.930	151.44	-0.716	-0.91	-41.36	11.42
Ether50	1.816	146.32	-0.797	-2.00	-48.89	12.57
Ether671	1.814	145.25	-0.787	-0.39	-37.25	10.72
F0	5.570	179.87	-0.754	-1.44	-39.42	10.31
F1	1.806	146.41	-0.888	-1.42	-41.10	10.83
F2	1.800	146.11	-0.871	-1.42	-41.99	11.10
F3	5.075	179.96	-0.672	-0.78	-37.50	10.40
F4	5.021	179.51	-0.635	-0.96	-40.34	11.07
F5	5.513	179.98	-0.620	-0.54	-38.84	11.04
F6	1.822	145.69	-0.851	-1.03	-42.52	11.65
I0	1.913	151.36	-0.609	-1.78	-43.14	11.08
I1	1.826	147.29	-0.642	-1.30	-40.59	10.80
I2	1.816	146.85	-0.670	-1.84	-45.67	11.78
I3	1.989	154.89	-0.454	-0.86	-40.15	11.11
I4	1.846	147.97	-0.526	-1.39	-44.64	11.92
I5	2.871	178.39	-0.250	-0.77	-40.57	11.33
I6	1.808	145.19	-0.459	-0.83	-41.79	11.63
ImidazoleBr1	2.661	176.95	-0.340	-1.49	-43.38	11.44
ImidazoleBr2	2.746	177.95	-0.344	-1.23	-41.68	11.19
ImidazoleC#N1	2.801	178.67	-0.138	-1.48	-43.22	11.40
ImidazoleC#N2	2.786	178.54	-0.164	-1.29	-41.81	11.17

ImidazoleCl1	2.736	178.10	-0.317	-1.50	-43.25	11.39
ImidazoleCl2	2.727	177.81	-0.323	-1.26	-41.56	11.13
ImidazoleF1	2.788	178.50	-0.363	-1.49	-42.85	11.28
ImidazoleF2	2.782	178.37	-0.354	-1.22	-41.04	11.02
ImidazoleI1	2.651	176.65	-0.357	-1.53	-43.81	11.53
ImidazoleI2	2.628	176.54	-0.358	-1.37	-42.94	11.43
ImidazoleN1	3.305	179.77	-0.428	-1.12	-39.92	10.78
ImidazoleN2	1.703	142.54	-0.691	-0.90	-39.13	10.76
ImidazoleNO2_1	3.252	178.35	-0.009	-1.41	-43.21	11.48
ImidazoleNO2_2	3.249	178.27	-0.010	-0.76	-38.01	10.57
ImidazoleO1	2.654	176.60	-0.409	-1.59	-43.46	11.37
ImidazoleO2	2.858	178.98	-0.398	-0.91	-38.85	10.67
ImidazoleS1	1.696	142.13	-0.659	-1.17	-40.50	10.91
ImidazoleS2	1.734	144.04	-0.638	-1.04	-40.09	10.91
Nitro0	1.944	152.93	-0.382	-1.45	-41.76	11.00
Nitro1	1.863	149.05	-0.438	-1.06	-39.93	10.85
Nitro3	1.794	145.74	-0.487	-1.75	-46.05	11.98
Nitro30	3.115	179.37	-0.010	-1.39	-45.39	12.15
Nitro430	3.368	178.45	-0.008	-0.35	-39.98	11.57
Nitro50	1.857	148.53	-0.350	-1.49	-46.52	12.37
Nitro671	1.783	143.50	-0.410	-0.85	-42.80	11.91
PiperidineBr1	1.811	145.60	-0.671	-1.62	-45.10	11.82

PiperidineBr2	1.788	144.80	-0.706	-1.64	-45.07	11.79
PiperidineBr3	1.779	144.30	-0.717	-1.65	-45.24	11.84
PiperidineC#N1	1.844	148.37	-0.624	-1.61	-44.57	11.67
PiperidineC#N2	1.800	145.45	-0.660	-1.63	-44.57	11.66
PiperidineC#N3	1.808	146.56	-0.666	-1.88	-46.35	11.94
PiperidineCl1	1.834	146.74	-0.643	-1.11	-40.91	11.09
PiperidineCl2	1.834	147.22	-0.662	-1.16	-40.86	11.03
PiperidineCl3	1.784	144.58	-0.713	-1.68	-45.17	11.78
PiperidineF1	1.868	149.51	-0.808	-1.69	-45.26	11.80
PiperidineF2	1.806	145.76	-0.867	-1.68	-45.12	11.77
PiperidineF3	1.821	146.53	-0.839	-1.26	-41.89	11.23
PiperidineI1	1.824	147.17	-0.610	-1.64	-45.60	11.95
PiperidineI2	1.813	146.16	-0.653	-1.15	-41.05	11.09
PiperidineI3	1.799	145.53	-0.660	-1.63	-44.88	11.75
PiperidineN1	1.767	144.49	-0.869	-1.22	-41.74	11.22
PiperidineN2	1.784	145.33	-0.859	-1.63	-44.21	11.55
PiperidineN3	1.778	145.07	-0.863	-1.67	-44.54	11.61
PiperidineNO2_1	1.950	152.84	-0.357	-1.05	-40.44	11.00
PiperidineNO2_2	1.821	146.62	-0.458	-1.65	-44.98	11.77
PiperidineNO2_3	1.813	146.03	-0.465	-1.23	-41.66	11.20
PiperidineO1	1.791	144.40	-0.864	-1.29	-42.82	11.48
PiperidineO2	1.803	146.32	-0.831	-1.73	-45.15	11.73

PiperidineO3	1.797	146.04	-0.826	-1.64	-44.49	11.63
PiperidineS1	1.772	143.54	-0.809	-1.39	-43.33	11.53
PiperidineS2	1.774	144.01	-0.838	-1.66	-44.91	11.73
PiperidineS3	1.774	143.97	-0.835	-1.65	-44.80	11.71
PyrazineBr1	2.887	179.42	-0.113	-1.20	-42.18	11.38
PyrazineBr2	2.793	179.01	-0.118	-1.16	-41.38	11.18
PyrazineC#N1	2.872	179.54	-0.023	-1.30	-42.34	11.32
PyrazineC#N2	2.884	179.59	-0.025	-1.14	-41.08	11.11
PyrazineCl1	2.877	179.45	-0.085	-1.21	-41.93	11.30
PyrazineCl2	2.824	179.20	-0.087	-1.17	-41.34	11.16
PyrazineF1	2.902	179.63	-0.094	-1.20	-41.61	11.20
PyrazineF2	2.869	179.54	-0.093	-1.20	-41.39	11.14
PyrazineI1	2.880	179.25	-0.131	-1.23	-42.69	11.50
PyrazineI2	1.989	156.00	-0.357	-1.17	-41.73	11.27
PyrazineN1	1.793	144.67	-0.507	-1.27	-42.13	11.29
PyrazineN2	1.821	147.67	-0.495	-1.23	-41.49	11.14
PyrazineNO2_1	3.975	179.11	-0.003	-1.02	-40.60	11.09
PyrazineNO2_2	2.972	179.87	-0.003	-1.13	-41.40	11.21
PyrazineO1	2.882	179.08	-0.133	-1.13	-41.24	11.17
PyrazineO2	1.927	152.95	-0.400	-1.12	-40.95	11.09
PyrazineS1	1.887	149.79	-0.424	-1.10	-40.86	11.08
PyrazineS2	1.876	150.44	-0.439	-1.15	-41.17	11.12

PyrazoleBr1	3.605	179.63	-0.328	-1.12	-40.62	11.00
PyrazoleBr2	3.508	179.65	-0.332	-1.50	-43.60	11.50
PyrazoleC#N1	3.655	179.37	-0.177	-1.13	-40.62	10.98
PyrazoleC#N2	4.480	179.98	-0.241	-1.18	-40.67	10.95
PyrazoleCl1	3.532	179.53	-0.298	-0.57	-35.76	10.09
PyrazoleCl2	3.927	179.47	-0.312	-0.98	-39.20	10.70
PyrazoleF1	4.958	179.61	-0.315	-0.61	-36.07	10.14
PyrazoleF2	5.834	179.99	-0.320	-1.07	-39.77	10.79
PyrazoleI1	3.523	179.73	-0.347	-1.17	-41.35	11.16
PyrazoleI2	3.132	179.84	-0.340	-1.58	-44.72	11.75
PyrazoleN1	3.501	179.88	-0.363	-1.16	-39.95	10.75
PyrazoleN2	2.905	179.11	-0.357	-1.47	-42.73	11.27
PyrazoleNO2_1	3.239	178.23	-0.013	-1.08	-40.51	11.00
PyrazoleNO2_2	3.625	178.93	-0.058	-1.45	-43.54	11.53
PyrazoleO1	4.610	179.68	-0.355	-1.16	-40.11	10.80
PyrazoleO2	3.042	179.75	-0.335	-0.98	-37.98	10.34
PyrazoleS1	2.949	179.39	-0.334	-1.16	-40.61	10.94
PyrazoleS2	3.226	179.85	-0.339	-1.56	-43.70	11.46
PyridineBr1	2.870	179.01	-0.178	-1.20	-42.09	11.35
PyridineBr2	1.858	149.51	-0.462	-1.06	-40.52	11.02
PyridineBr3	1.839	148.60	-0.475	-1.14	-40.44	10.92
PyridineC#N1	2.850	179.21	-0.054	-1.12	-41.06	11.13

PyridineC#N2	1.940	153.54	-0.345	-1.40	-43.20	11.48
PyridineC#N3	1.902	151.71	-0.386	-1.18	-40.56	10.91
PyridineCl1	2.855	179.09	-0.149	-1.21	-41.93	11.29
PyridineCl2	1.854	149.30	-0.456	-1.10	-40.98	11.11
PyridineCl3	1.827	148.00	-0.475	-1.16	-40.42	10.89
PyridineF1	2.861	179.16	-0.158	-1.22	-41.73	11.22
PyridineF2	1.911	152.12	-0.414	-1.13	-41.00	11.09
PyridineF3	1.879	150.56	-0.443	-1.17	-40.10	10.79
PyridineI1	1.936	152.02	-0.394	-1.19	-42.18	11.38
PyridineI2	1.838	148.52	-0.487	-1.10	-41.11	11.15
PyridineI3	1.822	147.79	-0.496	-1.16	-40.81	11.01
PyridineN1	1.741	142.16	-0.563	-1.25	-42.07	11.29
PyridineN2	1.764	144.55	-0.559	-1.12	-40.90	11.07
PyridineN3	1.709	142.12	-0.612	-1.15	-41.36	11.18
PyridineNO2_1	3.196	178.97	-0.015	-1.06	-41.04	11.18
PyridineNO2_2	2.911	179.78	-0.005	-1.11	-41.43	11.24
PyridineNO2_3	2.862	179.57	-0.008	-1.16	-40.62	10.96
PyridineO1	1.747	142.73	-0.531	-1.10	-40.89	11.09
PyridineO2	1.820	147.49	-0.500	-1.16	-41.17	11.12
PyridineO3	1.783	145.77	-0.538	-1.17	-41.46	11.19
PyridineS1	1.814	145.80	-0.488	-1.03	-40.36	11.00
PyridineS2	1.797	146.34	-0.519	-1.11	-40.90	11.08

PyridineS3	1.761	144.72	-0.547	-1.19	-41.86	11.29
PyrimidineBr1	2.888	179.43	-0.143	-1.13	-40.38	10.91
PyrimidineBr2	2.883	179.43	-0.141	-1.13	-41.49	11.24
PyrimidineBr3	2.830	179.26	-0.137	-1.10	-39.44	10.66
PyrimidineC#N1	2.871	179.60	-0.032	-1.07	-39.48	10.70
PyrimidineC#N2	2.867	179.58	-0.020	-1.09	-40.84	11.08
PyrimidineC#N3	2.880	179.62	-0.040	-1.39	-41.63	11.02
PyrimidineCl1	2.878	179.52	-0.112	-1.12	-39.98	10.80
PyrimidineCl2	2.875	179.50	-0.112	-1.17	-41.62	11.24
PyrimidineCl3	2.821	179.18	-0.116	-1.14	-39.76	10.72
PyrimidineF1	2.905	179.67	-0.115	-1.17	-40.05	10.78
PyrimidineF2	2.908	179.63	-0.132	-1.18	-41.38	11.16
PyrimidineF3	2.856	179.46	-0.118	-1.17	-39.84	10.71
PyrimidineI1	2.881	179.21	-0.169	-1.12	-40.37	10.92
PyrimidineI2	2.889	179.29	-0.155	-1.14	-41.69	11.29
PyrimidineI3	2.812	179.14	-0.160	-1.11	-39.58	10.69
PyrimidineN1	1.816	145.82	-0.488	-1.20	-41.82	11.27
PyrimidineN2	1.804	145.15	-0.505	-1.23	-41.84	11.24
PyrimidineN3	1.833	148.22	-0.481	-1.18	-41.27	11.12
PyrimidineNO2_1	3.285	178.81	-0.007	-1.06	-39.83	10.82
PyrimidineNO2_2	3.864	179.02	-0.004	-1.03	-40.75	11.12
PyrimidineNO2_3	3.015	179.76	-0.001	-1.14	-40.18	10.84

PyrimidineO1	2.864	179.09	-0.155	-1.10	-40.97	11.11
PyrimidineO2	1.863	149.01	-0.430	-1.04	-40.37	11.00
PyrimidineO3	1.993	156.05	-0.345	-1.18	-41.35	11.15
PyrimidineS1	1.932	151.99	-0.391	-1.11	-40.96	11.10
PyrimidineS2	2.877	179.40	-0.161	-1.00	-40.13	10.96
PyrimidineS3	1.906	151.89	-0.412	-1.14	-41.10	11.11
PyrroleBr1	4.374	179.83	-0.380	-1.52	-43.59	11.47
PyrroleBr2	4.204	179.76	-0.390	-1.52	-43.65	11.49
PyrroleC#N1	4.377	179.55	-0.188	-1.53	-43.60	11.47
PyrroleC#N2	4.197	179.54	-0.243	-1.53	-43.56	11.46
PyrroleCl1	4.263	179.68	-0.357	-1.52	-43.43	11.43
PyrroleCl2	4.784	179.64	-0.370	-1.55	-43.67	11.47
PyrroleF1	4.984	179.67	-0.391	-1.54	-43.26	11.36
PyrroleF2	6.176	179.34	-0.372	-1.60	-43.65	11.42
PyrroleI1	4.241	179.95	-0.395	-1.48	-43.20	11.40
PyrroleI2	4.196	179.77	-0.395	-1.56	-44.25	11.63
PyrroleN1	4.486	179.96	-0.447	-1.36	-42.15	11.21
PyrroleN2	3.240	179.72	-0.427	-1.36	-42.20	11.22
PyrroleNO2_1	3.649	178.99	-0.036	-1.48	-43.66	11.54
PyrroleNO2_2	3.196	177.76	-0.029	-1.49	-43.63	11.52
PyrroleO1	4.438	179.94	-0.437	-1.13	-39.78	10.73
PyrroleO2	3.681	179.87	-0.412	-1.58	-43.45	11.37

PyrroleS1	3.252	179.64	-0.403	-1.64	-44.23	11.54
PyrroleS2	4.132	179.88	-0.407	-1.58	-43.79	11.47
ThiazoleBr1	2.807	178.76	-0.225	-1.08	-41.09	11.17
ThiazoleBr2	2.808	178.87	-0.226	-1.07	-40.81	11.10
ThiazoleBr3	2.820	178.86	-0.233	-1.07	-40.90	11.13
ThiazoleC#N1	2.852	179.33	-0.066	-1.02	-40.19	10.97
ThiazoleC#N2	2.889	179.45	-0.085	-1.34	-42.67	11.38
ThiazoleC#N3	2.850	179.31	-0.132	-1.06	-40.58	11.04
ThiazoleCl1	2.810	178.87	-0.196	-1.08	-40.90	11.11
ThiazoleCl2	2.824	178.94	-0.202	-1.09	-40.83	11.08
ThiazoleCl3	2.828	179.08	-0.204	-1.09	-40.99	11.13
ThiazoleF1	2.873	179.32	-0.232	-1.14	-41.08	11.11
ThiazoleF2	2.851	179.22	-0.227	-1.16	-41.06	11.09
ThiazoleF3	2.857	179.29	-0.215	-1.12	-40.92	11.08
ThiazoleI1	2.770	178.25	-0.242	-1.08	-41.19	11.20
ThiazoleI2	1.983	155.71	-0.377	-1.07	-40.94	11.13
ThiazoleI3	2.821	178.65	-0.251	-1.09	-41.27	11.21
ThiazoleN1	1.711	141.96	-0.587	-1.29	-41.77	11.16
ThiazoleN2	1.795	146.61	-0.543	-1.27	-41.57	11.13
ThiazoleN3	1.817	146.85	-0.505	-1.32	-42.17	11.25
ThiazoleNO2_1	3.269	178.63	-0.008	-0.98	-40.13	10.99
ThiazoleNO2_2	3.013	179.86	-0.001	-1.11	-41.41	11.24

ThiazoleNO2_3	3.927	178.83	-0.007	-0.98	-40.29	11.03
ThiazoleO1	2.799	178.40	-0.284	-1.14	-40.95	11.07
ThiazoleO2	1.884	150.99	-0.463	-1.11	-40.75	11.04
ThiazoleO3	2.818	178.65	-0.253	-1.13	-40.92	11.07
ThiazoleS1	1.803	146.68	-0.499	-1.07	-40.30	10.95
ThiazoleS2	1.845	149.12	-0.477	-1.12	-40.99	11.10
ThiazoleS3	1.902	151.08	-0.432	-1.05	-40.29	10.97
Tio0	1.778	145.07	-0.818	-1.40	-40.74	10.75
Tio1	1.805	146.29	-0.777	-1.83	-45.00	11.59
Tio2	1.803	146.25	-0.773	-1.69	-43.84	11.38
Tio30	1.760	143.52	-0.741	-1.87	-47.66	12.34
Tio430	1.777	142.64	-0.697	-1.13	-45.06	12.31
Tio50	1.759	143.37	-0.754	-1.58	-46.38	12.25
Tio671	1.772	142.98	-0.711	-1.03	-44.09	12.12
TriazoleBr1	2.812	178.72	-0.290	-0.64	-36.32	10.19
TriazoleBr2	2.905	179.20	-0.274	-1.29	-42.79	11.47
TriazoleC#N1	2.910	179.29	-0.182	-0.65	-35.98	10.08
TriazoleC#N2	2.932	179.37	-0.126	-1.33	-42.43	11.32
TriazoleCl1	2.814	178.75	-0.264	-0.71	-36.66	10.22
TriazoleCl2	2.859	179.17	-0.258	-0.74	-37.09	10.32
TriazoleF1	2.875	179.09	-0.267	-0.73	-36.57	10.17
TriazoleF2	2.897	179.16	-0.276	-0.74	-37.20	10.35

TriazoleI1	2.783	178.19	-0.307	-1.23	-41.37	11.10
TriazoleI2	2.799	178.69	-0.290	-1.32	-42.96	11.49
TriazoleN1	2.919	178.85	-0.313	-0.73	-36.32	10.10
TriazoleN2	2.658	176.67	-0.331	-1.23	-41.41	11.12
TriazoleNO2_1	3.809	178.91	-0.039	-1.11	-40.21	10.88
TriazoleNO2_2	3.942	179.14	0.000	-1.32	-42.76	11.43
TriazoleO1	2.964	179.29	-0.286	-1.02	-38.96	10.60
TriazoleO2	2.783	178.44	-0.312	-1.08	-40.07	10.87
TriazoleS1	2.693	177.56	-0.300	-1.17	-40.07	10.78
TriazoleS2	2.795	178.56	-0.295	-1.32	-42.27	11.28

Table S6: Parameters calculated using the GFN2-xTB method.

Name	R (Å)	θ (°)	ΔQ	ΔH (kcal/mol)	ΔS (cal/molK)	ΔG (kcal/mol)
Acid0	1.773	143.92	-0.689	-0.94	-37.44	10.22
Acid1	1.712	140.99	-0.740	-1.74	-44.81	11.62
Acid2	1.735	141.96	-0.757	-1.73	-45.10	11.72
Acid30	2.723	177.10	-0.141	-1.23	-42.25	11.37
Acid3V2	6.310	119.88	-0.965	-2.18	-48.51	12.29
Acid430	2.837	177.60	-0.045	-0.65	-39.09	11.00
Acid4V2	6.849	119.77	-0.969	-2.22	-49.14	12.43
Acid50	1.785	144.33	-0.534	-1.74	-46.21	12.04
Acid671	1.776	141.95	-0.508	-1.15	-43.94	11.95
Acid6V2	6.204	119.52	-0.975	-2.46	-51.70	12.95

Acid7V2	9.356	119.70	-0.968	-2.55	-53.35	13.36
Acid8V2	12.597	119.69	-0.967	-2.22	-49.94	12.67
AcidBr1	1.833	146.96	-0.506	-1.70	-44.48	11.57
AcidBr2	1.773	143.97	-0.570	-1.61	-44.00	11.51
AcidBr3	2.878	179.51	-0.018	-0.64	-38.25	10.76
AcidBr4	2.770	178.44	-0.024	-1.12	-39.87	10.77
AcidBr5	3.526	179.95	-0.010	0.60	-26.97	8.64
AcidBr6	3.165	179.12	0.001	-0.89	-42.47	11.78
AcidCl0	2.702	177.17	-0.138	-1.16	-39.41	10.59
AcidCl1	1.782	144.44	-0.538	-1.61	-43.26	11.29
AcidCl2	1.753	142.95	-0.575	-1.57	-43.44	11.38
AcidCl3	2.838	178.59	-0.018	-1.16	-42.38	11.47
AcidCl4	1.810	145.35	-0.438	-1.77	-47.62	12.43
AcidCl5	3.038	179.23	-0.008	-0.55	-38.55	10.95
AcidCl6	1.800	143.39	-0.454	-1.77	-48.56	12.70
AcidF0	2.706	177.30	-0.209	-1.23	-39.92	10.67
AcidF1	1.796	145.16	-0.590	-1.67	-43.49	11.30
AcidF2	1.759	143.27	-0.634	-1.57	-43.30	11.34
AcidF3	2.863	178.91	-0.028	-1.12	-40.06	10.83
AcidF4	2.846	178.46	-0.031	-1.31	-43.28	11.59
AcidF5	2.947	179.63	-0.008	-1.28	-44.92	12.11
AcidF6	4.749	179.99	-0.006	-0.87	-40.99	11.35
AcidI0	2.821	178.57	-0.184	-1.71	-44.37	11.52

AcidI1	1.838	147.22	-0.518	-1.68	-44.53	11.60
AcidI2	1.777	144.19	-0.582	-1.57	-43.85	11.50
AcidI3	2.880	179.56	-0.025	-1.20	-42.91	11.59
AcidI4	2.766	178.45	-0.030	-1.66	-46.22	12.12
AcidI5	2.944	178.67	-0.013	-0.52	-39.27	11.19
AcidI6	4.084	178.98	-0.009	-2.37	-53.95	13.71
Acid_Sulf0	1.770	143.87	-0.931	-1.67	-44.44	11.57
Acid_Sulf1	1.749	142.71	-0.968	-1.62	-44.47	11.64
Acid_Sulf2	1.745	142.57	-0.973	-2.14	-47.90	12.14
Acid_Sulf30	1.817	145.70	-0.832	-1.60	-46.19	12.17
Acid_Sulf430	2.849	178.74	-0.470	-0.90	-42.18	11.67
Acid_Sulf50	1.881	148.44	-0.785	-1.09	-42.21	11.50
Acid_Sulf671	1.832	145.27	-0.800	-0.96	-43.88	12.12
Acid_Tio0	1.799	145.26	-0.510	-1.09	-39.21	10.60
Acid_Tio1	1.765	143.80	-0.567	-1.61	-43.28	11.30
Acid_Tio2	1.746	142.46	-0.596	-1.69	-45.14	11.76
Acid_Tio30	2.828	178.29	-0.032	-1.69	-46.45	12.16
Acid_Tio430	2.936	178.33	-0.019	-1.45	-45.61	12.15
Acid_Tio50	1.819	145.55	-0.423	-1.77	-47.13	12.28
Acid_Tio671	1.773	142.07	-0.481	-1.90	-49.79	12.95
Alcohol0	1.725	141.64	-1.319	-1.12	-38.45	10.34
Alcohol1	1.750	142.82	-1.292	-1.62	-42.62	11.09

Alcohol2	1.746	142.48	-1.313	-1.69	-43.72	11.35
Alcohol30	1.706	140.13	-1.315	-1.09	-41.35	11.24
Alcohol430	1.775	141.96	-1.262	-0.91	-42.21	11.68
Alcohol50	1.714	140.77	-1.317	-1.68	-46.39	12.15
Alcohol671	1.701	138.19	-1.325	-0.86	-41.44	11.50
Aldehyde0	1.763	143.49	-0.598	-1.28	-39.37	10.46
Aldehyde1	1.714	141.14	-0.645	-1.24	-40.03	10.70
Aldehyde2	1.746	142.62	-0.655	-1.63	-42.99	11.18
Aldehyde30	1.823	146.22	-0.436	-1.29	-42.28	11.32
Aldehyde430	2.779	177.75	-0.024	-0.76	-39.68	11.07
Aldehyde50	1.780	143.60	-0.484	-1.28	-42.70	11.45
Aldehyde671	1.782	142.68	-0.489	-1.19	-43.87	11.89
Amide0	1.746	142.64	-0.797	-1.31	-40.34	10.72
Amide1	1.726	141.66	-0.841	-1.75	-44.44	11.51
Amide2	1.740	142.35	-0.834	-1.71	-44.33	11.51
Amide30	1.746	141.64	-0.644	-1.62	-45.31	11.89
Amide430	2.663	175.51	-0.122	-0.94	-41.92	11.56
Amide50	1.749	142.58	-0.674	-1.52	-44.58	11.77
Amide671	1.826	143.84	-0.574	-0.81	-40.35	11.22
Amine0	1.730	141.72	-1.311	-1.82	-43.96	11.29
Amine1	1.729	141.67	-1.313	-1.82	-44.54	11.46
Amine2	1.725	141.50	-1.316	-1.24	-40.20	10.74

Amine30	1.695	140.16	-1.343	-1.66	-44.06	11.48
Amine430	1.714	138.79	-1.315	-0.52	-38.88	11.07
Amine50	1.702	140.21	-1.324	-1.85	-47.52	12.31
Amine671	1.695	137.42	-1.329	-1.59	-47.15	12.47
Anhydride0	1.780	144.17	-0.470	-1.74	-45.22	11.74
Anhydride1	1.773	144.10	-0.461	-1.67	-44.69	11.66
Anhydride2	1.757	143.20	-0.480	-1.57	-43.48	11.39
Anhydride30	2.742	178.22	-0.012	-1.76	-47.45	12.39
Anhydride3V2	4.630	122.93	-0.702	-2.36	-51.06	12.86
Anhydride430	2.912	178.42	-0.010	-1.22	-44.68	12.10
Anhydride4V2	7.494	123.92	-0.701	-2.27	-50.28	12.73
Anhydride50	1.941	151.18	-0.323	-1.77	-47.41	12.36
Anhydride5V2	7.680	123.78	-0.703	-1.67	-45.11	11.78
Anhydride671	3.398	179.60	0.005	-1.19	-42.88	11.60
Anhydride7V2	7.804	123.60	-0.706	-1.88	-48.23	12.50
Anhydride8V2	11.248	123.72	-0.704	-1.80	-45.95	11.90
AzolidineBr1	3.935	176.90	-0.116	-1.22	-41.92	11.28
AzolidineBr2	1.782	144.92	-0.634	-0.72	-37.64	10.51
AzolidineC#N1	1.755	143.74	-0.809	-1.17	-40.93	11.03
AzolidineC#N2	1.776	143.38	-0.844	-1.52	-43.78	11.53
AzolidineCl1	1.759	143.74	-0.821	-1.21	-41.44	11.14
AzolidineCl2	1.728	142.05	-0.854	-0.72	-37.38	10.42
AzolidineF1	1.820	146.54	-1.225	-1.21	-41.12	11.05

AzolidineF2	1.780	143.70	-1.273	-1.34	-42.38	11.30
AzolidineI1	4.530	178.48	-0.143	-0.63	-37.30	10.49
AzolidineI2	1.816	145.26	-0.627	-1.21	-41.67	11.22
AzolidineN1	1.654	138.72	-1.342	-1.23	-41.26	11.07
AzolidineN2	1.692	140.42	-1.324	-0.82	-38.08	10.54
AzolidineNO2_1	2.933	177.19	-0.054	-1.79	-46.62	12.11
AzolidineNO2_2	1.766	143.23	-0.489	-1.75	-45.85	11.93
AzolidineO1	1.688	140.27	-1.323	-1.32	-41.94	11.18
AzolidineO2	1.709	141.37	-1.308	-0.73	-37.00	10.31
AzolidineS1	1.787	144.60	-0.653	-1.16	-41.17	11.12
AzolidineS2	1.719	141.86	-0.726	-1.22	-41.23	11.07
Br0	4.736	179.12	-0.186	-1.75	-42.77	11.00
Br1	1.881	149.26	-0.558	-1.21	-39.66	10.61
Br2	1.788	144.73	-0.664	-1.65	-43.64	11.36
Br3	4.909	179.99	-0.064	-1.28	-42.93	11.52
Br4	3.861	179.54	-0.092	-1.88	-48.19	12.49
Br5	3.089	178.44	-0.174	-1.23	-44.95	12.17
Br6	5.491	179.95	-0.175	-1.34	-45.65	12.27
Cl0	3.011	179.36	-0.371	-1.78	-42.55	10.91
Cl1	1.748	142.55	-0.816	-1.81	-44.33	11.41
Cl2	1.758	143.20	-0.823	-1.63	-42.98	11.19
Cl3	4.462	178.91	-0.192	-1.32	-42.71	11.42

Cl4	2.948	179.38	-0.351	-1.84	-46.96	12.16
Cl5	3.184	178.88	-0.227	-0.79	-38.36	10.64
Cl6	1.871	147.29	-0.593	-1.93	-50.51	13.13
Cyan0	1.898	150.32	-0.684	-1.53	-41.36	10.80
Cyan1	1.775	144.04	-0.846	-1.46	-41.53	10.92
Cyan2	1.740	142.28	-0.897	-1.70	-43.99	11.42
Cyan30	2.645	177.52	-0.240	-0.99	-40.19	10.99
Cyan430	2.772	179.29	-0.129	-0.74	-37.14	10.33
Cyan50	1.877	149.28	-0.564	-1.44	-43.32	11.48
Cyan671	1.844	145.70	-0.587	0.15	-34.15	10.33
Ester0	1.782	144.33	-0.675	-1.66	-43.38	11.28
Ester1	1.764	143.60	-0.716	-2.20	-48.53	12.27
Ester2	1.749	142.61	-0.743	-2.23	-48.89	12.35
Ester30	2.589	175.58	-0.125	-1.60	-45.32	11.91
Ester3V2	5.932	123.12	-0.829	-1.73	-44.90	11.65
Ester430	2.723	176.36	-0.081	-0.63	-39.53	11.16
Ester4V2	6.876	123.07	-0.832	-2.24	-49.48	12.51
Ester50	1.773	143.76	-0.521	-1.70	-46.43	12.15
Ester5V2	8.301	122.98	-0.831	-1.73	-45.20	11.75
Ester671	1.778	142.30	-0.480	-1.34	-45.93	12.36
Ester6V2	10.059	122.99	-0.831	-1.70	-45.67	11.92
Ester7V2	10.528	122.96	-0.831	-2.35	-50.46	12.69

Ester8V2	12.591	122.98	-0.831	-2.24	-50.34	12.77
Ether0	2.708	176.89	-1.001	-2.36	-47.64	11.85
Ether1	1.740	142.34	-1.303	-1.81	-44.45	11.44
Ether2	1.740	142.31	-1.303	-2.30	-48.73	12.23
Ether30	2.518	173.87	-1.004	-1.20	-41.76	11.25
Ether430	3.093	176.77	-0.999	-1.26	-43.79	11.80
Ether50	1.775	143.20	-1.268	-1.93	-48.42	12.51
Ether671	1.784	142.51	-1.262	-0.89	-41.46	11.47
F0	2.933	178.95	-0.999	-1.30	-38.36	10.13
F1	1.771	143.74	-1.285	-1.36	-40.59	10.74
F2	1.732	141.95	-1.315	-1.30	-41.11	10.96
F3	4.036	179.29	-0.999	-1.11	-39.67	10.72
F4	2.984	179.42	-0.994	-1.36	-43.49	11.61
F5	2.983	179.56	-0.999	-0.96	-41.90	11.53
F6	3.126	179.66	-0.990	-0.84	-41.18	11.44
I0	5.257	179.78	-0.264	-1.71	-42.71	11.02
I1	1.888	149.62	-0.556	-1.23	-39.92	10.67
I2	1.789	144.76	-0.664	-1.65	-43.72	11.38
I3	4.075	179.77	-0.110	-1.86	-48.28	12.54
I4	3.015	179.50	-0.169	-1.86	-47.97	12.44
I5	4.826	179.67	-0.039	-0.68	-40.32	11.34
I6	2.722	178.38	-0.074	-1.28	-45.39	12.25

ImidazoleBr1	3.185	179.56	-0.346	-1.40	-42.91	11.40
ImidazoleBr2	3.257	179.49	-0.322	-0.89	-38.73	10.66
ImidazoleC#N1	3.195	179.50	-0.135	-1.37	-42.57	11.32
ImidazoleC#N2	3.331	179.60	-0.154	-1.20	-41.27	11.11
ImidazoleCl1	3.082	179.30	-0.368	-1.42	-42.81	11.34
ImidazoleCl2	3.449	179.36	-0.376	-1.22	-41.40	11.12
ImidazoleF1	3.118	179.38	-0.410	-1.42	-42.49	11.25
ImidazoleF2	3.295	179.47	-0.404	-1.05	-39.96	10.86
ImidazoleI1	3.166	179.47	-0.338	-1.39	-42.93	11.41
ImidazoleI2	3.303	179.44	-0.315	-1.23	-41.92	11.27
ImidazoleN1	3.145	178.49	-0.528	-1.18	-40.42	10.87
ImidazoleN2	3.051	178.37	-0.501	-0.95	-39.58	10.85
ImidazoleNO2_1	3.869	178.59	0.005	-1.30	-42.68	11.42
ImidazoleNO2_2	3.867	178.69	0.005	-0.64	-37.29	10.48
ImidazoleO1	3.016	178.61	-0.484	-1.43	-42.48	11.23
ImidazoleO2	3.266	178.80	-0.469	-1.08	-40.15	10.89
ImidazoleS1	3.118	179.16	-0.308	-1.21	-41.60	11.19
ImidazoleS2	3.509	179.55	-0.254	-0.58	-37.19	10.51
Nitro0	1.811	145.93	-0.422	-1.30	-40.90	10.89
Nitro1	1.772	143.93	-0.461	-1.51	-43.25	11.39
Nitro3	1.730	141.77	-0.513	-1.61	-44.85	11.76
Nitro30	1.843	146.97	-0.399	-1.40	-45.60	12.19
Nitro430	4.812	179.91	0.003	-0.30	-39.75	11.55
Nitro50	1.769	143.58	-0.484	-1.51	-46.53	12.37

Nitro671	1.711	138.40	-0.553	-0.75	-42.31	11.87
PiperidineBr1	2.712	177.61	-0.273	-1.51	-44.05	11.63
PiperidineBr2	1.800	144.53	-0.663	-1.55	-44.32	11.67
PiperidineBr3	1.756	142.33	-0.696	-1.54	-44.39	11.69
PiperidineC#N1	1.757	143.69	-0.818	-1.55	-44.11	11.60
PiperidineC#N2	1.770	142.94	-0.856	-1.53	-43.88	11.55
PiperidineC#N3	1.726	141.99	-0.898	-1.81	-45.75	11.83
PiperidineCl1	1.825	145.38	-0.745	-1.53	-44.03	11.60
PiperidineCl2	1.750	143.04	-0.830	-1.05	-39.81	10.82
PiperidineCl3	1.721	140.53	-0.879	-1.58	-44.39	11.66
PiperidineF1	1.808	146.02	-1.237	-1.62	-44.67	11.70
PiperidineF2	1.779	143.41	-1.274	-1.59	-44.48	11.67
PiperidineF3	1.726	141.89	-1.308	-1.17	-41.09	11.08
PiperidineI1	2.637	176.98	-0.282	-1.56	-44.84	11.81
PiperidineI2	1.793	145.33	-0.643	-1.05	-40.15	10.92
PiperidineI3	1.756	143.48	-0.664	-1.57	-44.45	11.68
PiperidineN1	1.673	139.21	-1.345	-1.05	-39.80	10.81
PiperidineN2	1.705	140.81	-1.325	-1.57	-43.71	11.47
PiperidineN3	1.698	140.55	-1.335	-1.60	-44.03	11.53
PiperidineNO2_1	1.840	146.77	-0.419	-0.96	-39.79	10.90
PiperidineNO2_2	1.775	143.42	-0.476	-1.59	-44.60	11.71
PiperidineNO2_3	1.777	143.32	-0.471	-1.12	-40.85	11.06

PiperidineO1	1.728	140.56	-1.297	-1.42	-43.60	11.58
PiperidineO2	1.725	141.80	-1.306	-1.66	-44.58	11.63
PiperidineO3	1.712	141.30	-1.321	-1.62	-44.37	11.60
PiperidineS1	1.780	142.96	-0.674	-1.30	-42.58	11.39
PiperidineS2	1.750	141.79	-0.712	-1.53	-43.92	11.56
PiperidineS3	1.750	141.77	-0.723	-1.53	-43.92	11.57
PyrazineBr1	2.769	178.47	-0.024	-1.16	-41.88	11.32
PyrazineBr2	2.642	177.44	-0.042	-1.15	-41.41	11.19
PyrazineC#N1	2.686	177.85	-0.026	-1.30	-42.27	11.31
PyrazineC#N2	2.618	177.16	-0.031	-1.16	-41.24	11.13
PyrazineCl1	2.707	177.60	-0.031	-1.20	-41.95	11.30
PyrazineCl2	2.609	176.71	-0.044	-0.59	-36.48	10.28
PyrazineF1	2.735	178.00	-0.033	-1.22	-41.76	11.23
PyrazineF2	2.628	177.08	-0.048	-0.63	-36.79	10.34
PyrazineI1	2.780	178.51	-0.025	-1.18	-42.28	11.42
PyrazineI2	2.639	177.43	-0.053	-1.15	-41.52	11.23
PyrazineN1	1.604	135.47	-0.761	-1.36	-42.69	11.36
PyrazineN2	1.649	139.27	-0.734	-1.22	-41.40	11.13
PyrazineNO2_1	3.409	178.98	-0.012	-1.06	-40.88	11.13
PyrazineNO2_2	2.705	178.20	-0.015	-1.16	-41.64	11.25
PyrazineO1	2.611	175.92	-0.075	-0.96	-39.96	10.96
PyrazineO2	1.726	143.36	-0.627	-1.16	-41.21	11.13

PyrazineS1	1.613	137.61	-0.684	-1.08	-40.85	11.10
PyrazineS2	1.731	143.76	-0.603	-1.14	-41.11	11.12
PyrazoleBr1	3.321	179.58	-0.329	-1.05	-40.14	10.91
PyrazoleBr2	3.452	179.54	-0.318	-0.86	-38.79	10.71
PyrazoleC#N1	3.370	179.44	-0.179	-1.08	-40.18	10.90
PyrazoleC#N2	3.410	179.63	-0.229	-1.09	-40.12	10.87
PyrazoleCl1	3.316	179.68	-0.337	-0.51	-35.05	9.94
PyrazoleCl2	4.038	179.81	-0.354	-0.90	-38.80	10.67
PyrazoleF1	3.324	179.60	-0.356	-0.56	-35.17	9.93
PyrazoleF2	4.325	179.51	-0.356	-0.99	-39.30	10.73
PyrazoleI1	3.335	179.51	-0.315	-1.09	-40.65	11.03
PyrazoleI2	3.461	179.52	-0.303	-1.47	-43.90	11.62
PyrazoleN1	3.487	178.72	-0.433	-1.01	-38.28	10.40
PyrazoleN2	3.211	178.89	-0.411	-1.37	-42.19	11.21
PyrazoleNO2_1	3.116	177.98	-0.017	-1.03	-40.08	10.92
PyrazoleNO2_2	3.197	179.44	-0.024	-1.36	-43.08	11.48
PyrazoleO1	3.441	179.38	-0.413	-1.13	-39.85	10.75
PyrazoleO2	3.498	179.65	-0.399	-1.51	-42.99	11.31
PyrazoleS1	3.161	179.70	-0.291	-1.10	-40.11	10.86
PyrazoleS2	3.963	179.72	-0.285	-1.48	-43.30	11.43
PyridineBr1	2.737	177.69	-0.085	-1.13	-41.57	11.26
PyridineBr2	1.774	145.68	-0.576	-1.08	-40.86	11.10

PyridineBr3	1.740	143.97	-0.605	-1.16	-40.66	10.96
PyridineC#N1	1.671	140.96	-0.639	-1.11	-41.02	11.12
PyridineC#N2	1.729	143.46	-0.572	-1.35	-42.70	11.38
PyridineC#N3	1.711	142.54	-0.602	-1.19	-40.58	10.91
PyridineCl1	1.753	143.44	-0.591	-1.17	-41.59	11.24
PyridineCl2	1.708	142.14	-0.645	-1.09	-40.88	11.09
PyridineCl3	1.679	140.73	-0.672	-1.17	-40.45	10.90
PyridineF1	2.656	176.74	-0.111	-1.19	-41.53	11.19
PyridineF2	1.732	143.44	-0.625	-1.14	-41.12	11.12
PyridineF3	1.710	142.32	-0.651	-1.19	-40.25	10.82
PyridineI1	2.759	177.79	-0.093	-1.14	-41.76	11.31
PyridineI2	1.757	144.87	-0.598	-1.07	-40.85	11.11
PyridineI3	1.732	143.61	-0.619	-1.16	-40.81	11.01
PyridineN1	1.570	132.90	-0.832	-1.34	-42.56	11.35
PyridineN2	1.602	136.39	-0.815	-1.12	-40.87	11.06
PyridineN3	1.552	134.23	-0.882	-1.17	-41.44	11.18
PyridineNO2_1	3.337	178.77	-0.016	-1.06	-41.08	11.19
PyridineNO2_2	2.621	176.82	-0.023	-1.11	-41.44	11.24
PyridineNO2_3	1.853	149.48	-0.407	-1.16	-40.66	10.96
PyridineO1	1.596	135.03	-0.773	-1.36	-42.67	11.36
PyridineO2	1.641	138.64	-0.749	-1.16	-41.19	11.12
PyridineO3	1.604	136.96	-0.797	-1.20	-41.62	11.21

PyridineS1	1.608	134.71	-0.733	-1.22	-42.12	11.34
PyridineS2	1.658	139.59	-0.711	-1.11	-40.89	11.09
PyridineS3	1.608	137.25	-0.756	-1.20	-42.00	11.32
PyrimidineBr1	2.777	178.42	-0.026	-1.09	-40.09	10.86
PyrimidineBr2	2.769	178.47	-0.032	-1.08	-41.13	11.18
PyrimidineBr3	2.676	177.71	-0.038	-1.09	-39.48	10.68
PyrimidineC#N1	2.695	177.83	-0.022	-1.08	-39.54	10.71
PyrimidineC#N2	2.708	177.97	-0.019	-1.07	-40.62	11.04
PyrimidineC#N3	2.655	177.56	-0.023	-1.37	-41.42	10.98
PyrimidineCl1	2.721	177.67	-0.027	-1.12	-40.06	10.83
PyrimidineCl2	2.715	177.67	-0.032	-1.12	-41.28	11.19
PyrimidineCl3	2.637	176.95	-0.041	-1.11	-39.53	10.68
PyrimidineF1	2.739	177.95	-0.039	-1.15	-40.02	10.78
PyrimidineF2	2.737	178.01	-0.043	-1.15	-41.19	11.13
PyrimidineF3	2.658	177.32	-0.046	-1.15	-39.76	10.70
PyrimidineI1	2.773	178.38	-0.032	-1.09	-40.22	10.90
PyrimidineI2	2.770	178.45	-0.037	-1.09	-41.35	11.24
PyrimidineI3	2.662	177.58	-0.046	-1.09	-39.56	10.71
PyrimidineN1	1.607	135.64	-0.757	-1.35	-42.70	11.39
PyrimidineN2	1.603	135.34	-0.770	-1.29	-42.15	11.28
PyrimidineN3	1.649	139.30	-0.729	-1.13	-40.88	11.06
PyrimidineNO2_1	3.469	178.75	-0.012	-1.09	-40.15	10.88

PyrimidineNO2_2	3.422	179.49	-0.008	-1.02	-40.64	11.10
PyrimidineNO2_3	2.712	178.22	-0.013	-1.14	-40.16	10.84
PyrimidineO1	2.615	175.97	-0.094	-1.10	-41.06	11.14
PyrimidineO2	1.649	138.49	-0.690	-1.36	-42.61	11.35
PyrimidineO3	1.730	143.59	-0.619	-1.15	-41.08	11.09
PyrimidineS1	1.622	138.17	-0.682	-1.10	-41.04	11.14
PyrimidineS2	1.662	137.69	-0.648	-1.18	-41.78	11.28
PyrimidineS3	1.770	145.65	-0.559	-1.16	-41.20	11.12
PyrroleBr1	3.580	179.82	-0.377	-1.47	-43.26	11.43
PyrroleBr2	3.760	179.78	-0.392	-1.53	-43.72	11.51
PyrroleC#N1	3.566	179.86	-0.197	-1.50	-43.41	11.44
PyrroleC#N2	3.718	179.63	-0.279	-1.53	-43.58	11.46
PyrroleCl1	3.530	179.27	-0.447	-1.49	-43.21	11.39
PyrroleCl2	3.858	179.27	-0.462	-1.56	-43.73	11.48
PyrroleF1	3.585	179.53	-0.480	-1.53	-43.19	11.35
PyrroleF2	4.538	179.44	-0.468	-1.64	-43.97	11.47
PyrroleI1	3.567	179.69	-0.371	-1.48	-43.40	11.46
PyrroleI2	3.649	179.81	-0.381	-1.56	-44.26	11.63
PyrroleN1	3.313	177.41	-0.576	-1.41	-42.41	11.24
PyrroleN2	3.509	178.04	-0.555	-1.40	-42.39	11.24
PyrroleNO2_1	3.848	178.24	0.001	-1.44	-43.43	11.51
PyrroleNO2_2	3.076	177.20	-0.023	-1.48	-43.57	11.51
PyrroleO1	3.590	178.51	-0.545	-1.37	-41.87	11.11

PyrroleO2	3.632	178.66	-0.515	-1.64	-43.80	11.42
PyrroleS1	3.576	179.31	-0.301	-1.21	-41.51	11.16
PyrroleS2	3.764	179.12	-0.355	-1.59	-43.87	11.49
ThiazoleBr1	2.719	178.14	-0.038	-1.03	-40.72	11.11
ThiazoleBr2	2.694	177.80	-0.030	-1.06	-40.77	11.10
ThiazoleBr3	2.738	178.17	-0.029	-0.50	-36.55	10.40
ThiazoleC#N1	2.712	178.05	-0.016	-1.01	-40.20	10.97
ThiazoleC#N2	2.698	177.91	-0.015	-0.76	-38.10	10.60
ThiazoleC#N3	2.739	178.18	-0.013	-0.51	-36.27	10.30
ThiazoleCl1	2.678	177.39	-0.031	-1.05	-40.64	11.06
ThiazoleCl2	2.677	177.34	-0.027	-0.51	-36.15	10.27
ThiazoleCl3	2.695	177.52	-0.030	-0.55	-36.64	10.38
ThiazoleF1	2.729	177.99	-0.049	-1.09	-40.75	11.06
ThiazoleF2	2.694	177.63	-0.038	-0.63	-36.82	10.35
ThiazoleF3	2.737	177.96	-0.036	-0.55	-36.32	10.28
ThiazoleI1	2.694	177.87	-0.037	-1.04	-40.90	11.15
ThiazoleI2	2.683	177.70	-0.042	-1.05	-40.81	11.11
ThiazoleI3	2.722	178.06	-0.032	-0.52	-36.85	10.47
ThiazoleN1	1.550	134.31	-0.793	-1.22	-41.55	11.17
ThiazoleN2	1.590	137.04	-0.793	-1.22	-41.29	11.09
ThiazoleN3	1.579	135.56	-0.774	-1.42	-42.80	11.34
ThiazoleNO2_1	3.463	178.99	-0.006	-0.95	-39.89	10.94

ThiazoleNO2_2	2.809	178.72	-0.007	-1.11	-41.40	11.24
ThiazoleNO2_3	3.294	179.08	-0.014	-0.97	-40.11	10.99
ThiazoleO1	1.687	141.33	-0.619	-1.23	-41.79	11.22
ThiazoleO2	1.647	140.05	-0.692	-1.16	-41.03	11.08
ThiazoleO3	1.714	142.50	-0.598	-0.43	-35.50	10.16
ThiazoleS1	1.553	135.62	-0.733	-1.07	-40.49	11.00
ThiazoleS2	1.654	140.50	-0.651	-1.05	-40.46	11.01
ThiazoleS3	1.592	137.40	-0.706	-0.42	-35.32	10.11
Tio0	1.738	142.34	-0.680	-1.81	-44.02	11.32
Tio1	1.753	143.02	-0.697	-1.71	-43.75	11.34
Tio2	1.743	142.56	-0.713	-2.22	-48.50	12.24
Tio30	1.744	142.59	-0.602	-1.70	-45.90	11.99
Tio430	1.786	142.48	-0.566	-0.79	-40.78	11.37
Tio50	1.727	141.27	-0.633	-1.87	-48.52	12.59
Tio671	1.744	140.50	-0.611	-0.85	-41.49	11.52
TriazoleBr1	4.145	179.99	-0.277	-0.97	-39.29	10.74
TriazoleBr2	4.036	179.89	-0.252	-1.26	-42.44	11.39
TriazoleC#N1	4.177	179.83	-0.174	-0.99	-39.09	10.67
TriazoleC#N2	4.206	179.90	-0.095	-1.24	-41.64	11.17
TriazoleCl1	4.171	179.76	-0.270	-0.48	-35.14	10.00
TriazoleCl2	4.062	179.31	-0.262	-1.26	-42.29	11.35
TriazoleF1	4.393	179.84	-0.276	-0.53	-35.19	9.96

TriazoleF2	4.117	179.85	-0.305	-1.20	-41.74	11.24
TriazoleI1	4.333	179.83	-0.262	-1.06	-40.22	10.93
TriazoleI2	3.997	179.91	-0.237	-0.70	-38.17	10.68
TriazoleN1	2.944	178.83	-0.318	-0.98	-38.56	10.52
TriazoleN2	3.061	178.88	-0.357	-1.65	-44.71	11.69
TriazoleNO2_1	3.399	179.68	-0.010	-0.96	-39.31	10.76
TriazoleNO2_2	3.700	179.98	-0.003	-1.37	-43.15	11.50
TriazoleO1	3.188	179.52	-0.306	-0.91	-38.43	10.55
TriazoleO2	3.894	179.69	-0.353	-1.21	-41.08	11.04
TriazoleS1	4.370	179.56	-0.234	-1.00	-39.15	10.67
TriazoleS2	3.828	179.30	-0.220	-1.22	-41.55	11.16

Table S7: Parameters calculated using the MNDO method.

Name	R (Å)	θ (°)	ΔQ	ΔH (kcal/mol)	ΔS (cal/molK)	ΔG (kcal/mol)
Acid0	1.607	133.91	-0.517	1.21	11.07	10.32
Acid1	1.591	133.11	-0.545	1.32	11.69	10.24
Acid2	1.583	132.64	-0.558	1.28	9.63	10.82
Acid30	5.357	179.71	0.000	3.08	37.70	4.26
Acid3V2	1.583	132.48	-0.561	1.57	12.63	10.21
Acid430	7.733	179.98	0.000	3.01	41.04	3.18
Acid4V2	1.582	132.67	-0.561	1.22	10.46	10.51
Acid50	7.141	179.88	0.000	2.91	38.07	3.97
Acid671	7.725	179.79	0.000	3.15	42.09	3.01
Acid6V2	1.585	132.79	-0.555	1.00	5.96	11.64

Acid7V2	1.581	132.50	-0.563	1.14	8.28	11.08
Acid8V2	1.581	132.47	-0.564	1.47	14.24	9.64
AcidBr1	1.600	134.05	-0.539	1.26	9.88	10.73
AcidBr2	1.584	132.73	-0.556	1.40	11.31	10.44
AcidBr3	6.993	179.85	0.000	2.87	32.03	5.74
AcidBr4	1.760	142.55	-0.335	1.81	13.03	10.33
AcidBr5	8.203	179.96	0.000	3.25	46.56	1.78
AcidBr6	7.445	179.97	0.000	2.99	33.11	5.53
AcidCl0	1.609	134.29	-0.507	1.21	9.92	10.67
AcidCl1	1.590	133.16	-0.543	1.36	10.51	10.64
AcidCl2	1.584	132.78	-0.555	1.55	16.61	9.01
AcidCl3	5.699	179.74	0.000	3.21	38.60	4.11
AcidCl4	6.077	179.81	0.000	2.91	38.80	3.75
AcidCl5	6.086	179.94	0.000	3.13	41.02	3.32
AcidCl6	5.952	179.72	0.000	2.92	27.74	7.06
AcidF0	1.611	134.58	-0.504	1.33	9.64	10.86
AcidF1	1.592	133.10	-0.542	1.41	12.30	10.15
AcidF2	1.583	132.59	-0.558	1.40	12.26	10.16
AcidF3	6.377	179.92	0.000	2.99	34.69	5.06
AcidF4	5.789	179.67	0.000	2.97	38.19	3.99
AcidF5	7.663	179.98	0.000	3.06	39.43	3.71
AcidF6	7.401	179.94	0.000	2.91	34.87	4.93
AcidI0	1.601	133.97	-0.522	1.11	8.89	10.88
AcidI1	1.587	133.00	-0.549	1.49	12.39	10.21
AcidI2	1.583	132.62	-0.559	1.48	14.35	9.61
AcidI3	6.380	179.85	0.000	2.97	36.41	4.53
AcidI4	1.664	137.02	-0.448	1.48	7.47	11.66

AcidI5	6.136	179.88	0.000	3.18	45.91	1.90
AcidI6	4.148	179.41	0.000	2.93	20.85	9.12
Acid_Halid3V2	1.583	132.52	-0.561	1.59	15.46	9.40
Acid_Halid4V2	1.582	132.59	-0.561	1.32	10.62	10.56
Acid_Halid5V2	1.583	132.28	-0.562	1.21	8.51	11.08
Acid_Halid6V2	1.581	132.47	-0.563	1.53	13.15	10.02
Acid_Halid7V2	1.580	132.47	-0.565	1.44	12.30	10.19
Acid_Halid8V2	1.582	132.56	-0.562	1.37	9.43	10.97
Acid_Sulf0	1.600	134.09	-0.517	1.15	10.59	10.40
Acid_Sulf1	1.600	133.57	-0.538	1.42	11.26	10.48
Acid_Sulf2	1.585	132.76	-0.555	1.35	12.54	10.02
Acid_Sulf30	5.111	179.89	0.000	2.95	34.15	5.18
Acid_Sulf3V2	1.585	132.79	-0.553	1.64	16.92	9.01
Acid_Sulf430	7.760	179.97	0.000	2.68	29.60	6.27
Acid_Sulf4V2	1.582	132.57	-0.561	1.38	14.09	9.60
Acid_Sulf50	7.833	179.77	0.000	3.36	44.63	2.47
Acid_Sulf5V2	1.581	132.52	-0.563	0.94	7.41	11.14
Acid_Sulf671	8.526	179.98	0.000	3.19	45.43	2.05
Acid_Sulf6V2	1.581	132.48	-0.564	1.31	6.90	11.66
Acid_Sulf7V2	1.583	132.54	-0.561	0.86	8.77	10.66
Acid_Sulf8V2	1.581	132.47	-0.563	1.17	7.18	11.44
Acid_Tio0	1.601	133.85	-0.524	1.46	13.36	9.89
Acid_Tio1	1.587	132.96	-0.550	1.51	13.95	9.77
Acid_Tio2	1.583	132.82	-0.557	1.19	9.23	10.85
Acid_Tio30	4.912	179.75	0.000	2.76	29.89	6.26

Acid_Tio3V2	1.582	132.47	-0.562	1.55	13.08	10.06
Acid_Tio430	6.914	179.96	0.000	2.85	30.48	6.18
Acid_Tio4V2	1.581	132.55	-0.562	1.52	14.16	9.71
Acid_Tio50	3.283	179.68	0.000	2.71	30.88	5.92
Acid_Tio5V2	1.582	132.52	-0.562	1.28	9.97	10.72
Acid_Tio671	7.843	179.91	0.000	3.00	28.33	6.96
Acid_Tio6V2	1.589	132.40	-0.556	1.04	9.21	10.71
Acid_Tio7V2	1.581	132.54	-0.563	1.08	9.79	10.58
Acid_Tio8V2	1.582	132.44	-0.562	1.52	12.32	10.26
Alcohol0	1.582	132.57	-0.562	1.28	12.06	10.10
Alcohol1	1.581	132.50	-0.563	1.46	12.82	10.05
Alcohol2	1.582	132.54	-0.561	1.42	14.45	9.53
Alcohol30	1.616	133.76	-0.523	0.99	4.89	11.94
Alcohol3V2	1.581	132.44	-0.564	1.17	7.96	11.21
Alcohol430	6.499	179.91	0.000	2.94	32.20	5.75
Alcohol4V2	1.570	132.65	-0.571	1.04	7.54	11.21
Alcohol50	1.634	135.36	-0.496	1.49	5.81	12.17
Alcohol5V2	1.581	132.48	-0.563	1.22	9.77	10.72
Alcohol671	1.663	133.00	-0.491	1.60	8.45	11.49
Alcohol6V2	1.581	132.49	-0.563	1.52	16.19	9.11
Alcohol7V2	1.580	132.46	-0.564	1.72	14.17	9.91
Alcohol8V2	1.581	132.48	-0.562	1.46	8.52	11.34
Aldehyde0	1.591	133.08	-0.544	1.34	11.98	10.18
Aldehyde1	1.588	132.89	-0.552	1.41	13.30	9.86

Aldehyde2	1.582	132.59	-0.560	1.42	13.97	9.67
Aldehyde30	1.656	136.64	-0.461	1.46	7.17	11.73
Aldehyde430	7.160	179.97	0.000	3.02	34.61	5.12
Aldehyde50	1.660	136.53	-0.457	1.38	7.97	11.42
Aldehyde671	7.305	179.97	0.000	2.98	35.35	4.86
Amide0	1.600	133.66	-0.527	0.98	8.68	10.80
Amide1	1.585	132.75	-0.555	1.33	10.66	10.57
Amide2	1.585	132.74	-0.555	1.46	13.03	9.99
Amide30	7.174	179.92	0.000	3.46	42.89	3.09
Amide3V2	1.581	132.50	-0.563	1.67	19.05	8.40
Amide430	6.246	179.78	0.000	2.87	29.39	6.52
Amide4V2	1.583	132.55	-0.561	1.36	9.50	10.94
Amide50	1.600	138.56	-0.516	0.76	-0.15	13.22
Amide5V2	1.581	132.61	-0.563	0.86	7.13	11.15
Amide671	6.453	179.85	0.000	3.22	37.58	4.43
Amide6V2	1.600	133.12	-0.533	0.32	-2.06	13.35
Amide7V2	1.581	132.50	-0.563	1.24	7.83	11.32
Amide8V2	1.581	132.45	-0.564	1.19	6.39	11.69
Amine0	1.582	132.49	-0.562	1.49	14.90	9.47
Amine1	1.582	132.64	-0.562	1.59	15.91	9.26
Amine2	1.581	132.35	-0.564	1.35	12.18	10.13
Amine30	1.575	133.16	-0.567	1.44	14.85	9.42
Amine3V2	1.582	132.46	-0.562	1.26	10.11	10.66
Amine430	1.665	133.11	-0.486	1.53	7.06	11.84

Amine4V2	1.582	132.56	-0.562	1.30	12.79	9.90
Amine50	1.617	134.14	-0.522	1.38	9.87	10.85
Amine5V2	1.583	132.47	-0.560	1.29	9.85	10.77
Amine671	1.663	133.07	-0.491	1.73	13.31	10.17
Amine6V2	1.581	132.47	-0.564	1.54	12.55	10.21
Amine7V2	1.579	132.31	-0.567	1.44	11.68	10.37
Amine8V2	1.581	132.50	-0.564	1.65	15.16	9.54
Anhydride0	1.600	134.21	-0.524	1.15	11.51	10.13
Anhydride1	1.600	133.23	-0.538	1.14	9.42	10.74
Anhydride2	1.586	132.68	-0.555	1.45	6.48	11.93
Anhydride30	5.204	179.89	0.000	2.90	37.71	4.06
Anhydride3V2	1.584	132.77	-0.556	1.32	8.88	11.08
Anhydride430	9.019	179.84	0.000	3.01	34.52	5.14
Anhydride4V2	1.581	132.62	-0.561	1.42	11.41	10.43
Anhydride50	1.685	137.38	-0.421	1.99	22.44	7.71
Anhydride5V2	1.582	132.61	-0.562	1.30	9.84	10.78
Anhydride671	7.327	179.95	0.000	3.09	31.87	6.00
Anhydride7V2	1.581	132.52	-0.562	1.14	9.84	10.62
Anhydride8V2	1.581	132.52	-0.563	1.13	5.27	11.97
AzolidineBr1	1.640	135.40	-0.473	1.61	13.41	10.03
AzolidineBr2	1.613	134.22	-0.516	1.62	14.67	9.65
AzolidineC#N1	1.629	135.14	-0.486	1.53	11.11	10.63
AzolidineC#N2	1.612	134.26	-0.518	1.58	15.85	9.27
AzolidineCl1	1.629	135.93	-0.475	1.32	8.40	11.23

AzolidineCl2	1.613	134.20	-0.513	1.65	18.09	8.67
AzolidineF1	1.621	134.82	-0.498	1.63	12.71	10.25
AzolidineF2	1.613	134.29	-0.516	1.50	13.01	10.03
AzolidineI1	1.607	134.82	-0.509	1.42	10.90	10.58
AzolidineI2	1.610	134.09	-0.519	1.64	15.43	9.45
AzolidineN1	1.600	133.15	-0.539	1.04	6.42	11.53
AzolidineN2	1.600	141.43	-0.535	0.99	4.19	12.16
AzolidineNO2_1	1.600	142.13	-0.489	1.03	5.77	11.73
AzolidineNO2_2	1.629	135.44	-0.487	1.68	15.59	9.44
AzolidineO1	1.600	132.93	-0.541	1.14	8.23	11.09
AzolidineO2	1.600	133.96	-0.528	0.92	5.49	11.69
AzolidineS1	1.624	134.22	-0.504	1.54	12.80	10.14
AzolidineS2	1.608	134.18	-0.513	1.35	6.51	11.82
Br0	1.607	134.97	-0.509	1.22	11.34	10.25
Br1	1.592	133.29	-0.540	1.25	11.85	10.13
Br2	1.585	132.69	-0.556	1.51	13.45	9.91
Br3	10.305	179.99	0.000	3.28	44.50	2.42
Br4	5.791	179.84	0.000	2.77	29.46	6.40
Br5	5.759	179.86	0.000	3.21	40.07	3.67
Br6	8.080	179.98	0.000	3.00	42.01	2.89
Cl0	1.617	135.52	-0.494	1.41	14.79	9.41
Cl1	1.600	142.40	-0.543	0.94	10.44	10.24
Cl2	1.584	132.74	-0.556	1.37	12.06	10.19
Cl3	8.073	179.94	0.000	2.88	37.67	4.06
Cl4	5.996	179.98	0.000	2.72	28.93	6.50

Cl5	6.339	179.85	0.000	3.11	42.59	2.82
Cl6	7.916	179.97	0.000	3.09	37.28	4.39
Cyan0	1.600	144.17	-0.536	1.07	11.15	10.16
Cyan1	1.586	132.91	-0.552	1.43	14.29	9.58
Cyan2	1.600	132.50	-0.547	0.99	8.68	10.81
Cyan30	1.684	138.74	-0.417	1.55	9.99	10.99
Cyan3V2	1.582	132.54	-0.561	1.31	11.80	10.20
Cyan430	5.628	179.88	0.000	2.85	32.69	5.52
Cyan4V2	1.580	132.57	-0.562	1.50	13.68	9.83
Cyan50	1.653	136.77	-0.461	1.62	16.65	9.07
Cyan5V2	1.582	132.52	-0.562	1.35	12.61	10.00
Cyan671	6.848	179.96	0.000	2.78	28.94	6.56
Cyan6V2	1.582	132.48	-0.562	1.40	9.82	10.89
Cyan7V2	1.600	134.45	-0.549	0.96	6.64	11.39
Cyan8V2	1.580	132.46	-0.565	1.49	11.41	10.50
Ester0	1.603	134.08	-0.518	1.44	13.45	9.84
Ester1	1.587	132.85	-0.551	1.54	14.62	9.59
Ester2	1.584	132.61	-0.557	1.07	5.28	11.91
Ester30	4.252	179.91	0.000	2.99	35.11	4.93
Ester3V2	1.583	132.59	-0.560	1.34	15.76	9.05
Ester430	7.413	180.00	0.000	3.62	48.47	1.59
Ester4V2	1.581	132.51	-0.563	1.33	10.41	10.64
Ester50	1.600	138.83	-0.521	0.82	0.38	13.12
Ester5V2	1.581	132.45	-0.563	1.29	11.00	10.43
Ester671	7.640	179.97	0.000	3.16	41.48	3.21

Ester6V2	1.580	132.46	-0.564	1.30	6.98	11.63
Ester7V2	1.581	132.52	-0.563	1.26	8.27	11.21
Ester8V2	1.581	132.52	-0.563	1.40	10.08	10.81
Ether0	1.600	133.40	-0.538	1.01	8.62	10.85
Ether1	1.584	132.74	-0.558	1.32	11.81	10.21
Ether2	1.581	132.41	-0.563	1.26	10.47	10.55
Ether30	1.615	134.11	-0.521	1.59	12.72	10.21
Ether3V2	1.583	132.33	-0.562	1.34	12.77	9.95
Ether430	6.771	179.96	0.000	3.00	36.11	4.64
Ether4V2	1.600	132.32	-0.548	0.85	3.34	12.27
Ether50	1.600	134.76	-0.532	0.79	-1.24	13.57
Ether5V2	1.581	132.54	-0.563	1.47	13.56	9.84
Ether671	8.906	179.94	0.000	3.21	35.66	5.00
Ether6V2	1.581	132.45	-0.563	1.29	10.13	10.68
Ether7V2	1.580	132.46	-0.565	1.31	8.81	11.10
Ether8V2	1.582	132.59	-0.562	1.08	7.07	11.39
F0	1.600	134.38	-0.519	1.30	14.05	9.52
F1	1.600	133.25	-0.527	1.12	11.22	10.19
F2	1.588	132.86	-0.551	1.42	13.76	9.73
F3	1.678	138.18	-0.422	1.64	12.07	10.45
F4	1.600	137.74	-0.508	0.75	4.55	11.81
F5	5.537	179.93	0.000	2.77	34.32	4.95
F6	7.266	179.98	0.000	2.84	34.67	4.92
Halid2V2	1.587	132.91	-0.552	1.37	13.23	9.84

Halid3V2	1.582	132.52	-0.561	1.37	12.49	10.06
Halid4V2	1.581	132.61	-0.563	1.33	12.75	9.94
Halid5V2	1.581	132.37	-0.564	1.23	8.92	10.99
Halid6V2	1.582	132.58	-0.562	1.37	10.04	10.79
Halid7V2	1.582	132.33	-0.564	1.40	10.99	10.54
Halid8V2	1.581	132.48	-0.564	1.47	9.02	11.19
I0	1.601	134.59	-0.521	1.12	10.15	10.50
I1	1.600	132.73	-0.535	1.05	9.29	10.69
I2	1.583	132.65	-0.559	1.50	13.63	9.85
I3	1.600	142.64	-0.502	1.01	5.13	11.89
I4	1.689	138.56	-0.415	1.47	8.60	11.32
I5	9.550	179.98	0.000	2.93	34.44	5.07
I6	7.298	179.98	0.000	3.10	41.56	3.12
ImidazoleBr1	5.193	179.92	0.000	2.97	37.06	4.33
ImidazoleBr2	6.058	179.95	0.000	3.03	41.93	2.94
ImidazoleC#N1	4.157	179.49	0.000	2.91	32.64	5.60
ImidazoleC#N2	5.721	179.94	0.000	2.95	35.58	4.76
ImidazoleCl1	5.537	179.94	0.000	2.99	37.79	4.14
ImidazoleCl2	4.757	179.69	0.000	2.96	35.19	4.88
ImidazoleF1	5.559	179.94	0.000	2.96	37.94	4.06
ImidazoleF2	4.899	179.80	0.000	2.91	33.40	5.36
ImidazoleI1	5.446	179.94	0.000	2.98	36.83	4.41
ImidazoleN1	4.418	179.50	0.000	2.85	36.90	4.26
ImidazoleN2	4.292	179.49	0.000	2.88	33.97	5.16
ImidazoleNO2_1	4.165	179.05	0.000	2.93	34.93	4.93
ImidazoleNO2_2	4.607	179.60	0.000	2.78	28.87	6.59
ImidazoleO1	5.352	179.87	0.000	2.68	32.20	5.50
ImidazoleO2	6.467	179.99	0.000	2.85	34.59	4.95
ImidazoleS1	4.553	179.65	0.000	2.87	30.13	6.30
ImidazoleS2	5.369	179.85	0.000	2.85	34.48	4.99

Ketone3V2	1.583	132.55	-0.560	1.46	13.13	9.96
Ketone4V2	1.580	132.44	-0.565	1.38	9.66	10.92
Ketone5V2	1.581	132.58	-0.563	1.30	13.88	9.57
Ketone6V2	1.583	132.60	-0.560	1.28	11.86	10.15
Ketone7V2	1.581	132.48	-0.563	1.29	6.23	11.85
Ketone8V2	1.581	132.50	-0.564	1.50	14.89	9.47
Nitro0	1.600	134.30	-0.522	1.09	10.54	10.36
Nitro1	1.589	133.00	-0.548	1.41	12.45	10.11
Nitro3	1.600	133.02	-0.546	1.19	10.70	10.41
Nitro30	1.709	140.15	-0.390	1.58	9.26	11.24
Nitro430	6.579	179.83	0.000	3.06	38.38	4.03
Nitro4V2	1.584	132.47	-0.561	1.27	11.11	10.37
Nitro50	1.657	136.99	-0.455	1.56	11.90	10.42
Nitro5V2	1.581	132.46	-0.563	1.54	14.48	9.63
Nitro671	2.150	158.52	-0.082	1.69	-2.15	14.75
Nitro6V2	1.581	132.61	-0.562	1.49	10.77	10.69
Nitro7V2	1.581	132.51	-0.564	1.39	10.43	10.70
Nitro8V2	1.580	132.46	-0.564	1.45	10.87	10.63
PiperidineBr1	1.658	136.31	-0.455	1.66	11.75	10.57
PiperidineBr2	1.621	134.01	-0.513	1.60	12.10	10.40
PiperidineBr3	1.620	134.14	-0.512	1.63	13.65	9.97
PiperidineC#N1	1.650	136.56	-0.462	1.68	13.26	10.13
PiperidineC#N2	1.623	134.15	-0.508	1.65	13.02	10.18

PiperidineC#N3	1.600	134.56	-0.525	1.08	7.16	11.36
PiperidineCl1	1.665	136.73	-0.446	1.71	12.49	10.40
PiperidineCl2	1.600	141.62	-0.522	1.48	12.94	10.04
PiperidineCl3	1.620	134.13	-0.512	1.57	12.34	10.30
PiperidineF1	1.647	136.10	-0.471	1.78	13.89	10.05
PiperidineF2	1.628	134.66	-0.500	1.60	12.21	10.37
PiperidineF3	1.620	134.11	-0.509	1.53	12.55	10.20
PiperidineI1	1.662	136.78	-0.452	1.75	12.95	10.31
PiperidineI2	1.600	141.48	-0.520	1.71	14.92	9.68
PiperidineI3	1.622	134.51	-0.508	1.68	15.09	9.59
PiperidineN1	1.606	133.12	-0.541	1.41	8.94	11.15
PiperidineN2	1.619	134.23	-0.515	1.66	12.67	10.29
PiperidineN3	1.600	133.85	-0.533	0.99	6.92	11.34
PiperidineNO2_1	5.669	179.56	0.000	2.79	28.22	6.79
PiperidineNO2_2	1.644	135.82	-0.472	1.64	12.44	10.35
PiperidineNO2_3	1.600	138.94	-0.515	1.21	9.74	10.72
PiperidineO1	1.610	133.65	-0.533	1.70	14.48	9.79
PiperidineO2	1.620	134.38	-0.511	1.68	13.22	10.15
PiperidineO3	1.600	135.26	-0.537	0.87	5.79	11.56
PiperidineS1	1.628	134.33	-0.504	1.62	13.81	9.91
PiperidineS2	1.618	133.95	-0.516	1.68	13.13	10.17
PiperidineS3	1.618	133.90	-0.517	1.54	10.97	10.68
PyrazineBr1	1.630	134.26	-0.432	1.81	13.28	10.27

PyrazineBr2	1.603	136.92	-0.448	1.96	16.08	9.57
PyrazineC#N1	1.625	138.32	-0.418	1.86	14.30	10.01
PyrazineC#N2	1.607	137.24	-0.441	1.90	15.54	9.68
PyrazineCl1	1.631	138.67	-0.409	1.91	14.79	9.92
PyrazineCl2	1.606	137.09	-0.443	1.86	15.01	9.80
PyrazineF1	1.627	135.07	-0.429	1.77	13.25	10.23
PyrazineF2	1.608	135.28	-0.452	1.82	17.06	9.15
PyrazineN1	1.568	134.26	-0.511	1.66	12.25	10.42
PyrazineN2	1.587	135.77	-0.478	1.80	14.93	9.76
PyrazineNO2_1	4.381	179.90	0.000	3.02	37.62	4.21
PyrazineNO2_2	1.641	139.49	-0.390	1.88	13.50	10.26
PyrazineO1	1.583	135.42	-0.482	1.60	11.29	10.64
PyrazineO2	1.599	133.25	-0.479	1.76	13.35	10.19
PyrazineS1	1.604	132.41	-0.472	1.77	13.61	10.13
PyrazineS2	1.594	133.37	-0.482	1.70	12.77	10.30
PyrazoleBr1	5.940	179.96	0.000	2.72	33.56	5.13
PyrazoleBr2	4.661	179.82	0.000	3.04	42.26	2.85
PyrazoleC#N1	5.507	179.89	0.000	2.88	33.42	5.33
PyrazoleC#N2	5.094	179.81	0.000	2.99	40.18	3.42
PyrazoleCl1	5.667	179.96	0.000	2.83	33.49	5.26
PyrazoleCl2	4.970	179.77	0.000	2.87	31.49	5.89
PyrazoleF1	5.373	179.94	0.000	2.87	32.95	5.46
PyrazoleF2	5.518	179.89	0.000	2.96	36.10	4.61
PyrazoleI1	5.965	179.99	0.000	2.89	32.76	5.54
PyrazoleI2	1.671	139.38	-0.401	1.81	15.17	9.70
PyrazoleN1	1.659	137.48	-0.443	1.38	11.39	10.40

PyrazoleN2	1.695	139.78	-0.393	1.67	15.10	9.58
PyrazoleNO2_1	5.330	179.98	0.000	3.01	41.01	3.19
PyrazoleNO2_2	5.106	179.86	0.000	2.89	35.57	4.69
PyrazoleO1	1.686	139.22	-0.405	1.48	12.54	10.15
PyrazoleO2	5.044	179.83	0.000	2.91	33.87	5.23
PyrazoleS1	5.916	179.96	0.000	2.82	32.67	5.49
PyrazoleS2	5.547	179.77	0.000	2.96	34.05	5.23
PyridineBr1	1.583	130.76	-0.511	1.73	12.82	10.32
PyridineBr2	1.566	131.23	-0.534	1.73	13.06	10.25
PyridineBr3	1.567	134.19	-0.515	1.85	14.40	9.96
PyridineC#N1	1.579	131.31	-0.512	1.68	12.49	10.37
PyridineC#N2	1.567	131.41	-0.529	1.71	12.85	10.30
PyridineC#N3	1.570	134.49	-0.509	1.81	15.11	9.72
PyridineCl1	1.581	130.93	-0.509	1.69	12.62	10.34
PyridineCl2	1.568	131.40	-0.529	1.72	13.10	10.23
PyridineCl3	1.568	134.25	-0.513	1.82	14.18	10.00
PyridineF1	1.581	135.32	-0.487	1.87	15.80	9.57
PyridineF2	1.571	131.52	-0.525	1.70	13.01	10.24
PyridineF3	1.560	132.33	-0.536	1.56	11.57	10.53
PyridineI1	1.580	134.79	-0.498	1.65	12.27	10.41
PyridineI2	1.565	131.19	-0.535	1.73	12.89	10.30
PyridineI3	1.568	134.28	-0.513	1.87	16.47	9.37
PyridineN1	1.552	132.82	-0.547	1.87	15.29	9.72
PyridineN2	1.559	133.48	-0.534	1.71	12.88	10.28

PyridineN3	1.540	132.09	-0.568	1.62	12.37	10.34
PyridineNO2_1	1.626	138.48	-0.414	2.02	16.28	9.58
PyridineNO2_2	1.587	135.76	-0.474	1.87	14.58	9.94
PyridineNO2_3	1.586	135.71	-0.478	1.83	12.97	10.38
PyridineO1	1.550	129.17	-0.564	1.88	16.08	9.50
PyridineO2	1.563	133.84	-0.525	1.57	11.35	10.59
PyridineO3	1.540	129.59	-0.582	1.61	12.67	10.25
PyridineS1	1.571	134.25	-0.515	1.90	18.63	8.76
PyridineS2	1.562	130.84	-0.543	1.65	11.35	10.68
PyridineS3	1.555	133.28	-0.537	1.74	14.43	9.85
PyrimidineBr1	1.597	136.45	-0.458	1.83	15.09	9.74
PyrimidineBr2	1.593	136.09	-0.464	1.87	14.77	9.87
PyrimidineBr3	1.575	132.36	-0.507	1.75	14.52	9.83
PyrimidineC#N1	1.598	136.57	-0.455	1.82	15.18	9.70
PyrimidineC#N2	1.594	136.19	-0.463	1.90	16.43	9.42
PyrimidineC#N3	1.583	135.58	-0.477	1.88	19.39	8.52
PyrimidineCl1	1.592	132.30	-0.479	1.82	17.53	9.01
PyrimidineCl2	1.597	136.45	-0.456	1.85	14.27	10.01
PyrimidineCl3	1.582	135.52	-0.479	1.80	15.42	9.62
PyrimidineF1	1.601	137.04	-0.443	1.86	16.39	9.38
PyrimidineF2	1.585	132.81	-0.485	1.77	13.66	10.11
PyrimidineF3	1.581	132.69	-0.498	1.74	14.69	9.78

PyrimidineI1	1.593	136.04	-0.466	1.64	12.40	10.36
PyrimidineI2	1.593	135.95	-0.468	1.83	13.01	10.36
PyrimidineI3	1.579	135.31	-0.484	1.84	15.84	9.54
PyrimidineN1	1.558	133.57	-0.525	1.72	13.39	10.14
PyrimidineN2	1.553	133.09	-0.538	2.01	19.93	8.48
PyrimidineN3	1.565	131.46	-0.529	1.64	12.34	10.37
PyrimidineNO2_1	1.681	142.06	-0.335	2.15	18.42	9.08
PyrimidineNO2_2	1.655	140.66	-0.368	2.08	16.87	9.47
PyrimidineNO2_3	1.602	136.83	-0.444	1.84	15.64	9.59
PyrimidineO1	1.568	134.39	-0.502	1.81	16.70	9.24
PyrimidineO2	1.556	130.08	-0.544	1.82	13.76	10.13
PyrimidineO3	1.570	131.73	-0.522	1.68	13.00	10.22
PyrimidineS1	1.569	130.96	-0.521	1.55	11.66	10.48
PyrimidineS2	1.579	135.10	-0.492	1.91	15.42	9.73
PyrimidineS3	1.570	132.08	-0.518	1.89	15.86	9.57
PyrroleBr1	1.671	138.34	-0.420	1.70	13.90	9.97
PyrroleBr2	1.657	137.55	-0.436	1.69	13.90	9.96
PyrroleC#N1	1.683	139.09	-0.404	1.77	14.74	9.79
PyrroleC#N2	1.678	138.81	-0.409	1.77	14.40	9.89
PyrroleCl1	1.674	138.69	-0.413	1.66	13.42	10.07
PyrroleCl2	1.659	137.77	-0.432	1.71	14.05	9.94
PyrroleF1	4.792	179.76	0.000	2.92	34.72	4.98
PyrroleF2	1.663	137.92	-0.428	1.67	13.90	9.94

PyrroleI1	1.663	137.86	-0.431	1.67	13.00	10.20
PyrroleI2	1.657	137.55	-0.436	1.75	14.46	9.85
PyrroleN1	1.646	136.85	-0.455	1.51	13.67	9.84
PyrroleN2	1.600	135.19	-0.517	1.17	9.62	10.71
PyrroleNO2_1	5.679	179.93	0.000	2.95	38.67	3.84
PyrroleNO2_2	5.661	179.97	0.000	2.97	36.15	4.60
PyrroleO1	1.627	135.74	-0.478	1.59	14.75	9.61
PyrroleO2	1.624	135.44	-0.487	1.27	9.43	10.87
PyrroleS1	1.639	136.31	-0.465	1.64	13.39	10.06
PyrroleS2	1.635	136.21	-0.468	1.60	13.23	10.07
ThiazoleBr1	1.568	132.27	-0.499	1.77	13.11	10.27
ThiazoleBr2	1.555	132.48	-0.517	1.75	13.58	10.12
ThiazoleBr3	1.573	135.31	-0.477	1.61	11.70	10.54
ThiazoleC#N1	1.571	133.03	-0.491	1.72	13.06	10.24
ThiazoleC#N2	1.557	132.84	-0.510	1.76	16.57	9.23
ThiazoleC#N3	1.576	135.63	-0.471	1.78	13.36	10.21
ThiazoleCl1	1.573	132.64	-0.490	1.73	12.98	10.27
ThiazoleCl2	1.560	132.69	-0.510	1.75	13.82	10.05
ThiazoleCl3	1.568	132.36	-0.496	1.74	13.39	10.16
ThiazoleF1	1.559	132.90	-0.502	1.72	13.29	10.17
ThiazoleF2	1.562	132.91	-0.504	1.73	13.61	10.08
ThiazoleF3	1.568	133.17	-0.491	1.72	13.29	10.17
ThiazoleI1	1.570	132.42	-0.498	1.78	13.49	10.17

ThiazoleI2	1.554	132.51	-0.518	1.78	13.87	10.06
ThiazoleN1	1.530	131.87	-0.561	2.08	16.48	9.58
ThiazoleN2	1.549	131.88	-0.532	1.64	12.55	10.31
ThiazoleN3	1.560	134.61	-0.504	1.72	13.42	10.13
ThiazoleNO2_1	1.621	138.75	-0.399	2.02	16.68	9.46
ThiazoleNO2_2	1.578	134.20	-0.472	1.78	13.72	10.10
ThiazoleNO2_3	1.608	137.95	-0.415	1.94	14.93	9.90
ThiazoleO1	1.546	133.37	-0.524	1.68	13.29	10.13
ThiazoleO2	1.552	132.05	-0.527	1.71	13.59	10.07
ThiazoleO3	1.560	134.39	-0.499	1.82	14.64	9.87
ThiazoleS1	1.549	130.95	-0.534	1.60	12.07	10.41
ThiazoleS2	1.548	132.06	-0.530	1.73	13.56	10.10
ThiazoleS3	1.566	134.66	-0.492	1.86	16.32	9.40
Tio0	1.587	132.82	-0.550	1.21	11.01	10.34
Tio1	1.582	132.54	-0.561	1.36	13.96	9.61
Tio2	1.582	132.53	-0.562	1.29	10.55	10.56
Tio30	1.638	135.50	-0.488	1.30	8.50	11.18
Tio3V2	1.580	132.47	-0.564	1.51	13.17	9.99
Tio430	5.863	179.77	0.000	3.07	35.11	5.02
Tio4V2	1.582	132.45	-0.563	1.30	11.98	10.14
Tio50	1.640	135.58	-0.484	1.34	8.83	11.12
Tio5V2	1.582	132.53	-0.562	1.48	14.21	9.65
Tio671	7.977	179.99	0.000	3.12	45.25	2.04

Tio6V2	1.580	132.38	-0.564	1.49	14.66	9.53
Tio7V2	1.581	132.36	-0.564	1.56	13.79	9.86
Tio8V2	1.580	132.51	-0.565	1.39	14.45	9.50
TriazoleBr2	5.844	179.97	0.000	2.47	35.58	4.27
TriazoleC#N1	4.734	179.80	0.000	2.89	34.12	5.13
TriazoleC#N2	4.649	179.63	0.000	2.34	30.72	5.60
TriazoleCl1	5.749	179.94	0.000	2.97	39.11	3.72
TriazoleCl2	5.541	179.97	0.000	2.51	36.01	4.19
TriazoleF1	5.601	179.96	0.000	2.92	35.26	4.82
TriazoleF2	5.350	179.98	0.000	2.52	36.97	3.91
TriazoleI2	4.884	179.65	0.000	2.35	27.82	6.47
TriazoleN1	5.428	179.96	0.000	2.73	31.18	5.85
TriazoleN2	4.987	179.82	0.000	2.47	29.33	6.13
TriazoleNO2_1	5.848	179.98	0.000	2.89	31.99	5.77
TriazoleNO2_2	4.749	179.63	0.000	2.48	33.82	4.81
TriazoleO1	5.720	179.95	0.000	2.86	37.48	4.10
TriazoleO2	5.329	179.84	0.000	2.78	33.48	5.21
TriazoleS1	5.237	179.96	0.000	2.89	33.85	5.21
TriazoleS2	6.968	179.91	0.000	2.40	28.88	6.20

Table S8: Parameters calculated using the MNDOD method.

Name	R (Å)	θ (°)	ΔQ	ΔH (kcal/mol)	ΔS (cal/molK)	ΔG (kcal/mol)
Acid0	1.609	134.14	-0.514	1.35	13.87	9.63
Acid1	1.590	132.87	-0.549	1.44	13.67	9.78
Acid2	1.583	132.56	-0.559	1.35	18.22	8.33
Acid30	5.431	179.68	0.000	3.08	41.63	3.08
Acid3V2	1.581	132.35	-0.564	1.70	18.65	8.56
Acid430	7.498	179.94	0.000	3.08	34.72	5.14
Acid4V2	1.581	132.46	-0.563	1.25	7.65	11.39
Acid50	6.528	179.73	0.000	2.91	38.08	3.97

Acid671	7.574	179.99	0.000	3.02	35.30	4.91
Acid6V2	1.581	133.04	-0.561	1.35	11.68	10.28
Acid7V2	1.582	133.11	-0.560	1.21	11.97	10.05
Acid8V2	1.582	132.38	-0.563	1.13	7.01	11.45
AcidBr1	1.588	132.99	-0.547	1.42	11.66	10.36
AcidBr2	1.584	132.61	-0.558	1.50	13.51	9.89
AcidBr3	7.033	179.94	0.000	2.98	37.99	4.06
AcidBr4	2.040	158.32	-0.119	1.94	13.81	10.24
AcidBr5	7.646	179.96	0.000	3.08	38.76	3.94
AcidBr6	7.181	179.93	0.000	2.87	25.11	7.79
AcidCl0	1.612	134.55	-0.505	1.37	9.26	11.02
AcidCl1	1.588	133.09	-0.547	1.41	10.85	10.59
AcidCl2	1.582	132.65	-0.560	1.37	11.71	10.29
AcidCl3	6.639	179.95	0.000	2.83	32.47	5.56
AcidCl4	6.206	179.89	0.000	2.98	36.95	4.38
AcidCl5	6.360	179.97	0.000	2.91	30.15	6.33
AcidCl6	7.055	179.89	0.000	2.98	33.86	5.30
AcidF0	1.612	134.68	-0.501	1.38	12.15	10.17
AcidF1	1.589	133.71	-0.542	1.32	13.10	9.83
AcidF2	1.584	132.69	-0.556	1.36	11.82	10.24
AcidF3	3.824	179.30	0.000	3.00	31.67	5.97
AcidF4	2.071	159.76	-0.103	1.91	13.64	10.26
AcidF5	7.258	179.92	0.000	3.07	40.63	3.37
AcidF6	6.912	179.79	0.000	2.99	38.59	3.90
AcidI0	1.603	134.11	-0.518	1.44	14.33	9.58
AcidI1	1.589	132.95	-0.547	1.54	14.17	9.73
AcidI2	1.583	132.64	-0.558	1.62	15.04	9.55

AcidI3	6.638	179.96	0.000	2.90	32.91	5.50
AcidI4	1.683	138.17	-0.422	1.60	10.64	10.84
AcidI5	6.651	179.89	0.000	3.30	45.45	2.16
AcidI6	6.580	179.95	0.000	3.01	35.76	4.77
Acid_Halid3V2	1.582	132.43	-0.563	1.56	13.53	9.94
Acid_Halid4V2	1.581	132.81	-0.562	1.35	11.50	10.34
Acid_Halid5V2	1.584	132.31	-0.562	1.35	14.00	9.59
Acid_Halid6V2	1.580	132.33	-0.565	1.52	12.78	10.12
Acid_Halid7V2	1.581	132.33	-0.565	1.49	12.07	10.31
Acid_Halid8V2	1.580	133.03	-0.562	1.56	16.68	9.00
Acid_Sulf0	1.596	133.62	-0.531	1.01	7.70	11.12
Acid_Sulf1	1.582	132.55	-0.560	1.32	12.75	9.93
Acid_Sulf2	1.583	132.68	-0.559	1.38	12.79	9.97
Acid_Sulf30	4.922	179.89	0.000	3.40	37.78	4.55
Acid_Sulf3V2	1.584	132.65	-0.557	1.49	11.62	10.44
Acid_Sulf430	6.293	179.92	0.000	3.15	39.20	3.88
Acid_Sulf4V2	1.583	132.52	-0.561	1.55	12.94	10.11
Acid_Sulf50	1.656	136.54	-0.460	1.71	12.17	10.49
Acid_Sulf5V2	1.583	132.33	-0.562	0.99	5.07	11.89
Acid_Sulf671	8.339	179.99	0.000	3.36	39.46	4.00
Acid_Sulf6V2	1.582	133.11	-0.561	1.19	10.04	10.61
Acid_Sulf7V2	1.581	133.00	-0.562	1.31	16.80	8.71
Acid_Sulf8V2	1.581	133.11	-0.561	1.30	12.29	10.05
Acid_Tio0	1.600	133.73	-0.526	1.10	8.20	11.07
Acid_Tio1	1.586	132.69	-0.553	1.55	15.15	9.45

Acid_Tio2	1.585	132.67	-0.557	1.26	13.07	9.77
Acid_Tio30	4.410	179.99	0.000	2.96	34.40	5.12
Acid_Tio3V2	1.581	132.33	-0.565	1.50	11.24	10.57
Acid_Tio430	7.885	179.94	0.000	2.80	31.92	5.69
Acid_Tio4V2	1.581	132.87	-0.563	1.39	10.09	10.80
Acid_Tio50	1.662	136.97	-0.451	1.39	7.52	11.56
Acid_Tio5V2	1.580	132.49	-0.564	1.25	11.05	10.37
Acid_Tio671	7.891	179.86	0.000	3.08	34.17	5.31
Acid_Tio6V2	1.583	132.32	-0.562	1.23	10.14	10.61
Acid_Tio7V2	1.580	132.99	-0.563	1.24	9.12	10.93
Acid_Tio8V2	1.580	132.99	-0.563	1.43	9.21	11.10
Alcohol0	1.582	132.62	-0.560	1.25	13.50	9.64
Alcohol1	1.581	132.36	-0.564	1.64	16.55	9.12
Alcohol2	1.582	132.46	-0.563	1.40	13.16	9.89
Alcohol30	1.618	133.89	-0.520	1.18	5.27	12.02
Alcohol3V2	1.579	132.96	-0.564	1.38	15.16	9.28
Alcohol430	6.449	179.89	0.000	2.85	31.52	5.87
Alcohol4V2	1.571	132.69	-0.572	0.84	3.51	12.21
Alcohol50	1.633	134.99	-0.497	1.31	8.95	11.05
Alcohol5V2	1.583	132.36	-0.563	1.27	11.96	10.12
Alcohol671	1.662	132.94	-0.492	1.71	11.94	10.56
Alcohol6V2	1.581	132.44	-0.564	1.40	11.97	10.25
Alcohol7V2	1.580	133.05	-0.562	1.76	17.50	8.96
Alcohol8V2	1.579	132.97	-0.564	1.47	9.38	11.08

Aldehyde0	1.593	133.20	-0.542	1.22	11.04	10.34
Aldehyde1	1.585	132.73	-0.556	1.39	11.45	10.38
Aldehyde2	1.583	132.47	-0.562	1.35	10.76	10.55
Aldehyde30	1.663	136.82	-0.453	1.40	7.01	11.72
Aldehyde430	7.003	180.00	0.000	3.05	36.88	4.47
Aldehyde50	1.659	136.48	-0.459	1.46	10.60	10.71
Aldehyde671	6.705	179.86	0.000	2.87	28.34	6.83
Amide0	1.594	134.33	-0.531	1.24	10.56	10.50
Amide1	1.582	133.34	-0.556	1.50	16.04	9.14
Amide2	1.584	133.27	-0.556	1.45	14.15	9.64
Amide30	2.192	166.61	-0.057	1.99	10.14	11.38
Amide3V2	1.583	132.37	-0.562	1.42	8.49	11.30
Amide430	6.262	179.82	0.000	2.62	29.98	6.10
Amide4V2	1.584	132.36	-0.562	1.50	15.23	9.37
Amide50	1.657	136.43	-0.460	1.61	8.48	11.49
Amide5V2	1.580	132.45	-0.565	1.17	11.40	10.19
Amide671	7.985	179.98	0.000	3.01	36.04	4.68
Amide6V2	1.586	132.62	-0.555	1.18	8.76	10.99
Amide7V2	1.582	132.30	-0.564	1.49	12.72	10.12
Amide8V2	1.579	132.95	-0.564	1.63	15.04	9.56
Amine0	1.580	132.94	-0.563	1.45	14.65	9.50
Amine1	1.579	132.60	-0.567	1.73	19.44	8.35
Amine2	1.577	132.77	-0.567	1.53	15.82	9.22
Amine30	1.581	133.09	-0.563	1.62	15.57	9.39

Amine3V2	1.581	132.33	-0.565	1.47	15.65	9.22
Amine430	1.665	133.12	-0.486	1.36	2.21	13.12
Amine4V2	1.582	132.44	-0.563	1.43	13.30	9.88
Amine50	1.616	134.11	-0.523	1.44	8.99	11.18
Amine5V2	1.582	132.35	-0.564	1.44	17.16	8.74
Amine671	1.662	132.98	-0.492	1.59	11.50	10.58
Amine6V2	1.580	132.33	-0.566	1.44	15.91	9.11
Amine7V2	1.584	132.26	-0.562	1.57	17.16	8.86
Amine8V2	1.580	133.03	-0.562	1.60	13.06	10.12
Anhydride0	1.603	134.37	-0.515	1.41	11.54	10.38
Anhydride1	1.588	132.96	-0.548	1.41	12.06	10.22
Anhydride2	1.582	132.54	-0.561	1.53	11.01	10.66
Anhydride30	2.938	179.90	-0.001	2.57	25.00	7.53
Anhydride3V2	1.583	133.25	-0.556	1.30	11.92	10.16
Anhydride430	9.138	179.93	0.000	2.98	35.78	4.72
Anhydride4V2	1.581	132.50	-0.563	1.49	14.25	9.66
Anhydride50	1.686	137.54	-0.420	1.56	9.65	11.10
Anhydride5V2	1.579	132.97	-0.564	1.27	8.92	11.03
Anhydride671	7.122	179.97	0.000	3.05	35.63	4.84
Anhydride7V2	1.582	133.14	-0.560	1.27	10.17	10.65
Anhydride8V2	1.580	133.02	-0.563	1.32	10.27	10.67
AzolidineBr1	1.639	135.30	-0.472	1.69	15.27	9.55
AzolidineBr2	1.613	134.21	-0.516	1.63	14.71	9.65

AzolidineC#N1	1.633	135.27	-0.482	1.49	9.56	11.05
AzolidineC#N2	1.612	134.23	-0.518	1.55	12.61	10.20
AzolidineCl1	1.632	135.39	-0.481	1.58	11.58	10.53
AzolidineCl2	1.613	134.16	-0.513	1.67	15.91	9.34
AzolidineF1	1.621	134.84	-0.498	1.71	14.98	9.65
AzolidineF2	1.612	134.22	-0.516	1.52	13.92	9.78
AzolidineI1	1.638	135.14	-0.473	1.64	14.73	9.66
AzolidineI2	1.612	134.13	-0.517	1.63	13.28	10.08
AzolidineN1	1.596	132.96	-0.547	1.51	12.22	10.28
AzolidineN2	1.605	133.66	-0.532	1.53	14.93	9.49
AzolidineNO2_1	2.294	172.71	-0.031	2.05	15.76	9.77
AzolidineNO2_2	1.629	135.44	-0.487	1.72	17.78	8.83
AzolidineO1	1.592	132.83	-0.551	1.56	12.86	10.14
AzolidineO2	1.607	133.64	-0.522	1.30	8.34	11.22
AzolidineS1	1.616	133.31	-0.517	1.47	11.02	10.60
AzolidineS2	1.605	133.68	-0.529	1.68	13.85	9.96
Br0	1.610	134.97	-0.506	1.35	13.76	9.66
Br1	1.591	133.16	-0.542	1.17	10.57	10.43
Br2	1.583	132.65	-0.559	1.41	14.22	9.58
Br4	3.718	179.44	0.000	2.77	29.53	6.38
Br5	5.719	179.95	0.000	3.00	32.66	5.67
Br6	6.883	179.95	0.000	3.01	36.39	4.57
Cl0	1.608	135.00	-0.508	1.42	14.95	9.38
Cl1	1.591	133.11	-0.543	1.32	13.00	9.86

Cl2	1.583	132.62	-0.558	1.15	8.53	11.01
Cl3	8.947	179.96	0.000	3.03	40.50	3.37
Cl4	5.728	179.97	0.000	2.82	33.28	5.31
Cl5	7.081	179.93	0.000	3.20	42.90	2.82
Cl6	7.853	179.94	0.000	3.14	45.97	1.85
Cyan0	1.598	134.56	-0.524	1.32	12.40	10.04
Cyan1	1.585	132.98	-0.553	1.45	14.03	9.68
Cyan2	1.582	132.33	-0.560	1.25	11.16	10.33
Cyan30	1.683	138.71	-0.418	1.77	14.86	9.75
Cyan3V2	1.581	133.04	-0.561	1.41	13.29	9.86
Cyan430	5.741	179.92	0.000	2.77	33.48	5.21
Cyan4V2	1.580	132.33	-0.565	1.54	15.28	9.39
Cyan50	1.650	136.65	-0.465	1.62	12.73	10.24
Cyan5V2	1.581	132.25	-0.565	1.26	8.62	11.10
Cyan671	6.945	179.97	0.000	3.01	36.80	4.46
Cyan6V2	1.581	132.45	-0.564	1.45	9.12	11.15
Cyan7V2	1.580	132.87	-0.564	1.69	17.03	9.03
Cyan8V2	1.583	132.46	-0.562	0.80	-2.50	13.96
Ester0	1.604	134.06	-0.518	1.28	11.27	10.33
Ester1	1.585	132.66	-0.554	1.20	10.74	10.41
Ester2	1.583	132.62	-0.558	1.29	11.94	10.14
Ester30	4.247	179.91	0.000	2.98	31.96	5.87
Ester3V2	1.582	132.32	-0.564	1.41	12.22	10.18
Ester430	7.033	179.91	0.000	3.61	45.79	2.37
Ester4V2	1.581	133.06	-0.561	1.56	13.54	9.93

Ester50	1.640	135.40	-0.483	1.67	15.70	9.40
Ester5V2	1.582	132.30	-0.563	1.33	9.47	10.91
Ester671	8.123	179.99	0.000	3.17	38.79	4.01
Ester6V2	1.580	133.02	-0.563	1.45	13.89	9.72
Ester7V2	1.580	133.05	-0.562	1.27	8.79	11.06
Ester8V2	1.580	133.02	-0.562	1.51	15.42	9.32
Ether0	1.588	133.01	-0.547	1.06	9.99	10.50
Ether1	1.585	132.71	-0.557	1.37	11.45	10.36
Ether2	1.582	132.36	-0.564	1.28	12.25	10.04
Ether30	1.615	134.10	-0.522	1.70	17.23	8.98
Ether3V2	1.582	132.43	-0.562	1.32	11.63	10.27
Ether430	6.866	179.97	0.000	3.17	43.35	2.66
Ether4V2	1.583	132.43	-0.561	1.12	8.06	11.13
Ether50	1.626	134.23	-0.511	1.35	4.19	12.51
Ether5V2	1.582	132.40	-0.563	1.52	13.75	9.84
Ether671	9.046	179.98	0.000	3.23	38.71	4.10
Ether6V2	1.577	132.34	-0.567	1.23	7.41	11.44
Ether7V2	1.580	132.97	-0.562	1.53	13.66	9.87
Ether8V2	1.580	133.15	-0.561	1.46	14.89	9.43
F0	1.592	134.13	-0.531	1.33	13.69	9.66
F1	1.591	133.25	-0.545	1.20	12.12	10.00
F2	1.589	133.04	-0.547	1.27	10.92	10.42
F3	1.669	137.48	-0.434	1.60	11.44	10.60
F4	1.667	135.81	-0.448	1.49	9.97	10.93

F5	5.403	179.87	0.000	2.81	30.63	6.09
F6	7.068	179.92	0.000	2.97	37.90	4.08
Halid2V2	1.587	132.86	-0.553	1.39	12.95	9.94
Halid3V2	1.581	132.88	-0.561	1.45	13.43	9.86
Halid4V2	1.579	132.88	-0.565	1.32	10.71	10.54
Halid5V2	1.582	132.75	-0.563	1.35	11.72	10.27
Halid6V2	1.582	132.33	-0.563	1.44	13.03	9.97
Halid7V2	1.582	132.33	-0.566	1.43	10.28	10.78
Halid8V2	1.581	133.08	-0.561	1.56	13.85	9.84
I0	1.610	134.93	-0.508	1.44	14.28	9.60
I1	1.587	133.16	-0.543	1.37	17.22	8.65
I2	1.583	132.58	-0.560	1.34	11.20	10.41
I3	10.850	179.98	0.000	3.06	39.69	3.64
I4	4.438	179.82	0.000	2.89	35.09	4.84
I5	8.940	179.89	0.000	2.99	38.08	4.05
I6	6.249	179.94	0.000	3.32	49.08	1.10
ImidazoleBr1	4.796	179.75	0.000	2.99	37.42	4.24
ImidazoleBr2	4.270	179.54	0.000	2.94	33.34	5.42
ImidazoleC#N1	4.034	179.39	0.000	2.88	30.05	6.34
ImidazoleC#N2	1.763	144.17	-0.299	1.78	13.67	10.12
ImidazoleCl1	4.378	179.90	0.000	2.90	36.16	4.53
ImidazoleCl2	3.941	179.36	0.000	2.94	34.04	5.20
ImidazoleF1	5.628	179.96	0.000	3.02	40.83	3.26
ImidazoleF2	5.285	179.85	0.000	2.89	33.00	5.46
ImidazoleI1	2.175	166.20	-0.049	2.40	20.62	8.66
ImidazoleN1	4.320	179.61	0.000	2.79	31.42	5.84
ImidazoleN2	3.571	179.38	0.000	2.91	29.44	6.55
ImidazoleNO2_1	4.789	179.73	0.000	2.97	33.88	5.28
ImidazoleNO2_2	5.437	179.89	0.000	2.94	36.58	4.44

ImidazoleO1	4.547	179.91	0.000	2.76	35.58	4.57
ImidazoleO2	5.284	179.87	0.000	2.97	36.48	4.50
ImidazoleS1	5.786	179.93	0.000	2.95	36.08	4.61
ImidazoleS2	5.446	179.99	0.000	2.82	30.86	6.03
Ketone3V2	1.580	132.85	-0.563	1.61	15.75	9.33
Ketone4V2	1.582	132.65	-0.563	1.50	12.25	10.26
Ketone5V2	1.579	132.49	-0.566	1.30	14.50	9.39
Ketone6V2	1.582	132.48	-0.562	1.21	10.30	10.55
Ketone7V2	1.582	132.31	-0.564	1.61	13.98	9.86
Ketone8V2	1.581	132.31	-0.565	1.56	14.56	9.63
Nitro0	1.598	134.43	-0.523	1.53	13.91	9.80
Nitro1	1.591	133.06	-0.544	1.31	12.32	10.05
Nitro3	1.579	132.89	-0.566	1.49	14.46	9.59
Nitro30	1.717	140.54	-0.379	1.55	5.97	12.19
Nitro430	6.506	179.77	0.000	3.12	44.62	2.23
Nitro4V2	1.582	132.33	-0.564	1.31	12.95	9.87
Nitro50	1.654	136.92	-0.458	1.59	13.71	9.92
Nitro5V2	1.580	132.33	-0.566	1.56	14.77	9.56
Nitro671	6.804	179.98	0.000	3.12	36.72	4.59
Nitro6V2	1.580	133.06	-0.562	1.36	9.40	10.97
Nitro7V2	1.580	133.06	-0.562	1.55	15.81	9.25
Nitro8V2	1.580	133.07	-0.562	1.44	9.26	11.09
PiperidineBr1	1.654	135.99	-0.460	1.78	13.87	10.06
PiperidineBr2	1.625	134.52	-0.504	1.70	13.57	10.06
PiperidineBr3	1.621	134.28	-0.510	1.70	16.91	9.07

PiperidineC#N1	1.648	136.43	-0.465	1.68	12.52	10.35
PiperidineC#N2	1.624	134.37	-0.506	1.60	12.36	10.33
PiperidineC#N3	1.621	134.48	-0.508	1.52	10.89	10.68
PiperidineCl1	1.649	135.60	-0.465	1.73	13.76	10.05
PiperidineCl2	1.629	134.74	-0.497	1.73	13.42	10.14
PiperidineCl3	1.620	134.13	-0.513	1.55	11.95	10.40
PiperidineF1	1.643	136.02	-0.475	1.76	13.34	10.19
PiperidineF2	1.628	134.65	-0.500	1.59	11.97	10.43
PiperidineF3	1.621	134.35	-0.511	1.60	12.42	10.31
PiperidineI1	1.661	136.70	-0.449	1.73	13.07	10.25
PiperidineI2	1.627	134.76	-0.499	1.69	12.91	10.25
PiperidineI3	1.623	134.56	-0.505	1.64	13.44	10.04
PiperidineN1	1.606	133.15	-0.540	1.40	10.07	10.81
PiperidineN2	1.619	134.24	-0.515	1.76	14.91	9.72
PiperidineN3	1.616	134.08	-0.519	1.66	13.69	9.99
PiperidineNO2_1	5.841	179.78	0.000	3.36	41.70	3.34
PiperidineNO2_2	1.644	135.78	-0.472	1.54	9.10	11.24
PiperidineNO2_3	1.628	134.76	-0.498	1.60	11.76	10.51
PiperidineO1	1.610	133.67	-0.533	1.66	13.38	10.08
PiperidineO2	1.622	134.43	-0.509	1.69	13.83	9.98
PiperidineO3	1.618	134.26	-0.515	1.56	12.55	10.23
PiperidineS1	1.638	134.63	-0.488	1.62	12.16	10.41
PiperidineS2	1.616	133.70	-0.521	1.61	12.01	10.44

PiperidineS3	1.616	133.78	-0.520	1.68	14.17	9.87
PyrazineBr1	1.617	133.69	-0.444	1.84	14.12	10.04
PyrazineBr2	1.601	136.73	-0.452	1.86	15.88	9.54
PyrazineC#N1	1.626	138.45	-0.417	1.81	13.29	10.27
PyrazineC#N2	1.605	134.80	-0.458	1.80	14.50	9.89
PyrazineCl1	1.608	133.52	-0.448	1.75	13.16	10.24
PyrazineCl2	1.604	133.96	-0.466	1.76	13.25	10.22
PyrazineF1	1.627	135.07	-0.429	1.77	13.25	10.23
PyrazineF2	1.610	137.43	-0.437	1.87	15.93	9.54
PyrazineI2	1.601	133.78	-0.471	1.78	13.47	10.18
PyrazineN1	1.568	134.21	-0.512	1.78	13.60	10.14
PyrazineN2	1.587	135.69	-0.479	1.67	13.57	10.04
PyrazineNO2_1	4.282	179.92	0.000	2.92	31.04	6.08
PyrazineNO2_2	1.638	139.38	-0.393	1.95	15.68	9.69
PyrazineO1	1.582	131.37	-0.502	1.66	13.35	10.10
PyrazineO2	1.592	135.85	-0.471	1.76	14.40	9.88
PyrazineS1	1.571	131.06	-0.508	1.60	12.40	10.32
PyrazineS2	1.590	135.98	-0.473	1.76	13.66	10.10
PyrazoleBr1	5.216	179.93	0.000	2.92	34.51	5.04
PyrazoleBr2	4.869	179.87	0.000	2.93	34.26	5.12
PyrazoleC#N1	5.453	179.99	0.000	2.98	37.99	4.07
PyrazoleC#N2	4.892	179.78	0.000	2.88	32.68	5.55
PyrazoleCl1	5.472	179.96	0.000	2.87	32.03	5.74
PyrazoleCl2	5.017	179.84	0.000	2.85	30.65	6.13
PyrazoleF1	5.366	179.94	0.000	2.96	37.93	4.06
PyrazoleF2	5.293	179.94	0.000	2.92	35.69	4.69

PyrazoleI1	5.483	179.97	0.000	2.92	36.57	4.43
PyrazoleI2	4.505	179.82	0.000	2.86	33.42	5.31
PyrazoleN1	1.657	137.44	-0.445	1.35	11.09	10.46
PyrazoleN2	1.698	140.02	-0.390	1.70	15.29	9.55
PyrazoleNO2_1	4.778	179.79	0.000	2.96	35.28	4.85
PyrazoleNO2_2	5.187	179.77	0.000	2.98	36.64	4.47
PyrazoleO1	1.687	139.28	-0.404	1.56	14.13	9.76
PyrazoleO2	4.567	179.98	0.000	2.86	33.74	5.22
PyrazoleS1	5.503	179.88	0.000	2.87	36.84	4.30
PyrazoleS2	7.447	179.82	0.000	2.95	35.97	4.64
PyridineBr1	1.572	130.59	-0.519	1.71	12.66	10.35
PyridineBr2	1.567	131.20	-0.533	1.74	13.14	10.23
PyridineBr3	1.565	133.92	-0.519	1.81	14.27	9.96
PyridineC#N1	1.580	135.06	-0.494	1.75	13.28	10.21
PyridineC#N2	1.567	131.41	-0.529	1.71	12.85	10.29
PyridineC#N3	1.570	134.45	-0.509	1.77	13.91	10.04
PyridineCl1	1.568	130.57	-0.519	1.68	12.62	10.33
PyridineCl2	1.564	131.18	-0.537	1.69	12.59	10.35
PyridineCl3	1.563	133.81	-0.523	1.78	13.98	10.03
PyridineF1	1.581	135.23	-0.488	1.81	14.06	10.03
PyridineF2	1.571	131.53	-0.525	1.70	13.02	10.24
PyridineF3	1.563	133.95	-0.520	1.84	15.67	9.58
PyridineI1	1.566	130.49	-0.524	1.71	12.49	10.40
PyridineI2	1.564	131.00	-0.538	1.72	12.77	10.33
PyridineI3	1.566	134.15	-0.516	1.74	12.75	10.35

PyridineN1	1.553	132.86	-0.546	1.75	13.64	10.09
PyridineN2	1.560	133.51	-0.533	1.74	14.19	9.92
PyridineN3	1.533	129.06	-0.597	1.71	14.12	9.92
PyridineNO2_1	1.626	138.49	-0.414	1.50	9.01	11.23
PyridineNO2_2	1.586	135.58	-0.476	1.90	15.71	9.63
PyridineNO2_3	1.587	135.71	-0.478	1.77	12.10	10.58
PyridineO1	1.550	129.17	-0.564	1.88	16.19	9.47
PyridineO2	1.563	133.82	-0.525	1.71	14.82	9.71
PyridineO3	1.540	129.60	-0.582	1.61	12.67	10.25
PyridineS1	1.574	134.10	-0.510	1.82	14.31	9.97
PyridineS2	1.564	133.88	-0.523	1.78	17.11	9.09
PyridineS3	1.547	130.09	-0.571	1.62	12.23	10.38
PyrimidineBr1	1.580	131.74	-0.495	1.78	16.08	9.40
PyrimidineBr2	1.590	135.59	-0.470	1.88	15.16	9.77
PyrimidineBr3	1.575	132.27	-0.509	1.77	14.68	9.80
PyrimidineC#N1	1.598	136.61	-0.454	1.80	15.06	9.73
PyrimidineC#N2	1.592	136.07	-0.465	1.88	14.92	9.84
PyrimidineC#N3	1.583	135.63	-0.476	1.80	15.83	9.49
PyrimidineCl1	1.577	131.88	-0.492	1.72	14.42	9.84
PyrimidineCl2	1.587	135.49	-0.471	1.85	14.52	9.94
PyrimidineCl3	1.578	135.17	-0.487	1.84	16.11	9.45
PyrimidineF1	1.601	137.06	-0.442	1.91	17.68	9.05

PyrimidineF2	1.593	136.31	-0.460	1.86	15.18	9.75
PyrimidineF3	1.581	132.69	-0.498	1.74	14.69	9.78
PyrimidineI1	1.586	135.29	-0.477	1.70	13.13	10.20
PyrimidineI2	1.586	135.21	-0.477	1.90	14.40	10.02
PyrimidineI3	1.572	132.05	-0.514	1.84	18.19	8.83
PyrimidineN1	1.558	133.64	-0.525	1.82	15.25	9.69
PyrimidineN2	1.553	133.02	-0.538	1.95	15.86	9.64
PyrimidineN3	1.565	131.46	-0.529	1.64	12.36	10.36
PyrimidineNO2_1	1.679	141.83	-0.339	2.19	20.96	8.36
PyrimidineNO2_2	1.649	140.02	-0.377	2.01	15.30	9.86
PyrimidineNO2_3	1.603	137.00	-0.442	1.85	15.42	9.67
PyrimidineO1	1.567	134.36	-0.503	1.81	15.77	9.52
PyrimidineO2	1.556	130.08	-0.544	1.82	13.75	10.13
PyrimidineO3	1.569	131.71	-0.522	1.68	12.96	10.23
PyrimidineS1	1.550	130.05	-0.541	1.53	11.22	10.60
PyrimidineS2	1.564	129.92	-0.533	1.69	12.55	10.36
PyrimidineS3	1.570	131.93	-0.520	1.71	12.97	10.25
PyrroleBr1	1.667	138.00	-0.424	1.73	15.66	9.48
PyrroleBr2	1.653	137.29	-0.441	1.73	14.93	9.69
PyrroleC#N1	1.683	139.11	-0.404	1.76	14.60	9.82
PyrroleC#N2	1.678	138.82	-0.409	1.81	15.62	9.56
PyrroleCl1	1.675	138.43	-0.414	1.67	13.19	10.15

PyrroleCl2	1.652	137.22	-0.443	1.68	14.46	9.79
PyrroleF1	1.730	141.91	-0.349	1.83	14.81	9.83
PyrroleF2	1.662	137.89	-0.429	1.69	14.57	9.76
PyrroleI1	1.661	137.45	-0.435	1.69	13.98	9.94
PyrroleI2	1.648	137.08	-0.447	1.72	14.48	9.81
PyrroleN1	1.647	136.92	-0.454	1.49	13.96	9.74
PyrroleN2	1.619	135.06	-0.496	1.50	13.86	9.78
PyrroleNO2_1	5.679	179.95	0.000	3.01	38.36	3.98
PyrroleNO2_2	5.129	179.85	0.000	2.97	36.26	4.57
PyrroleO1	1.630	135.87	-0.475	1.55	12.63	10.20
PyrroleO2	1.623	135.44	-0.488	1.32	12.84	9.91
PyrroleS1	1.635	135.78	-0.474	1.68	15.43	9.49
PyrroleS2	1.629	135.71	-0.479	1.47	10.98	10.61
ThiazoleBr1	1.541	130.25	-0.551	1.72	13.05	10.24
ThiazoleBr2	1.538	130.87	-0.556	1.72	13.30	10.17
ThiazoleBr3	1.545	130.56	-0.541	1.75	13.60	10.11
ThiazoleC#N1	1.547	130.94	-0.541	1.66	12.58	10.33
ThiazoleC#N2	1.542	131.19	-0.547	1.71	13.93	9.97
ThiazoleC#N3	1.560	134.20	-0.505	1.75	13.31	10.19
ThiazoleCl1	1.538	130.48	-0.549	1.68	12.51	10.36
ThiazoleCl2	1.539	130.88	-0.555	1.71	13.36	10.14
ThiazoleCl3	1.556	133.59	-0.511	1.75	13.17	10.24
ThiazoleF1	1.546	131.41	-0.535	1.70	13.18	10.18

ThiazoleF2	1.545	131.29	-0.543	1.70	13.40	10.12
ThiazoleF3	1.545	131.32	-0.537	1.69	12.94	10.24
ThiazoleI1	1.537	130.20	-0.555	1.73	12.88	10.30
ThiazoleI2	1.537	130.75	-0.560	1.72	13.22	10.19
ThiazoleN1	1.537	132.26	-0.551	1.82	15.79	9.52
ThiazoleN2	1.542	132.77	-0.541	2.00	19.59	8.57
ThiazoleN3	1.531	128.94	-0.579	1.54	11.44	10.54
ThiazoleNO2_1	1.562	132.80	-0.504	1.74	12.46	10.44
ThiazoleNO2_2	1.555	132.24	-0.518	1.76	13.34	10.19
ThiazoleNO2_3	1.574	133.57	-0.482	1.49	9.71	11.01
ThiazoleO1	1.541	132.70	-0.539	1.66	12.72	10.28
ThiazoleO2	1.540	130.78	-0.557	1.75	14.03	9.98
ThiazoleO3	1.533	130.05	-0.565	1.66	12.94	10.21
ThiazoleS1	1.524	129.28	-0.579	1.53	11.55	10.50
ThiazoleS2	1.535	130.53	-0.566	1.57	11.64	10.51
ThiazoleS3	1.547	132.58	-0.536	1.78	14.10	9.99
Tio0	1.586	132.80	-0.553	1.34	13.50	9.73
Tio1	1.580	132.46	-0.564	1.54	14.29	9.69
Tio2	1.580	133.00	-0.563	1.61	15.50	9.40
Tio30	1.634	135.11	-0.496	1.77	15.18	9.66
Tio3V2	1.581	132.84	-0.563	1.38	13.55	9.76
Tio430	7.123	179.62	0.000	2.96	32.83	5.58
Tio4V2	1.581	132.33	-0.566	1.28	14.73	9.30

Tio50	1.640	135.44	-0.486	1.37	8.70	11.19
Tio5V2	1.582	132.30	-0.565	1.48	13.56	9.85
Tio671	8.148	179.96	0.000	3.32	39.26	4.03
Tio6V2	1.580	132.32	-0.565	1.40	10.85	10.57
Tio7V2	1.580	133.01	-0.563	1.56	14.86	9.55
Tio8V2	1.580	133.06	-0.562	1.51	13.24	9.98
TriazoleBr2	4.669	179.56	0.000	2.38	27.01	6.74
TriazoleC#N1	5.105	179.87	0.000	2.86	31.03	6.02
TriazoleC#N2	4.665	179.66	0.000	2.31	27.70	6.47
TriazoleCl1	5.422	179.90	0.000	2.98	38.91	3.79
TriazoleCl2	4.493	179.57	0.000	2.47	32.20	5.29
TriazoleF1	4.894	179.82	0.000	2.92	37.31	4.21
TriazoleF2	5.367	179.98	0.000	2.51	35.20	4.43
TriazoleN1	5.148	179.98	0.000	2.89	33.41	5.34
TriazoleN2	5.536	179.97	0.000	2.53	32.38	5.28
TriazoleNO2_1	5.149	179.90	0.000	3.00	37.57	4.21
TriazoleNO2_2	5.331	179.96	0.000	2.39	27.82	6.51
TriazoleO1	4.898	179.87	0.000	2.83	32.09	5.68
TriazoleO2	5.361	179.85	0.000	2.70	33.02	5.27
TriazoleS1	5.321	179.97	0.000	2.84	37.59	4.04
TriazoleS2	5.430	179.79	0.000	2.56	38.61	3.46

Table S9: Parameters calculated using the PM3 method.

Name	R (Å)	θ (°)	ΔQ	ΔH (kcal/mol)	ΔS (cal/molK)	ΔG (kcal/mol)
Acid0	1.600	130.35	-0.704	1.22	11.48	10.21
Acid1	1.587	129.33	-0.731	1.35	10.14	10.74
Acid2	1.582	128.73	-0.755	1.39	13.04	9.91
Acid30	1.860	144.31	-0.318	1.54	7.76	11.64
Acid3V2	1.580	128.94	-0.748	1.60	12.20	10.38
Acid430	6.590	179.81	0.000	2.87	29.82	6.39
Acid4V2	1.577	128.91	-0.750	1.51	14.53	9.59
Acid50	1.659	133.66	-0.564	1.54	9.59	11.10
Acid671	2.244	165.30	-0.059	2.43	14.56	10.50

Acid6V2	1.586	129.35	-0.734	1.40	8.81	11.19
Acid7V2	1.581	128.83	-0.750	1.06	9.93	10.51
Acid8V2	1.579	128.80	-0.751	1.77	19.10	8.49
AcidBr1	1.600	129.54	-0.718	1.45	13.68	9.78
AcidBr2	1.585	129.22	-0.738	1.53	11.13	10.63
AcidBr3	4.405	179.48	0.000	2.87	30.14	6.30
AcidBr4	5.878	179.79	0.000	2.82	36.28	4.41
AcidBr5	6.358	179.77	0.000	3.02	36.01	4.70
AcidBr6	4.389	179.62	0.000	2.91	29.18	6.63
AcidCl0	1.600	130.52	-0.701	1.28	12.33	10.01
AcidCl1	1.587	129.45	-0.732	1.57	15.51	9.36
AcidCl2	1.583	129.03	-0.743	1.50	11.02	10.63
AcidCl3	4.210	179.58	0.000	2.85	32.22	5.65
AcidCl4	1.726	137.24	-0.510	1.60	7.52	11.77
AcidCl5	5.624	179.85	0.000	3.00	33.56	5.41
AcidCl6	4.105	179.86	0.000	2.82	24.18	8.02
AcidF0	1.603	130.87	-0.693	0.69	3.78	11.97
AcidF1	1.589	129.53	-0.728	1.56	12.61	10.21
AcidF2	1.559	130.03	-0.758	1.20	8.29	11.14
AcidF3	4.056	179.62	0.000	2.89	30.69	6.15
AcidF4	4.257	179.81	0.000	2.78	33.53	5.20
AcidF5	6.686	179.71	0.000	3.11	36.29	4.70
AcidF6	4.825	179.39	0.000	3.46	35.72	5.22
AcidI0	1.600	130.76	-0.696	1.14	9.87	10.61
AcidI1	1.600	129.55	-0.717	1.32	12.30	10.07
AcidI2	1.584	129.14	-0.740	1.60	14.94	9.57
AcidI3	5.547	179.73	0.000	3.00	38.07	4.07
AcidI4	5.418	179.63	0.000	2.93	35.35	4.80
AcidI5	5.696	179.57	0.000	3.39	41.04	3.57
AcidI6	4.193	179.88	0.000	3.12	28.34	7.09
Acid_Halid3V2	1.580	128.89	-0.748	1.56	13.57	9.93
Acid_Halid4V2	1.579	128.97	-0.747	1.46	11.70	10.39
Acid_Halid5V2	1.579	128.86	-0.750	1.16	9.23	10.82
Acid_Halid6V2	1.571	129.29	-0.753	1.52	12.25	10.28
Acid_Halid7V2	1.580	128.85	-0.750	1.62	13.79	9.93
Acid_Halid8V2	1.603	134.39	-0.703	1.43	8.66	11.26
Acid_Sulf0	1.590	129.19	-0.732	1.60	11.68	10.53

Acid_Sulf1	1.584	128.57	-0.752	1.31	11.94	10.16
Acid_Sulf2	1.580	128.96	-0.747	1.57	13.75	9.88
Acid_Sulf30	1.682	133.86	-0.572	1.67	10.33	11.01
Acid_Sulf3V2	1.581	128.85	-0.749	1.49	9.12	11.19
Acid_Sulf430	4.954	179.90	0.000	3.03	32.24	5.83
Acid_Sulf4V2	1.580	128.86	-0.749	1.63	10.34	10.95
Acid_Sulf50	1.600	136.74	-0.660	0.82	-2.13	13.87
Acid_Sulf5V2	1.581	128.81	-0.749	1.52	9.44	11.12
Acid_Sulf671	4.326	179.87	0.000	2.73	32.53	5.45
Acid_Sulf6V2	1.616	133.05	-0.701	1.46	10.20	10.84
Acid_Sulf7V2	1.601	130.81	-0.721	1.36	11.05	10.48
Acid_Sulf8V2	1.609	132.53	-0.710	1.47	9.99	10.90
Acid_Tio0	1.600	129.03	-0.723	1.43	11.94	10.28
Acid_Tio1	1.586	129.09	-0.735	1.68	16.13	9.29
Acid_Tio2	1.600	141.19	-0.718	1.18	8.54	11.05
Acid_Tio30	1.730	137.49	-0.507	1.85	10.39	11.17
Acid_Tio3V2	1.574	129.28	-0.750	1.48	14.22	9.65
Acid_Tio430	6.757	179.89	0.000	3.72	42.40	3.49
Acid_Tio4V2	1.582	129.05	-0.746	1.58	12.29	10.33
Acid_Tio50	1.600	142.10	-0.622	1.52	6.60	11.97
Acid_Tio5V2	1.579	128.87	-0.750	1.25	8.18	11.23
Acid_Tio671	4.759	179.76	0.000	3.18	36.77	4.64
Acid_Tio6V2	1.580	128.84	-0.749	1.11	10.24	10.47
Acid_Tio7V2	1.580	128.88	-0.750	0.92	5.87	11.59
Acid_Tio8V2	1.608	133.91	-0.705	1.36	6.81	11.74
Alcohol0	1.582	129.03	-0.746	1.43	12.98	9.97
Alcohol1	1.582	128.87	-0.747	1.43	12.13	10.23
Alcohol2	1.580	128.95	-0.748	1.38	10.86	10.56
Alcohol30	1.600	131.02	-0.701	1.29	9.08	10.99
Alcohol3V2	1.582	128.91	-0.746	1.64	15.41	9.46
Alcohol430	1.695	132.03	-0.567	1.68	12.59	10.34
Alcohol4V2	1.580	128.79	-0.751	1.02	7.70	11.14
Alcohol50	1.637	132.14	-0.644	1.53	9.65	11.07
Alcohol5V2	1.580	128.92	-0.748	1.51	11.77	10.42
Alcohol671	1.687	131.22	-0.606	1.77	12.93	10.33
Alcohol6V2	1.581	128.94	-0.747	1.55	12.48	10.24
Alcohol7V2	1.607	133.15	-0.709	1.16	6.80	11.54

Alcohol8V2	1.609	134.06	-0.702	1.44	12.41	10.15
Aldehyde0	1.587	129.31	-0.734	1.32	10.85	10.50
Aldehyde1	1.579	128.87	-0.741	1.12	8.64	10.96
Aldehyde2	1.582	129.03	-0.744	1.55	13.71	9.88
Aldehyde30	1.643	132.74	-0.638	1.59	12.94	10.14
Aldehyde430	6.713	179.94	0.000	2.56	23.49	7.97
Aldehyde50	1.600	140.28	-0.651	1.23	6.07	11.83
Aldehyde671	1.722	134.42	-0.544	1.83	13.80	10.13
Amide0	1.600	142.71	-0.667	1.31	10.11	10.71
Amide1	1.600	144.28	-0.689	1.21	8.75	11.01
Amide2	1.585	129.25	-0.735	1.44	13.37	9.87
Amide30	1.600	141.82	-0.593	1.03	7.39	11.24
Amide3V2	1.564	129.38	-0.759	1.44	10.43	10.74
Amide430	4.404	179.56	0.000	2.87	30.00	6.34
Amide4V2	1.579	128.88	-0.749	1.75	14.71	9.78
Amide50	1.600	138.20	-0.641	1.01	2.94	12.55
Amide5V2	1.583	128.57	-0.755	1.20	6.56	11.65
Amide671	4.040	179.73	0.000	2.93	30.21	6.33
Amide6V2	1.582	128.98	-0.745	0.74	2.72	12.34
Amide7V2	1.609	132.83	-0.710	1.10	3.29	12.53
Amide8V2	1.607	132.69	-0.709	1.19	7.20	11.46
Amine0	1.600	143.64	-0.703	1.34	13.51	9.72
Amine1	1.583	128.76	-0.754	1.57	15.71	9.29
Amine2	1.600	143.67	-0.702	1.73	17.38	8.96
Amine30	1.600	143.69	-0.685	1.51	15.30	9.36
Amine3V2	1.574	129.09	-0.753	1.40	11.02	10.53
Amine430	1.692	131.41	-0.594	2.12	15.91	9.79
Amine4V2	1.576	128.99	-0.751	1.34	12.37	10.07
Amine50	1.600	141.19	-0.673	1.25	8.98	10.98
Amine5V2	1.581	128.94	-0.747	1.43	12.08	10.24
Amine671	1.686	130.51	-0.612	1.84	11.40	10.85
Amine6V2	1.569	129.27	-0.755	1.38	10.69	10.61
Amine7V2	1.603	133.10	-0.712	1.13	5.71	11.84
Amine8V2	1.601	132.24	-0.717	1.07	5.66	11.79
Anhydride0	1.603	130.26	-0.704	1.29	9.92	10.75
Anhydride1	1.572	129.83	-0.744	1.37	7.98	11.41
Anhydride2	1.572	129.37	-0.749	1.49	9.50	11.07

Anhydride30	3.840	179.88	0.000	2.96	36.27	4.56
Anhydride3V2	1.585	129.14	-0.739	1.35	10.29	10.70
Anhydride430	8.478	179.88	0.000	3.02	34.25	5.23
Anhydride4V2	1.578	129.01	-0.747	1.39	10.99	10.53
Anhydride50	1.703	134.95	-0.551	1.74	9.92	11.20
Anhydride5V2	1.581	129.07	-0.748	1.29	8.96	11.03
Anhydride671	4.660	179.78	0.000	2.93	31.16	6.05
Anhydride7V2	1.580	128.85	-0.750	0.89	2.97	12.42
Anhydride8V2	1.581	128.92	-0.748	1.27	12.99	9.81
AzolidineBr1	1.600	142.81	-0.620	1.41	9.89	10.88
AzolidineBr2	1.600	141.65	-0.658	1.58	8.79	11.37
AzolidineC#N1	1.651	133.29	-0.605	1.81	14.09	10.03
AzolidineC#N2	1.622	131.08	-0.669	1.74	16.87	9.13
AzolidineCl1	1.641	132.64	-0.622	1.66	11.51	10.64
AzolidineCl2	1.600	141.79	-0.653	1.66	15.97	9.31
AzolidineF1	1.640	132.88	-0.617	1.66	10.59	10.92
AzolidineF2	1.600	131.84	-0.687	1.11	5.15	11.99
AzolidineI1	1.600	142.48	-0.626	1.39	11.00	10.52
AzolidineI2	1.618	130.90	-0.671	1.79	14.54	9.87
AzolidineN1	1.609	130.86	-0.674	1.85	15.86	9.54
AzolidineN2	1.600	141.24	-0.674	1.57	12.23	10.34
AzolidineNO2_1	1.600	142.12	-0.624	1.32	4.51	12.39
AzolidineNO2_2	1.600	143.17	-0.645	1.13	1.12	13.21
AzolidineO1	1.600	131.79	-0.673	1.01	6.47	11.50
AzolidineO2	1.600	142.81	-0.645	1.60	11.86	10.48
AzolidineS1	1.600	142.82	-0.626	1.32	6.20	11.89
AzolidineS2	1.618	131.16	-0.667	1.65	13.31	10.09
Br0	1.600	142.88	-0.676	1.22	11.56	10.19
Br1	1.588	129.99	-0.719	1.33	11.09	10.44
Br2	1.583	129.08	-0.742	1.55	14.14	9.75
Br3	5.093	179.38	0.000	2.49	26.04	7.14
Br4	1.600	142.47	-0.602	1.22	6.14	11.81
Br5	4.933	179.67	0.000	2.32	30.22	5.73
Br6	6.893	179.92	0.000	3.34	45.41	2.22
Cl0	1.600	145.41	-0.649	1.46	13.37	9.89
Cl1	1.600	129.01	-0.727	0.99	8.61	10.84
Cl2	1.582	129.08	-0.743	1.57	16.00	9.21

Cl3	1.600	143.75	-0.607	1.43	10.12	10.83
Cl4	1.600	143.28	-0.607	1.25	4.87	12.21
Cl5	6.428	179.93	0.000	3.04	41.50	3.08
Cl6	3.895	179.82	0.000	3.28	40.29	3.68
Cyan0	1.600	144.79	-0.659	1.67	14.95	9.62
Cyan1	1.600	142.83	-0.699	1.50	13.26	9.96
Cyan2	1.600	128.63	-0.740	1.46	13.18	9.94
Cyan30	2.239	167.11	-0.058	2.52	21.51	8.52
Cyan3V2	1.581	129.00	-0.745	1.57	15.71	9.30
Cyan430	6.251	179.91	0.000	3.49	43.00	3.08
Cyan4V2	1.583	128.76	-0.748	1.40	12.86	9.98
Cyan50	1.600	143.52	-0.635	1.50	9.54	11.07
Cyan5V2	1.571	129.11	-0.756	1.37	10.59	10.63
Cyan671	4.127	179.67	0.000	2.96	32.15	5.79
Cyan6V2	1.581	129.05	-0.746	1.42	10.41	10.73
Cyan7V2	1.581	128.84	-0.749	1.54	12.62	10.20
Cyan8V2	1.580	128.78	-0.749	1.59	15.44	9.40
Ester0	1.600	130.48	-0.702	1.22	9.10	10.93
Ester1	1.586	129.30	-0.735	1.64	14.16	9.83
Ester2	1.572	129.41	-0.749	1.23	8.46	11.12
Ester30	1.700	136.12	-0.542	1.90	14.51	9.98
Ester3V2	1.569	129.26	-0.756	1.26	9.31	10.90
Ester430	6.862	179.95	0.000	3.26	45.35	2.15
Ester4V2	1.582	128.88	-0.747	1.52	13.17	10.01
Ester50	1.600	137.28	-0.662	1.20	5.32	12.03
Ester5V2	1.579	128.80	-0.748	1.40	9.47	10.99
Ester671	2.323	169.84	-0.039	2.01	7.55	12.17
Ester6V2	1.607	133.00	-0.707	1.34	8.76	11.15
Ester7V2	1.603	133.04	-0.714	1.17	5.27	12.01
Ester8V2	1.607	133.82	-0.706	1.32	10.22	10.69
Ether0	1.600	129.31	-0.722	1.08	9.77	10.58
Ether1	1.581	128.93	-0.747	1.38	11.73	10.30
Ether2	1.571	129.12	-0.756	1.30	8.78	11.09
Ether30	1.650	132.49	-0.630	1.84	15.86	9.53
Ether3V2	1.579	128.91	-0.749	1.41	11.38	10.43
Ether430	5.846	179.91	0.000	3.83	49.42	1.51
Ether4V2	1.584	129.09	-0.744	1.50	13.30	9.95

Ether50	1.600	140.76	-0.649	1.27	7.37	11.49
Ether5V2	1.580	128.80	-0.751	1.27	8.78	11.07
Ether671	1.741	134.68	-0.525	2.48	20.51	8.78
Ether6V2	1.580	128.84	-0.749	1.56	14.58	9.63
Ether7V2	1.617	133.36	-0.699	1.26	6.06	11.86
Ether8V2	1.581	128.90	-0.748	1.46	12.90	10.03
F0	1.600	142.59	-0.672	1.06	10.39	10.38
F1	1.600	141.30	-0.707	1.16	10.20	10.53
F2	1.583	129.20	-0.733	1.18	9.26	10.84
F3	2.010	154.51	-0.176	2.11	16.76	9.52
F4	1.697	135.13	-0.550	1.87	16.08	9.49
F5	4.067	179.85	0.000	2.76	34.63	4.85
F6	5.051	179.59	0.000	3.15	37.24	4.46
Halid2V2	1.583	129.28	-0.731	1.21	9.58	10.77
Halid3V2	1.582	128.96	-0.745	1.55	15.32	9.39
Halid4V2	1.579	128.85	-0.759	1.42	13.09	9.93
Halid5V2	1.580	128.87	-0.750	1.28	9.95	10.73
Halid6V2	1.580	128.89	-0.749	1.55	13.38	9.98
Halid7V2	1.580	128.85	-0.750	1.54	13.42	9.95
Halid8V2	1.606	134.39	-0.703	1.33	9.02	11.06
I0	1.600	142.70	-0.678	1.12	8.49	11.00
I1	1.600	130.96	-0.706	1.36	13.17	9.84
I2	1.583	129.11	-0.741	1.53	13.84	9.82
I3	5.365	179.79	0.000	2.84	33.64	5.23
I4	4.914	179.49	0.000	2.84	32.85	5.46
I5	8.021	179.82	0.000	3.25	44.89	2.28
I6	4.675	179.45	0.000	3.28	41.93	3.20
ImidazoleBr1	5.228	179.81	0.000	3.07	38.62	3.97
ImidazoleC#N1	4.835	179.49	0.000	2.95	36.42	4.50
ImidazoleC#N2	5.655	179.92	0.000	2.92	33.39	5.37
ImidazoleF1	5.643	179.94	0.000	2.90	34.42	5.05
ImidazoleF2	5.807	179.84	0.000	2.99	36.39	4.55
ImidazoleI1	5.128	179.89	0.000	2.88	31.62	5.87
ImidazoleN1	5.758	179.74	0.000	2.84	32.80	5.47
ImidazoleN2	5.951	179.88	0.000	3.02	37.13	4.36
ImidazoleNO2_1	5.460	179.94	0.000	2.99	34.69	5.06
ImidazoleNO2_2	4.703	179.46	0.000	2.84	30.83	6.06

ImidazoleO1	4.861	179.73	0.000	2.69	28.44	6.63
ImidazoleO2	7.040	179.94	0.000	2.93	34.83	4.96
ImidazoleS1	5.828	179.23	0.000	3.00	35.41	4.86
ImidazoleS2	4.775	179.67	0.000	2.86	30.87	6.07
Ketone3V2	1.582	128.96	-0.746	1.48	12.21	10.26
Ketone4V2	1.573	129.19	-0.752	1.63	14.18	9.81
Ketone5V2	1.582	128.52	-0.757	1.25	9.10	10.95
Ketone6V2	1.580	128.94	-0.748	1.35	12.71	9.98
Ketone7V2	1.580	128.90	-0.748	1.46	10.40	10.77
Ketone8V2	1.605	133.52	-0.709	1.43	11.71	10.35
Nitro0	1.600	130.50	-0.705	1.09	8.50	10.97
Nitro1	1.593	129.61	-0.727	1.60	14.94	9.56
Nitro3	1.600	128.94	-0.740	1.17	9.57	10.73
Nitro30	1.704	135.99	-0.548	1.82	9.45	11.42
Nitro430	4.385	179.42	0.000	3.11	37.66	4.30
Nitro4V2	1.600	128.98	-0.733	1.16	11.22	10.23
Nitro50	1.662	133.44	-0.611	1.65	9.55	11.21
Nitro5V2	1.580	128.76	-0.751	1.65	14.98	9.60
Nitro671	5.546	179.80	0.000	3.42	41.90	3.34
Nitro6V2	1.583	128.62	-0.748	1.41	6.45	11.90
Nitro7V2	1.608	134.65	-0.699	1.31	7.59	11.47
Nitro8V2	1.580	128.86	-0.750	1.49	12.24	10.25
PiperidineBr1	1.665	132.37	-0.608	1.82	12.49	10.51
PiperidineBr2	1.624	130.70	-0.672	1.83	13.80	10.13
PiperidineBr3	1.619	130.07	-0.683	1.76	12.86	10.34
PiperidineC#N1	1.669	134.33	-0.586	1.85	13.31	10.29
PiperidineC#N2	1.627	130.57	-0.669	1.84	15.21	9.72
PiperidineC#N3	1.623	131.48	-0.662	1.73	12.76	10.34
PiperidineCl1	1.600	141.54	-0.643	1.50	10.56	10.77
PiperidineCl2	1.600	142.08	-0.642	1.71	13.61	10.07
PiperidineCl3	1.617	130.09	-0.685	1.84	16.31	9.39
PiperidineF1	1.600	134.86	-0.653	1.53	11.13	10.63
PiperidineF2	1.626	130.46	-0.672	1.83	14.62	9.89
PiperidineF3	1.600	141.35	-0.652	1.62	14.84	9.61
PiperidineI1	1.711	135.67	-0.541	2.04	15.59	9.81
PiperidineI2	1.600	142.17	-0.637	1.84	13.63	10.19
PiperidineI3	1.626	131.63	-0.658	1.93	22.31	7.69

PiperidineN1	1.600	132.27	-0.683	1.72	13.95	9.97
PiperidineN2	1.619	131.03	-0.672	1.95	16.25	9.52
PiperidineN3	1.602	131.14	-0.685	1.19	8.12	11.19
PiperidineNO2_1	1.600	141.77	-0.625	1.80	13.17	10.29
PiperidineNO2_2	1.600	142.31	-0.650	1.59	11.46	10.59
PiperidineNO2_3	1.600	140.74	-0.657	1.77	15.18	9.66
PiperidineO1	1.600	132.71	-0.653	1.38	10.46	10.67
PiperidineO2	1.617	131.31	-0.670	1.82	14.28	9.97
PiperidineO3	1.600	133.74	-0.679	1.42	10.49	10.71
PiperidineS1	1.650	131.25	-0.637	1.85	13.45	10.25
PiperidineS2	1.600	132.68	-0.696	1.67	12.32	10.41
PiperidineS3	1.621	130.09	-0.679	1.83	13.73	10.15
PyrazineBr1	1.631	132.30	-0.579	1.88	15.23	9.76
PyrazineBr2	1.612	131.61	-0.603	1.96	15.72	9.69
PyrazineC#N1	4.206	179.75	0.000	3.00	35.98	4.69
PyrazineC#N2	1.619	132.93	-0.584	1.90	14.18	10.09
PyrazineCl1	1.612	133.15	-0.592	2.03	16.09	9.64
PyrazineCl2	1.606	131.05	-0.617	1.92	15.37	9.75
PyrazineF1	3.948	179.90	0.000	2.94	33.09	5.49
PyrazineF2	1.619	131.82	-0.596	1.94	15.37	9.77
PyrazineI1	1.601	132.25	-0.614	1.83	13.65	10.17
PyrazineI2	1.603	130.91	-0.620	1.94	15.32	9.79
PyrazineN1	1.529	126.10	-0.720	1.73	15.44	9.54
PyrazineN2	1.579	128.82	-0.679	1.89	14.69	9.92
PyrazineNO2_1	4.994	179.91	0.000	3.00	35.10	4.95
PyrazineNO2_2	1.708	137.01	-0.466	2.26	18.40	9.19
PyrazineO1	1.613	133.38	-0.585	2.05	16.48	9.55
PyrazineO2	1.595	130.13	-0.642	1.87	14.97	9.82
PyrazineS1	1.651	135.12	-0.541	2.15	16.50	9.64
PyrazineS2	1.611	131.08	-0.613	1.92	15.03	9.85
PyrazoleBr1	5.899	179.97	0.000	3.03	38.20	4.06
PyrazoleBr2	4.356	179.45	0.000	2.80	27.63	6.97
PyrazoleC#N1	5.406	179.95	0.000	2.94	34.62	5.03
PyrazoleC#N2	5.004	179.66	0.000	3.00	37.11	4.35
PyrazoleCl1	5.844	179.94	0.000	2.93	33.10	5.47
PyrazoleCl2	5.013	179.67	0.000	2.94	34.01	5.22
PyrazoleF1	5.708	179.99	0.000	3.03	39.86	3.56

PyrazoleF2	5.627	179.86	0.000	2.99	37.11	4.34
PyrazoleI1	6.385	179.97	0.000	3.06	41.19	3.19
PyrazoleI2	5.636	179.81	0.000	2.99	38.15	4.03
PyrazoleN1	1.608	129.91	-0.703	1.72	13.93	9.98
PyrazoleN2	1.685	134.98	-0.563	1.88	16.59	9.34
PyrazoleNO2_1	5.334	179.74	0.000	2.82	32.46	5.55
PyrazoleNO2_2	5.684	179.92	0.000	2.86	32.72	5.52
PyrazoleO1	5.839	179.85	0.000	3.02	39.64	3.62
PyrazoleO2	1.600	140.38	-0.617	1.40	10.78	10.59
PyrazoleS1	5.996	179.92	0.000	2.98	35.12	4.92
PyrazoleS2	1.600	140.30	-0.599	1.58	10.52	10.86
PyridineBr1	1.582	130.89	-0.648	1.74	12.08	10.55
PyridineBr2	1.579	129.20	-0.665	1.93	17.84	9.02
PyridineBr3	1.581	129.12	-0.666	1.89	14.59	9.95
PyridineC#N1	1.604	132.09	-0.613	1.85	13.52	10.23
PyridineC#N2	1.588	129.70	-0.650	1.90	15.57	9.68
PyridineC#N3	1.584	129.43	-0.658	1.87	15.04	9.80
PyridineCl1	1.581	128.15	-0.671	1.85	14.24	10.01
PyridineCl2	1.577	128.95	-0.673	1.88	14.78	9.88
PyridineCl3	1.572	128.64	-0.680	1.87	14.45	9.98
PyridineF1	1.599	130.04	-0.623	1.93	14.82	9.92
PyridineF2	1.582	129.26	-0.663	1.85	14.50	9.94
PyridineF3	1.577	128.97	-0.669	1.88	15.58	9.65
PyridineI1	1.590	128.36	-0.662	1.81	12.95	10.36
PyridineI2	1.575	128.87	-0.675	1.92	15.85	9.60
PyridineI3	1.575	128.73	-0.677	1.90	15.05	9.83
PyridineN1	1.533	125.59	-0.729	1.41	10.87	10.58
PyridineN2	1.566	128.03	-0.702	1.81	14.07	10.03
PyridineN3	1.535	126.33	-0.749	1.88	13.85	10.16
PyridineNO2_1	1.618	133.14	-0.590	2.02	14.01	10.26
PyridineNO2_2	1.603	130.69	-0.620	1.97	15.34	9.81
PyridineNO2_3	1.599	130.40	-0.633	1.89	13.93	10.16
PyridineO1	1.552	127.49	-0.665	1.61	13.70	9.94
PyridineO2	1.570	128.18	-0.694	1.88	15.78	9.59
PyridineO3	1.558	127.77	-0.704	1.79	15.51	9.58
PyridineS1	1.581	128.95	-0.646	1.55	11.58	10.51
PyridineS2	1.576	128.83	-0.675	1.80	13.19	10.28

PyridineS3	1.568	128.42	-0.687	1.88	17.35	9.13
PyrimidineBr1	1.622	131.99	-0.583	1.87	14.96	9.82
PyrimidineBr2	1.601	132.53	-0.597	1.92	14.43	10.03
PyrimidineBr3	1.608	131.59	-0.600	1.98	17.05	9.31
PyrimidineC#N1	1.629	132.30	-0.566	2.00	16.42	9.52
PyrimidineC#N2	3.716	179.83	0.000	2.99	35.31	4.88
PyrimidineC#N3	1.622	132.46	-0.577	2.01	16.88	9.39
PyrimidineCl1	1.604	130.33	-0.614	1.90	15.74	9.62
PyrimidineCl2	1.622	132.31	-0.583	1.76	12.21	10.54
PyrimidineCl3	1.602	131.13	-0.612	1.94	16.82	9.34
PyrimidineF1	1.643	133.57	-0.537	2.06	17.36	9.29
PyrimidineF2	3.789	179.97	0.000	2.91	32.30	5.70
PyrimidineF3	1.614	131.81	-0.593	1.96	17.03	9.30
PyrimidineI1	1.598	132.37	-0.609	1.77	14.26	9.93
PyrimidineI2	1.619	132.35	-0.589	1.81	13.00	10.35
PyrimidineI3	1.598	130.96	-0.616	1.96	16.78	9.37
PyrimidineN1	1.530	126.55	-0.710	1.79	14.90	9.76
PyrimidineN2	1.532	126.42	-0.707	1.74	16.05	9.37
PyrimidineN3	1.579	129.42	-0.661	1.78	14.12	9.98
PyrimidineNO2_1	6.136	179.97	0.000	2.78	29.71	6.34
PyrimidineNO2_2	4.030	179.67	0.000	2.91	29.29	6.59
PyrimidineNO2_3	1.717	137.75	-0.447	2.31	21.51	8.31
PyrimidineO1	1.595	129.66	-0.619	2.00	17.17	9.29
PyrimidineO2	1.563	129.16	-0.622	1.64	13.42	10.05
PyrimidineO3	1.591	130.24	-0.637	1.85	15.03	9.78
PyrimidineS1	1.639	134.52	-0.551	1.88	13.05	10.40
PyrimidineS2	1.605	128.85	-0.598	1.70	13.39	10.13
PyrimidineS3	1.600	131.01	-0.617	1.88	14.40	9.99
PyrroleBr2	4.359	179.19	0.000	3.02	35.93	4.73
PyrroleC#N1	5.385	179.87	0.000	2.89	33.61	5.28
PyrroleC#N2	2.009	154.52	-0.167	2.26	18.75	9.08
PyrroleCl1	5.508	179.97	0.000	3.03	38.91	3.85
PyrroleCl2	1.676	134.09	-0.578	2.02	17.80	9.13
PyrroleF1	4.823	179.53	0.000	3.00	36.23	4.62
PyrroleF2	4.640	179.57	0.000	2.97	40.59	3.28
PyrroleI1	5.127	179.86	0.000	2.99	35.73	4.75
PyrroleI2	1.675	134.09	-0.578	2.03	17.78	9.14

PyrroleN1	1.663	133.34	-0.599	1.77	14.44	9.88
PyrroleN2	1.619	130.77	-0.677	1.69	13.23	10.16
PyrroleNO2_1	5.368	179.95	0.000	2.91	31.83	5.84
PyrroleNO2_2	5.324	179.94	0.000	2.76	27.48	6.98
PyrroleO1	1.637	132.36	-0.602	1.26	9.89	10.72
PyrroleO2	1.600	134.01	-0.666	1.33	10.75	10.54
PyrroleS1	5.322	179.88	0.000	2.98	37.45	4.22
PyrroleS2	1.672	133.88	-0.584	1.86	14.08	10.08
ThiazoleBr1	1.559	130.23	-0.657	1.86	16.27	9.42
ThiazoleBr2	1.558	130.02	-0.659	1.94	17.42	9.16
ThiazoleC#N1	1.573	130.47	-0.634	1.86	14.56	9.93
ThiazoleC#N2	1.567	130.54	-0.641	1.89	15.13	9.79
ThiazoleC#N3	1.576	130.43	-0.630	1.93	16.75	9.35
ThiazoleCl1	1.552	129.64	-0.670	1.86	13.93	10.12
ThiazoleCl2	1.555	129.75	-0.667	1.91	16.27	9.48
ThiazoleCl3	1.556	129.38	-0.665	1.91	19.55	8.49
ThiazoleF1	1.573	130.56	-0.626	1.89	14.82	9.88
ThiazoleF2	1.560	130.18	-0.654	1.90	16.26	9.46
ThiazoleF3	1.567	130.61	-0.632	1.88	15.33	9.72
ThiazoleI1	1.549	129.48	-0.679	1.73	11.56	10.70
ThiazoleI2	1.551	129.59	-0.672	1.93	16.44	9.44
ThiazoleI3	1.558	129.88	-0.664	1.81	13.34	10.25
ThiazoleN1	1.500	126.44	-0.739	1.50	11.78	10.40
ThiazoleN2	1.543	128.54	-0.702	1.79	14.61	9.85
ThiazoleN3	1.539	128.73	-0.676	1.57	11.71	10.49
ThiazoleNO2_1	1.588	131.88	-0.612	2.01	17.04	9.35
ThiazoleNO2_2	1.581	131.54	-0.612	1.99	15.66	9.73
ThiazoleNO2_3	1.585	132.08	-0.609	1.66	10.95	10.81
ThiazoleO1	1.551	128.22	-0.671	1.82	14.56	9.89
ThiazoleO2	1.550	129.19	-0.683	1.82	15.51	9.61
ThiazoleO3	1.559	129.00	-0.660	1.74	14.39	9.87
ThiazoleS1	1.567	128.28	-0.657	1.79	13.47	10.18
ThiazoleS2	1.556	129.80	-0.665	1.77	12.59	10.43
ThiazoleS3	1.588	132.13	-0.611	1.98	15.91	9.65
Tio0	1.586	129.29	-0.731	1.36	12.04	10.19
Tio1	1.573	129.26	-0.751	1.65	15.81	9.35
Tio2	1.581	128.95	-0.746	1.61	17.79	8.72

Tio30	1.660	133.44	-0.609	1.85	15.08	9.77
Tio3V2	1.581	128.99	-0.746	1.41	9.89	10.87
Tio430	5.244	179.47	0.000	2.87	37.74	4.03
Tio4V2	1.577	128.87	-0.750	1.52	11.88	10.40
Tio50	1.651	132.75	-0.621	1.41	10.37	10.74
Tio5V2	1.579	128.84	-0.751	1.39	10.80	10.59
Tio671	1.709	132.79	-0.569	1.95	13.33	10.39
Tio6V2	1.580	128.92	-0.748	1.52	9.94	10.97
Tio7V2	1.610	131.36	-0.710	1.49	8.24	11.45
Tio8V2	1.601	132.38	-0.716	1.20	8.41	11.11
TriazoleBr1	5.073	179.76	0.000	2.91	31.88	5.82
TriazoleBr2	5.153	179.49	0.000	2.33	31.05	5.49
TriazoleC#N1	4.940	179.94	0.000	2.90	32.29	5.69
TriazoleC#N2	5.357	179.98	0.000	1.79	21.76	7.72
TriazoleCl1	5.729	179.93	0.000	2.98	36.81	4.42
TriazoleCl2	5.860	179.94	0.000	2.06	26.74	6.50
TriazoleF1	5.409	179.92	0.000	3.00	37.28	4.29
TriazoleF2	5.739	179.85	0.000	2.26	34.91	4.27
TriazoleI1	5.473	179.95	0.000	3.01	36.54	4.53
TriazoleI2	5.938	179.92	0.000	2.41	32.05	5.26
TriazoleN1	5.398	179.89	0.000	2.80	31.31	5.88
TriazoleN2	5.680	179.93	0.000	2.25	28.42	6.20
TriazoleNO2_1	5.382	179.88	0.000	2.99	37.15	4.33
TriazoleNO2_2	5.002	179.75	0.000	1.98	20.90	8.16
TriazoleO1	5.165	179.86	0.000	3.04	40.33	3.43
TriazoleO2	5.660	179.81	0.000	2.67	26.98	7.04
TriazoleS1	5.936	179.98	0.000	3.18	39.66	3.76
TriazoleS2	5.806	179.93	0.000	1.81	18.39	8.74

Table S10: Parameters calculated using the PM6 method.

Name	R (Å)	θ (°)	ΔQ	ΔH (kcal/mol)	ΔS (cal/molK)	ΔG (kcal/mol)
Acid0	1.597	136.44	-0.523	1.87	16.59	9.32
Acid1	1.582	135.09	-0.555	1.41	11.35	10.43
Acid2	1.577	134.20	-0.574	1.52	15.19	9.40
Acid30	1.648	139.59	-0.443	1.68	12.22	10.44
Acid3V2	1.576	134.36	-0.575	1.61	16.84	8.99

Acid430	1.754	145.67	-0.341	1.97	11.74	10.87
Acid4V2	1.575	134.24	-0.578	1.54	15.56	9.31
Acid50	1.611	137.32	-0.470	1.55	12.71	10.16
Acid671	1.633	136.26	-0.509	1.89	9.20	11.55
Acid6V2	1.577	134.36	-0.573	1.29	8.73	11.09
Acid7V2	1.574	134.14	-0.581	1.10	7.99	11.12
Acid8V2	1.576	134.37	-0.577	1.29	6.00	11.90
AcidBr1	1.587	135.33	-0.549	1.25	9.24	10.89
AcidBr2	1.579	134.57	-0.568	1.60	14.80	9.59
AcidBr3	1.665	141.49	-0.426	1.59	12.19	10.36
AcidBr4	1.655	140.15	-0.449	1.55	11.09	10.64
AcidBr5	3.492	179.66	0.000	3.14	30.21	6.54
AcidBr6	1.660	138.24	-0.467	1.91	12.43	10.60
AcidCl0	1.601	136.90	-0.511	1.40	11.92	10.24
AcidCl1	1.583	135.42	-0.551	1.48	14.80	9.46
AcidCl2	1.574	134.80	-0.569	1.47	14.46	9.55
AcidCl3	1.661	141.25	-0.432	1.66	15.12	9.55
AcidCl4	1.638	138.98	-0.470	1.49	11.58	10.44
AcidCl5	3.236	179.72	0.000	2.75	23.37	8.18
AcidCl6	1.641	137.00	-0.489	1.42	4.62	12.44
AcidF0	1.602	137.01	-0.508	1.52	13.61	9.86
AcidF1	1.588	135.18	-0.548	1.54	17.32	8.77
AcidF2	1.578	134.50	-0.569	1.70	18.90	8.47
AcidF3	1.671	141.83	-0.418	1.89	15.33	9.73
AcidF4	1.638	138.84	-0.473	1.58	12.42	10.28
AcidF5	3.208	179.67	0.000	2.79	24.44	7.90
AcidF6	1.642	136.95	-0.489	1.97	15.84	9.64
AcidI0	1.600	136.78	-0.512	1.70	14.32	9.83
AcidI1	1.586	135.31	-0.550	1.62	14.60	9.67
AcidI2	1.574	134.91	-0.569	1.33	11.43	10.32
AcidI3	1.660	140.76	-0.434	1.82	16.18	9.40
AcidI4	1.640	139.21	-0.468	1.22	1.80	13.09
AcidI5	3.132	179.89	0.000	2.78	19.01	9.51
AcidI6	1.662	137.83	-0.478	1.34	3.37	12.73
Acid_Halid3V2	1.576	134.32	-0.575	1.49	12.39	10.20
Acid_Halid4V2	1.575	134.24	-0.577	1.59	14.90	9.54
Acid_Halid5V2	1.575	134.29	-0.579	1.36	11.78	10.25

Acid_Halid6V2	1.567	134.40	-0.584	1.35	11.89	10.21
Acid_Halid7V2	1.574	134.19	-0.579	1.66	18.15	8.65
Acid_Halid8V2	1.576	134.36	-0.577	1.44	12.85	10.01
Acid_Sulf0	1.584	135.10	-0.557	1.62	14.54	9.69
Acid_Sulf1	1.579	134.25	-0.574	1.54	11.49	10.51
Acid_Sulf2	1.577	134.21	-0.574	1.74	15.40	9.54
Acid_Sulf30	1.614	136.64	-0.517	1.59	13.62	9.93
Acid_Sulf3V2	1.569	134.47	-0.582	1.31	10.59	10.56
Acid_Sulf430	1.639	136.45	-0.490	1.48	6.41	11.97
Acid_Sulf4V2	1.575	134.23	-0.578	1.61	18.98	8.35
Acid_Sulf50	1.600	136.92	-0.542	1.64	12.22	10.40
Acid_Sulf5V2	1.574	134.09	-0.581	1.61	12.72	10.22
Acid_Sulf671	1.626	135.38	-0.531	1.60	7.89	11.65
Acid_Sulf6V2	1.574	134.16	-0.579	1.52	13.49	9.90
Acid_Sulf7V2	1.574	134.24	-0.580	1.05	7.40	11.24
Acid_Sulf8V2	1.575	134.18	-0.579	1.69	16.38	9.21
Acid_Tio0	1.600	135.64	-0.527	1.28	8.28	11.21
Acid_Tio1	1.580	134.96	-0.562	1.19	8.98	10.91
Acid_Tio2	1.600	134.49	-0.561	1.22	10.92	10.37
Acid_Tio30	1.638	139.38	-0.470	1.79	10.59	11.03
Acid_Tio3V2	1.575	134.19	-0.578	1.74	17.17	9.02
Acid_Tio430	1.699	141.74	-0.406	2.31	14.57	10.37
Acid_Tio4V2	1.575	134.20	-0.579	1.84	15.77	9.54
Acid_Tio50	1.605	135.59	-0.523	1.44	7.10	11.72
Acid_Tio5V2	1.573	134.11	-0.581	1.52	14.84	9.50
Acid_Tio671	1.605	133.08	-0.540	1.42	6.96	11.75
Acid_Tio6V2	1.569	134.09	-0.584	1.40	15.03	9.31
Acid_Tio7V2	1.573	134.05	-0.583	1.38	15.78	9.07
Acid_Tio8V2	1.576	134.31	-0.577	1.50	9.00	11.21
Alcohol0	1.578	134.55	-0.568	1.31	10.01	10.73
Alcohol1	1.575	134.25	-0.575	1.35	12.00	10.17
Alcohol2	1.574	134.24	-0.578	1.38	10.42	10.67
Alcohol30	1.591	134.80	-0.562	1.53	13.44	9.92
Alcohol3V2	1.574	134.18	-0.579	1.47	15.06	9.38
Alcohol430	1.624	134.99	-0.533	1.96	19.00	8.70
Alcohol4V2	1.568	134.44	-0.583	1.18	8.63	11.01
Alcohol50	1.600	136.43	-0.562	1.07	6.22	11.62

Alcohol5V2	1.574	134.10	-0.579	1.36	13.64	9.70
Alcohol671	1.600	132.58	-0.566	1.34	6.05	11.94
Alcohol6V2	1.574	134.18	-0.579	1.66	15.90	9.32
Alcohol7V2	1.574	134.17	-0.580	1.43	10.24	10.78
Alcohol8V2	1.574	134.05	-0.580	1.54	11.50	10.51
Aldehyde0	1.585	135.26	-0.551	1.39	12.66	10.01
Aldehyde1	1.575	134.43	-0.570	1.20	9.30	10.83
Aldehyde2	1.575	134.31	-0.576	1.66	16.46	9.16
Aldehyde30	1.615	136.84	-0.518	1.41	8.34	11.32
Aldehyde430	1.642	137.23	-0.496	1.42	6.40	11.91
Aldehyde50	1.600	137.54	-0.543	1.04	3.93	12.27
Aldehyde671	1.621	135.39	-0.538	1.97	14.17	10.14
Amide0	1.588	135.44	-0.542	1.28	10.88	10.44
Amide1	1.578	134.28	-0.571	1.51	13.49	9.89
Amide2	1.577	134.41	-0.573	1.75	20.38	8.08
Amide30	1.633	138.47	-0.479	1.71	14.79	9.70
Amide3V2	1.575	134.17	-0.578	1.44	13.03	9.96
Amide430	1.680	139.93	-0.435	1.72	8.24	11.66
Amide4V2	1.576	134.08	-0.578	1.59	13.47	9.97
Amide50	1.616	137.15	-0.511	1.62	12.85	10.19
Amide5V2	1.572	133.89	-0.586	0.95	5.26	11.79
Amide671	1.641	135.92	-0.517	1.47	6.89	11.82
Amide6V2	1.567	135.04	-0.583	1.05	5.76	11.74
Amide7V2	1.576	134.28	-0.577	1.41	9.18	11.08
Amide8V2	1.574	134.25	-0.579	1.47	11.39	10.47
Amine0	1.576	134.12	-0.577	1.68	16.62	9.12
Amine1	1.574	134.22	-0.583	1.46	13.90	9.72
Amine2	1.573	134.14	-0.583	1.41	14.39	9.52
Amine30	1.576	134.45	-0.576	1.61	16.58	9.06
Amine3V2	1.567	134.50	-0.585	1.16	3.79	12.43
Amine430	1.609	133.89	-0.559	1.48	9.61	11.01
Amine4V2	1.567	134.40	-0.585	1.42	12.89	9.98
Amine50	1.586	134.07	-0.574	1.62	11.81	10.50
Amine5V2	1.574	134.08	-0.580	1.34	11.27	10.38
Amine671	1.600	132.39	-0.578	1.82	15.67	9.55
Amine6V2	1.566	134.52	-0.585	1.51	13.06	10.02
Amine7V2	1.575	134.09	-0.579	1.44	6.68	11.85

Amine8V2	1.574	134.15	-0.580	1.73	19.64	8.28
Anhydride0	1.600	136.26	-0.520	1.36	10.17	10.73
Anhydride1	1.585	135.11	-0.554	1.62	14.63	9.66
Anhydride2	1.575	134.78	-0.568	1.44	12.74	10.04
Anhydride30	1.663	140.99	-0.427	1.71	10.37	11.02
Anhydride3V2	1.576	134.29	-0.573	1.37	12.14	10.15
Anhydride430	3.179	179.60	0.000	3.06	29.58	6.65
Anhydride4V2	1.574	134.26	-0.578	1.80	16.72	9.21
Anhydride50	1.619	136.18	-0.511	1.41	7.29	11.64
Anhydride5V2	1.573	134.13	-0.580	1.28	8.67	11.09
Anhydride671	1.635	136.30	-0.500	1.76	9.11	11.45
Anhydride7V2	1.574	134.20	-0.580	1.48	13.72	9.79
Anhydride8V2	1.574	134.15	-0.581	1.41	7.39	11.61
AzolidineBr1	1.600	136.32	-0.525	1.38	9.97	10.81
AzolidineBr2	1.590	134.79	-0.560	1.55	11.33	10.58
AzolidineC#N1	1.600	138.60	-0.516	1.55	11.92	10.40
AzolidineC#N2	1.600	137.08	-0.555	1.59	13.84	9.87
AzolidineCl1	1.600	136.70	-0.520	0.69	1.84	12.54
AzolidineCl2	1.600	135.39	-0.547	1.29	9.37	10.90
AzolidineF1	1.596	136.27	-0.529	1.61	11.68	10.52
AzolidineF2	1.590	134.70	-0.564	1.80	19.28	8.46
AzolidineI1	1.600	136.60	-0.531	1.02	3.10	12.50
AzolidineI2	1.587	134.70	-0.562	1.74	16.65	9.17
AzolidineN1	1.575	134.00	-0.579	1.68	15.25	9.53
AzolidineN2	1.583	134.02	-0.578	1.57	12.86	10.14
AzolidineNO2_1	1.600	137.26	-0.518	1.58	12.66	10.21
AzolidineNO2_2	1.600	135.96	-0.539	1.39	10.99	10.51
AzolidineO1	1.581	134.96	-0.557	1.50	11.61	10.44
AzolidineO2	1.590	135.15	-0.555	1.51	13.49	9.89
AzolidineS1	1.600	135.14	-0.549	1.26	7.16	11.52
AzolidineS2	1.586	134.79	-0.560	1.56	11.31	10.59
Br0	1.602	136.41	-0.538	1.26	12.97	9.80
Br1	1.583	134.94	-0.560	1.25	11.34	10.27
Br2	1.574	134.79	-0.571	1.56	16.51	9.04
Br3	1.663	139.91	-0.456	1.60	13.75	9.90
Br4	1.639	138.36	-0.486	1.53	10.69	10.74
Br5	3.429	178.81	0.000	3.35	36.19	4.96

Br6	1.627	135.84	-0.516	1.58	7.07	11.87
Cl0	1.604	136.90	-0.514	1.06	9.70	10.57
Cl1	1.580	134.90	-0.566	1.37	14.59	9.42
Cl2	1.578	134.57	-0.569	1.46	12.78	10.05
Cl3	1.651	139.79	-0.451	1.53	11.66	10.46
Cl4	1.630	138.60	-0.483	1.54	11.58	10.49
Cl5	3.325	179.22	-0.007	2.99	35.72	4.74
Cl6	1.643	137.27	-0.496	1.72	11.83	10.59
Cyan0	1.601	136.88	-0.515	1.27	12.33	9.99
Cyan1	1.581	134.43	-0.563	1.34	12.65	9.96
Cyan2	1.576	134.16	-0.576	1.53	16.35	9.05
Cyan30	1.642	139.63	-0.463	1.60	10.14	10.98
Cyan3V2	1.575	134.26	-0.576	1.42	12.55	10.08
Cyan430	1.721	143.18	-0.368	2.00	15.69	9.73
Cyan4V2	1.569	134.38	-0.582	1.40	18.17	8.39
Cyan50	1.618	137.84	-0.503	1.62	12.34	10.34
Cyan5V2	1.568	134.59	-0.583	1.21	10.89	10.37
Cyan671	1.632	136.14	-0.504	1.75	8.84	11.51
Cyan6V2	1.575	134.28	-0.578	1.60	13.92	9.85
Cyan7V2	1.574	134.17	-0.580	1.45	11.74	10.35
Cyan8V2	1.574	134.25	-0.579	1.56	14.07	9.76
Ester0	1.600	135.37	-0.526	1.71	13.94	9.95
Ester1	1.583	135.11	-0.558	1.11	6.77	11.50
Ester2	1.578	134.57	-0.568	1.60	11.16	10.67
Ester30	1.638	138.84	-0.469	1.83	14.93	9.78
Ester3V2	1.577	134.29	-0.575	1.41	12.08	10.21
Ester430	1.709	141.59	-0.407	1.67	8.35	11.58
Ester4V2	1.576	134.20	-0.577	1.30	8.13	11.27
Ester50	1.600	136.43	-0.537	1.37	10.94	10.51
Ester5V2	1.573	134.13	-0.579	1.43	14.31	9.56
Ester671	1.634	136.30	-0.510	1.53	5.18	12.39
Ester6V2	1.575	134.36	-0.577	1.56	13.91	9.81
Ester7V2	1.574	134.14	-0.580	1.38	11.48	10.36
Ester8V2	1.574	134.17	-0.580	1.56	16.22	9.13
Ether0	1.600	135.90	-0.525	1.33	11.77	10.22
Ether1	1.576	134.56	-0.571	1.11	8.35	11.02
Ether2	1.570	134.64	-0.578	1.37	7.91	11.42

Ether30	1.609	136.26	-0.522	1.63	11.50	10.60
Ether3V2	1.575	134.03	-0.579	1.33	9.20	10.99
Ether430	1.629	135.39	-0.512	1.69	11.51	10.66
Ether4V2	1.569	134.19	-0.583	1.64	17.94	8.69
Ether50	1.600	136.39	-0.538	1.42	9.33	11.04
Ether5V2	1.568	134.49	-0.584	1.28	13.65	9.61
Ether671	1.623	135.47	-0.535	1.84	13.04	10.35
Ether6V2	1.573	134.11	-0.580	1.46	11.70	10.38
Ether7V2	1.575	134.18	-0.579	1.52	13.96	9.76
Ether8V2	1.575	134.24	-0.577	1.30	9.59	10.84
F1	1.583	134.66	-0.568	1.32	11.84	10.19
F2	1.577	134.43	-0.572	1.47	14.08	9.68
F3	1.660	140.43	-0.434	1.83	14.72	9.84
F4	1.619	136.59	-0.510	1.62	12.81	10.21
F5	3.067	179.40	-0.001	2.82	26.73	7.25
F6	1.618	134.89	-0.536	1.54	10.32	10.87
Halid2V2	1.579	134.53	-0.569	1.41	12.39	10.11
Halid3V2	1.575	134.17	-0.577	1.49	16.53	8.96
Halid4V2	1.572	133.94	-0.587	1.43	13.81	9.72
Halid5V2	1.572	134.10	-0.581	1.51	14.35	9.64
Halid6V2	1.574	134.07	-0.581	1.36	11.57	10.31
Halid7V2	1.567	134.40	-0.585	1.40	12.84	9.97
Halid8V2	1.574	134.15	-0.580	1.55	14.16	9.73
I0	1.600	135.99	-0.536	1.23	10.55	10.48
I1	1.576	134.95	-0.561	1.20	10.74	10.40
I2	1.573	134.67	-0.573	1.40	12.59	10.04
I3	1.647	139.17	-0.469	1.71	12.87	10.27
I4	1.629	138.04	-0.495	1.54	11.28	10.57
I5	1.723	142.41	-0.387	1.62	8.41	11.51
I6	1.629	134.99	-0.523	1.67	10.39	10.97
ImidazoleBr1	3.319	179.06	0.000	2.96	32.87	5.56
ImidazoleC#N1	3.842	179.20	0.000	2.83	29.86	6.33
ImidazoleC#N2	3.456	178.76	0.000	2.87	31.43	5.90
ImidazoleF1	3.512	179.10	0.000	2.95	34.84	4.97
ImidazoleF2	1.706	144.65	-0.337	2.07	17.64	9.21
ImidazoleI1	3.331	179.31	0.000	2.97	32.03	5.83
ImidazoleN1	1.652	139.67	-0.432	1.79	14.10	9.99

ImidazoleN2	1.675	141.16	-0.395	1.93	17.42	9.14
ImidazoleNO2_1	3.938	178.82	0.000	2.92	33.31	5.39
ImidazoleNO2_2	3.553	178.53	0.000	2.99	31.87	5.89
ImidazoleO1	1.663	141.30	-0.388	1.63	13.42	10.03
ImidazoleO2	1.690	143.00	-0.358	1.80	15.74	9.51
ImidazoleS1	3.409	179.02	0.000	3.09	32.81	5.70
ImidazoleS2	3.500	178.95	0.000	2.67	28.94	6.44
Ketone3V2	1.570	134.42	-0.578	1.36	11.00	10.49
Ketone4V2	1.575	134.23	-0.580	1.55	12.32	10.27
Ketone5V2	1.573	133.87	-0.586	1.16	13.65	9.50
Ketone6V2	1.574	134.17	-0.579	1.06	7.25	11.30
Ketone7V2	1.570	134.05	-0.583	1.37	10.43	10.66
Ketone8V2	1.574	134.17	-0.580	1.44	8.96	11.17
Nitro0	1.600	135.90	-0.534	1.06	6.50	11.52
Nitro1	1.581	134.90	-0.562	1.59	18.45	8.49
Nitro3	1.573	133.95	-0.583	1.39	11.08	10.48
Nitro30	1.621	138.02	-0.503	1.67	12.81	10.26
Nitro430	1.668	139.58	-0.435	1.75	9.43	11.34
Nitro4V2	1.569	134.39	-0.583	1.04	9.32	10.66
Nitro50	1.611	136.85	-0.521	1.60	12.01	10.42
Nitro5V2	1.573	134.69	-0.579	1.42	11.49	10.39
Nitro671	1.607	133.66	-0.548	1.63	12.42	10.33
Nitro6V2	1.573	134.39	-0.578	1.59	11.37	10.60
Nitro7V2	1.573	134.14	-0.581	1.51	12.75	10.11
Nitro8V2	1.573	134.20	-0.580	1.59	13.92	9.84
PiperidineBr1	1.613	135.60	-0.526	1.65	11.96	10.49
PiperidineBr2	1.600	134.55	-0.560	1.54	10.42	10.84
PiperidineBr3	1.600	133.56	-0.569	1.63	11.63	10.56
PiperidineC#N1	1.609	136.94	-0.519	1.76	14.83	9.74
PiperidineC#N2	1.600	136.34	-0.563	1.73	12.81	10.31
PiperidineC#N3	1.600	135.93	-0.548	1.53	10.96	10.66
PiperidineCl1	1.600	136.81	-0.544	1.37	9.20	11.02
PiperidineCl2	1.600	135.42	-0.545	1.44	9.98	10.86
PiperidineCl3	1.600	133.41	-0.571	1.56	10.96	10.69
PiperidineF1	1.600	136.03	-0.531	1.48	10.37	10.79
PiperidineF2	1.600	134.11	-0.567	1.53	11.13	10.61
PiperidineF3	1.585	134.43	-0.568	1.76	13.85	10.04

PiperidineI1	1.610	136.80	-0.516	1.81	13.42	10.21
PiperidineI2	1.600	135.75	-0.547	1.46	9.87	10.92
PiperidineI3	1.587	134.95	-0.562	1.77	14.31	9.91
PiperidineN1	1.578	133.61	-0.588	1.67	12.72	10.28
PiperidineN2	1.587	134.69	-0.570	1.70	13.17	10.18
PiperidineN3	1.585	134.64	-0.573	1.93	17.31	9.17
PiperidineNO2_1	1.623	137.66	-0.497	1.91	17.80	9.00
PiperidineNO2_2	1.600	135.66	-0.548	1.54	9.85	11.01
PiperidineNO2_3	1.600	135.13	-0.570	1.54	10.67	10.76
PiperidineO1	1.600	135.38	-0.536	1.61	11.58	10.56
PiperidineO2	1.591	135.12	-0.559	1.63	15.20	9.50
PiperidineO3	1.587	134.94	-0.562	1.74	13.53	10.11
PiperidineS1	1.600	135.94	-0.556	1.54	11.97	10.38
PiperidineS2	1.600	133.45	-0.576	1.49	11.10	10.58
PiperidineS3	1.600	133.41	-0.575	1.37	7.23	11.61
PyrazineBr1	1.664	138.76	-0.385	1.93	16.34	9.46
PyrazineBr2	1.618	138.85	-0.432	1.92	15.46	9.71
PyrazineC#N1	1.639	140.04	-0.396	1.83	13.11	10.32
PyrazineC#N2	1.628	139.78	-0.414	1.89	14.55	9.95
PyrazineCl1	1.622	137.98	-0.426	1.86	16.36	9.38
PyrazineCl2	1.608	138.67	-0.443	1.93	17.90	8.99
PyrazineF1	1.624	139.99	-0.409	1.91	15.74	9.62
PyrazineF2	1.616	138.90	-0.434	1.90	15.96	9.55
PyrazineI1	1.629	139.06	-0.417	1.92	14.70	9.94
PyrazineI2	1.610	138.47	-0.442	1.92	16.92	9.28
PyrazineN1	1.541	132.73	-0.541	1.76	14.53	9.82
PyrazineN2	1.592	136.22	-0.492	1.92	16.93	9.27
PyrazineNO2_1	1.693	143.64	-0.333	1.88	11.24	10.93
PyrazineNO2_2	1.641	140.87	-0.390	1.93	14.35	10.05
PyrazineO1	1.589	136.67	-0.458	1.79	14.75	9.79
PyrazineO2	1.610	137.89	-0.453	1.85	15.14	9.73
PyrazineS1	1.576	136.35	-0.468	1.53	11.05	10.64
PyrazineS2	1.602	137.71	-0.460	1.91	15.60	9.66
PyrazoleBr1	3.674	179.75	0.000	2.93	32.65	5.60
PyrazoleBr2	3.668	179.42	0.000	2.96	31.07	6.10
PyrazoleC#N1	4.654	179.53	0.000	2.88	31.14	6.00
PyrazoleC#N2	3.389	179.02	0.000	2.88	30.14	6.30

PyrazoleCl1	3.580	179.61	0.000	2.91	31.48	5.92
PyrazoleCl2	3.394	179.82	0.000	2.89	30.57	6.18
PyrazoleF1	3.686	179.59	0.000	2.99	36.19	4.60
PyrazoleF2	3.592	179.43	0.000	2.89	33.98	5.16
PyrazoleI1	3.740	179.50	-0.001	2.91	31.22	6.00
PyrazoleI2	3.179	179.16	0.000	2.95	30.64	6.21
PyrazoleN1	1.636	137.75	-0.483	1.93	15.97	9.57
PyrazoleN2	1.641	138.44	-0.459	1.77	13.38	10.18
PyrazoleNO2_1	4.914	179.49	0.000	3.06	40.09	3.51
PyrazoleNO2_2	3.545	178.90	0.000	2.90	28.86	6.69
PyrazoleO1	1.685	141.53	-0.399	1.78	15.22	9.64
PyrazoleO2	1.714	143.19	-0.367	1.81	15.52	9.59
PyrazoleS1	1.731	144.20	-0.347	2.11	18.05	9.13
PyrazoleS2	1.739	144.62	-0.337	2.22	22.39	7.95
PyridineBr1	1.608	134.68	-0.464	1.80	13.13	10.29
PyridineBr2	1.579	135.83	-0.497	1.86	14.99	9.79
PyridineBr3	1.576	135.52	-0.502	1.85	15.72	9.57
PyridineC#N1	1.593	136.67	-0.466	1.83	13.90	10.08
PyridineC#N2	1.583	136.59	-0.484	1.87	15.70	9.59
PyridineC#N3	1.580	136.29	-0.495	1.86	16.31	9.40
PyridineCl1	1.585	134.78	-0.488	1.78	13.74	10.09
PyridineCl2	1.579	135.85	-0.498	1.89	18.55	8.76
PyridineCl3	1.572	135.36	-0.508	1.86	15.14	9.74
PyridineF1	1.579	136.59	-0.479	1.84	14.67	9.87
PyridineF2	1.576	136.01	-0.501	1.85	15.99	9.49
PyridineF3	1.571	135.39	-0.507	1.87	16.28	9.42
PyridineI1	1.565	138.11	-0.498	1.61	10.32	10.93
PyridineI2	1.577	135.56	-0.502	1.86	14.67	9.89
PyridineI3	1.576	135.55	-0.504	1.87	16.06	9.48
PyridineN1	1.530	131.26	-0.572	1.93	16.07	9.54
PyridineN2	1.568	134.33	-0.535	1.92	17.45	9.12
PyridineN3	1.521	131.64	-0.601	1.90	21.47	7.90
PyridineNO2_1	1.623	139.19	-0.421	2.04	16.54	9.51
PyridineNO2_2	1.596	137.55	-0.459	1.85	13.81	10.14
PyridineNO2_3	1.591	137.34	-0.473	1.81	13.19	10.28
PyridineO1	1.538	132.60	-0.505	1.66	12.64	10.29
PyridineO2	1.576	135.23	-0.514	1.79	14.11	9.98

PyridineO3	1.550	133.86	-0.543	1.77	14.26	9.92
PyridineS1	1.569	133.63	-0.495	1.86	14.73	9.87
PyridineS2	1.575	135.36	-0.508	1.93	17.18	9.21
PyridineS3	1.562	134.55	-0.528	1.80	14.78	9.79
PyrimidineBr1	1.618	137.44	-0.422	1.83	14.91	9.78
PyrimidineBr2	1.641	140.41	-0.384	1.95	15.15	9.83
PyrimidineBr3	1.586	137.83	-0.460	1.88	16.67	9.31
PyrimidineC#N1	1.627	139.07	-0.405	1.91	16.65	9.34
PyrimidineC#N2	1.615	139.11	-0.414	2.07	17.02	9.40
PyrimidineC#N3	1.608	138.78	-0.430	1.90	16.57	9.36
PyrimidineCl1	1.603	136.74	-0.443	1.84	16.83	9.22
PyrimidineCl2	1.602	137.81	-0.437	1.83	13.86	10.10
PyrimidineCl3	1.597	137.79	-0.451	1.91	17.20	9.18
PyrimidineF1	1.597	138.93	-0.431	1.88	15.95	9.52
PyrimidineF2	1.598	138.71	-0.432	1.88	14.72	9.90
PyrimidineF3	1.600	137.98	-0.447	1.90	16.93	9.25
PyrimidineI1	1.596	139.21	-0.442	1.89	15.00	9.82
PyrimidineI2	1.610	138.36	-0.428	1.85	13.33	10.28
PyrimidineI3	1.592	137.50	-0.457	1.90	16.73	9.32
PyrimidineN1	1.540	132.79	-0.536	1.72	13.83	10.00
PyrimidineN2	1.536	132.58	-0.542	1.69	13.62	10.03
PyrimidineN3	1.585	136.28	-0.486	1.80	14.74	9.80
PyrimidineNO2_1	1.644	141.46	-0.374	2.02	18.19	8.99
PyrimidineNO2_2	1.662	142.44	-0.356	2.10	16.18	9.68
PyrimidineNO2_3	1.624	140.07	-0.403	1.94	16.21	9.51
PyrimidineO1	1.575	136.07	-0.469	1.84	14.12	10.03
PyrimidineO2	1.543	134.09	-0.473	1.66	12.56	10.31
PyrimidineO3	1.591	137.20	-0.468	1.73	13.79	10.02
PyrimidineS1	1.560	135.58	-0.486	1.62	11.82	10.50
PyrimidineS2	1.579	135.27	-0.460	1.91	15.34	9.74
PyrimidineS3	1.591	137.29	-0.462	1.90	15.17	9.77
PyrroleBr1	1.663	140.03	-0.425	1.91	16.09	9.51
PyrroleBr2	1.658	139.87	-0.432	1.92	16.24	9.48
PyrroleC#N1	1.686	141.87	-0.391	1.92	14.69	9.94
PyrroleC#N2	1.695	142.27	-0.382	2.00	16.14	9.59
PyrroleCl1	1.658	139.86	-0.430	1.84	14.58	9.89
PyrroleCl2	1.653	139.45	-0.440	1.88	16.28	9.43

PyrroleF1	1.670	140.80	-0.410	1.91	16.28	9.46
PyrroleF2	1.653	139.51	-0.439	1.82	14.56	9.87
PyrroleI2	1.649	139.32	-0.441	1.95	17.11	9.25
PyrroleN1	1.633	138.63	-0.467	1.74	13.83	10.02
PyrroleN2	1.606	135.89	-0.525	1.80	16.85	9.17
PyrroleNO2_1	3.496	179.15	0.000	2.94	29.60	6.51
PyrroleNO2_2	3.982	179.73	0.000	2.93	31.64	5.90
PyrroleO1	1.630	138.42	-0.453	1.57	12.60	10.22
PyrroleO2	1.626	137.43	-0.485	1.76	14.48	9.84
PyrroleS1	1.662	140.43	-0.426	1.66	13.59	10.00
PyrroleS2	1.634	138.08	-0.470	1.74	13.95	9.98
ThiazoleBr1	1.568	134.41	-0.495	1.82	14.52	9.89
ThiazoleBr2	1.558	135.12	-0.508	1.87	15.06	9.78
ThiazoleBr3	1.573	134.01	-0.493	1.78	14.22	9.95
ThiazoleC#N1	1.563	135.42	-0.496	1.77	13.26	10.22
ThiazoleC#N2	1.563	135.71	-0.494	1.82	14.45	9.92
ThiazoleC#N3	1.570	135.91	-0.482	1.84	14.78	9.84
ThiazoleCl1	1.554	134.48	-0.513	1.70	12.16	10.48
ThiazoleCl2	1.559	135.14	-0.508	1.85	15.05	9.76
ThiazoleCl3	1.559	134.46	-0.506	1.84	16.20	9.41
ThiazoleF1	1.558	135.62	-0.494	1.89	15.11	9.78
ThiazoleF2	1.562	135.40	-0.502	1.85	15.31	9.69
ThiazoleF3	1.557	135.73	-0.495	1.89	15.24	9.74
ThiazoleI1	1.560	134.67	-0.506	1.79	12.88	10.35
ThiazoleN1	1.509	131.65	-0.585	1.65	13.34	10.08
ThiazoleN2	1.556	134.25	-0.532	1.92	16.56	9.39
ThiazoleN3	1.532	132.56	-0.550	1.70	13.50	10.07
ThiazoleNO2_1	1.569	136.13	-0.484	1.90	15.30	9.74
ThiazoleNO2_2	1.574	136.67	-0.472	1.91	15.83	9.59
ThiazoleNO2_3	1.589	137.32	-0.455	1.65	10.69	10.86
ThiazoleO1	1.539	133.88	-0.524	1.73	14.23	9.89
ThiazoleO2	1.560	135.31	-0.512	1.83	15.46	9.62
ThiazoleO3	1.544	134.01	-0.518	1.81	15.20	9.68
ThiazoleS1	1.525	133.17	-0.548	1.59	11.26	10.63
ThiazoleS2	1.551	134.40	-0.526	2.06	16.07	9.67
ThiazoleS3	1.532	132.95	-0.537	1.57	11.18	10.64
Tio0	1.579	134.32	-0.569	0.95	6.52	11.41

Tio1	1.575	134.15	-0.577	1.28	9.92	10.73
Tio2	1.574	134.12	-0.579	1.63	14.44	9.73
Tio30	1.603	135.73	-0.542	1.73	13.01	10.25
Tio3V2	1.574	134.22	-0.578	1.53	14.35	9.66
Tio430	1.632	134.30	-0.556	1.74	6.65	12.15
Tio4V2	1.574	134.07	-0.581	1.17	9.22	10.82
Tio50	1.600	136.04	-0.568	1.50	9.37	11.11
Tio5V2	1.567	134.40	-0.585	1.35	14.75	9.36
Tio671	1.612	134.27	-0.556	1.87	14.45	9.96
Tio6V2	1.569	134.03	-0.584	1.65	17.38	8.87
Tio7V2	1.576	134.30	-0.577	1.35	9.92	10.80
Tio8V2	1.575	134.28	-0.579	1.52	12.86	10.09
TriazoleBr2	3.251	178.17	0.000	2.19	22.81	7.79
TriazoleC#N1	3.891	179.07	0.000	2.97	33.94	5.25
TriazoleC#N2	4.104	178.75	0.000	2.11	22.23	7.89
TriazoleCl1	4.740	179.62	0.000	2.98	38.75	3.83
TriazoleCl2	4.093	179.01	0.000	2.11	23.18	7.60
TriazoleF1	4.365	179.37	0.000	2.96	36.24	4.56
TriazoleF2	4.169	179.13	0.000	2.27	29.64	5.84
TriazoleI2	3.740	179.14	0.000	1.67	15.29	9.51
TriazoleN1	3.771	179.16	0.000	2.95	34.42	5.09
TriazoleN2	4.125	179.22	0.000	2.38	29.31	6.05
TriazoleNO2_1	5.674	179.97	0.000	2.92	32.56	5.61
TriazoleNO2_2	3.186	177.01	0.001	2.14	21.68	8.08
TriazoleO1	3.613	178.99	0.000	3.03	32.65	5.70
TriazoleO2	3.767	179.22	0.000	2.97	33.09	5.50
TriazoleS1	3.977	179.36	0.000	2.94	31.56	5.94
TriazoleS2	3.265	177.82	0.001	1.92	18.89	8.69

Table S11: Parameters calculated using the PM6-D3 method.

Name	R (Å)	θ (°)	ΔQ	ΔH (kcal/mol)	ΔS (cal/molK)	ΔG (kcal/mol)
Acid0	1.598	136.61	-0.520	1.53	12.82	10.11
Acid1	1.573	135.58	-0.561	1.20	9.92	10.64
Acid2	1.578	134.38	-0.573	1.57	15.31	9.41
Acid30	1.648	139.79	-0.440	1.85	18.29	8.80
Acid3V2	1.576	134.43	-0.575	1.56	15.93	9.21

Acid430	1.761	146.60	-0.333	1.93	12.38	10.64
Acid4V2	1.576	134.25	-0.578	1.52	13.92	9.77
Acid50	1.614	137.46	-0.467	1.60	16.48	9.09
Acid671	1.623	135.26	-0.504	1.70	6.33	12.21
Acid6V2	1.576	134.54	-0.571	1.08	4.81	12.04
Acid7V2	1.575	134.20	-0.580	1.30	16.11	8.90
Acid8V2	1.575	134.28	-0.579	1.47	11.35	10.49
AcidBr1	1.588	135.43	-0.548	1.38	10.74	10.58
AcidBr2	1.580	134.67	-0.567	1.65	15.17	9.52
AcidBr3	1.674	141.55	-0.417	1.75	13.95	9.99
AcidBr4	1.653	140.21	-0.450	1.54	14.01	9.76
AcidBr5	3.179	178.64	-0.001	2.95	24.72	7.98
AcidBr6	1.661	138.65	-0.466	1.78	15.52	9.55
AcidCl0	1.600	136.97	-0.511	1.46	12.26	10.21
AcidCl1	1.583	135.51	-0.552	1.40	12.70	10.02
AcidCl2	1.600	133.94	-0.555	1.38	9.33	11.00
AcidCl3	1.654	141.42	-0.437	1.69	14.66	9.72
AcidCl4	1.635	139.02	-0.473	1.16	6.18	11.72
AcidCl5	2.890	179.37	-0.002	2.83	25.52	7.62
AcidCl6	1.654	137.04	-0.479	1.50	4.17	12.66
AcidF0	1.602	136.82	-0.510	1.55	15.01	9.48
AcidF1	1.586	135.31	-0.551	1.57	15.19	9.45
AcidF2	1.575	134.44	-0.571	1.70	18.06	8.72
AcidF3	1.669	141.93	-0.419	1.59	9.12	11.27
AcidF4	1.642	138.91	-0.468	1.67	13.39	10.08
AcidF5	2.895	179.08	-0.001	2.73	19.53	9.31
AcidF6	1.641	137.02	-0.490	1.43	3.97	12.64
AcidI0	1.600	136.91	-0.514	1.53	13.38	9.94
AcidI1	1.587	135.45	-0.549	1.52	14.63	9.56
AcidI2	1.573	135.10	-0.569	1.17	8.13	11.15
AcidI3	1.674	141.50	-0.421	1.89	17.76	8.99
AcidI4	1.644	139.65	-0.462	1.68	12.07	10.48
AcidI5	2.894	179.45	-0.002	2.63	20.91	8.79
AcidI6	1.641	137.49	-0.488	1.55	6.00	12.16
Acid_Halid3V2	1.576	134.38	-0.575	1.49	13.68	9.82
Acid_Halid4V2	1.575	134.27	-0.578	1.57	15.53	9.34
Acid_Halid5V2	1.572	134.21	-0.581	1.39	13.17	9.86

Acid_Halid6V2	1.573	134.29	-0.580	1.51	15.43	9.31
Acid_Halid7V2	1.575	134.25	-0.579	1.54	13.98	9.78
Acid_Halid8V2	1.575	134.34	-0.578	1.38	10.17	10.75
Acid_Sulf0	1.585	135.06	-0.556	1.51	11.76	10.40
Acid_Sulf1	1.578	134.32	-0.575	1.07	4.16	12.23
Acid_Sulf2	1.576	134.30	-0.576	1.49	11.41	10.49
Acid_Sulf30	1.616	136.68	-0.512	1.16	5.62	11.89
Acid_Sulf3V2	1.566	134.50	-0.585	1.99	14.31	10.12
Acid_Sulf430	1.640	136.81	-0.489	2.00	15.76	9.70
Acid_Sulf4V2	1.575	134.30	-0.578	1.25	7.24	11.49
Acid_Sulf50	1.612	136.21	-0.523	1.91	17.44	9.11
Acid_Sulf5V2	1.574	134.10	-0.581	1.00	6.31	11.51
Acid_Sulf671	1.625	135.43	-0.533	1.42	9.77	10.91
Acid_Sulf6V2	1.575	134.23	-0.579	1.66	14.25	9.81
Acid_Sulf7V2	1.576	134.25	-0.578	1.29	8.79	11.07
Acid_Sulf8V2	1.577	134.43	-0.576	1.02	1.87	12.86
Acid_Tio0	1.600	135.72	-0.526	1.16	8.84	10.92
Acid_Tio1	1.580	134.84	-0.564	1.41	12.07	10.21
Acid_Tio2	1.577	134.44	-0.575	1.68	18.38	8.60
Acid_Tio30	1.635	139.61	-0.472	2.12	18.94	8.87
Acid_Tio3V2	1.575	134.21	-0.579	1.64	16.12	9.23
Acid_Tio430	1.697	141.82	-0.407	2.05	16.67	9.48
Acid_Tio4V2	1.574	134.11	-0.580	1.75	16.23	9.31
Acid_Tio50	1.606	135.99	-0.526	1.44	6.55	11.89
Acid_Tio5V2	1.574	134.09	-0.582	1.29	11.45	10.28
Acid_Tio671	1.614	133.28	-0.540	1.09	-2.62	14.27
Acid_Tio6V2	1.574	134.18	-0.581	1.53	14.19	9.70
Acid_Tio7V2	1.574	134.12	-0.582	1.34	6.99	11.66
Acid_Tio8V2	1.575	134.16	-0.580	1.49	9.09	11.18
Alcohol0	1.577	134.66	-0.568	1.39	13.60	9.74
Alcohol1	1.577	134.41	-0.574	1.41	9.91	10.86
Alcohol2	1.574	134.33	-0.578	1.45	13.93	9.70
Alcohol30	1.591	134.89	-0.560	1.13	6.80	11.50
Alcohol3V2	1.575	134.27	-0.578	1.47	11.33	10.49
Alcohol430	1.626	135.14	-0.528	2.00	15.82	9.68
Alcohol4V2	1.569	134.57	-0.582	1.50	14.08	9.70
Alcohol50	1.600	136.06	-0.558	1.80	13.70	10.11

Alcohol5V2	1.575	134.19	-0.580	1.34	9.73	10.84
Alcohol671	1.600	132.42	-0.564	1.04	0.38	13.32
Alcohol6V2	1.574	134.19	-0.580	1.82	17.16	9.11
Alcohol7V2	1.576	134.28	-0.578	1.57	11.98	10.40
Alcohol8V2	1.574	134.18	-0.579	1.37	10.00	10.79
Aldehyde0	1.587	135.41	-0.550	1.48	14.62	9.52
Aldehyde1	1.575	134.58	-0.570	1.28	11.40	10.28
Aldehyde2	1.578	134.46	-0.574	1.62	13.67	9.95
Aldehyde30	1.617	136.98	-0.517	1.34	7.97	11.36
Aldehyde430	1.644	137.33	-0.495	1.56	10.58	10.80
Aldehyde50	1.600	137.67	-0.544	1.22	8.53	11.08
Aldehyde671	1.621	135.57	-0.537	1.58	10.17	10.95
Amide0	1.588	135.29	-0.537	1.20	8.67	11.02
Amide1	1.578	134.58	-0.574	1.35	11.36	10.36
Amide2	1.578	134.61	-0.571	1.37	11.35	10.38
Amide30	1.641	139.16	-0.459	1.50	12.39	10.21
Amide3V2	1.574	134.26	-0.578	1.65	17.44	8.85
Amide430	1.686	140.67	-0.426	1.43	6.95	11.75
Amide4V2	1.576	134.29	-0.578	1.48	11.41	10.48
Amide50	1.617	137.12	-0.510	1.48	8.68	11.29
Amide5V2	1.573	133.93	-0.586	0.93	3.54	12.27
Amide671	1.644	136.25	-0.513	1.48	4.59	12.51
Amide6V2	1.567	135.12	-0.582	1.02	5.04	11.92
Amide7V2	1.575	134.24	-0.579	1.54	12.34	10.26
Amide8V2	1.574	134.24	-0.579	1.34	7.30	11.56
Amine0	1.577	134.17	-0.576	1.54	14.77	9.54
Amine1	1.575	134.31	-0.582	1.44	14.37	9.56
Amine2	1.574	134.42	-0.583	1.54	15.40	9.35
Amine30	1.576	134.42	-0.575	1.23	6.52	11.68
Amine3V2	1.568	134.61	-0.584	1.37	11.32	10.40
Amine430	1.608	133.70	-0.559	1.30	3.54	12.65
Amine4V2	1.567	134.48	-0.585	0.91	4.47	11.98
Amine50	1.600	134.98	-0.563	1.32	9.23	10.97
Amine5V2	1.575	134.21	-0.579	1.23	7.73	11.32
Amine671	1.602	132.48	-0.576	0.91	-4.78	14.73
Amine6V2	1.567	134.60	-0.584	1.47	12.81	10.06
Amine7V2	1.575	134.23	-0.579	1.44	6.65	11.86

Amine8V2	1.575	134.22	-0.580	1.73	16.55	9.19
Anhydride0	1.601	136.13	-0.522	1.11	5.30	11.94
Anhydride1	1.585	135.22	-0.554	1.54	15.30	9.38
Anhydride2	1.575	134.85	-0.569	1.54	13.13	10.02
Anhydride30	1.663	141.17	-0.425	1.74	12.21	10.50
Anhydride3V2	1.576	134.55	-0.573	1.22	7.42	11.41
Anhydride430	2.846	179.15	-0.002	2.53	17.95	9.58
Anhydride4V2	1.576	134.28	-0.577	1.48	11.46	10.47
Anhydride50	1.621	136.43	-0.510	1.63	14.12	9.82
Anhydride5V2	1.573	134.22	-0.580	1.37	11.81	10.25
Anhydride671	1.636	136.58	-0.500	1.88	9.84	11.35
Anhydride7V2	1.574	134.21	-0.580	1.32	10.25	10.66
Anhydride8V2	1.575	134.17	-0.579	1.20	7.51	11.36
AzolidineBr1	1.600	136.39	-0.524	1.20	9.08	10.89
AzolidineBr2	1.591	134.86	-0.560	1.53	9.83	11.00
AzolidineC#N1	1.600	138.60	-0.516	1.55	11.45	10.54
AzolidineC#N2	1.600	137.14	-0.554	1.36	8.35	11.27
AzolidineCl1	1.601	136.79	-0.518	0.84	4.27	11.97
AzolidineCl2	1.600	134.66	-0.550	1.42	10.12	10.80
AzolidineF1	1.595	136.27	-0.530	1.71	15.77	9.41
AzolidineF2	1.590	134.91	-0.561	1.62	12.87	10.19
AzolidineI1	1.600	135.96	-0.528	1.06	2.84	12.62
AzolidineI2	1.588	134.88	-0.563	1.72	14.19	9.89
AzolidineN1	1.575	134.18	-0.578	1.51	11.76	10.40
AzolidineN2	1.585	134.32	-0.576	1.49	12.59	10.13
AzolidineNO2_1	1.600	137.32	-0.517	1.29	6.36	11.80
AzolidineNO2_2	1.600	136.07	-0.539	1.28	8.53	11.14
AzolidineO1	1.583	135.14	-0.558	1.45	12.63	10.08
AzolidineO2	1.590	135.21	-0.554	1.56	13.38	9.97
AzolidineS1	1.600	135.22	-0.549	1.43	9.57	10.98
AzolidineS2	1.587	134.93	-0.559	1.76	13.97	10.00
Br0	1.602	136.28	-0.540	1.07	9.14	10.75
Br1	1.584	135.03	-0.560	1.18	10.37	10.48
Br2	1.575	134.89	-0.571	1.44	13.33	9.86
Br3	1.664	140.02	-0.455	1.82	11.73	10.72
Br4	1.634	138.27	-0.491	1.49	9.38	11.10
Br5	3.076	178.40	0.000	3.15	29.58	6.73

Br6	1.629	135.93	-0.514	1.76	16.70	9.18
Cl0	1.604	137.01	-0.514	1.14	10.18	10.50
Cl1	1.581	134.75	-0.566	1.34	13.16	9.82
Cl2	1.577	134.60	-0.570	1.25	11.43	10.25
Cl3	1.651	139.91	-0.451	1.56	11.71	10.47
Cl4	1.632	138.84	-0.480	1.44	8.46	11.32
Cl5	3.020	179.44	-0.007	2.54	25.02	7.48
Cl6	1.644	137.31	-0.494	1.98	21.70	7.91
Cyan0	1.602	137.01	-0.515	1.50	13.66	9.83
Cyan1	1.581	134.48	-0.563	1.40	14.06	9.61
Cyan2	1.578	134.36	-0.574	1.52	19.15	8.21
Cyan30	1.643	139.90	-0.462	1.67	13.33	10.10
Cyan3V2	1.576	134.34	-0.576	1.41	11.42	10.40
Cyan430	1.727	144.03	-0.362	2.36	19.79	8.87
Cyan4V2	1.572	134.43	-0.581	1.28	12.49	9.96
Cyan50	1.629	137.75	-0.497	1.78	17.37	9.00
Cyan5V2	1.570	134.62	-0.583	1.24	10.28	10.58
Cyan671	1.634	136.22	-0.502	1.56	9.07	11.26
Cyan6V2	1.574	134.24	-0.579	1.70	16.32	9.23
Cyan7V2	1.574	134.23	-0.580	1.40	9.20	11.05
Cyan8V2	1.576	134.28	-0.578	1.49	15.61	9.23
Ester0	1.600	135.46	-0.525	1.58	14.12	9.78
Ester1	1.583	135.13	-0.557	1.37	10.67	10.59
Ester2	1.578	134.46	-0.571	1.37	11.58	10.31
Ester30	1.636	138.89	-0.472	1.73	13.11	10.22
Ester3V2	1.576	134.38	-0.576	1.37	12.19	10.13
Ester430	1.702	141.10	-0.420	1.89	14.21	10.06
Ester4V2	1.576	134.32	-0.577	1.61	14.46	9.70
Ester50	1.600	136.50	-0.538	1.31	7.80	11.38
Ester5V2	1.573	134.23	-0.581	1.17	5.36	11.97
Ester671	1.629	136.06	-0.518	1.41	3.35	12.82
Ester6V2	1.576	134.32	-0.577	1.60	13.58	9.95
Ester7V2	1.574	134.24	-0.580	1.21	6.56	11.65
Ester8V2	1.575	134.27	-0.579	1.68	14.64	9.71
Ether0	1.600	136.06	-0.525	1.47	12.70	10.08
Ether1	1.576	134.63	-0.570	1.49	18.57	8.36
Ether2	1.571	134.75	-0.578	1.41	13.94	9.65

Ether30	1.611	136.59	-0.516	1.63	10.10	11.02
Ether3V2	1.573	134.11	-0.580	1.45	15.50	9.23
Ether430	1.627	135.50	-0.512	1.57	10.52	10.83
Ether4V2	1.575	134.01	-0.581	1.25	8.49	11.12
Ether50	1.600	136.51	-0.537	1.30	9.11	10.99
Ether5V2	1.569	134.59	-0.583	1.22	11.17	10.29
Ether671	1.621	135.32	-0.536	1.72	9.46	11.30
Ether6V2	1.573	134.04	-0.581	1.24	8.30	11.16
Ether7V2	1.576	134.30	-0.578	1.51	15.02	9.44
Ether8V2	1.576	134.41	-0.576	1.49	16.70	8.92
F0	1.597	136.63	-0.521	1.99	20.80	8.19
F1	1.584	134.90	-0.566	1.32	11.70	10.24
F2	1.571	134.99	-0.571	1.13	10.41	10.42
F4	1.618	137.49	-0.505	1.64	12.86	10.21
F5	2.818	179.08	-0.003	2.76	26.40	7.29
F6	1.620	135.37	-0.533	1.61	9.55	11.16
Halid2V2	1.600	134.87	-0.549	1.29	10.54	10.55
Halid3V2	1.575	134.27	-0.577	1.27	9.51	10.83
Halid4V2	1.573	134.02	-0.586	1.48	14.77	9.48
Halid5V2	1.572	134.29	-0.581	1.40	12.39	10.10
Halid6V2	1.574	134.22	-0.580	1.43	15.06	9.34
Halid7V2	1.567	134.48	-0.585	1.39	12.70	10.01
Halid8V2	1.574	134.28	-0.579	1.69	16.49	9.18
I0	1.600	136.86	-0.534	1.29	11.74	10.19
I1	1.576	135.09	-0.561	1.24	11.17	10.30
I2	1.574	134.76	-0.573	1.39	12.30	10.12
I3	1.647	139.26	-0.468	1.55	15.16	9.43
I4	1.623	137.76	-0.504	1.80	18.02	8.83
I6	1.627	135.88	-0.524	1.79	11.15	10.87
ImidazoleBr1	3.164	178.85	0.000	2.80	26.82	7.20
ImidazoleC#N1	2.956	177.36	0.001	2.78	28.69	6.63
ImidazoleC#N2	3.119	178.29	0.000	2.85	29.51	6.45
ImidazoleCl1	3.260	179.08	0.001	2.85	31.58	5.83
ImidazoleF1	3.138	178.86	0.000	2.84	28.85	6.64
ImidazoleF2	1.704	144.64	-0.340	2.03	16.70	9.45
ImidazoleI1	3.171	179.18	-0.001	2.81	27.46	7.02
ImidazoleN1	1.651	140.01	-0.430	1.71	13.54	10.07

ImidazoleN2	1.676	141.46	-0.392	1.81	14.32	9.94
ImidazoleNO2_1	3.575	179.59	0.003	2.84	28.69	6.69
ImidazoleNO2_2	3.304	178.78	0.001	2.83	27.35	7.08
ImidazoleO1	1.662	141.11	-0.387	1.48	11.68	10.39
ImidazoleO2	1.678	142.20	-0.368	1.58	12.01	10.40
ImidazoleS1	3.151	178.75	0.000	2.82	29.74	6.36
ImidazoleS2	3.417	179.39	0.000	2.74	25.01	7.68
Ketone3V2	1.576	134.45	-0.575	1.56	13.30	9.99
Ketone4V2	1.575	134.25	-0.579	1.57	17.11	8.87
Ketone5V2	1.572	133.91	-0.587	1.41	12.02	10.22
Ketone6V2	1.574	134.20	-0.579	1.15	7.69	11.26
Ketone7V2	1.574	133.73	-0.582	1.36	9.17	11.03
Ketone8V2	1.575	134.27	-0.579	1.63	10.00	11.04
Nitro0	1.600	135.99	-0.534	1.07	9.26	10.71
Nitro1	1.582	134.87	-0.562	1.56	16.22	9.12
Nitro3	1.600	134.54	-0.585	0.97	4.26	12.10
Nitro30	1.619	137.77	-0.502	1.70	12.43	10.39
Nitro430	1.669	139.67	-0.434	1.66	9.14	11.34
Nitro4V2	1.570	134.47	-0.582	0.98	-0.10	13.42
Nitro50	1.611	137.12	-0.522	1.66	14.69	9.69
Nitro5V2	1.574	134.79	-0.578	1.39	10.86	10.55
Nitro671	1.607	133.77	-0.551	1.68	11.80	10.56
Nitro6V2	1.575	134.27	-0.578	1.51	10.74	10.70
Nitro7V2	1.575	134.21	-0.580	1.42	16.41	8.93
Nitro8V2	1.575	134.25	-0.579	1.50	10.17	10.86
PiperidineBr1	1.612	135.92	-0.524	1.67	11.54	10.63
PiperidineBr2	1.600	134.21	-0.562	1.61	11.52	10.57
PiperidineBr3	1.600	133.74	-0.569	1.63	11.81	10.51
PiperidineC#N1	1.610	137.16	-0.518	1.71	12.85	10.28
PiperidineC#N2	1.600	136.47	-0.562	1.66	11.46	10.64
PiperidineC#N3	1.600	135.17	-0.555	1.50	10.75	10.70
PiperidineCl1	1.600	136.93	-0.544	1.42	9.85	10.89
PiperidineCl2	1.600	135.35	-0.548	1.43	10.06	10.83
PiperidineCl3	1.600	133.76	-0.571	1.53	10.58	10.78
PiperidineF1	1.600	136.15	-0.530	1.47	10.32	10.80
PiperidineF2	1.600	134.30	-0.566	1.66	12.67	10.29
PiperidineF3	1.590	134.87	-0.565	1.74	14.23	9.90

PiperidineI1	1.612	136.94	-0.517	1.81	13.82	10.09
PiperidineI2	1.600	135.33	-0.550	1.47	10.71	10.68
PiperidineI3	1.591	135.09	-0.561	1.76	17.87	8.83
PiperidineN1	1.591	135.33	-0.555	1.81	15.18	9.69
PiperidineN2	1.589	134.80	-0.569	1.58	11.51	10.55
PiperidineN3	1.585	134.77	-0.572	1.77	15.15	9.65
PiperidineNO2_1	1.620	136.89	-0.506	1.59	10.54	10.84
PiperidineNO2_2	1.598	134.95	-0.550	1.63	11.98	10.46
PiperidineNO2_3	1.600	135.25	-0.569	1.51	9.02	11.23
PiperidineO1	1.600	135.12	-0.533	1.31	8.78	11.09
PiperidineO2	1.592	135.21	-0.558	1.78	15.30	9.62
PiperidineO3	1.590	135.37	-0.560	1.68	14.44	9.77
PiperidineS1	1.600	135.96	-0.555	1.58	12.67	10.20
PiperidineS2	1.600	133.58	-0.576	1.69	12.71	10.30
PiperidineS3	1.600	133.53	-0.575	1.50	9.90	10.95
PyrazineBr1	1.667	138.83	-0.382	1.96	17.51	9.14
PyrazineBr2	1.620	138.84	-0.431	1.92	15.43	9.72
PyrazineC#N1	1.646	140.17	-0.391	1.99	16.26	9.54
PyrazineC#N2	1.624	139.60	-0.418	1.85	14.26	10.00
PyrazineCl1	1.622	137.95	-0.427	1.85	14.47	9.94
PyrazineCl2	1.608	138.61	-0.443	1.90	15.86	9.57
PyrazineF1	1.625	139.94	-0.408	1.90	15.30	9.74
PyrazineF2	1.617	138.85	-0.433	1.91	17.07	9.22
PyrazineI1	1.627	138.72	-0.420	1.89	13.89	10.15
PyrazineI2	1.611	138.42	-0.442	1.90	15.05	9.81
PyrazineN1	1.544	132.58	-0.539	1.73	14.51	9.80
PyrazineN2	1.591	135.98	-0.496	1.92	15.94	9.57
PyrazineNO2_1	1.674	142.69	-0.352	1.84	12.55	10.50
PyrazineNO2_2	1.647	141.11	-0.384	1.93	14.12	10.12
PyrazineO1	1.588	136.49	-0.460	1.83	14.90	9.79
PyrazineO2	1.611	137.83	-0.453	1.83	15.19	9.70
PyrazineS1	1.578	136.32	-0.468	1.52	11.40	10.52
PyrazineS2	1.603	137.65	-0.459	1.90	15.55	9.67
PyrazoleBr1	3.197	179.09	0.001	2.82	31.56	5.81
PyrazoleBr2	1.810	149.07	-0.257	2.17	19.21	8.85
PyrazoleC#N1	3.284	178.95	0.001	2.82	29.66	6.38
PyrazoleC#N2	3.195	178.95	0.001	2.82	30.19	6.22

PyrazoleCl1	3.169	179.39	0.000	2.87	29.98	6.33
PyrazoleCl2	1.711	143.73	-0.354	2.02	17.71	9.14
PyrazoleF1	3.209	179.11	0.001	2.89	31.61	5.87
PyrazoleF2	3.178	179.23	0.000	2.88	33.77	5.21
PyrazoleI1	3.181	178.80	0.000	2.93	30.87	6.13
PyrazoleI2	3.177	178.89	0.001	2.82	30.86	6.01
PyrazoleN1	1.636	137.88	-0.483	1.98	16.20	9.55
PyrazoleN2	1.663	139.76	-0.440	1.80	14.64	9.83
PyrazoleNO2_1	3.234	178.46	0.000	2.88	31.31	5.95
PyrazoleNO2_2	3.400	179.26	0.001	2.83	30.88	6.02
PyrazoleO1	1.686	141.75	-0.397	1.81	15.27	9.66
PyrazoleO2	1.719	143.65	-0.362	1.84	15.65	9.58
PyrazoleS1	1.718	143.92	-0.356	1.97	16.10	9.57
PyrazoleS2	3.157	179.02	0.001	2.77	27.41	7.00
PyridineBr1	1.609	134.71	-0.463	1.76	12.71	10.37
PyridineBr2	1.581	135.70	-0.497	1.85	14.40	9.96
PyridineBr3	1.578	135.45	-0.501	1.84	15.43	9.64
PyridineC#N1	1.595	136.50	-0.465	1.85	17.00	9.18
PyridineC#N2	1.586	136.55	-0.482	1.83	14.30	9.96
PyridineC#N3	1.580	136.21	-0.495	1.85	15.22	9.71
PyridineCl1	1.586	134.73	-0.487	1.78	13.55	10.14
PyridineCl2	1.575	135.83	-0.503	1.81	14.31	9.95
PyridineCl3	1.573	135.27	-0.508	1.86	15.27	9.71
PyridineF1	1.580	136.50	-0.478	1.84	14.68	9.87
PyridineF2	1.582	135.91	-0.494	1.84	14.88	9.81
PyridineF3	1.574	135.29	-0.505	1.75	13.11	10.24
PyridineI1	1.576	139.85	-0.485	1.89	18.08	8.90
PyridineI2	1.577	135.52	-0.502	1.84	14.70	9.86
PyridineI3	1.577	135.49	-0.504	1.86	15.10	9.76
PyridineN1	1.529	130.90	-0.575	1.79	15.07	9.70
PyridineN2	1.568	134.25	-0.535	1.90	16.10	9.50
PyridineN3	1.522	131.57	-0.600	1.86	14.76	9.86
PyridineNO2_1	1.616	138.98	-0.428	2.04	16.86	9.42
PyridineNO2_2	1.599	137.56	-0.457	1.83	13.29	10.26
PyridineNO2_3	1.591	137.26	-0.473	1.87	14.90	9.83
PyridineO1	1.542	132.52	-0.503	1.60	12.41	10.30
PyridineO2	1.577	135.14	-0.513	1.81	14.24	9.96

PyridineO3	1.552	133.82	-0.542	1.78	14.44	9.87
PyridineS1	1.572	133.86	-0.497	1.98	16.51	9.46
PyridineS2	1.575	135.27	-0.508	1.94	15.77	9.64
PyridineS3	1.563	134.47	-0.527	1.81	15.23	9.67
PyrimidineBr1	1.620	137.45	-0.420	1.83	14.86	9.80
PyrimidineBr2	1.643	140.66	-0.382	1.92	14.47	10.00
PyrimidineBr3	1.587	137.81	-0.460	1.87	16.00	9.50
PyrimidineC#N1	1.626	139.35	-0.404	1.89	16.26	9.44
PyrimidineC#N2	1.616	139.14	-0.413	1.96	16.19	9.54
PyrimidineC#N3	1.607	138.75	-0.431	1.88	16.10	9.48
PyrimidineCl1	1.605	136.71	-0.443	1.81	15.55	9.58
PyrimidineCl2	1.603	137.95	-0.435	1.85	13.87	10.11
PyrimidineCl3	1.597	137.77	-0.451	1.90	16.74	9.31
PyrimidineF1	1.603	138.92	-0.426	1.93	18.08	8.94
PyrimidineF2	1.599	138.69	-0.430	1.88	14.30	10.01
PyrimidineF3	1.601	137.99	-0.447	1.89	16.94	9.24
PyrimidineI1	1.597	137.46	-0.449	1.96	17.02	9.29
PyrimidineI2	1.611	138.17	-0.428	1.96	15.45	9.76
PyrimidineI3	1.599	137.75	-0.450	1.95	17.58	9.11
PyrimidineN1	1.542	132.61	-0.533	1.72	13.91	9.98
PyrimidineN2	1.538	132.49	-0.540	1.63	13.32	10.06
PyrimidineN3	1.582	136.01	-0.492	1.87	16.40	9.38
PyrimidineNO2_1	1.648	141.62	-0.370	2.28	20.34	8.62
PyrimidineNO2_2	1.637	141.06	-0.380	1.99	14.34	10.11
PyrimidineNO2_3	1.625	140.09	-0.402	1.92	15.82	9.61
PyrimidineO1	1.575	136.03	-0.469	1.86	14.49	9.94
PyrimidineO2	1.547	134.05	-0.472	1.62	12.31	10.35
PyrimidineO3	1.593	137.10	-0.467	1.83	14.51	9.90
PyrimidineS1	1.558	135.17	-0.487	1.66	12.14	10.44
PyrimidineS2	1.579	135.12	-0.460	1.81	14.69	9.83
PyrimidineS3	1.592	137.26	-0.461	1.83	15.31	9.67
PyrroleBr1	1.664	140.27	-0.424	1.90	16.02	9.52
PyrroleBr2	1.658	139.87	-0.432	1.89	15.18	9.77
PyrroleC#N1	1.685	141.84	-0.394	1.97	16.32	9.51
PyrroleC#N2	1.698	142.44	-0.380	1.98	15.47	9.76
PyrroleCl1	1.656	140.22	-0.430	1.90	15.74	9.61
PyrroleCl2	1.652	139.43	-0.441	1.93	19.74	8.45

PyrroleF1	1.670	140.82	-0.411	1.93	18.63	8.78
PyrroleF2	1.654	139.63	-0.438	1.83	15.09	9.73
PyrroleI2	1.655	139.62	-0.436	1.89	15.11	9.79
PyrroleN1	1.631	138.08	-0.473	1.71	13.92	9.96
PyrroleN2	1.605	135.92	-0.526	1.65	13.22	10.11
PyrroleNO2_1	3.283	178.83	0.001	2.90	30.59	6.18
PyrroleNO2_2	2.065	163.96	-0.079	2.45	20.87	8.63
PyrroleO1	1.630	138.32	-0.452	1.61	13.02	10.13
PyrroleO2	1.628	137.60	-0.483	1.76	14.63	9.80
PyrroleS1	1.653	139.87	-0.438	1.56	12.21	10.32
PyrroleS2	1.633	138.07	-0.471	1.74	16.20	9.31
ThiazoleBr1	1.566	134.27	-0.499	1.79	13.74	10.10
ThiazoleBr2	1.558	135.14	-0.508	1.87	15.04	9.78
ThiazoleBr3	1.573	134.00	-0.493	1.76	13.65	10.10
ThiazoleC#N1	1.563	135.39	-0.496	1.79	14.12	9.98
ThiazoleC#N2	1.563	135.72	-0.494	1.82	14.43	9.92
ThiazoleC#N3	1.570	135.92	-0.482	1.82	14.59	9.88
ThiazoleCl1	1.555	134.46	-0.512	1.80	14.73	9.81
ThiazoleCl2	1.557	135.17	-0.510	1.86	15.94	9.51
ThiazoleCl3	1.559	134.48	-0.506	1.82	18.27	8.77
ThiazoleF1	1.557	135.60	-0.495	1.87	14.93	9.82
ThiazoleF2	1.562	135.41	-0.502	1.83	15.16	9.71
ThiazoleF3	1.557	135.67	-0.496	1.87	15.23	9.73
ThiazoleI1	1.561	134.63	-0.505	1.88	15.67	9.61
ThiazoleI2	1.554	134.89	-0.514	1.86	15.01	9.78
ThiazoleI3	1.557	134.55	-0.509	1.89	13.17	10.36
ThiazoleN1	1.510	131.59	-0.584	1.58	12.55	10.24
ThiazoleN2	1.556	134.28	-0.532	1.91	16.15	9.50
ThiazoleN3	1.536	132.94	-0.546	1.84	15.22	9.70
ThiazoleNO2_1	1.571	136.33	-0.482	1.78	13.01	10.30
ThiazoleNO2_2	1.575	136.62	-0.471	1.94	16.56	9.40
ThiazoleNO2_3	1.581	137.21	-0.460	1.68	12.64	10.31
ThiazoleO1	1.539	133.83	-0.524	1.76	14.53	9.83
ThiazoleO2	1.566	135.17	-0.506	1.84	15.18	9.71
ThiazoleO3	1.544	133.97	-0.519	1.78	14.51	9.85
ThiazoleS1	1.526	133.23	-0.549	1.63	11.80	10.52
ThiazoleS2	1.551	134.47	-0.525	2.19	18.15	9.18

ThiazoleS3	1.532	133.58	-0.534	1.65	12.95	10.19
Tio0	1.576	134.62	-0.570	1.24	15.52	9.02
Tio1	1.576	134.22	-0.577	1.42	13.75	9.72
Tio2	1.575	134.22	-0.579	1.71	17.91	8.77
Tio30	1.600	135.82	-0.547	1.23	6.85	11.59
Tio3V2	1.574	134.15	-0.581	1.59	15.48	9.38
Tio430	1.632	134.70	-0.554	1.27	4.52	12.32
Tio4V2	1.568	134.45	-0.585	1.07	5.63	11.79
Tio50	1.600	136.07	-0.567	1.62	14.39	9.73
Tio5V2	1.568	134.52	-0.584	1.26	7.77	11.35
Tio671	1.612	134.39	-0.557	1.73	11.25	10.77
Tio6V2	1.572	134.10	-0.583	1.27	5.81	11.93
Tio7V2	1.575	134.33	-0.578	1.36	10.55	10.61
Tio8V2	1.574	134.25	-0.579	1.59	13.10	10.08
TriazoleBr2	3.412	179.17	0.000	2.15	22.19	7.94
TriazoleC#N1	3.299	178.57	0.001	2.84	29.17	6.54
TriazoleC#N2	3.280	178.46	0.002	1.95	19.29	8.60
TriazoleCl1	3.682	179.85	0.004	2.82	27.15	7.13
TriazoleCl2	3.157	178.10	0.002	1.99	18.81	8.79
TriazoleF1	3.461	179.08	0.001	2.88	31.39	5.92
TriazoleF2	3.139	178.13	0.002	2.16	21.34	8.20
TriazoleI2	3.145	178.27	0.001	1.91	19.44	8.52
TriazoleN1	3.621	179.82	0.000	2.90	31.19	6.00
TriazoleN2	3.034	177.21	0.000	2.30	22.21	8.08
TriazoleNO2_1	3.596	179.57	0.001	2.88	29.32	6.54
TriazoleNO2_2	3.607	179.83	0.004	2.16	24.14	7.37
TriazoleO1	3.341	179.07	0.001	2.86	30.28	6.23
TriazoleO2	1.702	143.87	-0.293	1.56	12.85	10.13
TriazoleS1	3.129	177.84	0.001	2.99	34.16	5.20
TriazoleS2	3.426	179.26	0.001	2.20	22.58	7.87

Table S12: Parameters calculated using the PM6-D3H4 method.

Name	R (Å)	θ (°)	ΔQ	ΔH (kcal/mol)	ΔS (cal/molK)	ΔG (kcal/mol)
Acid0	1.595	136.08	-0.529	1.83	17.69	8.95
Acid1	1.578	134.87	-0.559	1.23	9.61	10.77
Acid2	1.578	133.72	-0.581	1.40	11.00	10.52

Acid30	1.651	139.67	-0.440	1.75	16.43	9.25
Acid3V2	1.570	134.06	-0.585	1.49	16.74	8.90
Acid430	1.740	144.71	-0.353	1.69	13.54	10.05
Acid4V2	1.574	133.84	-0.585	1.53	11.99	10.36
Acid50	1.616	137.12	-0.470	1.58	10.79	10.77
Acid671	1.629	135.47	-0.505	2.13	17.11	9.43
Acid6V2	1.576	134.38	-0.578	1.15	3.44	12.53
Acid7V2	1.573	133.98	-0.586	1.15	9.98	10.57
Acid8V2	1.573	134.03	-0.585	1.60	16.13	9.19
AcidBr1	1.585	135.06	-0.555	1.40	11.76	10.29
AcidBr2	1.578	134.38	-0.573	1.69	15.10	9.59
AcidBr3	1.664	141.52	-0.427	1.79	16.68	9.22
AcidBr4	1.648	139.74	-0.456	1.52	9.37	11.13
AcidBr5	3.275	179.48	0.000	2.84	22.62	8.50
AcidBr6	1.659	138.05	-0.472	1.99	16.69	9.41
AcidCl0	1.600	136.63	-0.516	1.20	9.54	10.76
AcidCl1	1.583	135.12	-0.557	1.42	13.06	9.93
AcidCl2	1.577	134.47	-0.573	1.50	12.51	10.17
AcidCl3	1.657	141.18	-0.435	1.58	13.64	9.92
AcidCl4	1.629	138.73	-0.480	1.43	9.70	10.94
AcidCl5	3.165	179.76	0.000	2.80	26.97	7.16
AcidCl6	1.639	136.68	-0.494	1.70	9.19	11.37
AcidF0	1.600	136.41	-0.517	1.50	13.66	9.83
AcidF1	1.585	135.18	-0.556	1.50	15.98	9.14
AcidF2	1.577	134.43	-0.573	1.28	13.15	9.76
AcidF3	1.673	141.59	-0.415	2.15	17.27	9.41
AcidF4	1.637	138.64	-0.475	1.48	11.68	10.40
AcidF5	3.078	179.58	0.000	2.90	28.95	6.67
AcidF6	1.639	136.80	-0.497	2.12	19.13	8.81
AcidI0	1.600	136.35	-0.520	1.21	9.25	10.86
AcidI1	1.586	135.01	-0.556	1.53	13.16	10.00
AcidI2	1.572	134.63	-0.575	1.31	9.65	10.84
AcidI3	1.659	140.62	-0.436	1.68	11.55	10.64
AcidI4	1.646	139.49	-0.461	1.49	7.25	11.73
AcidI5	3.057	179.84	-0.001	2.85	24.74	7.88
AcidI6	1.653	137.72	-0.484	1.65	13.57	10.01
Acid_Halid3V2	1.573	134.09	-0.583	1.42	11.70	10.34

Acid_Halid4V2	1.574	133.83	-0.584	1.46	12.43	10.15
Acid_Halid5V2	1.570	133.68	-0.588	1.27	8.41	11.17
Acid_Halid6V2	1.566	134.07	-0.591	1.39	12.14	10.17
Acid_Halid7V2	1.572	133.76	-0.587	1.64	18.97	8.38
Acid_Halid8V2	1.574	133.99	-0.585	1.27	10.14	10.65
Acid_Sulf0	1.582	134.97	-0.564	1.63	13.90	9.88
Acid_Sulf1	1.577	133.71	-0.583	1.56	13.23	10.02
Acid_Sulf2	1.575	134.01	-0.582	1.38	6.26	11.91
Acid_Sulf30	1.622	137.15	-0.498	1.73	12.81	10.31
Acid_Sulf3V2	1.573	133.97	-0.584	1.42	8.45	11.30
Acid_Sulf430	1.640	136.76	-0.492	2.21	16.19	9.79
Acid_Sulf4V2	1.571	133.74	-0.587	1.63	14.75	9.63
Acid_Sulf50	1.600	136.73	-0.544	2.01	18.37	8.93
Acid_Sulf5V2	1.574	133.77	-0.587	1.51	12.54	10.17
Acid_Sulf671	1.635	136.06	-0.520	1.46	4.02	12.66
Acid_Sulf6V2	1.574	134.06	-0.584	1.44	11.06	10.54
Acid_Sulf7V2	1.574	134.04	-0.584	0.93	6.64	11.35
Acid_Sulf8V2	1.574	134.06	-0.584	1.26	10.07	10.66
Acid_Tio0	1.600	135.45	-0.531	1.60	14.13	9.79
Acid_Tio1	1.579	134.52	-0.568	1.24	13.04	9.75
Acid_Tio2	1.577	134.26	-0.580	1.35	10.51	10.62
Acid_Tio30	1.647	139.27	-0.462	1.79	14.87	9.76
Acid_Tio3V2	1.574	133.81	-0.585	1.64	15.56	9.40
Acid_Tio430	1.727	143.65	-0.373	1.97	13.77	10.27
Acid_Tio4V2	1.572	133.76	-0.586	1.69	18.68	8.52
Acid_Tio50	1.607	135.35	-0.522	1.34	8.36	11.25
Acid_Tio5V2	1.572	133.66	-0.589	1.45	10.36	10.76
Acid_Tio671	1.626	135.62	-0.522	1.24	6.26	11.78
Acid_Tio6V2	1.572	133.64	-0.588	1.12	8.36	11.03
Acid_Tio7V2	1.572	133.84	-0.588	1.31	10.73	10.51
Acid_Tio8V2	1.573	133.98	-0.586	1.36	10.35	10.67
Alcohol0	1.577	134.31	-0.574	1.69	17.67	8.82
Alcohol1	1.575	134.03	-0.580	1.55	15.59	9.30
Alcohol2	1.575	133.92	-0.584	1.23	10.11	10.61
Alcohol30	1.592	134.66	-0.561	1.92	17.52	9.10
Alcohol3V2	1.573	133.85	-0.585	1.42	11.61	10.36
Alcohol430	1.629	135.07	-0.525	1.97	16.80	9.37

Alcohol4V2	1.573	133.82	-0.586	1.19	6.09	11.77
Alcohol50	1.600	136.42	-0.563	1.34	8.00	11.36
Alcohol5V2	1.573	133.78	-0.586	1.49	13.23	9.95
Alcohol671	1.599	132.39	-0.570	1.31	8.02	11.32
Alcohol6V2	1.573	133.84	-0.586	1.57	10.75	10.77
Alcohol7V2	1.573	133.97	-0.585	1.57	13.85	9.84
Alcohol8V2	1.574	134.07	-0.584	1.44	8.88	11.19
Aldehyde0	1.584	134.98	-0.557	1.30	14.45	9.40
Aldehyde1	1.571	133.94	-0.576	1.33	11.16	10.40
Aldehyde2	1.576	134.08	-0.580	1.41	9.31	11.03
Aldehyde30	1.617	137.24	-0.518	1.63	13.37	10.04
Aldehyde430	1.644	137.29	-0.494	1.79	11.41	10.79
Aldehyde50	1.603	136.39	-0.527	1.03	2.06	12.81
Aldehyde671	1.623	134.71	-0.543	1.52	10.50	10.79
Amide0	1.590	135.25	-0.543	1.50	13.00	10.03
Amide1	1.578	134.72	-0.574	1.22	6.64	11.64
Amide2	1.577	134.35	-0.576	1.49	12.92	10.04
Amide30	1.642	139.39	-0.459	1.81	16.86	9.18
Amide3V2	1.574	133.93	-0.583	1.59	13.04	10.10
Amide430	1.688	140.34	-0.425	1.42	5.58	12.16
Amide4V2	1.575	133.92	-0.584	1.49	11.41	10.49
Amide50	1.618	137.13	-0.511	1.59	12.05	10.40
Amide5V2	1.570	133.60	-0.594	1.07	9.89	10.52
Amide671	1.644	136.06	-0.516	1.67	8.89	11.42
Amide6V2	1.575	134.07	-0.582	1.08	8.01	11.09
Amide7V2	1.574	134.03	-0.584	1.78	17.74	8.89
Amide8V2	1.573	134.03	-0.585	1.39	3.28	12.81
Amine0	1.575	133.87	-0.583	1.62	17.31	8.86
Amine1	1.574	134.02	-0.587	1.49	15.48	9.28
Amine2	1.572	133.62	-0.590	1.77	18.15	8.76
Amine30	1.574	134.36	-0.577	1.45	12.92	9.99
Amine3V2	1.567	134.16	-0.590	1.26	10.77	10.45
Amine430	1.605	133.46	-0.567	1.56	8.52	11.42
Amine4V2	1.573	133.78	-0.587	1.35	9.00	11.06
Amine50	1.588	134.28	-0.574	1.47	10.11	10.86
Amine5V2	1.572	133.84	-0.586	1.13	7.34	11.34
Amine671	1.599	132.17	-0.579	1.23	3.36	12.62

Amine6V2	1.565	134.16	-0.592	1.84	17.81	8.93
Amine7V2	1.574	134.08	-0.584	1.45	11.51	10.41
Amine8V2	1.574	134.03	-0.584	1.47	13.86	9.74
Anhydride0	1.582	136.82	-0.539	1.30	11.63	10.23
Anhydride1	1.580	135.13	-0.561	1.38	10.04	10.78
Anhydride2	1.577	134.31	-0.574	1.76	17.19	9.04
Anhydride30	1.659	140.72	-0.429	1.61	9.26	11.25
Anhydride3V2	1.576	134.25	-0.577	1.16	6.30	11.68
Anhydride430	2.941	179.44	-0.001	2.96	27.98	7.02
Anhydride4V2	1.573	133.94	-0.584	1.40	6.80	11.77
Anhydride50	1.623	136.55	-0.507	1.24	6.18	11.79
Anhydride5V2	1.572	134.09	-0.584	1.54	15.62	9.28
Anhydride671	1.634	136.15	-0.505	1.74	11.79	10.63
Anhydride7V2	1.573	133.96	-0.586	1.10	3.92	12.33
Anhydride8V2	1.574	133.99	-0.585	1.35	11.23	10.40
AzolidineBr1	1.600	136.29	-0.524	1.15	7.60	11.29
AzolidineBr2	1.600	134.32	-0.554	1.47	10.27	10.81
AzolidineC#N1	1.600	138.45	-0.517	1.66	15.61	9.41
AzolidineC#N2	1.600	136.95	-0.556	1.53	12.91	10.08
AzolidineCl1	1.600	136.45	-0.521	0.68	2.18	12.43
AzolidineCl2	1.590	134.79	-0.559	1.65	13.84	9.93
AzolidineF1	1.597	136.01	-0.530	1.76	16.18	9.34
AzolidineF2	1.600	134.28	-0.555	1.26	7.81	11.34
AzolidineI1	1.600	135.81	-0.530	0.98	1.79	12.85
AzolidineI2	1.588	134.64	-0.565	1.54	9.53	11.10
AzolidineN1	1.570	133.70	-0.586	1.70	13.46	10.09
AzolidineN2	1.584	134.07	-0.580	1.61	12.95	10.15
AzolidineNO2_1	1.600	137.25	-0.517	1.31	7.69	11.41
AzolidineNO2_2	1.600	135.63	-0.541	1.30	8.14	11.27
AzolidineO1	1.584	134.96	-0.557	1.60	13.72	9.91
AzolidineS1	1.600	134.22	-0.564	1.32	9.81	10.80
AzolidineS2	1.587	134.75	-0.558	1.62	11.48	10.59
Br0	1.601	135.84	-0.543	1.55	15.08	9.45
Br1	1.582	134.67	-0.566	1.12	9.54	10.68
Br2	1.576	134.09	-0.578	1.56	14.83	9.54
Br3	1.664	139.86	-0.455	2.00	16.99	9.33
Br4	1.631	138.06	-0.493	1.43	10.02	10.85

Br5	3.224	178.55	0.000	2.78	31.12	5.90
Br6	1.626	135.64	-0.520	1.65	13.21	10.11
Cl0	1.603	136.76	-0.519	0.87	8.21	10.82
Cl1	1.584	134.28	-0.570	1.33	12.49	10.01
Cl2	1.578	134.32	-0.575	1.37	10.47	10.65
Cl3	1.652	139.61	-0.450	1.71	16.83	9.09
Cl4	1.635	138.70	-0.477	1.64	14.35	9.76
Cl5	3.203	179.41	-0.007	2.65	24.93	7.62
Cl6	1.644	137.12	-0.498	1.55	9.81	11.03
Cyan0	1.600	136.90	-0.521	1.10	10.03	10.51
Cyan1	1.578	134.93	-0.570	1.54	17.54	8.71
Cyan2	1.578	134.05	-0.581	1.53	16.67	8.96
Cyan30	1.642	139.57	-0.464	1.79	15.76	9.49
Cyan3V2	1.579	134.21	-0.580	1.38	13.65	9.71
Cyan430	1.722	143.30	-0.369	2.54	21.39	8.56
Cyan4V2	1.568	134.00	-0.588	1.37	11.99	10.19
Cyan50	1.612	138.11	-0.512	1.65	12.41	10.35
Cyan5V2	1.575	133.92	-0.584	1.51	14.25	9.66
Cyan671	1.631	135.82	-0.510	1.69	14.90	9.65
Cyan6V2	1.573	133.81	-0.587	1.60	12.55	10.26
Cyan7V2	1.575	134.17	-0.582	1.56	11.00	10.68
Cyan8V2	1.573	133.87	-0.586	1.65	15.60	9.40
Ester0	1.600	135.18	-0.531	1.22	7.68	11.33
Ester1	1.582	134.84	-0.563	1.58	15.14	9.47
Ester2	1.577	134.29	-0.575	1.34	9.82	10.81
Ester30	1.638	138.96	-0.469	1.87	15.43	9.67
Ester3V2	1.570	134.21	-0.586	1.17	7.11	11.45
Ester430	1.708	141.33	-0.412	1.94	12.98	10.47
Ester4V2	1.575	133.85	-0.584	1.60	15.81	9.28
Ester50	1.600	136.24	-0.540	1.25	5.77	11.93
Ester5V2	1.574	133.87	-0.585	1.34	12.07	10.14
Ester671	1.635	136.01	-0.515	1.53	5.29	12.36
Ester6V2	1.574	134.07	-0.584	1.48	15.82	9.16
Ester7V2	1.574	133.98	-0.584	1.37	8.19	11.33
Ester8V2	1.573	133.99	-0.585	1.59	14.89	9.55
Ether0	1.600	135.70	-0.530	1.27	10.27	10.61
Ether1	1.576	134.18	-0.577	1.11	7.87	11.16

Ether2	1.572	134.36	-0.582	1.27	8.71	11.07
Ether30	1.609	136.13	-0.523	1.70	14.11	9.89
Ether3V2	1.575	134.07	-0.584	1.43	11.37	10.44
Ether430	1.627	135.39	-0.513	1.54	10.85	10.71
Ether4V2	1.575	134.03	-0.585	1.34	11.05	10.44
Ether50	1.600	136.25	-0.539	1.18	6.35	11.69
Ether5V2	1.569	134.21	-0.589	1.34	12.12	10.13
Ether671	1.623	135.04	-0.540	2.57	26.63	7.03
Ether6V2	1.572	133.84	-0.587	1.19	6.18	11.74
Ether7V2	1.573	133.97	-0.586	1.52	12.84	10.09
Ether8V2	1.574	133.99	-0.584	1.45	10.52	10.71
F0	1.596	136.12	-0.529	1.91	17.68	9.04
F1	1.579	134.27	-0.576	1.25	11.59	10.19
F2	1.580	134.33	-0.574	1.19	9.94	10.62
F3	1.657	140.19	-0.437	1.76	14.80	9.75
F4	1.618	136.92	-0.508	1.71	14.59	9.76
F5	2.985	179.33	-0.001	2.81	28.32	6.76
F6	1.621	134.71	-0.536	1.78	14.15	9.96
Halid2V2	1.578	134.24	-0.576	1.43	14.09	9.63
Halid3V2	1.574	133.99	-0.583	1.47	13.39	9.87
Halid4V2	1.571	133.90	-0.593	1.48	12.57	10.13
Halid5V2	1.572	133.73	-0.587	1.38	12.80	9.97
Halid6V2	1.573	133.85	-0.586	1.44	12.67	10.07
Halid7V2	1.573	133.80	-0.587	1.37	10.63	10.60
Halid8V2	1.573	133.93	-0.585	1.68	15.55	9.44
I0	1.600	135.77	-0.541	0.97	7.78	11.05
I1	1.575	134.66	-0.567	1.21	11.29	10.24
I2	1.576	134.25	-0.577	1.52	14.39	9.63
I3	1.650	139.24	-0.465	1.78	16.32	9.31
I4	1.626	137.86	-0.498	1.24	7.76	11.33
I5	1.732	142.80	-0.377	1.95	12.29	10.69
I6	1.629	136.00	-0.524	2.21	20.90	8.38
ImidazoleBr1	3.318	178.88	0.000	2.96	33.23	5.45
ImidazoleC#N1	3.457	178.58	0.000	2.93	30.18	6.33
ImidazoleC#N2	3.442	178.72	0.000	2.83	28.47	6.74
ImidazoleF1	3.349	179.01	0.000	2.82	30.45	6.14
ImidazoleF2	3.336	178.95	0.000	2.90	32.91	5.49

ImidazoleI1	3.349	179.00	0.000	2.89	29.10	6.62
ImidazoleN1	1.654	139.78	-0.429	1.78	15.37	9.60
ImidazoleN2	1.675	140.97	-0.395	1.88	14.80	9.87
ImidazoleNO2_1	3.378	178.25	0.000	2.90	29.77	6.43
ImidazoleNO2_2	3.471	178.49	0.000	2.89	30.86	6.09
ImidazoleO1	1.663	140.70	-0.386	1.53	13.27	9.98
ImidazoleO2	1.681	141.77	-0.363	1.58	12.59	10.22
ImidazoleS1	3.288	178.85	0.000	2.84	28.65	6.70
ImidazoleS2	3.360	179.02	0.000	2.70	28.19	6.69
Ketone3V2	1.573	133.95	-0.584	1.58	14.61	9.62
Ketone4V2	1.569	133.99	-0.589	1.35	11.73	10.25
Ketone5V2	1.573	133.62	-0.593	1.22	11.87	10.08
Ketone6V2	1.573	133.87	-0.585	1.11	7.61	11.24
Ketone7V2	1.572	133.72	-0.588	1.00	2.63	12.62
Ketone8V2	1.573	133.93	-0.585	1.43	9.05	11.13
Nitro0	1.600	135.60	-0.540	1.04	8.72	10.84
Nitro1	1.582	134.57	-0.567	1.41	12.00	10.23
Nitro3	1.600	134.22	-0.589	1.00	5.69	11.70
Nitro30	1.623	137.86	-0.502	1.57	10.85	10.74
Nitro430	1.662	138.84	-0.447	2.15	15.05	10.06
Nitro4V2	1.569	134.21	-0.588	1.11	7.85	11.17
Nitro50	1.611	136.80	-0.526	1.75	16.80	9.14
Nitro5V2	1.567	134.07	-0.590	1.39	10.54	10.64
Nitro671	1.613	134.07	-0.546	1.97	19.96	8.42
Nitro6V2	1.573	133.99	-0.586	1.57	18.18	8.55
Nitro7V2	1.574	134.03	-0.585	1.42	9.87	10.88
Nitro8V2	1.574	133.92	-0.585	1.68	16.48	9.17
PiperidineBr1	1.614	135.76	-0.525	1.76	12.67	10.38
PiperidineBr2	1.600	134.03	-0.563	1.52	10.29	10.86
PiperidineBr3	1.588	133.70	-0.576	1.68	12.52	10.35
PiperidineC#N1	1.610	136.91	-0.519	1.81	14.15	10.00
PiperidineC#N2	1.600	136.11	-0.564	1.66	12.26	10.40
PiperidineC#N3	1.600	135.83	-0.549	1.49	10.65	10.71
PiperidineCl1	1.600	136.62	-0.546	1.31	8.59	11.15
PiperidineCl2	1.600	135.62	-0.545	1.28	8.04	11.28
PiperidineCl3	1.600	133.59	-0.573	1.49	10.30	10.82
PiperidineF1	1.600	135.92	-0.532	1.33	8.97	11.06

PiperidineF2	1.600	134.11	-0.568	1.58	11.75	10.48
PiperidineF3	1.589	134.78	-0.566	1.77	14.04	9.99
PiperidineI1	1.611	136.77	-0.517	1.74	12.79	10.33
PiperidineI2	1.596	135.40	-0.552	1.73	12.50	10.40
PiperidineI3	1.587	134.92	-0.563	1.82	15.30	9.66
PiperidineN1	1.600	134.84	-0.549	1.47	10.75	10.67
PiperidineN2	1.587	134.62	-0.570	1.78	14.37	9.89
PiperidineN3	1.585	134.54	-0.573	1.84	15.63	9.58
PiperidineNO2_1	1.620	136.85	-0.505	1.74	12.41	10.44
PiperidineNO2_2	1.600	135.52	-0.550	1.58	8.78	11.36
PiperidineNO2_3	1.600	136.92	-0.555	1.50	10.08	10.89
PiperidineO1	1.600	134.90	-0.538	1.33	10.07	10.73
PiperidineO2	1.593	135.03	-0.559	1.58	14.73	9.59
PiperidineO3	1.582	135.06	-0.564	1.66	12.49	10.34
PiperidineS1	1.600	135.77	-0.558	1.46	10.65	10.69
PiperidineS2	1.584	133.35	-0.584	1.86	14.11	10.05
PiperidineS3	1.600	133.40	-0.577	1.43	9.23	11.08
PyrazineBr1	1.664	138.73	-0.385	1.93	15.15	9.81
PyrazineBr2	1.618	138.84	-0.432	1.93	15.66	9.66
PyrazineC#N1	1.640	140.11	-0.395	1.85	15.58	9.60
PyrazineC#N2	1.622	139.65	-0.419	1.93	15.32	9.76
PyrazineCl1	1.619	137.95	-0.429	1.89	14.63	9.93
PyrazineCl2	1.612	138.64	-0.440	1.90	15.01	9.83
PyrazineF1	1.623	139.99	-0.409	1.92	15.64	9.66
PyrazineF2	1.616	138.91	-0.433	1.90	15.84	9.58
PyrazineN1	1.543	132.53	-0.538	1.70	13.95	9.94
PyrazineN2	1.590	135.97	-0.498	1.97	16.23	9.53
PyrazineNO2_1	1.672	142.66	-0.353	1.82	12.02	10.64
PyrazineNO2_2	1.645	141.09	-0.386	1.94	13.54	10.31
PyrazineO1	1.587	136.53	-0.460	1.83	15.56	9.59
PyrazineO2	1.609	137.90	-0.453	1.88	16.41	9.39
PyrazineS1	1.571	136.30	-0.470	1.49	10.36	10.80
PyrazineS2	1.605	137.77	-0.457	1.85	15.70	9.57
PyrazoleBr1	3.343	179.11	0.000	2.92	30.59	6.20
PyrazoleBr2	3.221	178.85	0.000	2.96	30.84	6.16
PyrazoleC#N1	3.375	178.62	0.000	2.97	33.73	5.32
PyrazoleC#N2	3.415	178.80	0.000	2.78	27.07	7.10

PyrazoleCl1	3.335	179.16	0.000	2.92	30.00	6.37
PyrazoleCl2	3.364	179.64	0.000	2.92	31.30	5.99
PyrazoleF1	3.330	179.56	0.000	2.82	32.79	5.45
PyrazoleF2	3.351	179.63	0.000	2.79	28.59	6.67
PyrazoleI1	3.589	179.91	0.000	2.86	29.37	6.50
PyrazoleI2	3.250	178.96	0.000	2.96	30.29	6.33
PyrazoleN1	1.645	138.18	-0.476	2.02	15.78	9.71
PyrazoleN2	1.656	139.17	-0.450	1.79	13.63	10.12
PyrazoleNO2_1	3.365	178.69	0.000	2.84	31.14	5.96
PyrazoleNO2_2	3.471	178.56	0.000	2.65	25.62	7.41
PyrazoleO1	1.686	141.66	-0.397	1.77	14.70	9.79
PyrazoleO2	1.719	143.51	-0.362	1.86	14.37	9.98
PyrazoleS1	3.576	179.68	0.000	2.76	30.00	6.22
PyrazoleS2	1.678	141.46	-0.397	1.91	16.45	9.41
PyridineBr1	1.608	134.57	-0.464	1.81	13.18	10.29
PyridineBr2	1.579	135.86	-0.497	1.87	15.06	9.78
PyridineBr3	1.576	135.61	-0.503	1.87	14.87	9.83
PyridineC#N1	1.593	136.42	-0.468	1.84	14.50	9.92
PyridineC#N2	1.583	136.59	-0.484	1.89	17.37	9.12
PyridineC#N3	1.583	136.21	-0.491	1.89	16.36	9.41
PyridineCl1	1.585	134.79	-0.488	1.78	13.67	10.10
PyridineCl2	1.579	135.85	-0.498	1.86	15.43	9.66
PyridineCl3	1.572	135.34	-0.509	1.85	14.91	9.81
PyridineF1	1.579	136.59	-0.479	1.85	14.90	9.81
PyridineF2	1.581	135.96	-0.495	1.87	16.46	9.37
PyridineF3	1.570	135.49	-0.506	1.84	15.32	9.67
PyridineI1	1.565	138.05	-0.498	1.60	10.32	10.93
PyridineI2	1.574	135.58	-0.505	1.84	13.99	10.07
PyridineI3	1.574	135.54	-0.508	1.85	14.30	9.98
PyridineN1	1.528	130.84	-0.575	1.82	14.95	9.76
PyridineN2	1.567	134.21	-0.538	1.86	15.41	9.67
PyridineN3	1.521	131.68	-0.600	1.84	13.77	10.14
PyridineNO2_1	1.618	139.07	-0.426	2.03	16.77	9.43
PyridineNO2_2	1.600	137.68	-0.454	1.90	14.80	9.89
PyridineNO2_3	1.591	137.35	-0.473	1.82	13.07	10.32
PyridineO1	1.543	132.43	-0.500	1.58	12.24	10.33
PyridineO2	1.575	135.20	-0.514	1.83	14.56	9.89

PyridineO3	1.550	133.85	-0.543	1.79	14.70	9.81
PyridineS1	1.568	133.65	-0.495	1.80	15.48	9.58
PyridineS3	1.562	134.53	-0.528	1.78	14.68	9.81
PyrimidineBr1	1.617	137.44	-0.422	1.84	15.00	9.77
PyrimidineBr2	1.638	140.37	-0.387	1.98	16.45	9.48
PyrimidineBr3	1.586	137.83	-0.460	1.90	16.80	9.29
PyrimidineC#N1	1.625	138.92	-0.408	1.94	16.76	9.34
PyrimidineC#N2	1.618	139.06	-0.411	2.04	17.47	9.23
PyrimidineC#N3	1.607	138.76	-0.430	1.91	16.65	9.35
PyrimidineCl1	1.604	136.73	-0.444	1.85	15.88	9.52
PyrimidineCl2	1.603	137.88	-0.435	1.85	15.98	9.49
PyrimidineCl3	1.596	137.77	-0.452	1.93	18.48	8.82
PyrimidineF1	1.600	138.93	-0.427	1.91	17.21	9.18
PyrimidineF2	1.598	138.72	-0.432	1.89	14.78	9.88
PyrimidineF3	1.599	137.97	-0.448	1.90	17.09	9.21
PyrimidineI1	1.603	138.00	-0.440	1.93	16.21	9.50
PyrimidineI2	1.610	138.33	-0.428	1.84	13.20	10.31
PyrimidineI3	1.595	137.79	-0.453	1.88	15.81	9.56
PyrimidineN1	1.541	132.73	-0.534	1.74	16.98	9.08
PyrimidineN2	1.537	132.36	-0.540	1.51	12.37	10.22
PyrimidineN3	1.580	135.84	-0.497	1.84	14.14	10.03
PyrimidineNO2_1	1.648	141.60	-0.370	2.06	17.91	9.12
PyrimidineNO2_2	1.645	141.68	-0.372	2.09	15.82	9.77
PyrimidineNO2_3	1.623	140.05	-0.403	2.00	17.38	9.22
PyrimidineO1	1.574	136.11	-0.470	1.85	14.32	9.98
PyrimidineO2	1.549	133.87	-0.466	1.63	12.45	10.32
PyrimidineO3	1.587	137.21	-0.471	1.75	14.46	9.84
PyrimidineS1	1.556	135.16	-0.487	1.66	12.49	10.34
PyrimidineS2	1.578	135.24	-0.460	1.86	14.71	9.87
PyrimidineS3	1.590	137.27	-0.462	1.89	14.98	9.82
PyrroleBr1	1.665	140.10	-0.424	1.95	19.68	8.48
PyrroleBr2	1.655	139.59	-0.435	1.91	15.86	9.58
PyrroleC#N1	1.683	141.76	-0.394	1.97	16.65	9.40
PyrroleC#N2	1.699	142.48	-0.378	2.03	16.60	9.48
PyrroleCl2	1.655	139.58	-0.438	1.85	14.70	9.87
PyrroleF1	1.673	141.02	-0.407	1.91	15.97	9.55
PyrroleF2	1.657	139.74	-0.435	1.80	14.12	9.99

PyrroleI1	1.657	139.59	-0.435	1.86	14.93	9.81
PyrroleI2	1.656	139.65	-0.435	1.92	15.74	9.63
PyrroleN1	1.637	138.43	-0.465	1.60	12.23	10.35
PyrroleN2	1.605	135.80	-0.528	1.81	15.00	9.74
PyrroleNO2_1	3.384	178.45	0.000	2.98	31.85	5.88
PyrroleNO2_2	3.676	179.68	0.000	2.91	31.16	6.02
PyrroleO1	1.626	137.54	-0.453	1.30	10.41	10.60
PyrroleO2	1.627	137.63	-0.483	1.77	14.99	9.70
PyrroleS1	1.665	140.52	-0.423	1.90	15.13	9.79
PyrroleS2	1.636	138.18	-0.468	1.71	14.86	9.68
ThiazoleBr1	1.567	134.37	-0.497	1.81	14.15	9.99
ThiazoleBr2	1.558	135.09	-0.508	1.87	15.20	9.74
ThiazoleBr3	1.573	134.01	-0.493	1.79	13.69	10.11
ThiazoleC#N1	1.563	135.37	-0.496	1.84	15.60	9.59
ThiazoleC#N2	1.564	135.70	-0.493	1.84	14.68	9.87
ThiazoleC#N3	1.570	135.85	-0.483	1.84	14.55	9.90
ThiazoleCl1	1.554	134.47	-0.513	1.75	12.98	10.28
ThiazoleCl2	1.555	135.34	-0.509	1.86	15.83	9.54
ThiazoleCl3	1.559	134.48	-0.506	1.82	16.02	9.44
ThiazoleF1	1.558	135.59	-0.494	1.91	15.46	9.70
ThiazoleF2	1.563	135.37	-0.501	1.85	15.46	9.64
ThiazoleF3	1.557	135.71	-0.495	1.89	15.39	9.70
ThiazoleI1	1.561	134.95	-0.503	1.83	14.64	9.87
ThiazoleI3	1.566	134.83	-0.497	1.94	16.88	9.31
ThiazoleN1	1.510	131.54	-0.583	1.54	12.26	10.28
ThiazoleN2	1.556	134.07	-0.537	1.78	15.14	9.66
ThiazoleN3	1.532	132.26	-0.551	1.74	13.99	9.97
ThiazoleNO2_1	1.567	136.19	-0.485	1.96	15.79	9.65
ThiazoleNO2_2	1.573	136.61	-0.472	1.94	15.78	9.63
ThiazoleNO2_3	1.582	137.15	-0.459	1.82	14.93	9.77
ThiazoleO1	1.539	133.89	-0.524	1.76	14.54	9.83
ThiazoleO2	1.560	135.30	-0.512	1.85	15.74	9.56
ThiazoleO3	1.542	133.98	-0.522	1.94	15.73	9.65
ThiazoleS1	1.524	133.13	-0.548	1.66	14.60	9.70
ThiazoleS2	1.552	134.48	-0.525	2.09	16.43	9.59
ThiazoleS3	1.537	133.73	-0.528	1.66	15.15	9.54
Tio0	1.575	134.26	-0.576	1.25	11.47	10.23

Tio1	1.570	134.21	-0.585	1.31	11.08	10.41
Tio2	1.573	133.94	-0.585	1.63	15.53	9.41
Tio30	1.600	135.55	-0.550	1.23	8.61	11.06
Tio3V2	1.573	133.84	-0.587	1.42	11.18	10.49
Tio430	1.630	134.09	-0.552	1.68	9.02	11.39
Tio4V2	1.573	133.66	-0.588	1.54	15.81	9.23
Tio50	1.600	135.92	-0.570	1.44	10.71	10.64
Tio5V2	1.574	133.83	-0.586	1.44	10.66	10.66
Tio671	1.615	134.11	-0.559	1.15	1.76	13.03
Tio6V2	1.573	133.93	-0.585	1.75	17.82	8.83
Tio7V2	1.574	134.03	-0.585	1.53	13.46	9.92
Tio8V2	1.573	133.97	-0.585	1.81	16.19	9.38
TriazoleBr2	3.429	178.28	0.001	2.33	25.12	7.24
TriazoleC#N1	3.444	178.45	0.001	2.93	33.09	5.47
TriazoleC#N2	3.508	178.50	0.001	1.90	15.47	9.69
TriazoleCl1	4.018	179.89	0.003	2.79	27.86	6.89
TriazoleCl2	3.225	177.82	0.000	1.85	16.74	9.26
TriazoleF1	3.400	178.42	0.000	2.86	29.29	6.52
TriazoleF2	3.660	179.42	0.000	2.04	20.70	8.27
TriazoleI2	3.409	178.60	0.000	1.62	13.21	10.09
TriazoleN1	3.379	178.77	0.000	2.92	30.63	6.19
TriazoleN2	3.353	178.25	0.000	2.19	21.55	8.17
TriazoleNO2_1	3.541	178.25	0.000	3.05	33.45	5.48
TriazoleNO2_2	3.466	177.94	0.000	2.28	24.99	7.23
TriazoleO1	3.348	178.70	0.000	2.95	31.67	5.91
TriazoleO2	1.703	143.77	-0.293	1.62	13.37	10.04
TriazoleS1	3.960	179.80	0.003	2.98	31.05	6.12
TriazoleS2	3.336	177.89	0.001	2.04	20.09	8.45

Table S13: Parameters calculated using the PM6-D3H4X method.

Name	R (Å)	θ (°)	ΔQ	ΔH (kcal/mol)	ΔS (cal/molK)	ΔG (kcal/mol)
Acid0	1.595	136.08	-0.529	1.83	17.69	8.95
Acid1	1.578	134.87	-0.559	1.23	9.61	10.77
Acid2	1.578	133.72	-0.581	1.40	11.00	10.52
Acid30	1.651	139.67	-0.440	1.75	16.43	9.25
Acid3V2	1.570	134.06	-0.585	1.49	16.74	8.90

Acid430	1.740	144.71	-0.353	1.69	13.54	10.05
Acid4V2	1.574	133.84	-0.585	1.53	11.99	10.36
Acid50	1.616	137.12	-0.470	1.58	10.79	10.77
Acid671	1.629	135.47	-0.505	2.13	17.11	9.43
Acid6V2	1.576	134.38	-0.578	1.15	3.44	12.53
Acid7V2	1.573	133.98	-0.586	1.15	9.98	10.57
Acid8V2	1.573	134.03	-0.585	1.60	16.13	9.19
AcidBr1	1.585	135.09	-0.555	1.32	11.45	10.31
AcidBr2	1.578	134.36	-0.574	1.68	15.93	9.33
AcidBr3	1.675	141.38	-0.418	1.63	14.78	9.62
AcidBr4	1.649	139.72	-0.457	1.28	5.22	12.13
AcidBr5	3.301	179.27	0.000	3.02	31.13	6.14
AcidBr6	1.660	138.21	-0.470	1.51	13.47	9.90
AcidCl0	1.600	136.78	-0.515	1.30	10.23	10.65
AcidCl1	1.589	135.16	-0.551	1.47	14.47	9.56
AcidCl2	1.577	134.26	-0.574	1.46	12.33	10.18
AcidCl3	1.665	141.28	-0.427	1.63	14.79	9.62
AcidCl4	1.632	138.97	-0.475	1.27	6.66	11.68
AcidCl5	3.029	179.81	-0.001	2.98	24.92	7.95
AcidCl6	1.673	137.22	-0.471	1.37	1.65	13.28
AcidF0	1.600	136.41	-0.517	1.50	13.66	9.83
AcidF1	1.585	135.18	-0.556	1.50	15.98	9.14
AcidF2	1.577	134.43	-0.573	1.28	13.15	9.76
AcidF3	1.673	141.59	-0.415	2.15	17.27	9.41
AcidF4	1.637	138.64	-0.475	1.48	11.68	10.40
AcidF5	3.078	179.58	0.000	2.90	28.95	6.67
AcidF6	1.639	136.80	-0.497	2.12	19.13	8.81
AcidI0	1.600	136.36	-0.518	1.30	10.13	10.68
AcidI1	1.587	135.13	-0.554	1.47	14.18	9.65
AcidI2	1.578	134.49	-0.574	1.46	12.80	10.04
AcidI3	1.659	140.75	-0.436	1.54	9.98	10.97
AcidI4	1.643	139.39	-0.463	1.59	11.20	10.65
AcidI5	3.042	179.83	-0.001	2.95	29.66	6.51
AcidI6	1.653	137.79	-0.484	2.00	18.99	8.74
Acid_Halid3V2	1.573	134.09	-0.583	1.42	11.70	10.34
Acid_Halid4V2	1.574	133.83	-0.584	1.46	12.43	10.15
Acid_Halid5V2	1.570	133.68	-0.588	1.27	8.41	11.17

Acid_Halid6V2	1.566	134.07	-0.591	1.39	12.14	10.17
Acid_Halid7V2	1.572	133.76	-0.587	1.64	18.97	8.38
Acid_Halid8V2	1.574	133.99	-0.585	1.27	10.14	10.65
Acid_Sulf0	1.582	134.97	-0.564	1.63	13.90	9.88
Acid_Sulf1	1.577	133.71	-0.583	1.56	13.23	10.02
Acid_Sulf2	1.575	134.01	-0.582	1.38	6.26	11.91
Acid_Sulf30	1.622	137.15	-0.498	1.73	12.81	10.31
Acid_Sulf3V2	1.573	133.97	-0.584	1.42	8.45	11.30
Acid_Sulf430	1.640	136.76	-0.492	2.21	16.19	9.79
Acid_Sulf4V2	1.571	133.74	-0.587	1.63	14.75	9.63
Acid_Sulf50	1.600	136.73	-0.544	2.01	18.37	8.93
Acid_Sulf5V2	1.574	133.77	-0.587	1.51	12.54	10.17
Acid_Sulf671	1.635	136.06	-0.520	1.46	4.02	12.66
Acid_Sulf6V2	1.574	134.06	-0.584	1.44	11.06	10.54
Acid_Sulf7V2	1.574	134.04	-0.584	0.93	6.64	11.35
Acid_Sulf8V2	1.574	134.06	-0.584	1.26	10.07	10.66
Acid_Tio0	1.600	135.45	-0.531	1.60	14.13	9.79
Acid_Tio1	1.579	134.52	-0.568	1.24	13.04	9.75
Acid_Tio2	1.577	134.26	-0.580	1.35	10.51	10.62
Acid_Tio30	1.647	139.27	-0.462	1.79	14.87	9.76
Acid_Tio3V2	1.574	133.81	-0.585	1.64	15.56	9.40
Acid_Tio430	1.727	143.65	-0.373	1.97	13.77	10.27
Acid_Tio4V2	1.572	133.76	-0.586	1.69	18.68	8.52
Acid_Tio50	1.607	135.35	-0.522	1.34	8.36	11.25
Acid_Tio5V2	1.572	133.66	-0.589	1.45	10.36	10.76
Acid_Tio671	1.626	135.62	-0.522	1.24	6.26	11.78
Acid_Tio6V2	1.572	133.64	-0.588	1.12	8.36	11.03
Acid_Tio7V2	1.572	133.84	-0.588	1.31	10.73	10.51
Acid_Tio8V2	1.573	133.98	-0.586	1.36	10.35	10.67
Alcohol0	1.577	134.31	-0.574	1.69	17.67	8.82
Alcohol1	1.575	134.03	-0.580	1.55	15.59	9.30
Alcohol2	1.575	133.92	-0.584	1.23	10.11	10.61
Alcohol30	1.592	134.66	-0.561	1.92	17.52	9.10
Alcohol3V2	1.573	133.85	-0.585	1.42	11.61	10.36
Alcohol430	1.629	135.07	-0.525	1.97	16.80	9.37
Alcohol4V2	1.573	133.82	-0.586	1.19	6.09	11.77
Alcohol50	1.600	136.42	-0.563	1.34	8.00	11.36

Alcohol5V2	1.573	133.78	-0.586	1.49	13.23	9.95
Alcohol671	1.599	132.39	-0.570	1.31	8.02	11.32
Alcohol6V2	1.573	133.84	-0.586	1.57	10.75	10.77
Alcohol7V2	1.573	133.97	-0.585	1.57	13.85	9.84
Alcohol8V2	1.574	134.07	-0.584	1.44	8.88	11.19
Aldehyde0	1.584	134.98	-0.557	1.30	14.45	9.40
Aldehyde1	1.571	133.94	-0.576	1.33	11.16	10.40
Aldehyde2	1.576	134.08	-0.580	1.41	9.31	11.03
Aldehyde30	1.617	137.24	-0.518	1.63	13.37	10.04
Aldehyde430	1.644	137.29	-0.494	1.79	11.41	10.79
Aldehyde50	1.603	136.39	-0.527	1.03	2.06	12.81
Aldehyde671	1.623	134.71	-0.543	1.52	10.50	10.79
Amide0	1.590	135.25	-0.543	1.50	13.00	10.03
Amide1	1.578	134.72	-0.574	1.22	6.64	11.64
Amide2	1.577	134.35	-0.576	1.49	12.92	10.04
Amide30	1.642	139.39	-0.459	1.81	16.86	9.18
Amide3V2	1.574	133.93	-0.583	1.59	13.04	10.10
Amide430	1.688	140.34	-0.425	1.42	5.58	12.16
Amide4V2	1.575	133.92	-0.584	1.49	11.41	10.49
Amide50	1.618	137.13	-0.511	1.59	12.05	10.40
Amide5V2	1.570	133.60	-0.594	1.07	9.89	10.52
Amide671	1.644	136.06	-0.516	1.67	8.89	11.42
Amide6V2	1.575	134.07	-0.582	1.08	8.01	11.09
Amide7V2	1.574	134.03	-0.584	1.78	17.74	8.89
Amide8V2	1.573	134.03	-0.585	1.39	3.28	12.81
Amine0	1.575	133.87	-0.583	1.62	17.31	8.86
Amine1	1.574	134.02	-0.587	1.49	15.48	9.28
Amine2	1.572	133.62	-0.590	1.77	18.15	8.76
Amine30	1.574	134.36	-0.577	1.45	12.92	9.99
Amine3V2	1.567	134.16	-0.590	1.26	10.77	10.45
Amine430	1.605	133.46	-0.567	1.56	8.52	11.42
Amine4V2	1.573	133.78	-0.587	1.35	9.00	11.06
Amine50	1.588	134.28	-0.574	1.47	10.11	10.86
Amine5V2	1.572	133.84	-0.586	1.13	7.34	11.34
Amine671	1.599	132.17	-0.579	1.23	3.36	12.62
Amine6V2	1.565	134.16	-0.592	1.84	17.81	8.93
Amine7V2	1.574	134.08	-0.584	1.45	11.51	10.41

Amine8V2	1.574	134.03	-0.584	1.47	13.86	9.74
Anhydride0	1.582	136.82	-0.539	1.30	11.63	10.23
Anhydride1	1.580	135.13	-0.561	1.38	10.04	10.78
Anhydride2	1.577	134.31	-0.574	1.76	17.19	9.04
Anhydride30	1.659	140.72	-0.429	1.61	9.26	11.25
Anhydride3V2	1.576	134.25	-0.577	1.16	6.30	11.68
Anhydride430	2.941	179.44	-0.001	2.96	27.98	7.02
Anhydride4V2	1.573	133.94	-0.584	1.40	6.80	11.77
Anhydride50	1.623	136.55	-0.507	1.24	6.18	11.79
Anhydride5V2	1.572	134.09	-0.584	1.54	15.62	9.28
Anhydride671	1.634	136.15	-0.505	1.74	11.79	10.63
Anhydride7V2	1.573	133.96	-0.586	1.10	3.92	12.33
Anhydride8V2	1.574	133.99	-0.585	1.35	11.23	10.40
AzolidineBr1	1.600	137.24	-0.539	0.86	2.30	12.57
AzolidineBr2	1.600	134.33	-0.554	1.42	9.44	11.00
AzolidineC#N1	1.600	138.45	-0.517	1.66	15.61	9.41
AzolidineC#N2	1.600	136.95	-0.556	1.53	12.91	10.08
AzolidineCl1	1.600	136.53	-0.517	0.79	3.74	12.07
AzolidineCl2	1.590	134.79	-0.559	1.66	14.06	9.87
AzolidineF1	1.597	136.01	-0.530	1.76	16.18	9.34
AzolidineF2	1.600	134.28	-0.555	1.26	7.81	11.34
AzolidineI1	1.600	135.78	-0.530	1.06	3.92	12.30
AzolidineI2	1.588	134.67	-0.564	1.58	12.45	10.27
AzolidineN1	1.570	133.70	-0.586	1.70	13.46	10.09
AzolidineN2	1.584	134.07	-0.580	1.61	12.95	10.15
AzolidineNO2_1	1.600	137.25	-0.517	1.31	7.69	11.41
AzolidineNO2_2	1.600	135.63	-0.541	1.30	8.14	11.27
AzolidineO1	1.584	134.96	-0.557	1.60	13.72	9.91
AzolidineS1	1.600	134.22	-0.564	1.32	9.81	10.80
AzolidineS2	1.587	134.75	-0.558	1.62	11.48	10.59
Br0	1.600	138.13	-0.543	1.37	13.54	9.73
Br1	1.581	134.84	-0.561	1.23	11.72	10.14
Br2	1.576	134.14	-0.578	1.53	14.58	9.58
Br3	1.655	139.69	-0.450	1.61	12.33	10.33
Br4	1.635	138.72	-0.477	1.46	10.92	10.60
Br5	3.136	179.10	0.000	2.94	33.94	5.23
Br6	1.627	135.75	-0.519	1.48	5.82	12.14

Cl0	1.604	136.91	-0.512	0.91	7.70	11.01
Cl1	1.584	134.28	-0.570	1.36	12.78	9.95
Cl2	1.578	134.32	-0.575	1.37	10.47	10.65
Cl3	1.651	139.97	-0.444	1.60	14.05	9.81
Cl4	1.636	138.86	-0.471	1.50	13.82	9.78
Cl5	3.186	178.72	-0.005	2.71	23.59	8.08
Cl6	1.646	137.03	-0.498	1.54	9.59	11.08
Cyan0	1.600	136.90	-0.521	1.10	10.03	10.51
Cyan1	1.578	134.93	-0.570	1.54	17.54	8.71
Cyan2	1.578	134.05	-0.581	1.53	16.67	8.96
Cyan30	1.642	139.57	-0.464	1.79	15.76	9.49
Cyan3V2	1.579	134.21	-0.580	1.38	13.65	9.71
Cyan430	1.722	143.30	-0.369	2.54	21.39	8.56
Cyan4V2	1.568	134.00	-0.588	1.37	11.99	10.19
Cyan50	1.612	138.11	-0.512	1.65	12.41	10.35
Cyan5V2	1.575	133.92	-0.584	1.51	14.25	9.66
Cyan671	1.631	135.82	-0.510	1.69	14.90	9.65
Cyan6V2	1.573	133.81	-0.587	1.60	12.55	10.26
Cyan7V2	1.575	134.17	-0.582	1.56	11.00	10.68
Cyan8V2	1.573	133.87	-0.586	1.65	15.60	9.40
Ester0	1.600	135.18	-0.531	1.22	7.68	11.33
Ester1	1.582	134.84	-0.563	1.58	15.14	9.47
Ester2	1.577	134.29	-0.575	1.34	9.82	10.81
Ester30	1.638	138.96	-0.469	1.87	15.43	9.67
Ester3V2	1.570	134.21	-0.586	1.17	7.11	11.45
Ester430	1.708	141.33	-0.412	1.94	12.98	10.47
Ester4V2	1.575	133.85	-0.584	1.60	15.81	9.28
Ester50	1.600	136.24	-0.540	1.25	5.77	11.93
Ester5V2	1.574	133.87	-0.585	1.34	12.07	10.14
Ester671	1.635	136.01	-0.515	1.53	5.29	12.36
Ester6V2	1.574	134.07	-0.584	1.48	15.82	9.16
Ester7V2	1.574	133.98	-0.584	1.37	8.19	11.33
Ester8V2	1.573	133.99	-0.585	1.59	14.89	9.55
Ether0	1.600	135.70	-0.530	1.27	10.27	10.61
Ether1	1.576	134.18	-0.577	1.11	7.87	11.16
Ether2	1.572	134.36	-0.582	1.27	8.71	11.07
Ether30	1.609	136.13	-0.523	1.70	14.11	9.89

Ether3V2	1.575	134.07	-0.584	1.43	11.37	10.44
Ether430	1.627	135.39	-0.513	1.54	10.85	10.71
Ether4V2	1.575	134.03	-0.585	1.34	11.05	10.44
Ether50	1.600	136.25	-0.539	1.18	6.35	11.69
Ether5V2	1.569	134.21	-0.589	1.34	12.12	10.13
Ether671	1.623	135.04	-0.540	2.57	26.63	7.03
Ether6V2	1.572	133.84	-0.587	1.19	6.18	11.74
Ether7V2	1.573	133.97	-0.586	1.52	12.84	10.09
Ether8V2	1.574	133.99	-0.584	1.45	10.52	10.71
F0	1.596	136.12	-0.529	1.91	17.68	9.04
F1	1.579	134.27	-0.576	1.25	11.59	10.19
F2	1.580	134.33	-0.574	1.19	9.94	10.62
F3	1.657	140.19	-0.437	1.76	14.80	9.75
F4	1.618	136.92	-0.508	1.71	14.59	9.76
F5	2.985	179.33	-0.001	2.81	28.32	6.76
F6	1.621	134.71	-0.536	1.78	14.15	9.96
Halid2V2	1.578	134.24	-0.576	1.43	14.09	9.63
Halid3V2	1.574	133.99	-0.583	1.47	13.39	9.87
Halid4V2	1.571	133.90	-0.593	1.48	12.57	10.13
Halid5V2	1.572	133.73	-0.587	1.38	12.80	9.97
Halid6V2	1.573	133.85	-0.586	1.44	12.67	10.07
Halid7V2	1.573	133.80	-0.587	1.37	10.63	10.60
Halid8V2	1.573	133.93	-0.585	1.68	15.55	9.44
I0	1.600	136.30	-0.536	1.10	8.88	10.85
I1	1.575	134.70	-0.566	1.22	11.17	10.29
I2	1.576	134.25	-0.577	1.52	14.39	9.63
I3	1.649	139.28	-0.465	1.71	15.71	9.43
I4	1.627	137.96	-0.495	1.27	8.14	11.24
I5	1.724	142.45	-0.384	1.99	15.47	9.78
I6	1.628	135.84	-0.525	1.77	15.98	9.41
ImidazoleBr1	3.129	177.87	0.000	2.72	24.17	7.91
ImidazoleBr2	3.719	179.78	0.000	2.70	24.03	7.93
ImidazoleC#N1	3.457	178.58	0.000	2.93	30.18	6.33
ImidazoleC#N2	3.442	178.72	0.000	2.83	28.47	6.74
ImidazoleCl1	3.113	178.29	0.000	2.79	27.19	7.08
ImidazoleF1	3.349	179.01	0.000	2.82	30.45	6.14
ImidazoleF2	3.336	178.95	0.000	2.90	32.91	5.49

ImidazoleN1	1.654	139.78	-0.429	1.78	15.37	9.60
ImidazoleN2	1.675	140.97	-0.395	1.88	14.80	9.87
ImidazoleNO2_1	3.378	178.25	0.000	2.90	29.77	6.43
ImidazoleNO2_2	3.471	178.49	0.000	2.89	30.86	6.09
ImidazoleO1	1.663	140.70	-0.386	1.53	13.27	9.98
ImidazoleO2	1.681	141.77	-0.363	1.58	12.59	10.22
ImidazoleS1	3.288	178.85	0.000	2.84	28.65	6.70
ImidazoleS2	3.360	179.02	0.000	2.70	28.19	6.69
Ketone3V2	1.573	133.95	-0.584	1.58	14.61	9.62
Ketone4V2	1.569	133.99	-0.589	1.35	11.73	10.25
Ketone5V2	1.573	133.62	-0.593	1.22	11.87	10.08
Ketone6V2	1.573	133.87	-0.585	1.11	7.61	11.24
Ketone7V2	1.572	133.72	-0.588	1.00	2.63	12.62
Ketone8V2	1.573	133.93	-0.585	1.43	9.05	11.13
Nitro0	1.600	135.60	-0.540	1.04	8.72	10.84
Nitro1	1.582	134.57	-0.567	1.41	12.00	10.23
Nitro3	1.600	134.22	-0.589	1.00	5.69	11.70
Nitro30	1.623	137.86	-0.502	1.57	10.85	10.74
Nitro430	1.662	138.84	-0.447	2.15	15.05	10.06
Nitro4V2	1.569	134.21	-0.588	1.11	7.85	11.17
Nitro50	1.611	136.80	-0.526	1.75	16.80	9.14
Nitro5V2	1.567	134.07	-0.590	1.39	10.54	10.64
Nitro671	1.613	134.07	-0.546	1.97	19.96	8.42
Nitro6V2	1.573	133.99	-0.586	1.57	18.18	8.55
Nitro7V2	1.574	134.03	-0.585	1.42	9.87	10.88
Nitro8V2	1.574	133.92	-0.585	1.68	16.48	9.17
PiperidineBr1	1.600	136.70	-0.547	1.42	9.21	11.08
PiperidineBr2	1.600	134.03	-0.563	1.58	11.21	10.63
PiperidineBr3	1.587	133.70	-0.576	1.68	12.45	10.36
PiperidineC#N1	1.610	136.91	-0.519	1.81	14.15	10.00
PiperidineC#N2	1.600	136.11	-0.564	1.66	12.26	10.40
PiperidineC#N3	1.600	135.83	-0.549	1.49	10.65	10.71
PiperidineCl1	1.600	134.56	-0.546	1.53	10.71	10.74
PiperidineCl2	1.600	135.62	-0.545	1.28	8.04	11.28
PiperidineCl3	1.600	133.59	-0.573	1.49	10.30	10.82
PiperidineF1	1.600	135.92	-0.532	1.33	8.97	11.06
PiperidineF2	1.600	134.11	-0.568	1.58	11.75	10.48

PiperidineF3	1.589	134.78	-0.566	1.77	14.04	9.99
PiperidineI1	1.611	136.68	-0.519	1.82	14.62	9.86
PiperidineI2	1.596	135.40	-0.552	1.73	12.50	10.40
PiperidineI3	1.593	135.27	-0.558	1.81	15.78	9.50
PiperidineN1	1.600	134.84	-0.549	1.47	10.75	10.67
PiperidineN2	1.587	134.62	-0.570	1.78	14.37	9.89
PiperidineN3	1.585	134.54	-0.573	1.84	15.63	9.58
PiperidineNO2_1	1.620	136.85	-0.505	1.74	12.41	10.44
PiperidineNO2_2	1.600	135.52	-0.550	1.58	8.78	11.36
PiperidineNO2_3	1.600	136.92	-0.555	1.50	10.08	10.89
PiperidineO1	1.600	134.90	-0.538	1.33	10.07	10.73
PiperidineO2	1.593	135.03	-0.559	1.58	14.73	9.59
PiperidineO3	1.582	135.06	-0.564	1.66	12.49	10.34
PiperidineS1	1.600	135.77	-0.558	1.46	10.65	10.69
PiperidineS2	1.584	133.35	-0.584	1.86	14.11	10.05
PiperidineS3	1.600	133.40	-0.577	1.43	9.23	11.08
PyrazineBr1	1.643	138.09	-0.407	1.91	15.15	9.79
PyrazineBr2	1.619	138.89	-0.431	1.92	15.56	9.68
PyrazineC#N1	1.640	140.11	-0.395	1.85	15.58	9.60
PyrazineC#N2	1.622	139.65	-0.419	1.93	15.32	9.76
PyrazineCl1	1.634	137.97	-0.415	1.86	15.33	9.69
PyrazineCl2	1.620	138.84	-0.432	1.91	15.45	9.70
PyrazineF1	1.623	139.99	-0.409	1.92	15.64	9.66
PyrazineF2	1.616	138.91	-0.433	1.90	15.84	9.58
PyrazineN1	1.543	132.53	-0.538	1.70	13.95	9.94
PyrazineN2	1.590	135.97	-0.498	1.97	16.23	9.53
PyrazineNO2_1	1.672	142.66	-0.353	1.82	12.02	10.64
PyrazineNO2_2	1.645	141.09	-0.386	1.94	13.54	10.31
PyrazineO1	1.587	136.53	-0.460	1.83	15.56	9.59
PyrazineO2	1.609	137.90	-0.453	1.88	16.41	9.39
PyrazineS1	1.571	136.30	-0.470	1.49	10.36	10.80
PyrazineS2	1.605	137.77	-0.457	1.85	15.70	9.57
PyrazoleBr1	3.232	179.80	0.000	2.86	29.29	6.53
PyrazoleBr2	3.357	178.94	0.000	2.76	24.20	7.94
PyrazoleC#N1	3.375	178.62	0.000	2.97	33.73	5.32
PyrazoleC#N2	3.415	178.80	0.000	2.78	27.07	7.10
PyrazoleCl1	3.323	179.14	0.000	2.96	34.76	5.00

PyrazoleCl2	3.364	179.68	0.000	2.81	27.47	7.02
PyrazoleF1	3.330	179.56	0.000	2.82	32.79	5.45
PyrazoleF2	3.351	179.63	0.000	2.79	28.59	6.67
PyrazoleI1	3.578	179.84	0.000	2.87	29.04	6.61
PyrazoleI2	3.253	178.96	0.000	3.00	31.45	6.02
PyrazoleN1	1.645	138.18	-0.476	2.02	15.78	9.71
PyrazoleN2	1.656	139.17	-0.450	1.79	13.63	10.12
PyrazoleNO2_1	3.365	178.69	0.000	2.84	31.14	5.96
PyrazoleNO2_2	3.471	178.56	0.000	2.65	25.62	7.41
PyrazoleO1	1.686	141.66	-0.397	1.77	14.70	9.79
PyrazoleO2	1.719	143.51	-0.362	1.86	14.37	9.98
PyrazoleS1	3.576	179.68	0.000	2.76	30.00	6.22
PyrazoleS2	1.678	141.46	-0.397	1.91	16.45	9.41
PyridineBr1	1.600	134.57	-0.474	1.83	13.50	10.20
PyridineBr2	1.579	135.85	-0.497	1.88	15.12	9.77
PyridineBr3	1.576	135.60	-0.503	1.87	14.94	9.81
PyridineC#N1	1.593	136.42	-0.468	1.84	14.50	9.92
PyridineC#N2	1.583	136.59	-0.484	1.89	17.37	9.12
PyridineC#N3	1.583	136.21	-0.491	1.89	16.36	9.41
PyridineCl1	1.583	134.23	-0.490	1.75	12.95	10.29
PyridineCl2	1.579	135.85	-0.498	1.86	15.43	9.66
PyridineCl3	1.572	135.34	-0.509	1.85	14.91	9.81
PyridineF1	1.579	136.59	-0.479	1.85	14.90	9.81
PyridineF2	1.581	135.96	-0.495	1.87	16.46	9.37
PyridineF3	1.570	135.49	-0.506	1.84	15.32	9.67
PyridineI1	1.585	134.62	-0.490	1.80	13.21	10.26
PyridineI2	1.574	135.58	-0.505	1.84	14.00	10.07
PyridineI3	1.574	135.54	-0.508	1.85	14.30	9.98
PyridineN1	1.528	130.84	-0.575	1.82	14.95	9.76
PyridineN2	1.567	134.21	-0.538	1.86	15.41	9.67
PyridineN3	1.521	131.68	-0.600	1.84	13.77	10.14
PyridineNO2_1	1.618	139.07	-0.426	2.03	16.77	9.43
PyridineNO2_2	1.600	137.68	-0.454	1.90	14.80	9.89
PyridineNO2_3	1.591	137.35	-0.473	1.82	13.07	10.32
PyridineO1	1.543	132.43	-0.500	1.58	12.24	10.33
PyridineO2	1.575	135.20	-0.514	1.83	14.56	9.89
PyridineO3	1.550	133.85	-0.543	1.79	14.70	9.81

PyridineS1	1.568	133.65	-0.495	1.80	15.48	9.58
PyridineS3	1.562	134.53	-0.528	1.78	14.68	9.81
PyrimidineBr1	1.616	136.21	-0.433	1.88	15.47	9.66
PyrimidineBr2	1.607	137.50	-0.433	1.97	17.02	9.29
PyrimidineBr3	1.586	137.82	-0.460	1.86	15.97	9.50
PyrimidineC#N1	1.625	138.92	-0.408	1.94	16.76	9.34
PyrimidineC#N2	1.618	139.06	-0.411	2.04	17.47	9.23
PyrimidineC#N3	1.607	138.76	-0.430	1.91	16.65	9.35
PyrimidineCl1	1.612	138.59	-0.423	1.92	16.20	9.50
PyrimidineCl2	1.606	138.20	-0.430	1.91	14.78	9.90
PyrimidineCl3	1.596	137.77	-0.452	1.93	18.48	8.82
PyrimidineF1	1.600	138.93	-0.427	1.91	17.21	9.18
PyrimidineF2	1.598	138.72	-0.432	1.89	14.78	9.88
PyrimidineF3	1.599	137.97	-0.448	1.90	17.09	9.21
PyrimidineI1	1.627	139.34	-0.407	1.88	15.81	9.56
PyrimidineI3	1.595	137.79	-0.453	1.88	15.81	9.56
PyrimidineN1	1.541	132.73	-0.534	1.74	16.98	9.08
PyrimidineN2	1.537	132.36	-0.540	1.51	12.37	10.22
PyrimidineN3	1.580	135.84	-0.497	1.84	14.14	10.03
PyrimidineNO2_1	1.648	141.60	-0.370	2.06	17.91	9.12
PyrimidineNO2_2	1.645	141.68	-0.372	2.09	15.82	9.77
PyrimidineNO2_3	1.623	140.05	-0.403	2.00	17.38	9.22
PyrimidineO1	1.574	136.11	-0.470	1.85	14.32	9.98
PyrimidineO2	1.549	133.87	-0.466	1.63	12.45	10.32
PyrimidineO3	1.587	137.21	-0.471	1.75	14.46	9.84
PyrimidineS1	1.556	135.16	-0.487	1.66	12.49	10.34
PyrimidineS2	1.578	135.24	-0.460	1.86	14.71	9.87
PyrimidineS3	1.590	137.27	-0.462	1.89	14.98	9.82
PyrroleBr1	1.659	139.90	-0.429	1.96	17.80	9.05
PyrroleBr2	1.659	139.89	-0.430	1.95	18.39	8.86
PyrroleC#N1	1.683	141.76	-0.394	1.97	16.65	9.40
PyrroleC#N2	1.699	142.48	-0.378	2.03	16.60	9.48
PyrroleCl1	1.659	139.99	-0.428	1.84	14.41	9.94
PyrroleCl2	1.654	139.56	-0.438	1.88	15.60	9.63
PyrroleF1	1.673	141.02	-0.407	1.91	15.97	9.55
PyrroleF2	1.657	139.74	-0.435	1.80	14.12	9.99
PyrroleI2	1.656	139.66	-0.435	1.96	18.74	8.77

PyrroleN1	1.637	138.43	-0.465	1.60	12.23	10.35
PyrroleN2	1.605	135.80	-0.528	1.81	15.00	9.74
PyrroleNO2_1	3.384	178.45	0.000	2.98	31.85	5.88
PyrroleNO2_2	3.676	179.68	0.000	2.91	31.16	6.02
PyrroleO1	1.626	137.54	-0.453	1.30	10.41	10.60
PyrroleO2	1.627	137.63	-0.483	1.77	14.99	9.70
PyrroleS1	1.665	140.52	-0.423	1.90	15.13	9.79
PyrroleS2	1.636	138.18	-0.468	1.71	14.86	9.68
ThiazoleBr1	1.566	134.42	-0.501	1.82	14.41	9.92
ThiazoleBr2	1.558	135.10	-0.508	1.87	15.13	9.76
ThiazoleC#N1	1.563	135.37	-0.496	1.84	15.60	9.59
ThiazoleC#N2	1.564	135.70	-0.493	1.84	14.68	9.87
ThiazoleC#N3	1.570	135.85	-0.483	1.84	14.55	9.90
ThiazoleCl1	1.557	134.30	-0.511	1.80	15.20	9.67
ThiazoleCl2	1.555	135.34	-0.509	1.86	15.83	9.54
ThiazoleCl3	1.559	134.18	-0.507	1.78	14.23	9.93
ThiazoleF1	1.558	135.59	-0.494	1.91	15.46	9.70
ThiazoleF2	1.563	135.37	-0.501	1.85	15.46	9.64
ThiazoleF3	1.557	135.71	-0.495	1.89	15.39	9.70
ThiazoleI1	1.568	134.23	-0.501	1.86	17.08	9.17
ThiazoleI2	1.555	134.86	-0.513	1.87	15.02	9.79
ThiazoleN1	1.510	131.54	-0.583	1.54	12.26	10.28
ThiazoleN2	1.556	134.07	-0.537	1.78	15.14	9.66
ThiazoleN3	1.532	132.26	-0.551	1.74	13.99	9.97
ThiazoleNO2_1	1.567	136.19	-0.485	1.96	15.79	9.65
ThiazoleNO2_2	1.573	136.61	-0.472	1.94	15.78	9.63
ThiazoleNO2_3	1.582	137.15	-0.459	1.82	14.93	9.77
ThiazoleO1	1.539	133.89	-0.524	1.76	14.54	9.83
ThiazoleO2	1.560	135.30	-0.512	1.85	15.74	9.56
ThiazoleO3	1.542	133.98	-0.522	1.94	15.73	9.65
ThiazoleS1	1.524	133.13	-0.548	1.66	14.60	9.70
ThiazoleS2	1.552	134.48	-0.525	2.09	16.43	9.59
ThiazoleS3	1.537	133.73	-0.528	1.66	15.15	9.54
Tio0	1.575	134.26	-0.576	1.25	11.47	10.23
Tio1	1.570	134.21	-0.585	1.31	11.08	10.41
Tio2	1.573	133.94	-0.585	1.63	15.53	9.41
Tio30	1.600	135.55	-0.550	1.23	8.61	11.06

Tio3V2	1.573	133.84	-0.587	1.42	11.18	10.49
Tio430	1.630	134.09	-0.552	1.68	9.02	11.39
Tio4V2	1.573	133.66	-0.588	1.54	15.81	9.23
Tio50	1.600	135.92	-0.570	1.44	10.71	10.64
Tio5V2	1.574	133.83	-0.586	1.44	10.66	10.66
Tio671	1.615	134.11	-0.559	1.15	1.76	13.03
Tio6V2	1.573	133.93	-0.585	1.75	17.82	8.83
Tio7V2	1.574	134.03	-0.585	1.53	13.46	9.92
Tio8V2	1.573	133.97	-0.585	1.81	16.19	9.38
TriazoleBr2	3.332	178.10	0.000	1.84	16.36	9.37
TriazoleC#N1	3.444	178.45	0.001	2.93	33.09	5.47
TriazoleC#N2	3.508	178.50	0.001	1.90	15.47	9.69
TriazoleCl1	3.473	178.56	0.000	3.00	35.71	4.75
TriazoleCl2	3.397	178.26	0.000	2.12	23.20	7.60
TriazoleF1	3.400	178.42	0.000	2.86	29.29	6.52
TriazoleF2	3.660	179.42	0.000	2.04	20.70	8.27
TriazoleI2	3.739	179.62	0.002	1.95	19.52	8.53
TriazoleN1	3.379	178.77	0.000	2.92	30.63	6.19
TriazoleN2	3.353	178.25	0.000	2.19	21.55	8.17
TriazoleNO2_1	3.541	178.25	0.000	3.05	33.45	5.48
TriazoleNO2_2	3.466	177.94	0.000	2.28	24.99	7.23
TriazoleO1	3.348	178.70	0.000	2.95	31.67	5.91
TriazoleO2	1.703	143.77	-0.293	1.62	13.37	10.04
TriazoleS1	3.960	179.80	0.003	2.98	31.05	6.12
TriazoleS2	3.336	177.89	0.001	2.04	20.09	8.45

Table S14: Parameters calculated using the PM6-DH+ method.

Name	R (Å)	θ (°)	ΔQ	ΔH (kcal/mol)	ΔS (cal/molK)	ΔG (kcal/mol)
Acid0	1.597	136.35	-0.523	1.97	17.44	9.18
Acid1	1.571	135.49	-0.562	1.03	6.50	11.49
Acid2	1.577	134.23	-0.575	1.49	15.52	9.26
Acid30	1.645	139.11	-0.444	2.04	16.21	9.61
Acid3V2	1.575	134.27	-0.576	1.52	17.75	8.63
Acid430	1.740	144.46	-0.358	2.01	10.79	11.19
Acid4V2	1.575	134.21	-0.579	1.48	11.99	10.31
Acid50	1.611	137.09	-0.468	1.65	13.77	9.95

Acid671	1.635	136.36	-0.507	2.07	11.78	10.96
Acid6V2	1.577	134.60	-0.571	1.38	14.32	9.51
Acid7V2	1.574	134.09	-0.581	1.50	20.25	7.87
Acid8V2	1.574	134.17	-0.580	1.67	20.90	7.84
AcidBr1	1.587	135.31	-0.549	1.21	7.79	11.29
AcidBr2	1.577	134.49	-0.569	1.66	15.46	9.45
AcidBr3	1.664	141.16	-0.428	1.59	8.34	11.51
AcidBr4	1.650	139.88	-0.454	1.62	13.42	10.02
AcidBr5	2.968	179.17	-0.001	2.92	28.38	6.86
AcidBr6	1.661	138.49	-0.465	1.58	5.96	12.21
AcidCl0	1.601	136.88	-0.511	1.37	11.58	10.31
AcidCl1	1.583	135.40	-0.552	1.58	15.11	9.47
AcidCl2	1.573	134.77	-0.570	1.42	12.55	10.07
AcidCl3	1.661	141.25	-0.431	1.55	12.20	10.31
AcidCl4	1.634	138.96	-0.472	1.53	14.91	9.49
AcidCl5	3.168	179.74	0.000	2.87	25.88	7.56
AcidCl6	1.653	136.89	-0.483	1.59	11.39	10.60
AcidF0	1.603	136.98	-0.508	1.65	17.44	8.85
AcidF1	1.585	135.19	-0.552	1.59	17.36	8.82
AcidF2	1.578	134.49	-0.569	1.57	13.28	10.01
AcidF3	1.676	141.54	-0.415	1.86	8.40	11.75
AcidF4	1.634	138.61	-0.474	1.65	15.78	9.35
AcidF5	2.934	179.11	-0.001	2.53	19.65	9.08
AcidF6	1.643	137.07	-0.488	1.66	8.06	11.66
AcidI0	1.600	136.82	-0.514	1.17	8.67	10.99
AcidI1	1.600	135.12	-0.544	1.49	12.78	10.08
AcidI2	1.578	134.46	-0.570	1.46	10.68	10.68
AcidI3	1.661	140.79	-0.434	1.95	23.29	7.40
AcidI4	1.645	139.58	-0.461	1.60	10.13	10.98
AcidI5	1.936	156.39	-0.176	2.02	10.77	11.21
AcidI6	1.663	137.96	-0.474	0.75	-8.50	15.68
Acid_Halid3V2	1.576	134.28	-0.575	1.52	12.87	10.09
Acid_Halid4V2	1.575	134.17	-0.578	1.59	16.93	8.94
Acid_Halid5V2	1.574	134.10	-0.580	1.32	11.44	10.31
Acid_Halid6V2	1.567	134.37	-0.584	1.38	8.20	11.34
Acid_Halid7V2	1.572	133.88	-0.582	1.55	12.82	10.13
Acid_Halid8V2	1.575	134.26	-0.578	1.23	9.43	10.82

Acid_Sulf0	1.584	135.10	-0.557	1.22	7.31	11.44
Acid_Sulf1	1.579	134.25	-0.574	1.56	12.63	10.20
Acid_Sulf2	1.576	134.28	-0.576	1.56	16.54	9.03
Acid_Sulf30	1.610	136.45	-0.518	2.11	18.04	9.13
Acid_Sulf3V2	1.576	134.31	-0.575	1.51	12.96	10.05
Acid_Sulf430	1.642	136.87	-0.487	1.88	11.63	10.81
Acid_Sulf4V2	1.573	133.97	-0.581	1.75	12.78	10.34
Acid_Sulf50	1.600	136.93	-0.542	1.70	13.42	10.10
Acid_Sulf5V2	1.557	134.60	-0.595	1.07	5.79	11.74
Acid_Sulf671	1.624	135.30	-0.533	1.52	8.33	11.43
Acid_Sulf6V2	1.575	134.21	-0.578	1.70	13.92	9.95
Acid_Sulf7V2	1.574	134.12	-0.581	1.44	14.04	9.66
Acid_Sulf8V2	1.574	134.18	-0.579	1.43	13.24	9.89
Acid_Tio0	1.600	135.62	-0.527	1.74	11.71	10.65
Acid_Tio1	1.579	134.77	-0.564	1.30	10.16	10.67
Acid_Tio2	1.600	134.49	-0.561	1.23	14.06	9.44
Acid_Tio30	1.632	139.67	-0.475	1.86	16.11	9.46
Acid_Tio3V2	1.574	134.15	-0.579	1.59	15.02	9.51
Acid_Tio430	1.676	139.68	-0.429	2.13	9.43	11.72
Acid_Tio4V2	1.574	134.04	-0.580	1.85	17.23	9.12
Acid_Tio50	1.627	137.98	-0.492	1.55	8.65	11.38
Acid_Tio5V2	1.574	134.09	-0.581	1.35	11.20	10.41
Acid_Tio671	1.610	132.58	-0.546	0.65	-8.75	15.66
Acid_Tio6V2	1.568	134.07	-0.584	1.32	8.74	11.12
Acid_Tio7V2	1.574	134.05	-0.582	1.37	11.78	10.26
Acid_Tio8V2	1.575	134.27	-0.578	1.71	14.00	9.94
Alcohol0	1.576	134.49	-0.570	1.38	13.79	9.67
Alcohol1	1.572	134.80	-0.575	1.25	10.32	10.57
Alcohol2	1.575	134.29	-0.577	1.63	17.79	8.73
Alcohol30	1.591	134.72	-0.559	0.97	4.53	12.02
Alcohol3V2	1.574	134.18	-0.578	1.57	15.12	9.46
Alcohol430	1.625	134.84	-0.530	2.01	17.76	9.12
Alcohol4V2	1.568	134.42	-0.583	1.24	10.07	10.63
Alcohol50	1.600	136.43	-0.562	1.52	11.06	10.62
Alcohol5V2	1.575	134.09	-0.580	1.39	9.20	11.05
Alcohol671	1.598	131.87	-0.564	1.30	4.10	12.48
Alcohol6V2	1.574	134.18	-0.579	1.51	15.71	9.22

Alcohol7V2	1.574	134.18	-0.580	1.46	11.17	10.53
Alcohol8V2	1.574	134.05	-0.580	1.77	14.28	9.92
Aldehyde0	1.585	135.25	-0.551	1.48	14.78	9.48
Aldehyde1	1.573	134.36	-0.573	1.34	13.91	9.59
Aldehyde2	1.575	134.47	-0.571	1.52	14.16	9.70
Aldehyde30	1.614	136.77	-0.519	1.49	10.97	10.62
Aldehyde430	1.645	137.34	-0.496	1.57	10.35	10.89
Aldehyde50	1.600	136.26	-0.533	0.89	1.01	12.99
Aldehyde671	1.621	135.42	-0.536	1.84	13.16	10.32
Amide0	1.589	135.08	-0.538	1.33	13.12	9.82
Amide1	1.580	134.82	-0.566	1.55	14.44	9.65
Amide2	1.577	134.63	-0.571	1.55	17.97	8.59
Amide30	1.633	137.63	-0.479	1.41	7.84	11.47
Amide3V2	1.574	134.13	-0.579	1.57	18.47	8.46
Amide430	1.688	140.68	-0.425	1.58	7.78	11.66
Amide4V2	1.574	134.05	-0.579	1.61	14.27	9.76
Amide50	1.618	136.88	-0.508	1.53	12.04	10.34
Amide5V2	1.573	133.89	-0.586	0.99	6.96	11.32
Amide671	1.641	136.10	-0.515	1.78	10.16	11.15
Amide6V2	1.567	135.03	-0.583	1.03	3.36	12.43
Amide7V2	1.575	134.20	-0.579	1.48	10.41	10.77
Amide8V2	1.574	134.18	-0.579	1.54	11.76	10.43
Amine0	1.577	134.12	-0.576	1.64	18.21	8.62
Amine1	1.574	134.21	-0.583	1.44	17.30	8.69
Amine2	1.573	134.18	-0.583	1.52	14.81	9.50
Amine30	1.574	134.37	-0.576	1.48	14.48	9.56
Amine3V2	1.567	134.48	-0.585	1.11	1.83	12.97
Amine430	1.608	133.89	-0.558	1.72	9.67	11.24
Amine4V2	1.567	134.39	-0.585	1.34	10.31	10.67
Amine50	1.587	134.30	-0.574	1.44	9.21	11.10
Amine5V2	1.574	134.05	-0.580	1.31	8.75	11.10
Amine671	1.600	132.29	-0.576	1.58	8.06	11.57
Amine6V2	1.566	134.49	-0.585	1.47	14.67	9.49
Amine7V2	1.574	134.19	-0.579	1.43	8.61	11.26
Amine8V2	1.574	134.18	-0.580	1.54	14.76	9.54
Anhydride0	1.600	136.26	-0.520	1.34	10.22	10.69
Anhydride1	1.583	134.96	-0.555	1.44	10.17	10.80

Anhydride2	1.600	134.41	-0.557	1.52	8.06	11.52
Anhydride30	1.663	141.02	-0.425	1.57	8.14	11.54
Anhydride3V2	1.576	134.46	-0.573	1.14	6.10	11.72
Anhydride430	3.089	179.67	-0.001	2.26	11.51	11.23
Anhydride4V2	1.574	134.19	-0.578	1.50	12.43	10.20
Anhydride50	1.620	136.39	-0.510	1.80	15.39	9.62
Anhydride5V2	1.573	134.13	-0.580	1.29	8.80	11.06
Anhydride671	1.638	136.34	-0.496	1.80	6.73	12.20
Anhydride7V2	1.574	134.12	-0.581	1.29	9.92	10.74
Anhydride8V2	1.575	134.19	-0.579	1.45	10.66	10.68
AzolidineBr1	1.600	136.30	-0.525	1.06	5.25	11.90
AzolidineBr2	1.591	134.80	-0.560	1.64	13.22	10.10
AzolidineC#N1	1.600	138.59	-0.516	1.53	11.00	10.65
AzolidineC#N2	1.600	137.08	-0.554	1.52	12.22	10.28
AzolidineCl1	1.604	136.70	-0.518	1.21	10.25	10.55
AzolidineCl2	1.600	134.51	-0.550	1.44	10.83	10.61
AzolidineF1	1.600	136.27	-0.525	1.43	9.89	10.88
AzolidineF2	1.590	134.67	-0.563	1.74	17.03	9.07
AzolidineI1	1.600	136.59	-0.531	1.02	2.33	12.73
AzolidineI2	1.589	134.71	-0.563	1.56	11.72	10.46
AzolidineN1	1.576	134.09	-0.578	1.56	12.80	10.14
AzolidineN2	1.585	134.15	-0.578	1.72	15.86	9.39
AzolidineNO2_1	1.600	137.27	-0.517	1.32	8.37	11.23
AzolidineNO2_2	1.600	135.95	-0.540	1.32	9.64	10.85
AzolidineO1	1.582	134.96	-0.559	1.53	9.75	11.03
AzolidineO2	1.600	134.59	-0.551	1.48	12.32	10.21
AzolidineS1	1.600	135.12	-0.549	1.39	8.98	11.11
AzolidineS2	1.586	134.83	-0.560	1.48	10.96	10.61
Br0	1.602	136.30	-0.538	1.54	13.80	9.82
Br1	1.583	134.91	-0.561	1.33	12.88	9.89
Br2	1.574	134.77	-0.571	1.48	14.29	9.62
Br3	1.664	139.84	-0.455	1.53	11.41	10.53
Br4	1.634	137.97	-0.492	1.64	13.77	9.93
Br5	3.332	178.55	0.000	2.85	31.70	5.80
Br6	1.626	135.56	-0.515	1.90	17.35	9.13
Cl0	1.604	136.85	-0.514	1.23	10.71	10.44
Cl1	1.579	134.57	-0.569	1.44	16.64	8.88

Cl2	1.577	134.49	-0.571	1.30	10.02	10.71
Cl3	1.652	139.75	-0.450	1.71	15.39	9.52
Cl4	1.631	138.52	-0.482	1.50	10.12	10.88
Cl5	3.230	179.27	-0.007	2.87	30.87	6.07
Cl6	1.643	137.25	-0.495	2.29	23.15	7.79
Cyan0	1.602	136.85	-0.515	1.64	15.02	9.57
Cyan1	1.581	134.47	-0.563	1.38	14.69	9.40
Cyan2	1.576	134.07	-0.576	1.47	13.99	9.70
Cyan30	1.643	139.63	-0.462	1.74	16.01	9.36
Cyan3V2	1.576	134.33	-0.575	1.46	13.58	9.81
Cyan430	1.724	143.26	-0.365	1.93	12.46	10.61
Cyan4V2	1.568	134.36	-0.582	1.34	12.33	10.06
Cyan50	1.619	137.77	-0.503	1.54	6.55	11.99
Cyan5V2	1.568	134.57	-0.583	1.17	9.43	10.76
Cyan671	1.634	136.00	-0.502	1.93	19.51	8.51
Cyan6V2	1.575	134.22	-0.579	1.45	13.86	9.72
Cyan7V2	1.574	134.16	-0.580	1.43	11.54	10.39
Cyan8V2	1.574	134.15	-0.580	1.59	14.29	9.73
Ester0	1.600	135.36	-0.525	1.53	11.51	10.50
Ester1	1.582	134.95	-0.558	1.48	14.07	9.69
Ester2	1.577	134.28	-0.572	1.46	14.18	9.63
Ester30	1.637	138.77	-0.470	1.80	15.53	9.57
Ester3V2	1.576	134.32	-0.576	1.33	8.76	11.12
Ester430	1.706	141.37	-0.413	1.81	11.68	10.73
Ester4V2	1.575	134.23	-0.577	1.52	13.01	10.04
Ester50	1.600	136.44	-0.537	1.25	6.83	11.61
Ester5V2	1.571	134.10	-0.582	1.30	10.02	10.71
Ester671	1.634	136.15	-0.511	1.69	3.88	12.93
Ester6V2	1.575	134.15	-0.578	1.70	14.90	9.66
Ester7V2	1.573	134.07	-0.581	1.45	12.36	10.16
Ester8V2	1.574	134.12	-0.580	1.79	20.90	7.96
Ether0	1.600	135.87	-0.525	1.30	9.61	10.84
Ether1	1.578	134.56	-0.568	1.27	9.84	10.74
Ether2	1.570	134.61	-0.579	1.36	12.56	10.02
Ether30	1.611	136.37	-0.519	1.54	8.70	11.34
Ether3V2	1.575	134.24	-0.578	1.43	12.11	10.22
Ether430	1.629	135.28	-0.515	1.78	12.65	10.41

Ether4V2	1.568	134.15	-0.584	1.82	21.85	7.71
Ether50	1.600	135.39	-0.540	1.19	4.58	12.22
Ether5V2	1.568	134.47	-0.584	1.27	7.32	11.49
Ether671	1.620	135.11	-0.540	2.06	16.35	9.59
Ether6V2	1.574	134.14	-0.580	1.83	20.54	8.10
Ether7V2	1.575	134.20	-0.578	1.59	13.68	9.91
Ether8V2	1.576	134.34	-0.576	1.32	8.91	11.06
F0	1.598	136.44	-0.522	1.54	15.47	9.33
F1	1.584	134.65	-0.568	1.37	12.51	10.04
F2	1.580	134.59	-0.568	1.21	9.97	10.64
F3	1.661	140.38	-0.434	1.84	16.87	9.21
F4	1.615	136.24	-0.511	1.63	12.62	10.27
F5	3.054	179.44	-0.001	2.78	28.57	6.66
F6	1.620	134.84	-0.534	1.44	4.35	12.55
Halid2V2	1.578	134.57	-0.570	1.55	17.39	8.77
Halid3V2	1.575	134.29	-0.577	1.34	12.66	9.96
Halid4V2	1.572	133.94	-0.586	1.39	11.52	10.36
Halid5V2	1.574	134.18	-0.580	1.24	10.62	10.48
Halid6V2	1.571	133.96	-0.583	1.46	12.59	10.11
Halid7V2	1.567	134.37	-0.585	1.41	14.27	9.56
Halid8V2	1.574	134.16	-0.580	1.55	14.65	9.59
I0	1.600	136.75	-0.534	1.30	11.52	10.27
I1	1.575	134.99	-0.562	1.26	11.19	10.33
I2	1.573	134.65	-0.573	1.22	9.21	10.87
I3	1.648	139.10	-0.468	1.76	15.62	9.50
I4	1.624	137.63	-0.503	1.45	9.91	10.90
I5	1.696	141.41	-0.415	1.68	9.26	11.32
I6	1.627	135.93	-0.522	1.72	8.97	11.44
ImidazoleBr1	3.314	178.62	0.000	2.84	31.02	5.99
ImidazoleC#N1	3.477	178.83	0.000	2.73	30.39	6.07
ImidazoleC#N2	3.382	178.78	0.000	2.63	25.83	7.33
ImidazoleCl1	3.302	178.88	0.000	2.74	29.29	6.41
ImidazoleF1	3.330	179.11	0.000	2.83	30.30	6.20
ImidazoleF2	3.370	178.81	0.000	2.78	30.62	6.05
ImidazoleN1	1.654	139.72	-0.428	1.62	12.22	10.38
ImidazoleN2	1.678	141.35	-0.390	1.78	14.45	9.87
ImidazoleNO2_1	3.098	176.37	0.001	2.81	27.44	7.03

ImidazoleNO2_2	3.489	178.60	0.000	2.82	29.16	6.53
ImidazoleO1	1.651	140.26	-0.393	1.52	11.81	10.40
ImidazoleO2	1.678	142.08	-0.366	1.56	12.68	10.17
ImidazoleS1	3.307	178.82	0.000	2.96	31.91	5.85
ImidazoleS2	3.456	178.87	0.000	2.57	24.09	7.79
Ketone3V2	1.570	134.40	-0.579	1.41	10.17	10.78
Ketone4V2	1.573	134.16	-0.580	1.64	15.63	9.38
Ketone5V2	1.574	133.75	-0.586	1.20	9.08	10.89
Ketone6V2	1.574	134.05	-0.580	1.25	8.37	11.16
Ketone7V2	1.570	134.04	-0.583	1.50	16.30	9.04
Ketone8V2	1.574	134.16	-0.580	1.61	13.97	9.84
Nitro0	1.600	135.87	-0.534	1.24	11.75	10.13
Nitro1	1.600	135.39	-0.548	1.15	10.88	10.31
Nitro3	1.574	133.94	-0.583	1.39	11.09	10.48
Nitro30	1.624	138.10	-0.497	1.74	12.59	10.39
Nitro430	1.673	139.26	-0.435	1.52	3.62	12.84
Nitro4V2	1.568	134.36	-0.583	0.93	3.65	12.24
Nitro50	1.611	136.87	-0.524	1.38	6.45	11.86
Nitro5V2	1.573	134.66	-0.579	1.36	9.23	11.01
Nitro671	1.610	133.82	-0.548	1.72	13.53	10.08
Nitro6V2	1.574	134.16	-0.579	1.59	12.38	10.29
Nitro7V2	1.574	134.07	-0.580	1.25	7.02	11.56
Nitro8V2	1.574	134.17	-0.580	1.60	15.47	9.39
PiperidineBr1	1.614	135.81	-0.524	1.73	12.71	10.34
PiperidineBr2	1.600	134.51	-0.561	1.50	10.06	10.90
PiperidineBr3	1.600	133.54	-0.569	1.56	10.63	10.79
PiperidineC#N1	1.611	137.04	-0.518	1.74	13.59	10.09
PiperidineC#N2	1.600	136.30	-0.563	1.71	12.93	10.25
PiperidineC#N3	1.600	135.91	-0.548	1.49	10.53	10.75
PiperidineCl1	1.600	136.78	-0.544	1.44	9.92	10.88
PiperidineCl2	1.600	135.35	-0.546	1.48	10.59	10.72
PiperidineCl3	1.600	133.40	-0.571	1.54	10.78	10.72
PiperidineF1	1.600	136.00	-0.531	1.52	10.77	10.71
PiperidineF2	1.590	134.18	-0.569	1.69	13.57	10.05
PiperidineF3	1.590	134.79	-0.565	1.81	16.59	9.26
PiperidineI1	1.612	136.76	-0.518	1.78	13.23	10.24
PiperidineI2	1.600	136.06	-0.546	1.41	8.93	11.15

PiperidineI3	1.587	134.91	-0.563	1.80	15.84	9.48
PiperidineN1	1.579	133.66	-0.586	1.56	11.11	10.64
PiperidineN2	1.586	134.57	-0.572	1.57	12.42	10.26
PiperidineN3	1.586	134.67	-0.572	1.59	10.89	10.74
PiperidineNO2_1	1.628	137.59	-0.494	1.87	16.39	9.38
PiperidineNO2_2	1.600	135.62	-0.549	1.64	12.82	10.22
PiperidineNO2_3	1.600	135.14	-0.570	1.55	11.32	10.57
PiperidineO1	1.600	135.05	-0.533	1.32	9.05	11.02
PiperidineO2	1.591	135.07	-0.560	1.70	14.48	9.79
PiperidineO3	1.588	135.08	-0.563	1.76	16.34	9.29
PiperidineS1	1.600	135.94	-0.556	1.56	13.74	9.87
PiperidineS2	1.600	133.42	-0.576	1.52	11.11	10.61
PiperidineS3	1.586	133.36	-0.581	1.79	15.43	9.59
PyrazineBr1	1.664	138.81	-0.384	1.95	15.96	9.59
PyrazineBr2	1.617	138.81	-0.433	1.92	15.47	9.71
PyrazineC#N1	1.641	140.07	-0.394	1.95	16.60	9.40
PyrazineC#N2	1.626	139.80	-0.416	1.89	14.82	9.87
PyrazineCl1	1.627	138.17	-0.421	1.89	16.13	9.48
PyrazineCl2	1.607	138.64	-0.444	1.92	16.72	9.34
PyrazineF1	1.623	139.95	-0.410	1.91	15.76	9.62
PyrazineF2	1.615	138.87	-0.434	1.91	16.05	9.52
PyrazineI1	1.631	139.18	-0.415	2.00	16.21	9.57
PyrazineI2	1.609	138.44	-0.443	1.93	17.30	9.17
PyrazineN1	1.542	132.68	-0.539	1.86	14.79	9.85
PyrazineN2	1.591	136.20	-0.493	1.90	16.81	9.28
PyrazineNO2_1	1.693	143.57	-0.333	1.85	10.48	11.12
PyrazineNO2_2	1.641	140.93	-0.390	1.96	15.00	9.89
PyrazineO1	1.586	136.52	-0.461	1.84	15.37	9.66
PyrazineO2	1.609	137.86	-0.454	1.84	15.16	9.72
PyrazineS1	1.576	136.29	-0.469	1.61	12.43	10.30
PyrazineS2	1.601	137.68	-0.461	1.90	15.46	9.69
PyrazoleBr1	3.477	179.86	0.000	2.85	27.46	7.07
PyrazoleBr2	1.836	150.69	-0.233	2.15	19.23	8.81
PyrazoleC#N1	3.464	178.82	0.000	2.82	33.05	5.37
PyrazoleC#N2	3.410	178.87	0.000	2.76	30.87	5.96
PyrazoleCl1	3.171	179.50	0.000	2.85	29.67	6.41
PyrazoleCl2	2.147	168.17	-0.054	2.45	21.77	8.36

PyrazoleF1	3.293	179.67	0.000	2.78	29.28	6.45
PyrazoleF2	3.325	179.76	0.000	2.93	36.23	4.53
PyrazoleI1	3.353	178.90	0.000	2.76	31.88	5.66
PyrazoleI2	3.270	177.86	0.001	2.74	26.99	7.10
PyrazoleN1	1.637	137.79	-0.482	2.15	18.44	9.05
PyrazoleN2	1.641	138.45	-0.459	1.71	13.00	10.24
PyrazoleNO2_1	3.869	179.17	0.000	2.84	30.43	6.17
PyrazoleNO2_2	3.544	178.87	0.000	2.54	25.41	7.36
PyrazoleO1	1.695	142.02	-0.390	1.82	16.51	9.30
PyrazoleO2	1.717	143.27	-0.364	1.94	15.93	9.59
PyrazoleS1	1.719	143.85	-0.355	2.00	16.27	9.55
PyridineBr1	1.606	134.62	-0.466	1.80	13.25	10.25
PyridineBr2	1.578	135.82	-0.498	1.86	14.93	9.81
PyridineBr3	1.575	135.58	-0.504	1.88	15.39	9.69
PyridineC#N1	1.592	136.62	-0.467	1.84	14.00	10.06
PyridineC#N2	1.584	136.51	-0.483	1.93	17.00	9.26
PyridineC#N3	1.579	136.27	-0.496	1.87	17.01	9.20
PyridineCl1	1.584	134.77	-0.488	1.78	13.53	10.14
PyridineCl2	1.578	135.82	-0.498	1.86	15.36	9.68
PyridineCl3	1.571	135.33	-0.509	1.86	15.21	9.73
PyridineF1	1.578	136.56	-0.479	1.85	14.72	9.86
PyridineF2	1.581	135.93	-0.496	1.87	16.36	9.40
PyridineF3	1.571	135.40	-0.507	1.84	15.16	9.72
PyridineI1	1.564	138.07	-0.499	1.61	10.33	10.93
PyridineI2	1.576	135.49	-0.503	1.84	14.54	9.91
PyridineI3	1.575	135.54	-0.505	1.83	14.69	9.85
PyridineN1	1.528	131.05	-0.574	1.70	13.50	10.08
PyridineN2	1.567	134.31	-0.536	1.91	17.67	9.05
PyridineN3	1.520	131.64	-0.601	1.81	16.36	9.33
PyridineNO2_1	1.619	138.97	-0.425	2.04	16.50	9.52
PyridineNO2_2	1.598	137.66	-0.456	1.88	14.48	9.96
PyridineNO2_3	1.590	137.31	-0.474	1.81	13.13	10.29
PyridineO1	1.538	132.40	-0.499	1.50	11.40	10.50
PyridineO2	1.575	135.17	-0.515	1.84	14.32	9.97
PyridineO3	1.550	133.83	-0.544	1.76	14.30	9.90
PyridineS1	1.569	133.63	-0.495	1.91	15.39	9.72
PyridineS2	1.574	135.41	-0.509	1.85	15.08	9.75

PyridineS3	1.561	134.53	-0.528	1.82	14.93	9.76
PyrimidineBr1	1.618	137.49	-0.421	1.84	15.00	9.77
PyrimidineBr2	1.645	140.66	-0.380	1.99	15.22	9.85
PyrimidineBr3	1.585	137.80	-0.461	1.88	16.73	9.29
PyrimidineC#N1	1.622	139.04	-0.409	1.88	15.61	9.63
PyrimidineC#N2	1.614	139.04	-0.415	2.03	17.61	9.18
PyrimidineC#N3	1.605	138.73	-0.432	1.93	18.99	8.67
PyrimidineCl1	1.602	136.72	-0.445	1.84	16.50	9.32
PyrimidineCl2	1.599	137.53	-0.441	1.87	15.21	9.74
PyrimidineCl3	1.596	137.74	-0.453	1.90	16.86	9.28
PyrimidineF1	1.600	138.97	-0.427	1.91	16.76	9.31
PyrimidineF2	1.597	138.68	-0.433	1.88	14.75	9.89
PyrimidineF3	1.599	137.96	-0.448	1.90	16.98	9.24
PyrimidineI1	1.602	138.14	-0.440	1.94	16.49	9.42
PyrimidineI2	1.610	138.31	-0.429	1.88	13.32	10.31
PyrimidineI3	1.593	137.56	-0.457	1.97	20.93	8.13
PyrimidineN1	1.541	132.77	-0.533	1.63	12.79	10.22
PyrimidineN2	1.537	132.57	-0.540	1.66	12.98	10.19
PyrimidineN3	1.584	136.26	-0.487	1.87	15.16	9.75
PyrimidineNO2_1	1.634	140.82	-0.386	2.02	17.13	9.31
PyrimidineNO2_2	1.651	141.79	-0.366	2.15	17.96	9.19
PyrimidineNO2_3	1.622	140.01	-0.404	1.94	16.19	9.52
PyrimidineO1	1.573	136.07	-0.470	1.84	14.32	9.97
PyrimidineO2	1.546	133.83	-0.464	1.56	11.64	10.49
PyrimidineO3	1.591	137.18	-0.468	1.89	14.99	9.83
PyrimidineS1	1.559	135.56	-0.487	1.61	11.55	10.57
PyrimidineS2	1.578	135.26	-0.461	1.87	15.38	9.69
PyrimidineS3	1.579	137.26	-0.473	1.92	16.66	9.36
PyrroleBr1	1.664	140.02	-0.425	1.87	14.54	9.94
PyrroleBr2	1.659	139.83	-0.431	1.88	14.83	9.86
PyrroleC#N1	1.683	141.65	-0.395	1.95	15.91	9.60
PyrroleC#N2	1.699	142.47	-0.378	1.96	15.03	9.88
PyrroleCl1	1.660	139.97	-0.428	1.84	14.37	9.95
PyrroleCl2	1.653	139.42	-0.440	1.85	14.69	9.87
PyrroleF1	1.671	140.84	-0.409	1.92	17.85	9.00
PyrroleF2	1.656	139.73	-0.435	1.88	15.49	9.67
PyrroleI2	1.651	139.37	-0.440	1.90	14.90	9.86

PyrroleN1	1.639	138.63	-0.462	1.75	14.48	9.83
PyrroleN2	1.606	135.91	-0.525	1.66	12.84	10.23
PyrroleNO2_1	3.401	178.69	0.000	2.77	28.46	6.68
PyrroleNO2_2	3.699	179.62	0.000	2.78	29.06	6.52
PyrroleO1	1.626	137.83	-0.455	1.44	12.67	10.06
PyrroleO2	1.628	137.63	-0.482	1.73	14.40	9.84
PyrroleS1	1.664	140.55	-0.423	1.91	15.53	9.68
PyrroleS2	1.637	138.16	-0.468	1.81	14.60	9.86
ThiazoleBr1	1.564	134.28	-0.500	1.80	14.08	10.00
ThiazoleBr2	1.557	135.10	-0.509	1.87	15.09	9.77
ThiazoleBr3	1.572	134.02	-0.494	1.78	13.96	10.01
ThiazoleC#N1	1.562	135.38	-0.497	1.84	15.42	9.64
ThiazoleC#N2	1.563	135.69	-0.495	1.83	14.46	9.92
ThiazoleC#N3	1.568	136.17	-0.483	1.78	13.46	10.17
ThiazoleCl1	1.554	134.46	-0.514	1.76	13.28	10.20
ThiazoleCl2	1.558	135.13	-0.509	1.85	15.06	9.76
ThiazoleCl3	1.559	134.51	-0.506	1.83	17.83	8.92
ThiazoleF1	1.557	135.58	-0.495	1.90	15.31	9.73
ThiazoleF2	1.558	135.44	-0.507	1.79	14.25	9.94
ThiazoleF3	1.557	135.70	-0.496	1.88	15.51	9.66
ThiazoleI1	1.561	134.76	-0.505	1.81	13.23	10.27
ThiazoleI3	1.557	134.66	-0.509	1.90	13.31	10.33
ThiazoleN1	1.509	131.64	-0.584	1.76	13.81	10.05
ThiazoleN2	1.556	134.25	-0.533	2.02	17.92	9.08
ThiazoleN3	1.534	132.62	-0.546	1.65	12.88	10.21
ThiazoleNO2_1	1.568	136.17	-0.485	1.91	15.28	9.76
ThiazoleNO2_2	1.573	136.67	-0.473	1.91	15.12	9.81
ThiazoleNO2_3	1.587	137.33	-0.455	1.65	11.49	10.62
ThiazoleO1	1.538	133.87	-0.524	1.76	14.51	9.83
ThiazoleO2	1.560	135.30	-0.512	1.81	15.21	9.67
ThiazoleO3	1.543	133.98	-0.519	1.78	14.52	9.85
ThiazoleS1	1.524	133.13	-0.548	1.61	11.53	10.57
ThiazoleS2	1.550	134.51	-0.526	2.06	16.02	9.69
ThiazoleS3	1.535	133.62	-0.530	1.55	11.17	10.62
Tio0	1.579	134.31	-0.570	1.31	11.63	10.25
Tio1	1.577	134.19	-0.576	1.41	12.18	10.18
Tio2	1.575	134.12	-0.579	1.46	9.74	10.96

Tio30	1.600	135.59	-0.546	1.08	5.22	11.92
Tio3V2	1.574	134.11	-0.580	1.34	12.10	10.14
Tio430	1.630	134.09	-0.551	1.71	8.82	11.48
Tio4V2	1.574	134.05	-0.581	1.44	11.33	10.46
Tio50	1.600	136.02	-0.568	1.65	16.10	9.25
Tio5V2	1.567	134.39	-0.585	1.10	6.70	11.50
Tio671	1.613	134.32	-0.555	2.01	16.64	9.45
Tio6V2	1.575	134.19	-0.578	1.58	12.87	10.14
Tio7V2	1.576	134.35	-0.576	1.24	7.67	11.35
Tio8V2	1.574	134.14	-0.580	1.62	15.28	9.46
TriazoleBr2	3.292	178.01	0.001	2.18	22.66	7.82
TriazoleC#N1	3.632	179.60	0.001	0.95	10.06	10.35
TriazoleC#N2	3.319	177.67	0.001	1.77	16.51	9.25
TriazoleCl1	3.623	179.42	0.000	2.33	29.95	5.80
TriazoleCl2	3.529	178.90	0.000	1.90	18.13	8.89
TriazoleF1	3.526	178.82	0.000	2.79	31.39	5.83
TriazoleF2	3.774	179.33	0.000	2.20	24.95	7.16
TriazoleI2	3.659	178.94	0.000	1.69	18.33	8.63
TriazoleN1	3.266	179.62	0.000	2.89	32.13	5.71
TriazoleN2	3.152	177.27	0.000	1.93	16.05	9.55
TriazoleNO2_1	3.661	179.07	0.000	2.71	28.31	6.67
TriazoleNO2_2	3.493	177.85	0.000	2.15	25.45	6.96
TriazoleO1	3.430	178.97	0.000	2.82	29.54	6.42
TriazoleO2	1.681	142.89	-0.307	1.71	14.56	9.77
TriazoleS1	3.496	179.33	0.000	2.65	25.52	7.44
TriazoleS2	3.312	177.69	0.001	1.90	19.56	8.47

Table S15: Parameters calculated using the PM6-DH2 method.

Name	R (Å)	θ (°)	ΔQ	ΔH (kcal/mol)	ΔS (cal/molK)	ΔG (kcal/mol)
Acid0	1.597	136.48	-0.521	1.78	17.04	9.10
Acid1	1.571	135.43	-0.562	1.04	6.82	11.41
Acid2	1.577	134.23	-0.575	1.53	15.38	9.34
Acid30	1.635	138.99	-0.452	1.66	14.14	9.84
Acid3V2	1.575	134.27	-0.576	1.52	17.75	8.63
Acid430	1.735	144.21	-0.363	1.76	9.61	11.29
Acid4V2	1.575	134.21	-0.579	1.48	11.99	10.31

Acid50	1.612	137.19	-0.468	1.67	17.19	8.95
Acid671	1.624	134.54	-0.501	1.61	6.44	12.09
Acid6V2	1.576	134.43	-0.573	1.09	4.89	12.03
Acid7V2	1.574	134.09	-0.581	1.50	20.25	7.87
Acid8V2	1.574	134.17	-0.580	1.67	20.90	7.84
AcidBr1	1.587	135.31	-0.549	1.21	7.79	11.29
AcidBr2	1.577	134.49	-0.569	1.52	12.99	10.05
AcidBr3	1.664	141.16	-0.428	1.71	10.39	11.01
AcidBr4	1.644	139.76	-0.458	1.61	14.89	9.57
AcidBr5	2.968	179.17	-0.001	2.92	28.38	6.86
AcidBr6	1.662	138.49	-0.466	1.42	6.01	12.02
AcidCl0	1.601	136.88	-0.511	1.27	9.01	10.99
AcidCl1	1.583	135.40	-0.552	1.58	15.11	9.47
AcidCl2	1.573	134.77	-0.570	1.47	14.14	9.65
AcidCl3	1.661	141.25	-0.431	1.67	14.64	9.70
AcidCl4	1.635	138.84	-0.473	1.28	10.92	10.43
AcidCl5	3.187	179.85	0.000	2.95	28.49	6.86
AcidCl6	1.652	136.68	-0.484	1.57	9.14	11.25
AcidF0	1.603	136.98	-0.508	1.70	18.00	8.73
AcidF1	1.585	135.19	-0.552	1.59	17.36	8.82
AcidF2	1.578	134.49	-0.569	1.57	13.28	10.01
AcidF3	1.674	141.59	-0.415	1.81	15.06	9.72
AcidF4	1.634	138.61	-0.474	1.59	10.46	10.87
AcidF5	2.973	179.28	-0.001	2.55	19.74	9.06
AcidF6	1.643	136.96	-0.488	1.56	5.43	12.34
AcidI0	1.600	136.82	-0.514	1.20	9.19	10.86
AcidI1	1.600	135.12	-0.544	1.49	12.78	10.08
AcidI2	1.578	134.46	-0.570	1.46	10.58	10.70
AcidI3	1.661	140.79	-0.434	1.76	22.86	7.34
AcidI4	1.642	139.43	-0.462	1.68	13.54	10.05
AcidI5	1.936	156.39	-0.176	1.99	12.04	10.80
AcidI6	1.683	138.31	-0.461	1.52	5.70	12.22
Acid_Halid3V2	1.576	134.28	-0.575	1.52	12.87	10.09
Acid_Halid4V2	1.575	134.17	-0.578	1.59	16.93	8.94
Acid_Halid5V2	1.574	134.16	-0.580	1.27	10.20	10.63
Acid_Halid6V2	1.567	134.37	-0.584	1.38	8.20	11.34
Acid_Halid7V2	1.572	133.88	-0.582	1.55	12.82	10.13

Acid_Halid8V2	1.575	134.26	-0.578	1.23	9.43	10.82
Acid_Sulf0	1.583	135.08	-0.557	1.33	10.90	10.48
Acid_Sulf1	1.579	134.25	-0.574	1.39	10.85	10.56
Acid_Sulf2	1.576	134.28	-0.576	1.56	16.54	9.03
Acid_Sulf30	1.615	136.61	-0.511	1.64	14.94	9.58
Acid_Sulf3V2	1.576	134.39	-0.576	1.57	9.51	11.14
Acid_Sulf430	1.642	136.87	-0.487	1.88	11.63	10.81
Acid_Sulf4V2	1.573	133.97	-0.581	1.54	9.86	11.00
Acid_Sulf50	1.600	136.93	-0.542	1.72	17.86	8.79
Acid_Sulf5V2	1.557	134.60	-0.595	1.07	5.79	11.74
Acid_Sulf671	1.624	135.30	-0.533	1.52	8.33	11.43
Acid_Sulf6V2	1.575	134.21	-0.578	1.70	13.92	9.95
Acid_Sulf7V2	1.574	134.12	-0.581	1.44	14.04	9.66
Acid_Sulf8V2	1.574	134.18	-0.579	1.43	13.24	9.89
Acid_Tio0	1.600	135.62	-0.527	1.30	9.99	10.72
Acid_Tio1	1.580	134.63	-0.564	1.40	11.27	10.44
Acid_Tio2	1.600	134.49	-0.561	1.19	10.52	10.45
Acid_Tio30	1.632	139.67	-0.475	1.61	11.65	10.54
Acid_Tio3V2	1.574	134.15	-0.579	1.59	15.03	9.51
Acid_Tio430	1.696	141.55	-0.409	2.05	17.53	9.22
Acid_Tio4V2	1.575	134.06	-0.579	1.64	15.46	9.43
Acid_Tio50	1.606	135.40	-0.523	1.39	8.06	11.38
Acid_Tio5V2	1.574	134.05	-0.581	1.33	11.57	10.29
Acid_Tio671	1.610	132.58	-0.546	0.87	-6.42	15.19
Acid_Tio6V2	1.568	134.07	-0.584	1.23	8.39	11.13
Acid_Tio7V2	1.574	134.05	-0.582	1.27	11.37	10.28
Acid_Tio8V2	1.575	134.27	-0.578	1.71	14.00	9.94
Alcohol0	1.576	134.52	-0.569	1.36	13.15	9.84
Alcohol1	1.572	134.80	-0.575	1.25	10.32	10.57
Alcohol2	1.574	134.27	-0.578	1.50	13.29	9.93
Alcohol30	1.590	134.54	-0.556	1.10	6.09	11.68
Alcohol3V2	1.574	134.18	-0.578	1.57	15.15	9.45
Alcohol430	1.625	134.55	-0.522	1.65	12.87	10.22
Alcohol4V2	1.568	134.42	-0.583	1.18	8.07	11.17
Alcohol50	1.600	136.44	-0.562	1.19	10.22	10.54
Alcohol5V2	1.575	134.09	-0.580	1.39	9.20	11.05
Alcohol671	1.598	131.92	-0.561	1.06	0.37	13.35

Alcohol6V2	1.574	134.18	-0.579	1.51	15.71	9.22
Alcohol7V2	1.574	134.18	-0.580	1.46	11.17	10.53
Alcohol8V2	1.574	134.05	-0.580	1.77	14.28	9.92
Aldehyde0	1.585	135.25	-0.551	1.49	15.53	9.25
Aldehyde1	1.573	134.36	-0.572	1.21	10.44	10.50
Aldehyde2	1.575	134.47	-0.571	1.55	14.70	9.57
Aldehyde30	1.615	136.80	-0.519	1.41	9.22	11.06
Aldehyde430	1.646	137.37	-0.491	1.38	4.98	12.30
Aldehyde50	1.601	136.13	-0.532	1.04	4.55	12.08
Aldehyde671	1.621	135.37	-0.537	1.81	11.88	10.67
Amide0	1.591	135.33	-0.532	1.31	11.42	10.31
Amide1	1.580	134.82	-0.566	1.55	14.44	9.65
Amide2	1.577	134.63	-0.571	1.54	15.05	9.45
Amide30	1.633	137.81	-0.475	1.58	9.42	11.17
Amide3V2	1.574	134.13	-0.579	1.57	18.47	8.46
Amide430	1.680	140.14	-0.435	1.87	11.57	10.83
Amide4V2	1.574	134.05	-0.579	1.61	14.27	9.76
Amide50	1.615	136.75	-0.510	1.45	12.14	10.23
Amide5V2	1.573	133.89	-0.586	0.99	6.96	11.32
Amide671	1.641	136.11	-0.515	1.51	1.85	13.36
Amide6V2	1.566	135.02	-0.583	1.05	3.80	12.32
Amide7V2	1.575	134.20	-0.579	1.48	10.41	10.77
Amide8V2	1.574	134.18	-0.579	1.54	11.76	10.43
Amine0	1.577	134.12	-0.576	1.64	18.21	8.62
Amine1	1.574	134.21	-0.583	1.44	17.30	8.69
Amine2	1.573	134.18	-0.583	1.52	14.81	9.50
Amine30	1.573	134.54	-0.577	1.67	13.24	10.13
Amine3V2	1.567	134.48	-0.585	1.11	1.83	12.97
Amine430	1.609	133.89	-0.557	1.49	4.81	12.46
Amine4V2	1.567	134.39	-0.585	1.34	10.31	10.67
Amine50	1.589	134.41	-0.570	1.97	16.84	9.35
Amine5V2	1.574	134.05	-0.580	1.31	8.75	11.10
Amine671	1.600	132.37	-0.575	1.45	8.07	11.45
Amine6V2	1.566	134.49	-0.585	1.47	14.67	9.49
Amine7V2	1.574	134.19	-0.579	1.43	8.61	11.26
Amine8V2	1.574	134.18	-0.580	1.54	14.76	9.54
Anhydride0	1.600	136.26	-0.520	1.37	10.17	10.74

Anhydride1	1.583	134.96	-0.555	1.44	10.17	10.80
Anhydride2	1.575	134.80	-0.568	1.38	6.77	11.76
Anhydride30	1.663	140.89	-0.425	1.42	5.28	12.25
Anhydride3V2	1.577	134.42	-0.573	1.10	6.13	11.67
Anhydride430	3.089	179.67	-0.001	2.26	11.51	11.23
Anhydride4V2	1.574	134.19	-0.578	1.50	12.43	10.20
Anhydride50	1.622	136.54	-0.508	1.85	14.13	10.03
Anhydride5V2	1.573	134.13	-0.580	1.28	9.38	10.88
Anhydride671	1.638	136.34	-0.496	1.80	6.73	12.20
Anhydride7V2	1.574	134.00	-0.582	1.22	6.34	11.73
Anhydride8V2	1.575	134.19	-0.579	1.45	10.66	10.68
AzolidineBr1	1.600	136.30	-0.525	1.07	4.66	12.08
AzolidineBr2	1.591	134.80	-0.560	1.65	13.79	9.94
AzolidineC#N1	1.600	138.59	-0.516	1.53	10.98	10.65
AzolidineC#N2	1.600	137.08	-0.554	1.52	12.24	10.27
AzolidineCl1	1.604	136.70	-0.518	1.22	10.17	10.59
AzolidineCl2	1.600	134.51	-0.550	1.44	10.83	10.61
AzolidineF1	1.600	136.27	-0.525	1.43	9.89	10.88
AzolidineF2	1.591	134.82	-0.562	1.73	15.53	9.50
AzolidineI1	1.600	136.59	-0.531	1.02	2.56	12.65
AzolidineI2	1.589	134.71	-0.562	1.69	12.66	10.31
AzolidineN1	1.575	133.99	-0.579	1.57	13.62	9.91
AzolidineN2	1.585	134.13	-0.577	1.65	14.21	9.82
AzolidineNO2_1	1.600	137.27	-0.517	1.30	8.82	11.07
AzolidineNO2_2	1.600	135.96	-0.540	1.34	9.90	10.79
AzolidineO1	1.582	134.96	-0.559	1.53	9.75	11.03
AzolidineO2	1.600	134.59	-0.551	1.48	12.32	10.21
AzolidineS1	1.600	135.11	-0.550	1.31	5.37	12.11
AzolidineS2	1.586	134.83	-0.560	1.47	10.83	10.64
Br0	1.602	136.30	-0.538	1.56	13.77	9.86
Br1	1.583	134.91	-0.561	1.33	12.88	9.89
Br2	1.574	134.77	-0.571	1.48	14.29	9.62
Br3	1.664	139.84	-0.455	1.50	11.12	10.58
Br4	1.636	138.06	-0.490	1.60	12.25	10.35
Br5	3.337	178.56	0.000	2.88	34.28	5.06
Br6	1.628	135.66	-0.515	1.83	15.37	9.65
Cl0	1.604	136.85	-0.514	1.23	10.71	10.44

Cl1	1.579	134.57	-0.569	1.42	14.38	9.54
Cl2	1.577	134.49	-0.571	1.30	10.02	10.71
Cl3	1.651	139.75	-0.450	1.57	13.77	9.87
Cl4	1.631	138.52	-0.482	1.50	10.12	10.88
Cl5	3.230	179.27	-0.007	2.87	30.87	6.07
Cl6	1.643	137.22	-0.496	2.21	23.23	7.69
Cyan0	1.602	136.85	-0.515	1.64	15.02	9.57
Cyan1	1.581	134.47	-0.563	1.38	14.69	9.40
Cyan2	1.576	134.10	-0.577	1.50	14.72	9.51
Cyan30	1.643	139.63	-0.462	1.76	16.72	9.18
Cyan3V2	1.576	134.33	-0.575	1.48	14.47	9.57
Cyan430	1.722	143.18	-0.367	2.03	14.23	10.19
Cyan4V2	1.568	134.36	-0.582	1.34	12.33	10.06
Cyan50	1.619	137.77	-0.503	1.57	8.35	11.48
Cyan5V2	1.568	134.57	-0.583	1.17	9.43	10.76
Cyan671	1.634	136.00	-0.502	1.81	15.65	9.55
Cyan6V2	1.574	134.11	-0.580	1.52	13.92	9.77
Cyan7V2	1.574	134.16	-0.580	1.43	10.50	10.70
Cyan8V2	1.574	134.15	-0.580	1.62	13.11	10.12
Ester0	1.600	135.36	-0.525	1.55	11.47	10.53
Ester1	1.582	134.95	-0.558	1.48	14.07	9.69
Ester2	1.577	134.33	-0.572	1.46	14.80	9.45
Ester30	1.637	138.78	-0.471	1.81	15.83	9.49
Ester3V2	1.576	134.32	-0.576	1.33	8.76	11.12
Ester430	1.706	141.37	-0.413	1.81	11.68	10.73
Ester4V2	1.575	134.23	-0.577	1.52	13.01	10.04
Ester50	1.600	136.44	-0.537	1.27	6.83	11.63
Ester5V2	1.571	134.10	-0.582	1.30	10.02	10.71
Ester671	1.634	136.15	-0.511	1.69	3.88	12.93
Ester6V2	1.575	134.15	-0.578	1.70	14.90	9.66
Ester7V2	1.573	134.07	-0.581	1.45	12.36	10.16
Ester8V2	1.574	134.12	-0.580	1.79	20.90	7.96
Ether0	1.600	135.87	-0.525	1.30	9.61	10.84
Ether1	1.577	134.52	-0.569	1.28	13.08	9.78
Ether2	1.570	134.61	-0.579	1.36	12.56	10.02
Ether30	1.611	136.37	-0.519	1.54	8.70	11.34
Ether3V2	1.572	134.06	-0.580	1.37	10.80	10.55

Ether430	1.629	135.28	-0.515	1.78	12.65	10.41
Ether4V2	1.568	134.15	-0.584	1.82	21.85	7.71
Ether50	1.601	135.20	-0.543	1.55	11.35	10.57
Ether5V2	1.568	134.47	-0.584	1.27	7.32	11.49
Ether671	1.620	135.11	-0.540	2.06	16.35	9.59
Ether6V2	1.574	134.15	-0.580	1.80	20.85	7.99
Ether7V2	1.575	134.20	-0.578	1.59	13.68	9.91
Ether8V2	1.576	134.34	-0.576	1.32	8.91	11.06
F0	1.598	136.44	-0.522	1.54	15.47	9.33
F1	1.584	134.65	-0.568	1.37	12.50	10.04
F2	1.580	134.59	-0.568	1.21	9.91	10.65
F3	1.661	140.38	-0.434	1.82	16.61	9.27
F4	1.615	136.24	-0.511	1.63	12.61	10.27
F5	3.063	179.43	-0.001	2.84	30.18	6.24
F6	1.620	134.85	-0.534	1.43	4.98	12.35
Halid2V2	1.578	134.56	-0.570	1.46	15.33	9.29
Halid3V2	1.575	134.29	-0.577	1.34	12.68	9.96
Halid4V2	1.572	133.94	-0.586	1.35	11.70	10.26
Halid5V2	1.574	134.09	-0.580	1.39	14.62	9.43
Halid6V2	1.571	133.96	-0.583	1.46	12.59	10.11
Halid7V2	1.567	134.37	-0.585	1.41	14.27	9.56
Halid8V2	1.574	134.16	-0.580	1.55	14.65	9.59
I0	1.600	136.75	-0.534	1.30	11.52	10.27
I1	1.575	134.99	-0.562	1.26	11.19	10.33
I2	1.573	134.65	-0.573	1.22	9.21	10.87
I3	1.648	139.10	-0.468	1.71	15.12	9.61
I4	1.624	137.63	-0.503	1.45	9.91	10.90
I5	1.696	141.41	-0.415	1.84	13.50	10.22
I6	1.627	135.98	-0.522	1.77	11.48	10.74
ImidazoleBr1	3.062	178.09	0.000	2.62	25.10	7.54
ImidazoleC#N1	3.448	178.66	0.000	2.70	35.30	4.58
ImidazoleC#N2	3.297	178.60	0.000	2.78	30.74	6.02
ImidazoleCl1	3.323	178.92	0.000	2.74	29.54	6.33
ImidazoleF1	3.092	178.83	0.000	2.69	26.93	7.07
ImidazoleF2	3.372	178.79	0.000	2.70	27.78	6.82
ImidazoleN1	1.651	140.12	-0.426	1.71	15.37	9.53
ImidazoleN2	1.678	141.34	-0.390	1.79	14.69	9.81

ImidazoleNO2_1	3.083	176.48	0.001	2.67	24.48	7.77
ImidazoleNO2_2	3.226	177.50	0.000	2.82	30.79	6.04
ImidazoleO1	1.651	140.10	-0.393	1.45	11.99	10.27
ImidazoleO2	1.670	141.43	-0.370	1.42	10.85	10.59
ImidazoleS1	3.327	178.87	0.000	2.66	25.67	7.40
ImidazoleS2	3.535	178.99	0.000	2.65	25.33	7.50
Ketone3V2	1.570	134.40	-0.579	1.41	10.17	10.78
Ketone4V2	1.573	134.16	-0.580	1.64	15.63	9.38
Ketone5V2	1.574	133.75	-0.586	1.20	9.08	10.89
Ketone6V2	1.574	134.05	-0.580	1.25	8.37	11.16
Ketone7V2	1.570	134.04	-0.583	1.50	16.30	9.04
Ketone8V2	1.574	134.16	-0.580	1.61	13.97	9.84
Nitro0	1.600	135.87	-0.534	1.23	11.73	10.14
Nitro1	1.600	135.39	-0.548	1.15	10.88	10.31
Nitro3	1.575	134.13	-0.581	1.48	14.47	9.56
Nitro30	1.624	138.24	-0.496	1.73	11.60	10.67
Nitro430	1.671	139.09	-0.436	1.62	8.83	11.38
Nitro4V2	1.568	134.36	-0.583	0.93	3.65	12.24
Nitro50	1.611	136.84	-0.523	1.61	13.45	10.00
Nitro5V2	1.573	134.66	-0.579	1.36	9.23	11.01
Nitro671	1.610	133.82	-0.548	1.72	13.53	10.08
Nitro6V2	1.574	134.16	-0.579	1.59	12.38	10.29
Nitro7V2	1.574	134.07	-0.580	1.25	7.02	11.56
Nitro8V2	1.574	134.17	-0.580	1.60	15.47	9.39
PiperidineBr1	1.612	135.42	-0.527	1.67	11.83	10.55
PiperidineBr2	1.600	134.51	-0.560	1.52	10.20	10.88
PiperidineBr3	1.600	133.59	-0.569	1.54	10.42	10.83
PiperidineC#N1	1.611	137.04	-0.517	1.84	15.03	9.76
PiperidineC#N2	1.600	136.33	-0.563	1.69	12.36	10.41
PiperidineC#N3	1.600	135.81	-0.549	1.55	11.11	10.64
PiperidineCl1	1.600	136.84	-0.544	1.37	9.33	10.99
PiperidineCl2	1.600	135.35	-0.546	1.48	10.59	10.72
PiperidineCl3	1.600	133.42	-0.570	1.55	11.00	10.67
PiperidineF1	1.600	136.00	-0.531	1.52	10.77	10.71
PiperidineF2	1.600	134.13	-0.567	1.58	11.76	10.48
PiperidineF3	1.590	134.79	-0.565	1.81	16.58	9.27
PiperidineI1	1.612	136.76	-0.518	1.78	13.23	10.24

PiperidineI2	1.600	136.06	-0.546	1.41	8.93	11.15
PiperidineI3	1.590	135.17	-0.561	1.78	13.39	10.19
PiperidineN1	1.600	134.97	-0.542	1.51	11.04	10.62
PiperidineN2	1.589	134.78	-0.569	1.64	12.73	10.25
PiperidineN3	1.585	134.58	-0.573	1.74	14.61	9.79
PiperidineNO2_1	1.628	137.60	-0.494	1.92	17.97	8.96
PiperidineNO2_2	1.600	135.62	-0.548	1.64	12.51	10.31
PiperidineNO2_3	1.600	135.14	-0.570	1.56	12.35	10.28
PiperidineO1	1.600	135.16	-0.530	1.58	11.49	10.56
PiperidineO2	1.588	135.06	-0.559	1.52	11.68	10.44
PiperidineO3	1.585	134.94	-0.562	1.84	15.73	9.55
PiperidineS1	1.600	135.94	-0.556	1.56	13.73	9.87
PiperidineS2	1.584	133.39	-0.584	1.76	13.74	10.06
PiperidineS3	1.600	133.42	-0.575	1.57	11.08	10.67
PyrazineBr1	1.663	138.76	-0.386	1.91	14.78	9.91
PyrazineBr2	1.617	138.81	-0.433	1.93	15.59	9.68
PyrazineC#N1	1.641	140.08	-0.394	1.95	16.31	9.48
PyrazineC#N2	1.627	139.65	-0.415	1.90	14.62	9.94
PyrazineCl1	1.622	137.98	-0.426	1.82	13.91	10.07
PyrazineCl2	1.607	138.64	-0.444	1.92	16.74	9.33
PyrazineF1	1.623	139.95	-0.410	1.91	15.76	9.61
PyrazineF2	1.615	138.87	-0.434	1.90	16.03	9.53
PyrazineI1	1.631	139.18	-0.415	2.00	16.20	9.57
PyrazineI2	1.609	138.45	-0.443	1.88	14.85	9.85
PyrazineN1	1.542	132.79	-0.538	1.62	12.49	10.30
PyrazineN2	1.591	136.21	-0.493	1.95	17.35	9.18
PyrazineNO2_1	1.674	142.75	-0.351	1.85	12.36	10.57
PyrazineNO2_2	1.641	140.93	-0.390	1.96	15.00	9.89
PyrazineO1	1.586	136.51	-0.461	1.84	15.73	9.56
PyrazineO2	1.609	137.88	-0.454	1.89	15.81	9.58
PyrazineS1	1.577	136.18	-0.468	1.60	11.10	10.69
PyrazineS2	1.601	137.68	-0.461	1.90	15.47	9.69
PyrazoleBr1	3.504	179.72	0.000	2.93	31.84	5.83
PyrazoleBr2	1.836	150.69	-0.233	2.14	18.46	9.04
PyrazoleC#N1	3.452	178.84	0.000	2.82	32.88	5.42
PyrazoleC#N2	3.378	178.90	0.000	2.76	31.41	5.80
PyrazoleCl1	3.360	179.92	0.000	2.85	28.55	6.74

PyrazoleCl2	3.284	179.14	0.000	2.67	28.34	6.62
PyrazoleF1	3.303	179.64	0.000	2.84	29.23	6.53
PyrazoleF2	3.421	179.79	0.000	2.86	30.47	6.17
PyrazoleI1	3.328	179.02	0.000	2.77	29.24	6.46
PyrazoleI2	3.248	179.84	0.000	2.88	29.98	6.34
PyrazoleN1	1.638	137.92	-0.481	2.01	16.71	9.43
PyrazoleN2	1.663	139.59	-0.440	1.97	18.84	8.76
PyrazoleNO2_1	3.392	178.46	0.000	2.80	30.73	6.04
PyrazoleNO2_2	3.439	178.88	0.000	2.61	27.83	6.72
PyrazoleO1	1.685	141.58	-0.398	1.76	14.56	9.82
PyrazoleO2	1.722	143.57	-0.359	1.86	14.97	9.80
PyrazoleS1	3.327	179.50	0.000	1.20	13.76	9.50
PyrazoleS2	1.723	143.58	-0.353	1.97	15.86	9.64
PyridineBr1	1.606	134.63	-0.466	1.80	13.25	10.25
PyridineBr2	1.578	135.81	-0.498	1.86	14.95	9.80
PyridineBr3	1.575	135.58	-0.504	1.88	15.39	9.69
PyridineC#N1	1.592	136.61	-0.467	1.83	13.92	10.08
PyridineC#N2	1.582	136.57	-0.485	1.84	15.66	9.57
PyridineC#N3	1.579	136.27	-0.496	1.87	17.02	9.20
PyridineCl1	1.584	134.79	-0.488	1.78	13.54	10.14
PyridineCl2	1.579	135.83	-0.498	1.86	15.41	9.67
PyridineCl3	1.571	135.33	-0.509	1.86	15.13	9.75
PyridineF1	1.578	136.56	-0.479	1.84	14.72	9.86
PyridineF2	1.582	135.97	-0.495	1.86	15.52	9.64
PyridineF3	1.571	135.40	-0.507	1.84	15.11	9.73
PyridineI1	1.582	138.42	-0.481	1.85	13.47	10.24
PyridineI2	1.575	135.49	-0.503	1.84	14.54	9.91
PyridineI3	1.575	135.54	-0.505	1.83	14.69	9.85
PyridineN1	1.528	131.05	-0.573	1.68	13.40	10.08
PyridineN2	1.567	134.32	-0.536	1.93	21.00	8.07
PyridineN3	1.521	131.66	-0.601	1.83	16.23	9.39
PyridineNO2_1	1.618	138.97	-0.425	2.02	16.97	9.36
PyridineNO2_2	1.598	137.66	-0.456	1.89	14.58	9.94
PyridineNO2_3	1.590	137.31	-0.474	1.81	13.23	10.27
PyridineO1	1.539	132.39	-0.498	1.54	11.64	10.47
PyridineO2	1.574	135.19	-0.515	1.83	14.68	9.86
PyridineO3	1.550	133.86	-0.544	1.77	14.36	9.89

PyridineS1	1.569	133.64	-0.495	1.90	16.33	9.43
PyridineS2	1.576	135.28	-0.508	2.20	18.53	9.07
PyridineS3	1.561	134.53	-0.528	1.82	14.93	9.77
PyrimidineBr1	1.618	137.49	-0.421	1.84	14.99	9.77
PyrimidineBr2	1.646	140.69	-0.379	1.95	14.75	9.96
PyrimidineBr3	1.585	137.81	-0.461	1.88	16.74	9.29
PyrimidineC#N1	1.622	139.04	-0.409	1.88	15.65	9.62
PyrimidineC#N2	1.614	139.05	-0.415	2.04	18.15	9.03
PyrimidineC#N3	1.605	138.73	-0.432	1.93	18.99	8.67
PyrimidineCl1	1.602	136.73	-0.445	1.86	17.68	8.99
PyrimidineCl2	1.599	137.53	-0.441	1.87	15.22	9.73
PyrimidineCl3	1.596	137.74	-0.453	1.90	16.85	9.28
PyrimidineF1	1.601	139.00	-0.427	1.91	17.32	9.15
PyrimidineF2	1.597	138.68	-0.433	1.88	14.75	9.89
PyrimidineF3	1.599	137.96	-0.448	1.90	16.98	9.24
PyrimidineI1	1.562	138.32	-0.459	1.59	11.63	10.52
PyrimidineI2	1.611	138.65	-0.425	1.89	13.89	10.15
PyrimidineI3	1.601	137.71	-0.448	1.97	18.48	8.86
PyrimidineN1	1.540	132.69	-0.532	1.59	12.52	10.26
PyrimidineN2	1.536	132.53	-0.540	1.63	12.64	10.26
PyrimidineN3	1.580	135.98	-0.494	1.84	14.03	10.06
PyrimidineNO2_1	1.640	141.09	-0.380	1.98	19.17	8.66
PyrimidineNO2_2	1.651	141.79	-0.366	2.09	16.46	9.59
PyrimidineNO2_3	1.622	140.01	-0.404	1.94	16.10	9.54
PyrimidineO1	1.573	136.07	-0.470	1.84	14.31	9.98
PyrimidineO2	1.548	133.86	-0.462	1.59	11.87	10.46
PyrimidineO3	1.591	137.19	-0.468	1.72	13.73	10.02
PyrimidineS1	1.556	135.15	-0.488	1.63	11.94	10.47
PyrimidineS2	1.578	135.24	-0.461	1.92	15.36	9.74
PyrimidineS3	1.579	137.26	-0.473	1.92	16.39	9.43
PyrroleBr1	1.664	140.13	-0.424	1.83	13.69	10.14
PyrroleBr2	1.659	139.88	-0.430	1.89	15.02	9.81
PyrroleC#N1	1.684	141.75	-0.394	1.93	15.30	9.77
PyrroleC#N2	1.699	142.44	-0.378	1.96	14.96	9.90
PyrroleCl1	1.660	139.97	-0.428	1.89	15.69	9.62
PyrroleCl2	1.653	139.42	-0.440	1.82	14.63	9.86
PyrroleF1	1.673	140.95	-0.408	1.91	15.96	9.55

PyrroleF2	1.656	139.66	-0.436	1.79	14.07	9.99
PyrroleI2	1.649	139.31	-0.441	1.91	15.17	9.79
PyrroleN1	1.636	138.53	-0.464	1.73	15.40	9.54
PyrroleN2	1.600	136.88	-0.528	1.48	12.46	10.17
PyrroleNO2_1	1.733	145.39	-0.331	2.26	19.93	8.71
PyrroleNO2_2	3.499	179.25	0.000	2.66	25.79	7.37
PyrroleO1	1.634	139.96	-0.441	1.48	12.14	10.26
PyrroleO2	1.631	137.74	-0.479	1.59	13.30	10.02
PyrroleS1	1.659	139.89	-0.431	1.87	15.35	9.70
PyrroleS2	1.636	138.14	-0.468	1.71	13.59	10.06
ThiazoleBr1	1.564	134.28	-0.500	1.80	14.08	10.00
ThiazoleBr2	1.557	135.10	-0.509	1.87	15.09	9.77
ThiazoleBr3	1.574	134.06	-0.492	1.80	14.78	9.80
ThiazoleC#N1	1.562	135.38	-0.497	1.84	15.54	9.61
ThiazoleC#N2	1.563	135.69	-0.495	1.83	14.46	9.92
ThiazoleC#N3	1.568	136.15	-0.483	1.79	13.55	10.15
ThiazoleCl1	1.554	134.47	-0.514	1.78	13.66	10.11
ThiazoleCl2	1.558	135.13	-0.509	1.85	15.07	9.75
ThiazoleCl3	1.558	134.49	-0.506	1.83	17.91	8.89
ThiazoleF1	1.557	135.58	-0.495	1.90	15.31	9.73
ThiazoleF2	1.558	135.43	-0.507	1.79	14.25	9.94
ThiazoleF3	1.557	135.69	-0.496	1.88	15.52	9.66
ThiazoleI1	1.561	134.77	-0.505	1.82	13.33	10.25
ThiazoleI3	1.557	134.66	-0.509	1.90	13.32	10.33
ThiazoleN1	1.509	131.65	-0.583	1.58	12.22	10.33
ThiazoleN2	1.556	134.25	-0.533	1.96	16.45	9.45
ThiazoleN3	1.533	132.57	-0.547	1.72	13.35	10.14
ThiazoleNO2_1	1.571	136.18	-0.483	1.92	15.23	9.78
ThiazoleNO2_2	1.573	136.62	-0.472	1.97	18.08	8.98
ThiazoleNO2_3	1.587	137.34	-0.455	1.67	12.04	10.48
ThiazoleO1	1.538	133.87	-0.524	1.76	14.51	9.83
ThiazoleO2	1.560	135.30	-0.512	1.85	15.90	9.51
ThiazoleO3	1.544	133.99	-0.519	1.82	15.29	9.66
ThiazoleS1	1.524	133.17	-0.548	1.58	11.23	10.64
ThiazoleS2	1.550	134.51	-0.526	2.06	15.98	9.70
ThiazoleS3	1.535	133.62	-0.530	1.53	10.97	10.66
Tio0	1.577	134.44	-0.570	1.57	15.21	9.44

Tio1	1.577	134.19	-0.576	1.32	10.56	10.57
Tio2	1.575	134.12	-0.579	1.48	12.74	10.09
Tio30	1.600	135.63	-0.547	1.14	6.94	11.47
Tio3V2	1.574	134.11	-0.580	1.36	12.63	9.99
Tio430	1.630	134.35	-0.557	1.78	10.20	11.14
Tio4V2	1.574	134.05	-0.581	1.42	11.35	10.44
Tio50	1.600	136.02	-0.568	1.66	16.24	9.21
Tio5V2	1.567	134.39	-0.585	1.06	5.75	11.74
Tio671	1.613	134.32	-0.555	2.01	16.64	9.45
Tio6V2	1.574	134.16	-0.579	1.66	15.35	9.49
Tio7V2	1.576	134.35	-0.576	1.23	7.53	11.38
Tio8V2	1.574	134.14	-0.580	1.62	15.28	9.46
TriazoleBr2	3.290	178.20	0.001	2.18	23.31	7.63
TriazoleC#N1	3.629	179.56	0.001	1.26	14.15	9.45
TriazoleC#N2	3.304	177.62	0.001	1.76	16.31	9.30
TriazoleCl1	3.477	178.49	0.000	2.70	28.42	6.62
TriazoleCl2	3.175	177.47	0.000	1.74	15.67	9.47
TriazoleF1	3.423	178.80	0.000	2.85	42.16	2.68
TriazoleF2	3.582	179.16	0.000	2.01	19.27	8.67
TriazoleI2	3.567	179.94	0.001	-0.72	-9.02	14.37
TriazoleN1	3.224	179.81	0.000	2.86	29.90	6.35
TriazoleN2	3.137	177.52	0.000	1.92	16.93	9.27
TriazoleNO2_1	3.632	179.70	0.002	0.44	6.33	10.95
TriazoleNO2_2	3.196	176.65	0.001	2.06	21.50	8.05
TriazoleO1	3.447	178.90	0.000	2.85	31.49	5.86
TriazoleO2	3.083	178.72	0.001	1.92	18.50	8.80
TriazoleS1	3.349	178.71	0.001	2.98	34.95	4.96
TriazoleS2	3.289	177.57	0.001	1.32	10.57	10.56

Table S16: Parameters calculated using the PM6-DH2X method.

Name	R (Å)	θ (°)	ΔQ	ΔH (kcal/mol)	ΔS (cal/molK)	ΔG (kcal/mol)
Acid0	1.597	136.48	-0.521	1.78	17.04	9.10
Acid1	1.571	135.43	-0.562	1.04	6.82	11.41
Acid2	1.577	134.23	-0.575	1.53	15.38	9.34
Acid30	1.635	138.99	-0.452	1.66	14.14	9.84
Acid3V2	1.575	134.27	-0.576	1.52	17.75	8.63

Acid430	1.735	144.21	-0.363	1.76	9.61	11.29
Acid4V2	1.575	134.21	-0.579	1.48	11.99	10.31
Acid50	1.612	137.19	-0.468	1.67	17.19	8.95
Acid671	1.624	134.54	-0.501	1.61	6.44	12.09
Acid6V2	1.576	134.43	-0.573	1.09	4.89	12.03
Acid7V2	1.574	134.09	-0.581	1.50	20.25	7.87
Acid8V2	1.574	134.17	-0.580	1.67	20.90	7.84
AcidBr1	1.587	135.33	-0.549	1.40	11.52	10.37
AcidBr2	1.577	134.46	-0.569	1.46	11.77	10.35
AcidBr3	1.674	141.61	-0.417	1.55	10.28	10.88
AcidBr4	1.653	139.91	-0.451	1.36	6.20	11.91
AcidBr5	3.260	179.52	0.000	2.83	29.21	6.52
AcidBr6	1.662	138.52	-0.464	1.84	10.87	11.00
AcidCl0	1.604	136.92	-0.509	1.18	10.11	10.56
AcidCl1	1.584	135.45	-0.550	1.24	8.35	11.15
AcidCl2	1.574	134.78	-0.569	1.34	11.26	10.38
AcidCl3	1.665	141.42	-0.426	1.59	15.26	9.44
AcidCl4	1.640	139.20	-0.466	1.39	12.09	10.19
AcidCl5	3.112	179.84	-0.001	2.89	29.91	6.38
AcidCl6	1.655	137.13	-0.478	1.51	8.81	11.28
AcidF0	1.603	136.98	-0.508	1.70	18.00	8.73
AcidF1	1.585	135.19	-0.552	1.59	17.36	8.82
AcidF2	1.578	134.49	-0.569	1.57	13.28	10.01
AcidF3	1.674	141.59	-0.415	1.81	15.06	9.72
AcidF4	1.634	138.61	-0.474	1.59	10.46	10.87
AcidF5	2.973	179.28	-0.001	2.55	19.74	9.06
AcidF6	1.643	136.96	-0.488	1.56	5.43	12.34
AcidI0	1.600	136.74	-0.512	1.77	16.66	9.20
AcidI1	1.587	135.26	-0.548	1.58	16.00	9.21
AcidI2	1.574	134.53	-0.571	1.22	6.31	11.73
AcidI3	1.665	141.04	-0.429	1.63	9.70	11.14
AcidI4	1.643	139.56	-0.462	1.49	8.29	11.42
AcidI5	2.862	179.44	-0.002	2.58	21.78	8.49
AcidI6	1.651	137.96	-0.479	1.75	13.40	10.16
Acid_Halid3V2	1.576	134.28	-0.575	1.52	12.87	10.09
Acid_Halid4V2	1.575	134.17	-0.578	1.59	16.93	8.94
Acid_Halid5V2	1.574	134.16	-0.580	1.27	10.20	10.63

Acid_Halid6V2	1.567	134.37	-0.584	1.38	8.20	11.34
Acid_Halid7V2	1.572	133.88	-0.582	1.55	12.82	10.13
Acid_Halid8V2	1.575	134.26	-0.578	1.23	9.43	10.82
Acid_Sulf0	1.583	135.08	-0.557	1.33	10.90	10.48
Acid_Sulf1	1.579	134.25	-0.574	1.39	10.85	10.56
Acid_Sulf2	1.576	134.28	-0.576	1.56	16.54	9.03
Acid_Sulf30	1.615	136.61	-0.511	1.64	14.94	9.58
Acid_Sulf3V2	1.576	134.39	-0.576	1.57	9.51	11.14
Acid_Sulf430	1.642	136.87	-0.487	1.88	11.63	10.81
Acid_Sulf4V2	1.573	133.97	-0.581	1.54	9.86	11.00
Acid_Sulf50	1.600	136.93	-0.542	1.72	17.86	8.79
Acid_Sulf5V2	1.557	134.60	-0.595	1.07	5.79	11.74
Acid_Sulf671	1.624	135.30	-0.533	1.52	8.33	11.43
Acid_Sulf6V2	1.575	134.21	-0.578	1.70	13.92	9.95
Acid_Sulf7V2	1.574	134.12	-0.581	1.44	14.04	9.66
Acid_Sulf8V2	1.574	134.18	-0.579	1.43	13.24	9.89
Acid_Tio0	1.600	135.62	-0.527	1.30	9.99	10.72
Acid_Tio1	1.580	134.63	-0.564	1.40	11.27	10.44
Acid_Tio2	1.600	134.49	-0.561	1.19	10.52	10.45
Acid_Tio30	1.632	139.67	-0.475	1.61	11.65	10.54
Acid_Tio3V2	1.574	134.15	-0.579	1.59	15.03	9.51
Acid_Tio430	1.696	141.55	-0.409	2.05	17.53	9.22
Acid_Tio4V2	1.575	134.06	-0.579	1.64	15.46	9.43
Acid_Tio50	1.606	135.40	-0.523	1.39	8.06	11.38
Acid_Tio5V2	1.574	134.05	-0.581	1.33	11.57	10.29
Acid_Tio671	1.610	132.58	-0.546	0.87	-6.42	15.19
Acid_Tio6V2	1.568	134.07	-0.584	1.23	8.39	11.13
Acid_Tio7V2	1.574	134.05	-0.582	1.27	11.37	10.28
Acid_Tio8V2	1.575	134.27	-0.578	1.71	14.00	9.94
Alcohol0	1.576	134.52	-0.569	1.36	13.15	9.84
Alcohol1	1.572	134.80	-0.575	1.25	10.32	10.57
Alcohol2	1.574	134.27	-0.578	1.50	13.29	9.93
Alcohol30	1.590	134.54	-0.556	1.10	6.09	11.68
Alcohol3V2	1.574	134.18	-0.578	1.57	15.15	9.45
Alcohol430	1.625	134.55	-0.522	1.65	12.87	10.22
Alcohol4V2	1.568	134.42	-0.583	1.18	8.07	11.17
Alcohol50	1.600	136.44	-0.562	1.19	10.22	10.54

Alcohol5V2	1.575	134.09	-0.580	1.39	9.20	11.05
Alcohol671	1.598	131.92	-0.561	1.06	0.37	13.35
Alcohol6V2	1.574	134.18	-0.579	1.51	15.71	9.22
Alcohol7V2	1.574	134.18	-0.580	1.46	11.17	10.53
Alcohol8V2	1.574	134.05	-0.580	1.77	14.28	9.92
Aldehyde0	1.585	135.25	-0.551	1.49	15.53	9.25
Aldehyde1	1.573	134.36	-0.572	1.21	10.44	10.50
Aldehyde2	1.575	134.47	-0.571	1.55	14.70	9.57
Aldehyde30	1.615	136.80	-0.519	1.41	9.22	11.06
Aldehyde430	1.646	137.37	-0.491	1.38	4.98	12.30
Aldehyde50	1.601	136.13	-0.532	1.04	4.55	12.08
Aldehyde671	1.621	135.37	-0.537	1.81	11.88	10.67
Amide0	1.591	135.33	-0.532	1.31	11.42	10.31
Amide1	1.580	134.82	-0.566	1.55	14.44	9.65
Amide2	1.577	134.63	-0.571	1.54	15.05	9.45
Amide30	1.633	137.81	-0.475	1.58	9.42	11.17
Amide3V2	1.574	134.13	-0.579	1.57	18.47	8.46
Amide430	1.680	140.14	-0.435	1.87	11.57	10.83
Amide4V2	1.574	134.05	-0.579	1.61	14.27	9.76
Amide50	1.615	136.75	-0.510	1.45	12.14	10.23
Amide5V2	1.573	133.89	-0.586	0.99	6.96	11.32
Amide671	1.641	136.11	-0.515	1.51	1.85	13.36
Amide6V2	1.566	135.02	-0.583	1.05	3.80	12.32
Amide7V2	1.575	134.20	-0.579	1.48	10.41	10.77
Amide8V2	1.574	134.18	-0.579	1.54	11.76	10.43
Amine0	1.577	134.12	-0.576	1.64	18.21	8.62
Amine1	1.574	134.21	-0.583	1.44	17.30	8.69
Amine2	1.573	134.18	-0.583	1.52	14.81	9.50
Amine30	1.573	134.54	-0.577	1.67	13.24	10.13
Amine3V2	1.567	134.48	-0.585	1.11	1.83	12.97
Amine430	1.609	133.89	-0.557	1.49	4.81	12.46
Amine4V2	1.567	134.39	-0.585	1.34	10.31	10.67
Amine50	1.589	134.41	-0.570	1.97	16.84	9.35
Amine5V2	1.574	134.05	-0.580	1.31	8.75	11.10
Amine671	1.600	132.37	-0.575	1.45	8.07	11.45
Amine6V2	1.566	134.49	-0.585	1.47	14.67	9.49
Amine7V2	1.574	134.19	-0.579	1.43	8.61	11.26

Amine8V2	1.574	134.18	-0.580	1.54	14.76	9.54
Anhydride0	1.600	136.26	-0.520	1.37	10.17	10.74
Anhydride1	1.583	134.96	-0.555	1.44	10.17	10.80
Anhydride2	1.575	134.80	-0.568	1.38	6.77	11.76
Anhydride30	1.663	140.89	-0.425	1.42	5.28	12.25
Anhydride3V2	1.577	134.42	-0.573	1.10	6.13	11.67
Anhydride430	3.089	179.67	-0.001	2.26	11.51	11.23
Anhydride4V2	1.574	134.19	-0.578	1.50	12.43	10.20
Anhydride50	1.622	136.54	-0.508	1.85	14.13	10.03
Anhydride5V2	1.573	134.13	-0.580	1.28	9.38	10.88
Anhydride671	1.638	136.34	-0.496	1.80	6.73	12.20
Anhydride7V2	1.574	134.00	-0.582	1.22	6.34	11.73
Anhydride8V2	1.575	134.19	-0.579	1.45	10.66	10.68
AzolidineBr1	1.600	137.35	-0.539	0.90	3.06	12.39
AzolidineBr2	1.600	135.00	-0.544	1.49	12.50	10.17
AzolidineC#N1	1.600	138.59	-0.516	1.53	10.98	10.65
AzolidineC#N2	1.600	137.08	-0.554	1.52	12.24	10.27
AzolidineCl1	1.604	136.75	-0.516	1.22	10.02	10.64
AzolidineCl2	1.600	134.51	-0.550	1.44	10.84	10.61
AzolidineF1	1.600	136.27	-0.525	1.43	9.89	10.88
AzolidineF2	1.591	134.82	-0.562	1.73	15.53	9.50
AzolidineI1	1.600	135.85	-0.528	1.06	2.77	12.63
AzolidineI2	1.589	134.75	-0.561	1.70	13.27	10.15
AzolidineN1	1.575	133.99	-0.579	1.57	13.62	9.91
AzolidineN2	1.585	134.13	-0.577	1.65	14.21	9.82
AzolidineNO2_1	1.600	137.27	-0.517	1.30	8.82	11.07
AzolidineNO2_2	1.600	135.96	-0.540	1.34	9.90	10.79
AzolidineO1	1.582	134.96	-0.559	1.53	9.75	11.03
AzolidineO2	1.600	134.59	-0.551	1.48	12.32	10.21
AzolidineS1	1.600	135.11	-0.550	1.31	5.37	12.11
AzolidineS2	1.586	134.83	-0.560	1.47	10.83	10.64
Br0	1.600	138.35	-0.539	1.17	9.50	10.74
Br1	1.582	135.10	-0.555	1.19	9.85	10.65
Br2	1.574	134.78	-0.571	1.43	13.94	9.68
Br3	1.655	139.94	-0.447	1.72	16.40	9.23
Br4	1.633	138.48	-0.480	1.12	4.72	12.11
Br5	3.236	179.04	0.000	2.90	26.06	7.53

Br6	1.626	135.52	-0.516	1.79	13.02	10.31
Cl0	1.607	137.07	-0.507	1.12	11.18	10.19
Cl1	1.581	134.63	-0.565	1.27	11.24	10.32
Cl2	1.577	134.49	-0.571	1.30	10.02	10.71
Cl3	1.653	140.05	-0.442	1.79	14.65	9.82
Cl4	1.636	138.91	-0.471	1.54	11.49	10.51
Cl5	3.203	178.60	-0.004	2.96	31.14	6.07
Cl6	1.644	137.27	-0.495	2.29	23.16	7.78
Cyan0	1.602	136.85	-0.515	1.64	15.02	9.57
Cyan1	1.581	134.47	-0.563	1.38	14.69	9.40
Cyan2	1.576	134.10	-0.577	1.50	14.72	9.51
Cyan30	1.643	139.63	-0.462	1.76	16.72	9.18
Cyan3V2	1.576	134.33	-0.575	1.48	14.47	9.57
Cyan430	1.722	143.18	-0.367	2.03	14.23	10.19
Cyan4V2	1.568	134.36	-0.582	1.34	12.33	10.06
Cyan50	1.619	137.77	-0.503	1.57	8.35	11.48
Cyan5V2	1.568	134.57	-0.583	1.17	9.43	10.76
Cyan671	1.634	136.00	-0.502	1.81	15.65	9.55
Cyan6V2	1.574	134.11	-0.580	1.52	13.92	9.77
Cyan7V2	1.574	134.16	-0.580	1.43	10.50	10.70
Cyan8V2	1.574	134.15	-0.580	1.62	13.11	10.12
Ester0	1.600	135.36	-0.525	1.55	11.47	10.53
Ester1	1.582	134.95	-0.558	1.48	14.07	9.69
Ester2	1.577	134.33	-0.572	1.46	14.80	9.45
Ester30	1.637	138.78	-0.471	1.81	15.83	9.49
Ester3V2	1.576	134.32	-0.576	1.33	8.76	11.12
Ester430	1.706	141.37	-0.413	1.81	11.68	10.73
Ester4V2	1.575	134.23	-0.577	1.52	13.01	10.04
Ester50	1.600	136.44	-0.537	1.27	6.83	11.63
Ester5V2	1.571	134.10	-0.582	1.30	10.02	10.71
Ester671	1.634	136.15	-0.511	1.69	3.88	12.93
Ester6V2	1.575	134.15	-0.578	1.70	14.90	9.66
Ester7V2	1.573	134.07	-0.581	1.45	12.36	10.16
Ester8V2	1.574	134.12	-0.580	1.79	20.90	7.96
Ether0	1.600	135.87	-0.525	1.30	9.61	10.84
Ether1	1.577	134.52	-0.569	1.28	13.08	9.78
Ether2	1.570	134.61	-0.579	1.36	12.56	10.02

Ether30	1.611	136.37	-0.519	1.54	8.70	11.34
Ether3V2	1.572	134.06	-0.580	1.37	10.80	10.55
Ether430	1.629	135.28	-0.515	1.78	12.65	10.41
Ether4V2	1.568	134.15	-0.584	1.82	21.85	7.71
Ether50	1.601	135.20	-0.543	1.55	11.35	10.57
Ether5V2	1.568	134.47	-0.584	1.27	7.32	11.49
Ether671	1.620	135.11	-0.540	2.06	16.35	9.59
Ether6V2	1.574	134.15	-0.580	1.80	20.85	7.99
Ether7V2	1.575	134.20	-0.578	1.59	13.68	9.91
Ether8V2	1.576	134.34	-0.576	1.32	8.91	11.06
F0	1.598	136.44	-0.522	1.54	15.47	9.33
F1	1.584	134.65	-0.568	1.37	12.50	10.04
F2	1.580	134.59	-0.568	1.21	9.91	10.65
F3	1.661	140.38	-0.434	1.82	16.61	9.27
F4	1.615	136.24	-0.511	1.63	12.61	10.27
F5	3.063	179.43	-0.001	2.84	30.18	6.24
F6	1.620	134.85	-0.534	1.43	4.98	12.35
Halid2V2	1.578	134.56	-0.570	1.46	15.33	9.29
Halid3V2	1.575	134.29	-0.577	1.34	12.68	9.96
Halid4V2	1.572	133.94	-0.586	1.35	11.70	10.26
Halid5V2	1.574	134.09	-0.580	1.39	14.62	9.43
Halid6V2	1.571	133.96	-0.583	1.46	12.59	10.11
Halid7V2	1.567	134.37	-0.585	1.41	14.27	9.56
Halid8V2	1.574	134.16	-0.580	1.55	14.65	9.59
I0	1.600	135.93	-0.534	0.84	5.92	11.48
I1	1.575	134.99	-0.562	1.25	11.07	10.35
I2	1.573	134.65	-0.573	1.22	9.21	10.87
I3	1.647	139.09	-0.469	1.68	14.33	9.81
I4	1.625	137.78	-0.499	1.52	11.87	10.38
I5	1.717	142.09	-0.393	1.81	13.73	10.12
I6	1.628	136.01	-0.521	1.70	7.26	11.94
ImidazoleBr1	3.359	178.88	0.000	2.81	27.31	7.07
ImidazoleBr2	3.644	179.79	0.000	2.88	30.05	6.33
ImidazoleC#N1	3.448	178.66	0.000	2.70	35.30	4.58
ImidazoleC#N2	3.297	178.60	0.000	2.78	30.74	6.02
ImidazoleF1	3.092	178.83	0.000	2.69	26.93	7.07
ImidazoleF2	3.372	178.79	0.000	2.70	27.78	6.82

ImidazoleI1	3.362	179.19	0.000	2.40	21.64	8.34
ImidazoleN1	1.651	140.12	-0.426	1.71	15.37	9.53
ImidazoleN2	1.678	141.34	-0.390	1.79	14.69	9.81
ImidazoleNO2_1	3.083	176.48	0.001	2.67	24.48	7.77
ImidazoleNO2_2	3.226	177.50	0.000	2.82	30.79	6.04
ImidazoleO1	1.651	140.10	-0.393	1.45	11.99	10.27
ImidazoleO2	1.670	141.43	-0.370	1.42	10.85	10.59
ImidazoleS1	3.327	178.87	0.000	2.66	25.67	7.40
ImidazoleS2	3.535	178.99	0.000	2.65	25.33	7.50
Ketone3V2	1.570	134.40	-0.579	1.41	10.17	10.78
Ketone4V2	1.573	134.16	-0.580	1.64	15.63	9.38
Ketone5V2	1.574	133.75	-0.586	1.20	9.08	10.89
Ketone6V2	1.574	134.05	-0.580	1.25	8.37	11.16
Ketone7V2	1.570	134.04	-0.583	1.50	16.30	9.04
Ketone8V2	1.574	134.16	-0.580	1.61	13.97	9.84
Nitro0	1.600	135.87	-0.534	1.23	11.73	10.14
Nitro1	1.600	135.39	-0.548	1.15	10.88	10.31
Nitro3	1.575	134.13	-0.581	1.48	14.47	9.56
Nitro30	1.624	138.24	-0.496	1.73	11.60	10.67
Nitro430	1.671	139.09	-0.436	1.62	8.83	11.38
Nitro4V2	1.568	134.36	-0.583	0.93	3.65	12.24
Nitro50	1.611	136.84	-0.523	1.61	13.45	10.00
Nitro5V2	1.573	134.66	-0.579	1.36	9.23	11.01
Nitro671	1.610	133.82	-0.548	1.72	13.53	10.08
Nitro6V2	1.574	134.16	-0.579	1.59	12.38	10.29
Nitro7V2	1.574	134.07	-0.580	1.25	7.02	11.56
Nitro8V2	1.574	134.17	-0.580	1.60	15.47	9.39
PiperidineBr1	1.600	136.93	-0.545	1.42	9.01	11.13
PiperidineBr2	1.600	134.51	-0.560	1.55	10.65	10.77
PiperidineBr3	1.600	133.61	-0.569	1.54	10.58	10.78
PiperidineC#N1	1.611	137.04	-0.517	1.84	15.03	9.76
PiperidineC#N2	1.600	136.33	-0.563	1.69	12.36	10.41
PiperidineC#N3	1.600	135.81	-0.549	1.55	11.11	10.64
PiperidineCl1	1.600	136.81	-0.546	1.41	9.47	10.99
PiperidineCl2	1.600	135.35	-0.546	1.48	10.59	10.72
PiperidineCl3	1.600	133.42	-0.570	1.55	11.00	10.67
PiperidineF1	1.600	136.00	-0.531	1.52	10.77	10.71

PiperidineF2	1.600	134.13	-0.567	1.58	11.76	10.48
PiperidineF3	1.590	134.79	-0.565	1.81	16.58	9.27
PiperidineI1	1.612	136.76	-0.518	1.78	13.20	10.24
PiperidineI2	1.600	136.06	-0.546	1.41	8.93	11.15
PiperidineI3	1.590	135.17	-0.561	1.78	13.39	10.19
PiperidineN1	1.600	134.97	-0.542	1.51	11.04	10.62
PiperidineN2	1.589	134.78	-0.569	1.64	12.73	10.25
PiperidineN3	1.585	134.58	-0.573	1.74	14.61	9.79
PiperidineNO2_1	1.628	137.60	-0.494	1.92	17.97	8.96
PiperidineNO2_2	1.600	135.62	-0.548	1.64	12.51	10.31
PiperidineNO2_3	1.600	135.14	-0.570	1.56	12.35	10.28
PiperidineO1	1.600	135.16	-0.530	1.58	11.49	10.56
PiperidineO2	1.588	135.06	-0.559	1.52	11.68	10.44
PiperidineO3	1.585	134.94	-0.562	1.84	15.73	9.55
PiperidineS1	1.600	135.94	-0.556	1.56	13.73	9.87
PiperidineS2	1.584	133.39	-0.584	1.76	13.74	10.06
PiperidineS3	1.600	133.42	-0.575	1.57	11.08	10.67
PyrazineBr1	1.636	137.70	-0.415	1.91	15.83	9.59
PyrazineBr2	1.618	138.93	-0.431	1.93	15.23	9.79
PyrazineC#N1	1.641	140.08	-0.394	1.95	16.31	9.48
PyrazineC#N2	1.627	139.65	-0.415	1.90	14.62	9.94
PyrazineCl1	1.630	137.71	-0.420	1.96	15.60	9.71
PyrazineCl2	1.617	138.75	-0.435	1.90	15.43	9.70
PyrazineF1	1.623	139.95	-0.410	1.91	15.76	9.61
PyrazineF2	1.615	138.87	-0.434	1.90	16.03	9.53
PyrazineN1	1.542	132.79	-0.538	1.62	12.49	10.30
PyrazineN2	1.591	136.21	-0.493	1.95	17.35	9.18
PyrazineNO2_1	1.674	142.75	-0.351	1.85	12.36	10.57
PyrazineNO2_2	1.641	140.93	-0.390	1.96	15.00	9.89
PyrazineO1	1.586	136.51	-0.461	1.84	15.73	9.56
PyrazineO2	1.609	137.88	-0.454	1.89	15.81	9.58
PyrazineS1	1.577	136.18	-0.468	1.60	11.10	10.69
PyrazineS2	1.601	137.68	-0.461	1.90	15.47	9.69
PyrazoleBr1	3.333	178.88	0.000	2.79	30.99	5.95
PyrazoleBr2	3.343	179.26	0.000	2.83	30.46	6.15
PyrazoleC#N1	3.452	178.84	0.000	2.82	32.88	5.42
PyrazoleC#N2	3.378	178.90	0.000	2.76	31.41	5.80

PyrazoleCl1	3.297	179.90	0.000	2.87	29.23	6.55
PyrazoleCl2	3.283	179.08	0.000	2.70	28.57	6.58
PyrazoleF1	3.303	179.64	0.000	2.84	29.23	6.53
PyrazoleF2	3.421	179.79	0.000	2.86	30.47	6.17
PyrazoleI1	3.613	179.63	0.000	2.80	27.64	6.96
PyrazoleI2	3.184	179.09	0.000	2.92	32.50	5.63
PyrazoleN1	1.638	137.92	-0.481	2.01	16.71	9.43
PyrazoleN2	1.663	139.59	-0.440	1.97	18.84	8.76
PyrazoleNO2_1	3.392	178.46	0.000	2.80	30.73	6.04
PyrazoleNO2_2	3.439	178.88	0.000	2.61	27.83	6.72
PyrazoleO1	1.685	141.58	-0.398	1.76	14.56	9.82
PyrazoleO2	1.722	143.57	-0.359	1.86	14.97	9.80
PyrazoleS1	3.327	179.50	0.000	1.20	13.76	9.50
PyrazoleS2	1.723	143.58	-0.353	1.97	15.86	9.64
PyridineBr1	1.598	134.52	-0.476	1.80	12.99	10.32
PyridineBr2	1.578	135.82	-0.498	1.86	14.99	9.79
PyridineBr3	1.575	135.58	-0.504	1.88	15.16	9.76
PyridineC#N1	1.592	136.61	-0.467	1.83	13.92	10.08
PyridineC#N2	1.582	136.57	-0.485	1.84	15.66	9.57
PyridineC#N3	1.579	136.27	-0.496	1.87	17.02	9.20
PyridineCl1	1.587	134.22	-0.487	1.76	13.17	10.24
PyridineCl2	1.579	135.83	-0.498	1.86	15.41	9.67
PyridineCl3	1.571	135.33	-0.509	1.86	15.13	9.75
PyridineF1	1.578	136.56	-0.479	1.84	14.72	9.86
PyridineF2	1.582	135.97	-0.495	1.86	15.52	9.64
PyridineF3	1.571	135.40	-0.507	1.84	15.11	9.73
PyridineI1	1.587	134.47	-0.489	1.79	12.98	10.32
PyridineI2	1.575	135.49	-0.503	1.84	14.54	9.91
PyridineI3	1.575	135.54	-0.505	1.83	14.69	9.85
PyridineN1	1.528	131.05	-0.573	1.68	13.40	10.08
PyridineN2	1.567	134.32	-0.536	1.93	21.00	8.07
PyridineN3	1.521	131.66	-0.601	1.83	16.23	9.39
PyridineNO2_1	1.618	138.97	-0.425	2.02	16.97	9.36
PyridineNO2_2	1.598	137.66	-0.456	1.89	14.58	9.94
PyridineNO2_3	1.590	137.31	-0.474	1.81	13.23	10.27
PyridineO1	1.539	132.39	-0.498	1.54	11.64	10.47
PyridineO2	1.574	135.19	-0.515	1.83	14.68	9.86

PyridineO3	1.550	133.86	-0.544	1.77	14.36	9.89
PyridineS1	1.569	133.64	-0.495	1.90	16.33	9.43
PyridineS2	1.576	135.28	-0.508	2.20	18.53	9.07
PyridineS3	1.561	134.53	-0.528	1.82	14.93	9.77
PyrimidineBr1	1.622	139.04	-0.411	1.92	16.00	9.55
PyrimidineBr2	1.618	137.82	-0.421	1.97	16.30	9.51
PyrimidineBr3	1.585	137.80	-0.461	1.85	15.96	9.49
PyrimidineC#N1	1.622	139.04	-0.409	1.88	15.65	9.62
PyrimidineC#N2	1.614	139.05	-0.415	2.04	18.15	9.03
PyrimidineC#N3	1.605	138.73	-0.432	1.93	18.99	8.67
PyrimidineCl1	1.611	138.45	-0.425	1.92	16.22	9.49
PyrimidineCl2	1.606	138.16	-0.430	1.90	14.97	9.83
PyrimidineCl3	1.596	137.74	-0.453	1.90	16.85	9.28
PyrimidineF1	1.601	139.00	-0.427	1.91	17.32	9.15
PyrimidineF2	1.597	138.68	-0.433	1.88	14.75	9.89
PyrimidineF3	1.599	137.96	-0.448	1.90	16.98	9.24
PyrimidineI1	1.631	139.61	-0.401	1.97	17.56	9.14
PyrimidineI2	1.611	137.51	-0.429	1.93	14.88	9.89
PyrimidineI3	1.601	137.71	-0.448	1.97	18.48	8.86
PyrimidineN1	1.540	132.69	-0.532	1.59	12.52	10.26
PyrimidineN2	1.536	132.53	-0.540	1.63	12.64	10.26
PyrimidineN3	1.580	135.98	-0.494	1.84	14.03	10.06
PyrimidineNO2_1	1.640	141.09	-0.380	1.98	19.17	8.66
PyrimidineNO2_2	1.651	141.79	-0.366	2.09	16.46	9.59
PyrimidineNO2_3	1.622	140.01	-0.404	1.94	16.10	9.54
PyrimidineO1	1.573	136.07	-0.470	1.84	14.31	9.98
PyrimidineO2	1.548	133.86	-0.462	1.59	11.87	10.46
PyrimidineO3	1.591	137.19	-0.468	1.72	13.73	10.02
PyrimidineS1	1.556	135.15	-0.488	1.63	11.94	10.47
PyrimidineS2	1.578	135.24	-0.461	1.92	15.36	9.74
PyrimidineS3	1.579	137.26	-0.473	1.92	16.39	9.43
PyrroleBr2	1.660	139.88	-0.430	1.92	16.44	9.42
PyrroleC#N1	1.684	141.75	-0.394	1.93	15.30	9.77
PyrroleC#N2	1.699	142.44	-0.378	1.96	14.96	9.90
PyrroleCl1	1.659	139.93	-0.429	1.83	14.30	9.97
PyrroleCl2	1.654	139.47	-0.439	1.84	14.47	9.93
PyrroleF1	1.673	140.95	-0.408	1.91	15.96	9.55

PyrroleF2	1.656	139.66	-0.436	1.79	14.07	9.99
PyrroleI2	1.649	139.31	-0.441	1.92	15.18	9.79
PyrroleN1	1.636	138.53	-0.464	1.73	15.40	9.54
PyrroleN2	1.600	136.88	-0.528	1.48	12.46	10.17
PyrroleNO2_1	1.733	145.39	-0.331	2.26	19.93	8.71
PyrroleNO2_2	3.499	179.25	0.000	2.66	25.79	7.37
PyrroleO1	1.634	139.96	-0.441	1.48	12.14	10.26
PyrroleO2	1.631	137.74	-0.479	1.59	13.30	10.02
PyrroleS1	1.659	139.89	-0.431	1.87	15.35	9.70
PyrroleS2	1.636	138.14	-0.468	1.71	13.59	10.06
ThiazoleBr1	1.566	134.70	-0.498	1.85	14.76	9.85
ThiazoleBr2	1.557	135.10	-0.509	1.86	15.06	9.77
ThiazoleBr3	1.567	134.15	-0.500	1.84	14.84	9.81
ThiazoleC#N1	1.562	135.38	-0.497	1.84	15.54	9.61
ThiazoleC#N2	1.563	135.69	-0.495	1.83	14.46	9.92
ThiazoleC#N3	1.568	136.15	-0.483	1.79	13.55	10.15
ThiazoleCl1	1.556	134.25	-0.512	1.76	13.34	10.19
ThiazoleCl2	1.558	135.13	-0.509	1.85	15.07	9.75
ThiazoleCl3	1.559	134.10	-0.507	1.76	13.33	10.19
ThiazoleF1	1.557	135.58	-0.495	1.90	15.31	9.73
ThiazoleF2	1.558	135.43	-0.507	1.79	14.25	9.94
ThiazoleF3	1.557	135.69	-0.496	1.88	15.52	9.66
ThiazoleI1	1.564	134.26	-0.504	1.84	14.41	9.94
ThiazoleI2	1.554	134.86	-0.514	1.86	15.12	9.75
ThiazoleN1	1.509	131.65	-0.583	1.58	12.22	10.33
ThiazoleN2	1.556	134.25	-0.533	1.96	16.45	9.45
ThiazoleN3	1.533	132.57	-0.547	1.72	13.35	10.14
ThiazoleNO2_1	1.571	136.18	-0.483	1.92	15.23	9.78
ThiazoleNO2_2	1.573	136.62	-0.472	1.97	18.08	8.98
ThiazoleNO2_3	1.587	137.34	-0.455	1.67	12.04	10.48
ThiazoleO1	1.538	133.87	-0.524	1.76	14.51	9.83
ThiazoleO2	1.560	135.30	-0.512	1.85	15.90	9.51
ThiazoleO3	1.544	133.99	-0.519	1.82	15.29	9.66
ThiazoleS1	1.524	133.17	-0.548	1.58	11.23	10.64
ThiazoleS2	1.550	134.51	-0.526	2.06	15.98	9.70
ThiazoleS3	1.535	133.62	-0.530	1.53	10.97	10.66
Tio0	1.577	134.44	-0.570	1.57	15.21	9.44

Tio1	1.577	134.19	-0.576	1.32	10.56	10.57
Tio2	1.575	134.12	-0.579	1.48	12.74	10.09
Tio30	1.600	135.63	-0.547	1.14	6.94	11.47
Tio3V2	1.574	134.11	-0.580	1.36	12.63	9.99
Tio430	1.630	134.35	-0.557	1.78	10.20	11.14
Tio4V2	1.574	134.05	-0.581	1.42	11.35	10.44
Tio50	1.600	136.02	-0.568	1.66	16.24	9.21
Tio5V2	1.567	134.39	-0.585	1.06	5.75	11.74
Tio671	1.613	134.32	-0.555	2.01	16.64	9.45
Tio6V2	1.574	134.16	-0.579	1.66	15.35	9.49
Tio7V2	1.576	134.35	-0.576	1.23	7.53	11.38
Tio8V2	1.574	134.14	-0.580	1.62	15.28	9.46
TriazoleBr1	3.475	179.25	0.001	2.54	22.22	8.32
TriazoleBr2	3.542	178.97	0.000	1.89	23.21	7.37
TriazoleC#N1	3.629	179.56	0.001	1.26	14.15	9.45
TriazoleC#N2	3.304	177.62	0.001	1.76	16.31	9.30
TriazoleCl1	3.568	179.31	0.000	2.59	25.28	7.45
TriazoleCl2	3.456	178.78	0.000	2.10	22.77	7.71
TriazoleF1	3.423	178.80	0.000	2.85	42.16	2.68
TriazoleF2	3.582	179.16	0.000	2.01	19.27	8.67
TriazoleI2	3.616	179.46	0.000	1.11	7.16	11.38
TriazoleN1	3.224	179.81	0.000	2.86	29.90	6.35
TriazoleN2	3.137	177.52	0.000	1.92	16.93	9.27
TriazoleNO2_1	3.632	179.70	0.002	0.44	6.33	10.95
TriazoleNO2_2	3.196	176.65	0.001	2.06	21.50	8.05
TriazoleO1	3.447	178.90	0.000	2.85	31.49	5.86
TriazoleO2	3.083	178.72	0.001	1.92	18.50	8.80
TriazoleS1	3.349	178.71	0.001	2.98	34.95	4.96
TriazoleS2	3.289	177.57	0.001	1.32	10.57	10.56

Table S17: Parameters calculated using the PM7 method.

Name	R (Å)	θ (°)	ΔQ	ΔH (kcal/mol)	ΔS (cal/molK)	ΔG (kcal/mol)
Acid0	1.581	137.04	-0.532	1.69	14.73	9.70
Acid1	1.572	135.19	-0.554	1.44	14.25	9.60
Acid2	1.572	134.44	-0.571	1.22	10.62	10.46
Acid30	1.636	139.67	-0.445	2.16	20.36	8.49

Acid3V2	1.563	134.68	-0.571	1.39	13.41	9.80
Acid430	2.677	179.35	-0.003	1.47	4.96	12.39
Acid4V2	1.566	134.64	-0.571	1.46	14.40	9.57
Acid50	1.600	137.76	-0.479	0.95	6.15	11.52
Acid671	1.635	137.37	-0.492	1.70	9.53	11.27
Acid6V2	1.569	134.87	-0.565	1.25	10.28	10.59
Acid7V2	1.566	134.63	-0.572	1.26	14.43	9.36
Acid8V2	1.566	134.66	-0.572	1.56	13.91	9.82
AcidBr1	1.576	135.46	-0.551	1.34	10.53	10.60
AcidBr2	1.567	134.68	-0.569	1.59	14.68	9.61
AcidBr3	1.671	142.40	-0.414	1.89	14.12	10.08
AcidBr4	1.655	140.59	-0.441	1.56	10.86	10.73
AcidBr5	2.705	177.54	-0.005	2.82	24.79	7.83
AcidBr6	1.669	139.48	-0.446	1.81	15.52	9.58
AcidCl0	1.600	137.48	-0.504	1.71	16.98	9.05
AcidCl1	1.573	135.30	-0.553	1.35	12.91	9.91
AcidCl2	1.564	134.85	-0.569	1.28	10.73	10.49
AcidCl3	1.674	143.04	-0.407	1.79	16.55	9.26
AcidCl4	1.654	140.29	-0.442	1.35	6.28	11.89
AcidCl5	3.167	179.51	0.003	2.86	27.91	6.94
AcidCl6	3.537	178.58	0.001	2.50	17.45	9.70
AcidF0	1.600	137.46	-0.507	1.58	14.87	9.55
AcidF1	1.576	135.47	-0.553	1.62	17.56	8.79
AcidF2	1.566	134.73	-0.572	1.43	10.85	10.60
AcidF3	1.852	151.59	-0.259	1.82	11.43	10.81
AcidF4	1.628	139.06	-0.463	1.56	9.24	11.21
AcidF5	3.196	179.55	0.003	2.77	29.00	6.53
AcidF6	1.638	137.88	-0.479	1.52	6.44	12.00
AcidI0	1.601	137.10	-0.510	1.01	5.42	11.80
AcidI1	1.575	135.28	-0.553	1.52	14.13	9.71
AcidI2	1.564	134.57	-0.573	1.37	8.91	11.12
AcidI3	1.662	141.64	-0.428	1.92	14.62	9.96
AcidI4	1.646	140.00	-0.452	1.66	13.80	9.94
AcidI5	3.075	178.83	-0.001	2.90	22.75	8.52
AcidI6	3.038	178.16	0.000	2.55	18.14	9.54
Acid_Halid3V2	1.575	134.60	-0.564	1.47	13.00	10.00
Acid_Halid4V2	1.568	134.79	-0.568	1.25	10.06	10.66

Acid_Halid5V2	1.555	135.42	-0.580	1.01	4.04	12.21
Acid_Halid6V2	1.575	134.56	-0.567	1.25	3.50	12.61
Acid_Halid7V2	1.564	134.69	-0.572	1.46	14.40	9.57
Acid_Halid8V2	1.565	134.47	-0.573	1.57	12.61	10.21
Acid_Sulf0	1.569	134.59	-0.570	1.63	13.88	9.89
Acid_Sulf1	1.565	134.01	-0.577	1.31	9.12	10.99
Acid_Sulf2	1.565	134.62	-0.570	1.58	12.96	10.12
Acid_Sulf30	1.600	135.87	-0.530	1.37	7.19	11.63
Acid_Sulf3V2	1.568	134.69	-0.567	1.64	14.30	9.78
Acid_Sulf430	1.614	135.99	-0.516	1.68	4.76	12.66
Acid_Sulf4V2	1.566	134.64	-0.571	1.53	11.87	10.40
Acid_Sulf50	1.600	134.77	-0.546	1.30	10.15	10.68
Acid_Sulf5V2	1.567	134.77	-0.571	1.05	4.11	12.23
Acid_Sulf671	1.601	134.72	-0.547	1.27	4.77	12.25
Acid_Sulf6V2	1.567	134.68	-0.570	1.49	14.28	9.64
Acid_Sulf7V2	1.567	134.76	-0.570	0.99	8.04	11.00
Acid_Sulf8V2	1.567	134.79	-0.570	1.31	8.88	11.07
Acid_Tio0	1.575	136.90	-0.538	1.07	9.56	10.63
Acid_Tio1	1.565	135.40	-0.567	1.00	8.01	11.01
Acid_Tio2	1.600	135.48	-0.548	1.10	7.20	11.36
Acid_Tio30	1.635	139.97	-0.465	1.43	10.46	10.71
Acid_Tio3V2	1.565	134.54	-0.572	1.51	15.43	9.31
Acid_Tio430	2.827	177.71	-0.003	2.63	25.15	7.53
Acid_Tio4V2	1.567	134.70	-0.570	1.16	2.72	12.76
Acid_Tio50	1.600	144.02	-0.495	1.08	1.02	13.18
Acid_Tio5V2	1.566	134.48	-0.574	1.38	11.23	10.43
Acid_Tio671	1.617	136.15	-0.506	1.27	3.13	12.74
Acid_Tio6V2	1.566	134.39	-0.572	1.26	8.47	11.13
Acid_Tio7V2	1.566	134.53	-0.573	1.19	10.02	10.60
Acid_Tio8V2	1.565	134.45	-0.574	1.63	14.01	9.86
Alcohol0	1.565	134.93	-0.568	1.40	14.42	9.50
Alcohol1	1.562	134.82	-0.573	1.47	12.38	10.18
Alcohol2	1.567	134.73	-0.571	1.31	11.98	10.14
Alcohol30	1.576	134.74	-0.555	1.32	10.16	10.69
Alcohol3V2	1.565	134.56	-0.572	1.32	10.56	10.57
Alcohol430	1.621	135.70	-0.495	1.61	12.26	10.36
Alcohol4V2	1.561	134.70	-0.575	1.09	8.16	11.06

Alcohol50	1.600	135.19	-0.544	1.61	10.64	10.84
Alcohol5V2	1.566	134.64	-0.572	1.28	9.76	10.77
Alcohol671	1.587	133.48	-0.551	1.24	6.67	11.65
Alcohol6V2	1.566	134.76	-0.571	1.44	10.28	10.78
Alcohol7V2	1.566	134.64	-0.573	1.59	13.68	9.91
Alcohol8V2	1.565	134.54	-0.573	1.44	10.14	10.82
Aldehyde0	1.566	136.40	-0.556	1.30	12.13	10.09
Aldehyde1	1.567	134.86	-0.562	1.12	9.77	10.61
Aldehyde2	1.566	134.59	-0.571	1.40	11.84	10.28
Aldehyde30	1.600	143.34	-0.516	1.36	8.94	11.10
Aldehyde430	1.644	138.78	-0.467	1.76	9.86	11.22
Aldehyde50	1.600	143.42	-0.514	1.20	5.83	11.86
Aldehyde671	1.611	136.05	-0.523	1.82	19.41	8.44
Amide0	1.582	135.69	-0.538	1.34	12.43	10.04
Amide1	1.575	135.60	-0.564	1.31	9.35	10.92
Amide2	1.568	135.08	-0.568	1.58	19.76	8.10
Amide30	1.666	141.83	-0.425	1.48	10.03	10.89
Amide3V2	1.556	135.32	-0.576	1.29	8.98	11.01
Amide430	1.718	143.02	-0.378	1.53	8.95	11.26
Amide4V2	1.567	134.62	-0.570	1.56	13.70	9.87
Amide50	1.612	137.46	-0.502	1.61	15.67	9.34
Amide5V2	1.564	134.16	-0.581	1.04	6.45	11.52
Amide671	1.651	137.54	-0.484	1.68	7.33	11.90
Amide6V2	1.561	135.31	-0.575	0.90	3.58	12.24
Amide7V2	1.566	134.13	-0.574	1.32	10.30	10.65
Amide8V2	1.566	134.65	-0.574	1.49	16.11	9.09
Amine0	1.569	135.12	-0.567	1.75	17.95	8.80
Amine1	1.567	134.77	-0.574	1.34	10.97	10.47
Amine2	1.565	134.69	-0.576	1.40	13.97	9.64
Amine30	1.566	134.91	-0.566	1.39	12.64	10.03
Amine3V2	1.562	134.71	-0.575	1.27	12.15	10.05
Amine430	1.603	135.00	-0.529	1.58	11.47	10.56
Amine4V2	1.561	134.73	-0.575	1.43	12.47	10.11
Amine50	1.579	134.74	-0.559	1.48	8.46	11.36
Amine5V2	1.567	134.68	-0.571	1.56	18.00	8.60
Amine671	1.589	133.66	-0.554	1.73	11.11	10.82
Amine6V2	1.560	134.82	-0.575	1.60	14.73	9.61

Amine7V2	1.566	134.51	-0.573	1.56	14.22	9.72
Amine8V2	1.565	134.44	-0.574	1.23	9.27	10.87
Anhydride0	1.600	137.24	-0.521	1.46	12.05	10.26
Anhydride1	1.574	135.15	-0.558	1.58	12.82	10.16
Anhydride2	1.564	134.50	-0.574	1.52	15.02	9.45
Anhydride30	1.676	141.96	-0.413	1.97	15.80	9.66
Anhydride3V2	1.567	134.75	-0.568	1.36	8.20	11.32
Anhydride430	3.621	179.57	0.000	2.77	23.37	8.21
Anhydride4V2	1.567	134.94	-0.569	1.40	13.88	9.66
Anhydride50	1.600	143.34	-0.495	1.24	3.76	12.52
Anhydride5V2	1.568	135.09	-0.568	1.26	6.36	11.76
Anhydride671	1.623	136.81	-0.497	1.43	3.69	12.73
Anhydride7V2	1.566	134.61	-0.572	1.16	10.65	10.39
Anhydride8V2	1.567	134.70	-0.571	1.32	11.19	10.38
AzolidineBr1	1.600	144.21	-0.490	0.86	2.61	12.49
AzolidineBr2	1.600	134.83	-0.539	1.32	6.34	11.83
AzolidineC#N1	1.600	137.51	-0.500	1.68	17.84	8.76
AzolidineC#N2	1.583	135.21	-0.548	1.65	13.00	10.18
AzolidineCl1	1.600	137.45	-0.497	0.81	4.72	11.81
AzolidineCl2	1.600	135.72	-0.524	1.33	10.43	10.63
AzolidineF1	1.600	137.73	-0.507	1.38	10.44	10.67
AzolidineF2	1.581	135.21	-0.548	1.74	16.49	9.23
AzolidineI1	1.600	136.06	-0.515	0.83	0.65	13.04
AzolidineI2	1.584	135.30	-0.543	1.68	13.00	10.21
AzolidineN1	1.565	134.66	-0.564	1.72	13.48	10.10
AzolidineN2	1.600	135.17	-0.539	1.38	9.63	10.91
AzolidineNO2_1	1.613	138.41	-0.482	1.58	9.50	11.15
AzolidineNO2_2	1.600	136.39	-0.527	1.23	7.31	11.45
AzolidineO1	1.570	135.28	-0.553	1.65	11.96	10.49
AzolidineO2	1.578	135.44	-0.545	1.63	13.33	10.06
AzolidineS1	1.600	143.93	-0.502	1.27	8.01	11.28
AzolidineS2	1.576	135.39	-0.545	1.69	12.86	10.26
Br0	1.600	146.13	-0.518	0.92	7.42	11.12
Br1	1.579	135.75	-0.546	1.28	11.53	10.25
Br2	1.567	134.87	-0.566	1.36	12.35	10.08
Br3	4.112	179.72	-0.001	2.98	27.82	7.09
Br4	1.656	140.41	-0.446	1.07	5.67	11.78

Br5	3.764	179.35	0.000	2.98	33.55	5.38
Br6	1.629	137.21	-0.487	1.99	18.68	8.82
Cl0	1.613	138.19	-0.493	1.19	14.40	9.30
Cl1	1.576	135.38	-0.556	1.31	12.90	9.87
Cl2	1.568	134.93	-0.565	1.37	11.08	10.47
Cl3	4.284	178.83	-0.001	3.13	30.91	6.32
Cl4	1.669	141.57	-0.426	1.39	13.50	9.76
Cl5	3.911	179.26	0.000	2.96	32.52	5.66
Cl6	1.663	139.59	-0.448	2.68	27.77	6.80
Cyan0	1.600	147.43	-0.505	1.42	13.81	9.71
Cyan1	1.600	135.30	-0.540	1.21	10.30	10.55
Cyan2	1.568	134.61	-0.571	1.43	13.00	9.96
Cyan30	1.655	141.04	-0.440	1.72	13.47	10.11
Cyan3V2	1.565	134.56	-0.572	1.39	13.16	9.87
Cyan430	3.414	179.64	0.000	2.81	26.19	7.40
Cyan4V2	1.565	134.61	-0.574	1.49	14.79	9.48
Cyan50	1.616	138.65	-0.490	1.75	14.44	9.85
Cyan5V2	1.566	134.63	-0.572	1.25	9.31	10.87
Cyan671	1.636	137.75	-0.476	1.67	8.72	11.47
Cyan6V2	1.566	134.81	-0.571	1.43	10.93	10.57
Cyan7V2	1.565	134.75	-0.573	1.39	9.95	10.83
Cyan8V2	1.564	134.55	-0.575	1.45	8.44	11.34
Ester0	1.587	136.53	-0.526	1.54	15.69	9.27
Ester1	1.575	135.13	-0.557	1.35	13.83	9.62
Ester2	1.567	134.91	-0.567	1.20	8.60	11.04
Ester30	1.633	139.70	-0.464	1.79	16.26	9.35
Ester3V2	1.569	134.47	-0.573	1.23	10.94	10.37
Ester430	2.874	177.69	-0.003	2.90	27.82	7.01
Ester4V2	1.567	134.61	-0.570	1.52	16.88	8.89
Ester50	1.600	141.69	-0.515	1.27	6.89	11.62
Ester5V2	1.563	134.57	-0.574	1.40	11.19	10.47
Ester671	1.628	136.85	-0.498	1.86	13.50	10.24
Ester6V2	1.567	134.71	-0.571	1.43	9.24	11.08
Ester7V2	1.565	134.46	-0.574	1.43	11.86	10.30
Ester8V2	1.565	134.64	-0.574	1.39	13.23	9.85
Ether0	1.600	135.45	-0.523	1.05	8.18	11.01
Ether1	1.568	135.14	-0.563	1.03	8.04	11.04

Ether2	1.563	134.79	-0.571	1.36	11.63	10.30
Ether30	1.600	136.26	-0.516	1.61	14.65	9.64
Ether3V2	1.566	134.56	-0.571	1.29	5.94	11.92
Ether430	1.610	136.00	-0.503	1.70	13.41	10.10
Ether4V2	1.567	134.57	-0.573	1.50	14.45	9.59
Ether50	1.600	135.85	-0.527	1.39	8.90	11.14
Ether5V2	1.561	134.66	-0.577	1.40	13.79	9.70
Ether671	1.618	136.29	-0.513	1.79	10.55	11.05
Ether6V2	1.564	134.53	-0.576	1.53	15.10	9.43
Ether7V2	1.565	134.53	-0.574	1.27	7.65	11.40
Ether8V2	1.566	134.70	-0.571	1.46	14.80	9.45
F1	1.571	134.90	-0.567	1.25	11.87	10.12
F2	1.574	134.98	-0.560	1.22	10.92	10.37
F3	1.674	142.10	-0.412	1.82	13.84	10.09
F4	1.615	137.65	-0.499	1.69	15.10	9.59
F5	3.141	179.31	-0.001	2.96	34.25	5.15
F6	1.610	135.81	-0.515	1.60	8.67	11.42
Halid2V2	1.571	135.04	-0.563	1.38	13.37	9.80
Halid3V2	1.566	134.63	-0.571	1.54	15.33	9.37
Halid4V2	1.566	134.25	-0.578	1.34	11.00	10.47
Halid5V2	1.565	134.54	-0.574	1.22	10.91	10.37
Halid6V2	1.566	134.56	-0.573	1.36	11.40	10.37
Halid7V2	1.562	134.60	-0.575	1.37	15.42	9.18
Halid8V2	1.565	134.71	-0.573	1.35	11.68	10.27
I0	1.600	146.40	-0.517	0.62	2.43	12.30
I1	1.600	136.53	-0.531	1.07	7.80	11.15
I2	1.566	134.88	-0.567	1.46	13.49	9.84
I3	4.383	178.83	0.000	2.99	34.19	5.20
I4	1.683	142.01	-0.419	1.74	14.49	9.82
I5	5.577	179.88	0.000	2.97	31.69	5.93
I6	1.649	138.56	-0.468	1.51	4.90	12.45
ImidazoleBr1	3.481	178.76	0.000	2.82	28.91	6.61
ImidazoleC#N1	3.457	178.69	0.000	2.86	33.65	5.22
ImidazoleC#N2	3.780	179.50	0.001	0.58	6.67	10.99
ImidazoleCl1	3.201	178.21	0.000	2.75	27.22	7.04
ImidazoleF1	3.581	178.52	0.000	2.85	34.44	4.98
ImidazoleF2	3.481	178.47	0.000	2.82	30.58	6.10

ImidazoleI1	3.494	178.42	0.000	2.86	32.41	5.60
ImidazoleI2	3.436	179.13	0.001	2.67	25.44	7.49
ImidazoleN1	3.347	178.82	0.000	2.54	28.43	6.47
ImidazoleN2	3.660	178.79	0.000	1.94	20.44	8.25
ImidazoleNO2_1	3.119	177.09	0.000	2.67	24.73	7.70
ImidazoleNO2_2	3.208	176.86	0.000	2.88	31.05	6.03
ImidazoleO1	3.483	179.15	0.001	2.53	24.16	7.73
ImidazoleO2	3.377	178.21	0.000	2.88	30.38	6.22
ImidazoleS1	2.954	176.71	0.000	2.50	22.61	8.16
ImidazoleS2	3.849	179.01	0.000	2.88	30.62	6.15
Ketone3V2	1.566	134.72	-0.570	1.46	12.43	10.16
Ketone4V2	1.569	134.52	-0.571	1.29	10.49	10.56
Ketone5V2	1.564	134.15	-0.579	1.15	7.27	11.39
Ketone6V2	1.566	134.65	-0.571	1.29	10.45	10.58
Ketone7V2	1.563	134.36	-0.575	1.13	7.16	11.40
Ketone8V2	1.563	134.45	-0.576	1.47	13.02	9.99
Nitro0	1.580	135.77	-0.544	1.52	12.77	10.12
Nitro1	1.568	134.78	-0.568	1.55	15.73	9.26
Nitro3	1.565	134.30	-0.575	1.48	12.15	10.26
Nitro30	1.620	138.63	-0.492	1.72	13.55	10.08
Nitro430	1.703	142.28	-0.393	2.43	20.25	8.80
Nitro4V2	1.562	134.73	-0.573	1.23	11.03	10.34
Nitro50	1.604	137.17	-0.517	1.68	13.19	10.15
Nitro5V2	1.566	134.62	-0.572	1.56	13.93	9.81
Nitro671	1.601	135.13	-0.531	1.91	16.10	9.52
Nitro6V2	1.567	134.64	-0.570	1.43	11.38	10.44
Nitro7V2	1.565	134.56	-0.574	1.51	13.02	10.03
Nitro8V2	1.566	134.63	-0.572	1.45	12.95	9.99
PiperidineBr1	1.600	135.82	-0.520	1.50	10.11	10.89
PiperidineBr2	1.581	134.94	-0.552	1.75	14.21	9.91
PiperidineBr3	1.600	134.14	-0.558	1.56	11.36	10.58
PiperidineC#N1	1.600	137.72	-0.502	1.52	10.70	10.74
PiperidineC#N2	1.600	136.48	-0.536	1.50	10.64	10.73
PiperidineC#N3	1.584	135.74	-0.543	1.78	14.64	9.82
PiperidineCl1	1.605	136.27	-0.511	1.80	14.10	10.00
PiperidineCl2	1.591	136.33	-0.530	1.78	13.69	10.11
PiperidineCl3	1.579	134.52	-0.560	1.61	12.14	10.40

PiperidineF1	1.600	136.33	-0.519	1.52	10.76	10.72
PiperidineF2	1.582	135.00	-0.555	1.69	12.81	10.27
PiperidineF3	1.600	134.55	-0.543	1.57	11.52	10.54
PiperidineI1	1.606	137.69	-0.505	1.86	14.67	9.89
PiperidineI2	1.591	136.20	-0.532	1.81	14.62	9.86
PiperidineI3	1.577	135.75	-0.544	1.48	10.78	10.67
PiperidineN1	1.581	135.81	-0.542	1.70	12.45	10.39
PiperidineN2	1.580	135.71	-0.548	1.91	17.54	9.08
PiperidineN3	1.576	135.52	-0.553	1.91	19.23	8.58
PiperidineNO2_1	1.615	137.51	-0.495	1.78	13.46	10.16
PiperidineNO2_2	1.600	135.72	-0.535	1.47	8.36	11.38
PiperidineNO2_3	1.577	134.13	-0.562	1.68	11.55	10.64
PiperidineO1	1.600	135.24	-0.541	1.27	9.79	10.75
PiperidineO2	1.584	135.66	-0.545	1.84	15.58	9.60
PiperidineO3	1.582	135.44	-0.547	1.77	15.73	9.48
PiperidineS1	1.600	134.95	-0.535	1.09	6.48	11.56
PiperidineS2	1.600	135.08	-0.546	1.63	12.17	10.40
PiperidineS3	1.578	134.64	-0.564	1.59	13.56	9.95
PyrazineBr1	2.708	177.31	-0.004	2.88	29.47	6.50
PyrazineBr2	1.619	140.01	-0.407	1.97	15.40	9.78
PyrazineC#N1	2.676	177.59	-0.004	2.87	28.44	6.79
PyrazineC#N2	1.654	142.14	-0.368	2.07	15.57	9.83
PyrazineCl1	1.669	141.50	-0.353	2.11	16.50	9.59
PyrazineCl2	1.625	140.40	-0.400	1.98	15.64	9.72
PyrazineF1	1.654	142.03	-0.359	2.08	17.40	9.29
PyrazineF2	1.626	140.44	-0.399	1.98	16.51	9.46
PyrazineI1	2.937	178.15	-0.001	2.85	27.78	6.98
PyrazineI2	1.617	139.78	-0.411	1.97	15.28	9.82
PyrazineN1	1.532	133.27	-0.516	1.50	12.65	10.13
PyrazineN2	1.585	137.21	-0.464	1.77	13.82	10.05
PyrazineNO2_1	3.079	179.29	0.000	2.85	28.42	6.78
PyrazineNO2_2	1.643	142.09	-0.371	2.03	14.97	9.97
PyrazineO1	1.585	136.68	-0.439	1.89	17.75	9.00
PyrazineO2	1.604	138.70	-0.432	1.90	15.46	9.70
PyrazineS1	1.589	137.01	-0.444	1.87	15.76	9.58
PyrazineS2	1.603	138.86	-0.430	1.88	14.55	9.95
PyrazoleBr1	3.540	179.01	0.000	2.82	31.78	5.75

PyrazoleBr2	3.533	179.00	0.000	2.80	31.95	5.68
PyrazoleC#N1	3.541	178.80	0.000	2.89	34.78	4.92
PyrazoleC#N2	3.204	177.77	0.000	2.71	27.96	6.78
PyrazoleCl1	3.541	178.99	0.000	2.82	31.99	5.69
PyrazoleCl2	3.509	178.94	0.000	2.72	28.53	6.61
PyrazoleF1	3.531	178.86	0.000	2.84	32.10	5.67
PyrazoleF2	3.528	179.03	0.000	2.74	29.28	6.41
PyrazoleI1	3.482	178.93	0.000	2.84	30.46	6.16
PyrazoleI2	3.491	178.78	0.000	2.80	31.21	5.90
PyrazoleN1	3.474	179.28	0.000	2.86	33.08	5.40
PyrazoleN2	3.753	179.86	0.000	3.05	34.29	5.23
PyrazoleNO2_1	3.852	178.80	0.000	2.95	35.15	4.87
PyrazoleNO2_2	3.509	178.54	0.000	2.74	28.87	6.53
PyrazoleO1	3.511	179.37	0.000	2.77	28.55	6.66
PyrazoleO2	3.363	179.26	0.000	2.60	27.92	6.68
PyrazoleS1	3.483	178.87	0.000	2.88	32.73	5.52
PyrazoleS2	3.442	179.00	0.000	2.74	29.98	6.21
PyridineBr1	1.585	136.05	-0.461	1.86	13.84	10.13
PyridineBr2	1.565	136.21	-0.484	1.86	15.32	9.70
PyridineBr3	1.561	135.90	-0.490	1.85	14.06	10.06
PyridineC#N1	1.580	137.26	-0.451	1.82	14.41	9.93
PyridineC#N2	1.573	136.86	-0.470	1.82	14.67	9.85
PyridineC#N3	1.567	136.19	-0.484	1.76	13.02	10.28
PyridineCl1	1.570	135.61	-0.468	1.81	13.82	10.10
PyridineCl2	1.567	136.43	-0.479	1.85	14.87	9.82
PyridineCl3	1.564	135.85	-0.487	1.84	14.80	9.83
PyridineF1	1.574	136.31	-0.461	1.87	15.75	9.57
PyridineF2	1.564	136.28	-0.484	1.82	14.34	9.95
PyridineF3	1.557	135.66	-0.496	1.81	14.21	9.98
PyridineI1	1.597	134.27	-0.459	1.86	13.46	10.25
PyridineI2	1.566	136.18	-0.483	1.87	14.79	9.87
PyridineI3	1.560	135.77	-0.491	1.92	17.07	9.24
PyridineN1	1.516	131.45	-0.553	1.83	16.68	9.26
PyridineN2	1.551	134.64	-0.519	1.78	14.56	9.84
PyridineN3	1.509	132.34	-0.573	1.59	12.73	10.19
PyridineNO2_1	1.621	140.68	-0.395	2.01	14.50	10.09
PyridineNO2_2	1.583	137.64	-0.453	1.86	14.26	10.01

PyridineNO2_3	1.578	136.95	-0.469	1.93	17.60	9.08
PyridineO1	1.527	132.55	-0.490	1.54	12.21	10.31
PyridineO2	1.559	135.45	-0.500	1.88	15.40	9.70
PyridineO3	1.535	134.17	-0.529	1.81	15.62	9.55
PyridineS1	1.571	134.79	-0.483	1.82	14.10	10.02
PyridineS2	1.563	135.96	-0.489	1.74	13.67	10.07
PyridineS3	1.548	134.97	-0.510	1.72	13.36	10.14
PyrimidineBr1	1.605	138.41	-0.417	1.88	14.93	9.84
PyrimidineBr2	1.633	141.00	-0.380	2.13	19.10	8.84
PyrimidineBr3	1.588	138.46	-0.436	1.96	17.27	9.21
PyrimidineC#N1	1.615	140.27	-0.392	1.89	15.67	9.63
PyrimidineC#N2	1.619	140.69	-0.388	2.06	17.14	9.35
PyrimidineC#N3	1.584	139.11	-0.432	1.83	16.10	9.43
PyrimidineCl1	1.613	138.52	-0.404	1.92	15.89	9.59
PyrimidineCl2	1.587	138.51	-0.423	1.86	14.16	10.04
PyrimidineCl3	1.590	138.76	-0.431	1.90	16.06	9.51
PyrimidineF1	1.596	139.08	-0.410	1.85	15.40	9.66
PyrimidineF2	1.598	139.13	-0.410	1.88	14.15	10.07
PyrimidineF3	1.593	138.72	-0.431	1.91	17.01	9.25
PyrimidineI1	1.641	141.31	-0.374	2.10	16.89	9.47
PyrimidineI2	1.622	138.24	-0.404	1.97	14.35	10.09
PyrimidineI3	1.586	138.35	-0.438	1.95	16.85	9.33
PyrimidineN1	1.530	133.30	-0.513	1.77	14.95	9.72
PyrimidineN2	1.526	133.03	-0.520	1.55	13.04	10.07
PyrimidineN3	1.566	136.36	-0.480	1.76	13.53	10.13
PyrimidineNO2_1	2.785	178.44	-0.002	3.05	34.28	5.23
PyrimidineNO2_2	2.786	178.54	-0.002	2.83	26.15	7.43
PyrimidineNO2_3	1.626	141.03	-0.387	2.02	16.77	9.42
PyrimidineO1	1.565	135.89	-0.456	1.82	14.23	9.98
PyrimidineO2	1.535	133.97	-0.455	1.58	12.29	10.32
PyrimidineO3	1.574	137.63	-0.458	1.77	17.34	9.01
PyrimidineS1	1.561	135.66	-0.473	1.69	12.76	10.29
PyrimidineS2	1.589	136.48	-0.439	1.94	15.43	9.74
PyrimidineS3	1.586	138.25	-0.439	1.87	14.74	9.88
PyrroleBr1	3.373	179.26	0.001	2.73	29.26	6.41
PyrroleBr2	3.472	179.38	0.000	2.69	26.63	7.15
PyrroleC#N1	3.418	179.27	0.000	2.72	27.31	6.98

PyrroleC#N2	3.006	176.07	0.000	2.76	25.50	7.56
PyrroleCl1	3.291	178.61	0.000	2.86	34.20	5.07
PyrroleCl2	3.512	179.02	0.002	2.70	26.88	7.09
PyrroleF1	3.369	178.93	0.001	2.79	30.61	6.06
PyrroleF2	3.522	178.95	0.002	2.66	25.59	7.43
PyrroleI1	3.404	179.27	0.000	2.70	28.22	6.68
PyrroleI2	3.396	179.98	0.000	0.35	4.91	11.29
PyrroleN1	1.675	141.37	-0.416	2.01	15.35	9.83
PyrroleN2	1.600	137.73	-0.502	1.57	14.51	9.64
PyrroleNO2_1	3.460	178.64	0.000	2.86	33.23	5.36
PyrroleNO2_2	3.466	179.11	0.000	2.81	29.99	6.27
PyrroleO1	1.653	140.33	-0.418	1.62	12.82	10.21
PyrroleO2	1.656	139.97	-0.438	2.01	17.80	9.10
PyrroleS1	3.502	178.90	0.000	2.71	29.48	6.32
PyrroleS2	3.375	178.87	0.001	2.71	27.19	7.01
ThiazoleBr1	1.536	134.43	-0.504	1.83	14.48	9.92
ThiazoleBr2	1.531	135.00	-0.507	1.84	14.45	9.94
ThiazoleBr3	1.538	134.46	-0.501	1.83	15.33	9.66
ThiazoleC#N1	1.530	135.17	-0.503	1.77	13.97	10.01
ThiazoleC#N2	1.535	135.29	-0.501	1.80	14.43	9.90
ThiazoleC#N3	1.540	135.88	-0.485	1.75	13.48	10.13
ThiazoleCl1	1.535	134.42	-0.501	1.78	13.49	10.16
ThiazoleCl2	1.532	135.29	-0.505	1.80	14.26	9.95
ThiazoleCl3	1.536	134.46	-0.497	1.81	15.37	9.63
ThiazoleF1	1.512	135.91	-0.512	1.79	14.33	9.92
ThiazoleF2	1.534	135.15	-0.504	1.81	14.94	9.76
ThiazoleF3	1.525	135.40	-0.501	1.79	14.66	9.82
ThiazoleI1	1.539	133.27	-0.506	1.81	13.72	10.12
ThiazoleI2	1.533	135.09	-0.505	1.84	14.67	9.87
ThiazoleI3	1.562	136.11	-0.467	1.82	13.77	10.12
ThiazoleN1	1.491	131.82	-0.570	1.24	7.25	11.49
ThiazoleN2	1.532	134.29	-0.525	1.74	14.18	9.91
ThiazoleN3	1.505	132.23	-0.550	1.71	12.75	10.31
ThiazoleNO2_1	1.542	136.37	-0.480	1.86	13.93	10.11
ThiazoleNO2_2	1.530	137.01	-0.494	1.75	12.64	10.39
ThiazoleNO2_3	1.552	137.20	-0.460	1.70	12.57	10.35
ThiazoleO1	1.517	133.32	-0.522	1.80	15.78	9.50

ThiazoleO2	1.534	134.88	-0.510	1.77	14.83	9.75
ThiazoleO3	1.520	133.49	-0.519	1.90	16.35	9.42
ThiazoleS1	1.514	132.86	-0.541	1.68	12.66	10.31
ThiazoleS3	1.519	133.12	-0.531	1.63	11.98	10.46
Tio0	1.570	135.09	-0.561	1.41	15.98	9.05
Tio1	1.563	134.85	-0.570	1.28	10.97	10.41
Tio2	1.566	134.31	-0.574	1.58	13.77	9.88
Tio30	1.602	136.94	-0.521	1.39	4.89	12.34
Tio3V2	1.563	134.52	-0.574	1.43	12.60	10.08
Tio430	1.615	135.27	-0.519	1.87	12.32	10.60
Tio4V2	1.564	134.38	-0.576	1.37	12.72	9.98
Tio50	1.600	135.62	-0.534	1.30	11.32	10.33
Tio5V2	1.566	134.54	-0.573	1.48	12.47	10.16
Tio671	1.609	135.39	-0.528	2.41	20.92	8.57
Tio6V2	1.565	134.54	-0.573	1.04	7.36	11.25
Tio7V2	1.566	134.67	-0.572	1.05	6.54	11.50
Tio8V2	1.565	134.53	-0.574	1.25	8.08	11.25
TriazoleBr2	3.745	179.60	0.001	0.59	5.85	11.25
TriazoleC#N1	3.555	178.05	0.000	2.82	31.54	5.82
TriazoleC#N2	3.380	177.43	0.000	2.01	22.30	7.77
TriazoleCl1	3.509	178.58	0.000	2.82	30.90	6.01
TriazoleCl2	3.190	177.49	0.001	1.98	29.12	5.70
TriazoleF1	3.455	178.96	0.001	2.72	28.87	6.52
TriazoleF2	3.550	178.16	0.000	2.14	24.36	7.28
TriazoleI2	3.368	177.83	0.000	2.03	19.98	8.47
TriazoleN1	3.681	179.48	0.001	1.99	18.45	8.90
TriazoleN2	3.228	177.36	0.000	1.55	14.18	9.73
TriazoleNO2_1	3.582	178.54	0.000	2.77	29.32	6.44
TriazoleNO2_2	3.665	178.06	0.000	2.28	28.65	6.14
TriazoleO1	3.478	178.49	0.001	2.90	36.90	4.30
TriazoleO2	1.712	144.59	-0.280	1.51	12.53	10.18
TriazoleS1	3.588	179.43	0.002	1.93	20.00	8.37
TriazoleS2	3.421	177.69	0.000	1.82	17.55	8.99

Table S18: Parameters calculated using the RM1 method.

Name	R (Å)	θ (°)	ΔQ	ΔH (kcal/mol)	ΔS (cal/molK)	ΔG (kcal/mol)

Acid0	1.603	133.72	-0.521	1.45	12.03	10.26
Acid1	1.585	132.31	-0.559	1.37	10.85	10.53
Acid2	1.577	131.66	-0.574	1.37	10.76	10.56
Acid30	1.656	137.23	-0.418	1.42	7.98	11.44
Acid3V2	1.578	131.85	-0.572	1.50	13.75	9.80
Acid430	2.860	179.78	0.000	2.57	21.32	8.61
Acid4V2	1.578	131.76	-0.573	1.25	8.93	10.99
Acid50	1.592	134.47	-0.465	1.46	11.48	10.43
Acid671	2.728	177.22	-0.005	2.79	20.38	9.11
Acid6V2	1.580	132.04	-0.567	1.22	7.37	11.43
Acid7V2	1.577	131.75	-0.574	1.20	12.49	9.88
Acid8V2	1.577	131.64	-0.575	1.52	14.17	9.70
AcidBr1	1.588	132.61	-0.549	1.36	11.04	10.47
AcidBr2	1.584	132.32	-0.561	1.51	12.72	10.12
AcidBr3	2.728	178.43	-0.004	2.73	21.01	8.87
AcidBr4	1.683	138.82	-0.421	1.88	14.60	9.93
AcidBr5	2.849	179.18	-0.002	2.67	19.34	9.31
AcidBr6	2.825	178.19	-0.002	2.64	21.67	8.58
AcidCl0	1.611	134.72	-0.503	1.66	15.00	9.59
AcidCl1	1.579	132.89	-0.554	1.32	11.03	10.43
AcidCl2	1.600	131.70	-0.549	1.45	11.81	10.33
AcidCl3	1.777	144.82	-0.317	2.08	16.76	9.49
AcidCl4	1.669	138.22	-0.438	1.41	10.88	10.56
AcidCl5	3.170	179.63	0.000	3.12	27.40	7.35
AcidCl6	2.861	178.15	0.000	2.54	17.07	9.86
AcidF0	1.591	134.53	-0.523	1.06	5.73	11.75
AcidF1	1.587	132.36	-0.553	1.44	12.32	10.17
AcidF2	1.581	131.95	-0.567	1.55	14.01	9.77
AcidF3	2.677	177.31	-0.007	2.87	27.09	7.20
AcidF4	1.657	137.39	-0.449	1.54	8.57	11.39
AcidF5	2.823	178.70	-0.002	2.72	24.55	7.80
AcidF6	1.683	136.58	-0.436	1.74	10.67	10.97
AcidI0	1.601	134.09	-0.518	1.16	11.61	10.10
AcidI1	1.589	132.71	-0.548	1.38	13.05	9.89
AcidI2	1.580	132.32	-0.565	1.44	11.89	10.30
AcidI3	1.762	142.98	-0.342	1.70	14.14	9.89
AcidI4	1.688	138.69	-0.419	1.69	16.94	9.04

AcidI5	2.950	179.00	-0.001	2.44	19.52	9.02
AcidI6	2.899	177.85	0.000	2.60	19.60	9.16
Acid_Halid3V2	1.574	132.01	-0.574	1.46	15.91	9.12
Acid_Halid4V2	1.578	131.76	-0.573	1.38	14.63	9.42
Acid_Halid5V2	1.578	131.70	-0.574	1.42	16.62	8.86
Acid_Halid6V2	1.569	131.91	-0.580	1.41	12.56	10.07
Acid_Halid7V2	1.577	131.73	-0.574	1.54	16.49	9.03
Acid_Halid8V2	1.577	131.80	-0.573	1.36	10.52	10.63
Acid_Sulf0	1.593	132.61	-0.546	1.41	12.58	10.06
Acid_Sulf1	1.579	131.87	-0.571	1.49	14.22	9.66
Acid_Sulf2	1.579	131.99	-0.569	1.18	9.04	10.88
Acid_Sulf30	1.641	134.94	-0.481	1.96	15.31	9.79
Acid_Sulf3V2	1.568	132.09	-0.578	1.30	11.72	10.20
Acid_Sulf430	3.542	179.94	0.001	3.25	36.74	4.70
Acid_Sulf4V2	1.577	131.79	-0.573	1.56	13.75	9.86
Acid_Sulf50	1.600	133.58	-0.530	1.00	5.60	11.74
Acid_Sulf5V2	1.577	131.68	-0.575	1.29	15.01	9.22
Acid_Sulf671	1.680	135.94	-0.452	1.52	5.73	12.21
Acid_Sulf6V2	1.578	131.89	-0.573	1.35	12.38	10.06
Acid_Sulf7V2	1.577	131.88	-0.573	1.26	12.60	9.90
Acid_Sulf8V2	1.578	131.96	-0.572	1.32	9.87	10.78
Acid_Tio0	1.600	133.60	-0.526	1.46	13.04	9.98
Acid_Tio1	1.585	132.37	-0.557	1.47	12.42	10.17
Acid_Tio2	1.577	131.70	-0.576	1.32	16.59	8.78
Acid_Tio30	1.671	137.79	-0.437	1.63	6.12	12.21
Acid_Tio3V2	1.578	131.83	-0.571	1.72	18.23	8.69
Acid_Tio430	2.906	178.73	-0.001	2.73	21.01	8.87
Acid_Tio4V2	1.578	131.77	-0.573	1.42	11.60	10.37
Acid_Tio50	1.657	137.12	-0.453	1.83	12.39	10.54
Acid_Tio5V2	1.578	131.64	-0.574	1.43	18.58	8.29
Acid_Tio671	3.416	179.24	0.002	3.01	28.45	6.93
Acid_Tio6V2	1.579	131.77	-0.572	1.51	16.67	8.94
Acid_Tio7V2	1.577	131.75	-0.574	1.02	8.68	10.84
Acid_Tio8V2	1.577	131.84	-0.574	1.33	8.28	11.27
Alcohol0	1.576	132.03	-0.570	1.52	18.51	8.41
Alcohol1	1.572	132.18	-0.574	1.28	12.04	10.09
Alcohol2	1.577	131.75	-0.574	1.28	11.26	10.33

Alcohol30	1.594	132.50	-0.552	1.32	7.86	11.38
Alcohol3V2	1.577	131.74	-0.574	1.31	14.16	9.50
Alcohol430	1.649	133.56	-0.492	1.76	10.59	11.01
Alcohol4V2	1.578	131.80	-0.572	1.39	16.34	8.92
Alcohol50	1.610	133.43	-0.529	1.53	10.96	10.66
Alcohol5V2	1.578	131.73	-0.574	1.40	14.98	9.34
Alcohol671	1.621	131.73	-0.528	1.02	-0.80	13.67
Alcohol6V2	1.571	131.86	-0.577	1.47	14.24	9.63
Alcohol7V2	1.577	131.79	-0.574	1.46	13.69	9.78
Alcohol8V2	1.577	131.83	-0.574	1.43	12.50	10.11
Aldehyde0	1.585	132.71	-0.550	1.02	7.13	11.30
Aldehyde1	1.582	132.09	-0.566	1.18	6.66	11.60
Aldehyde2	1.579	131.85	-0.570	1.51	13.48	9.90
Aldehyde30	1.620	134.88	-0.506	1.48	8.09	11.47
Aldehyde430	1.706	137.97	-0.419	1.51	5.20	12.36
Aldehyde50	1.614	134.01	-0.520	1.33	10.16	10.70
Aldehyde671	1.648	134.16	-0.493	1.73	11.24	10.78
Amide0	1.583	132.31	-0.539	1.13	9.03	10.84
Amide1	1.579	131.95	-0.571	1.43	15.32	9.27
Amide2	1.581	132.31	-0.565	1.37	14.39	9.48
Amide30	1.683	138.51	-0.425	1.64	10.29	10.98
Amide3V2	1.575	131.84	-0.574	1.48	14.81	9.47
Amide430	2.788	175.18	-0.018	2.61	23.66	7.95
Amide4V2	1.577	131.77	-0.573	1.46	14.10	9.66
Amide50	1.620	134.35	-0.500	1.44	7.76	11.53
Amide5V2	1.574	131.57	-0.582	1.21	13.48	9.59
Amide671	2.223	162.98	-0.068	2.03	5.73	12.73
Amide6V2	1.579	131.85	-0.571	1.14	9.39	10.74
Amide7V2	1.577	131.78	-0.574	1.50	16.09	9.11
Amide8V2	1.577	131.77	-0.574	1.47	10.89	10.63
Amine0	1.572	132.32	-0.578	1.52	16.42	9.02
Amine1	1.575	131.66	-0.580	1.13	10.51	10.40
Amine2	1.571	131.96	-0.580	1.33	13.56	9.69
Amine30	1.578	132.31	-0.572	1.42	12.99	9.95
Amine3V2	1.576	131.86	-0.574	1.15	6.48	11.62
Amine430	1.624	132.30	-0.520	1.51	7.89	11.56
Amine4V2	1.577	131.67	-0.575	1.49	16.88	8.86

Amine50	1.588	132.41	-0.561	1.62	13.58	9.97
Amine5V2	1.577	131.76	-0.574	1.39	13.16	9.87
Amine671	1.620	131.31	-0.530	1.12	3.70	12.42
Amine6V2	1.577	131.73	-0.574	1.55	15.79	9.25
Amine7V2	1.576	131.84	-0.575	1.38	13.28	9.82
Amine8V2	1.577	131.83	-0.574	1.52	14.48	9.61
Anhydride0	1.600	133.61	-0.526	1.08	8.01	11.10
Anhydride1	1.577	132.63	-0.562	1.33	9.18	10.99
Anhydride2	1.572	132.20	-0.572	1.71	17.43	8.91
Anhydride30	1.731	141.57	-0.368	1.77	14.32	9.90
Anhydride3V2	1.580	131.98	-0.567	1.32	11.46	10.30
Anhydride430	2.857	178.19	-0.003	2.62	23.76	7.94
Anhydride4V2	1.577	131.81	-0.573	1.42	12.93	9.97
Anhydride50	1.640	135.25	-0.478	1.64	12.50	10.32
Anhydride5V2	1.576	131.72	-0.575	1.27	10.24	10.62
Anhydride671	2.862	177.86	-0.001	2.79	28.51	6.70
Anhydride7V2	1.577	131.76	-0.574	1.15	11.36	10.17
Anhydride8V2	1.577	131.88	-0.573	1.32	10.56	10.57
AzolidineBr1	1.647	135.39	-0.474	1.38	11.35	10.40
AzolidineBr2	1.600	133.27	-0.536	1.15	6.83	11.51
AzolidineC#N1	1.615	134.47	-0.499	1.63	13.16	10.11
AzolidineC#N2	1.600	132.48	-0.543	1.25	9.75	10.75
AzolidineCl1	1.635	135.94	-0.476	1.42	11.37	10.44
AzolidineCl2	1.607	133.55	-0.526	1.55	12.58	10.20
AzolidineF1	1.600	133.22	-0.524	1.23	7.97	11.26
AzolidineF2	1.600	133.15	-0.536	1.17	7.68	11.28
AzolidineI1	1.627	134.39	-0.500	1.27	6.69	11.68
AzolidineI2	1.600	133.10	-0.537	1.19	7.11	11.48
AzolidineN1	1.569	131.12	-0.589	1.58	8.02	11.59
AzolidineN2	1.600	132.68	-0.550	1.41	12.83	9.99
AzolidineNO2_1	1.663	136.66	-0.453	0.34	-2.70	13.54
AzolidineNO2_2	1.613	134.32	-0.516	1.66	13.44	10.05
AzolidineO1	1.580	132.40	-0.557	1.45	13.15	9.93
AzolidineO2	1.600	133.75	-0.528	1.00	5.47	11.77
AzolidineS1	1.618	133.56	-0.517	1.22	10.84	10.39
AzolidineS2	1.600	133.17	-0.523	0.97	5.45	11.75
Br0	1.624	135.47	-0.496	1.46	13.11	9.96

Br1	1.587	132.76	-0.548	1.32	13.15	9.80
Br2	1.582	132.11	-0.564	1.51	14.78	9.51
Br3	4.829	177.35	-0.003	2.88	30.20	6.27
Br4	1.688	138.74	-0.419	1.59	11.55	10.55
Br5	4.921	179.75	0.000	3.03	36.64	4.51
Br6	1.679	136.70	-0.440	1.56	2.79	13.13
Cl0	1.626	135.67	-0.490	1.58	16.32	9.12
Cl1	1.584	132.37	-0.561	1.22	12.77	9.82
Cl2	1.581	132.13	-0.565	1.36	11.61	10.30
Cl3	4.410	175.78	-0.008	2.87	28.14	6.88
Cl4	1.729	141.26	-0.376	1.72	12.84	10.30
Cl5	4.088	176.40	-0.007	3.08	39.36	3.75
Cl6	2.831	178.21	-0.002	2.79	28.26	6.77
Cyan0	1.600	134.43	-0.522	1.05	10.06	10.46
Cyan1	1.580	131.95	-0.565	1.41	13.30	9.85
Cyan2	1.580	131.78	-0.571	1.25	11.02	10.37
Cyan30	1.662	137.68	-0.446	1.71	15.20	9.58
Cyan3V2	1.578	131.81	-0.571	1.24	11.82	10.11
Cyan430	2.776	178.64	-0.003	2.50	19.82	9.00
Cyan4V2	1.578	131.73	-0.573	1.41	14.23	9.57
Cyan50	1.628	135.65	-0.494	1.49	9.90	10.95
Cyan5V2	1.577	131.69	-0.574	1.35	13.57	9.71
Cyan671	1.661	134.94	-0.461	1.74	17.54	8.92
Cyan6V2	1.576	131.75	-0.575	1.48	14.12	9.67
Cyan7V2	1.578	131.84	-0.573	1.36	13.39	9.77
Cyan8V2	1.578	131.83	-0.574	1.45	13.81	9.73
Ester0	1.600	133.70	-0.524	1.50	13.87	9.77
Ester1	1.585	132.31	-0.557	1.56	14.01	9.79
Ester2	1.583	132.13	-0.564	1.26	8.46	11.14
Ester30	1.669	137.68	-0.442	1.66	12.09	10.46
Ester3V2	1.569	131.99	-0.579	1.21	8.28	11.15
Ester430	2.754	177.96	-0.004	2.51	19.38	9.13
Ester4V2	1.579	131.79	-0.572	1.57	15.00	9.50
Ester50	1.614	132.93	-0.528	1.55	10.62	10.79
Ester5V2	1.574	131.82	-0.576	1.30	9.91	10.75
Ester671	2.708	176.86	-0.006	2.52	16.96	9.87
Ester6V2	1.578	131.82	-0.574	1.56	15.89	9.22

Ester7V2	1.577	131.82	-0.574	1.50	13.67	9.83
Ester8V2	1.577	131.85	-0.574	1.57	18.46	8.47
Ether0	1.600	132.97	-0.537	1.38	12.95	9.93
Ether1	1.579	131.94	-0.570	1.37	13.41	9.78
Ether2	1.567	132.01	-0.581	1.26	13.03	9.78
Ether30	1.613	133.77	-0.519	1.52	11.07	10.63
Ether3V2	1.578	131.66	-0.574	1.50	14.59	9.55
Ether430	1.655	134.97	-0.469	1.65	7.98	11.67
Ether4V2	1.578	131.72	-0.574	1.20	11.15	10.28
Ether50	1.608	132.99	-0.536	1.55	13.83	9.82
Ether5V2	1.578	131.74	-0.574	1.49	15.61	9.23
Ether671	1.649	134.36	-0.487	1.62	7.54	11.77
Ether6V2	1.572	131.80	-0.578	1.35	15.17	9.23
Ether7V2	1.577	131.87	-0.574	1.62	15.72	9.34
Ether8V2	1.606	134.04	-0.547	1.55	12.33	10.28
F0	1.587	133.27	-0.539	1.35	13.58	9.71
F1	1.581	132.05	-0.569	1.10	10.55	10.36
F2	1.584	132.17	-0.563	1.39	13.83	9.67
F3	1.651	136.95	-0.451	1.72	13.91	9.97
F4	1.626	134.61	-0.497	1.65	15.98	9.29
F5	2.739	178.27	-0.004	2.77	26.59	7.25
F6	1.638	133.86	-0.492	1.57	10.53	10.83
Halid2V2	1.564	132.10	-0.579	1.17	10.02	10.59
Halid3V2	1.579	131.78	-0.572	1.30	11.20	10.36
Halid4V2	1.572	131.39	-0.585	1.32	12.77	9.91
Halid5V2	1.574	131.75	-0.577	1.38	12.88	9.95
Halid6V2	1.577	131.75	-0.574	1.31	13.33	9.74
Halid7V2	1.577	131.74	-0.574	1.52	14.10	9.72
Halid8V2	1.578	131.80	-0.574	1.52	14.46	9.61
I0	1.614	134.61	-0.513	1.21	11.78	10.10
I1	1.588	132.69	-0.548	0.99	8.36	10.90
I2	1.581	132.06	-0.565	1.42	14.14	9.61
I3	3.968	178.04	0.000	3.05	30.51	6.36
I4	1.677	138.00	-0.437	1.59	11.29	10.63
I5	8.689	179.94	0.000	2.97	32.75	5.61
I6	1.698	137.41	-0.427	1.64	6.43	12.12
ImidazoleBr1	3.899	179.58	0.000	2.92	34.05	5.17

ImidazoleC#N1	3.778	179.32	0.000	2.79	28.92	6.57
ImidazoleC#N2	4.523	179.65	0.000	2.98	35.48	4.80
ImidazoleF1	3.858	179.46	0.000	2.93	33.65	5.30
ImidazoleF2	4.330	179.64	0.000	2.97	36.01	4.63
ImidazoleI1	4.482	179.63	0.000	2.83	30.32	6.20
ImidazoleN1	3.258	179.92	0.000	2.52	23.71	7.85
ImidazoleN2	3.786	179.63	0.000	2.86	31.24	5.95
ImidazoleNO2_1	4.076	179.36	0.000	2.87	29.79	6.39
ImidazoleNO2_2	3.753	179.60	0.000	3.00	34.16	5.22
ImidazoleO1	5.945	179.92	0.000	2.97	37.57	4.18
ImidazoleO2	4.501	179.77	0.000	2.93	34.85	4.95
ImidazoleS1	4.340	179.56	0.000	2.93	35.80	4.66
ImidazoleS2	4.159	179.67	0.000	2.94	31.80	5.86
Ketone3V2	1.577	131.78	-0.573	1.41	12.43	10.11
Ketone4V2	1.576	131.77	-0.574	1.33	11.60	10.28
Ketone5V2	1.575	131.28	-0.584	1.31	13.02	9.83
Ketone6V2	1.578	131.86	-0.572	1.17	12.85	9.74
Ketone7V2	1.575	131.72	-0.576	1.39	12.41	10.09
Ketone8V2	1.577	131.68	-0.575	1.39	12.07	10.19
Nitro0	1.600	133.84	-0.529	1.08	9.66	10.61
Nitro1	1.585	132.31	-0.558	1.48	12.99	10.01
Nitro3	1.573	131.65	-0.579	1.52	17.50	8.70
Nitro30	1.628	136.12	-0.490	1.31	2.13	13.08
Nitro430	3.657	179.83	0.001	2.99	26.66	7.45
Nitro4V2	1.569	131.92	-0.580	1.19	11.29	10.23
Nitro50	1.621	134.81	-0.508	1.50	9.45	11.09
Nitro5V2	1.568	131.94	-0.581	1.26	10.23	10.61
Nitro671	1.635	133.66	-0.499	1.53	11.30	10.57
Nitro6V2	1.577	131.80	-0.574	1.50	14.24	9.66
Nitro7V2	1.577	131.78	-0.575	1.49	15.35	9.32
Nitro8V2	1.577	131.69	-0.575	1.48	15.79	9.18
PiperidineBr1	1.627	134.18	-0.496	1.68	12.75	10.29
PiperidineBr2	1.600	133.10	-0.540	1.59	10.91	10.74
PiperidineBr3	1.600	132.90	-0.545	1.57	10.95	10.71
PiperidineC#N1	1.622	135.10	-0.500	1.71	13.11	10.20
PiperidineC#N2	1.600	132.87	-0.542	1.61	11.12	10.69
PiperidineC#N3	1.600	133.48	-0.538	1.42	10.19	10.78

PiperidineCl1	1.637	134.56	-0.487	1.65	11.95	10.49
PiperidineCl2	1.614	134.07	-0.515	1.72	13.06	10.22
PiperidineCl3	1.600	132.78	-0.545	1.63	11.56	10.59
PiperidineF1	1.600	134.63	-0.526	1.34	9.13	11.03
PiperidineF2	1.600	133.07	-0.543	1.65	11.89	10.51
PiperidineF3	1.600	132.91	-0.542	1.45	10.57	10.70
PiperidineI1	1.636	135.52	-0.485	1.80	14.34	9.93
PiperidineI2	1.600	133.64	-0.528	1.48	10.69	10.70
PiperidineI3	1.600	133.34	-0.534	1.42	10.01	10.84
PiperidineN1	1.600	132.38	-0.537	1.33	9.19	10.99
PiperidineN2	1.600	132.90	-0.546	1.39	9.99	10.81
PiperidineN3	1.589	132.81	-0.552	1.70	14.47	9.79
PiperidineNO2_1	1.628	134.20	-0.496	1.53	9.99	10.96
PiperidineNO2_2	1.608	133.53	-0.526	1.78	15.32	9.61
PiperidineNO2_3	1.600	139.18	-0.529	1.49	11.40	10.50
PiperidineO1	1.600	132.80	-0.532	1.63	12.00	10.45
PiperidineO2	1.600	133.22	-0.537	1.32	9.50	10.89
PiperidineO3	1.600	133.17	-0.537	1.35	10.17	10.72
PiperidineS1	1.607	132.28	-0.537	1.53	11.26	10.58
PiperidineS2	1.600	132.62	-0.545	1.47	9.65	10.99
PiperidineS3	1.600	132.88	-0.545	1.50	10.27	10.85
PyrazineBr1	1.652	136.20	-0.392	1.98	14.81	9.97
PyrazineBr2	1.617	135.63	-0.430	1.85	14.24	10.01
PyrazineC#N1	2.706	178.21	-0.003	2.74	25.24	7.61
PyrazineC#N2	1.658	137.81	-0.386	2.00	17.02	9.33
PyrazineCl1	2.730	177.79	-0.003	2.77	26.73	7.20
PyrazineCl2	1.626	136.04	-0.421	1.87	14.77	9.86
PyrazineF1	1.632	137.24	-0.402	1.93	16.23	9.49
PyrazineF2	1.652	137.33	-0.394	1.93	15.41	9.74
PyrazineI1	1.628	137.64	-0.412	1.89	13.36	10.31
PyrazineI2	1.615	136.45	-0.431	1.93	14.91	9.89
PyrazineN1	1.530	129.24	-0.543	1.51	12.61	10.15
PyrazineN2	1.589	134.54	-0.475	1.85	18.30	8.79
PyrazineNO2_1	2.759	178.71	-0.002	2.87	29.03	6.62
PyrazineNO2_2	2.714	177.99	-0.003	2.80	29.26	6.48
PyrazineO1	1.587	133.65	-0.461	1.79	14.47	9.88
PyrazineO2	1.619	135.14	-0.438	1.85	14.78	9.84

PyrazineS1	1.629	134.57	-0.423	1.95	15.28	9.80
PyrazineS2	1.613	135.04	-0.442	1.84	14.54	9.91
PyrazoleBr1	4.831	179.66	0.000	3.00	40.64	3.28
PyrazoleBr2	3.430	179.55	0.000	2.96	33.19	5.46
PyrazoleC#N1	4.675	179.65	0.000	2.96	36.51	4.48
PyrazoleC#N2	4.704	179.69	0.000	2.92	34.33	5.09
PyrazoleCl1	4.771	179.66	0.000	2.96	37.44	4.20
PyrazoleCl2	3.298	179.69	0.000	2.93	33.30	5.41
PyrazoleF1	3.285	179.49	0.000	2.91	33.29	5.38
PyrazoleF2	4.135	179.71	0.000	2.88	33.56	5.28
PyrazoleI1	5.131	179.69	0.000	2.92	32.93	5.50
PyrazoleI2	3.415	179.61	0.000	2.92	31.58	5.91
PyrazoleN1	3.530	179.90	0.000	3.14	39.28	3.83
PyrazoleN2	3.333	179.76	0.000	2.98	36.90	4.38
PyrazoleNO2_1	4.965	179.68	0.000	2.97	37.15	4.29
PyrazoleNO2_2	4.793	179.72	0.000	2.77	29.46	6.39
PyrazoleO1	4.682	179.70	0.000	2.83	30.89	6.03
PyrazoleO2	3.541	179.60	0.000	2.92	32.43	5.66
PyrazoleS1	4.115	179.55	0.000	2.99	34.92	4.98
PyrazoleS2	3.074	179.71	0.000	2.81	27.86	6.91
PyridineBr1	1.599	132.14	-0.469	1.84	13.26	10.29
PyridineBr2	1.575	132.36	-0.500	1.84	17.75	8.95
PyridineBr3	1.576	132.12	-0.502	1.78	14.03	10.01
PyridineC#N1	1.590	133.30	-0.469	1.81	14.30	9.95
PyridineC#N2	1.580	132.84	-0.490	1.82	14.32	9.96
PyridineC#N3	1.578	133.68	-0.489	1.85	14.15	10.04
PyridineCl1	1.591	132.63	-0.472	1.83	13.76	10.13
PyridineCl2	1.580	132.65	-0.492	1.81	14.91	9.77
PyridineCl3	1.576	132.63	-0.499	1.74	13.38	10.15
PyridineF1	1.574	133.19	-0.487	1.81	14.77	9.81
PyridineF2	1.583	132.72	-0.489	1.79	14.26	9.94
PyridineF3	1.567	131.78	-0.512	1.75	13.89	10.01
PyridineI1	1.580	131.70	-0.497	1.72	12.43	10.41
PyridineI2	1.571	132.02	-0.508	1.83	14.39	9.94
PyridineI3	1.574	132.07	-0.506	1.80	13.54	10.16
PyridineN1	1.517	127.77	-0.575	1.52	14.42	9.62
PyridineN2	1.564	130.95	-0.532	1.60	12.59	10.25

PyridineN3	1.521	128.69	-0.596	1.60	12.96	10.14
PyridineNO2_1	1.627	137.93	-0.407	1.92	14.40	10.03
PyridineNO2_2	1.602	134.16	-0.456	1.89	15.70	9.61
PyridineNO2_3	1.602	134.02	-0.460	1.85	15.72	9.56
PyridineO1	1.519	129.40	-0.508	1.50	11.50	10.47
PyridineO2	1.569	132.99	-0.506	1.72	12.98	10.25
PyridineO3	1.549	130.50	-0.545	1.66	13.72	9.97
PyridineS1	1.587	134.97	-0.473	2.02	17.13	9.32
PyridineS2	1.569	131.97	-0.512	1.79	15.68	9.52
PyridineS3	1.561	131.30	-0.525	1.71	13.62	10.05
PyrimidineBr1	1.592	133.17	-0.457	1.84	15.17	9.72
PyrimidineBr2	1.620	137.63	-0.410	2.04	15.50	9.83
PyrimidineBr3	1.589	134.03	-0.465	1.85	16.17	9.43
PyrimidineC#N1	1.611	135.37	-0.427	1.84	16.09	9.45
PyrimidineC#N2	1.603	135.17	-0.435	1.91	15.56	9.68
PyrimidineC#N3	1.597	134.65	-0.452	1.88	16.00	9.51
PyrimidineCl1	1.616	134.78	-0.428	1.86	15.46	9.66
PyrimidineCl2	1.623	137.90	-0.405	2.05	16.45	9.55
PyrimidineCl3	1.594	134.37	-0.458	1.85	16.56	9.31
PyrimidineF1	1.595	135.38	-0.441	1.88	17.12	9.18
PyrimidineF2	1.582	134.61	-0.459	1.80	13.66	10.13
PyrimidineF3	1.600	134.61	-0.451	1.85	16.14	9.44
PyrimidineI1	1.587	132.99	-0.475	1.77	14.15	9.95
PyrimidineI2	1.587	135.21	-0.459	1.82	13.32	10.25
PyrimidineI3	1.578	133.48	-0.480	1.85	16.01	9.48
PyrimidineN1	1.523	128.87	-0.549	1.57	14.79	9.56
PyrimidineN2	1.521	128.86	-0.554	1.57	13.68	9.90
PyrimidineN3	1.573	132.19	-0.503	1.73	16.75	9.13
PyrimidineNO2_1	2.712	178.58	-0.003	2.83	29.68	6.38
PyrimidineNO2_2	2.736	177.98	-0.004	2.67	24.33	7.82
PyrimidineNO2_3	1.628	136.60	-0.410	2.06	18.64	8.90
PyrimidineO1	1.563	132.60	-0.490	1.83	14.78	9.83
PyrimidineO2	1.523	130.93	-0.488	1.53	11.29	10.57
PyrimidineO3	1.585	133.43	-0.477	1.81	17.54	8.98
PyrimidineS1	1.582	132.19	-0.477	1.76	13.67	10.08
PyrimidineS2	1.570	131.13	-0.470	1.68	13.44	10.08
PyrimidineS3	1.583	133.61	-0.477	1.88	15.32	9.71

PyrroleBr1	3.532	179.69	0.000	2.91	28.95	6.68
PyrroleBr2	3.842	179.76	0.000	2.91	30.88	6.10
PyrroleC#N1	4.178	179.69	0.000	2.94	34.62	5.02
PyrroleC#N2	3.066	179.85	0.000	2.95	32.78	5.57
PyrroleCl1	3.426	179.66	0.000	2.84	30.28	6.22
PyrroleCl2	4.409	179.77	0.000	2.95	35.45	4.78
PyrroleF1	4.446	179.77	0.000	3.03	39.15	3.76
PyrroleF2	4.195	179.81	0.000	2.92	33.51	5.34
PyrroleI1	3.771	179.38	0.001	2.52	22.37	8.25
PyrroleI2	3.570	179.78	0.000	2.97	34.51	5.09
PyrroleN1	1.651	136.56	-0.453	1.89	17.25	9.15
PyrroleN2	1.613	133.87	-0.515	1.77	14.34	9.89
PyrroleNO2_1	5.391	179.72	0.000	2.91	34.60	5.00
PyrroleNO2_2	5.026	179.78	0.000	2.95	32.54	5.65
PyrroleO1	1.640	137.30	-0.442	1.87	15.74	9.58
PyrroleO2	1.662	137.00	-0.444	1.71	14.23	9.87
PyrroleS1	4.035	179.74	0.000	2.79	30.34	6.15
PyrroleS2	4.173	179.73	0.000	2.75	31.21	5.85
ThiazoleBr1	1.544	132.82	-0.507	1.78	13.83	10.06
ThiazoleBr3	1.544	131.27	-0.513	1.65	11.74	10.56
ThiazoleC#N1	1.536	131.86	-0.518	1.72	16.96	9.06
ThiazoleC#N2	1.537	132.13	-0.519	1.69	13.36	10.11
ThiazoleC#N3	1.545	132.70	-0.499	1.75	14.29	9.90
ThiazoleCl1	1.539	131.49	-0.519	1.76	13.78	10.05
ThiazoleCl2	1.535	131.92	-0.525	1.73	13.94	9.97
ThiazoleCl3	1.542	131.64	-0.512	1.77	16.15	9.36
ThiazoleF1	1.537	132.53	-0.513	1.78	14.91	9.74
ThiazoleF2	1.546	132.58	-0.506	1.76	15.52	9.53
ThiazoleF3	1.530	132.36	-0.518	1.77	14.59	9.82
ThiazoleI1	1.528	131.05	-0.541	1.69	13.68	10.01
ThiazoleI2	1.524	131.26	-0.545	1.74	14.83	9.72
ThiazoleI3	1.537	131.85	-0.525	1.68	12.44	10.37
ThiazoleN1	1.497	128.64	-0.593	1.60	12.72	10.21
ThiazoleN2	1.540	131.45	-0.533	1.66	13.20	10.12
ThiazoleN3	1.496	128.56	-0.592	1.53	12.48	10.22
ThiazoleNO2_1	1.544	132.91	-0.498	1.83	15.15	9.72
ThiazoleNO2_2	1.546	132.97	-0.498	1.77	14.87	9.74

ThiazoleNO2_3	1.559	134.34	-0.468	1.92	15.07	9.83
ThiazoleO1	1.525	131.07	-0.537	1.65	14.62	9.70
ThiazoleO2	1.543	132.14	-0.517	1.70	15.53	9.47
ThiazoleO3	1.516	130.79	-0.545	1.62	13.58	9.98
ThiazoleS1	1.533	130.50	-0.535	1.64	13.98	9.87
ThiazoleS2	1.530	131.42	-0.538	1.67	13.65	10.01
ThiazoleS3	1.536	130.76	-0.528	1.70	14.37	9.82
Tio0	1.584	132.32	-0.557	1.32	12.74	9.92
Tio1	1.580	131.89	-0.569	1.44	13.53	9.80
Tio2	1.579	131.86	-0.571	1.49	14.60	9.54
Tio30	1.620	134.60	-0.511	1.54	10.87	10.71
Tio3V2	1.577	131.84	-0.573	1.43	13.29	9.87
Tio430	1.671	134.81	-0.464	1.75	12.98	10.28
Tio4V2	1.578	131.68	-0.574	1.38	14.79	9.38
Tio50	1.615	133.92	-0.518	1.46	10.81	10.64
Tio5V2	1.578	131.68	-0.574	1.48	16.98	8.83
Tio671	1.638	133.31	-0.505	1.45	11.85	10.32
Tio6V2	1.572	131.95	-0.577	1.41	12.68	10.03
Tio7V2	1.577	131.76	-0.574	1.54	16.06	9.16
Tio8V2	1.576	131.69	-0.575	1.62	16.68	9.05
TriazoleBr2	3.935	179.32	0.000	1.89	21.60	7.85
TriazoleC#N1	4.611	179.71	0.000	2.97	34.35	5.13
TriazoleC#N2	3.083	178.42	0.001	1.90	17.08	9.22
TriazoleCl2	4.067	179.29	0.000	1.94	24.83	6.94
TriazoleF1	4.547	179.69	0.000	2.94	35.27	4.83
TriazoleF2	4.111	179.33	0.000	2.16	25.84	6.86
TriazoleI2	4.380	179.45	0.000	1.96	24.43	7.08
TriazoleN1	3.600	179.40	0.000	2.90	35.47	4.72
TriazoleN2	3.883	179.43	0.000	2.50	27.28	6.77
TriazoleNO2_1	4.792	179.75	0.000	2.93	32.52	5.64
TriazoleNO2_2	3.862	179.16	0.000	2.15	26.32	6.71
TriazoleO1	4.580	179.74	0.000	2.90	34.05	5.15
TriazoleO2	4.604	179.83	0.000	3.02	36.70	4.48
TriazoleS1	4.594	179.67	0.000	2.85	32.51	5.57
TriazoleS2	4.575	179.51	0.000	2.12	26.18	6.72

S3. Population distribution analysis and histograms of geometric, thermodynamic, and charge-transfer data calculated using the DFT method and the semiempirical methods.

S3. 1 Population distribution analysis and histograms of geometric, thermodynamic, and charge-transfer data calculated using DFT

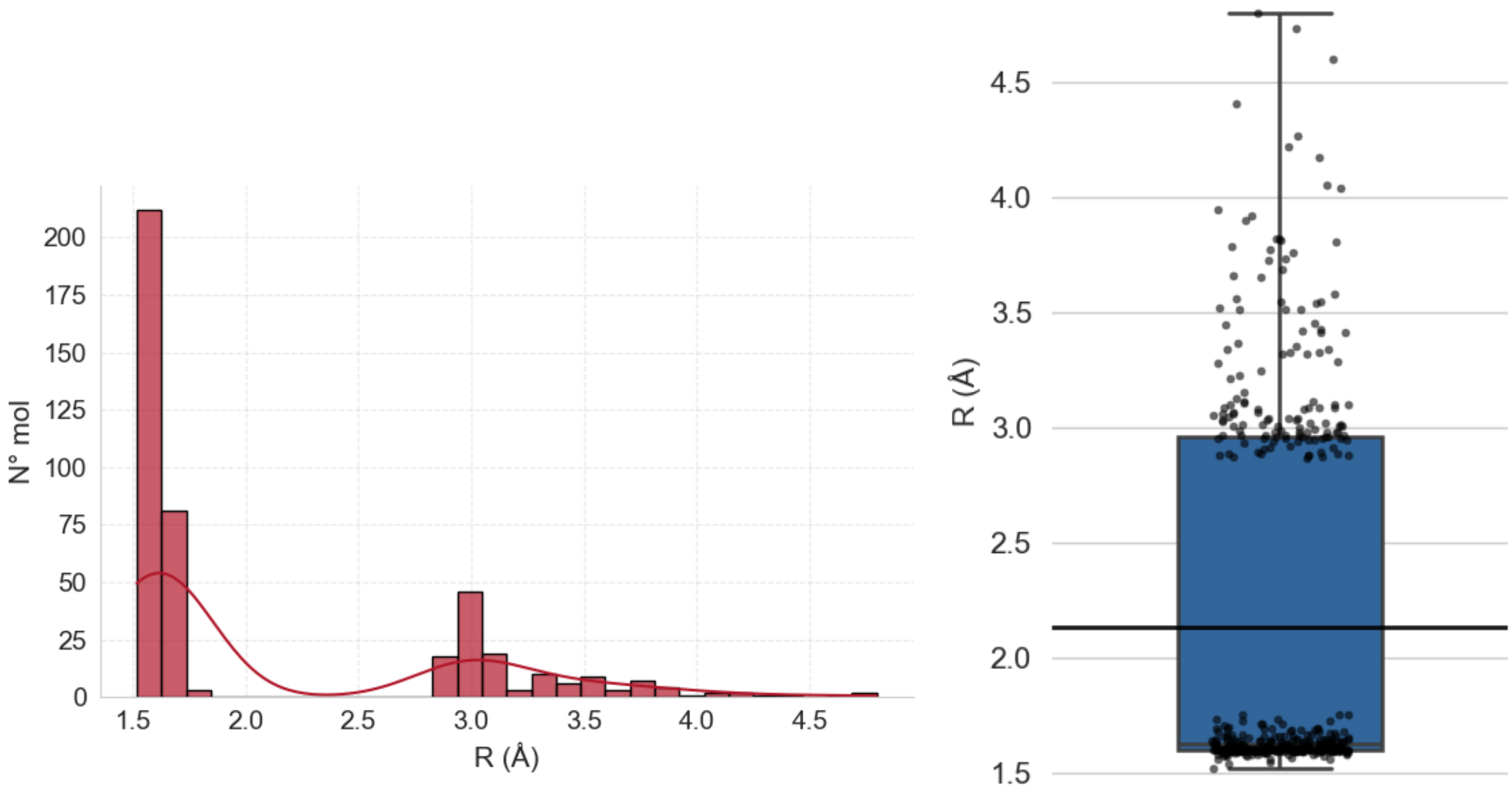

Figure S1: Statistical distribution of the length R (Å) in the analyzed molecular dataset computed at the CAM-B3LYP/6-311++G(d,p) theory level. On the left, a histogram with its corresponding density curve; on the right, a boxplot with superimposed individual data points.

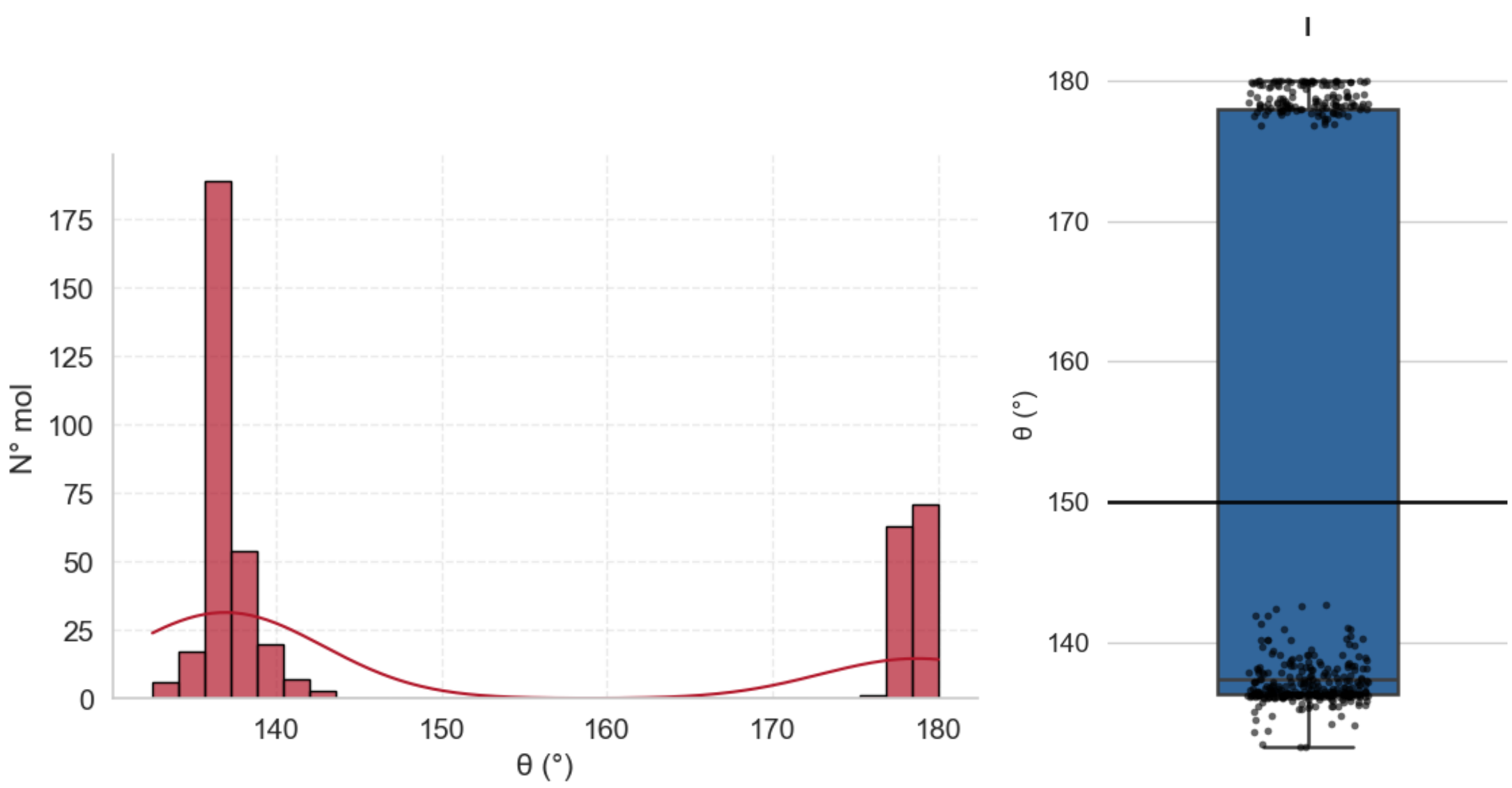

Figure S2: Statistical distribution of the bond angle (θ) of the O=C=O group in the analyzed molecular dataset computed at the CAM-B3LYP/6-311++G(d,p) theory level. On the left, a histogram with its corresponding density curve; on the right, a boxplot with superimposed individual data points.

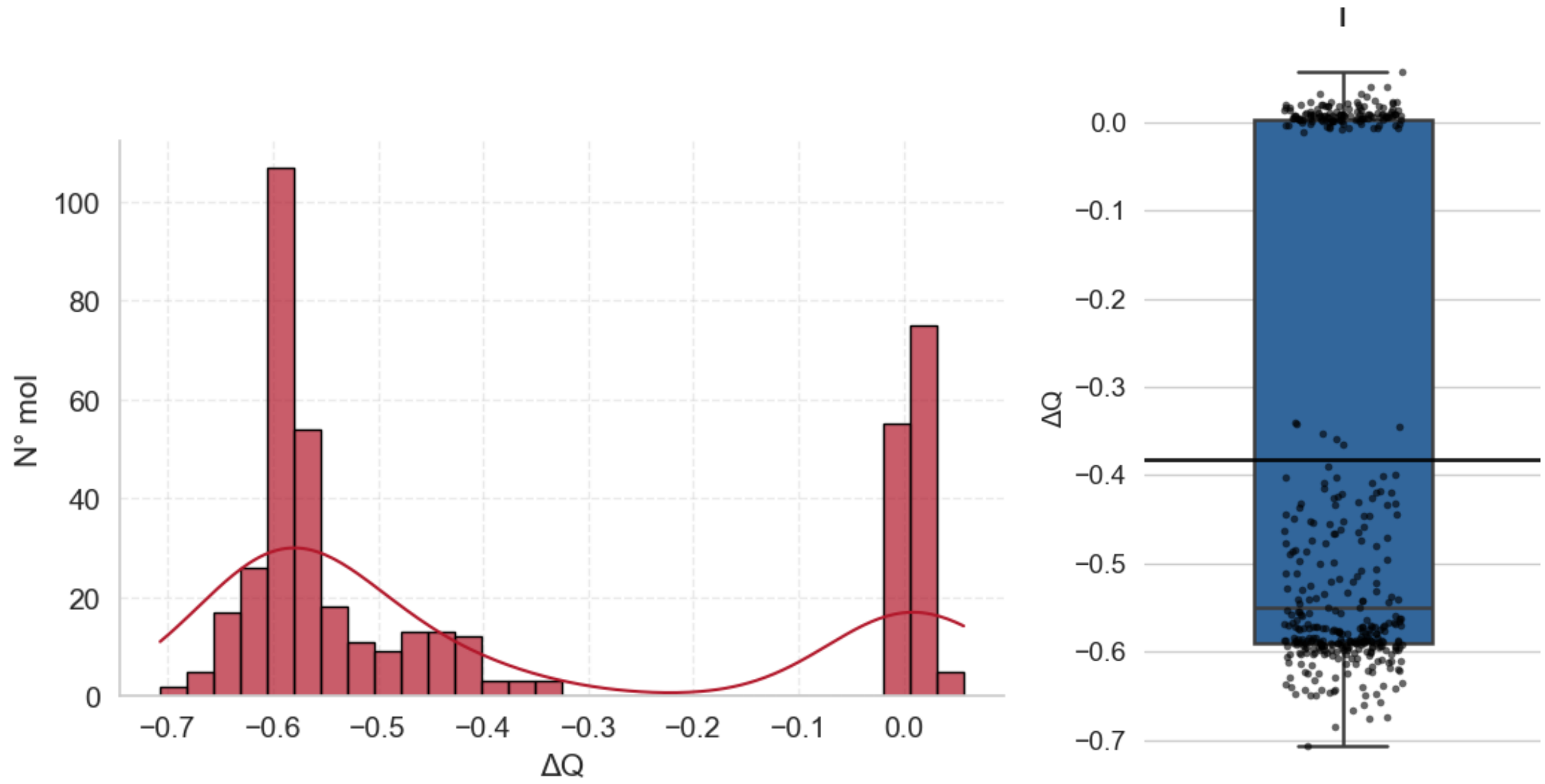

Figure S3: Statistical distribution of the charge transfer (ΔQ) in the analyzed molecular dataset computed at the CAM-B3LYP/6-311++G(d,p) theory level. On the left, a histogram with its corresponding density curve; on the right, a boxplot with superimposed individual data points.

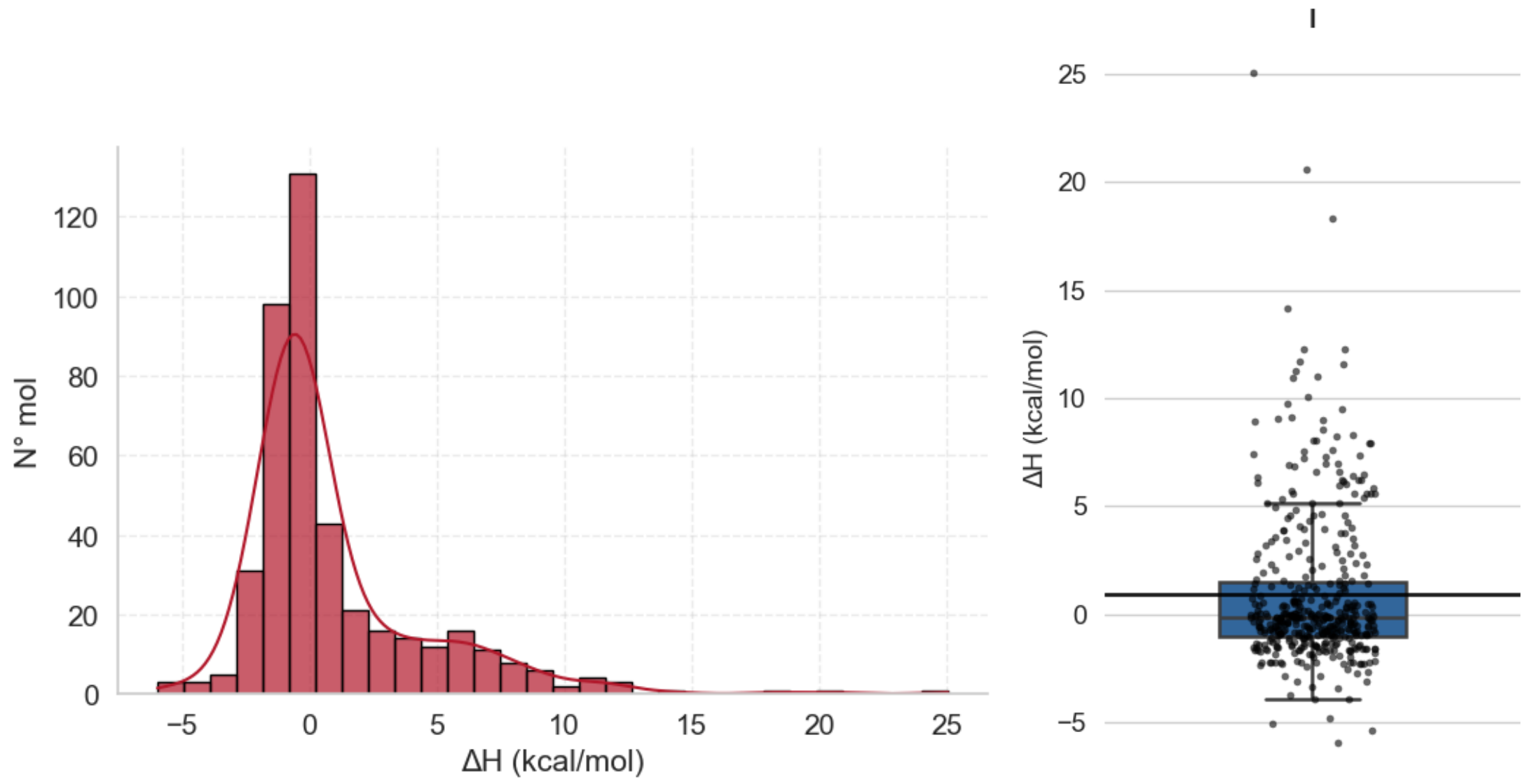

Figure S4: Statistical distribution of the enthalpy (ΔH) in the analyzed molecular dataset computed at the CAM-B3LYP/6-311++G(d,p) theory level. On the left, a histogram with

its corresponding density curve; on the right, a boxplot with superimposed individual data points.

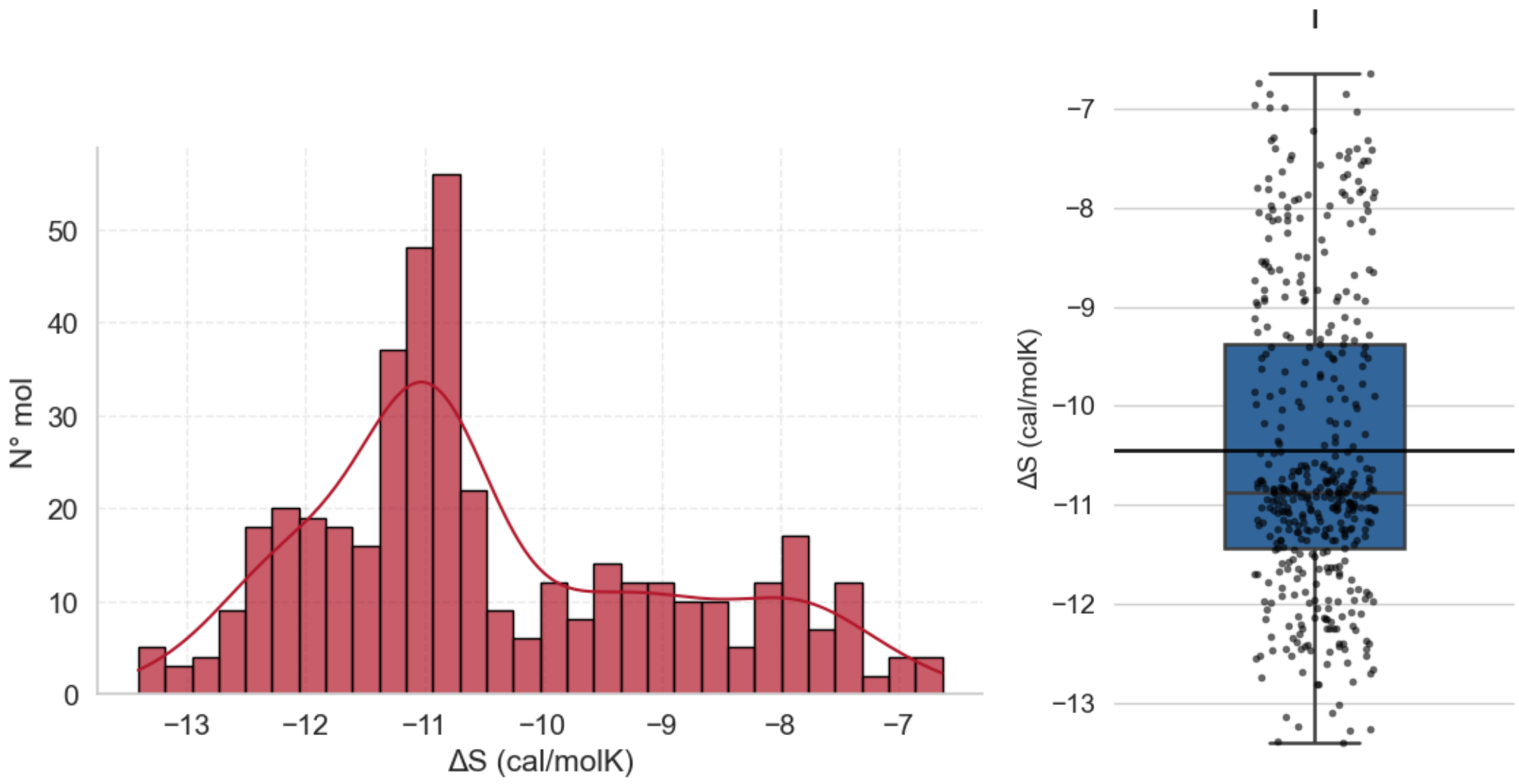

Figure S5: Statistical distribution of the entropy (ΔS) in the analyzed molecular dataset computed at the CAM-B3LYP/6-311++G(d,p) theory level. On the left, a histogram with its corresponding density curve; on the right, a boxplot with superimposed individual data points.

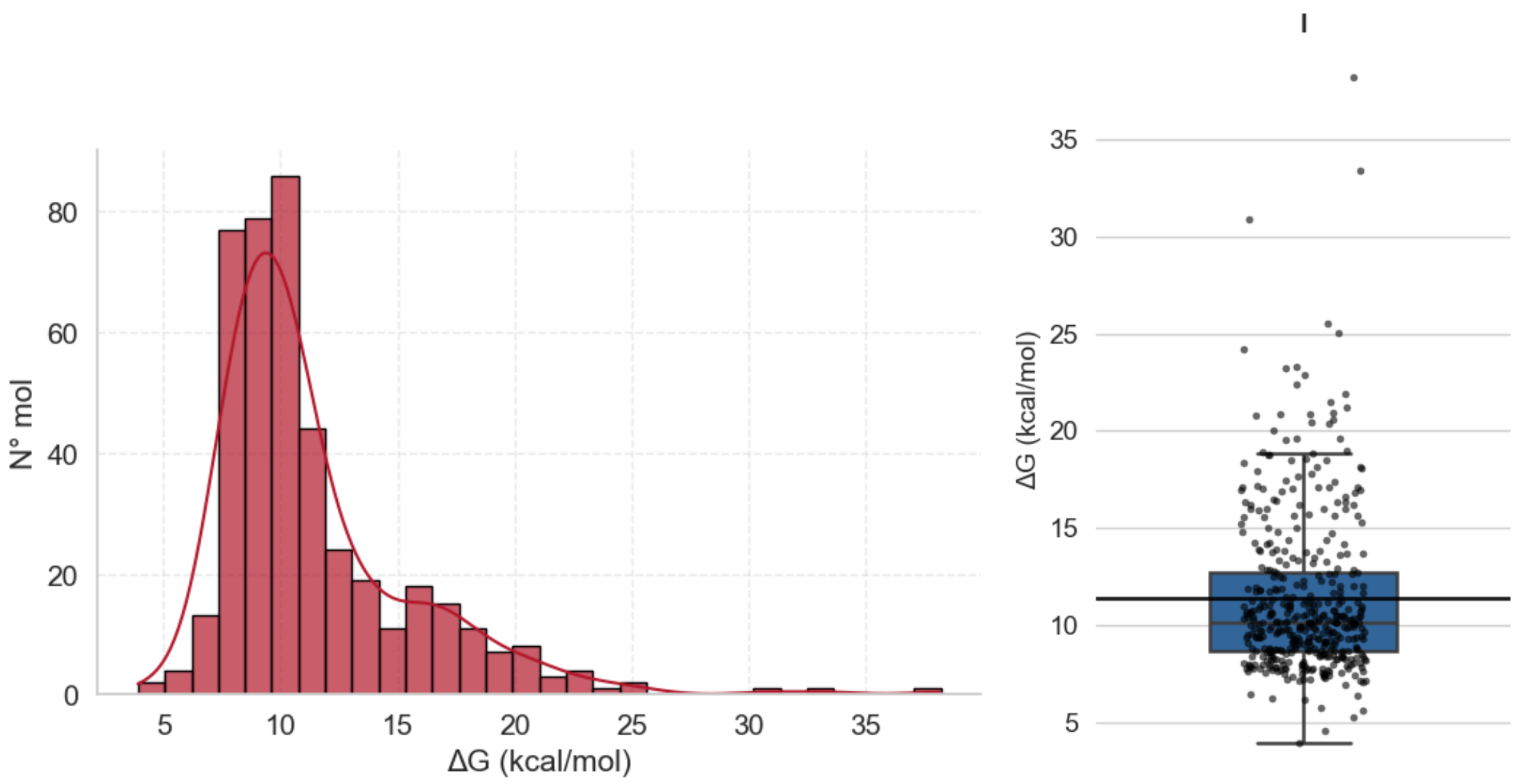

Figure S6: Statistical distribution of the Gibbs free energy (ΔG) in the analyzed molecular dataset computed at the CAM-B3LYP/6-311++G(d,p) theory level. On the left, a histogram with its corresponding density curve; on the right, a boxplot with superimposed individual data points.

S3. 2 Population distribution analysis and histograms of geometric, thermodynamic, and charge-transfer data calculated using AM1 method

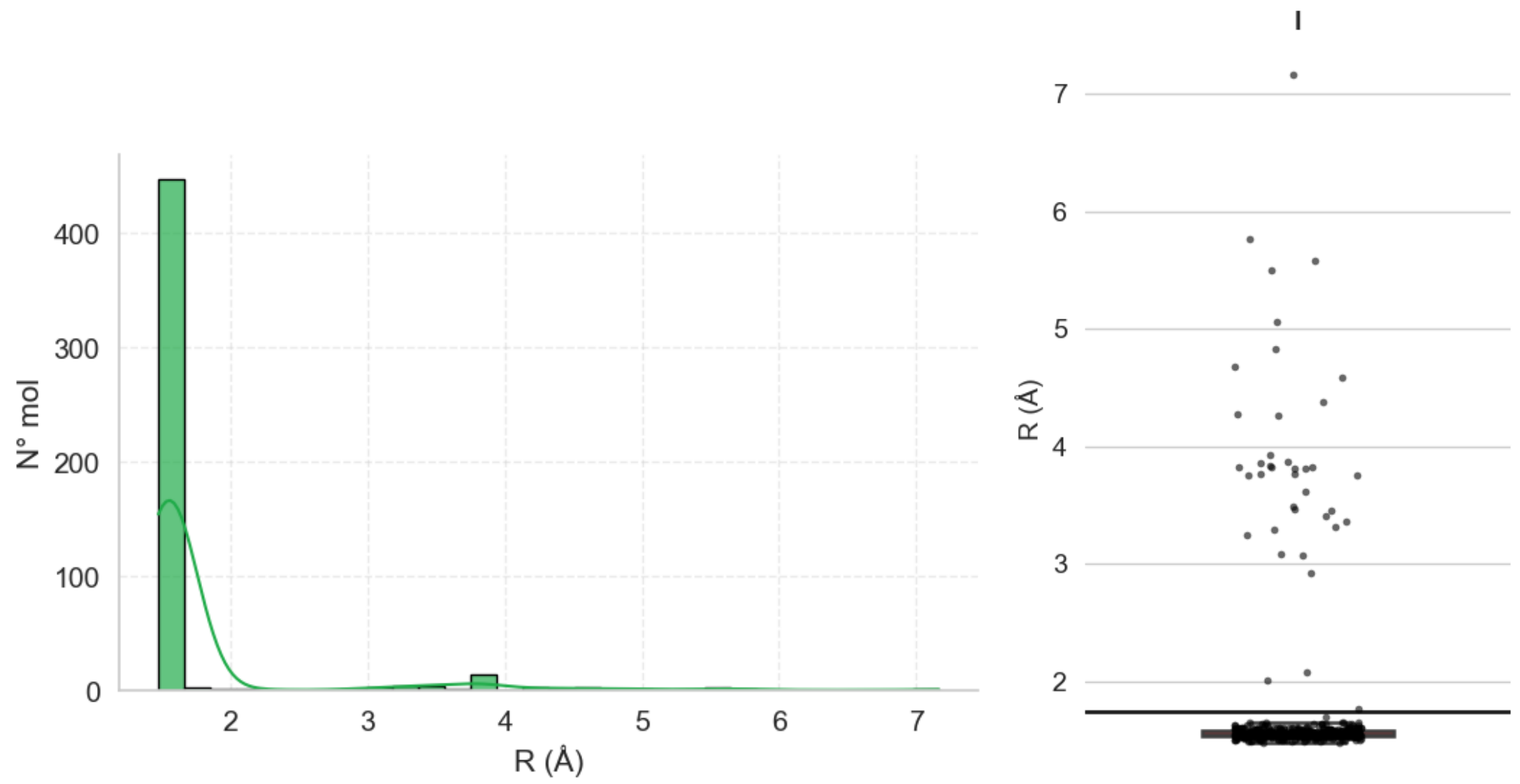

Figure S7: Statistical distribution of the length R (Å) in the analyzed molecular dataset computed at the AM1 method. On the left, a histogram with its corresponding density curve; on the right, a boxplot with superimposed individual data points.

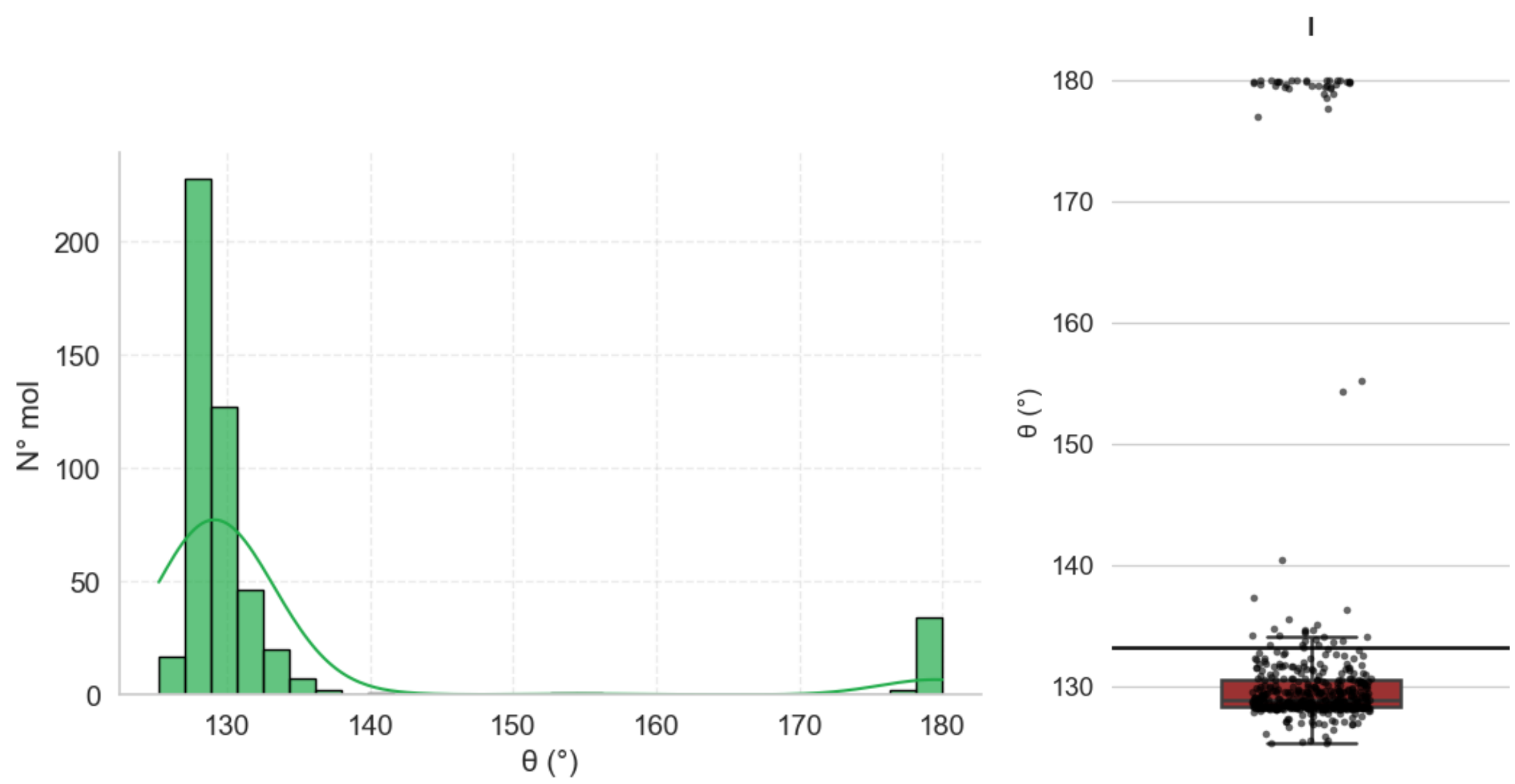

Figure S8: Statistical distribution of the bond angle (θ) of the O=C=O group in the analyzed molecular dataset computed at the AM1 method. On the left, a histogram with its corresponding density curve; on the right, a boxplot with superimposed individual data points.

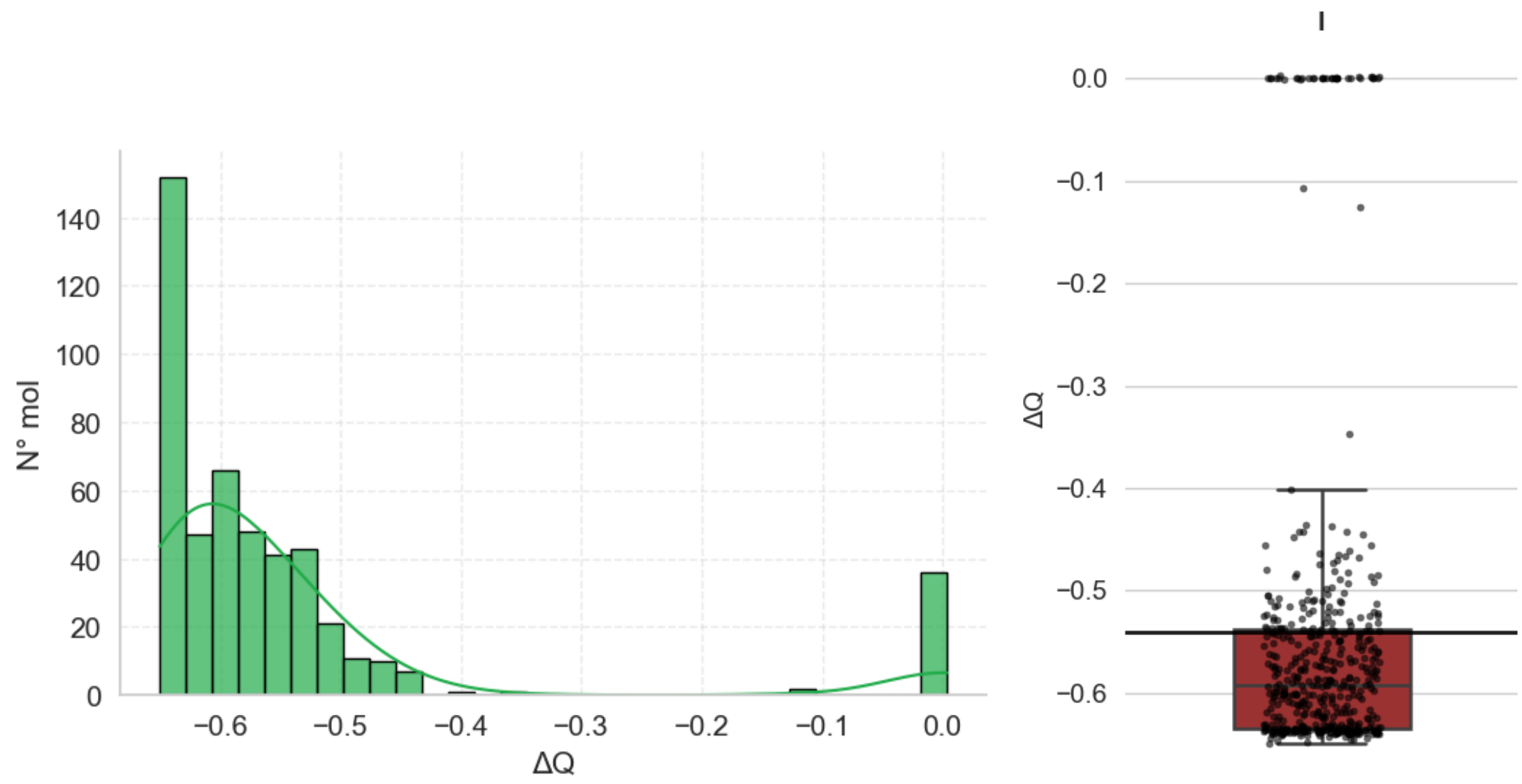

Figure S9: Statistical distribution of the charge transfer (ΔQ) in the analyzed molecular dataset computed at the CAM-B3LYP/6-311++G(d,p) theory level. On the left, a histogram with its corresponding density curve; on the right, a boxplot with superimposed individual data points.

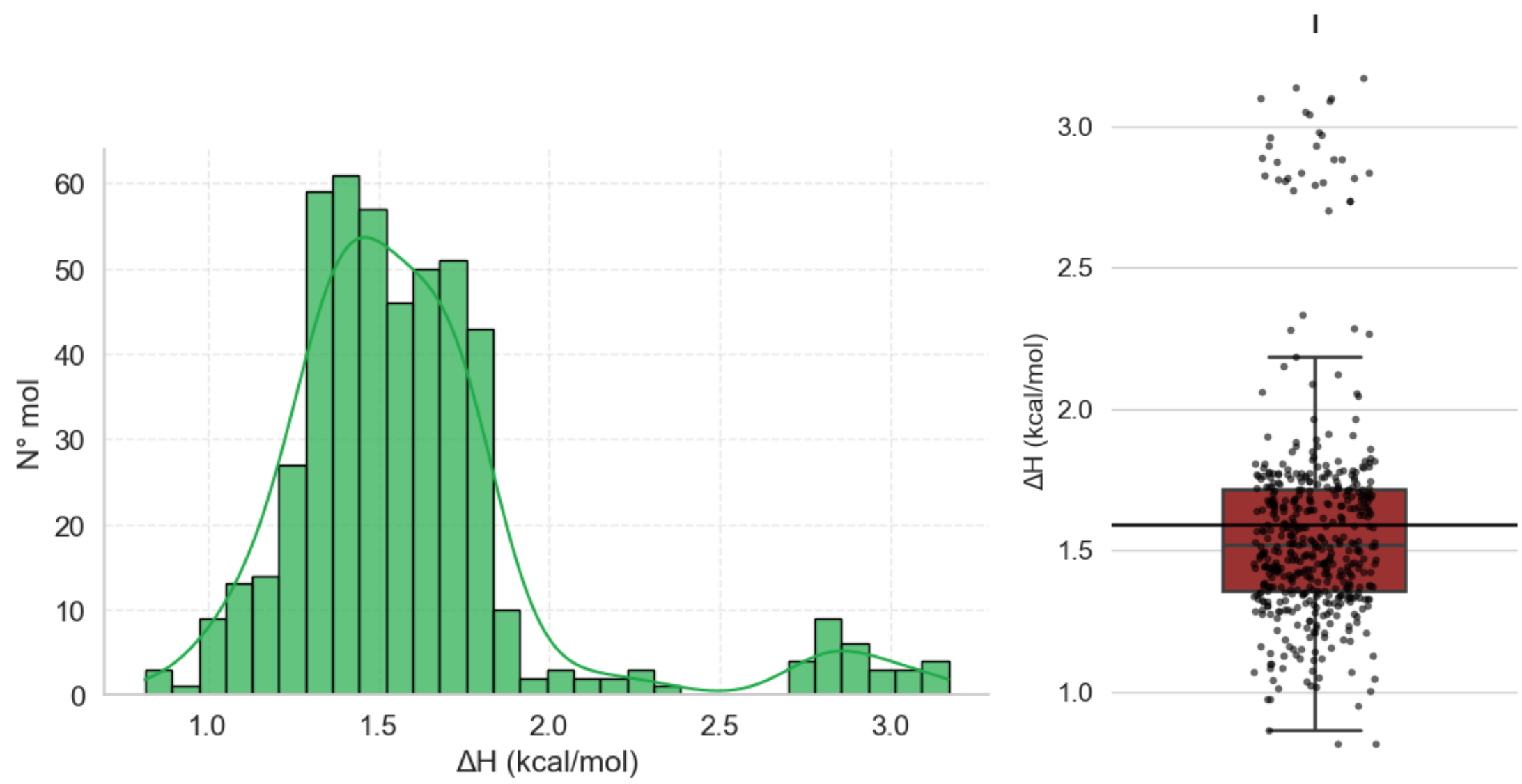

Figure S10: Statistical distribution of the enthalpy (ΔH) in the analyzed molecular dataset computed at the AM1 method. On the left, a histogram with its corresponding density curve; on the right, a boxplot with superimposed individual data points.

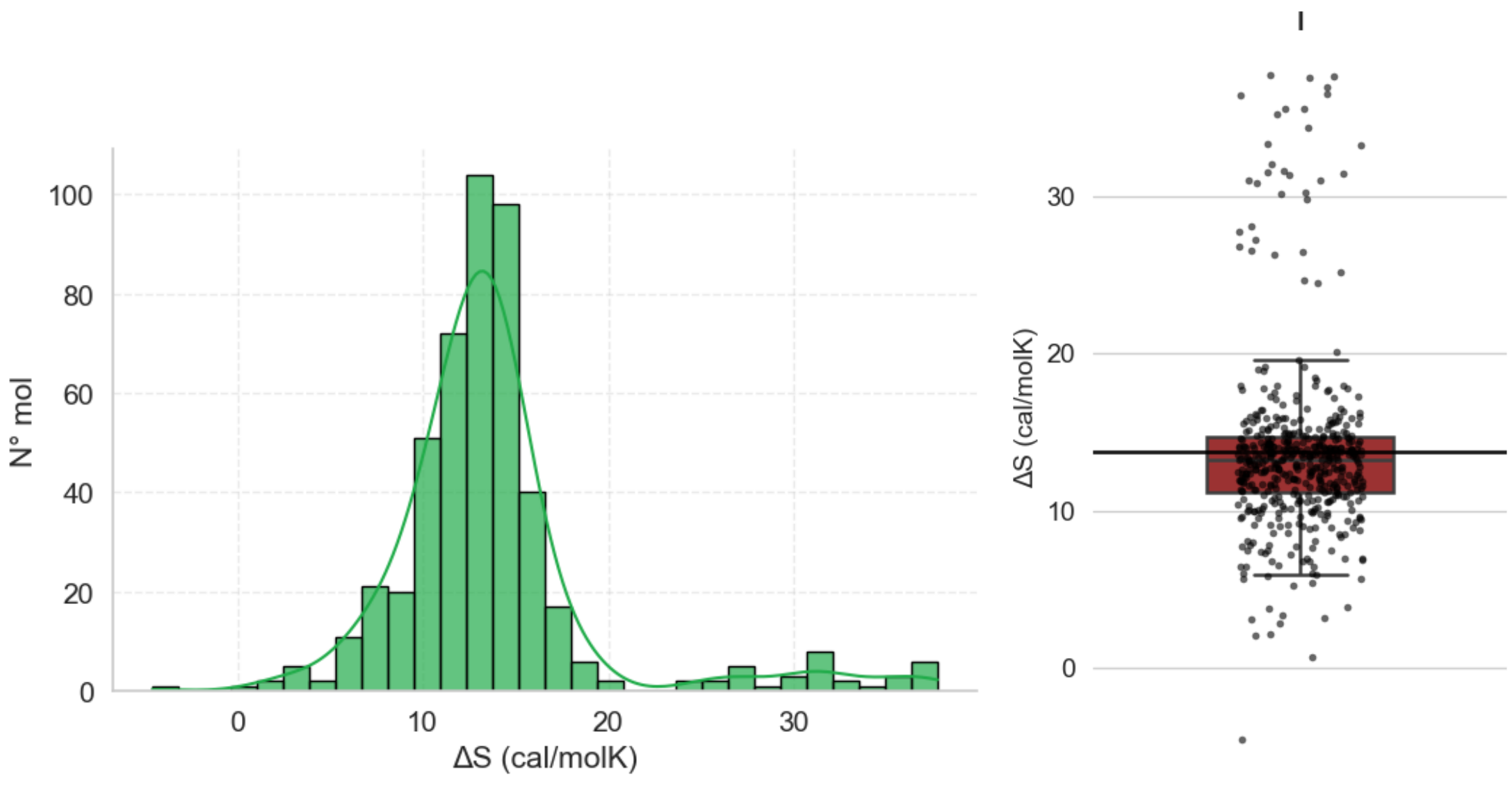

Figure S11: Statistical distribution of the entropy (ΔS) in the analyzed molecular dataset computed at the AM1 method. On the left, a histogram with its corresponding density curve; on the right, a boxplot with superimposed individual data points.

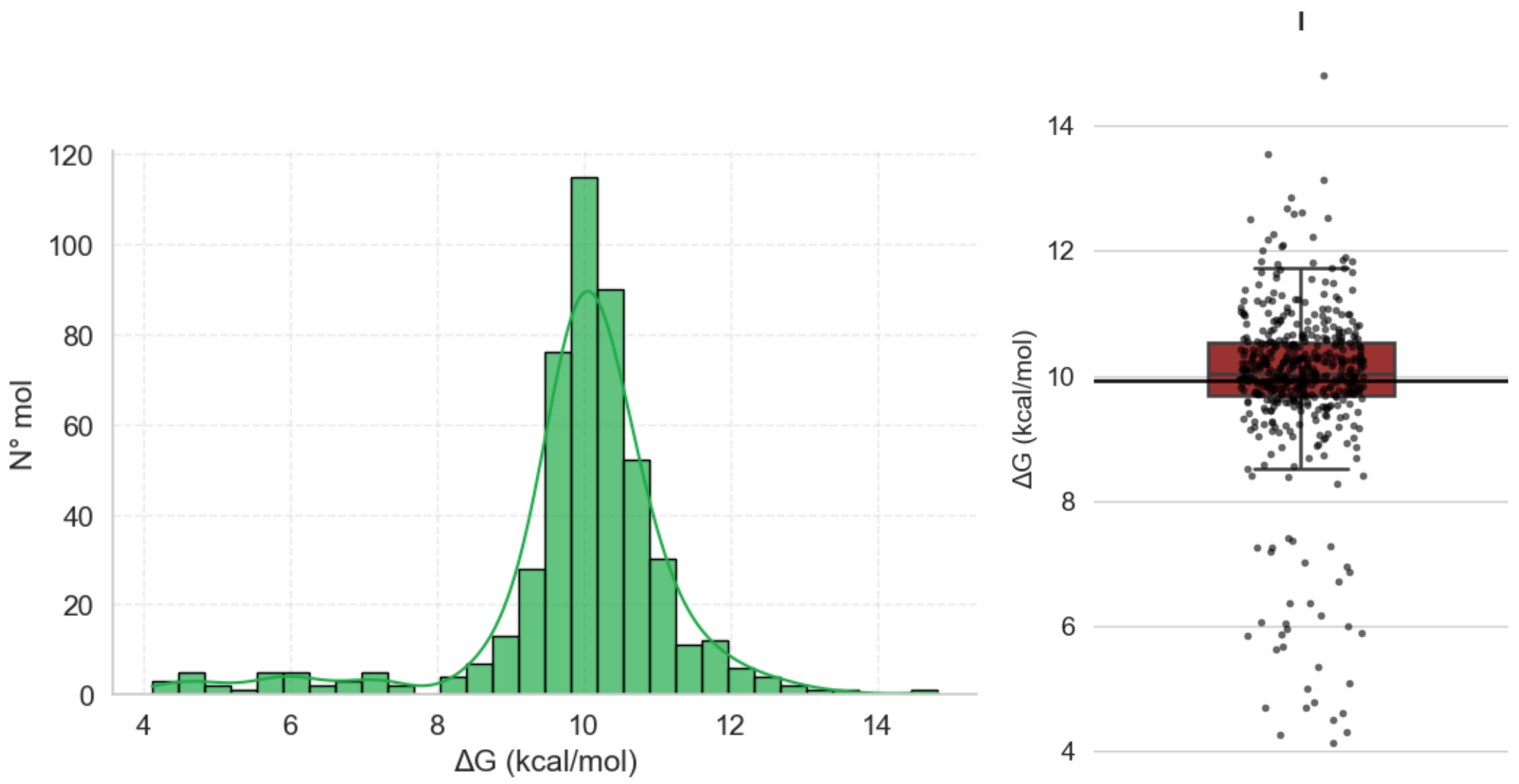

Figure S12: Statistical distribution of the Gibbs free energy (ΔG) in the analyzed molecular dataset computed at the AM1 method. On the left, a histogram with its corresponding density curve; on the right, a boxplot with superimposed individual data points.

S3. 3 Population distribution analysis and histograms of geometric, thermodynamic, and charge-transfer data calculated using GFN0 method

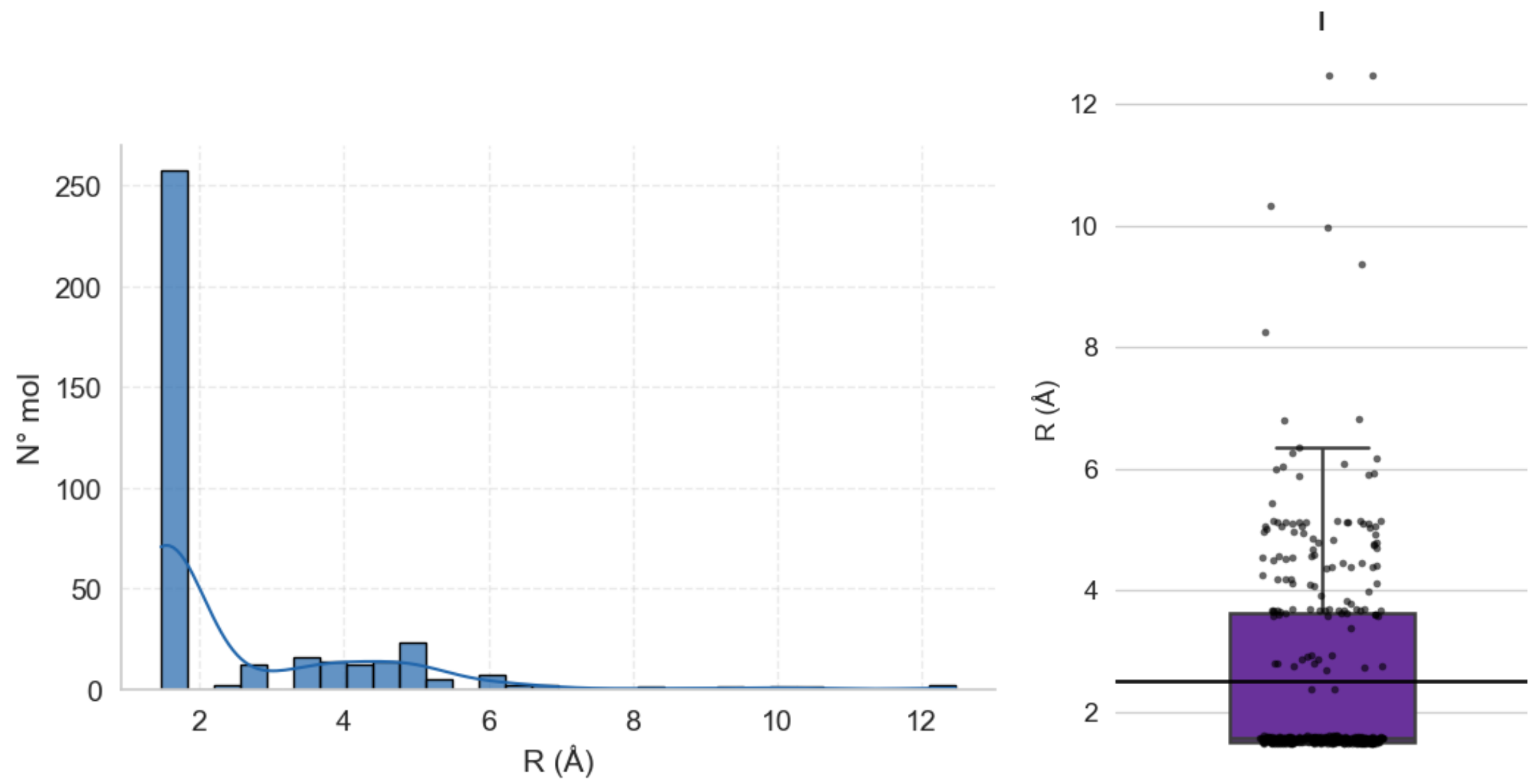

Figure S13: Statistical distribution of the length R (Å) in the analyzed molecular dataset computed at the GFN0 method. On the left, a histogram with its corresponding density curve; on the right, a boxplot with superimposed individual data points.

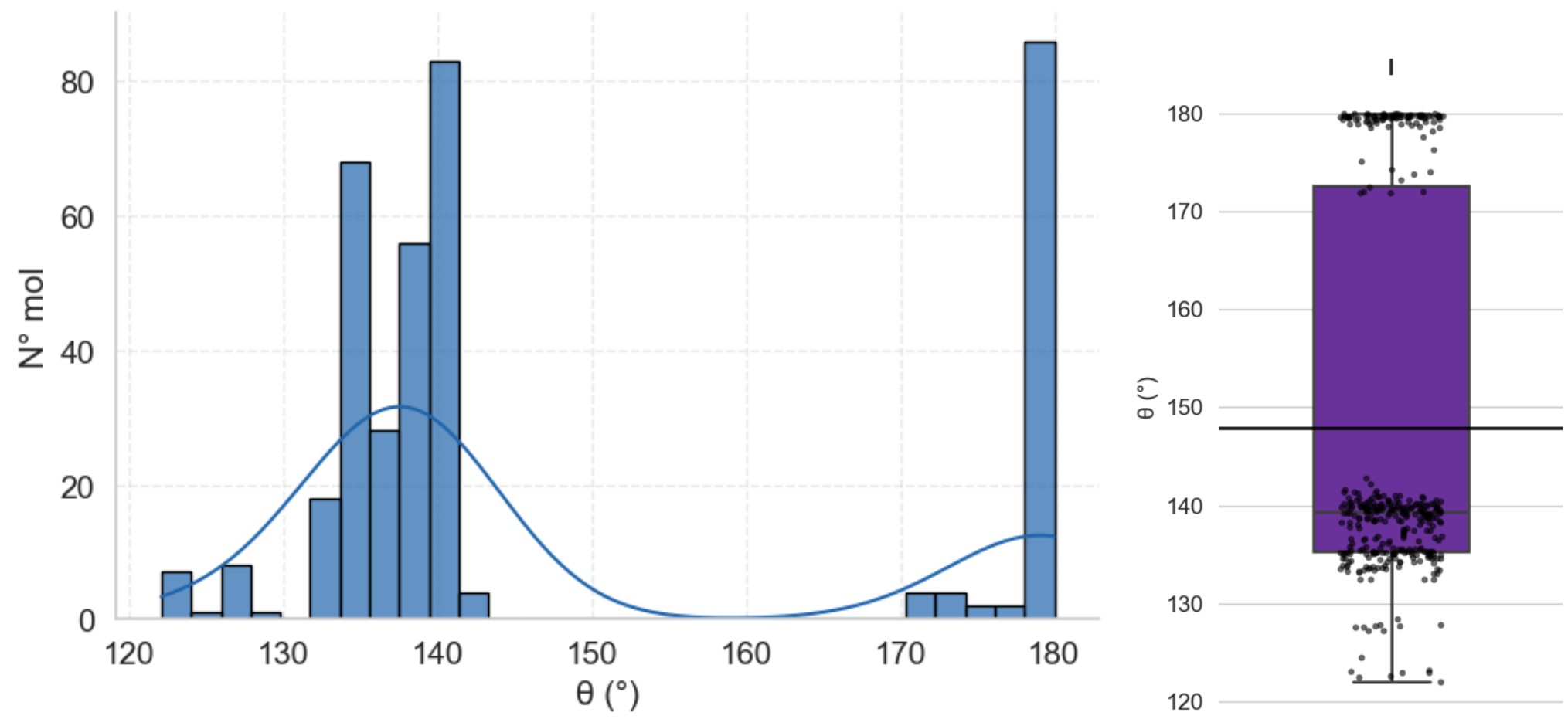

Figure S14: Statistical distribution of the bond angle (θ) of the O=C=O group in the analyzed molecular dataset computed at the GFN0 method. On the left, a histogram with its corresponding density curve; on the right, a boxplot with superimposed individual data points.

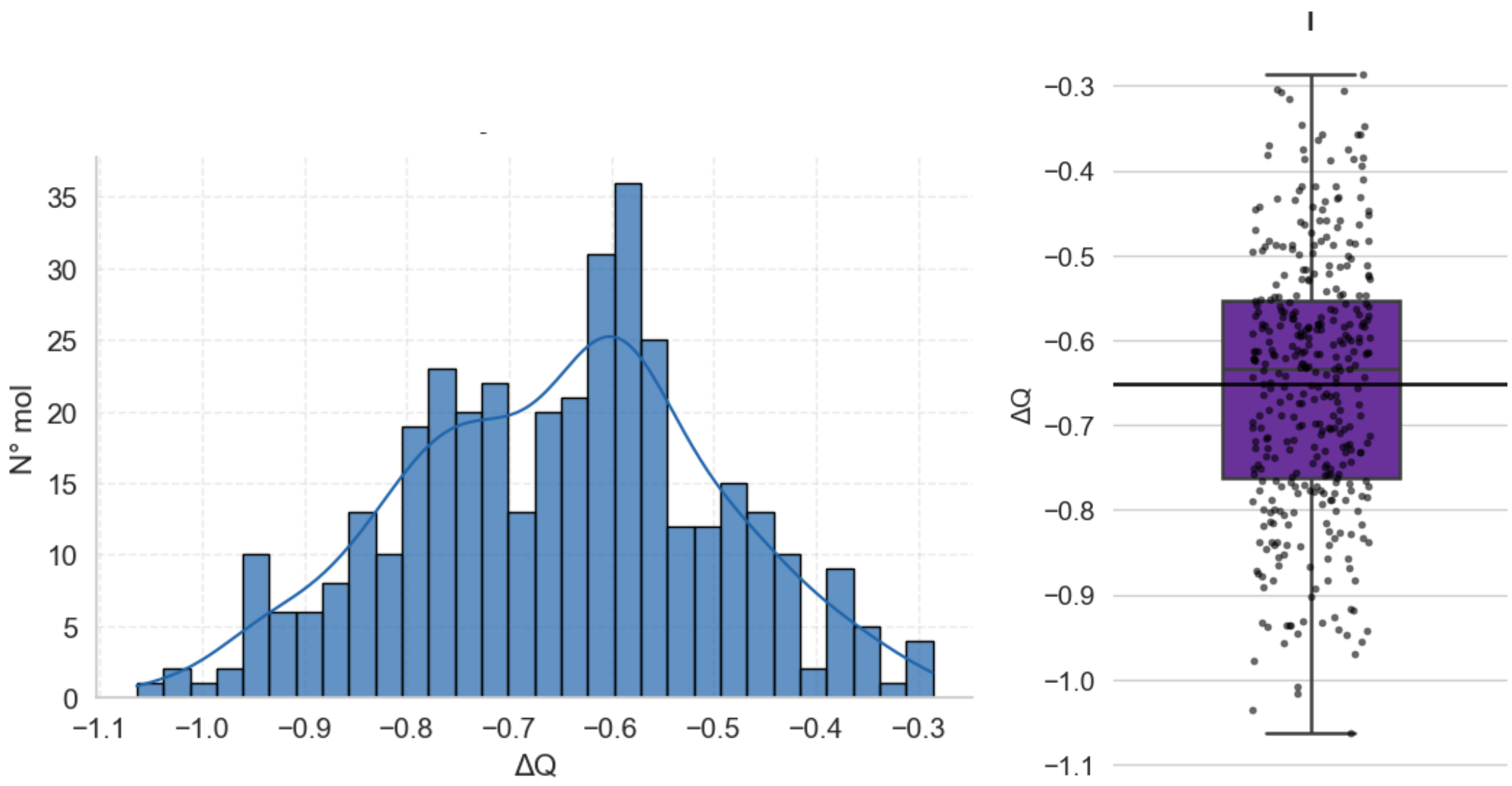

Figure S15: Statistical distribution of the charge transfer (ΔQ) in the analyzed molecular dataset computed at the GFN0 method. On the left, a histogram with its corresponding density curve; on the right, a boxplot with superimposed individual data points.

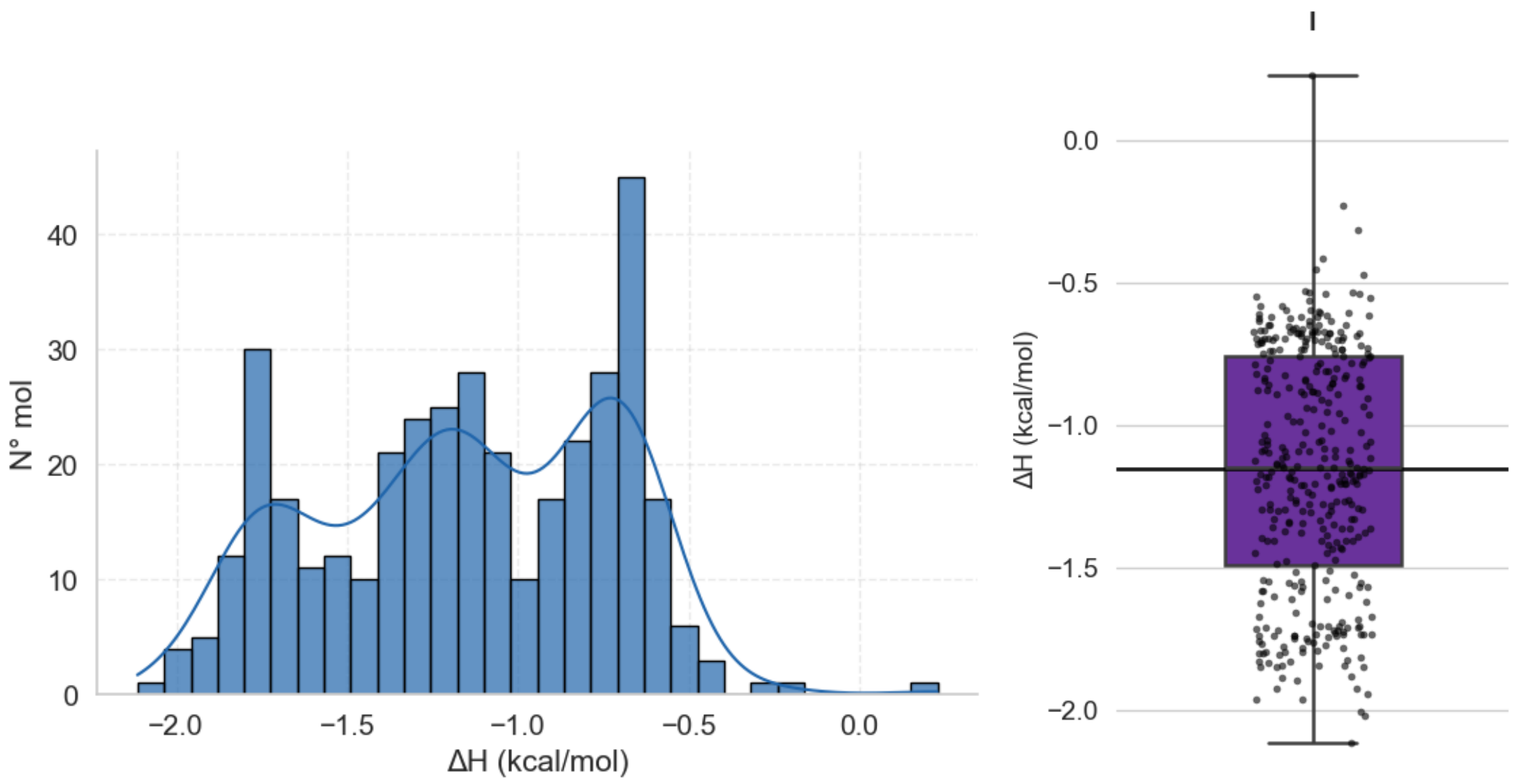

Figure S16: Statistical distribution of the enthalpy (ΔH) in the analyzed molecular dataset computed at the GFN0 method. On the left, a histogram with its corresponding density curve; on the right, a boxplot with superimposed individual data points.

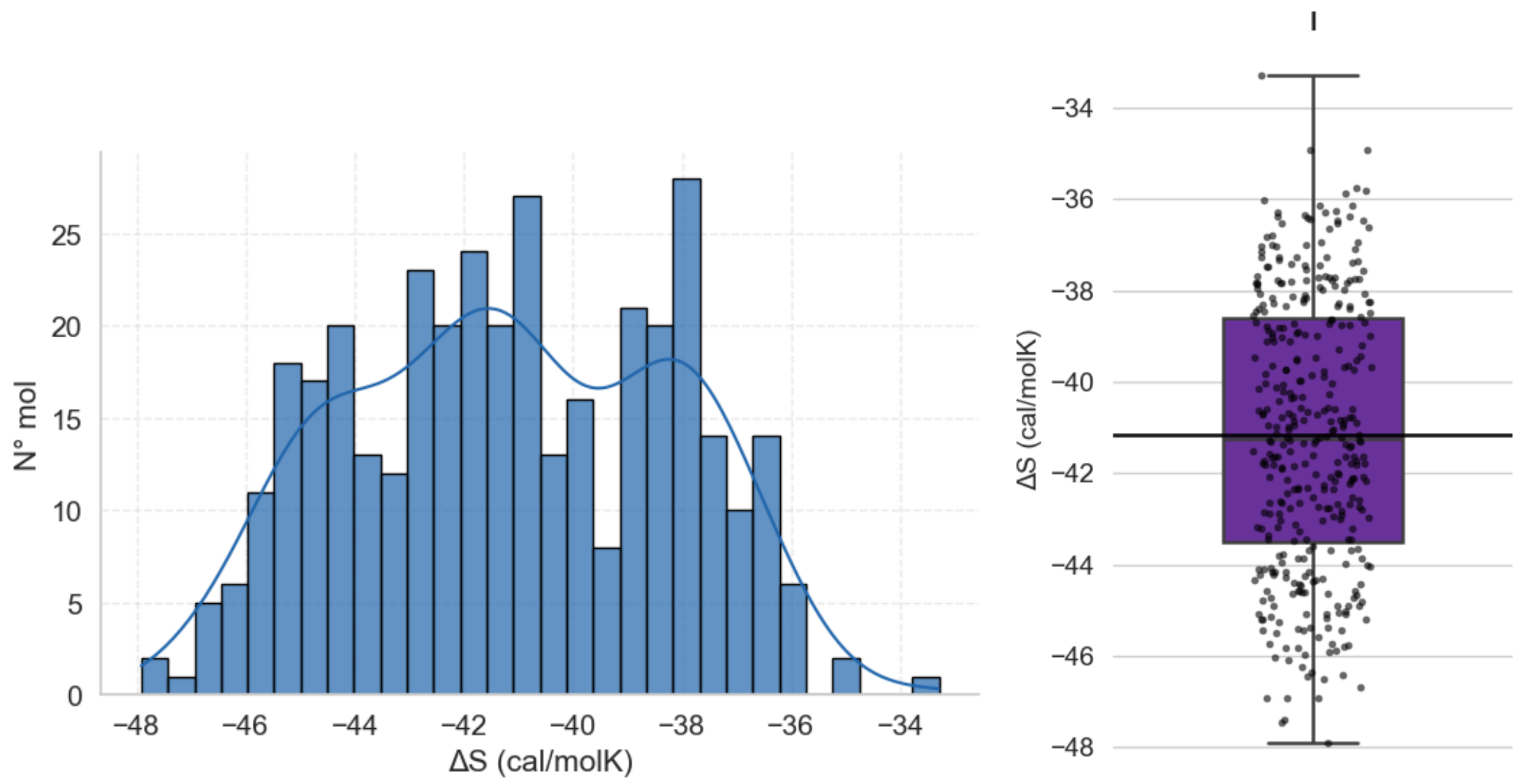

Figure S17: Statistical distribution of the entropy (ΔS) in the analyzed molecular dataset computed at the GFN0 method. On the left, a histogram with its corresponding density curve; on the right, a boxplot with superimposed individual data points.

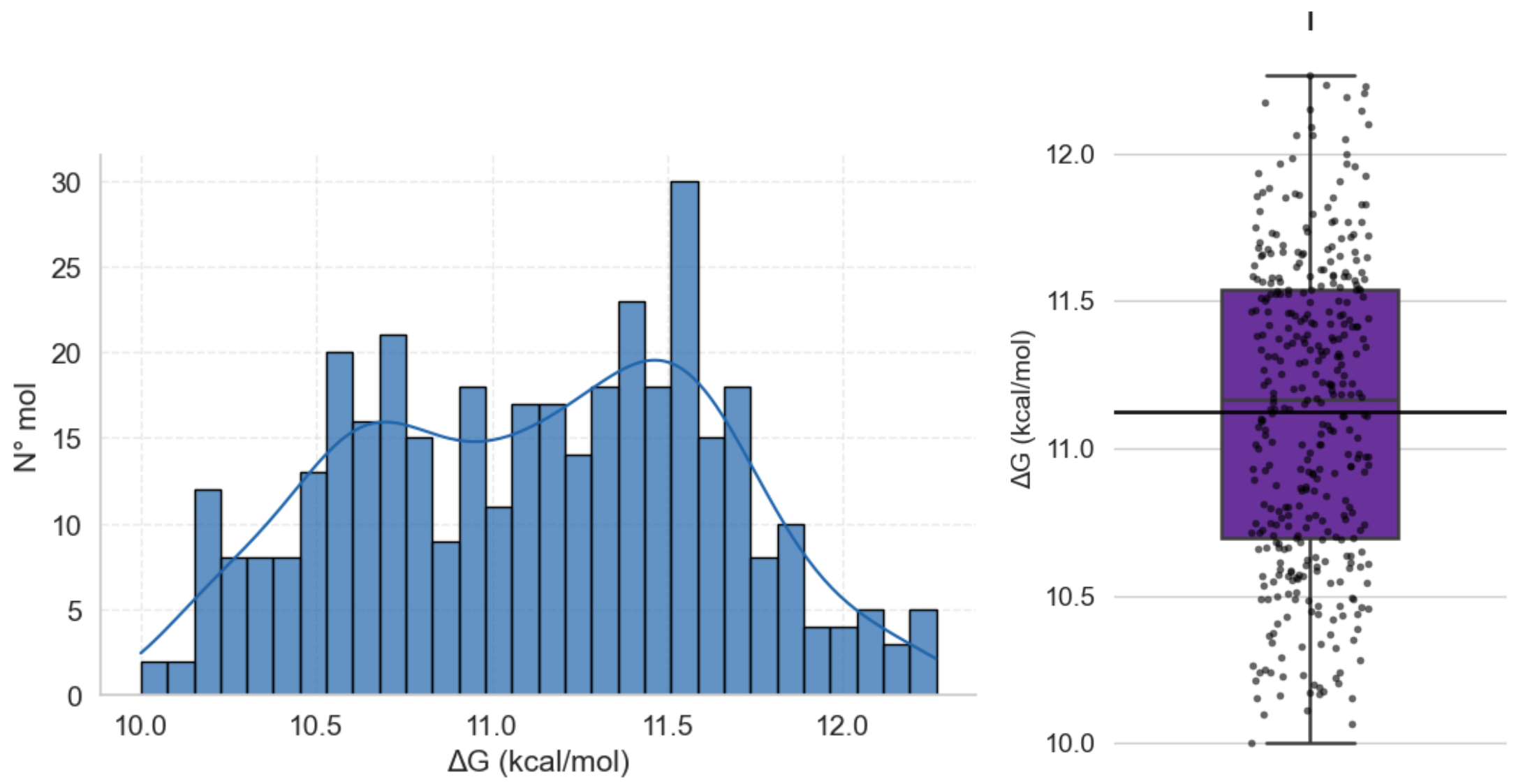

Figure S18: Statistical distribution of the Gibbs free energy (ΔG) in the analyzed molecular dataset computed at the GFN0 method. On the left, a histogram with its corresponding density curve; on the right, a boxplot with superimposed individual data points.

S3. 4 Population distribution analysis and histograms of geometric, thermodynamic, and charge-transfer data calculated using GFN1 method

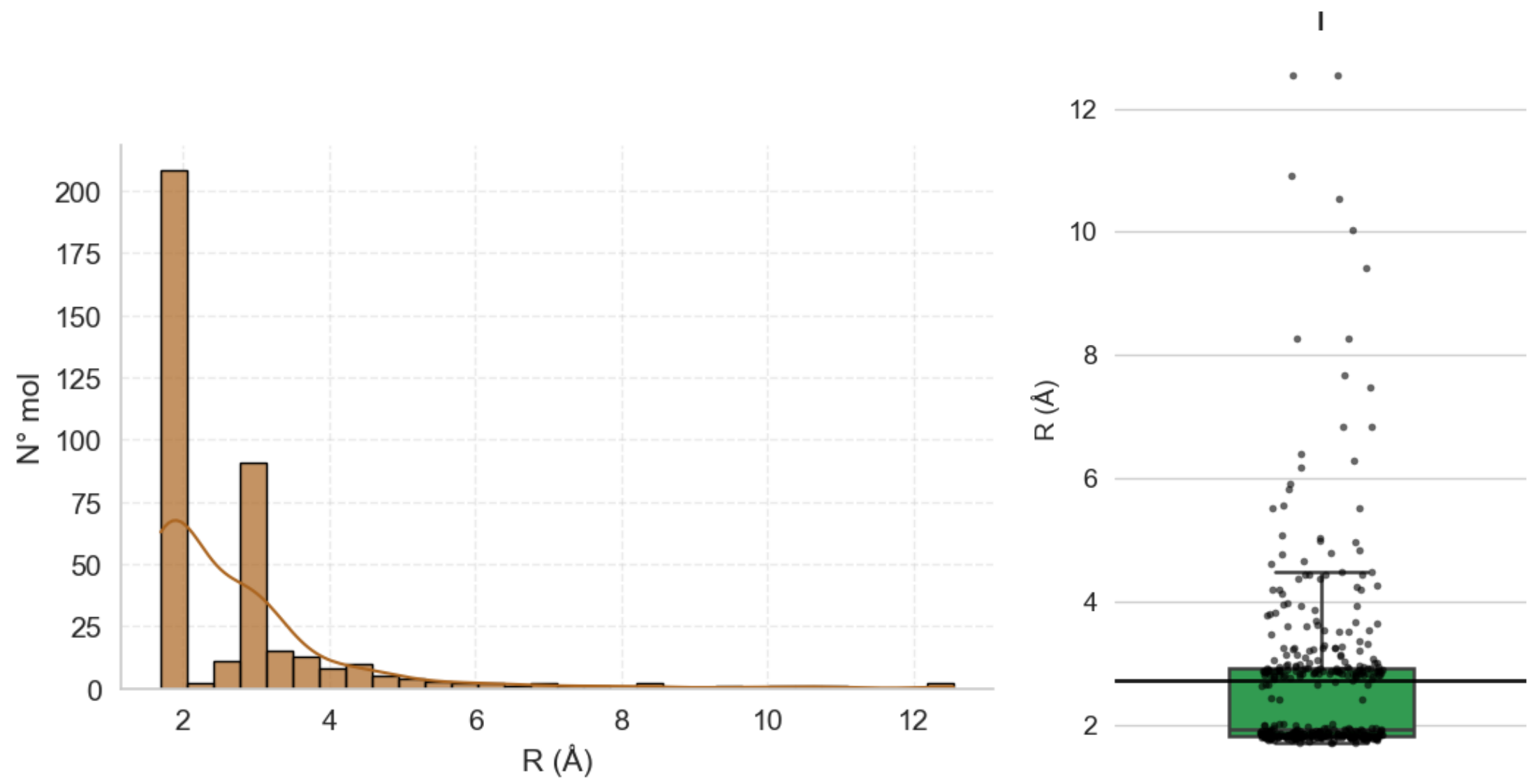

Figure S19: Statistical distribution of the length R (Å) in the analyzed molecular dataset computed at the GFN1 method. On the left, a histogram with its corresponding density curve; on the right, a boxplot with superimposed individual data points.

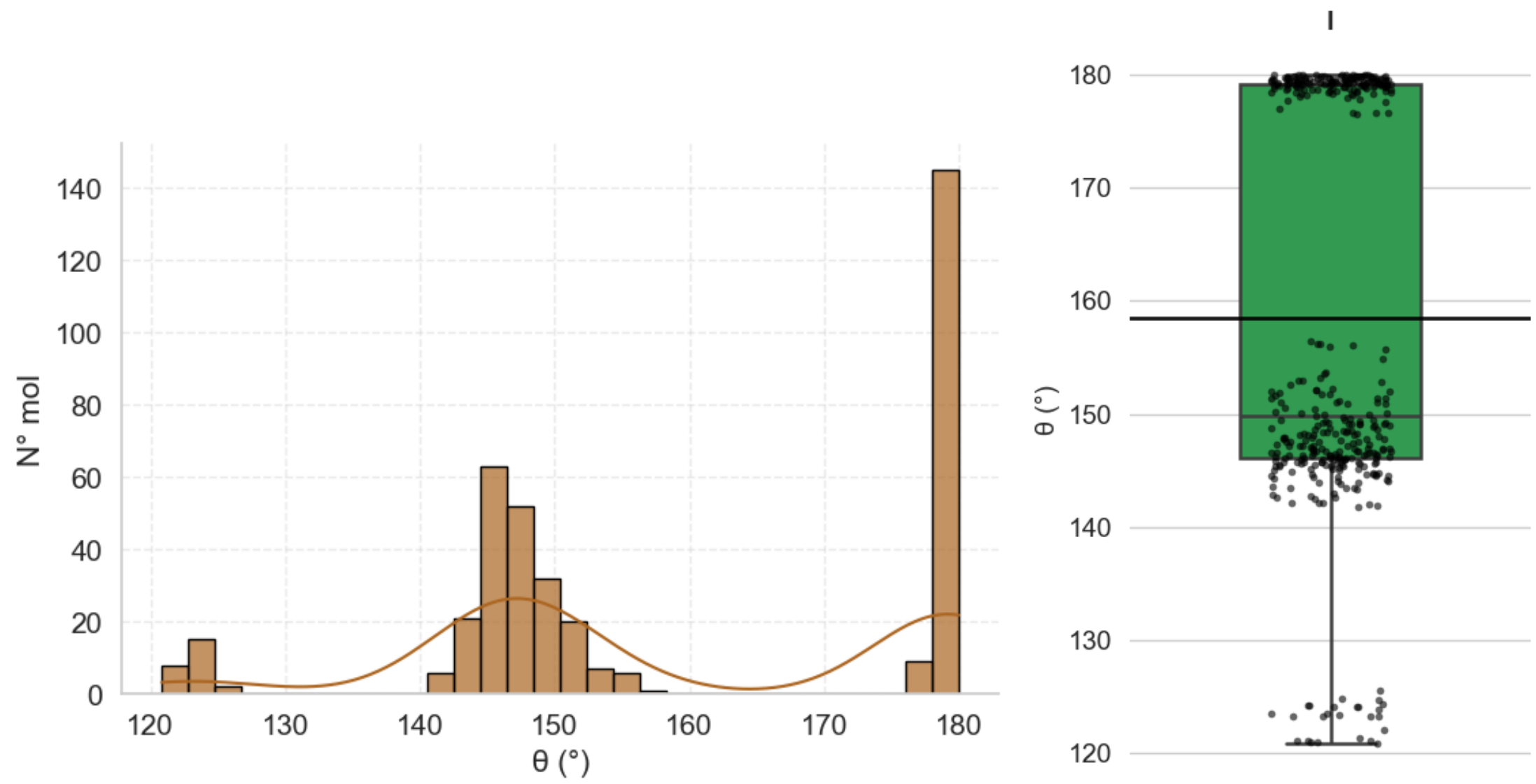

Figure S20: Statistical distribution of the bond angle (θ) of the O=C=O group in the analyzed molecular dataset computed at the GFN1 method. On the left, a histogram with its corresponding density curve; on the right, a boxplot with superimposed individual data points.

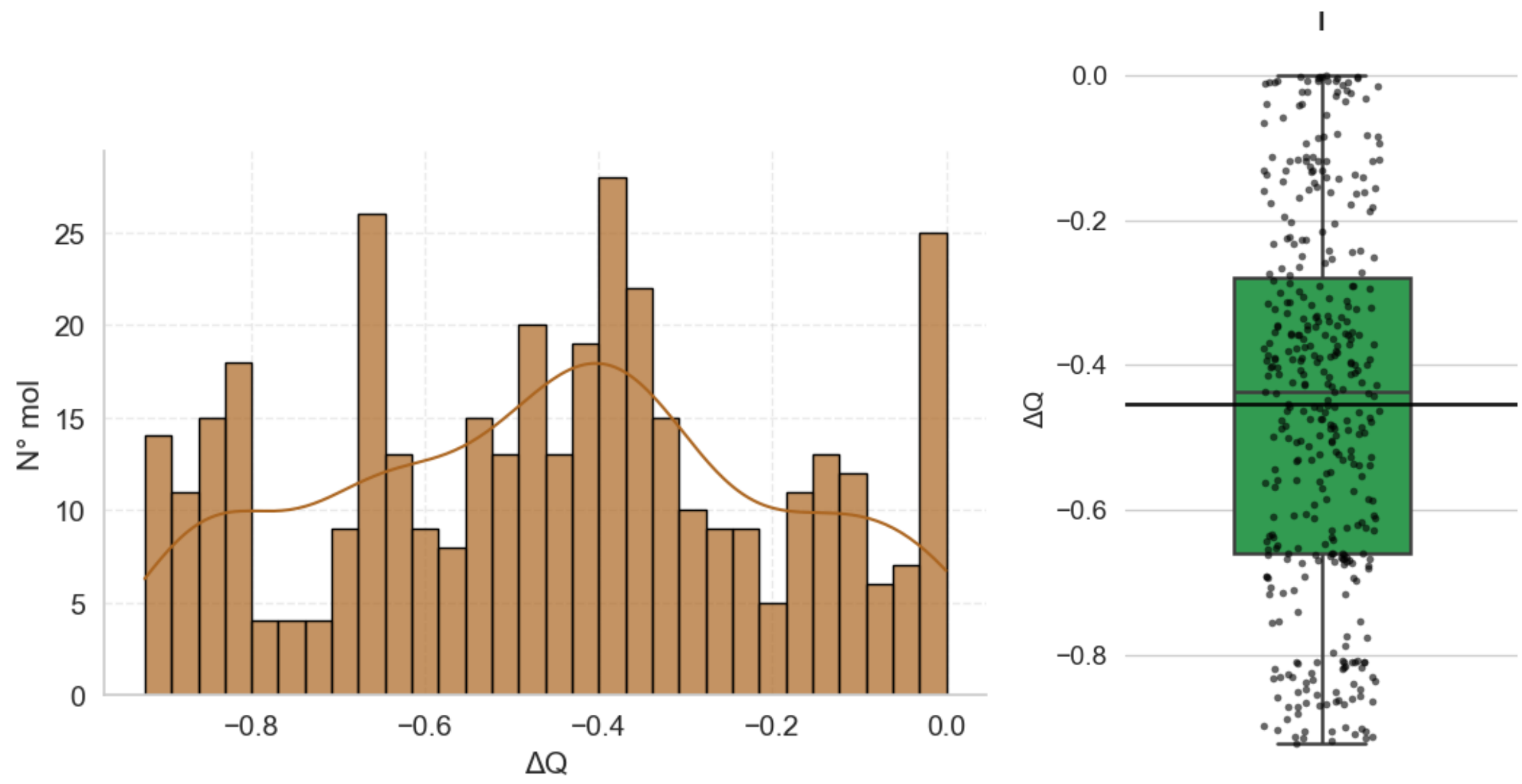

Figure S21: Statistical distribution of the charge transfer (ΔQ) in the analyzed molecular dataset computed at the GFN1 method. On the left, a histogram with its corresponding density curve; on the right, a boxplot with superimposed individual data points.

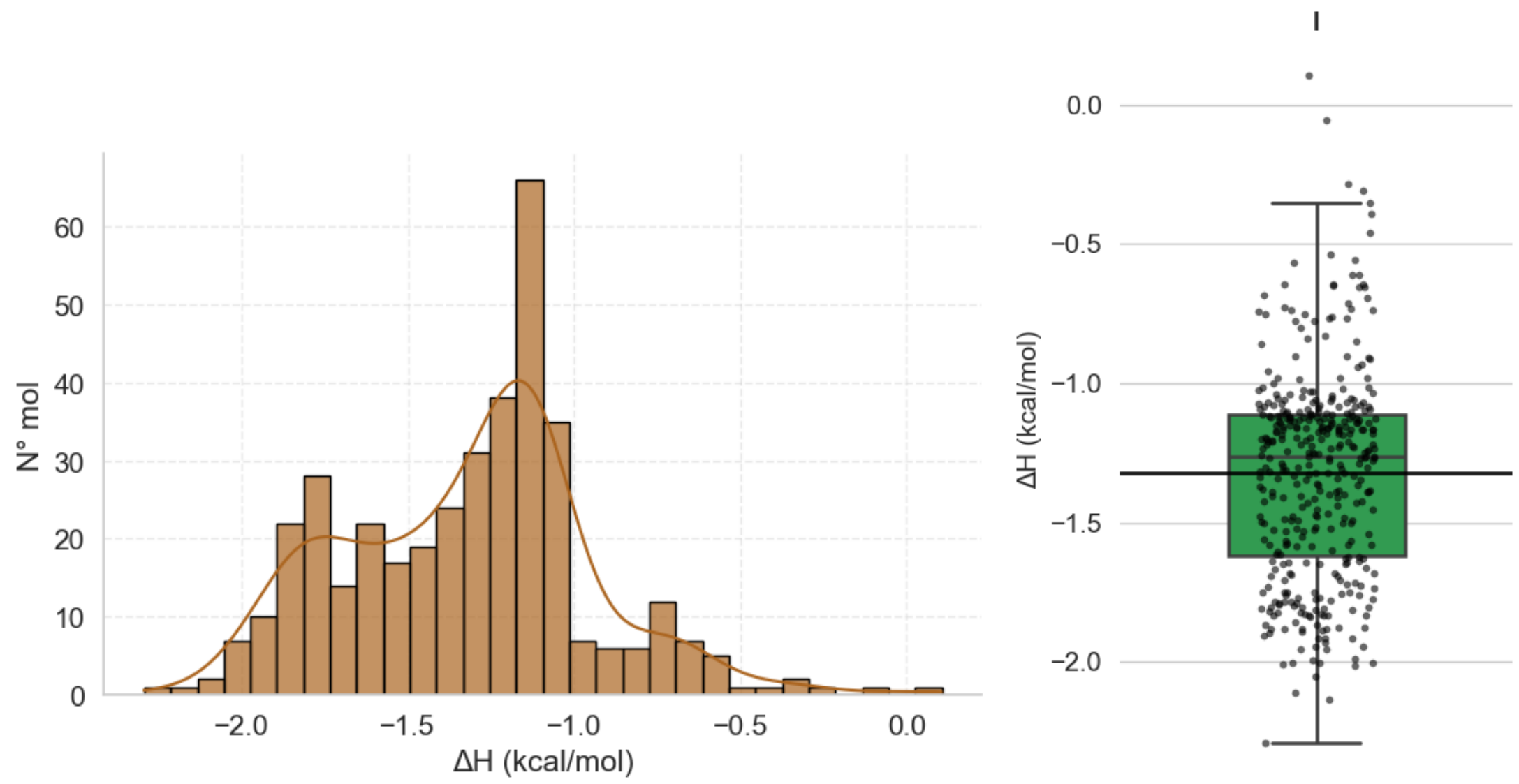

Figure S22: Statistical distribution of the enthalpy (ΔH) in the analyzed molecular dataset computed at the GFN1 method. On the left, a histogram with its corresponding density curve; on the right, a boxplot with superimposed individual data points.

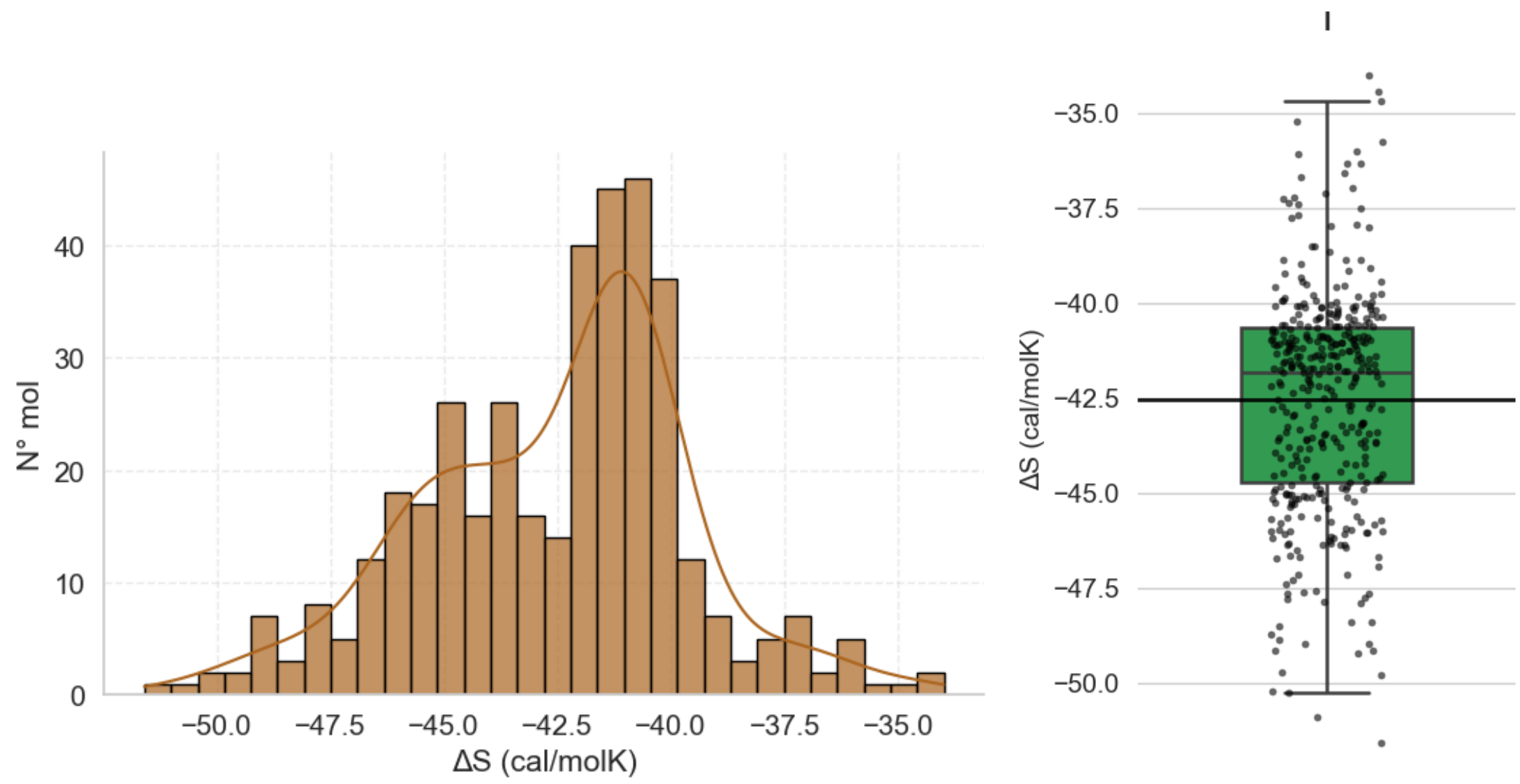

Figure S23: Statistical distribution of the entropy (ΔS) in the analyzed molecular dataset computed at the GFN1 method. On the left, a histogram with its corresponding density curve; on the right, a boxplot with superimposed individual data points.

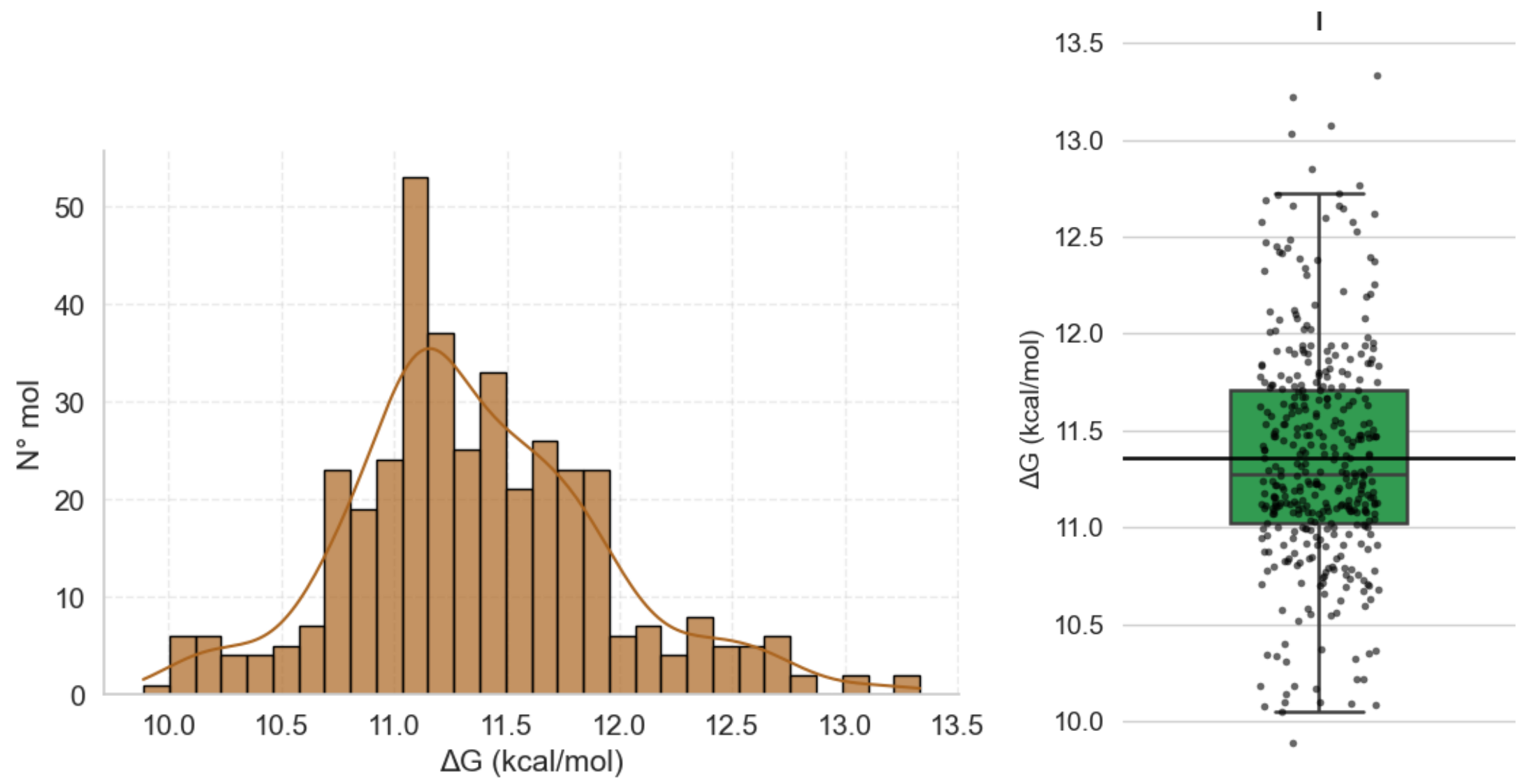

Figure S24: Statistical distribution of the Gibbs free energy (ΔG) in the analyzed molecular dataset computed at the GFN1 method. On the left, a histogram with its corresponding density curve; on the right, a boxplot with superimposed individual data points.

S3. 5 Population distribution analysis and histograms of geometric, thermodynamic, and charge-transfer data calculated using GFN2 method

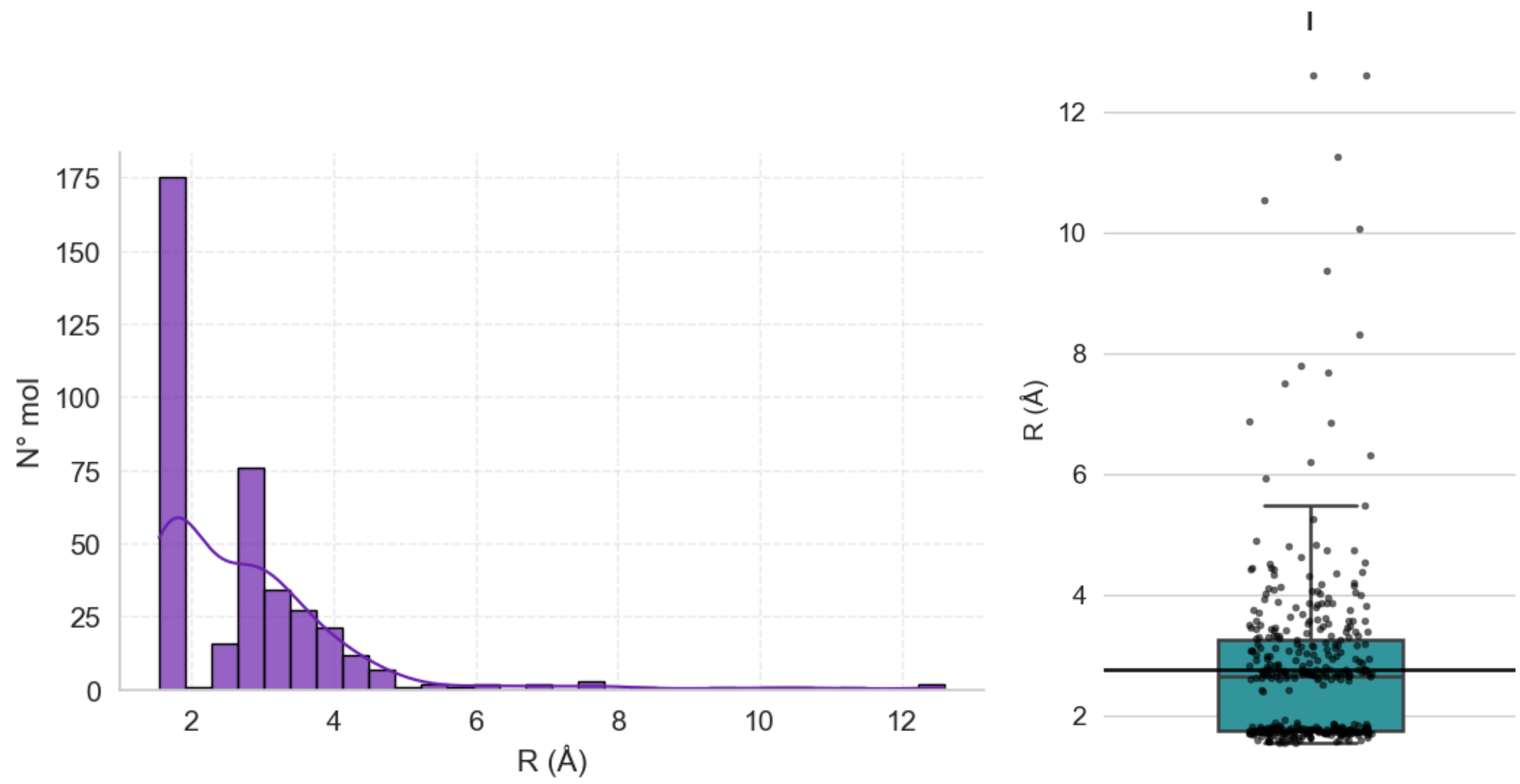

Figure S25: Statistical distribution of the length R (Å) in the analyzed molecular dataset computed at the GFN2 method. On the left, a histogram with its corresponding density curve; on the right, a boxplot with superimposed individual data points.

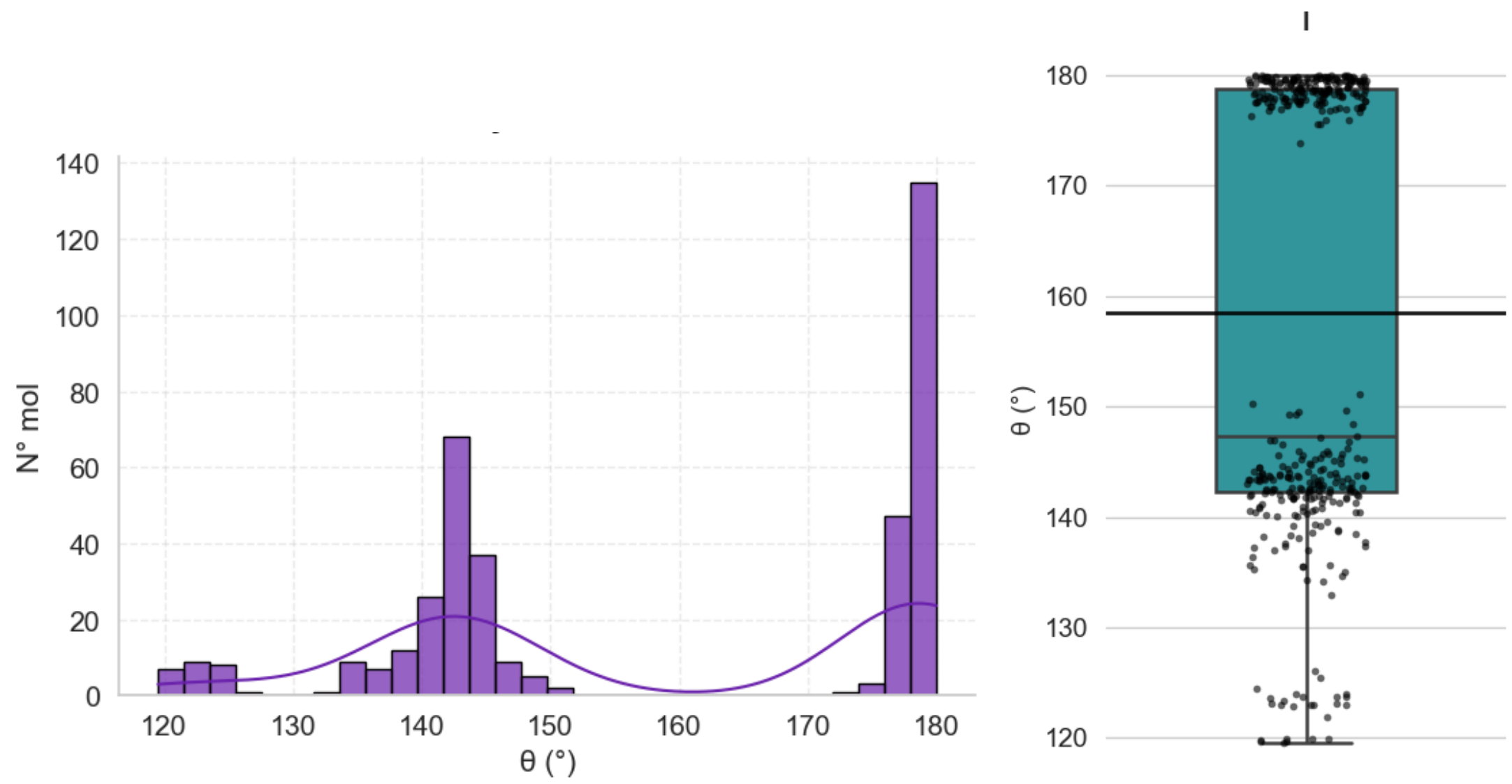

Figure S26: Statistical distribution of the bond angle (θ) of the O=C=O group in the analyzed molecular dataset computed at the GFN2 method. On the left, a histogram with its corresponding density curve; on the right, a boxplot with superimposed individual data points.

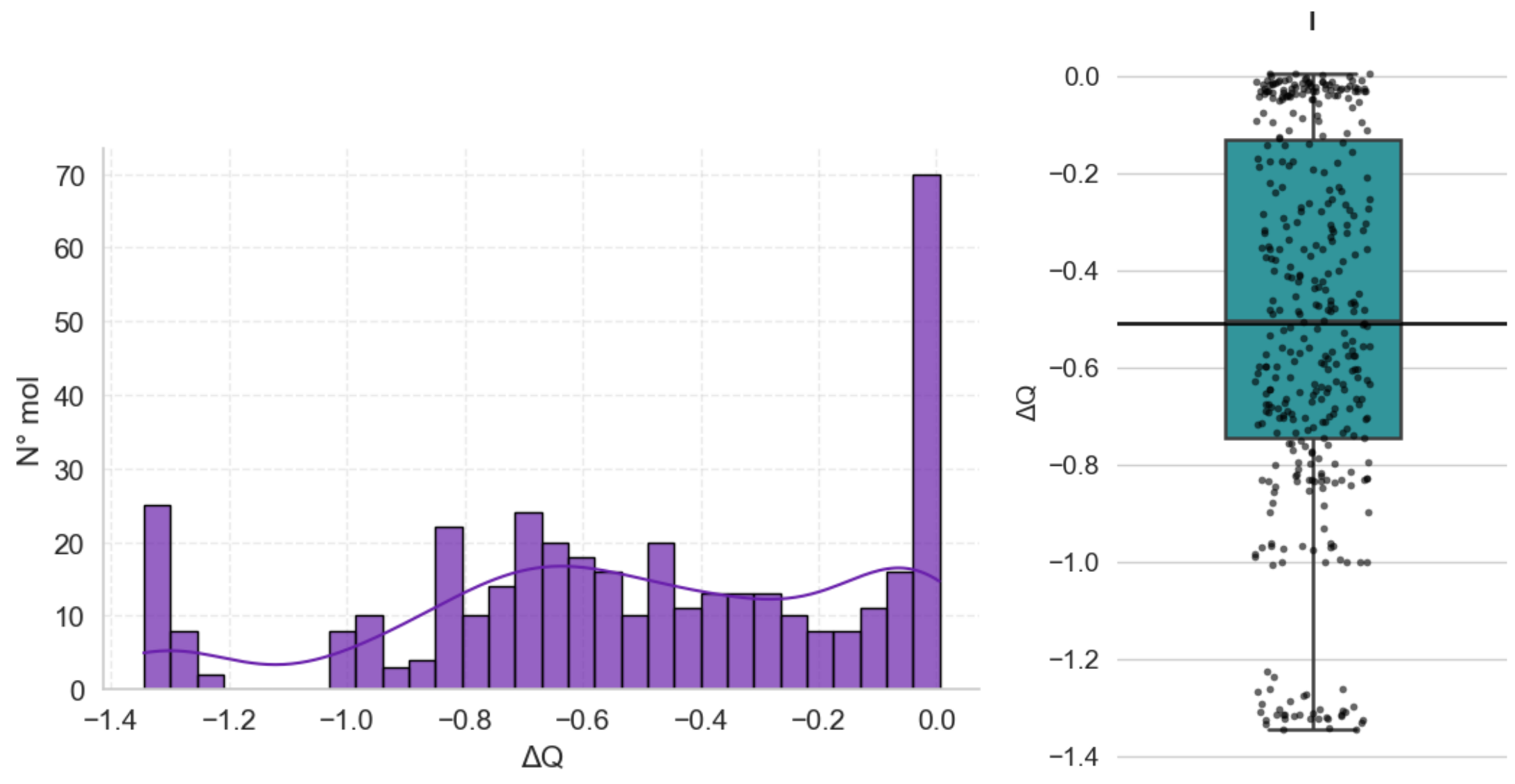

Figure S27: Statistical distribution of the charge transfer (ΔQ) in the analyzed molecular dataset computed at the GFN2 method. On the left, a histogram with its corresponding density curve; on the right, a boxplot with superimposed individual data points.

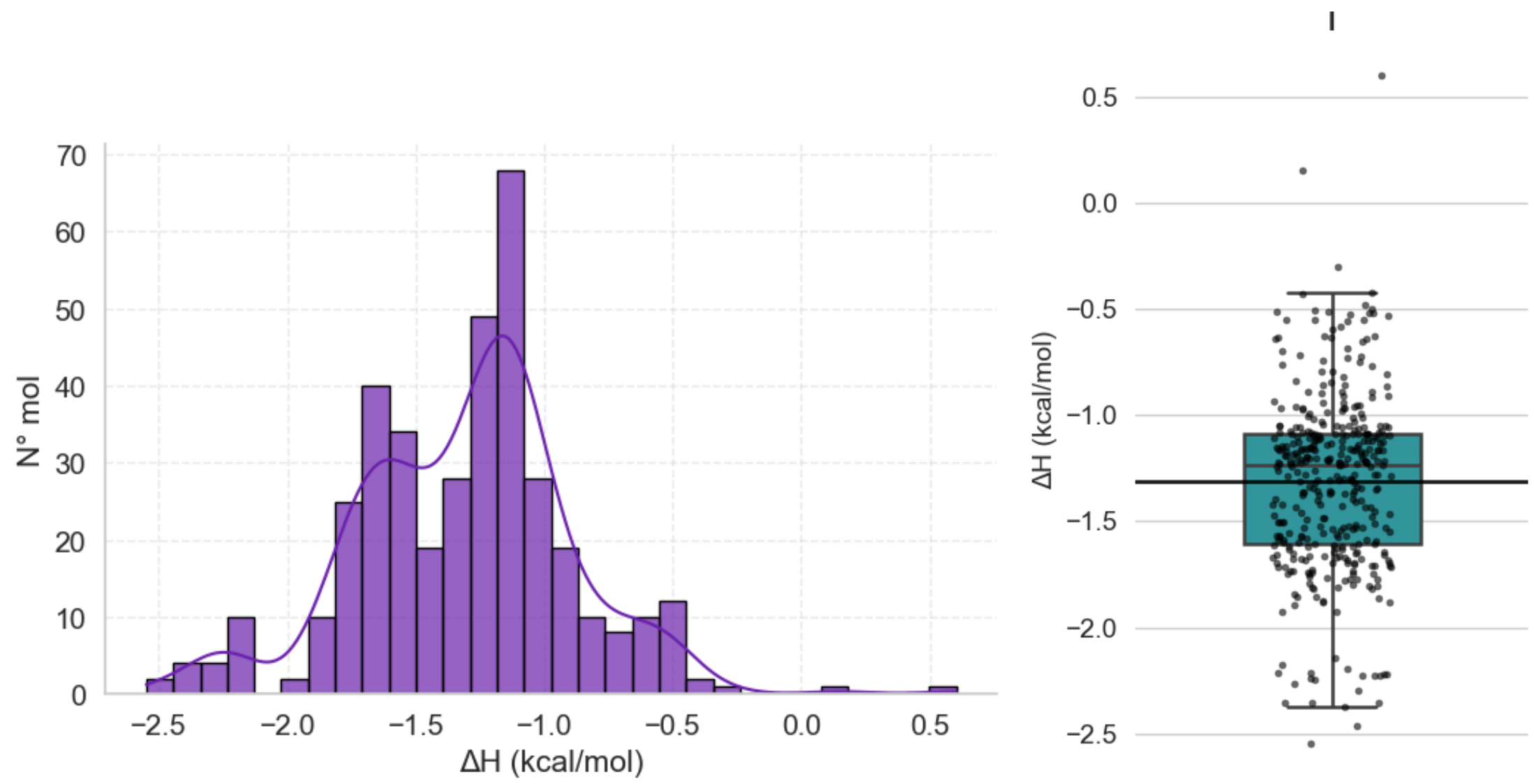

Figure S28: Statistical distribution of the enthalpy (ΔH) in the analyzed molecular dataset computed at the GFN2 method. On the left, a histogram with its corresponding density curve; on the right, a boxplot with superimposed individual data points.

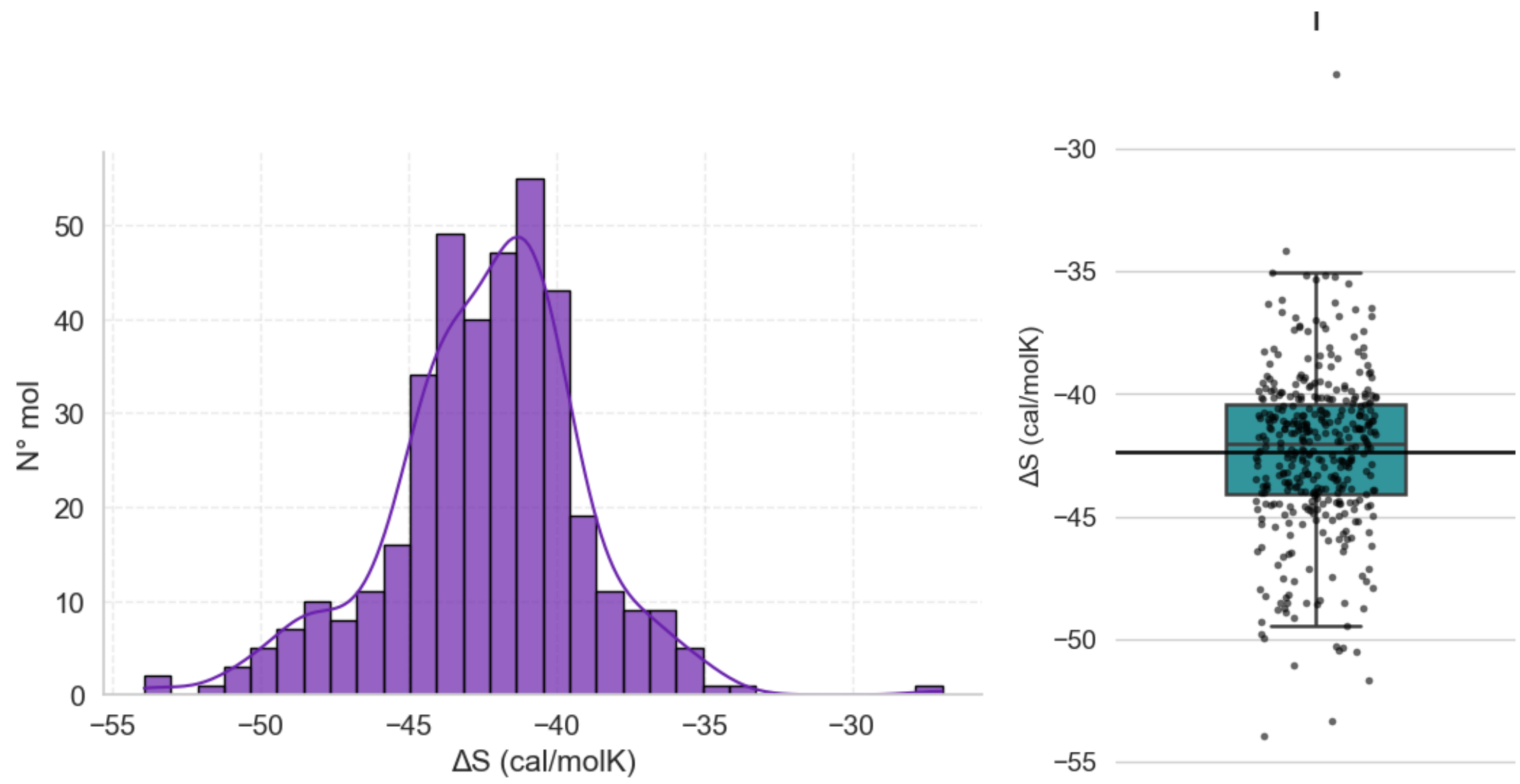

Figure S29: Statistical distribution of the entropy (ΔS) in the analyzed molecular dataset computed at the GFN2 method. On the left, a histogram with its corresponding density curve; on the right, a boxplot with superimposed individual data points.

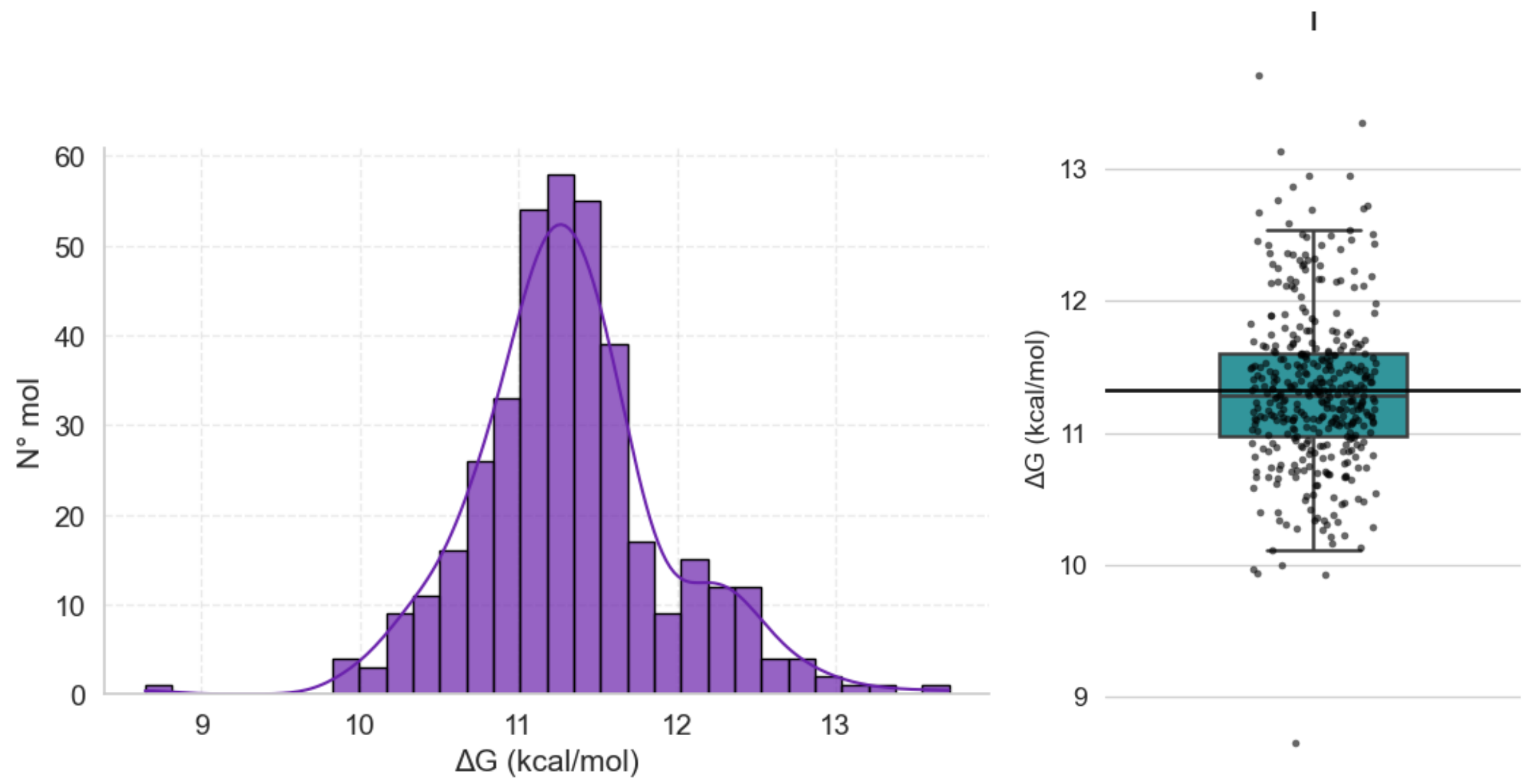

Figure S30: Statistical distribution of the Gibbs free energy (ΔG) in the analyzed molecular dataset computed at the GFN2 method. On the left, a histogram with its corresponding density curve; on the right, a boxplot with superimposed individual data points.

S3. 6 Population distribution analysis and histograms of geometric, thermodynamic, and charge-transfer data calculated using MNDO method

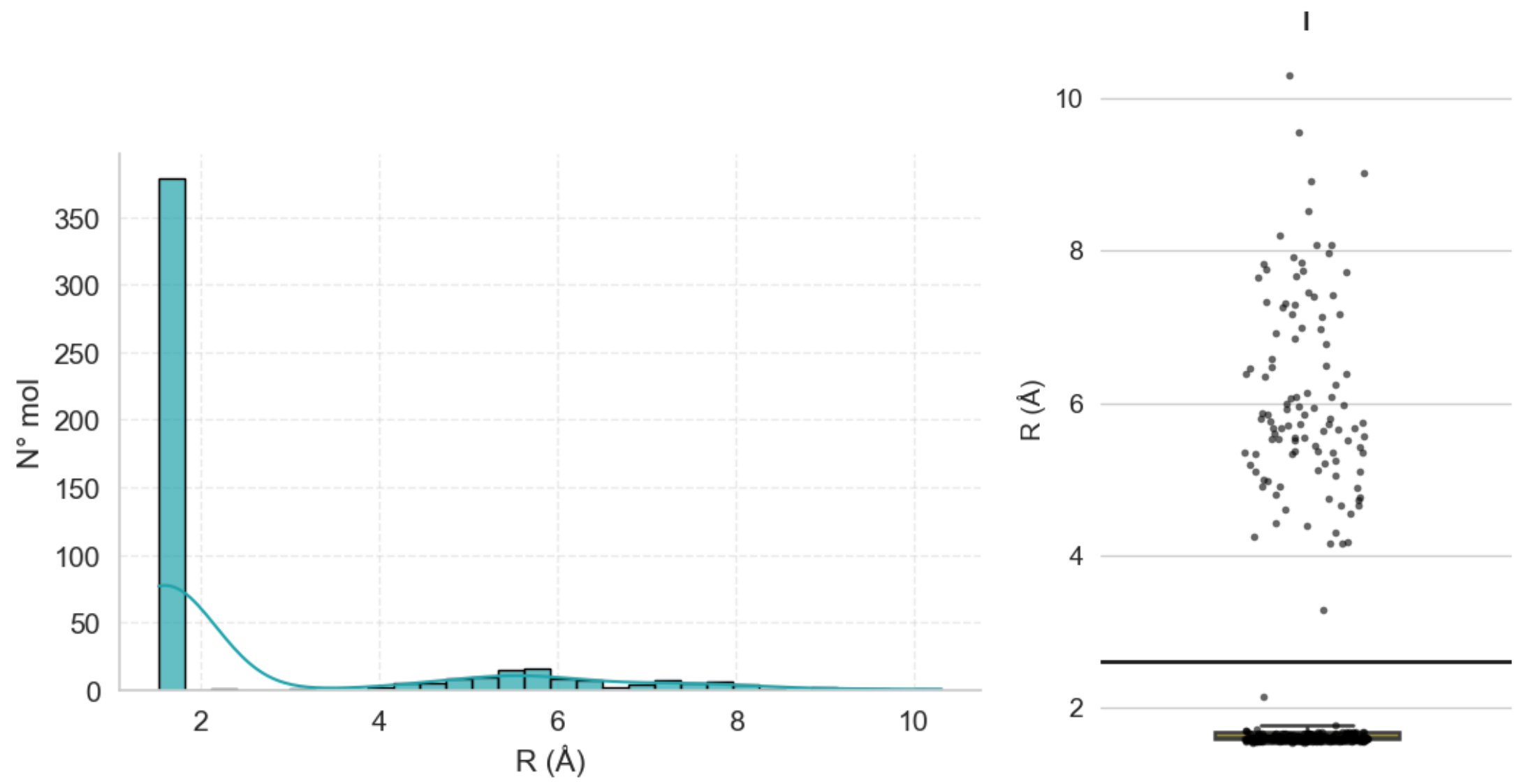

Figure S31: Statistical distribution of the length R (Å) in the analyzed molecular dataset computed at the MNDO method. On the left, a histogram with its corresponding density curve; on the right, a boxplot with superimposed individual data points.

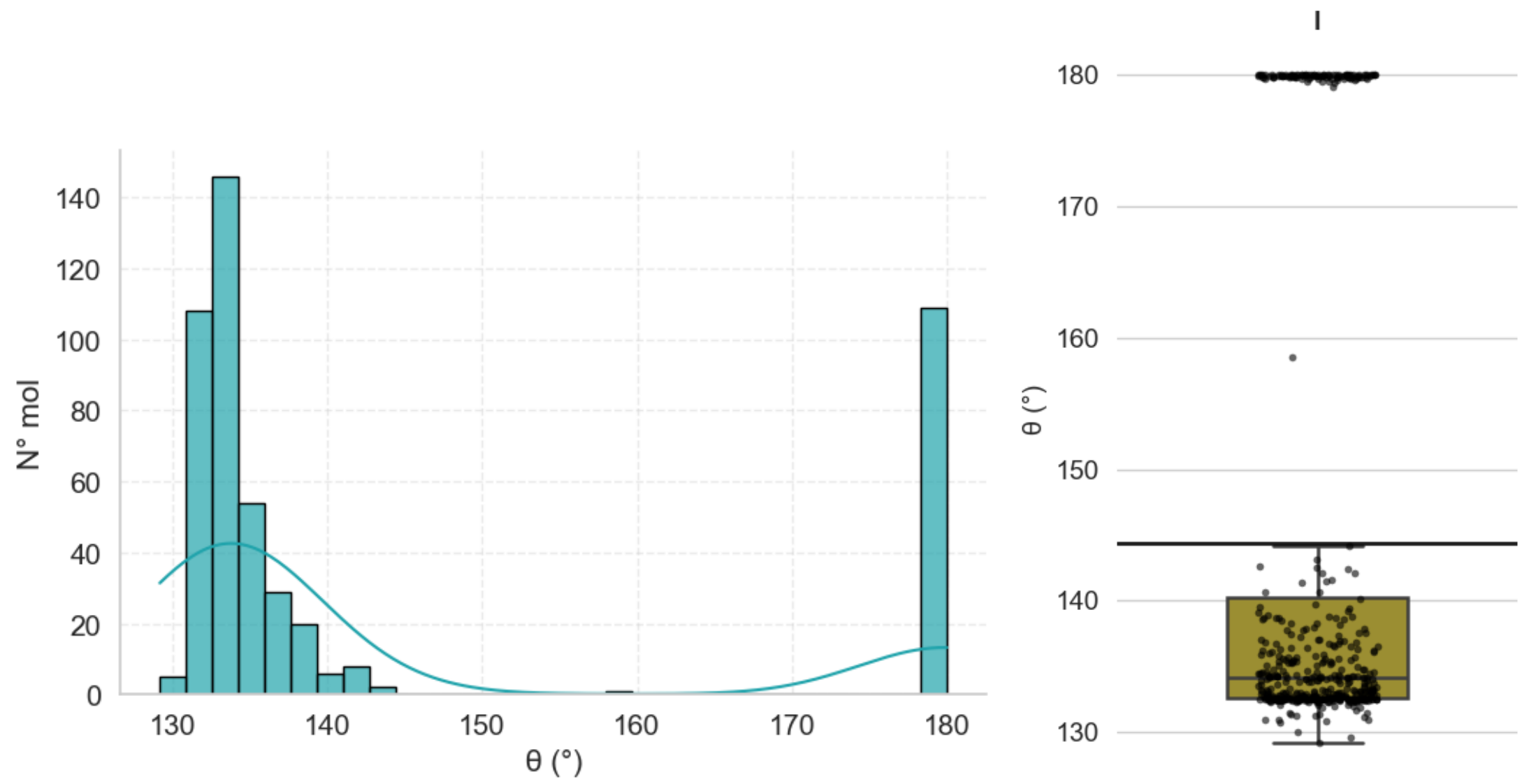

Figure S32: Statistical distribution of the bond angle (θ) of the O=C=O group in the analyzed molecular dataset computed at the MNDO method. On the left, a histogram with its corresponding density curve; on the right, a boxplot with superimposed individual data points.

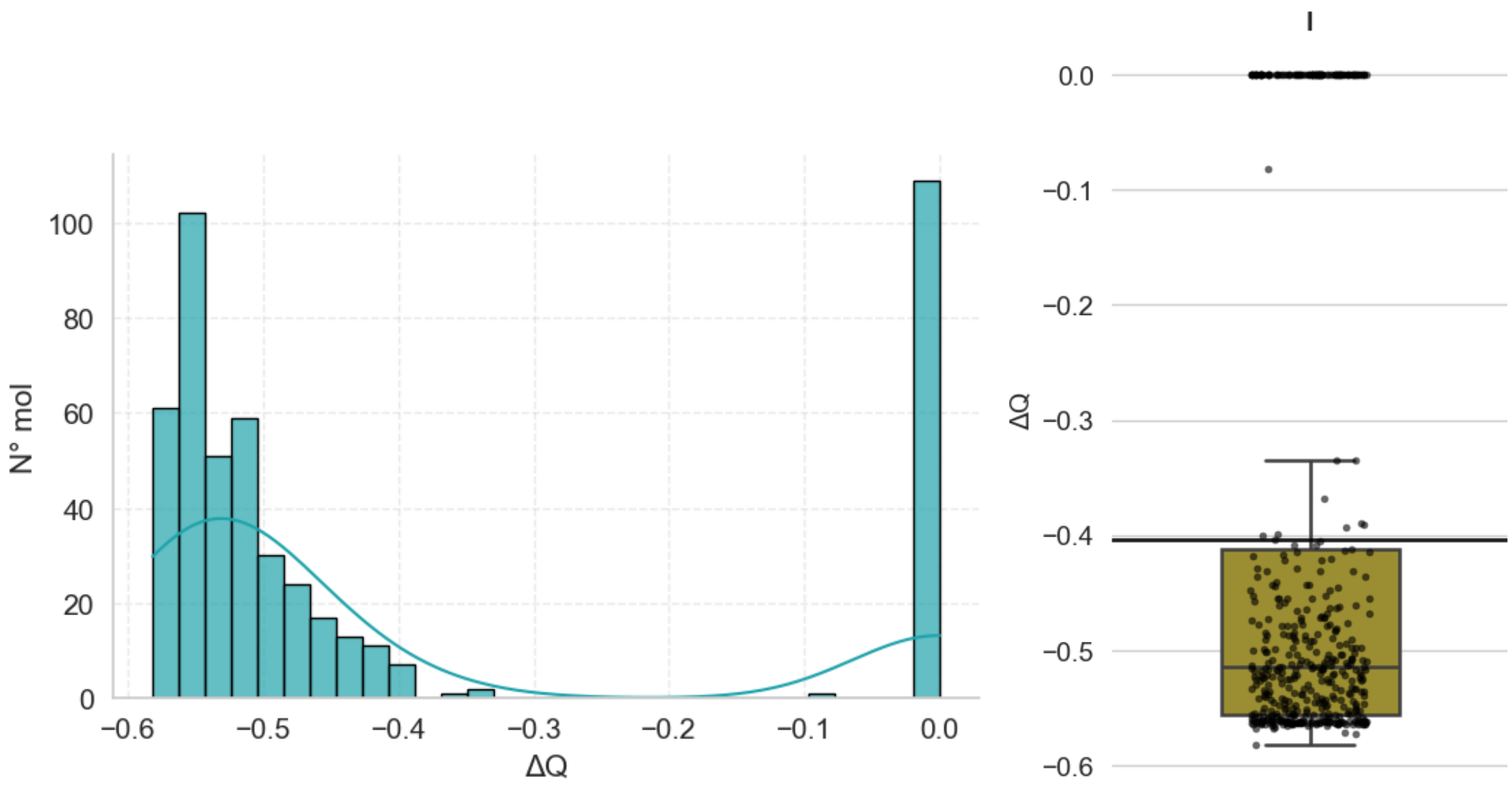

Figure S33: Statistical distribution of the charge transfer (ΔQ) in the analyzed molecular dataset computed at the MNDO method. On the left, a histogram with its corresponding density curve; on the right, a boxplot with superimposed individual data points.

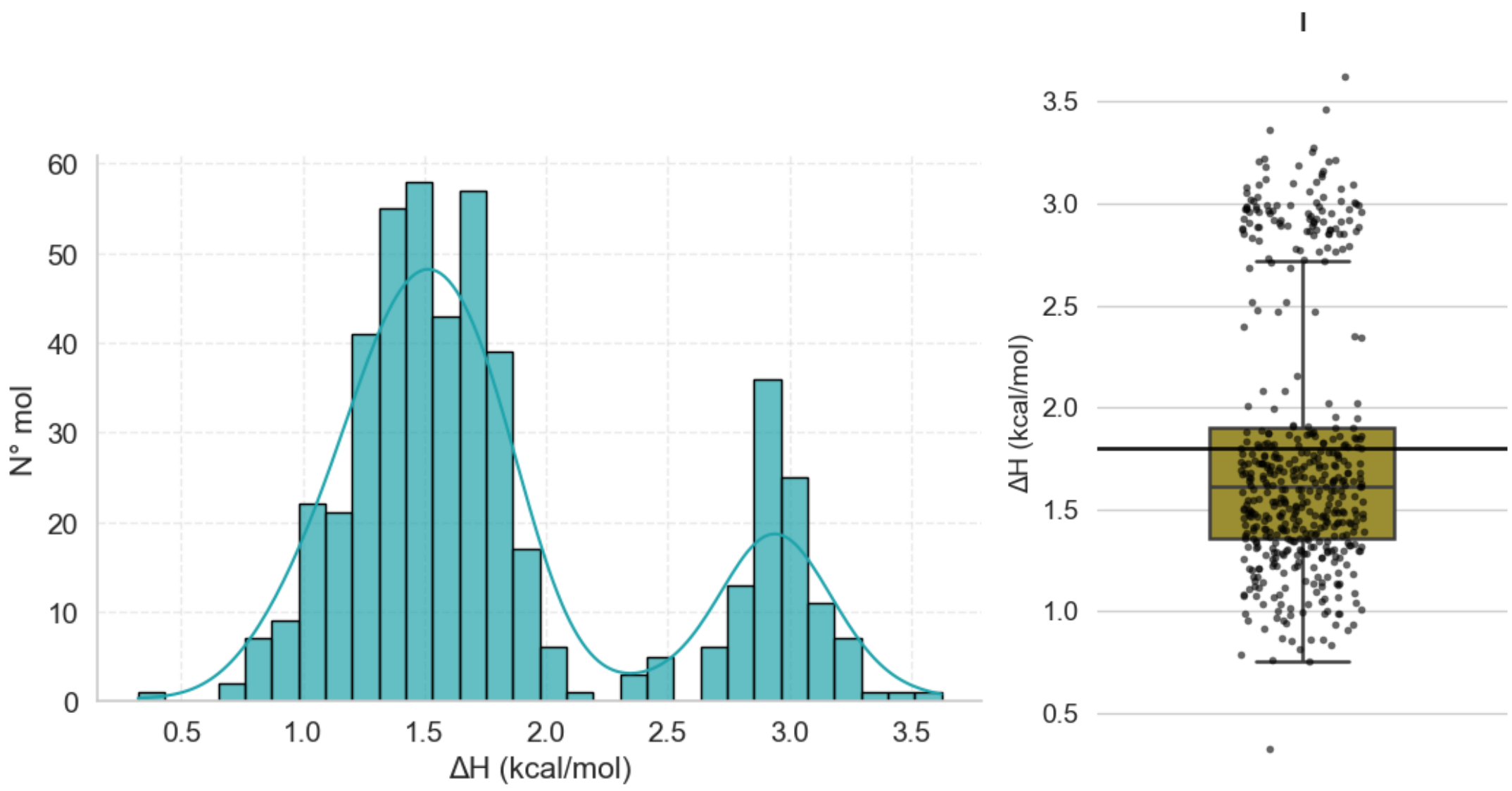

Figure S34: Statistical distribution of the enthalpy (ΔH) in the analyzed molecular dataset computed at the MNDO method. On the left, a histogram with its corresponding density curve; on the right, a boxplot with superimposed individual data points.

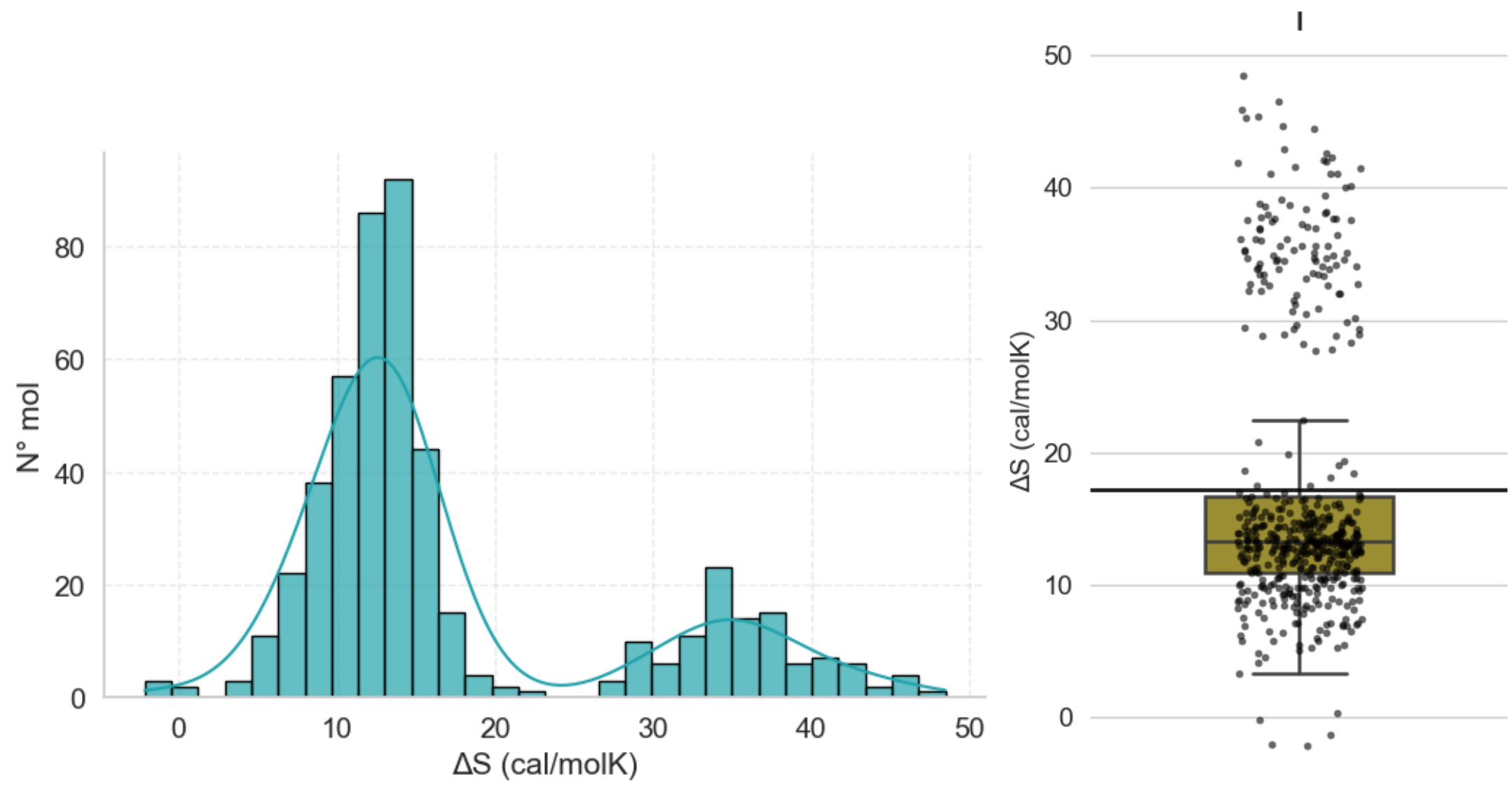

Figure S35: Statistical distribution of the entropy (ΔS) in the analyzed molecular dataset computed at the MNDO method. On the left, a histogram with its corresponding density curve; on the right, a boxplot with superimposed individual data points.

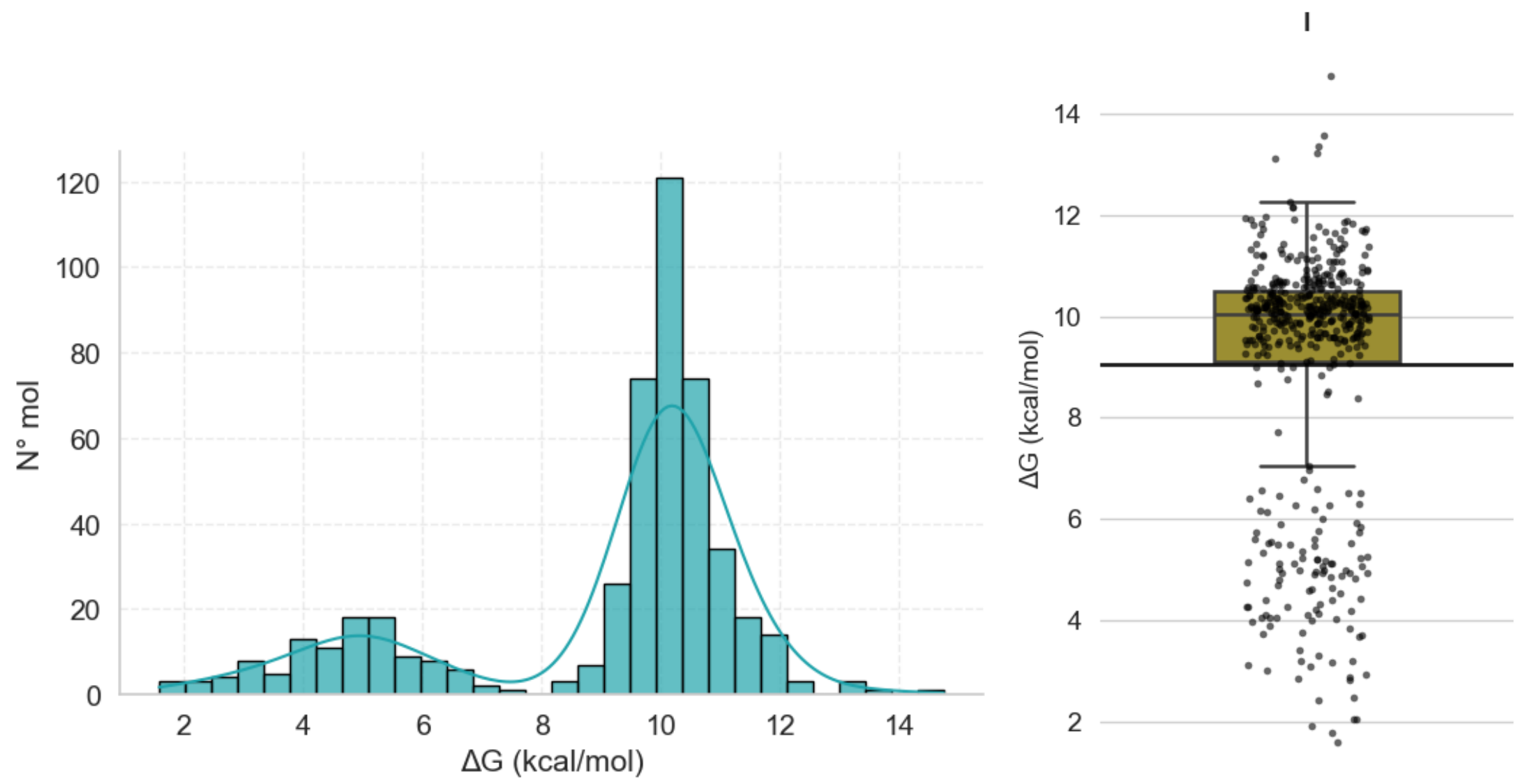

Figure S36: Statistical distribution of the Gibbs free energy (ΔG) in the analyzed molecular dataset computed at the MNDO method. On the left, a histogram with its corresponding density curve; on the right, a boxplot with superimposed individual data points.

S3. 7 Population distribution analysis and histograms of geometric, thermodynamic, and charge-transfer data calculated using MNDOD method

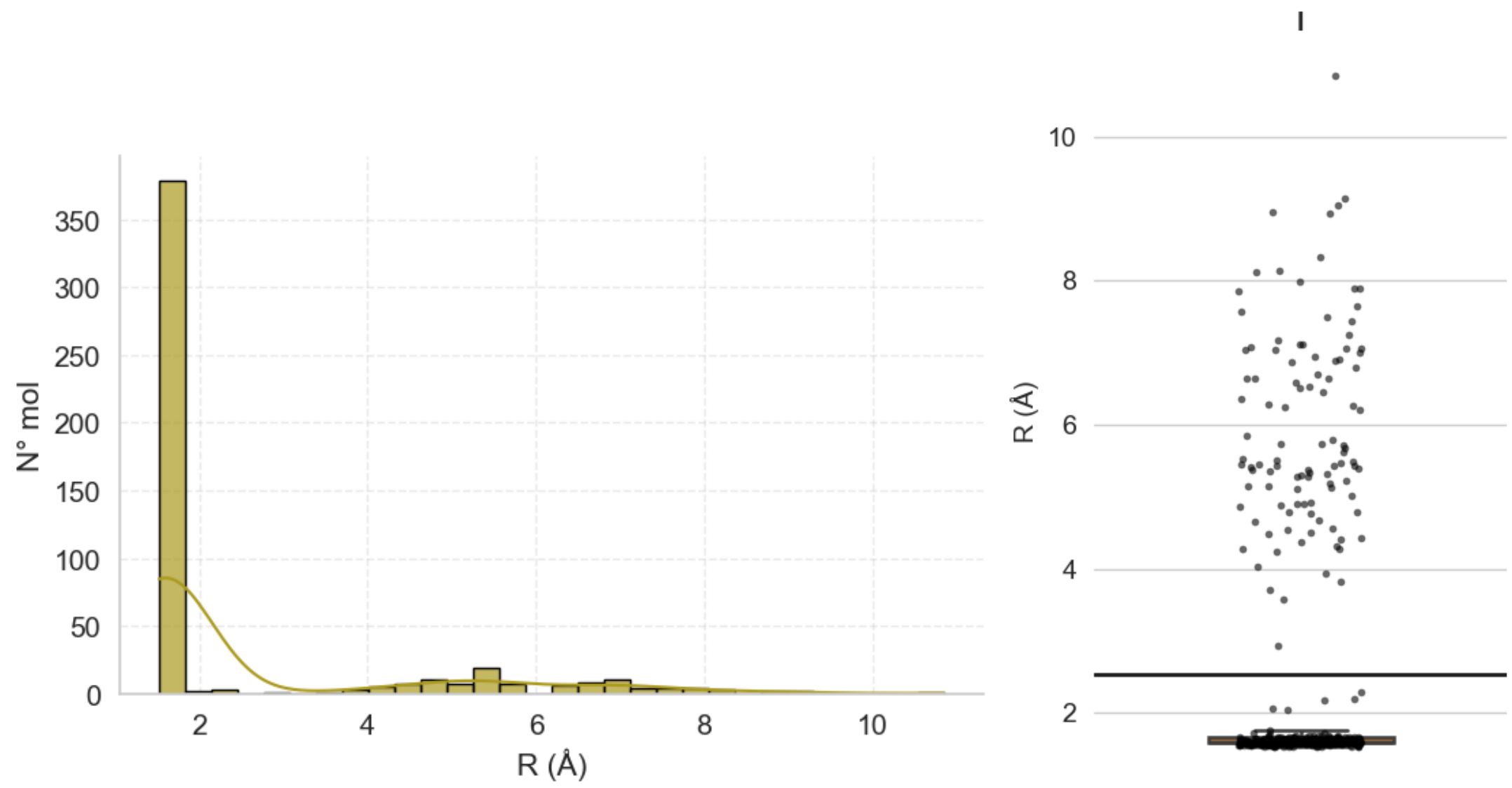

Figure S37: Statistical distribution of the length R (Å) in the analyzed molecular dataset computed at the MNDOD method. On the left, a histogram with its corresponding density curve; on the right, a boxplot with superimposed individual data points.

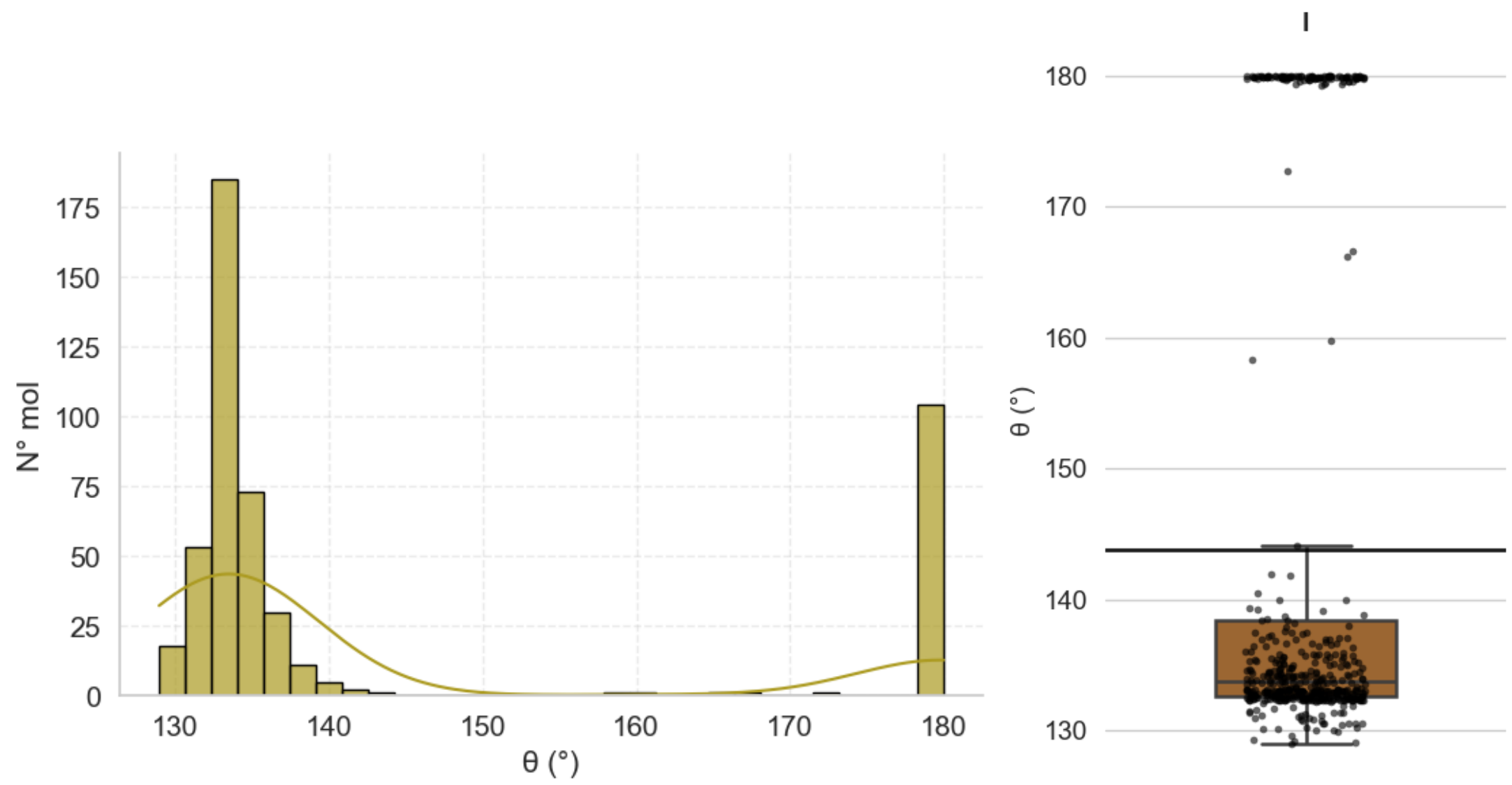

Figure S38: Statistical distribution of the bond angle (θ) of the O=C=O group in the analyzed molecular dataset computed at the MNDOD method. On the left, a histogram with its corresponding density curve; on the right, a boxplot with superimposed individual data points.

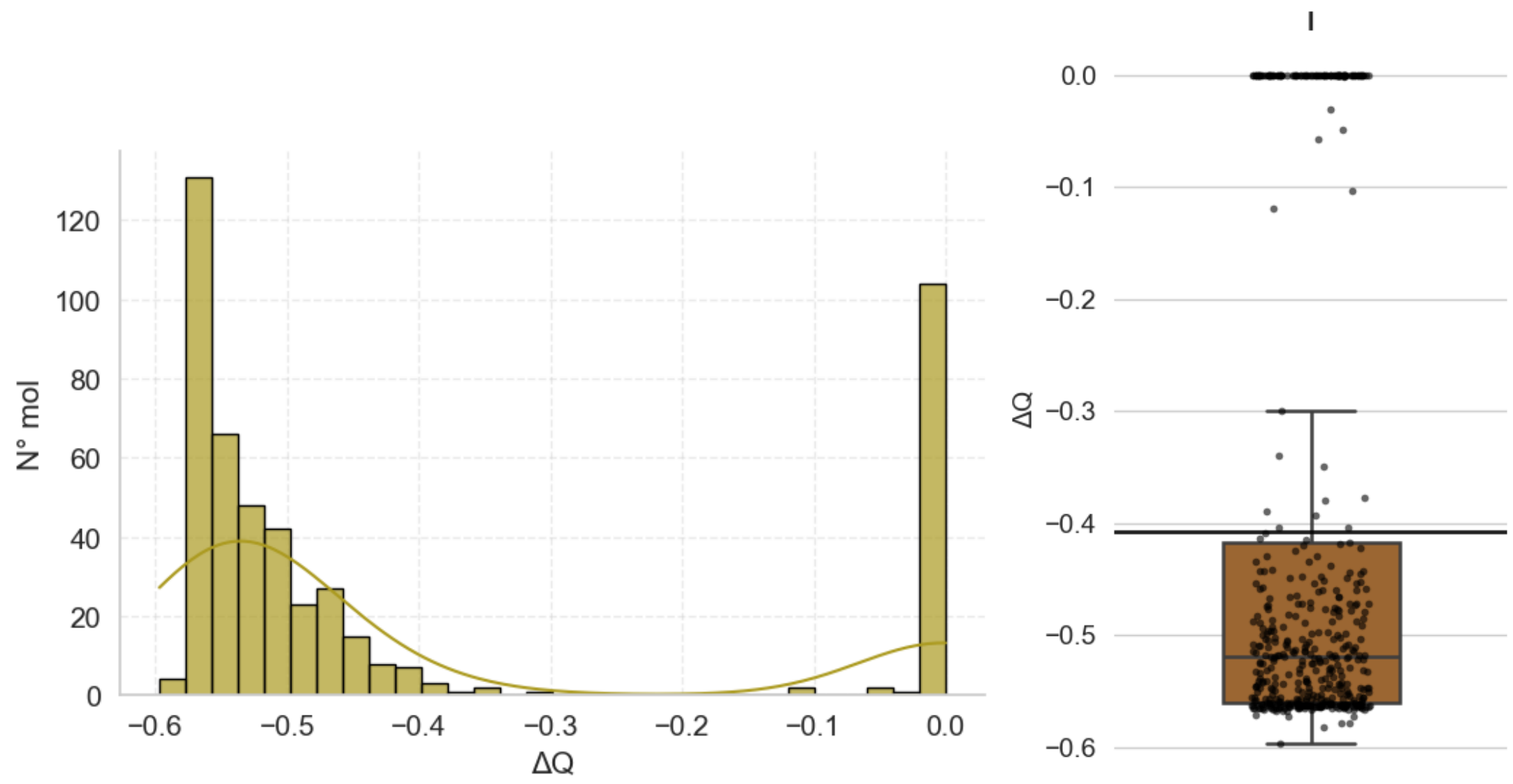

Figure S39: Statistical distribution of the charge transfer (ΔQ) in the analyzed molecular dataset computed at the MNDOD method. On the left, a histogram with its corresponding density curve; on the right, a boxplot with superimposed individual data points.

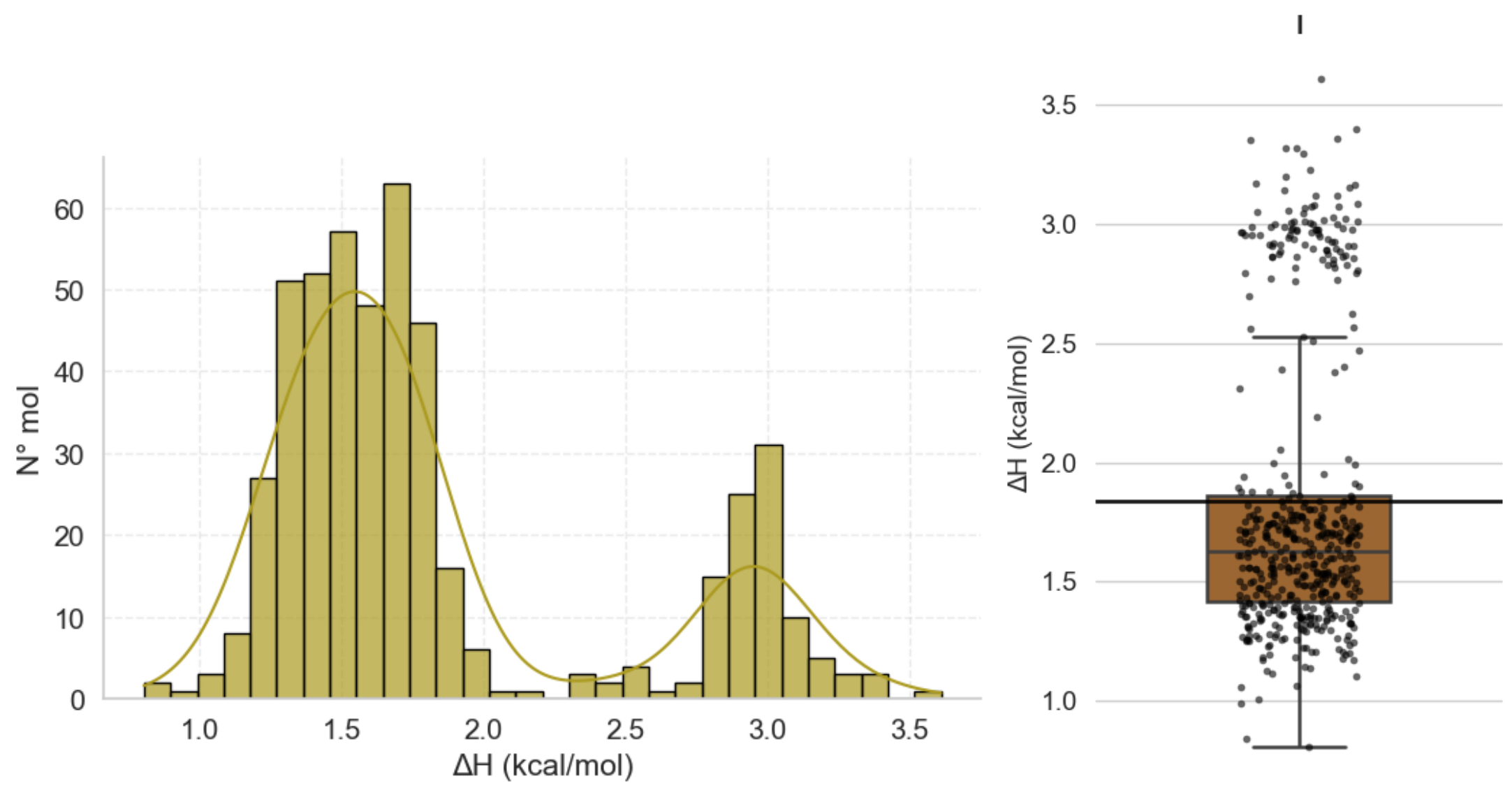

Figure S40: Statistical distribution of the enthalpy (ΔH) in the analyzed molecular dataset computed at the MNDOD method. On the left, a histogram with its corresponding density curve; on the right, a boxplot with superimposed individual data points.

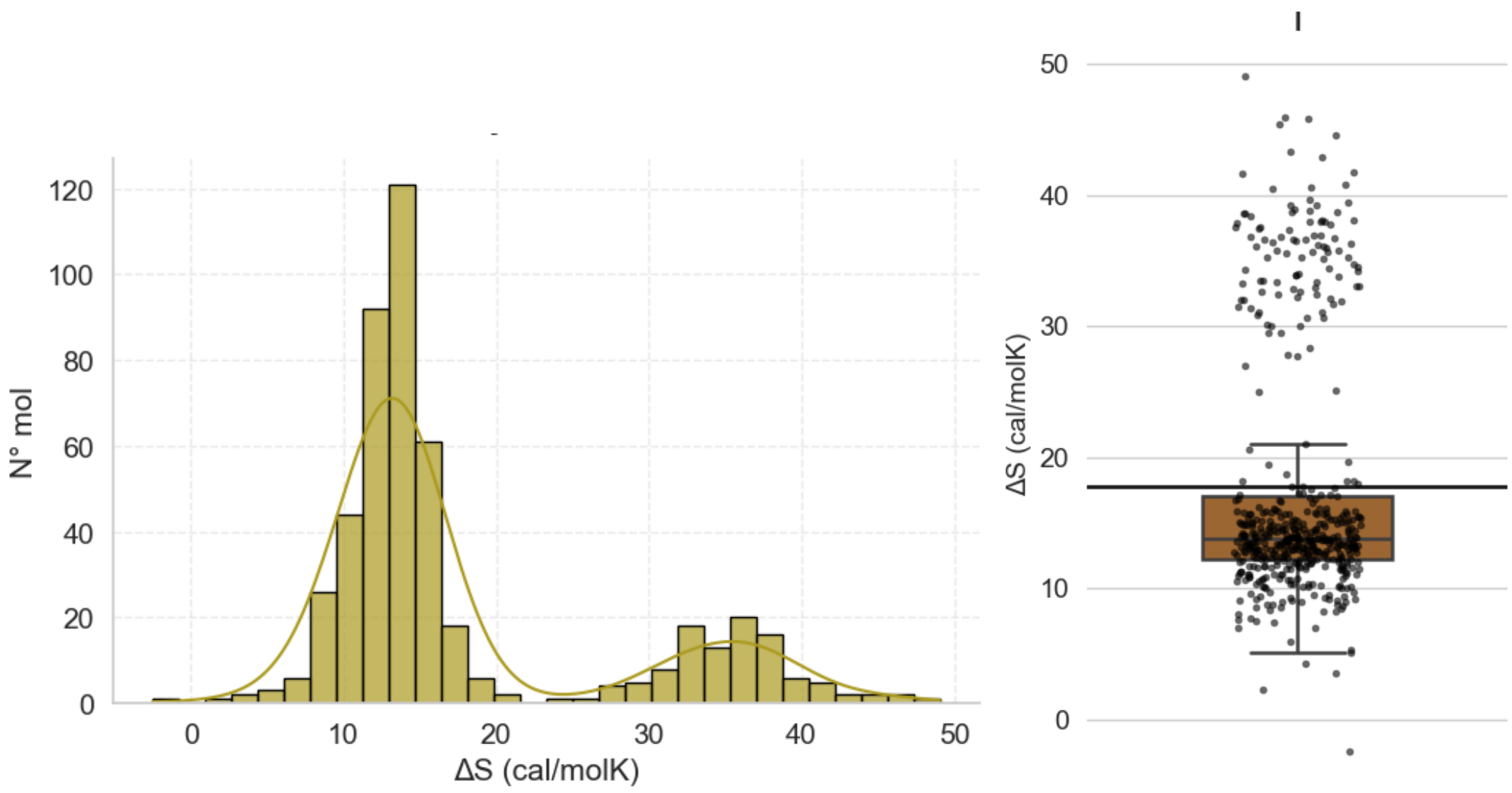

Figure S41: Statistical distribution of the entropy (ΔS) in the analyzed molecular dataset computed at the MNDOD method. On the left, a histogram with its corresponding density curve; on the right, a boxplot with superimposed individual data points.

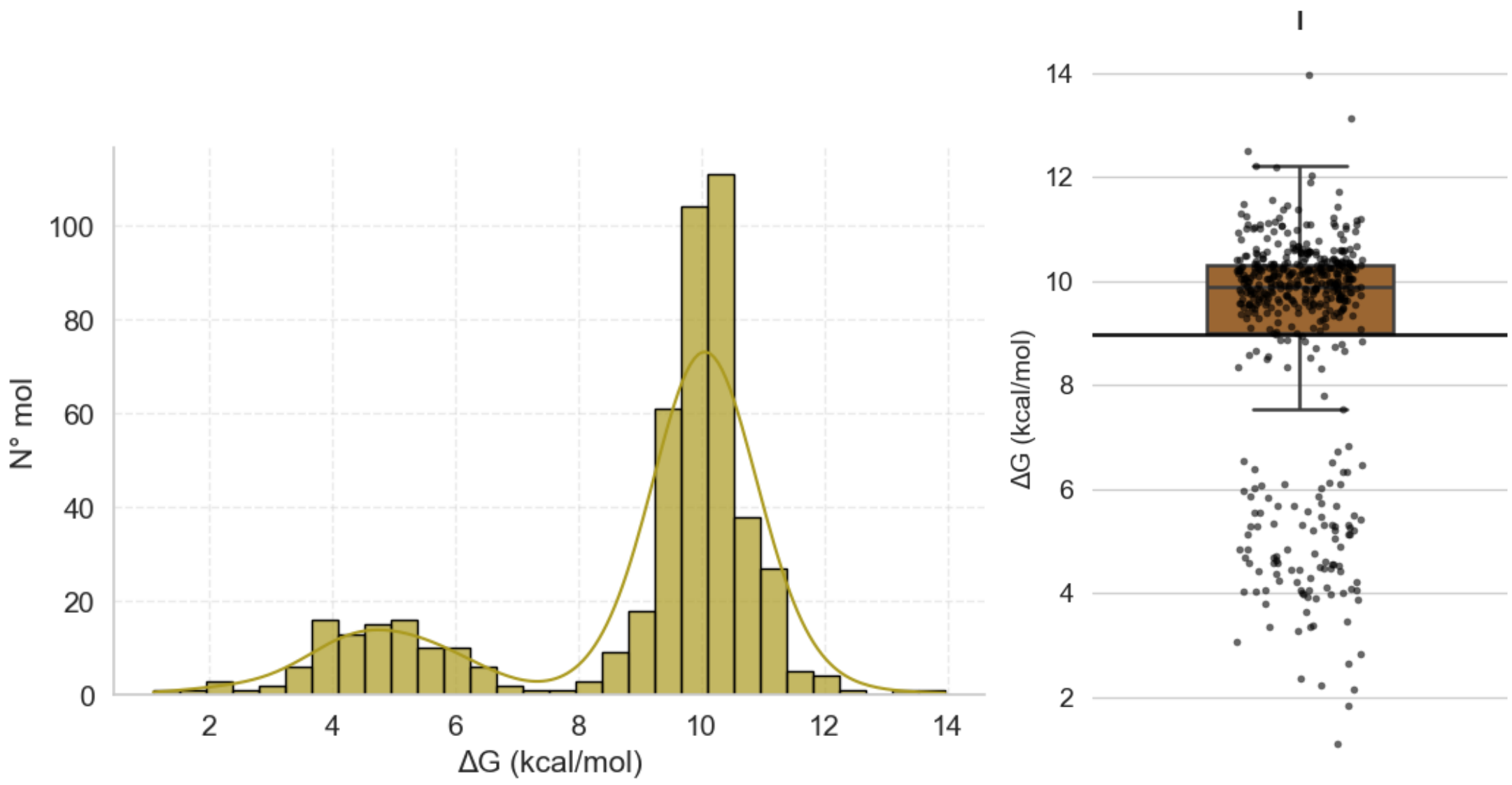

Figure S42: Statistical distribution of the Gibbs free energy (ΔG) in the analyzed molecular dataset computed at the MNDOD method. On the left, a histogram with its corresponding density curve; on the right, a boxplot with superimposed individual data points.

S3. 8 Population distribution analysis and histograms of geometric, thermodynamic, and charge-transfer data calculated using PM3 method

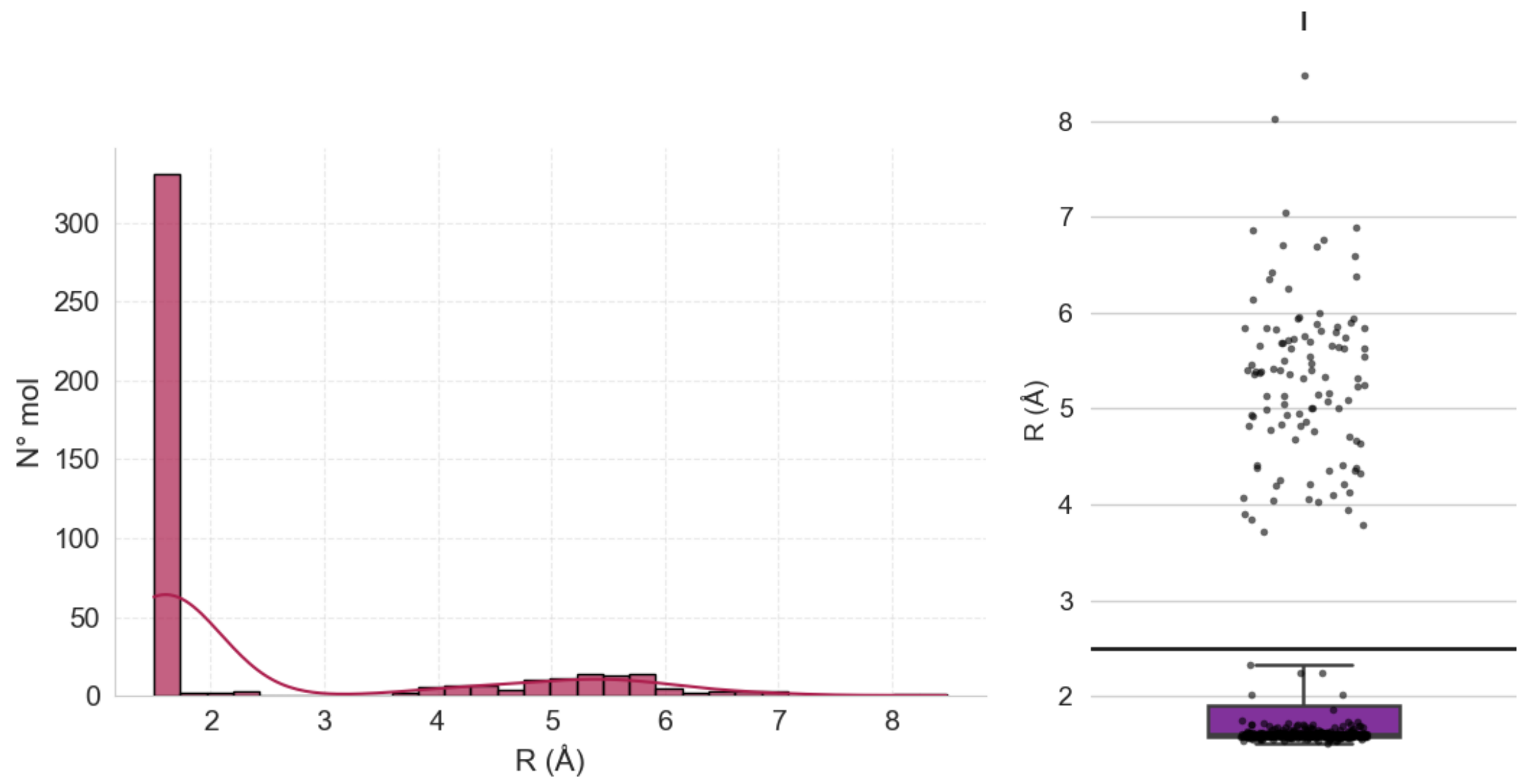

Figure S43: Statistical distribution of the length R (Å) in the analyzed molecular dataset computed at the PM3 method. On the left, a histogram with its corresponding density curve; on the right, a boxplot with superimposed individual data points.

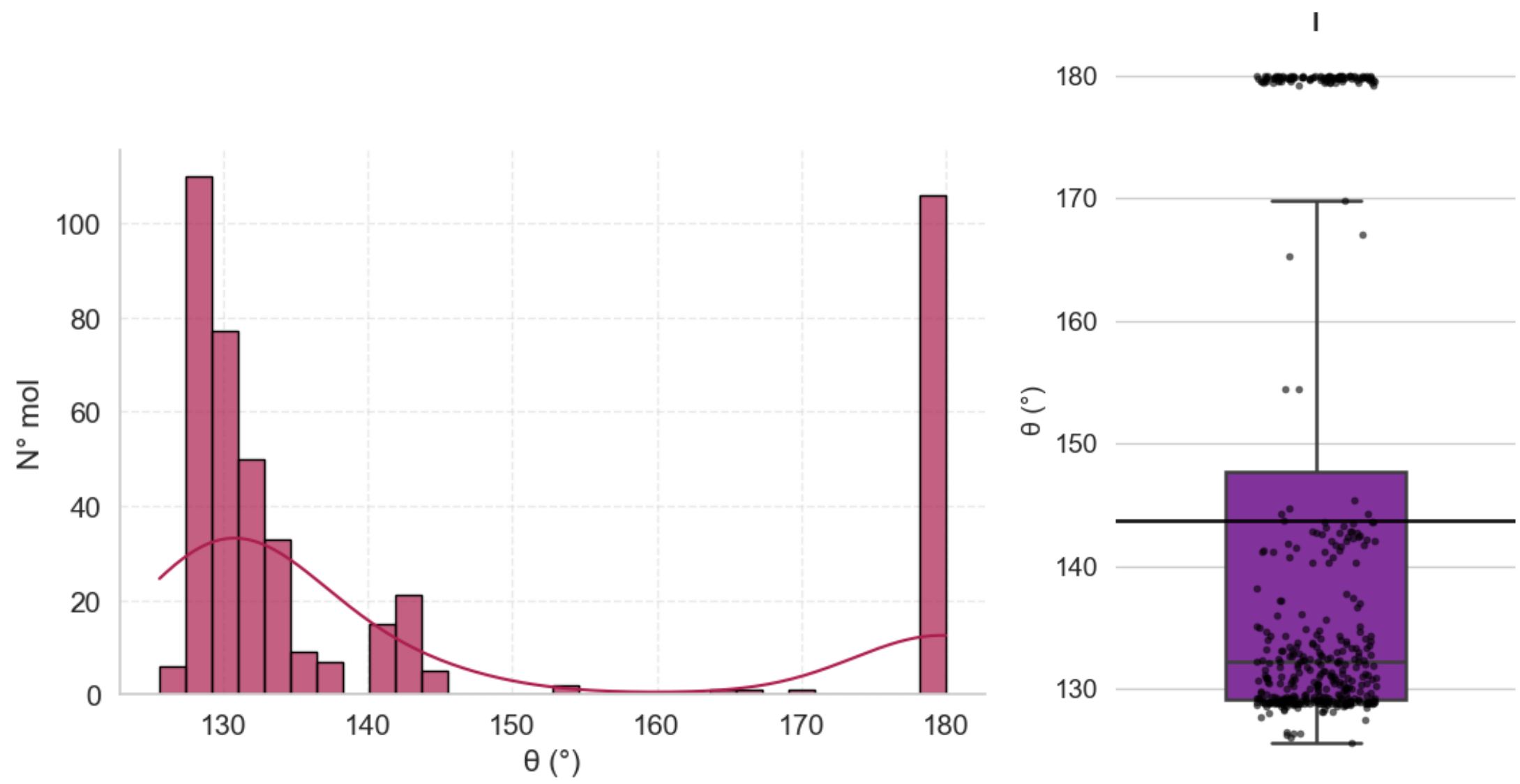

Figure S44: Statistical distribution of the bond angle (θ) of the O=C=O group in the analyzed molecular dataset computed at the PM3 method. On the left, a histogram with its corresponding density curve; on the right, a boxplot with superimposed individual data points.

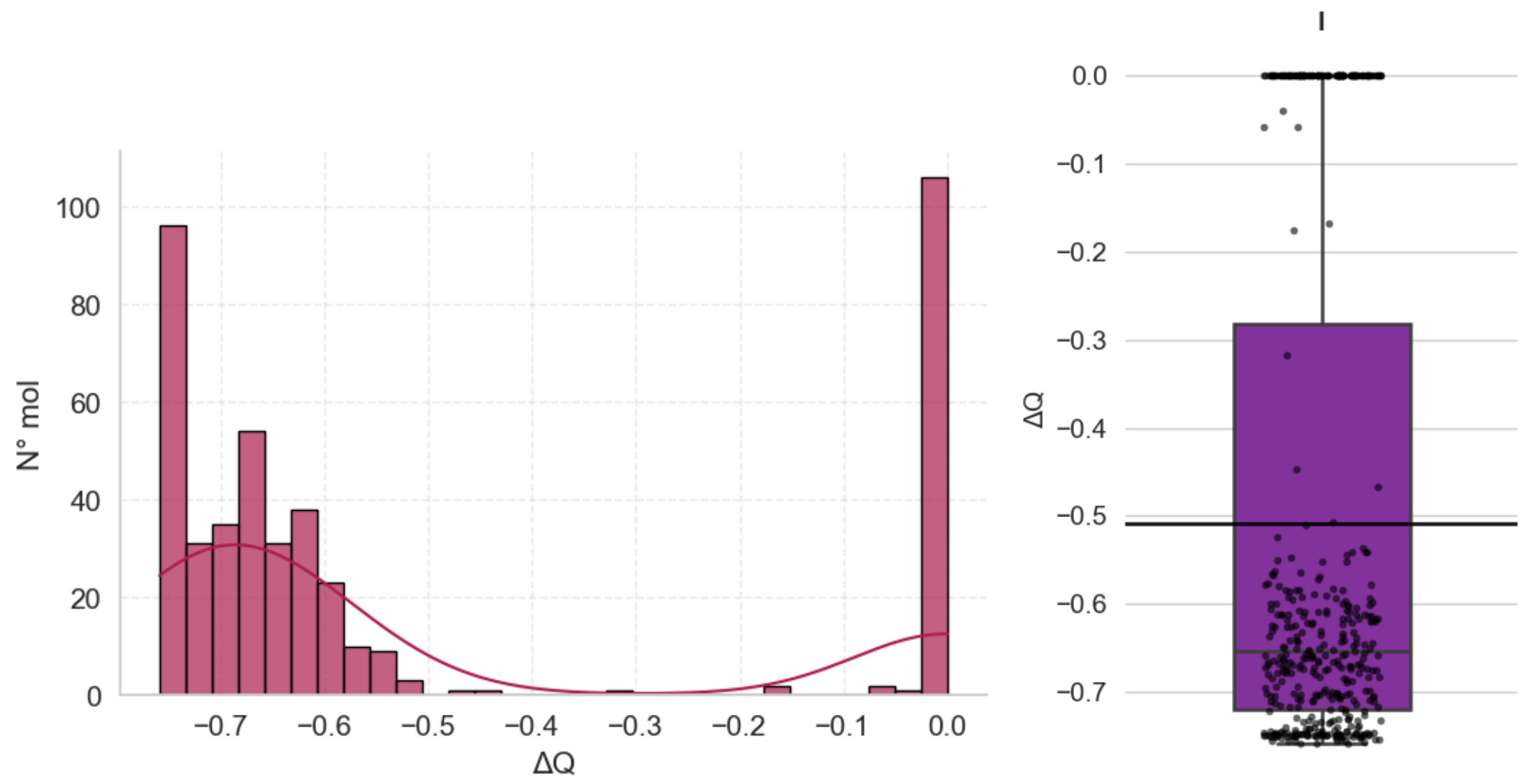

Figure S45: Statistical distribution of the charge transfer (ΔQ) in the analyzed molecular dataset computed at the PM3 method. On the left, a histogram with its corresponding density curve; on the right, a boxplot with superimposed individual data points.

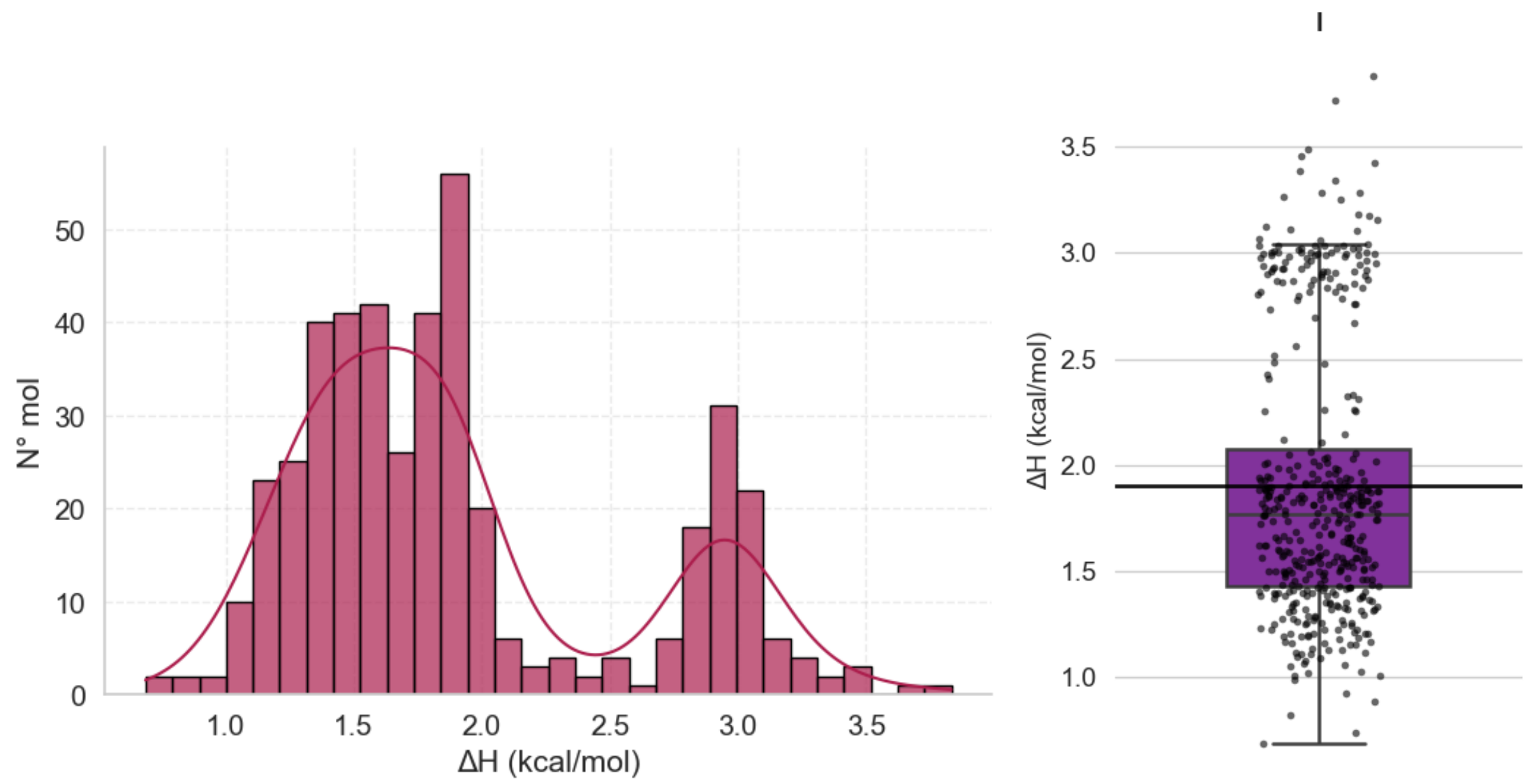

Figure S46: Statistical distribution of the enthalpy (ΔH) in the analyzed molecular dataset computed at the PM3 method. On the left, a histogram with its corresponding density curve; on the right, a boxplot with superimposed individual data points.

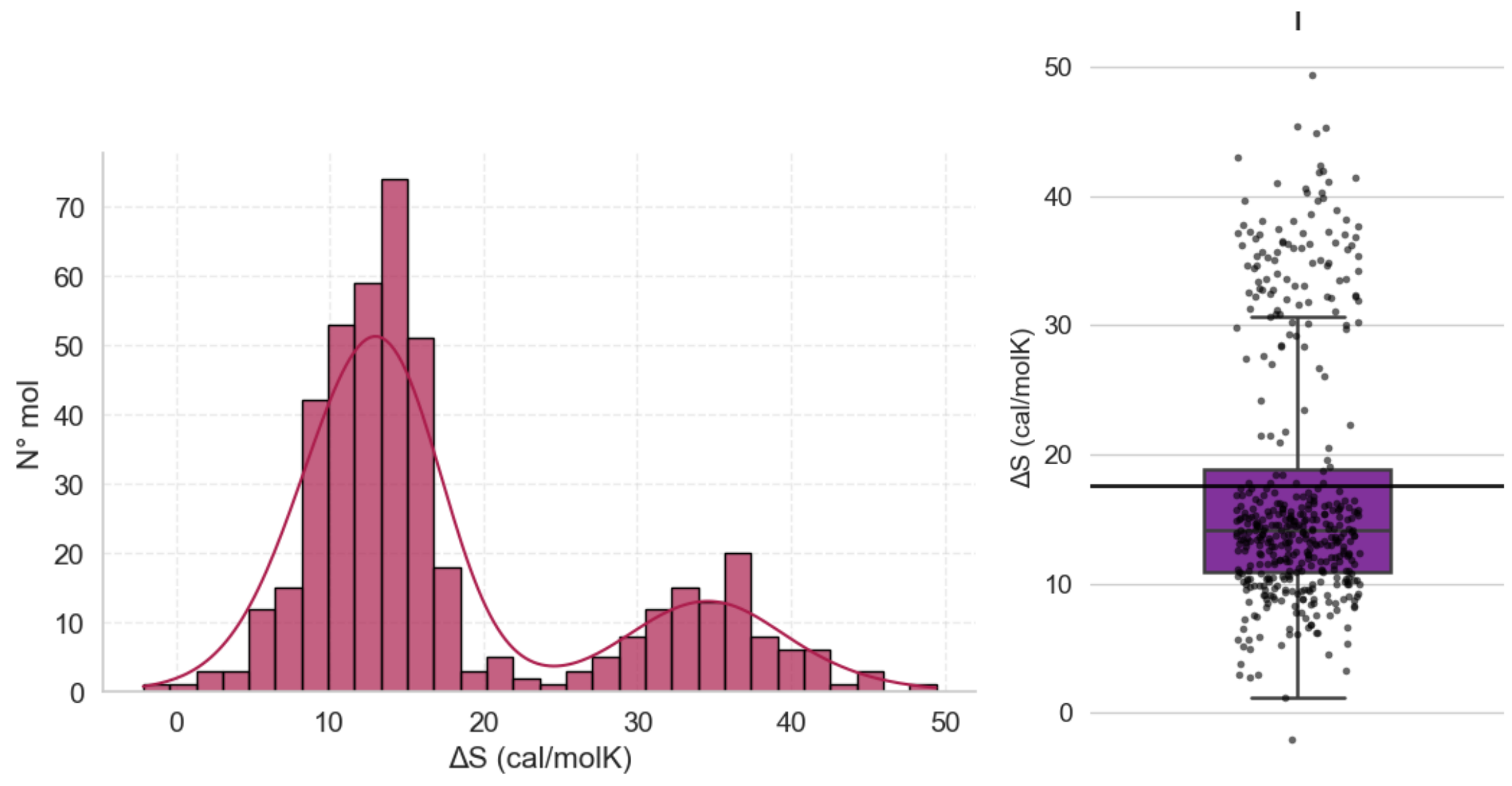

Figure S47: Statistical distribution of the entropy (ΔS) in the analyzed molecular dataset computed at the PM3 method. On the left, a histogram with its corresponding density curve; on the right, a boxplot with superimposed individual data points.

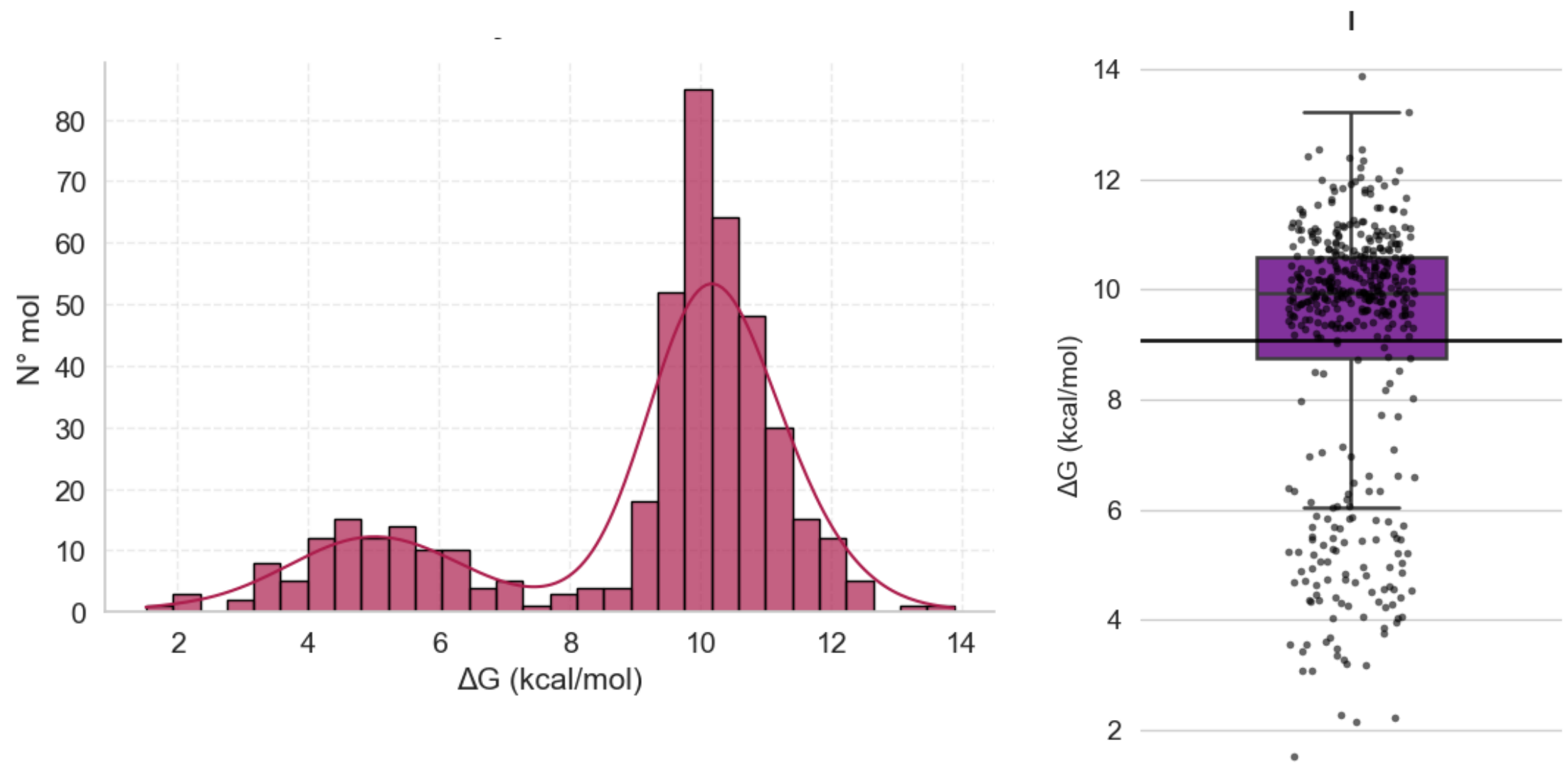

Figure S48: Statistical distribution of the Gibbs free energy (ΔG) in the analyzed molecular dataset computed at the PM3 method. On the left, a histogram with its corresponding density curve; on the right, a boxplot with superimposed individual data points.

S3. 9 Population distribution analysis and histograms of geometric, thermodynamic, and charge-transfer data calculated using PM6 method

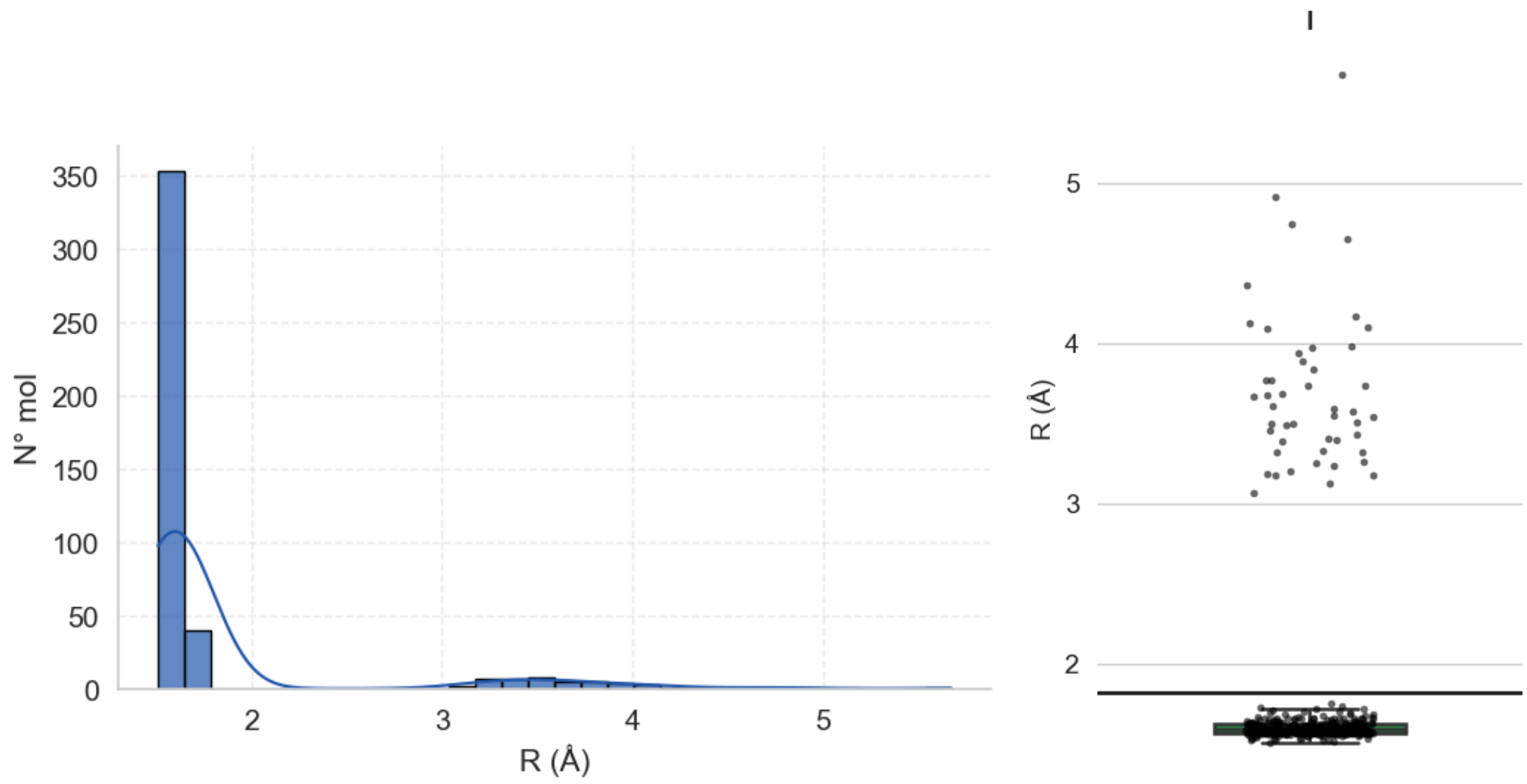

Figure S49: Statistical distribution of the length R (Å) in the analyzed molecular dataset computed at the PM6 method. On the left, a histogram with its corresponding density curve; on the right, a boxplot with superimposed individual data points.

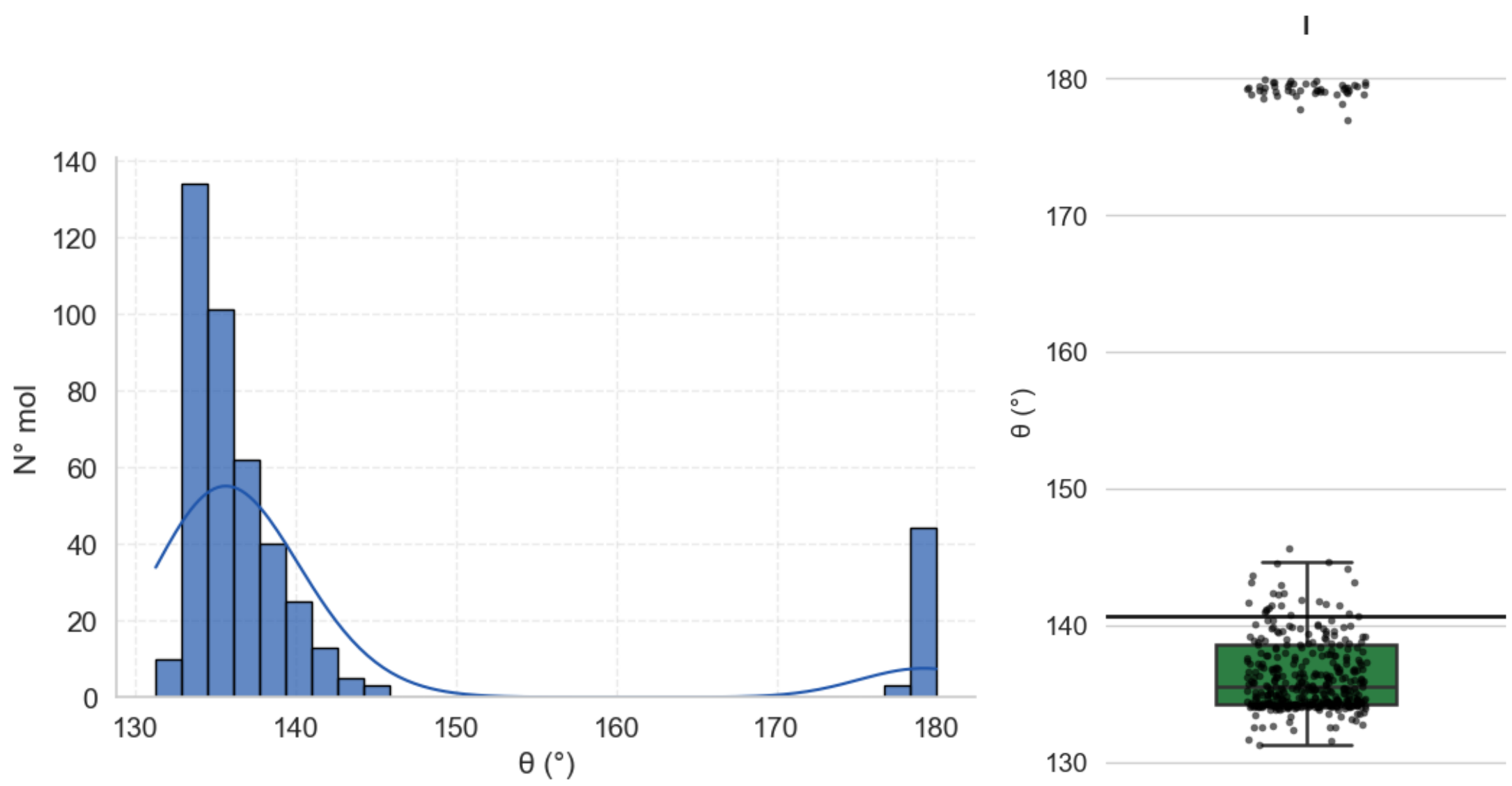

Figure S50: Statistical distribution of the bond angle (θ) of the O=C=O group in the analyzed molecular dataset computed at the PM6 method. On the left, a histogram with its corresponding density curve; on the right, a boxplot with superimposed individual data points.

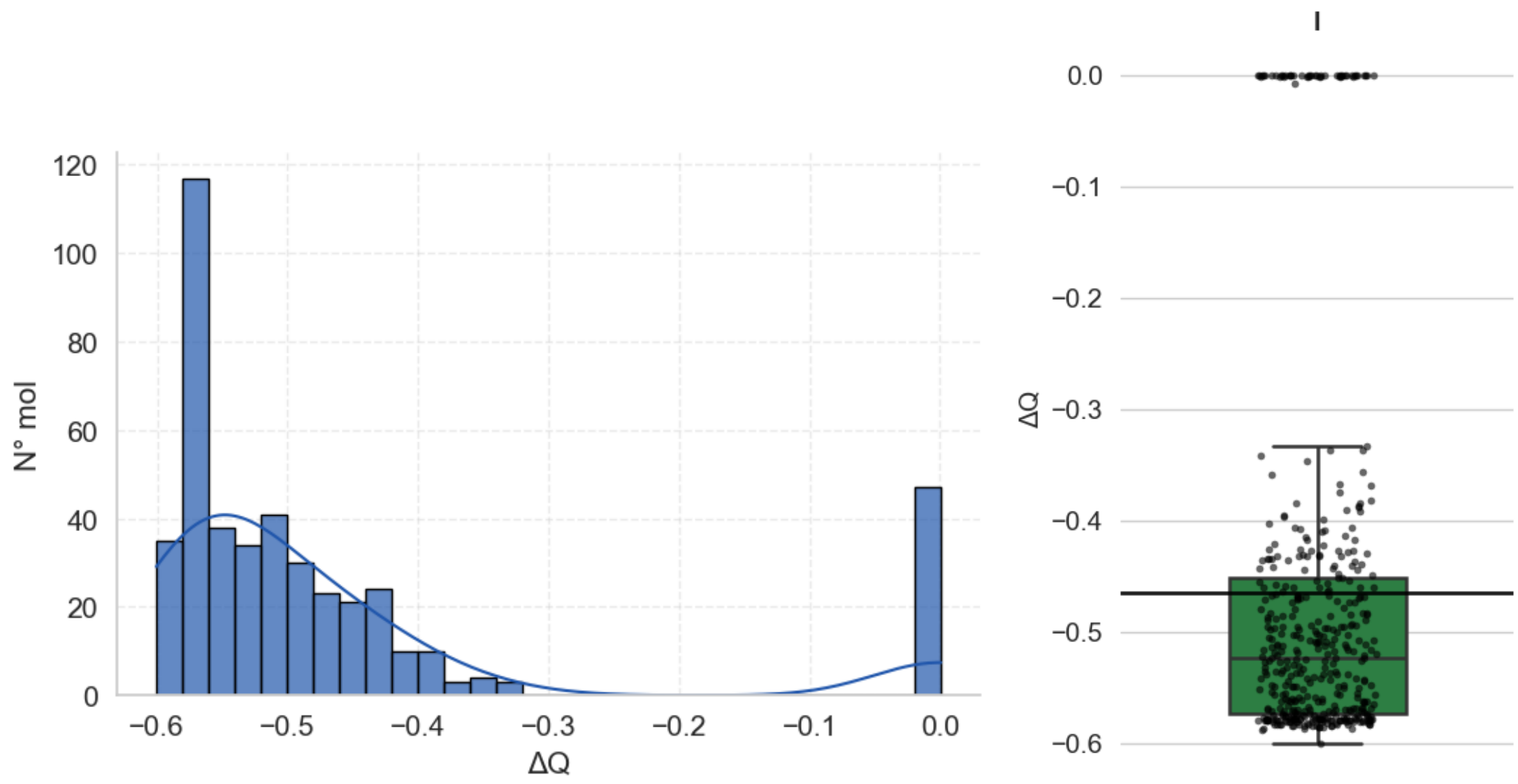

Figure S51: Statistical distribution of the charge transfer (ΔQ) in the analyzed molecular dataset computed at the PM6 method. On the left, a histogram with its corresponding density curve; on the right, a boxplot with superimposed individual data points.

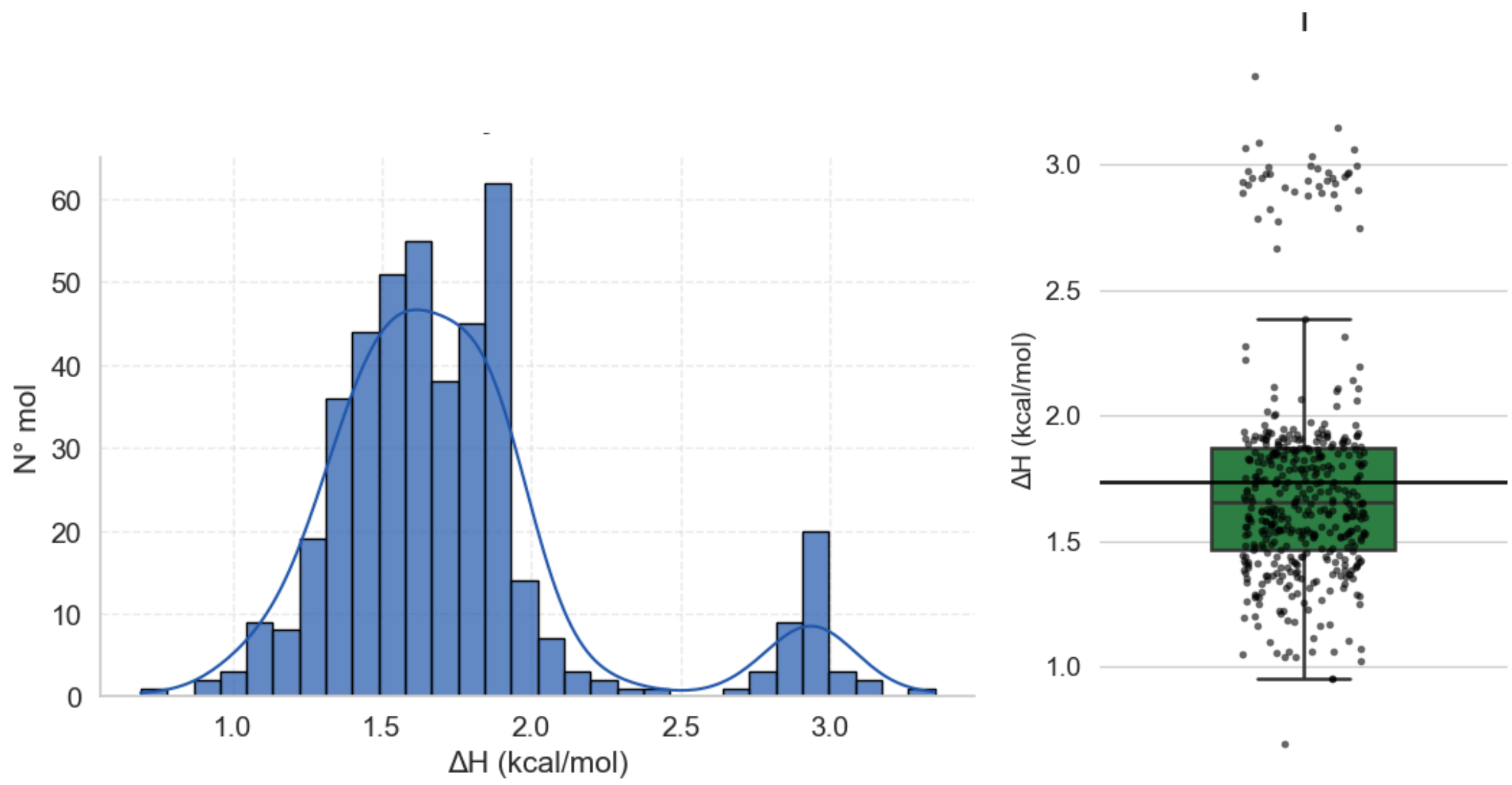

Figure S52: Statistical distribution of the enthalpy (ΔH) in the analyzed molecular dataset computed at the PM6 method. On the left, a histogram with its corresponding density curve; on the right, a boxplot with superimposed individual data points.

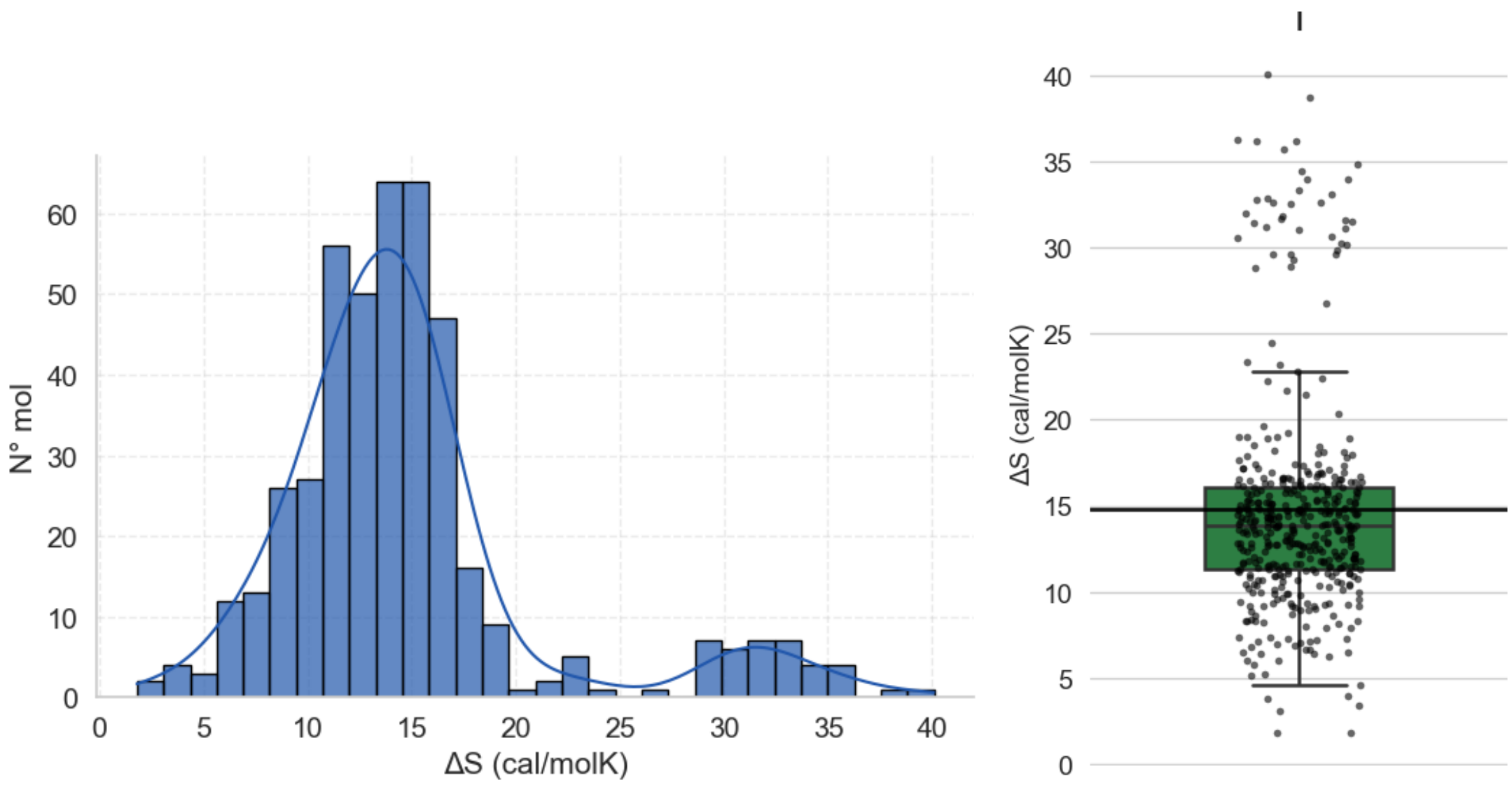

Figure S53: Statistical distribution of the entropy (ΔS) in the analyzed molecular dataset computed at the PM6 method. On the left, a histogram with its corresponding density curve; on the right, a boxplot with superimposed individual data points.

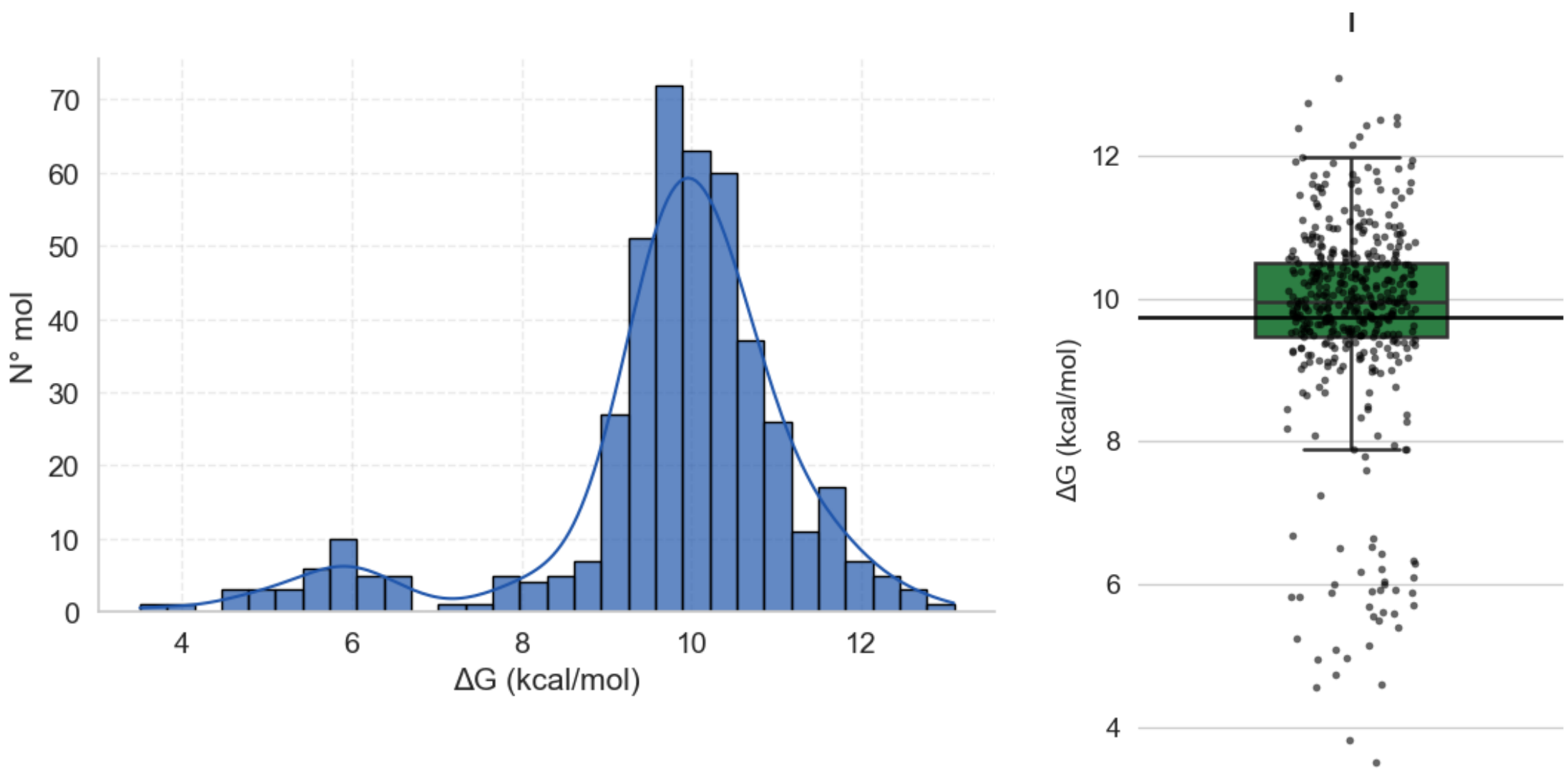

Figure S54: Statistical distribution of the Gibbs free energy (ΔG) in the analyzed molecular dataset computed at the PM6 method. On the left, a histogram with its corresponding density curve; on the right, a boxplot with superimposed individual data points.

S3. 9 Population distribution analysis and histograms of geometric, thermodynamic, and charge-transfer data calculated using PM7 method

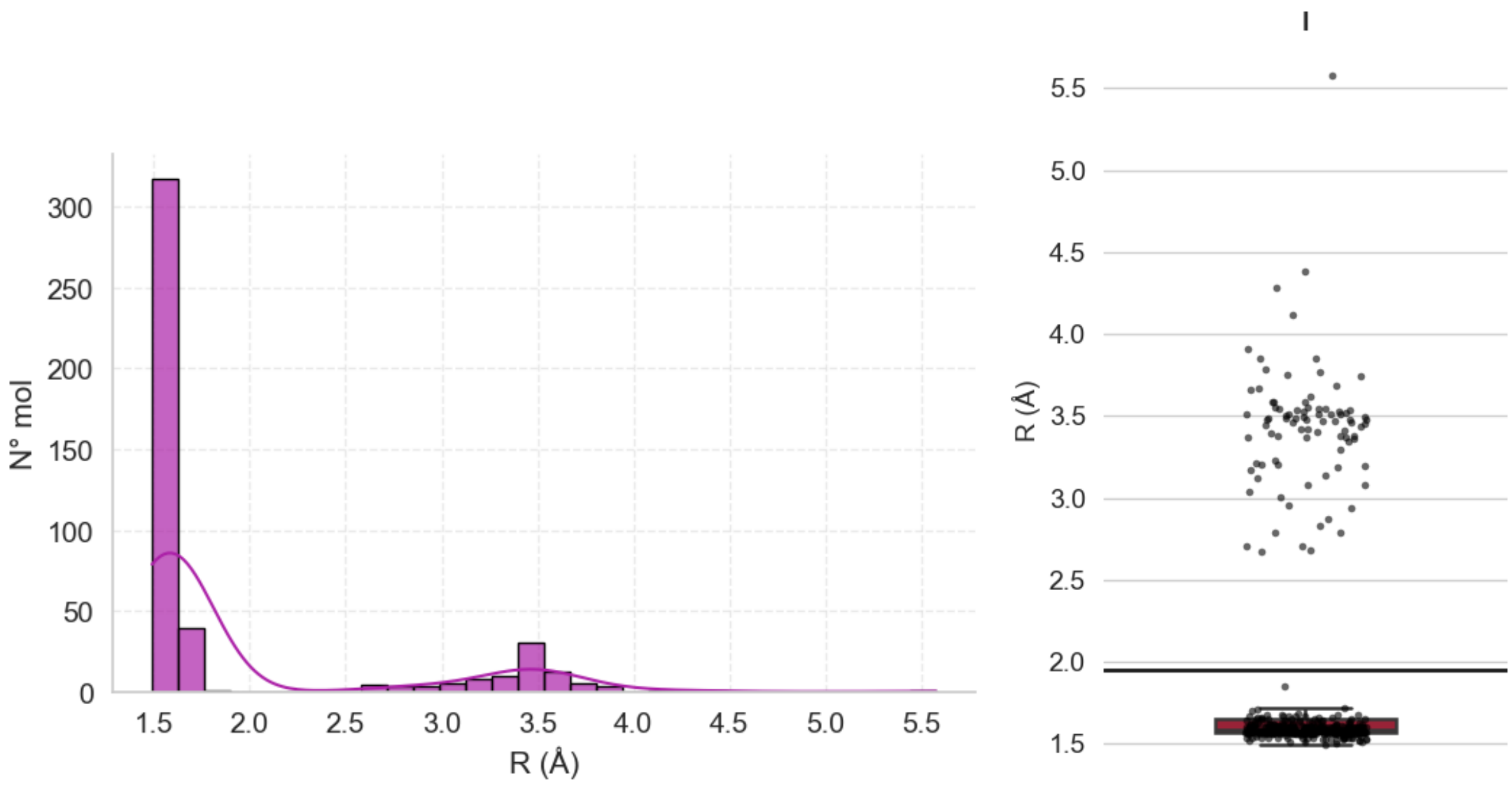

Figure S55: Statistical distribution of the length R (Å) in the analyzed molecular dataset computed at the PM7 method. On the left, a histogram with its corresponding density curve; on the right, a boxplot with superimposed individual data points.

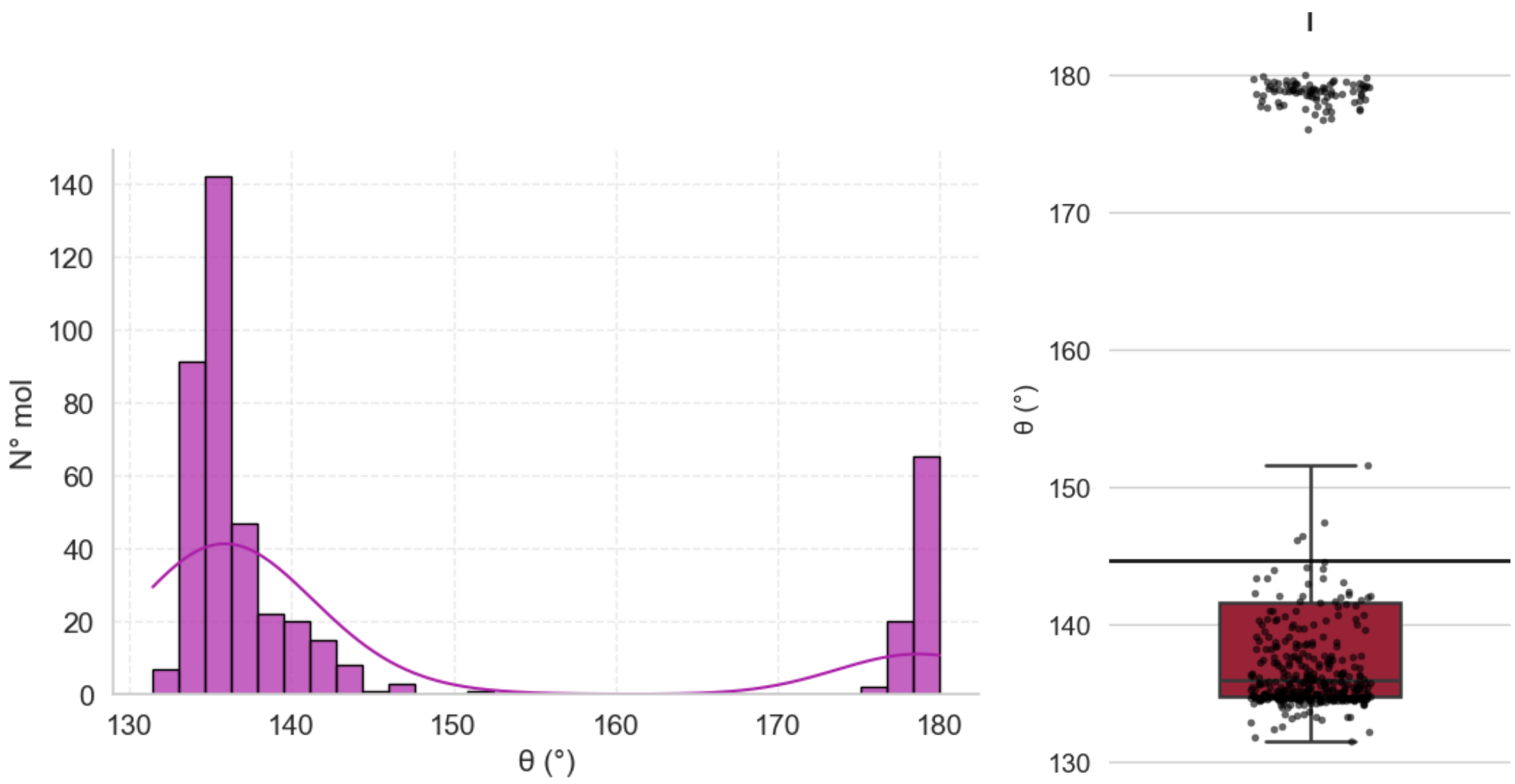

Figure S56: Statistical distribution of the bond angle (θ) of the O=C=O group in the analyzed molecular dataset computed at the PM7 method. On the left, a histogram with its corresponding density curve; on the right, a boxplot with superimposed individual data points.

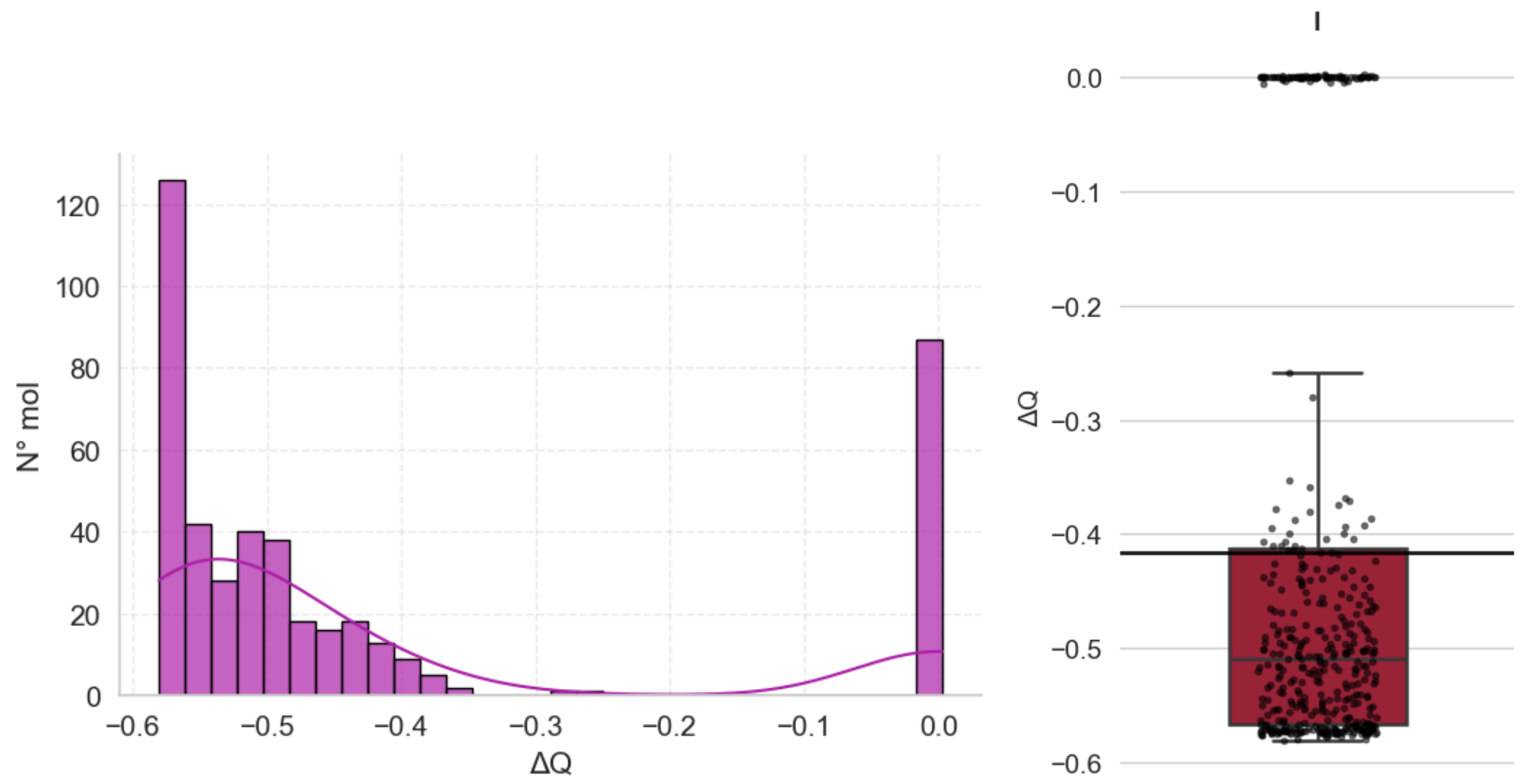

Figure S57: Statistical distribution of the charge transfer (ΔQ) in the analyzed molecular dataset computed at the PM7 method. On the left, a histogram with its corresponding density curve; on the right, a boxplot with superimposed individual data points.

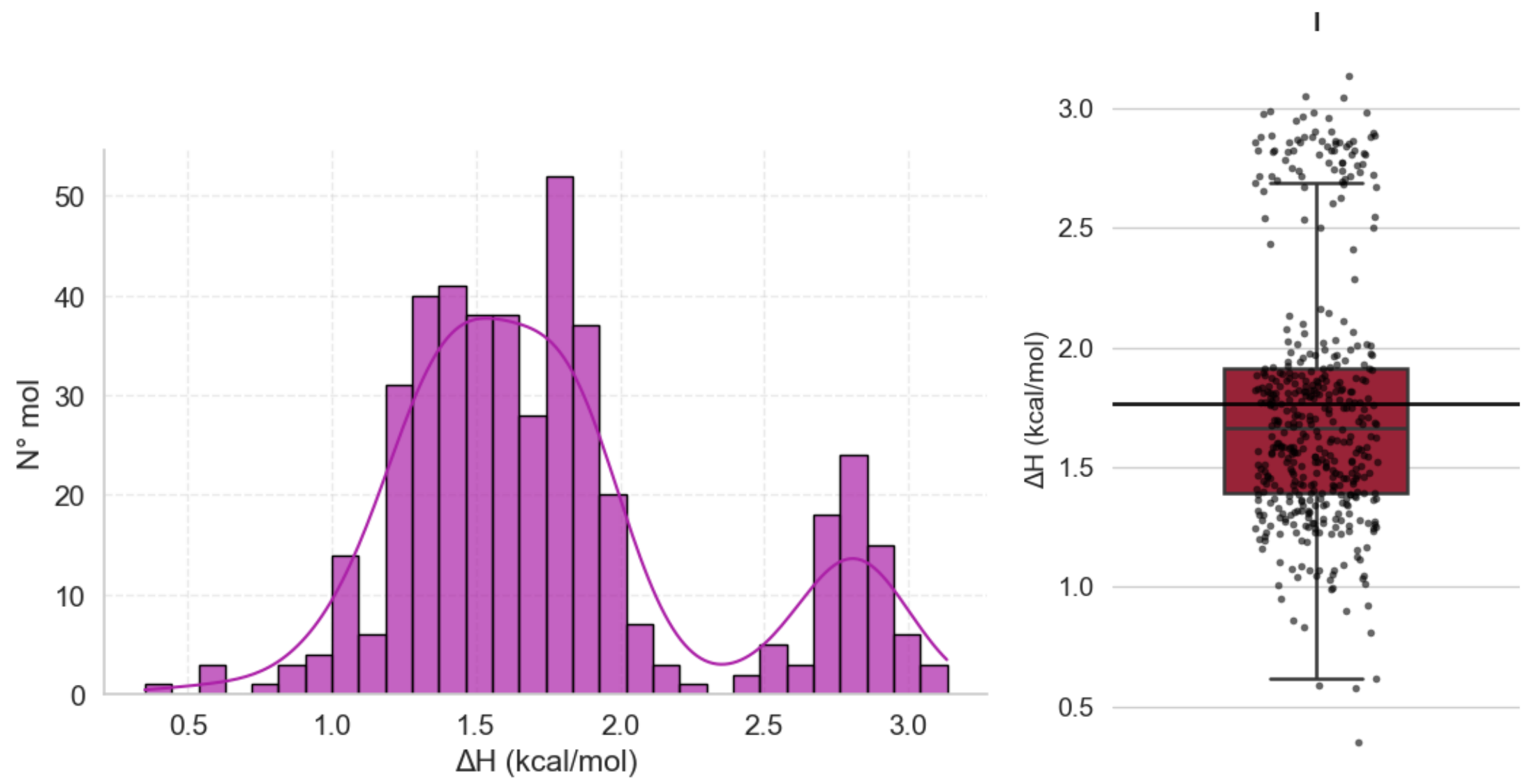

Figure S58: Statistical distribution of the enthalpy (ΔH) in the analyzed molecular dataset computed at the PM7 method. On the left, a histogram with its corresponding density curve; on the right, a boxplot with superimposed individual data points.

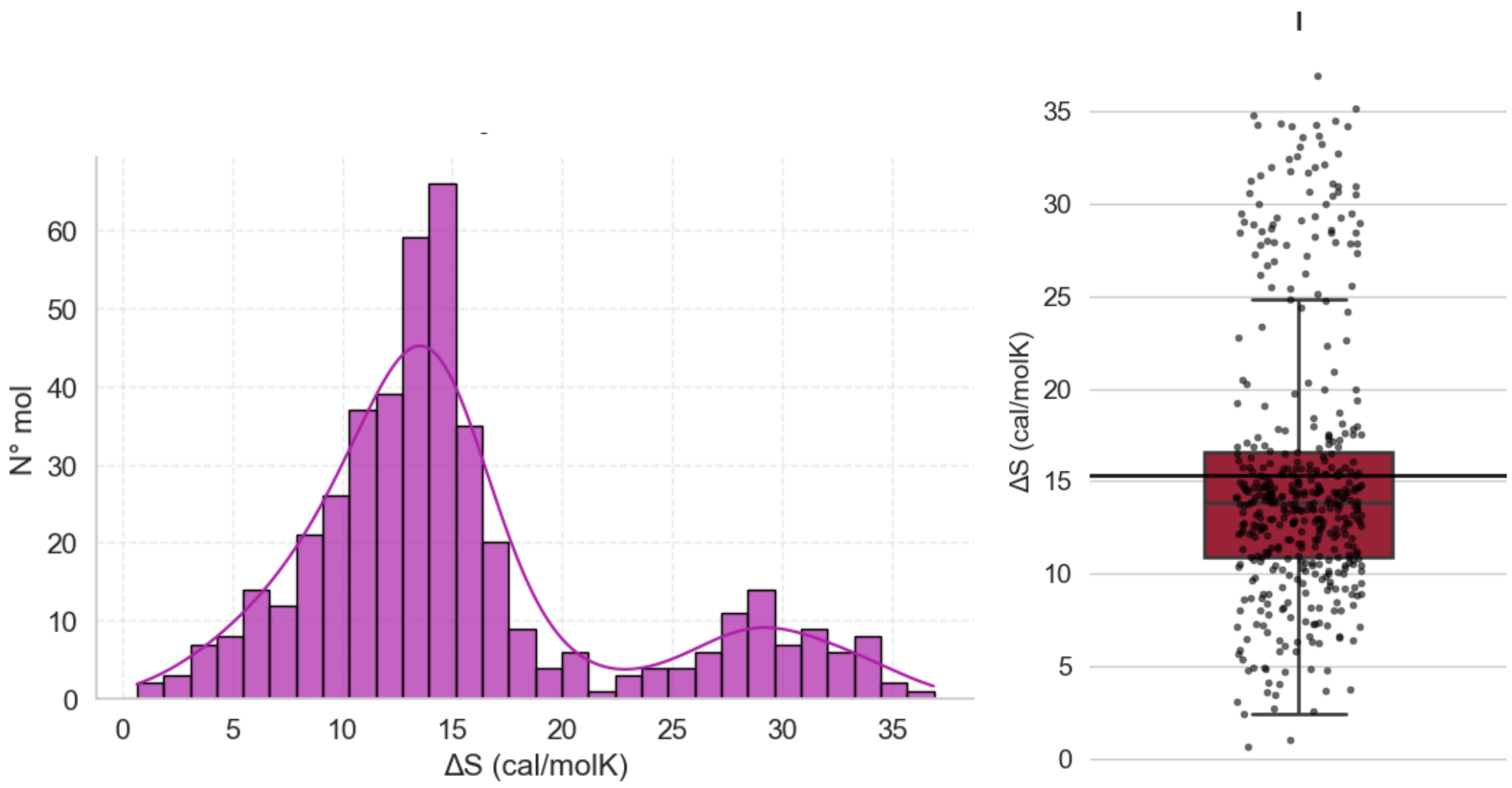

Figure S59: Statistical distribution of the entropy (ΔS) in the analyzed molecular dataset computed at the PM7 method. On the left, a histogram with its corresponding density curve; on the right, a boxplot with superimposed individual data points.

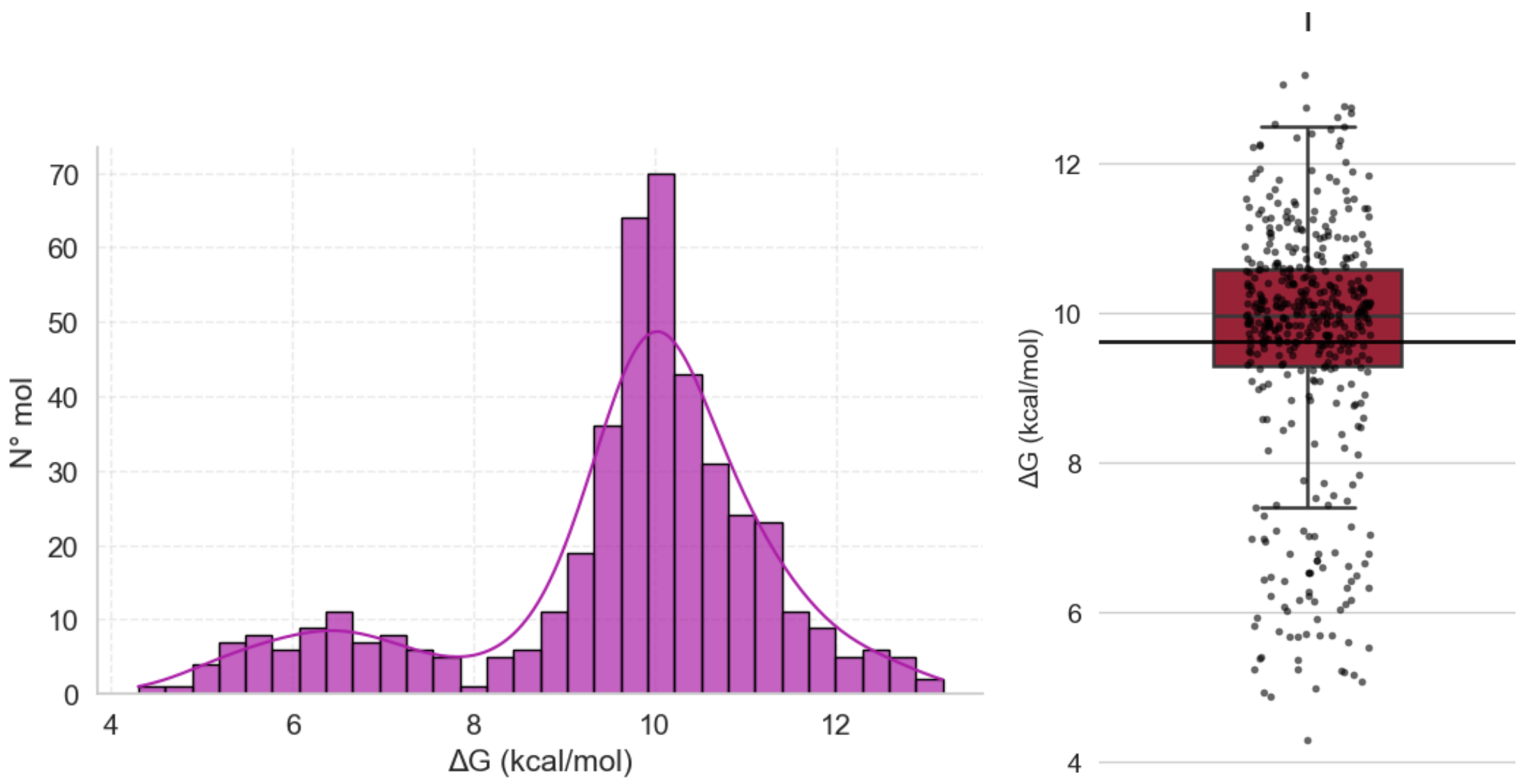

Figure S60: Statistical distribution of the Gibbs free energy (ΔG) in the analyzed molecular dataset computed at the PM7 method. On the left, a histogram with its corresponding density curve; on the right, a boxplot with superimposed individual data points.

S3. 9 Population distribution analysis and histograms of geometric, thermodynamic, and charge-transfer data calculated using RM1 method

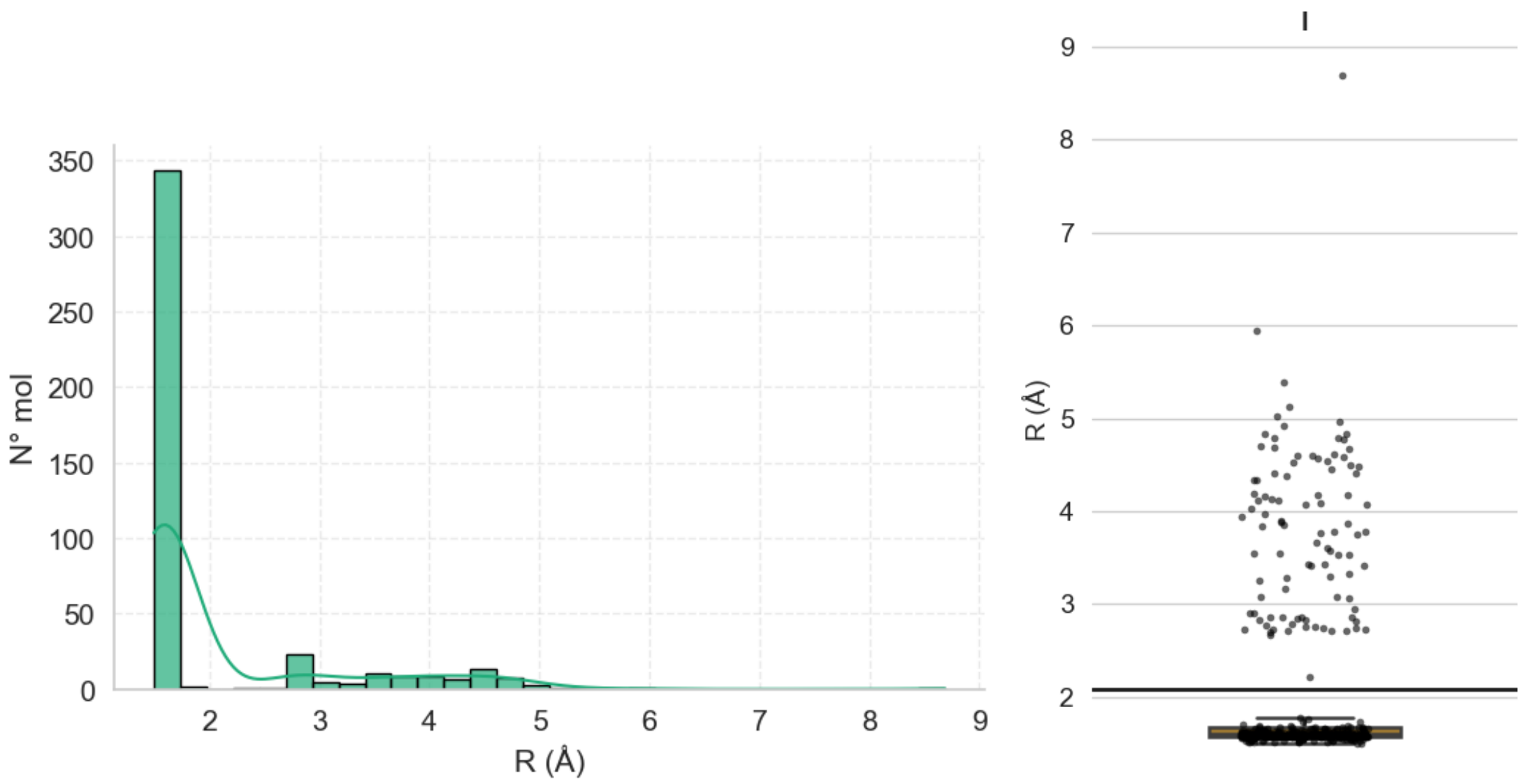

Figure S61: Statistical distribution of the length R (Å) in the analyzed molecular dataset computed at the RM1 method. On the left, a histogram with its corresponding density curve; on the right, a boxplot with superimposed individual data points.

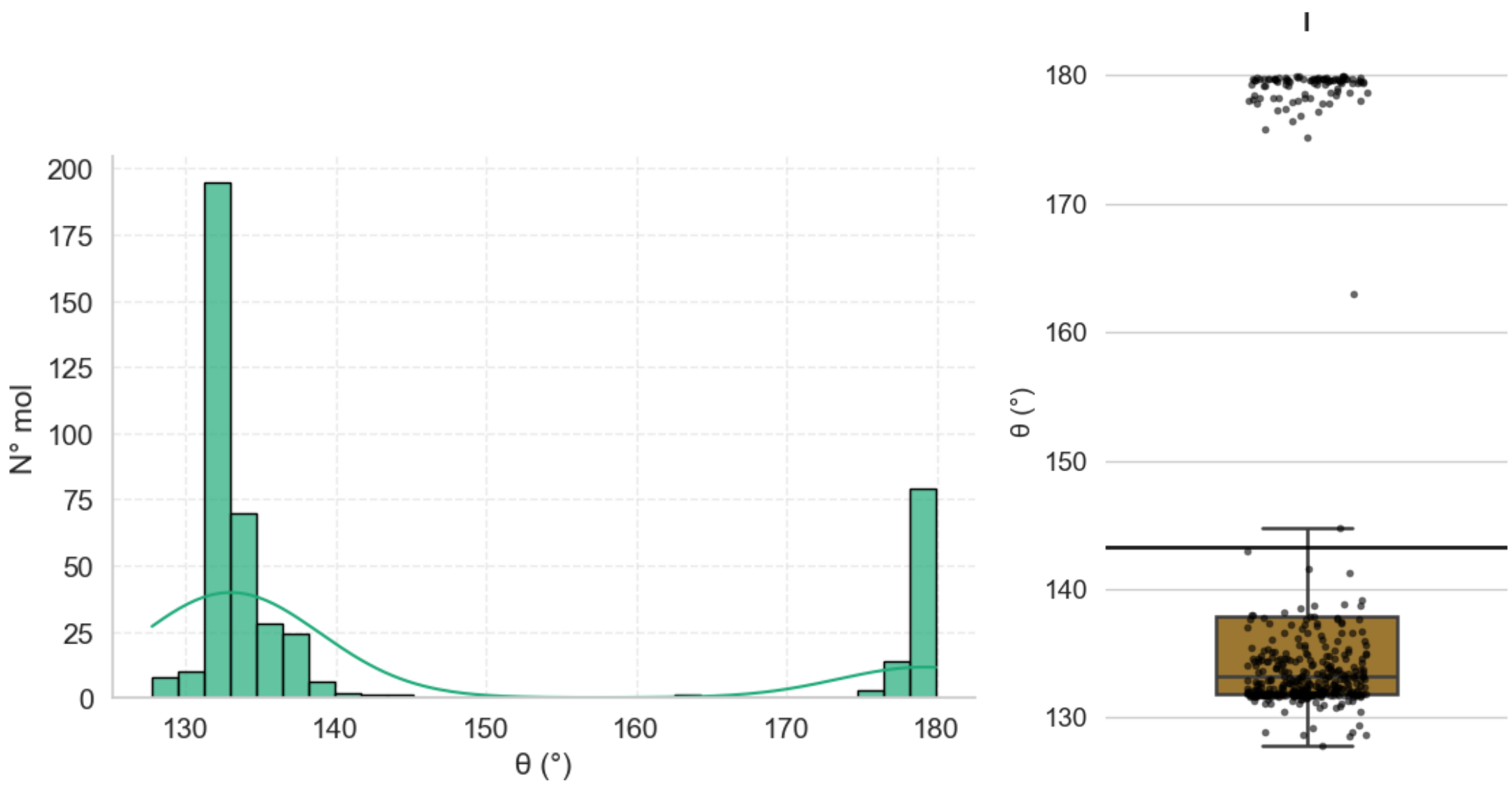

Figure S62: Statistical distribution of the bond angle (θ) of the O=C=O group in the analyzed molecular dataset computed at the RM1 method. On the left, a histogram with its corresponding density curve; on the right, a boxplot with superimposed individual data points.

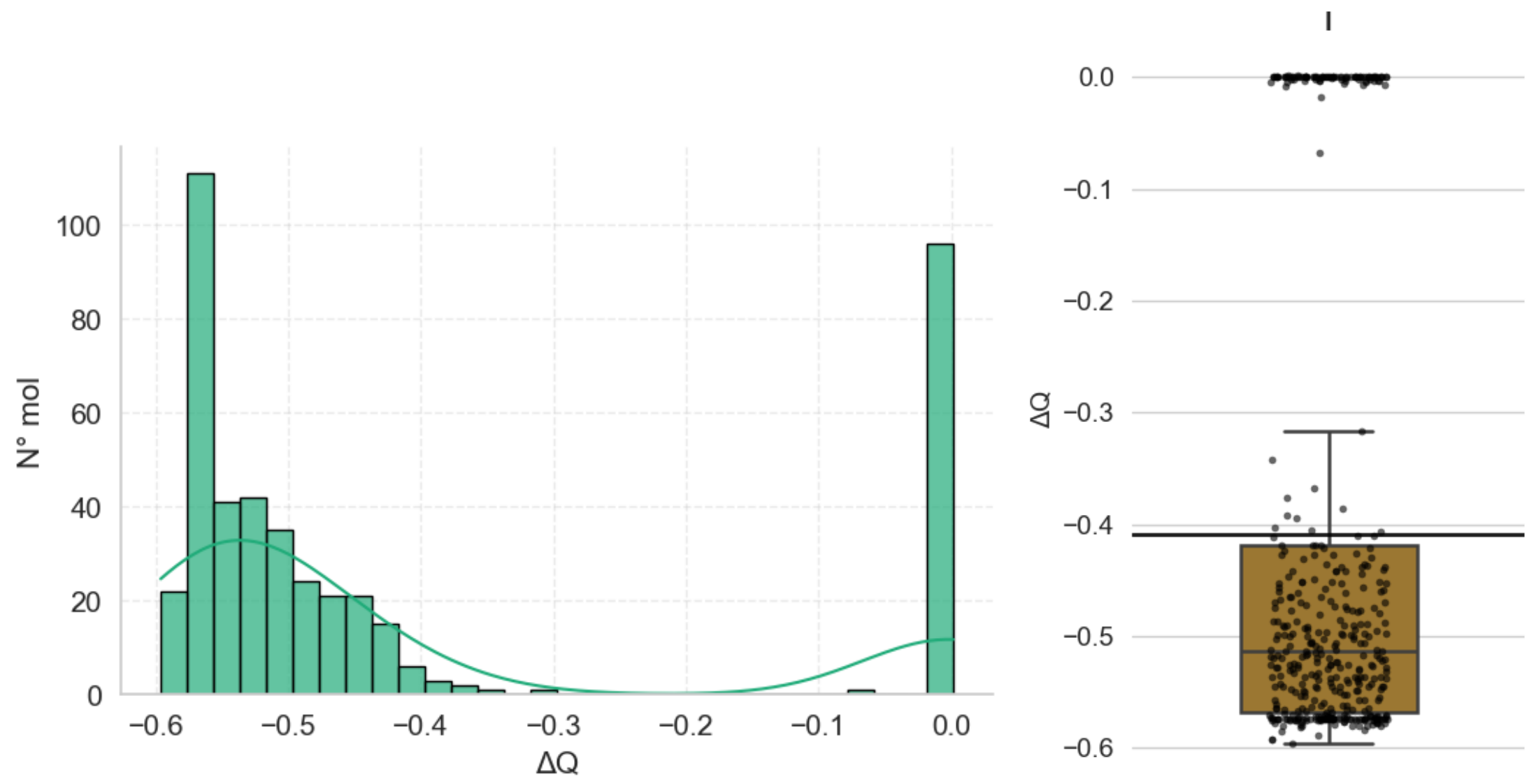

Figure S63: Statistical distribution of the charge transfer (ΔQ) in the analyzed molecular dataset computed at the RM1 method. On the left, a histogram with its corresponding density curve; on the right, a boxplot with superimposed individual data points.

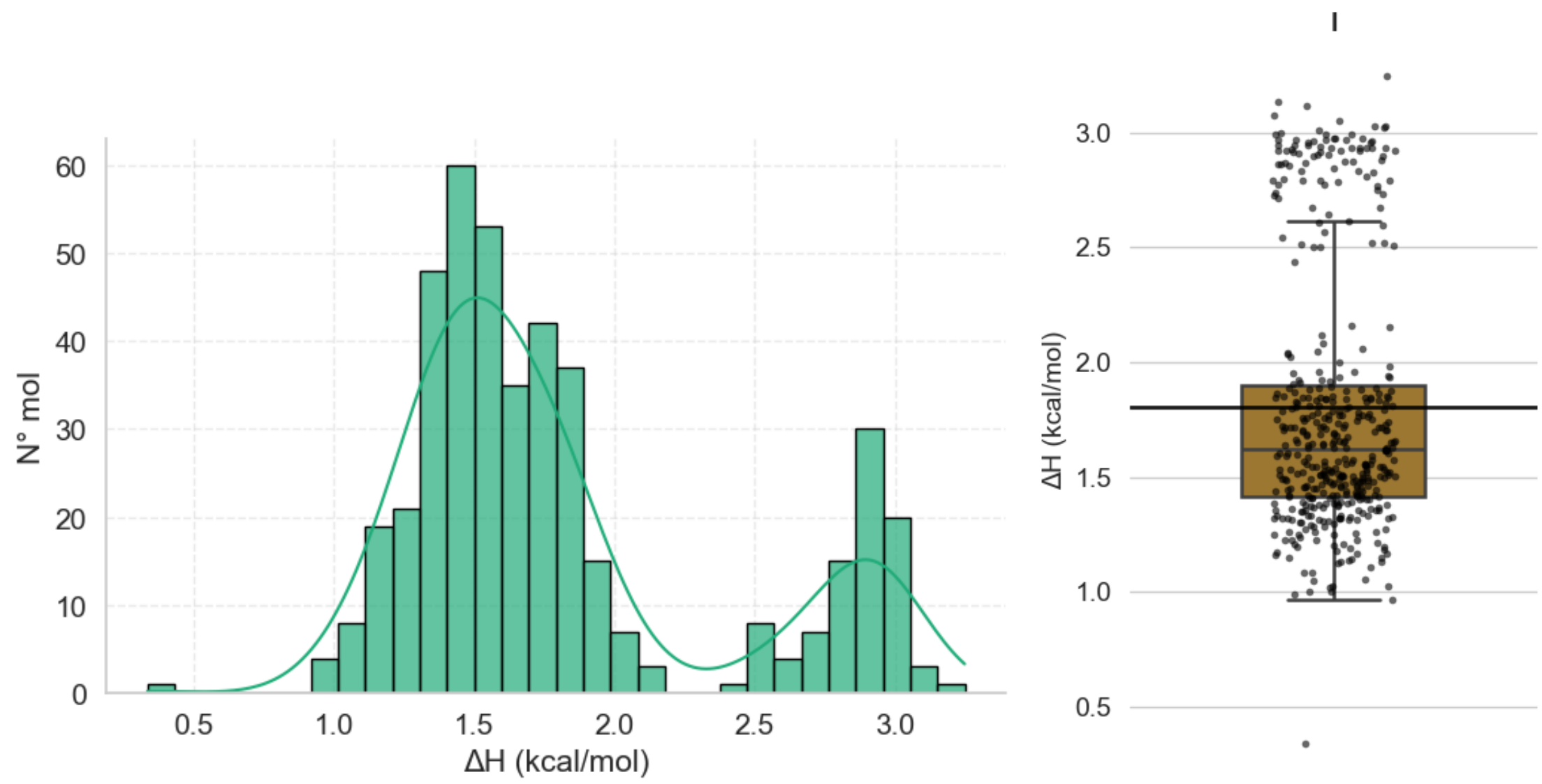

Figure S64: Statistical distribution of the enthalpy (ΔH) in the analyzed molecular dataset computed at the RM1 method. On the left, a histogram with its corresponding density curve; on the right, a boxplot with superimposed individual data points.

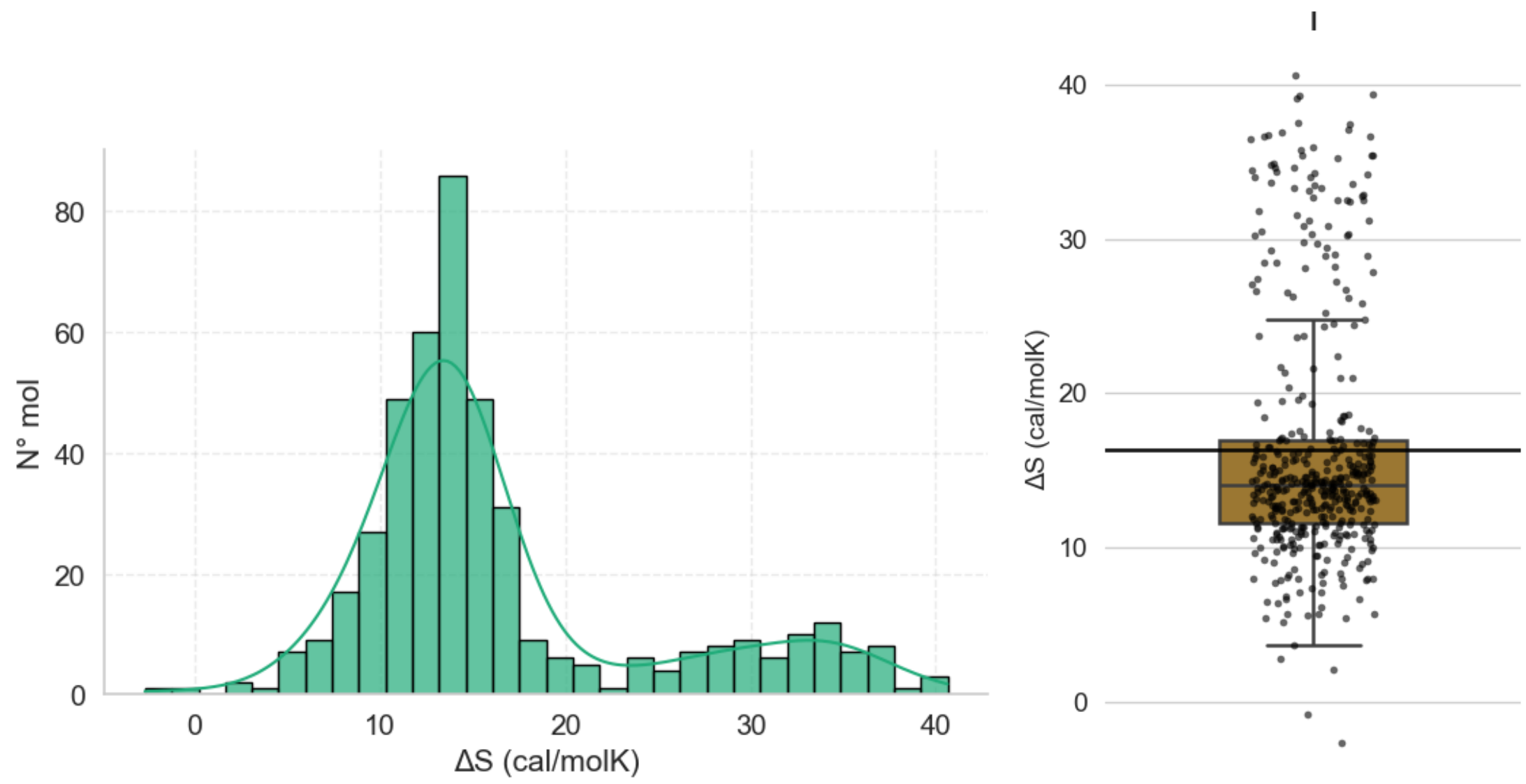

Figure S65: Statistical distribution of the entropy (ΔS) in the analyzed molecular dataset computed at the RM1 method. On the left, a histogram with its corresponding density curve; on the right, a boxplot with superimposed individual data points.

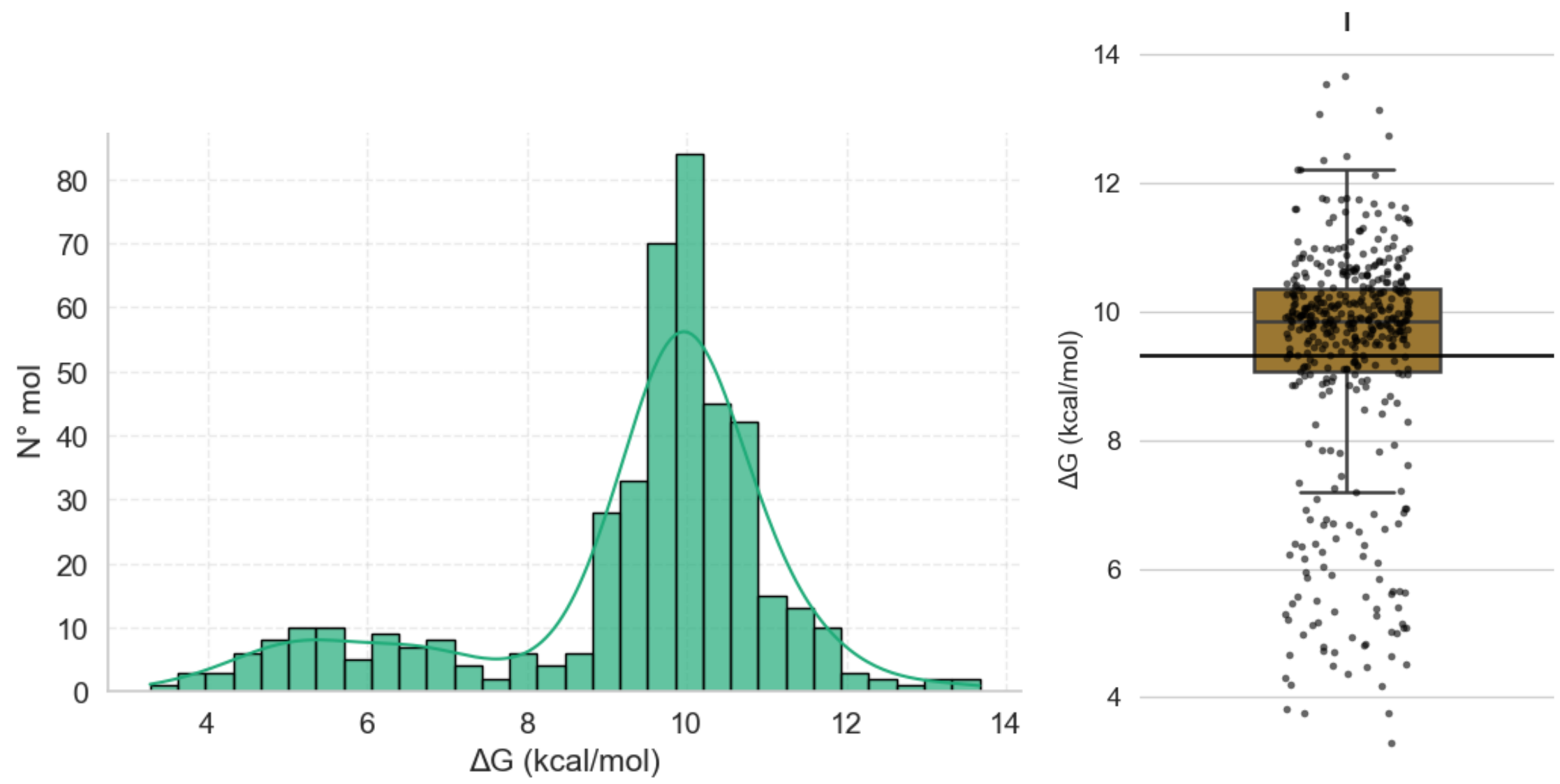

Figure S66: Statistical distribution of the Gibbs free energy (ΔG) in the analyzed molecular dataset computed at the RM1 method. On the left, a histogram with its corresponding density curve; on the right, a boxplot with superimposed individual data points.

S4. Scatter plots, Pearson R, MAE, RMSE, and R^2 for geometrical, thermodynamic, and charge-transfer data calculated using the semiempirical methods, employing the DFT method as reference.

S4. 1 Correlations between the AM1 method and the CAM-B3LYP/6-311++G(d,p) level of theory

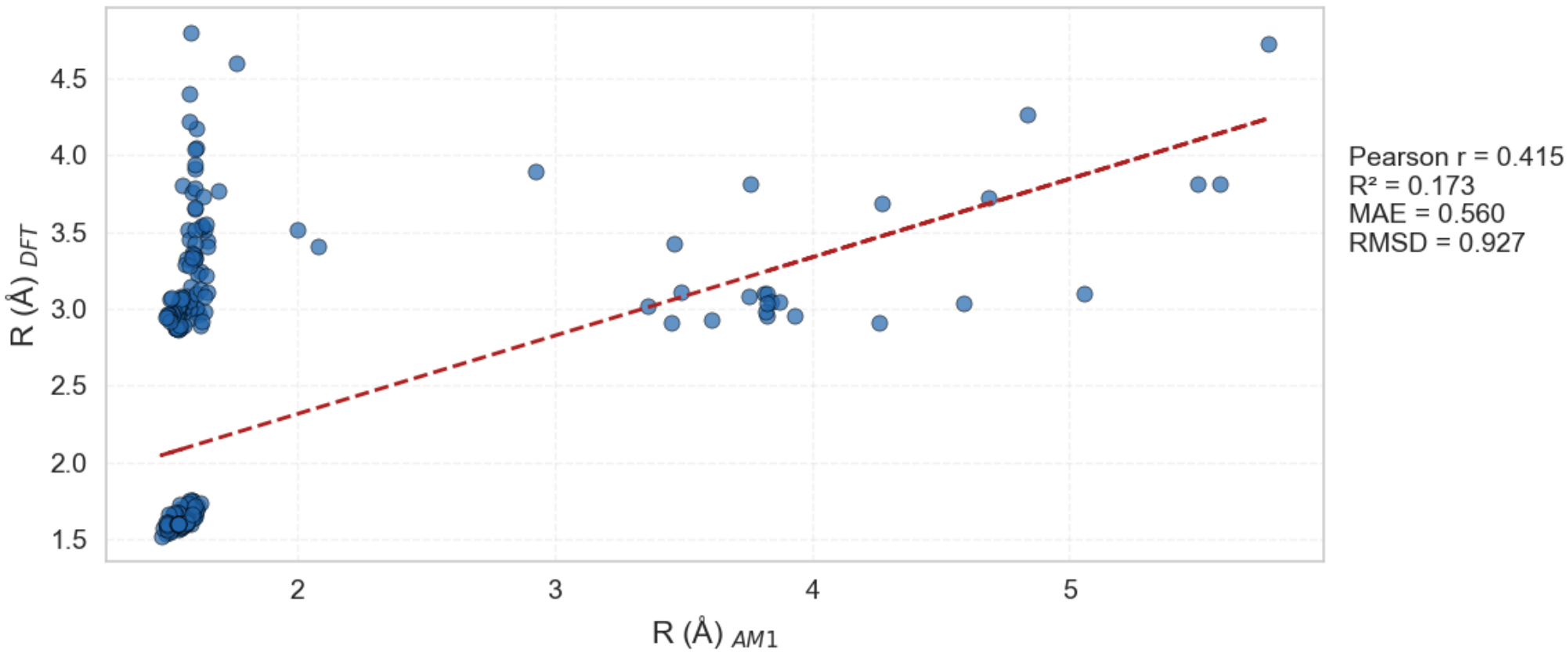

Figure S67: Correlation plot of the bond length R (Å) between values computed with the AM1 method and those obtained at the DFT CAM-B3LYP/6-311++G(d,p) level of theory.

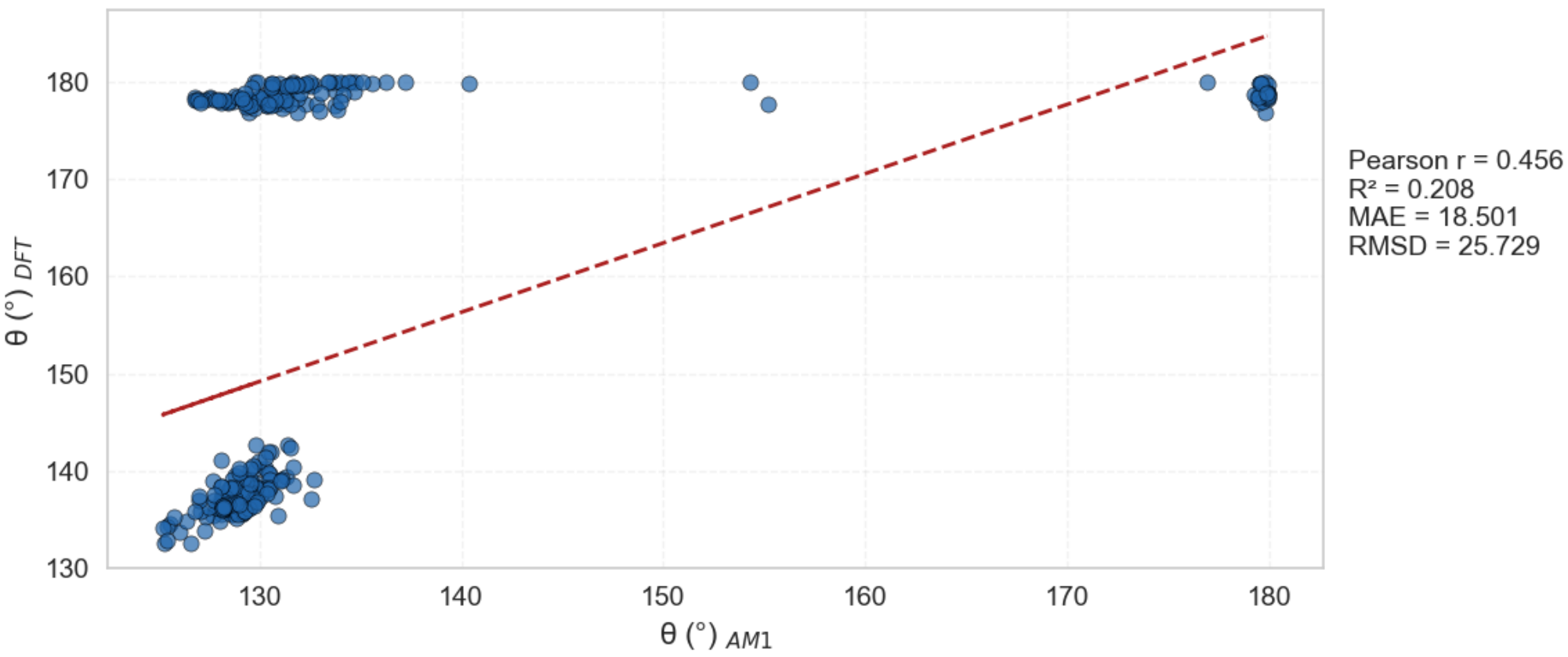

Figure S68: Correlation plot of the bond angle (θ) of the O=C=O between values computed with the AM1 method and those obtained at the DFT CAM-B3LYP/6-311++G(d,p) level of theory.

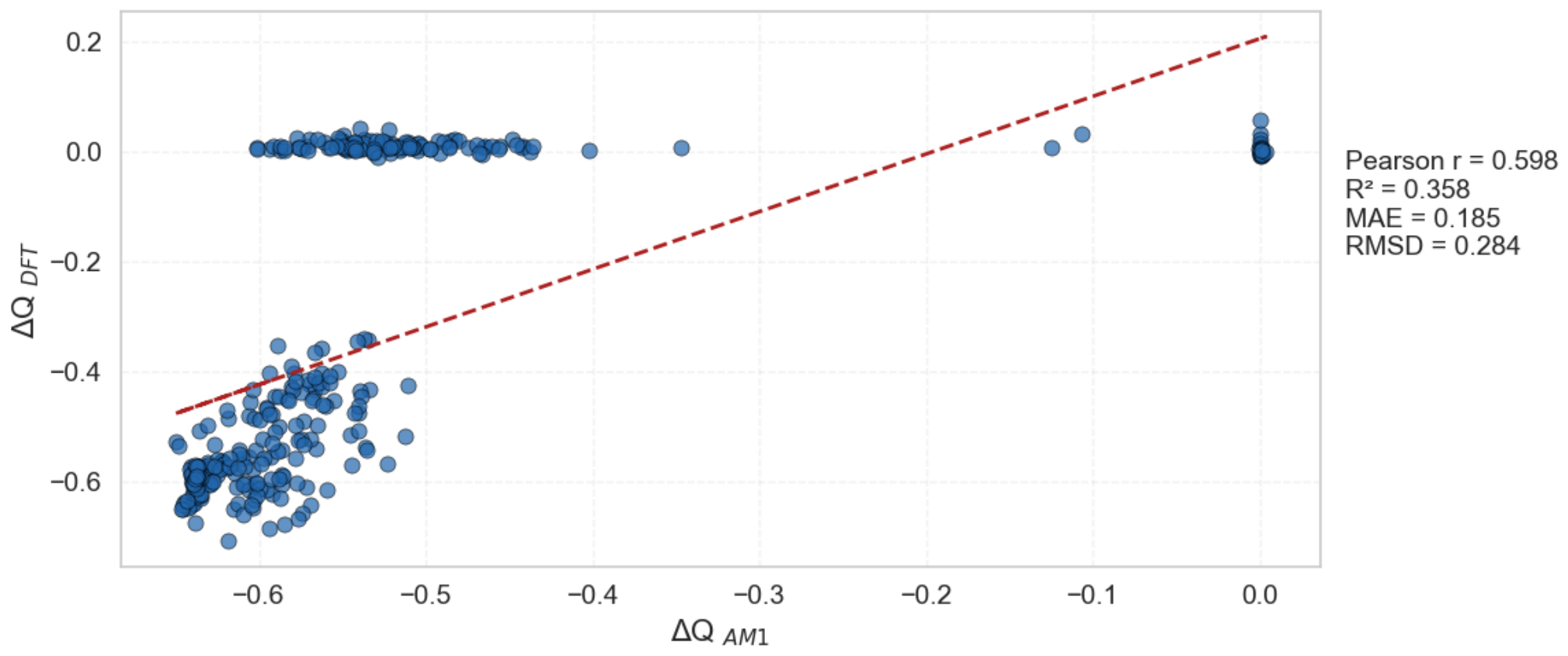

Figure S69: Correlation plot of the charge transfer (ΔQ) between values computed with the AM1 method and those obtained at the DFT CAM-B3LYP/6-311++G(d,p) level of theory.

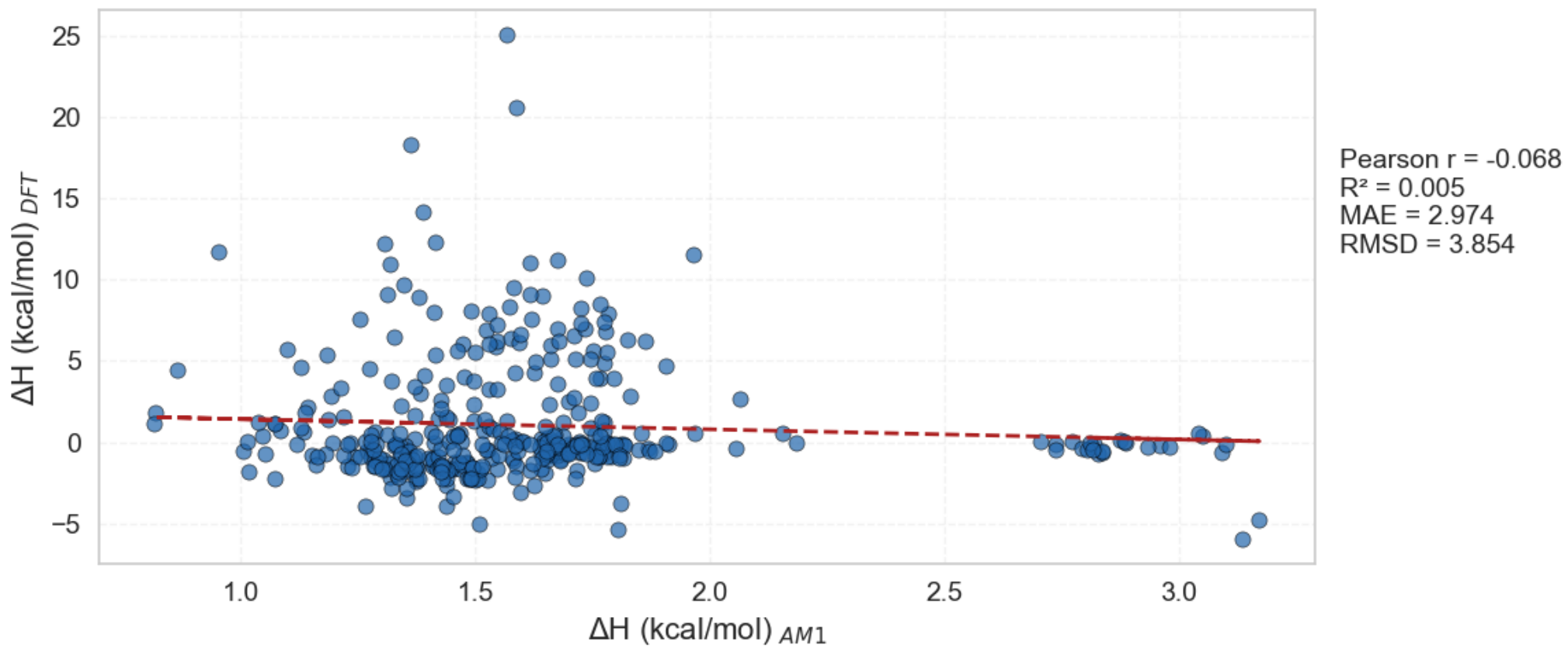

Figure S70: Correlation plot of the enthalpy (ΔH) between values computed with the AM1 method and those obtained at the DFT CAM-B3LYP/6-311++G(d,p) level of theory.

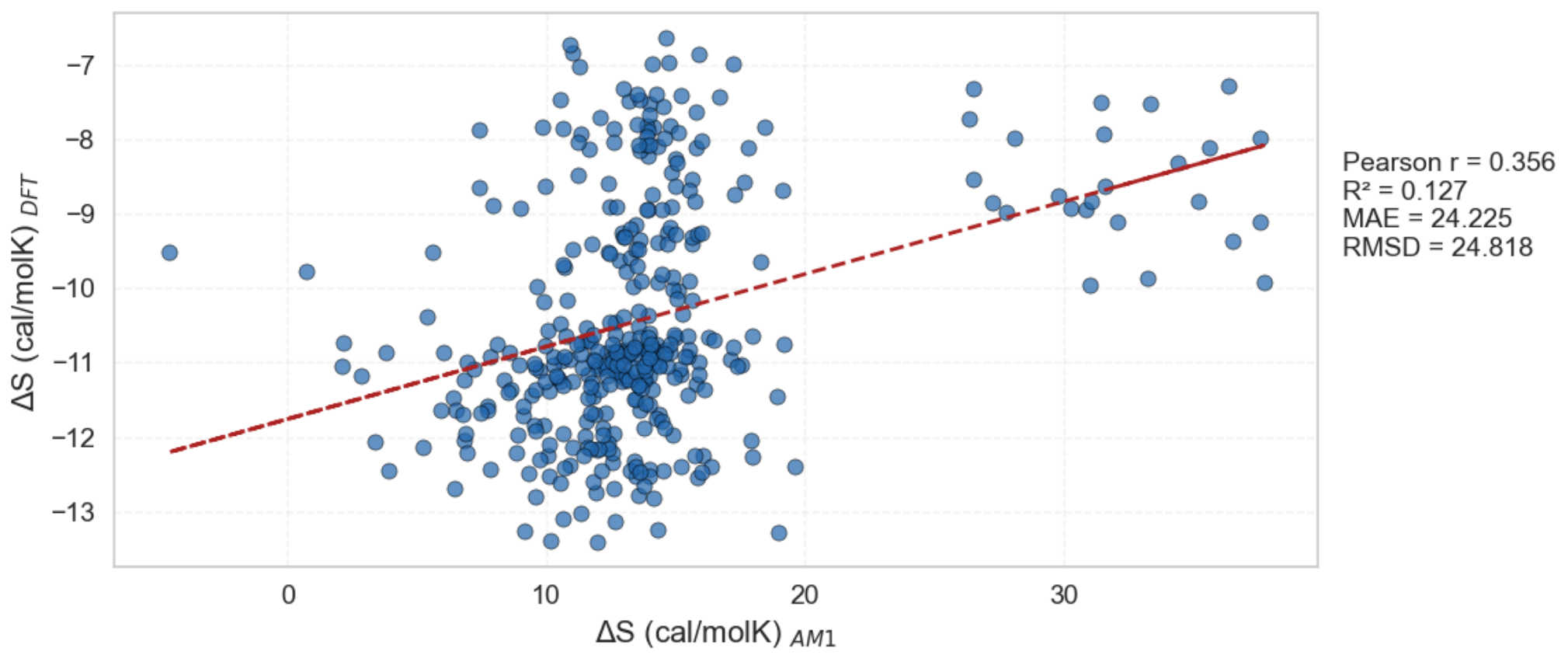

Figure S71: Correlation plot of the the entropy (ΔS) between values computed with the AM1 method and those obtained at the DFT CAM-B3LYP/6-311++G(d,p) level of theory.

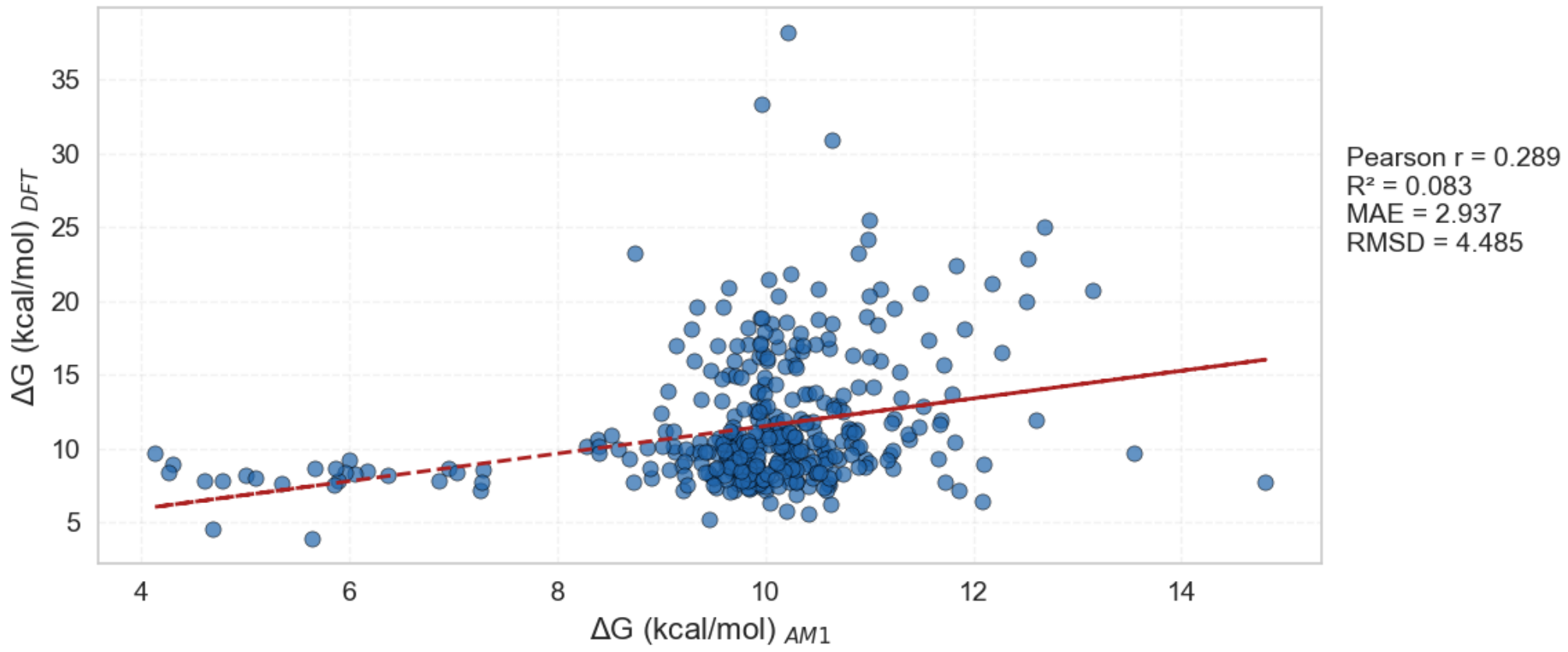

Figure S72: Correlation plot of the the Gibbs free energy (ΔG) between values computed with the AM1 method and those obtained at the DFT CAM-B3LYP/6-311++G(d,p) level of theory.

S4. 2 Correlations between the GFN0 method and the CAM-B3LYP/6-311++G(d,p) level of theory

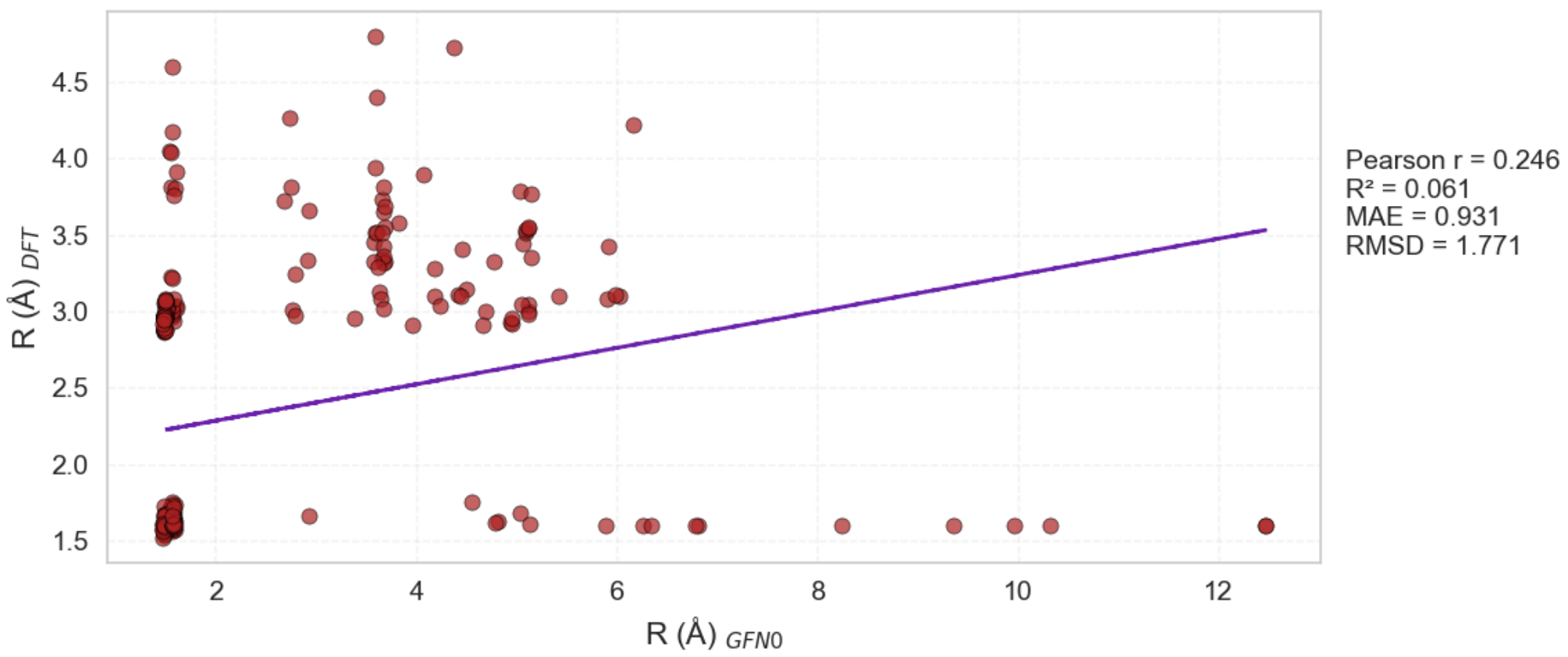

Figure S73: Correlation plot of the bond length R (Å) between values computed with the GFN0 method and those obtained at the DFT CAM-B3LYP/6-311++G(d,p) level of theory.

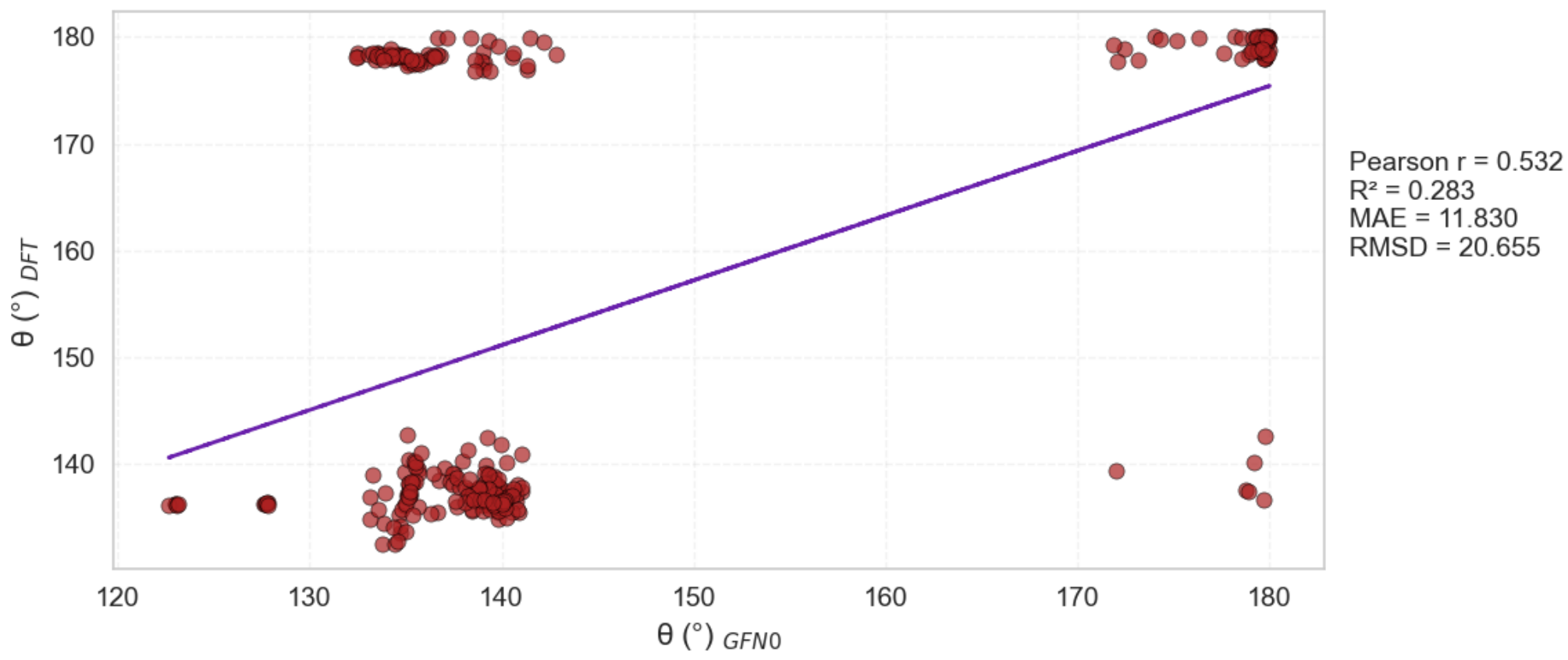

Figure S74: Correlation plot of the bond angle (θ) of the O=C=O between values computed with the GFN0 method and those obtained at the DFT CAM-B3LYP/6-311++G(d,p) level of theory.

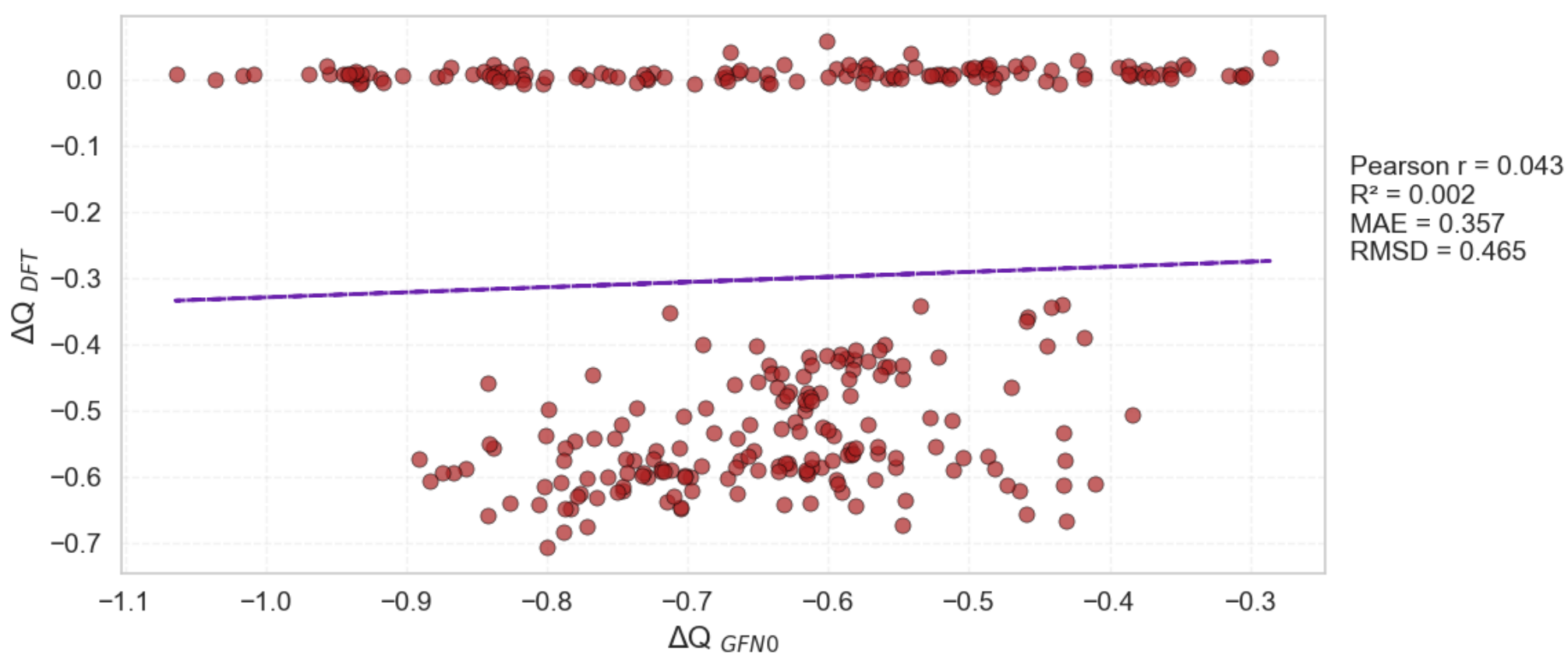

Figure S75: Correlation plot of the charge transfer (ΔQ) between values computed with the GFN0 method and those obtained at the DFT CAM-B3LYP/6-311++G(d,p) level of theory.

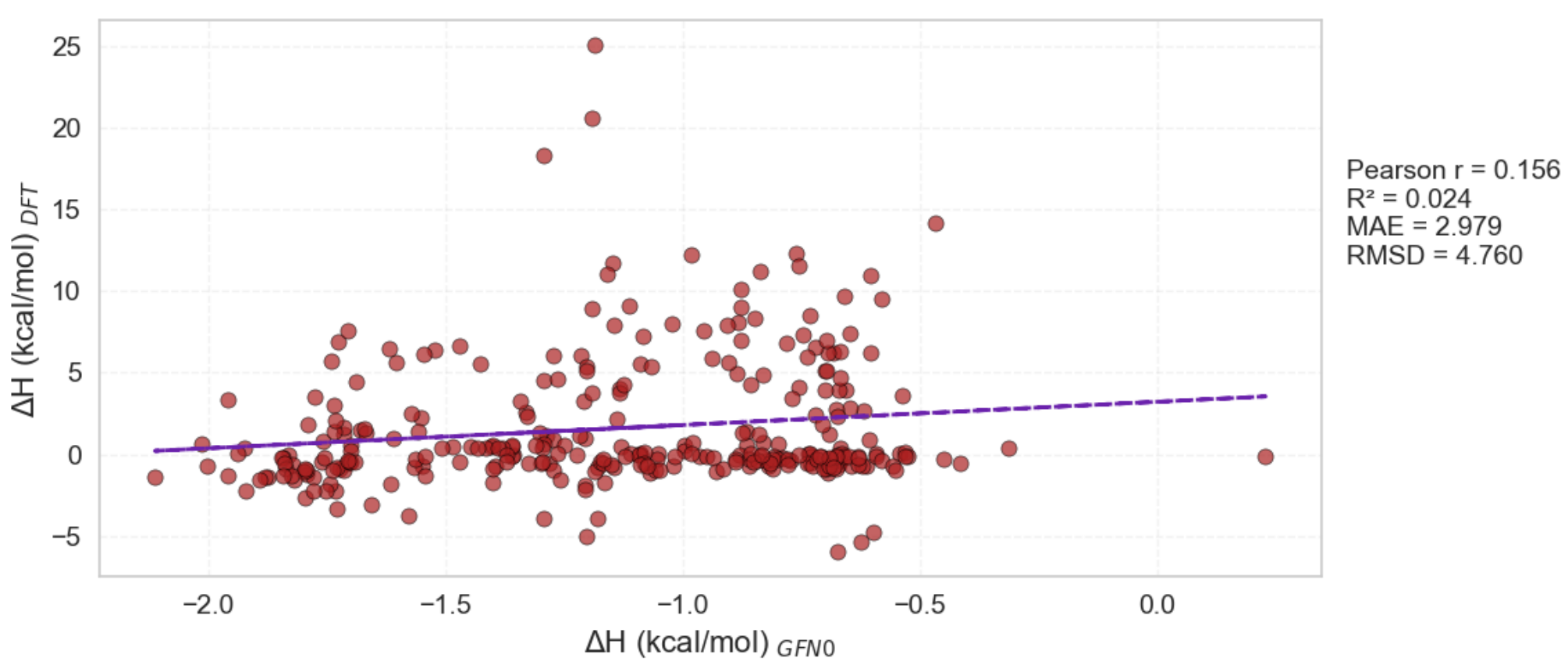

Figure S76: Correlation plot of the enthalpy (ΔH) between values computed with the GFN0 method and those obtained at the DFT CAM-B3LYP/6-311++G(d,p) level of theory.

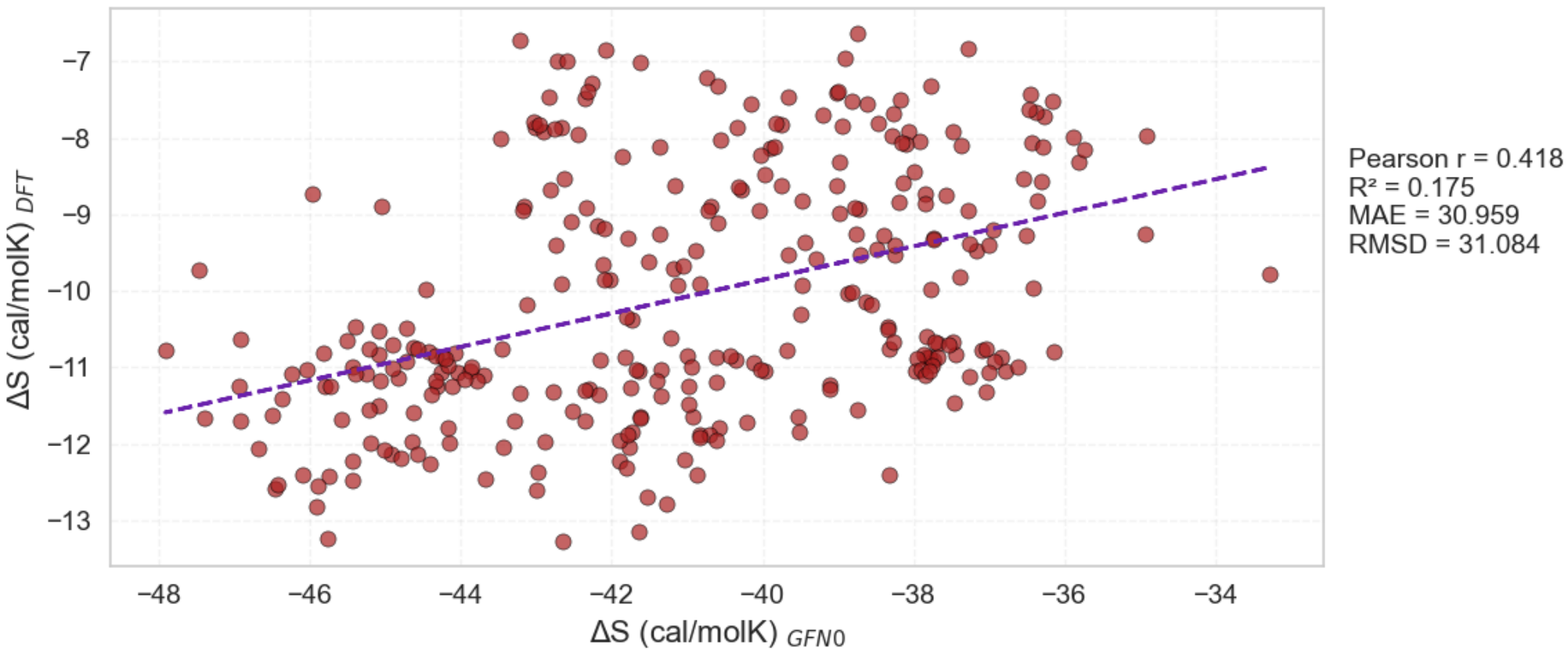

Figure S77: Correlation plot of the the entropy (ΔS) between values computed with the GFN0 method and those obtained at the DFT CAM-B3LYP/6-311++G(d,p) level of theory.

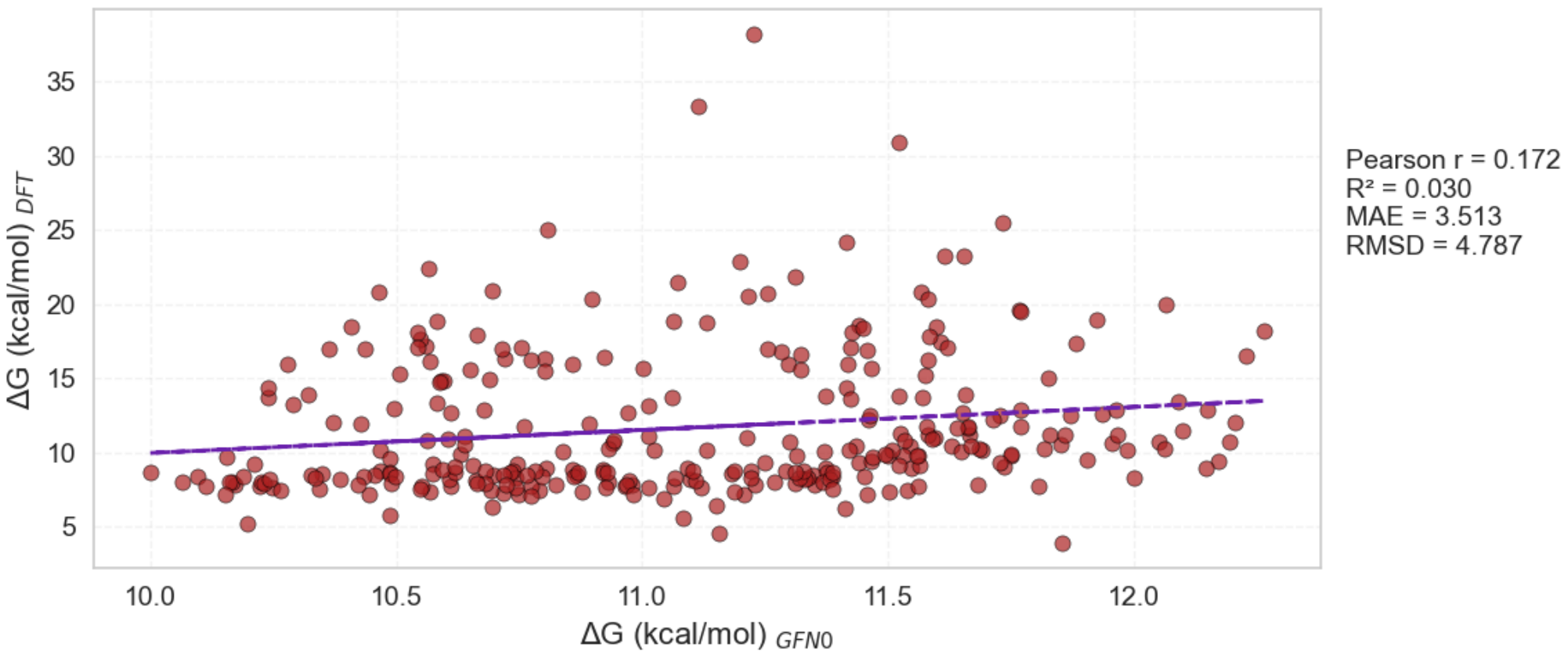

Figure S78: Correlation plot of the the Gibbs free energy (ΔG) between values computed with the GFN0 method and those obtained at the DFT CAM-B3LYP/6-311++G(d,p) level of theory.

S4. 3 Correlations between the GFN1 method and the CAM-B3LYP/6-311++G(d,p) level of theory

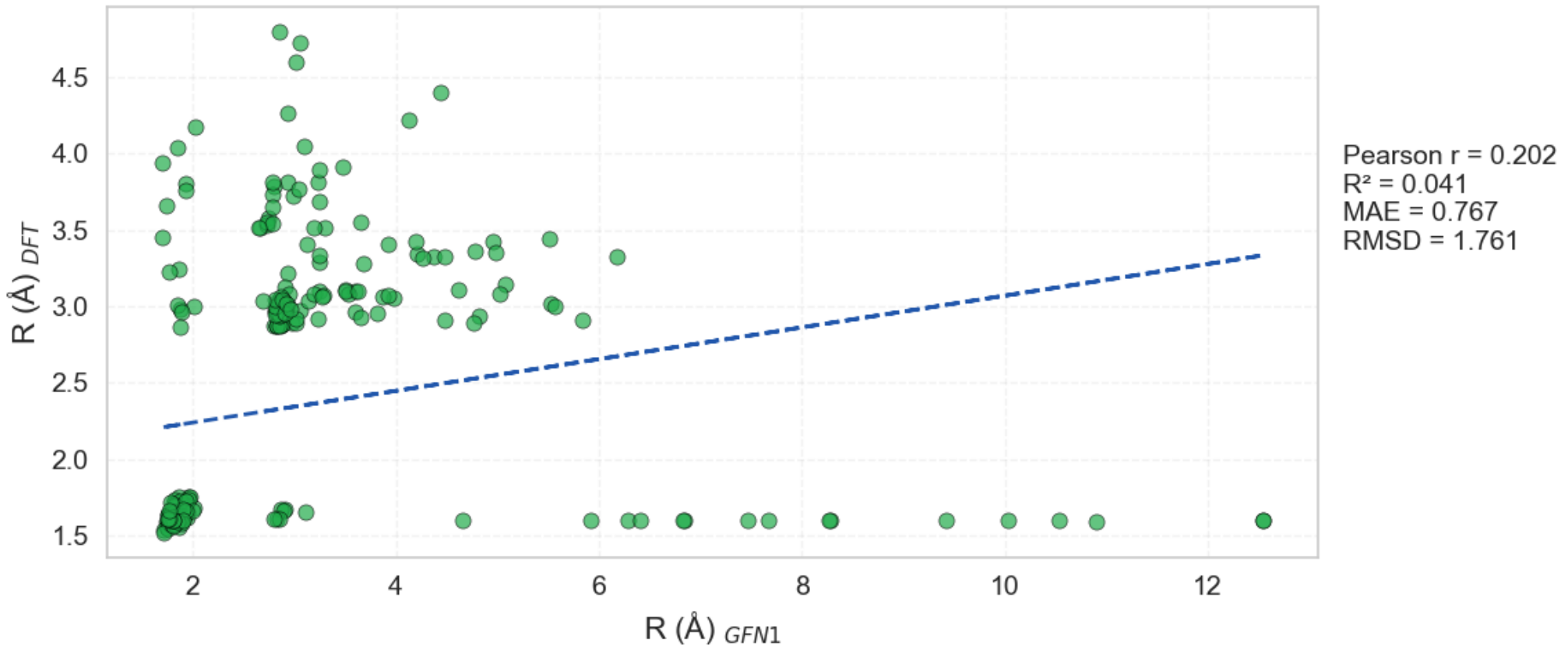

Figure S79: Correlation plot of the bond length R (Å) between values computed with the GFN1 method and those obtained at the DFT CAM-B3LYP/6-311++G(d,p) level of theory.

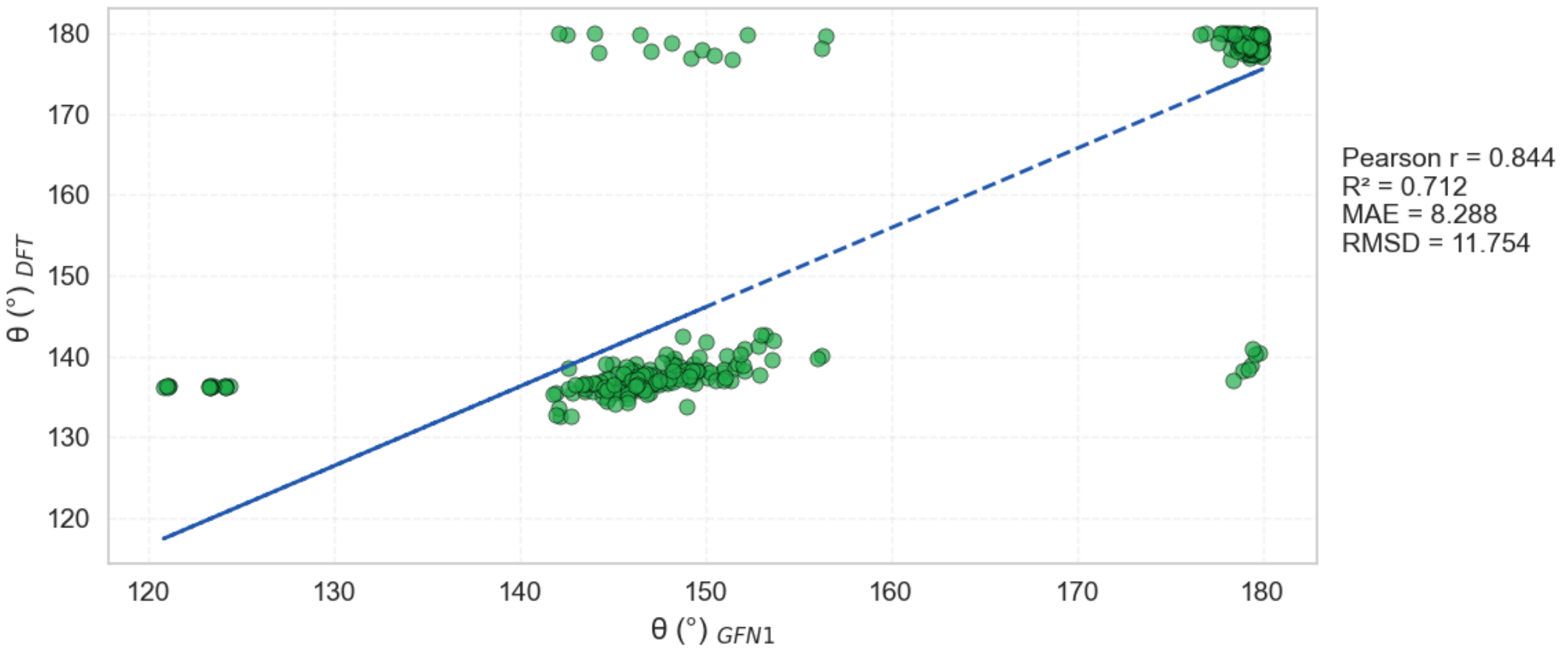

Figure S80: Correlation plot of the bond angle (θ) of the O=C=O between values computed with the GFN1 method and those obtained at the DFT CAM-B3LYP/6-311++G(d,p) level of theory.

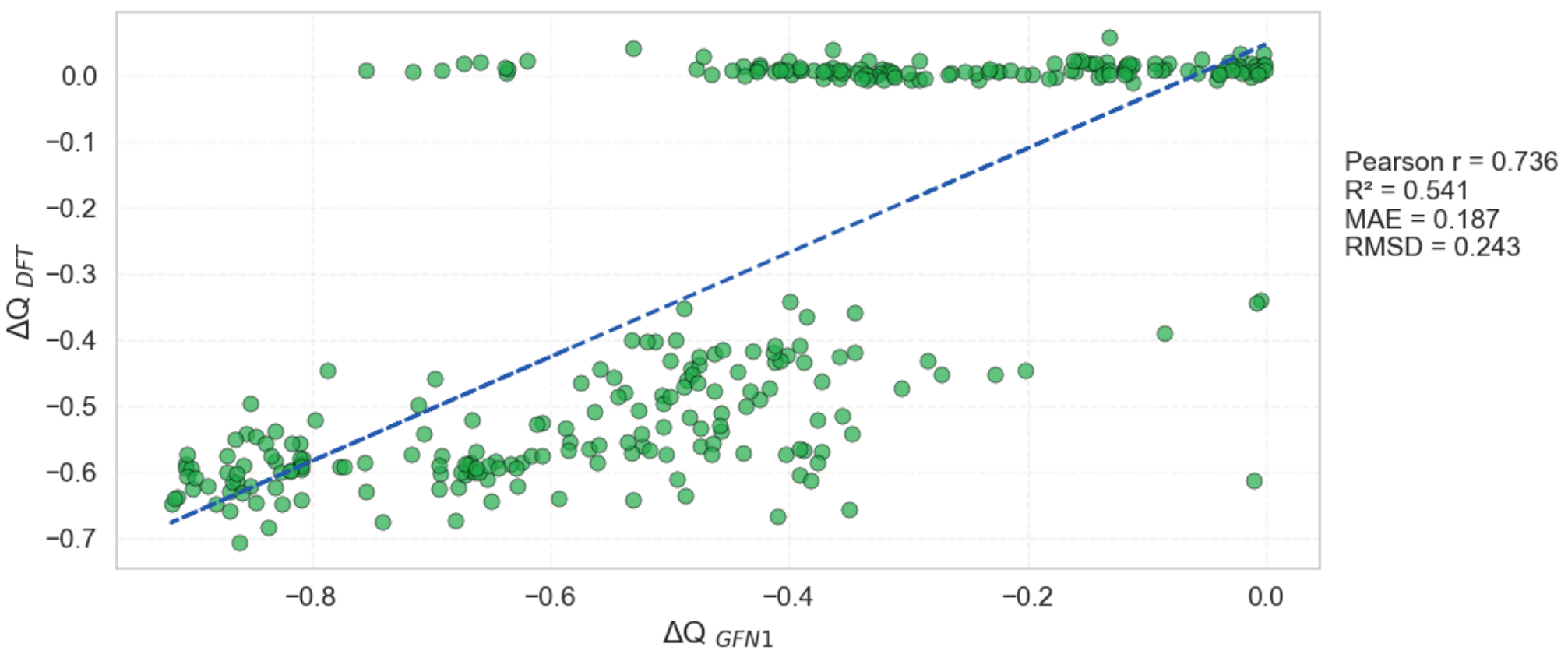

Figure S81: Correlation plot of the charge transfer (ΔQ) between values computed with the GFN1 method and those obtained at the DFT CAM-B3LYP/6-311++G(d,p) level of theory.

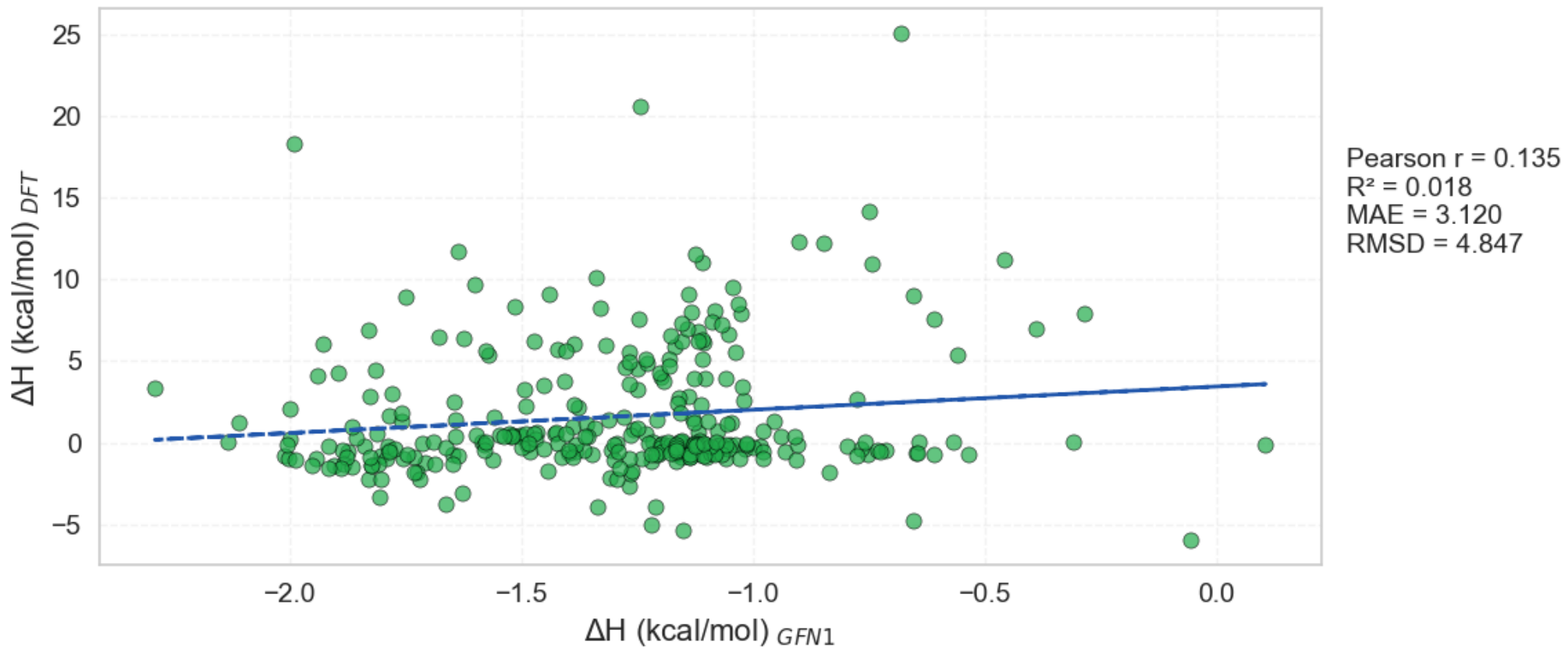

Figure S82: Correlation plot of the enthalpy (ΔH) between values computed with the GFN1 method and those obtained at the DFT CAM-B3LYP/6-311++G(d,p) level of theory.

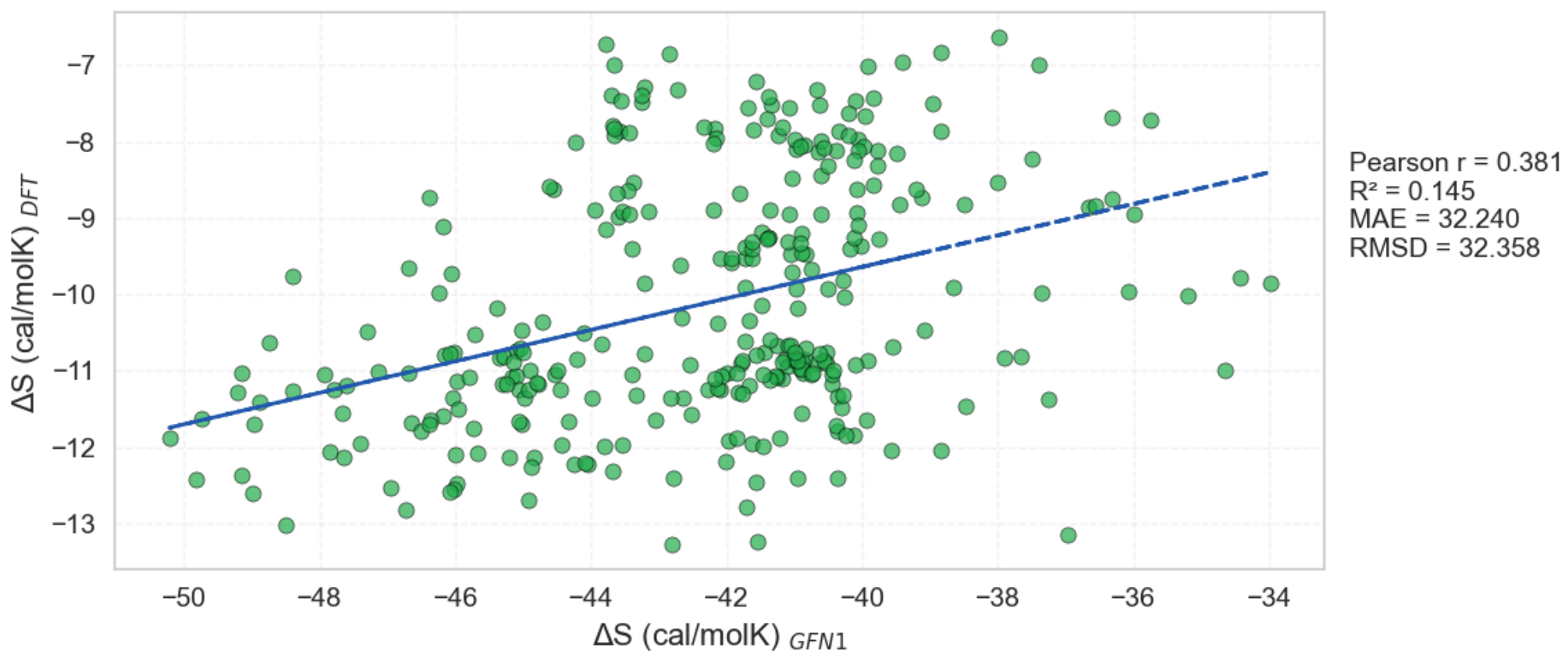

Figure S83: Correlation plot of the the entropy (ΔS) between values computed with the GFN1 method and those obtained at the DFT CAM-B3LYP/6-311++G(d,p) level of theory.

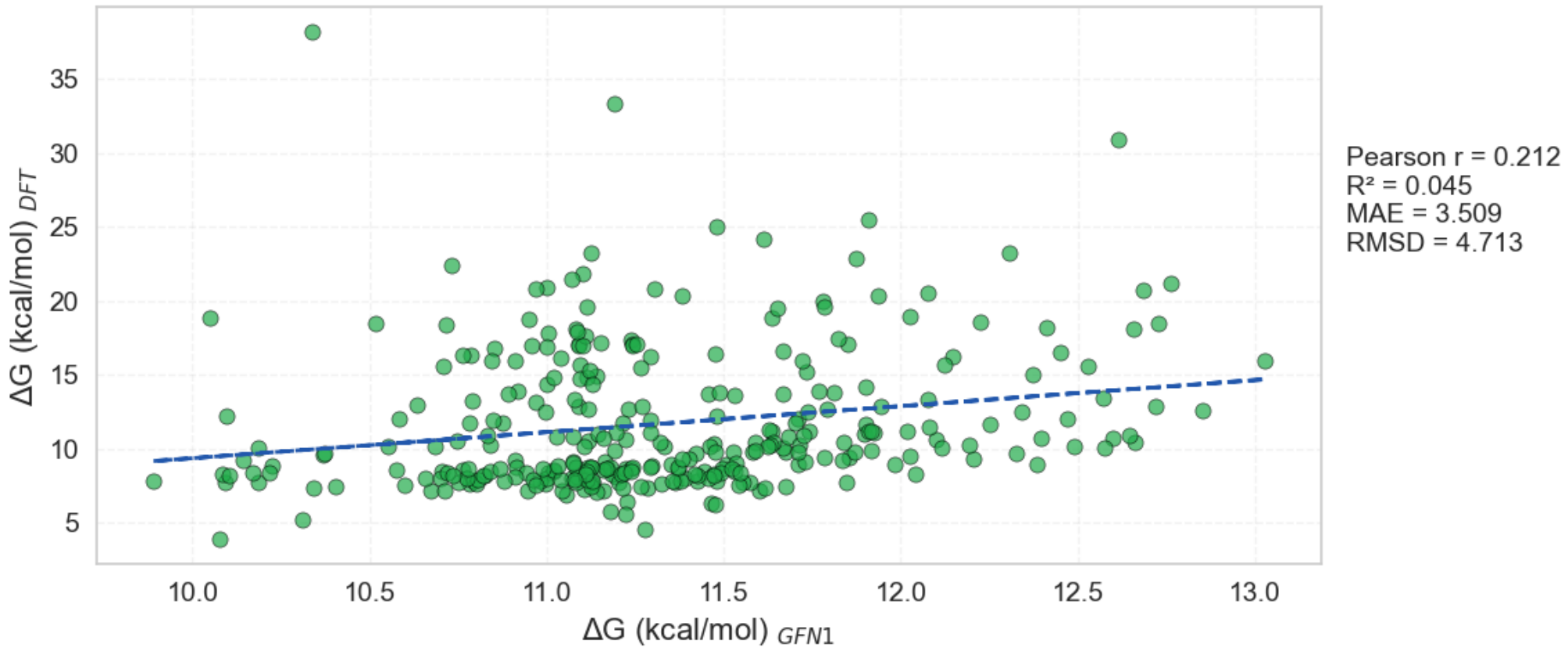

Figure S84: Correlation plot of the the Gibbs free energy (ΔG) between values computed with the GFN1 method and those obtained at the DFT CAM-B3LYP/6-311++G(d,p) level of theory.

S4. 4 Correlations between the GFN2 method and the CAM-B3LYP/6-311++G(d,p) level of theory

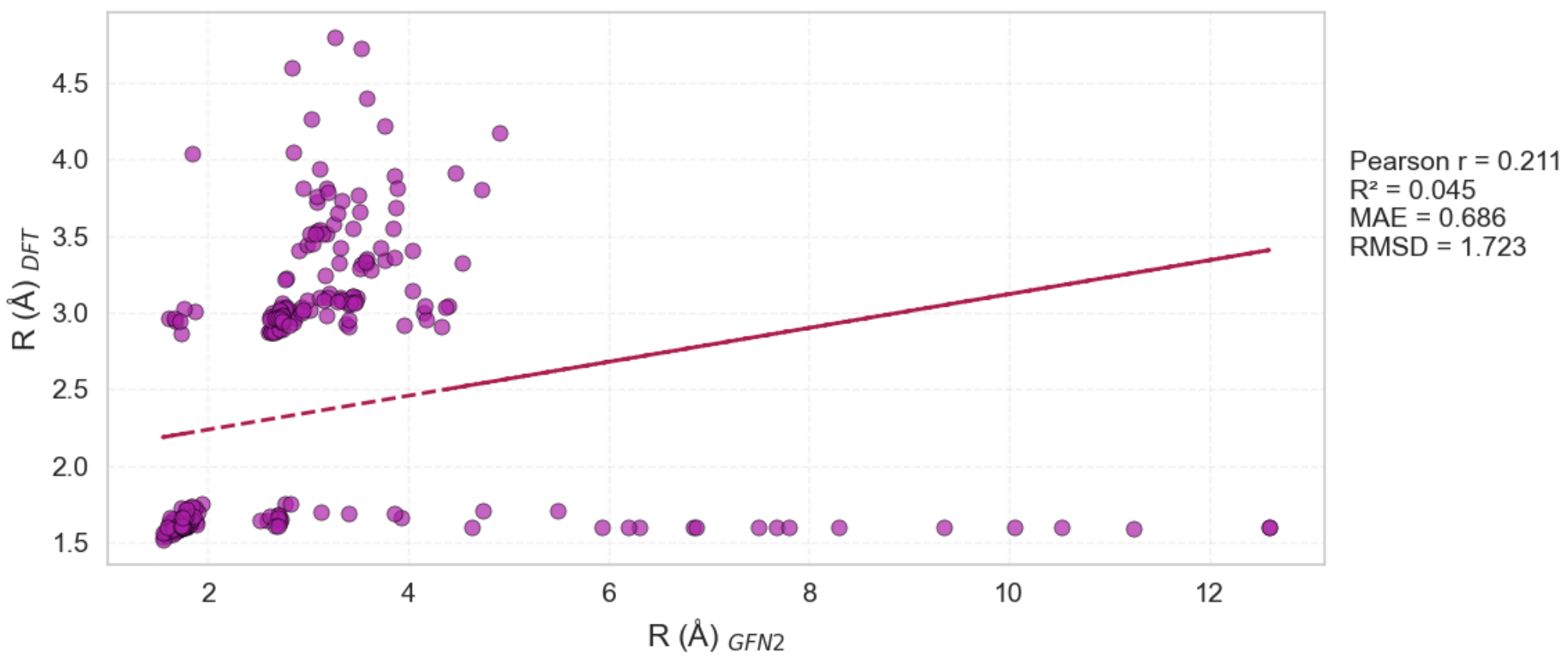

Figure S85: Correlation plot of the bond length R (Å) between values computed with the GFN2 method and those obtained at the DFT CAM-B3LYP/6-311++G(d,p) level of theory.

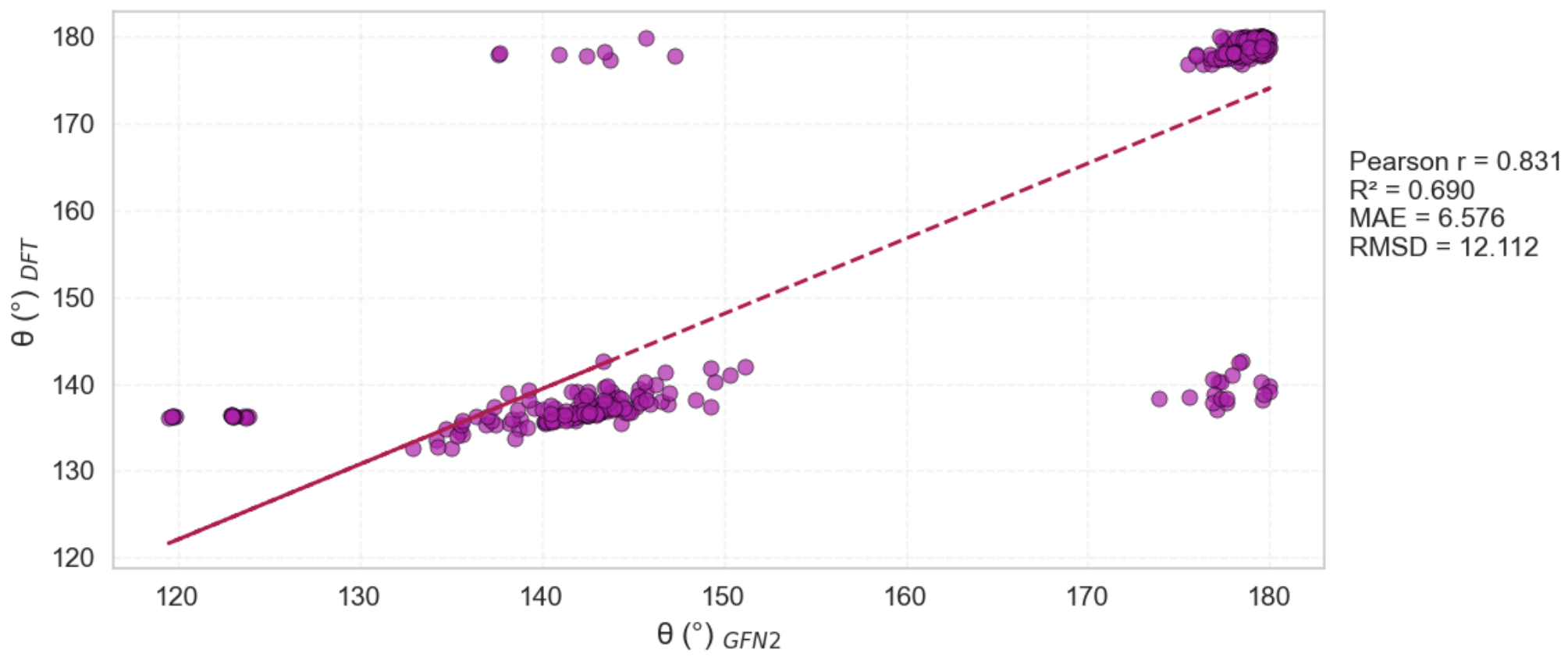

Figure S86: Correlation plot of the bond angle (θ) of the O=C=O between values computed with the GFN2 method and those obtained at the DFT CAM-B3LYP/6-311++G(d,p) level of theory.

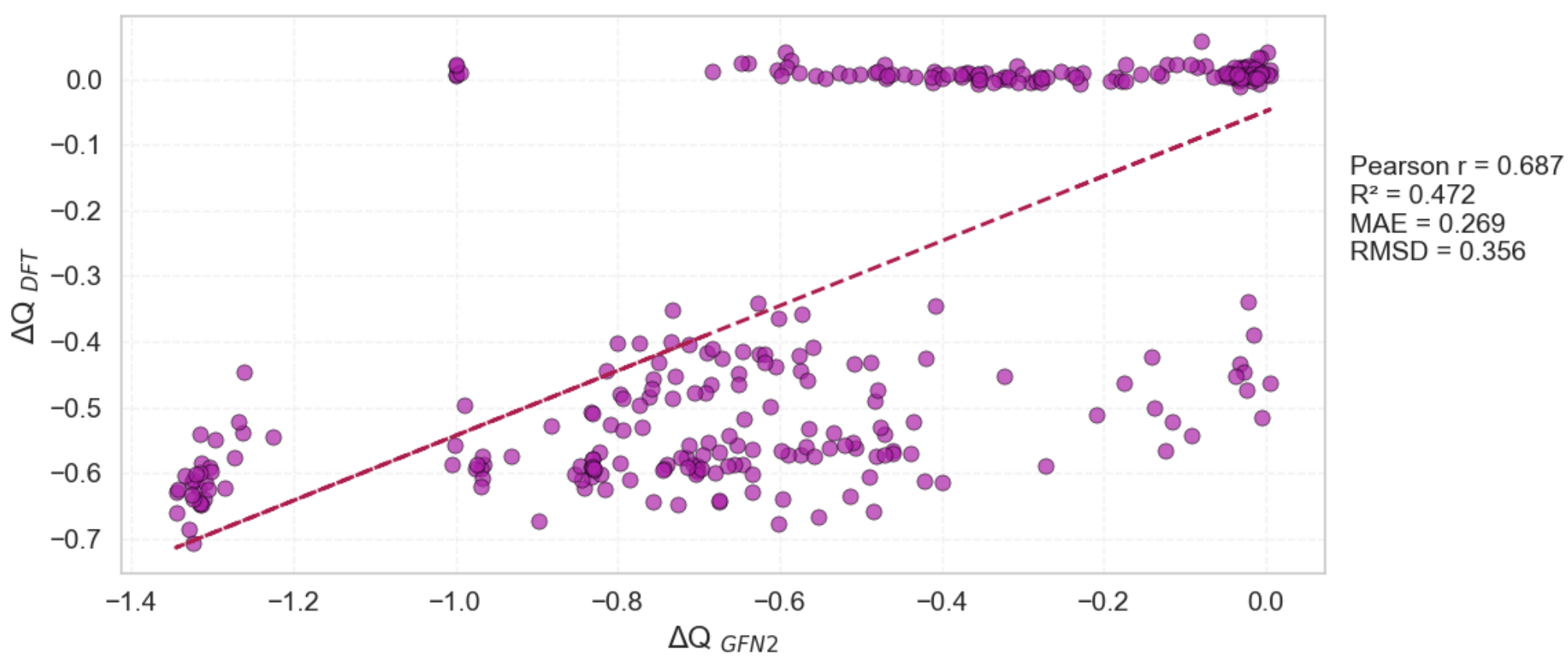

Figure S87: Correlation plot of the charge transfer (ΔQ) between values computed with the GFN2 method and those obtained at the DFT CAM-B3LYP/6-311++G(d,p) level of theory.

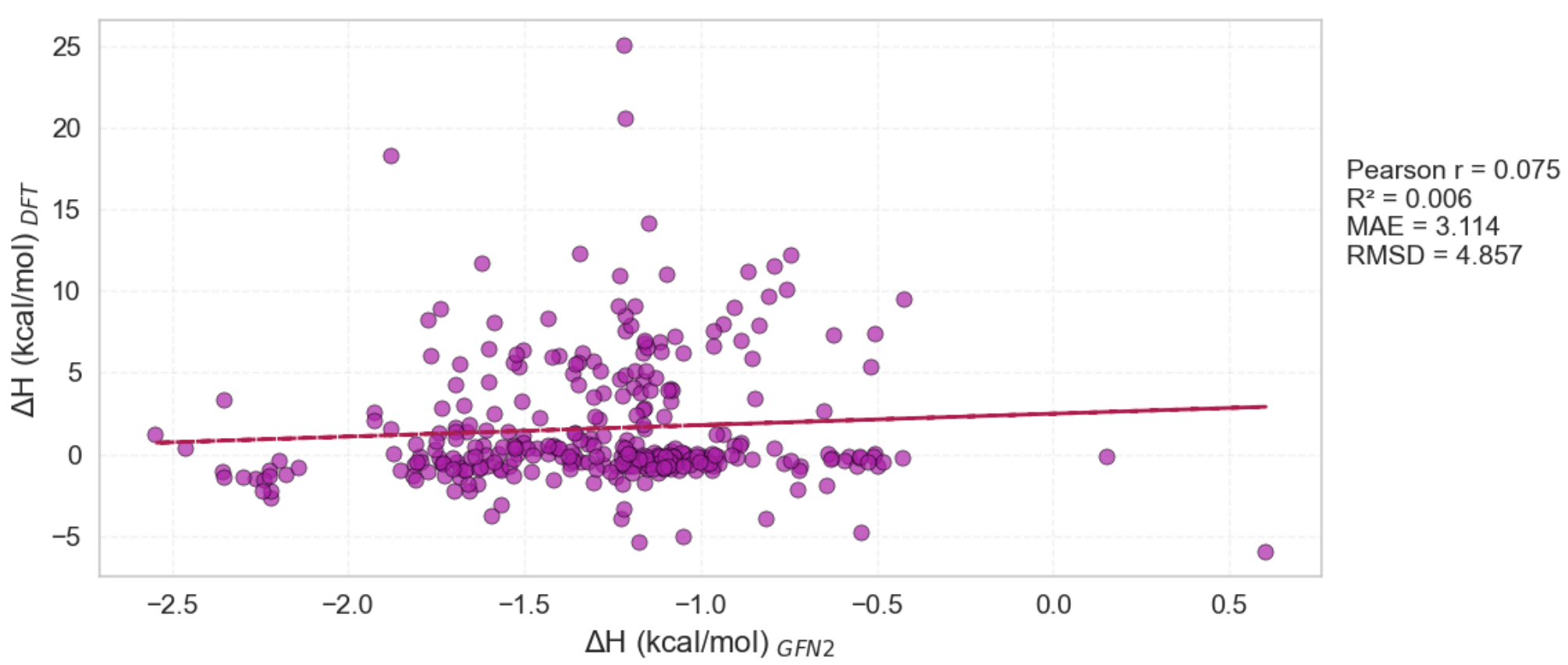

Figure S88: Correlation plot of the enthalpy (ΔH) between values computed with the GFN2 method and those obtained at the DFT CAM-B3LYP/6-311++G(d,p) level of theory.

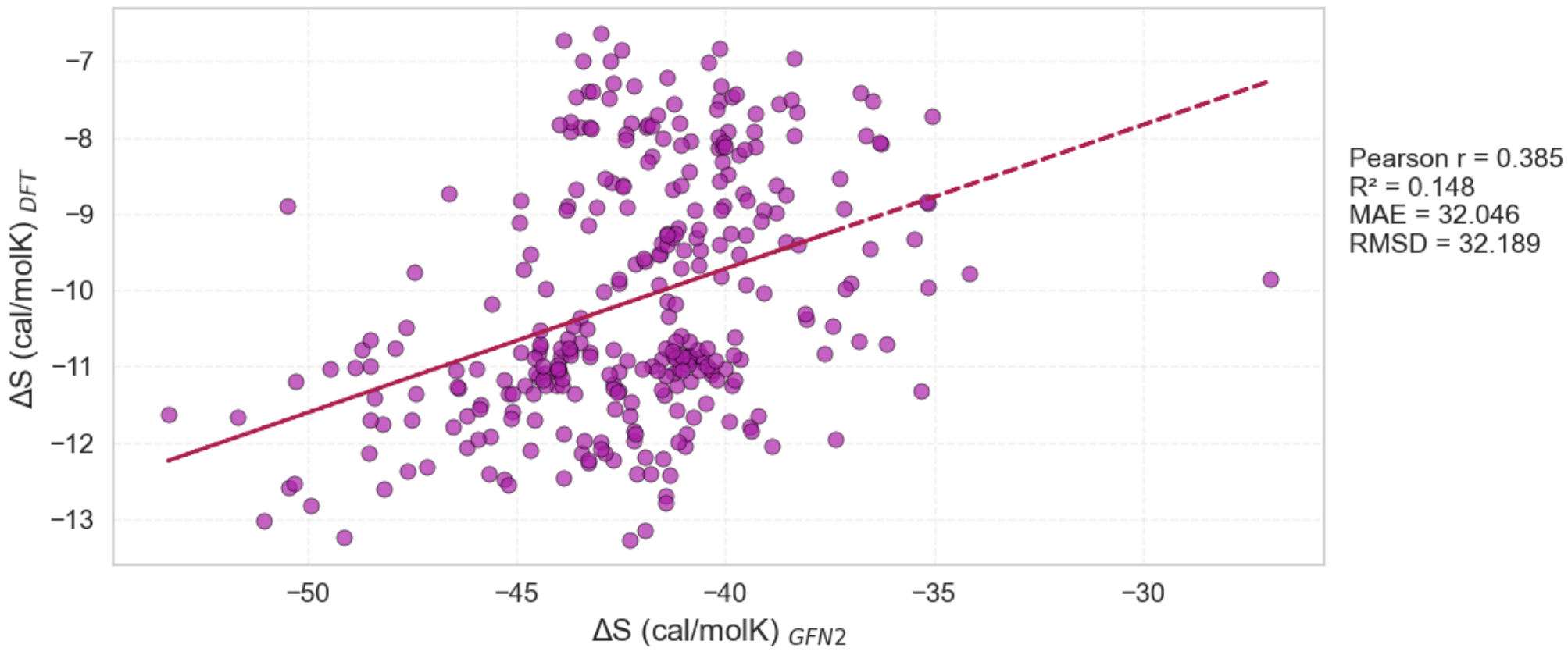

Figure S89: Correlation plot of the the entropy (ΔS) between values computed with the GFN2 method and those obtained at the DFT CAM-B3LYP/6-311++G(d,p) level of theory.

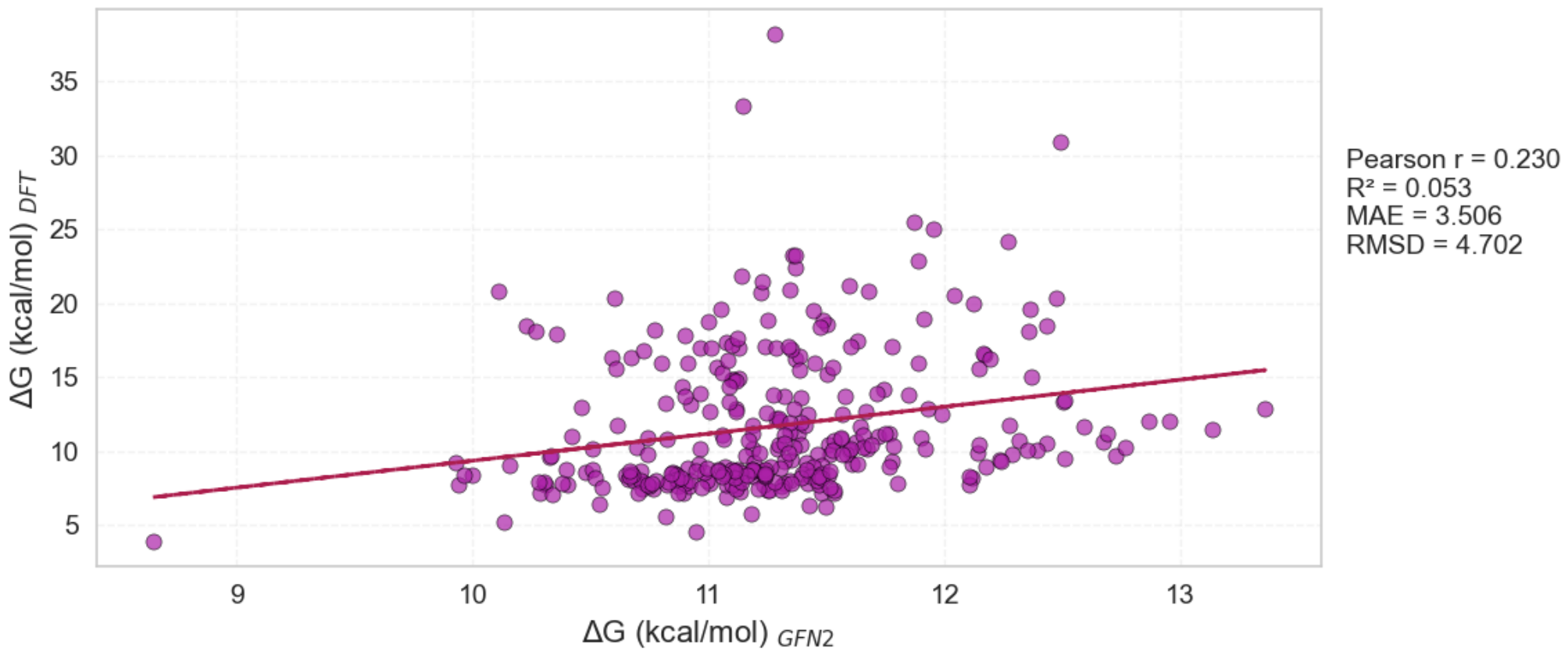

Figure S90: Correlation plot of the the Gibbs free energy (ΔG) between values computed with the GFN2 method and those obtained at the DFT CAM-B3LYP/6-311++G(d,p) level of theory.

S4. 5 Correlations between the MNDO method and the CAM-B3LYP/6-311++G(d,p) level of theory

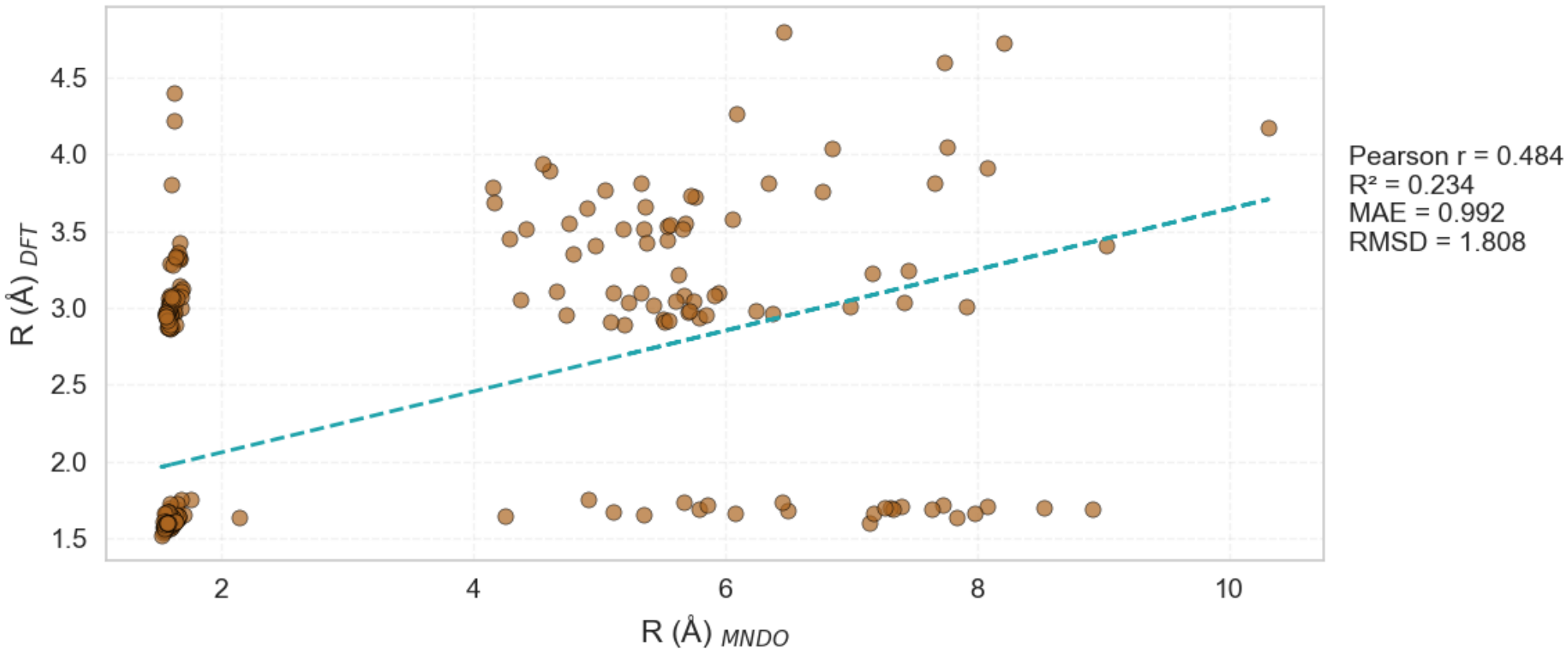

Figure S91: Correlation plot of the bond length R (Å) between values computed with the MNDO method and those obtained at the DFT CAM-B3LYP/6-311++G(d,p) level of theory.

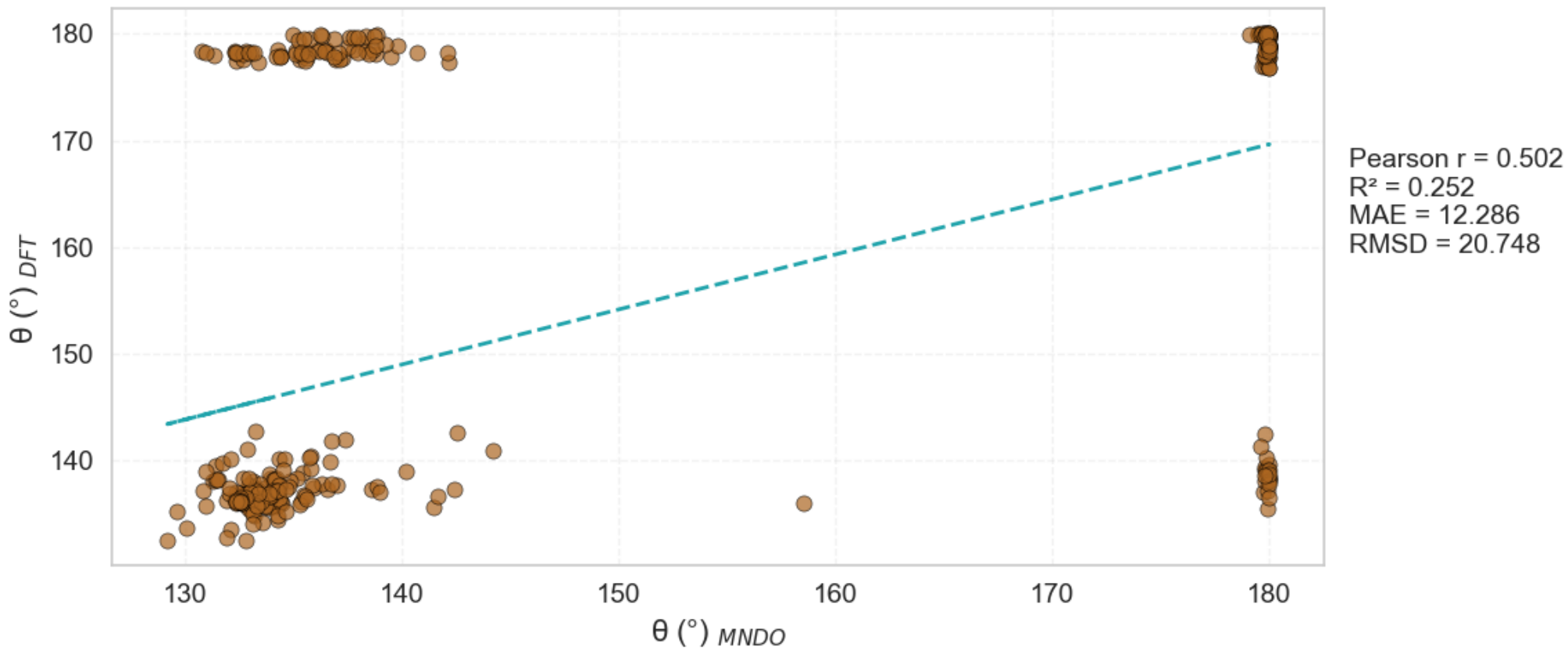

Figure S92: Correlation plot of the bond angle (θ) of the O=C=O between values computed with the MNDO method and those obtained at the DFT CAM-B3LYP/6-311++G(d,p) level of theory.

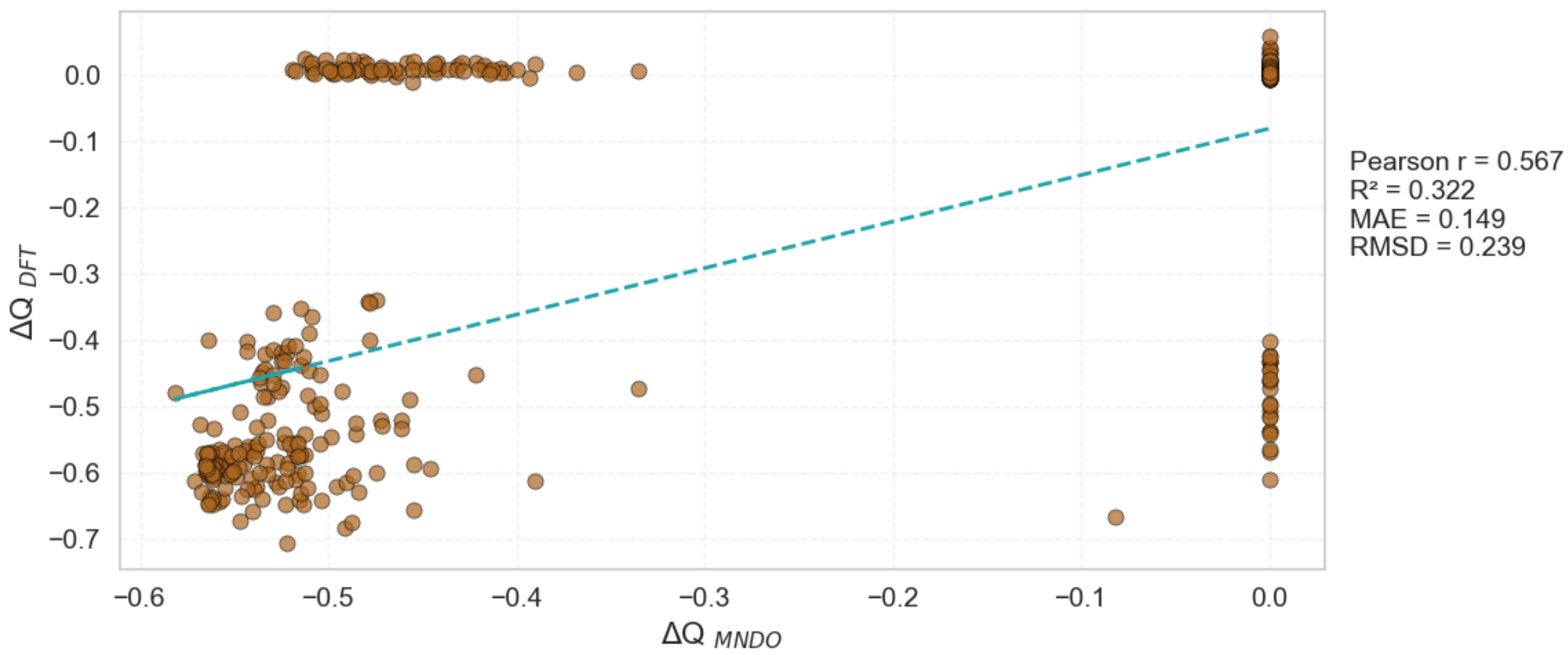

Figure S93: Correlation plot of the charge transfer (ΔQ) between values computed with the MNDO method and those obtained at the DFT CAM-B3LYP/6-311++G(d,p) level of theory.

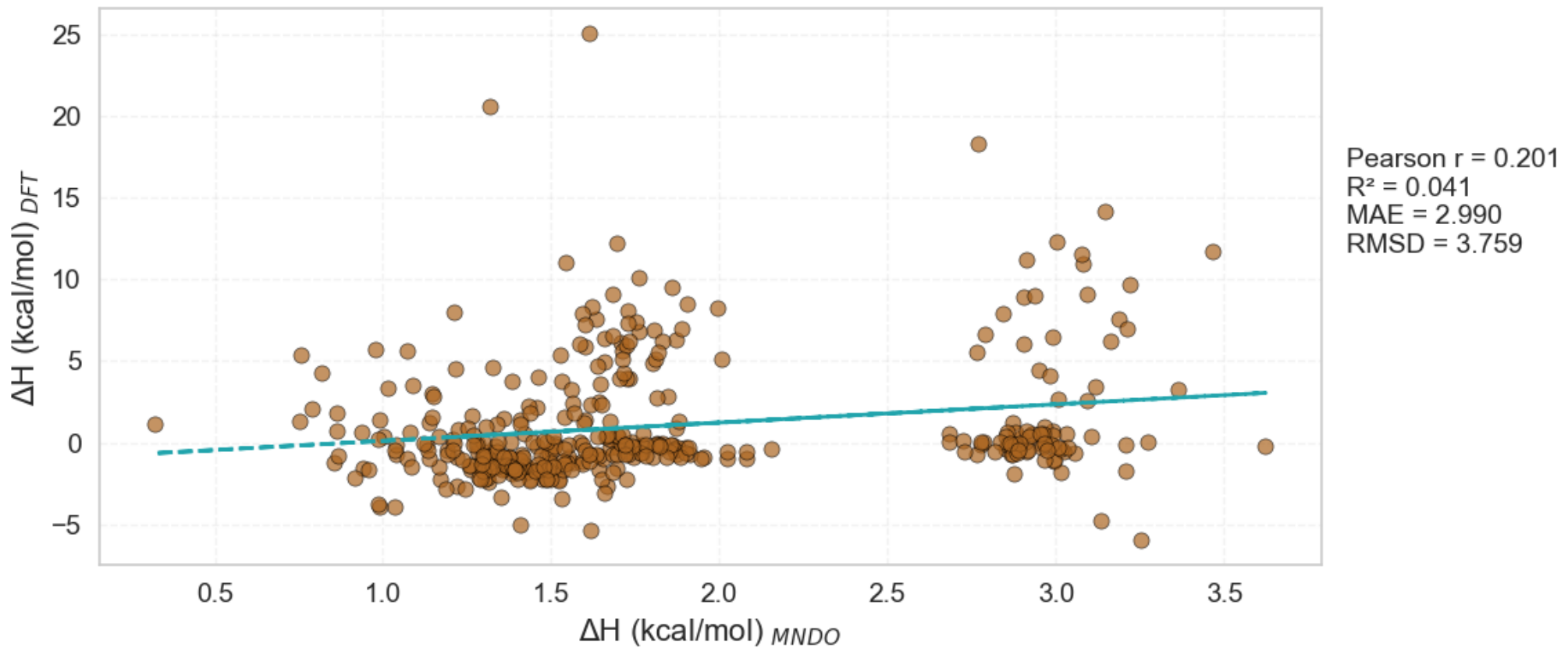

Figure S94: Correlation plot of the enthalpy (ΔH) between values computed with the MNDO method and those obtained at the DFT CAM-B3LYP/6-311++G(d,p) level of theory.

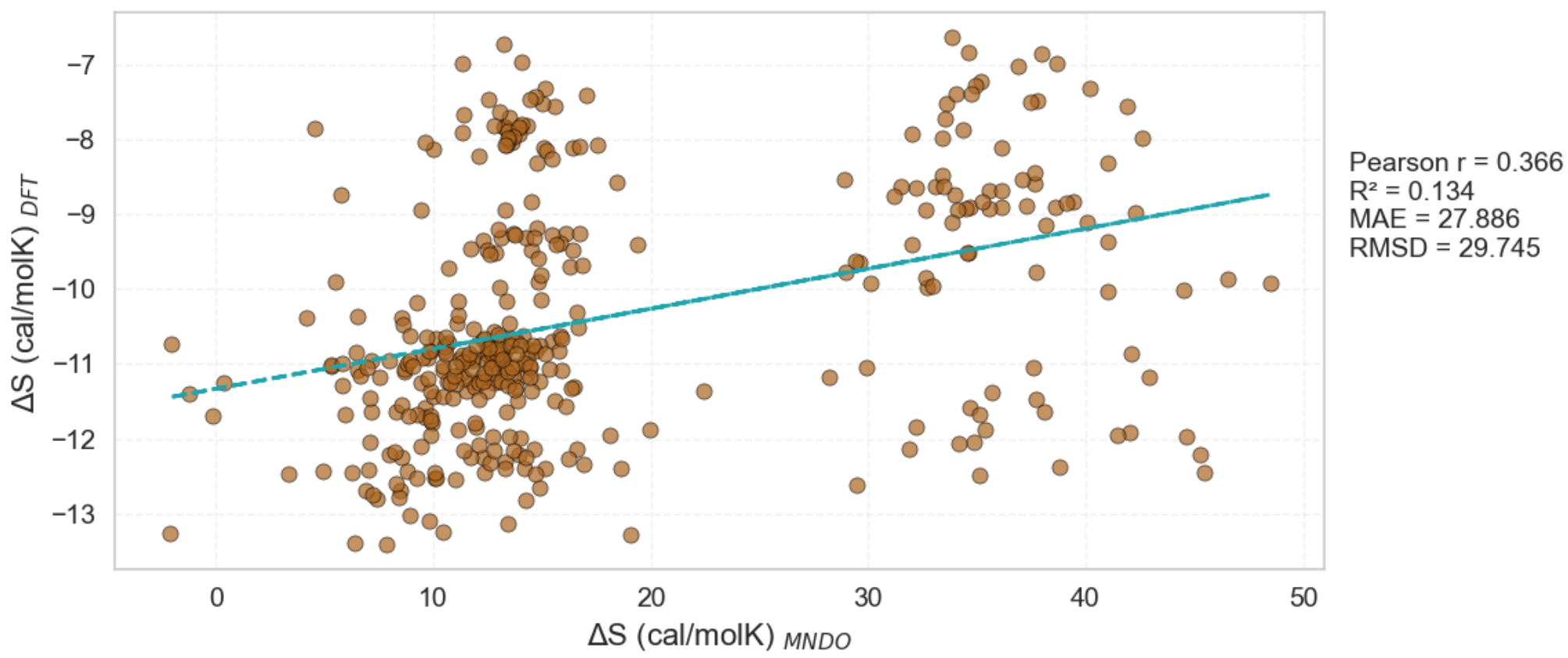

Figure S95: Correlation plot of the the entropy (ΔS) between values computed with the MNDO method and those obtained at the DFT CAM-B3LYP/6-311++G(d,p) level of theory.

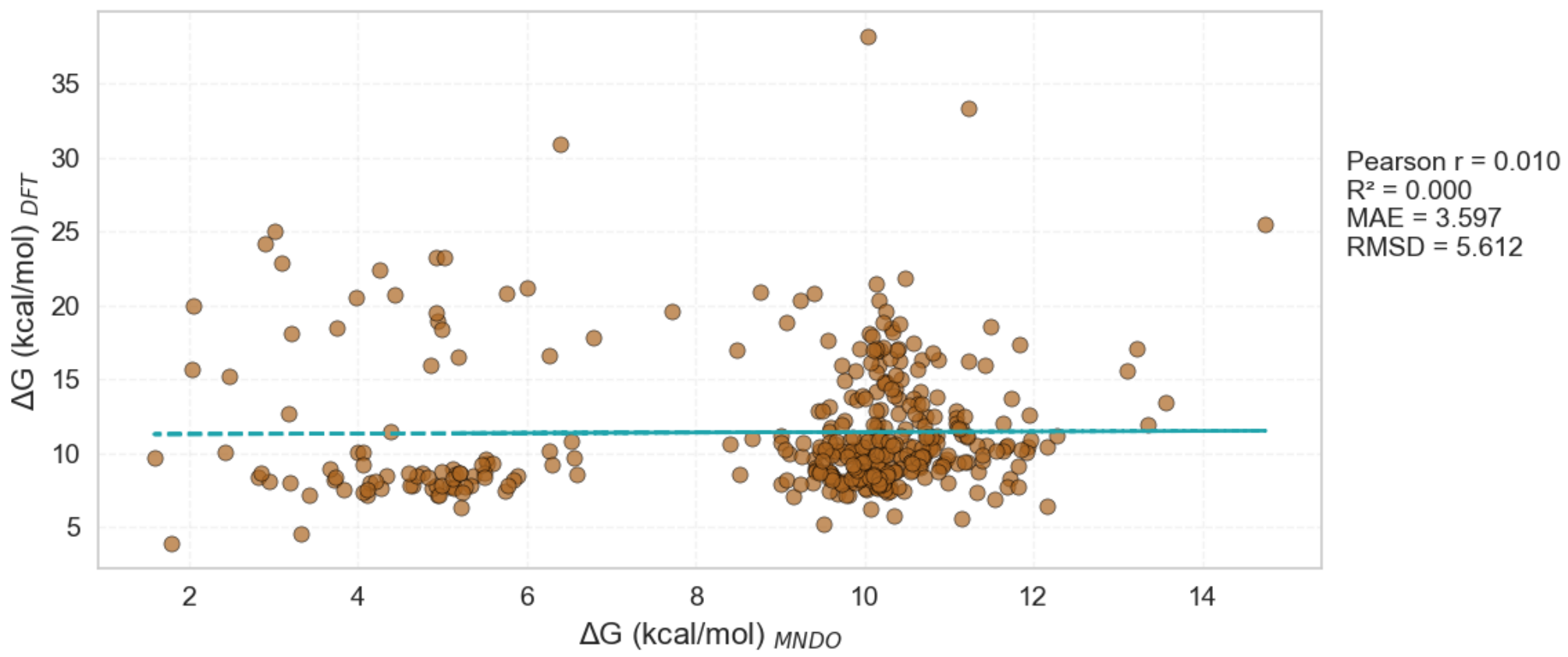

Figure S96: Correlation plot of the the Gibbs free energy (ΔG) between values computed with the MNDO method and those obtained at the DFT CAM-B3LYP/6-311++G(d,p) level of theory.

S4. 6 Correlations between the MNDOD method and the CAM-B3LYP/6-311++G(d,p) level of theory

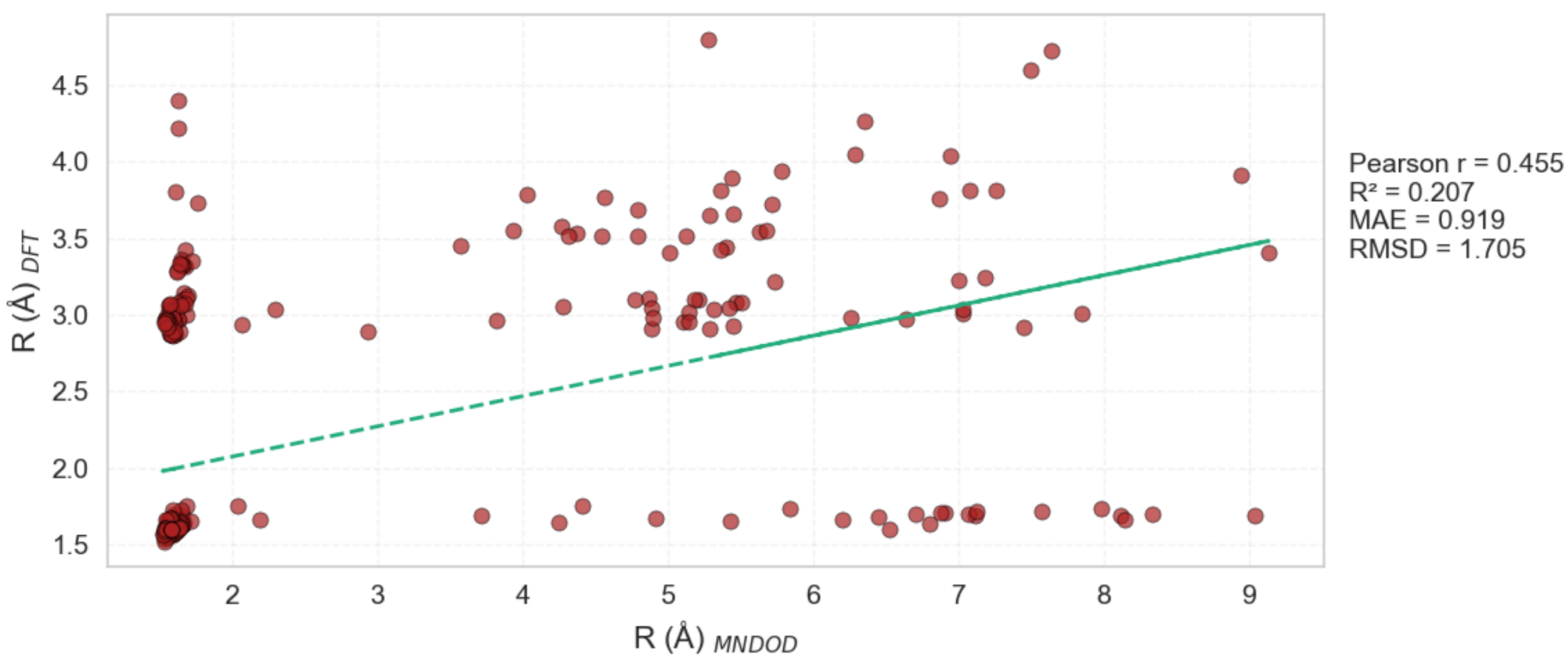

Figure S97: Correlation plot of the bond length R (Å) between values computed with the MNDOD method and those obtained at the DFT CAM-B3LYP/6-311++G(d,p) level of theory.

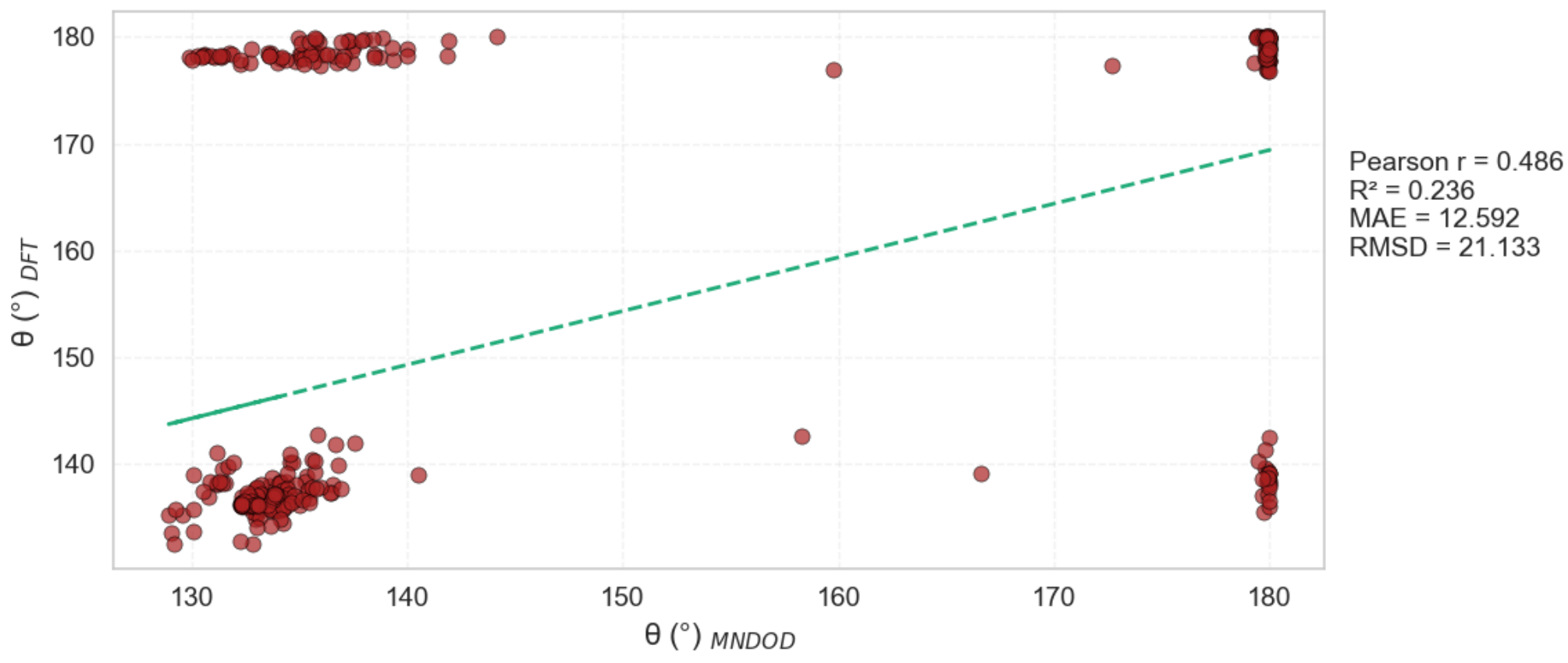

Figure S98: Correlation plot of the bond angle (θ) of the O=C=O between values computed with the MNDOD method and those obtained at the DFT CAM-B3LYP/6-311++G(d,p) level of theory.

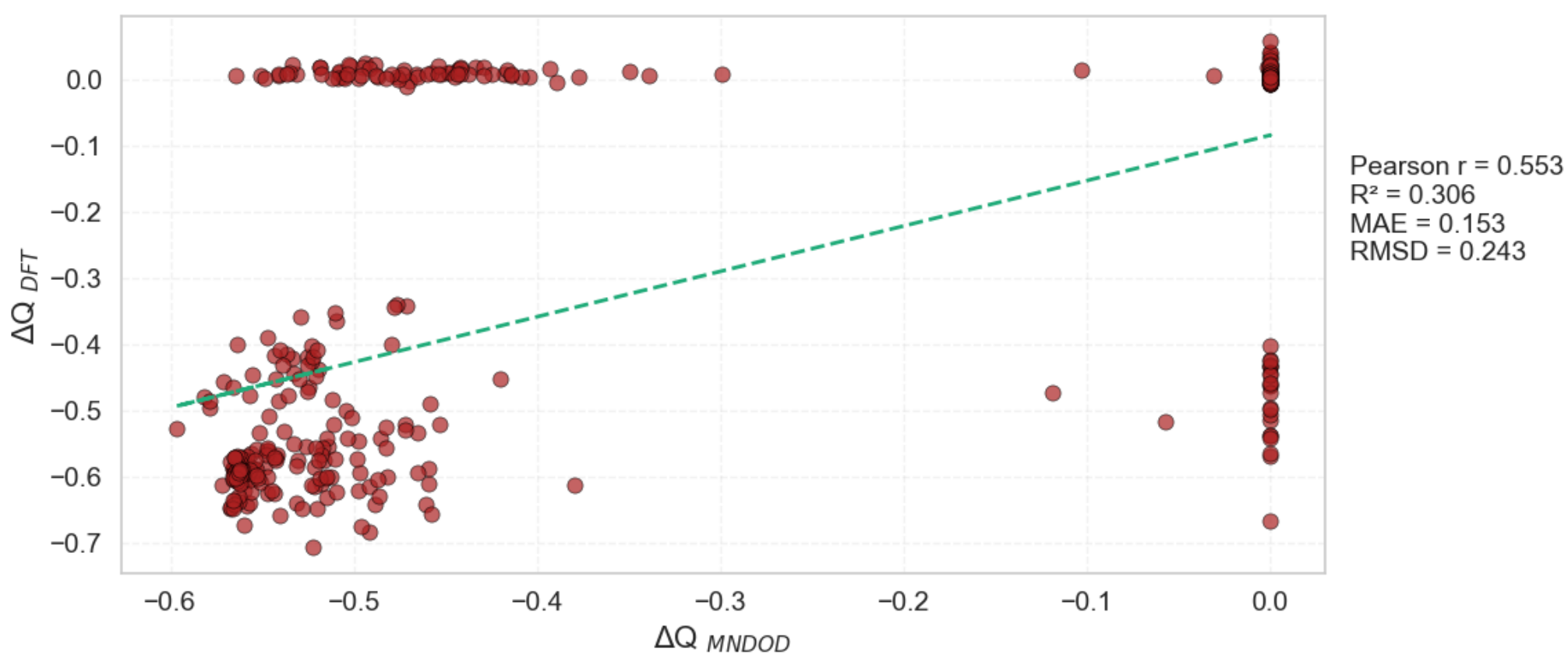

Figure S99: Correlation plot of the charge transfer (ΔQ) between values computed with the MNDOD method and those obtained at the DFT CAM-B3LYP/6-311++G(d,p) level of theory.

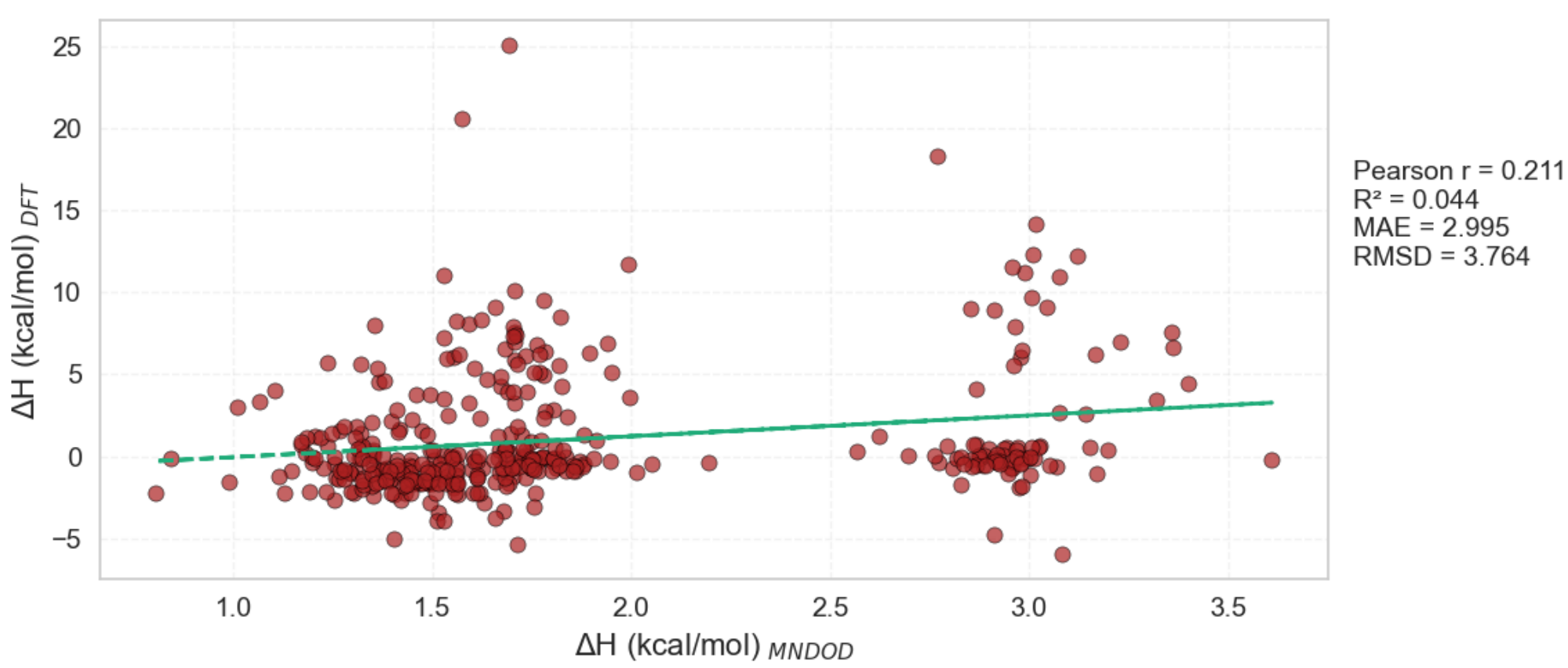

Figure S100: Correlation plot of the enthalpy (ΔH) between values computed with the MNDOD method and those obtained at the DFT CAM-B3LYP/6-311++G(d,p) level of theory.

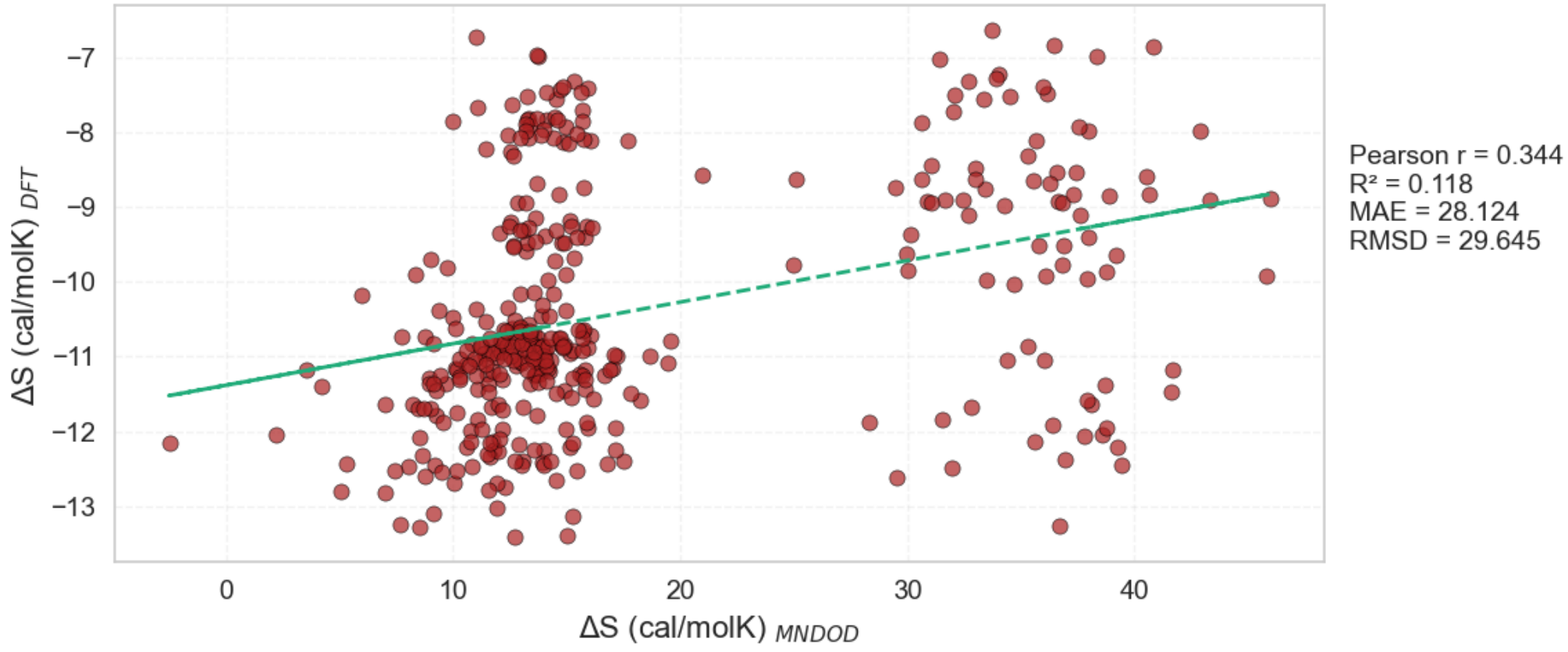

Figure S101: Correlation plot of the the entropy (ΔS) between values computed with the MNDOD method and those obtained at the DFT CAM-B3LYP/6-311++G(d,p) level of theory.

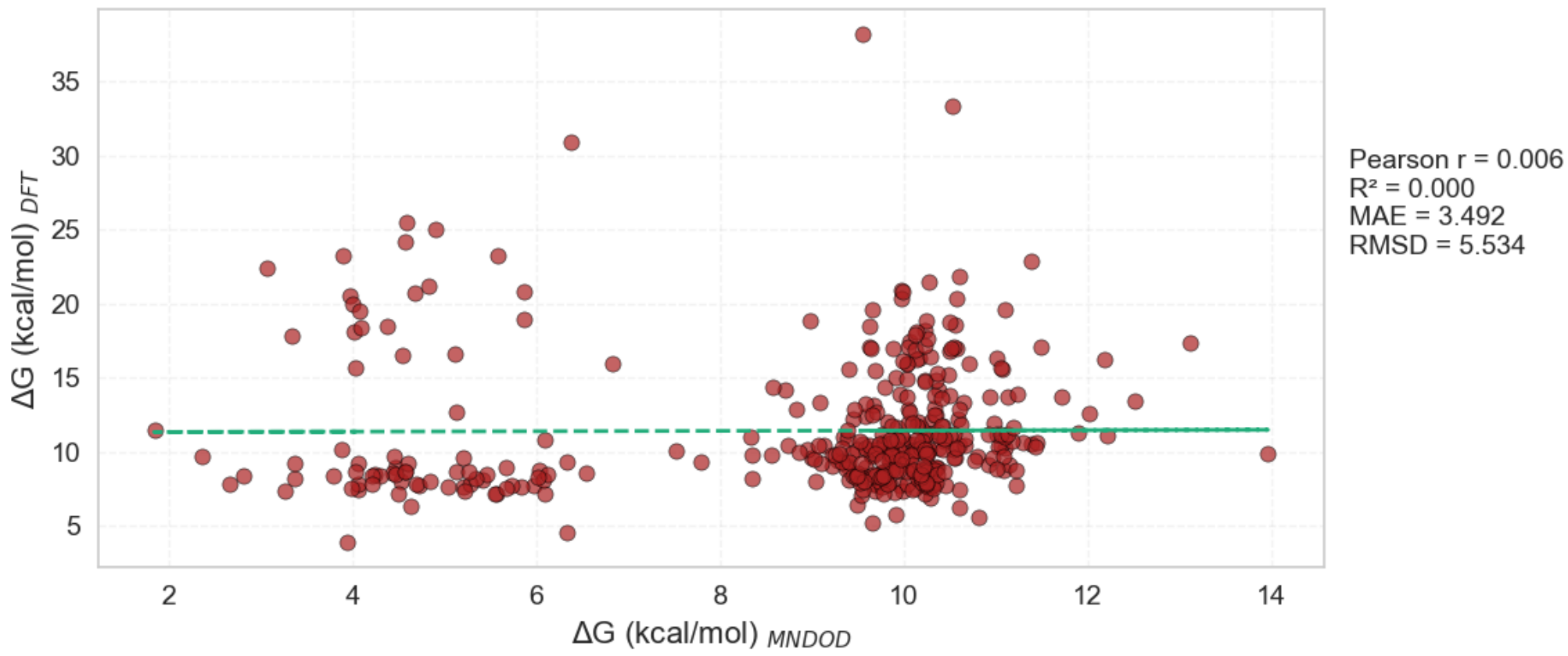

Figure S102: Correlation plot of the the Gibbs free energy (ΔG) between values computed with the MNDOD method and those obtained at the DFT CAM-B3LYP/6-311++G(d,p) level of theory.

S4. 7 Correlations between the PM3 method and the CAM-B3LYP/6-311++G(d,p) level of theory

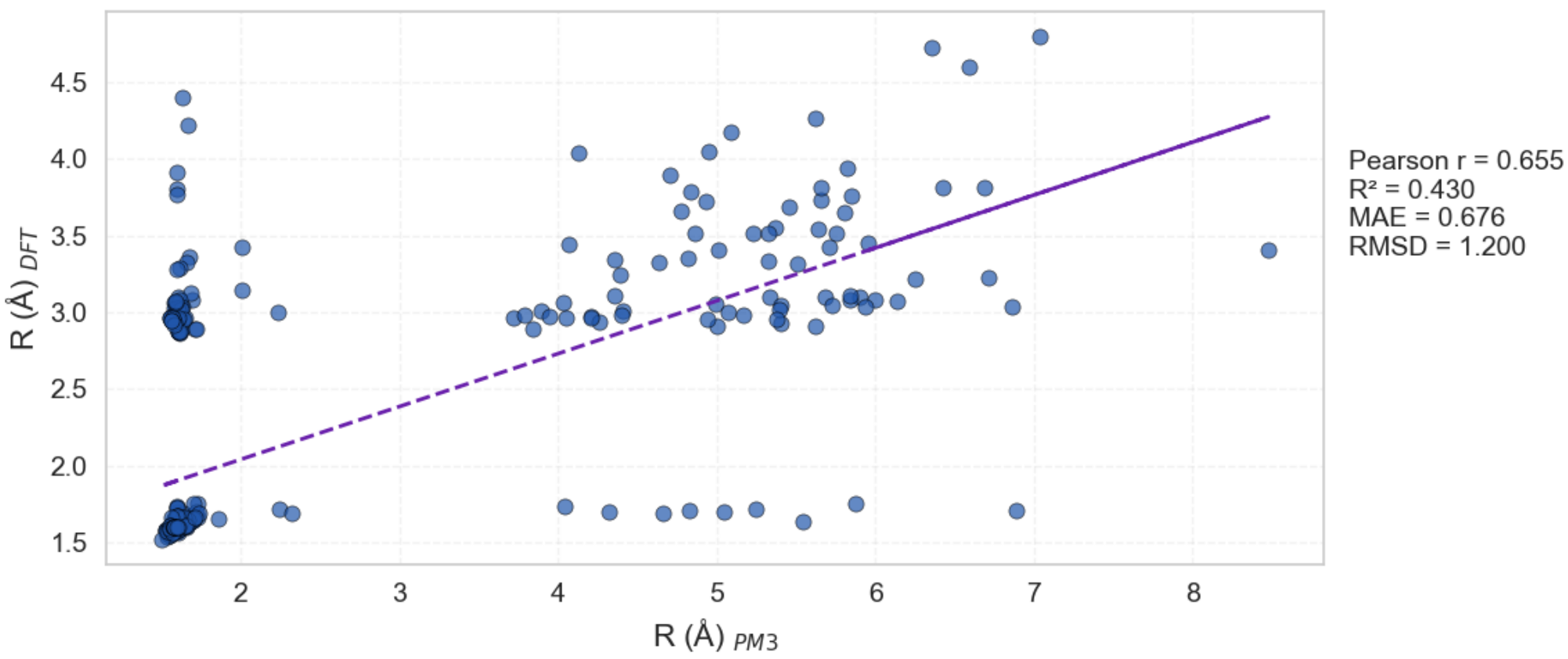

Figure S103: Correlation plot of the bond length R (Å) between values computed with the PM3 method and those obtained at the DFT CAM-B3LYP/6-311++G(d,p) level of theory.

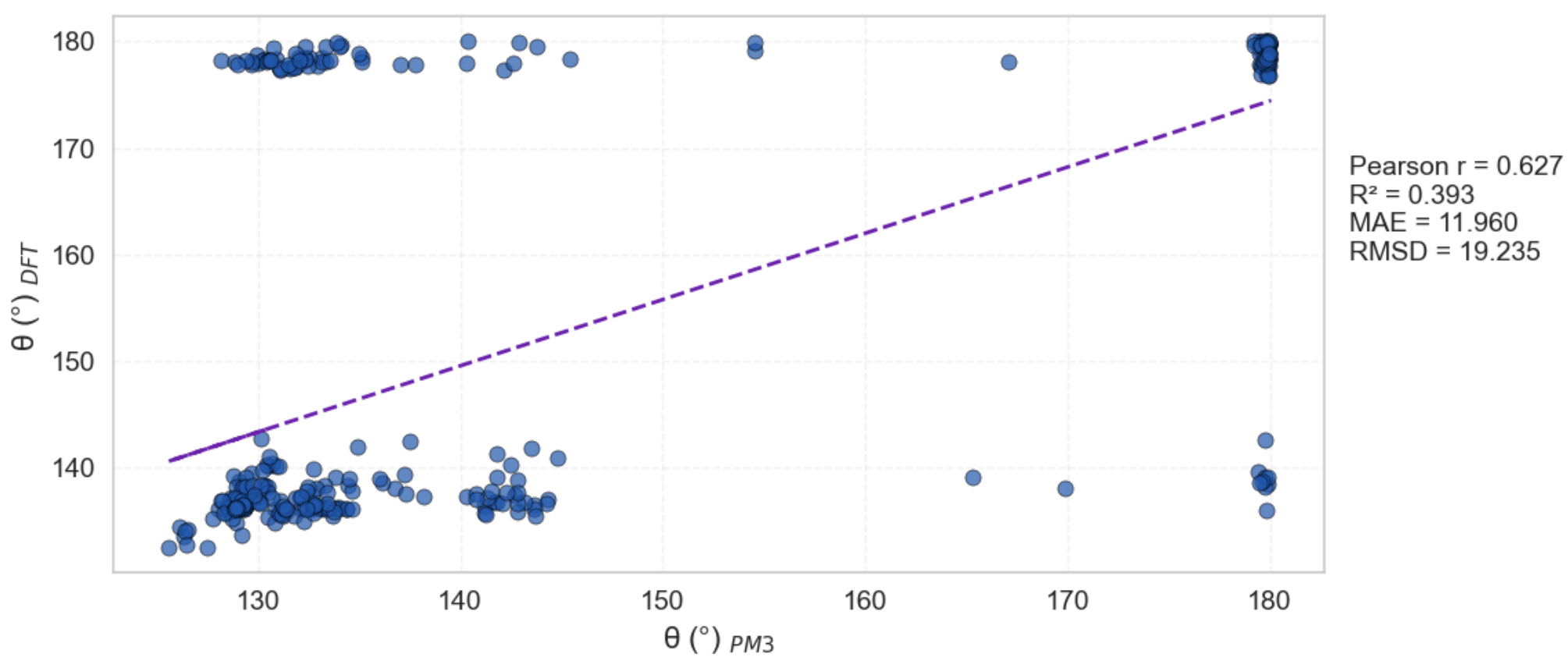

Figure S104: Correlation plot of the bond angle (θ) of the O=C=O between values computed with the PM3 method and those obtained at the DFT CAM-B3LYP/6-311++G(d,p) level of theory.

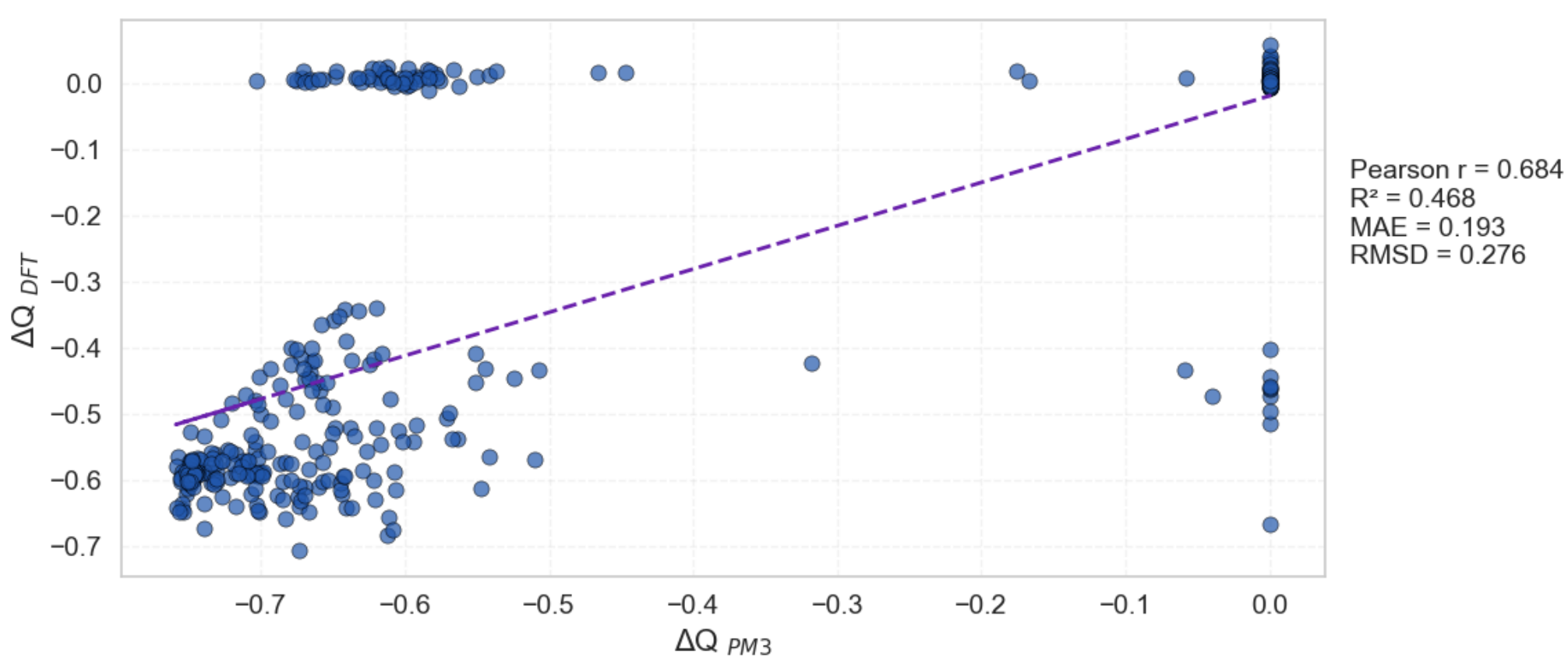

Figure S105: Correlation plot of the charge transfer (ΔQ) between values computed with the PM3 method and those obtained at the DFT CAM-B3LYP/6-311++G(d,p) level of theory.

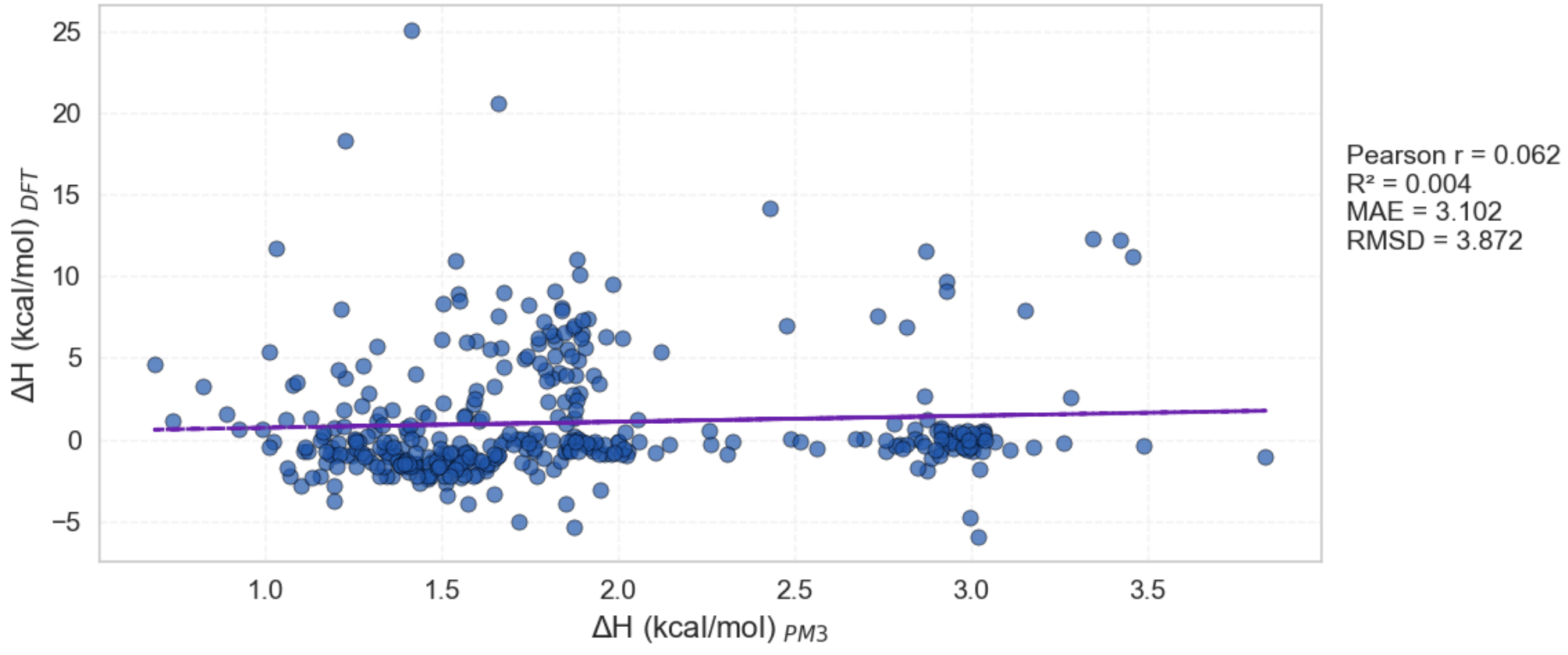

Figure S106: Correlation plot of the enthalpy (ΔH) between values computed with the PM3 method and those obtained at the DFT CAM-B3LYP/6-311++G(d,p) level of theory.

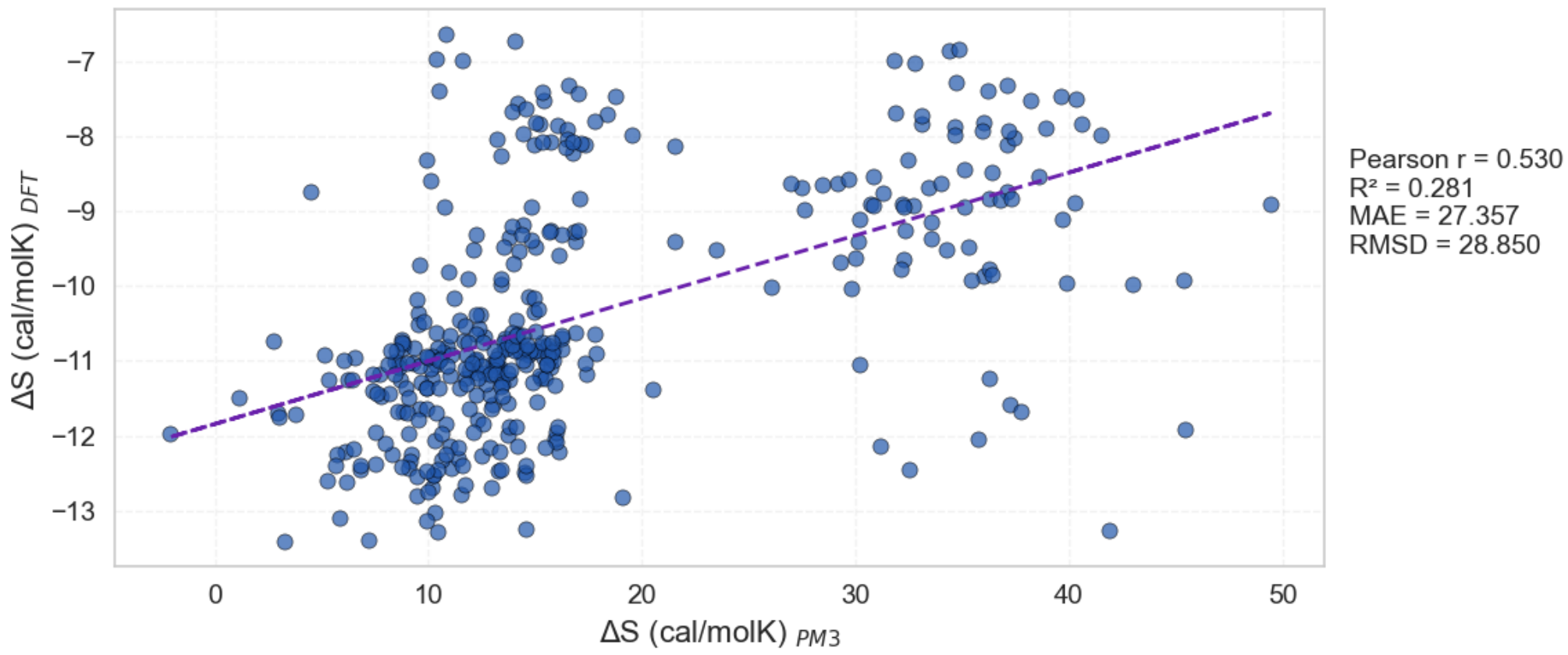

Figure S107: Correlation plot of the the entropy (ΔS) between values computed with the PM3 method and those obtained at the DFT CAM-B3LYP/6-311++G(d,p) level of theory.

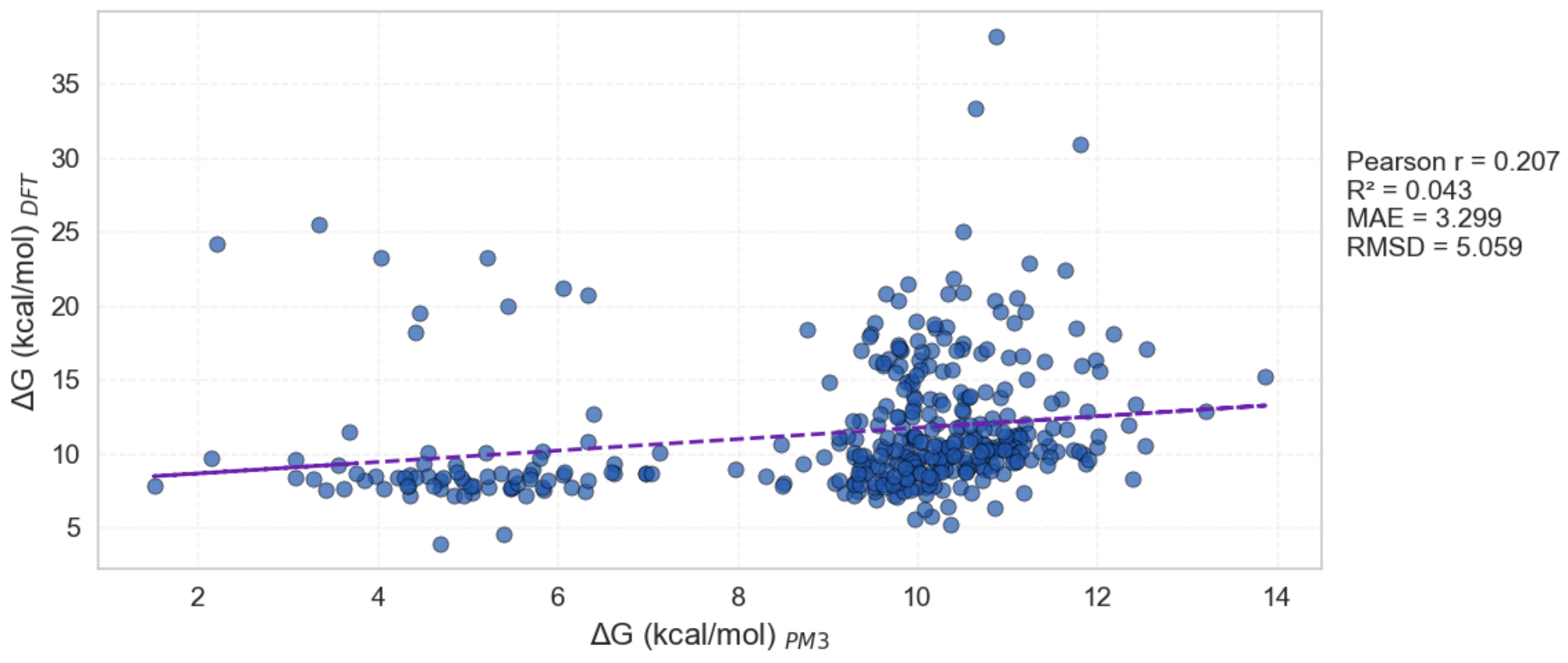

Figure S108: Correlation plot of the the Gibbs free energy (ΔG) between values computed with the PM3 method and those obtained at the DFT CAM-B3LYP/6-311++G(d,p) level of theory.

S4. 8 Correlations between the PM6 method and the CAM-B3LYP/6-311++G(d,p) level of theory

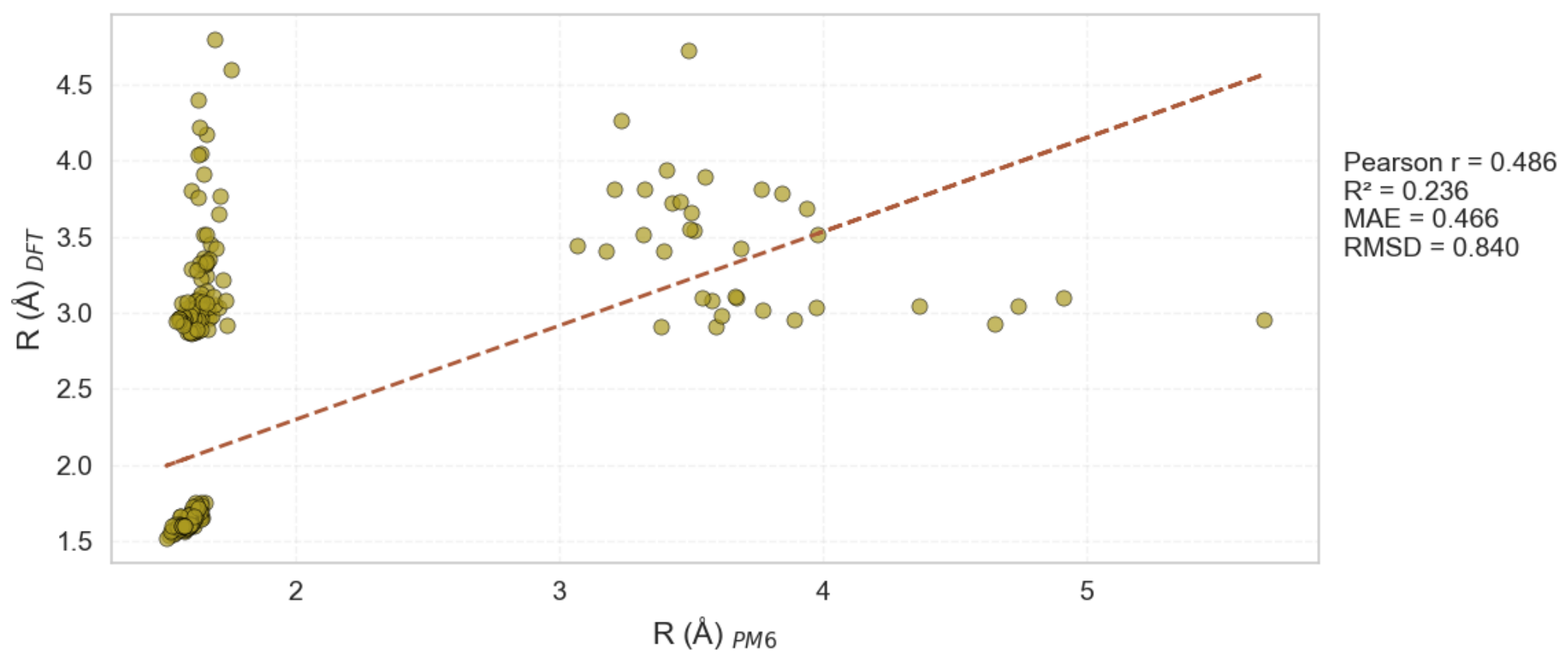

Figure S109: Correlation plot of the bond length R (Å) between values computed with the PM6 method and those obtained at the DFT CAM-B3LYP/6-311++G(d,p) level of theory.

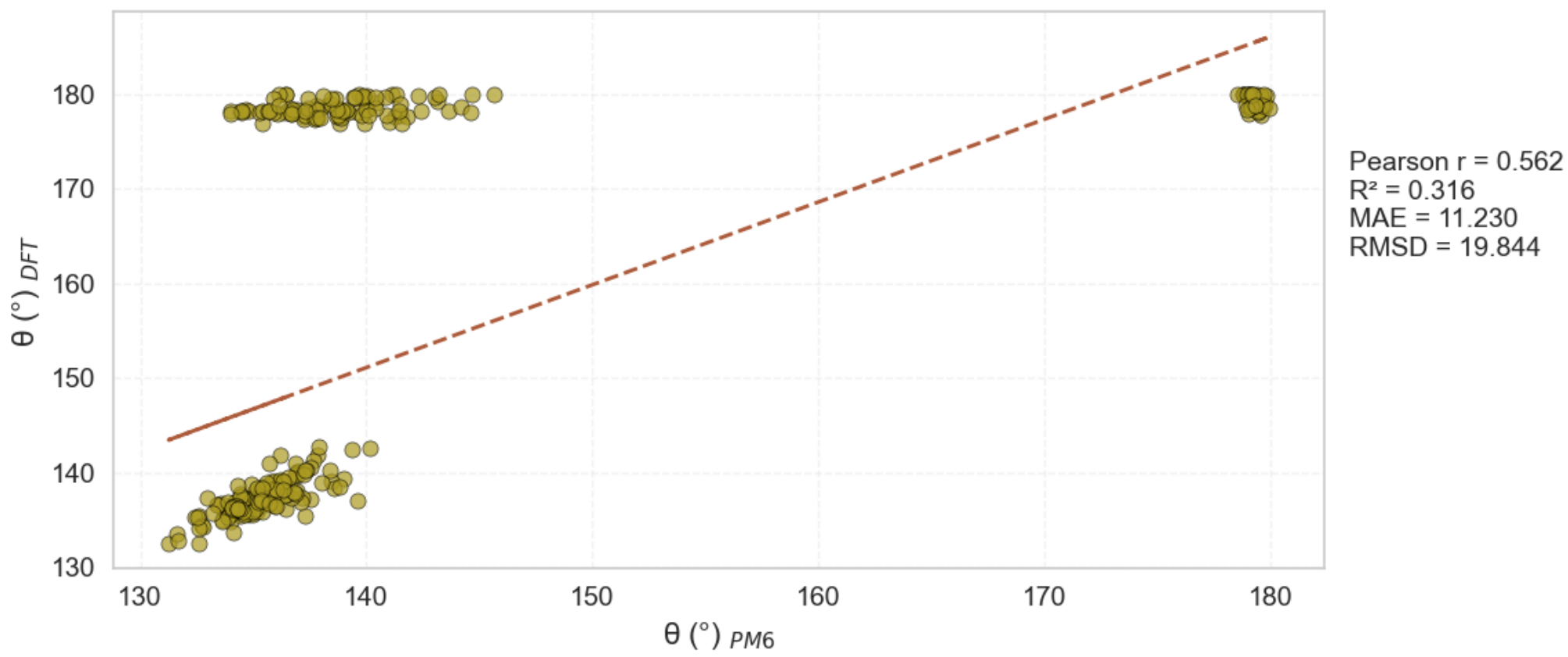

Figure S110: Correlation plot of the bond angle (θ) of the O=C=O between values computed with the PM6 method and those obtained at the DFT CAM-B3LYP/6-311++G(d,p) level of theory.

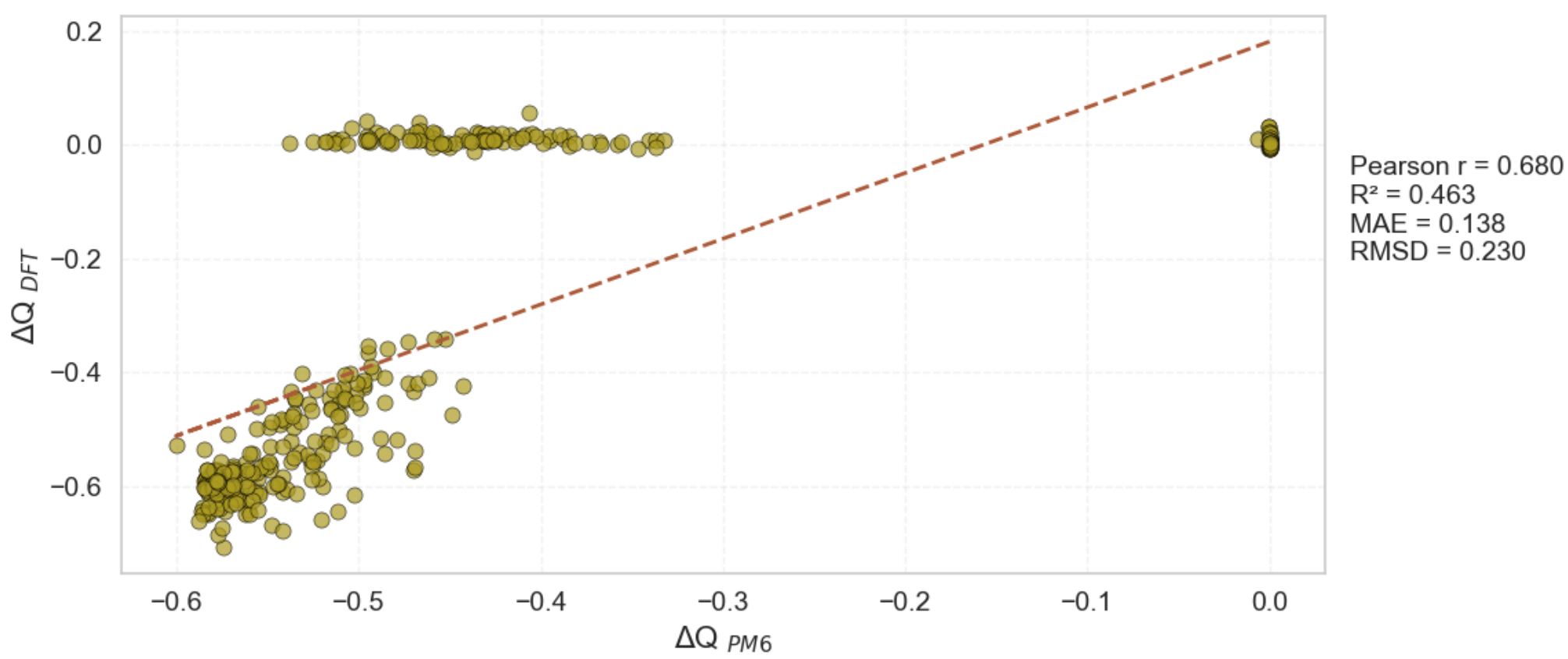

Figure S111: Correlation plot of the charge transfer (ΔQ) between values computed with the PM6 method and those obtained at the DFT CAM-B3LYP/6-311++G(d,p) level of theory.

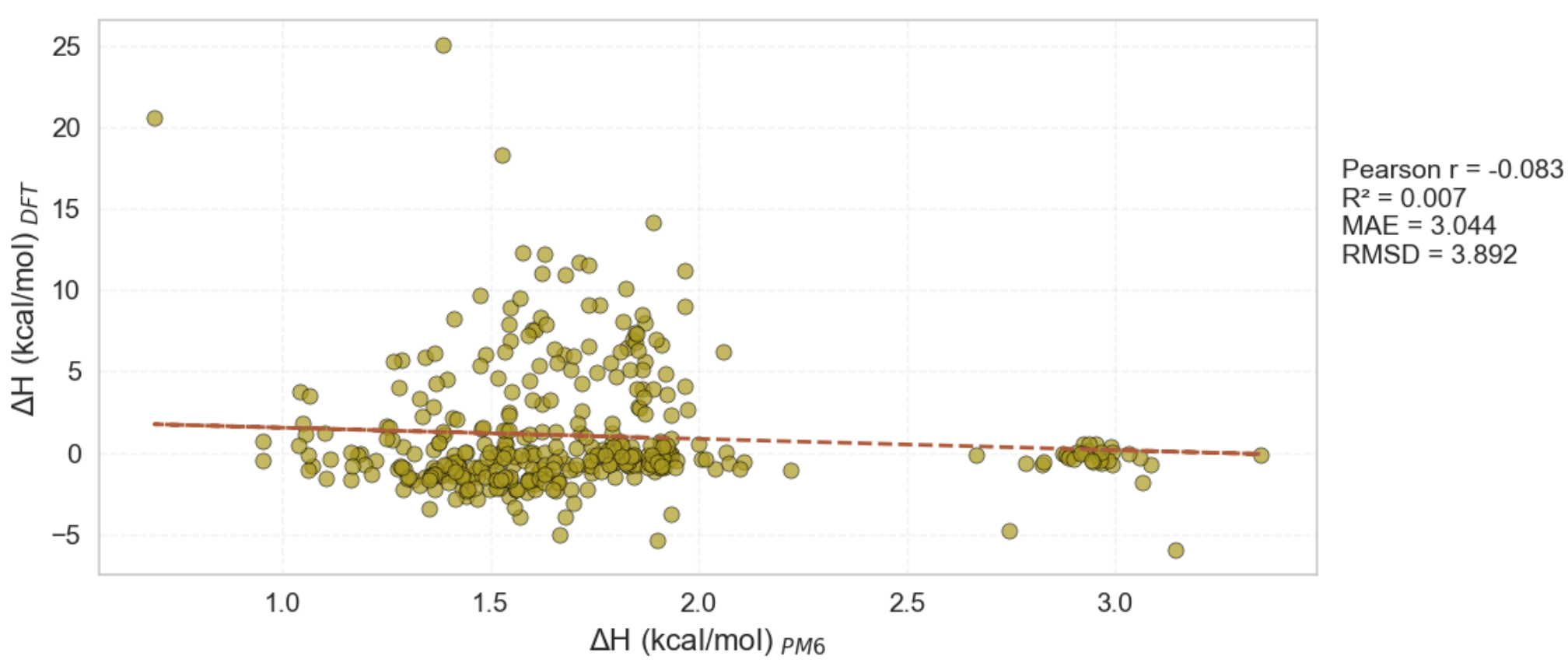

Figure S112: Correlation plot of the enthalpy (ΔH) between values computed with the PM6 method and those obtained at the DFT CAM-B3LYP/6-311++G(d,p) level of theory.

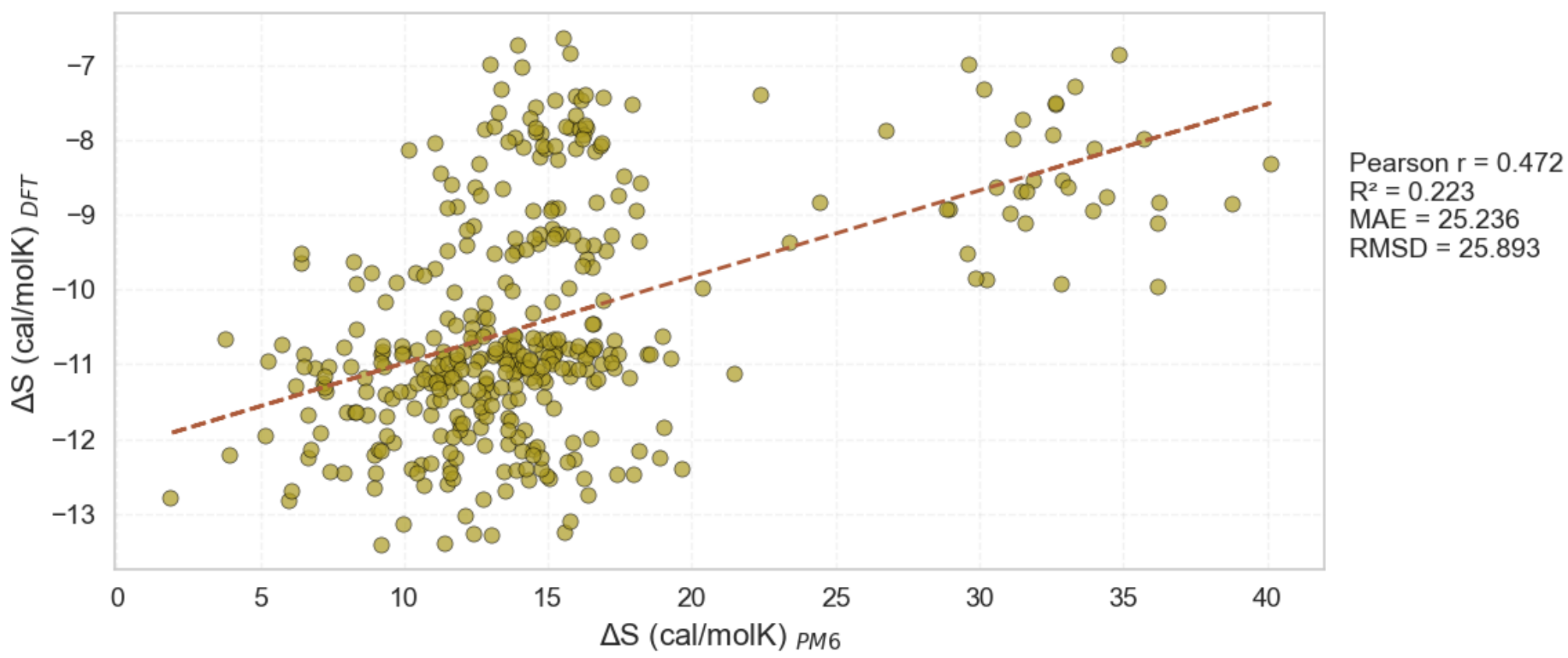

Figure S113: Correlation plot of the the entropy (ΔS) between values computed with the PM6 method and those obtained at the DFT CAM-B3LYP/6-311++G(d,p) level of theory.

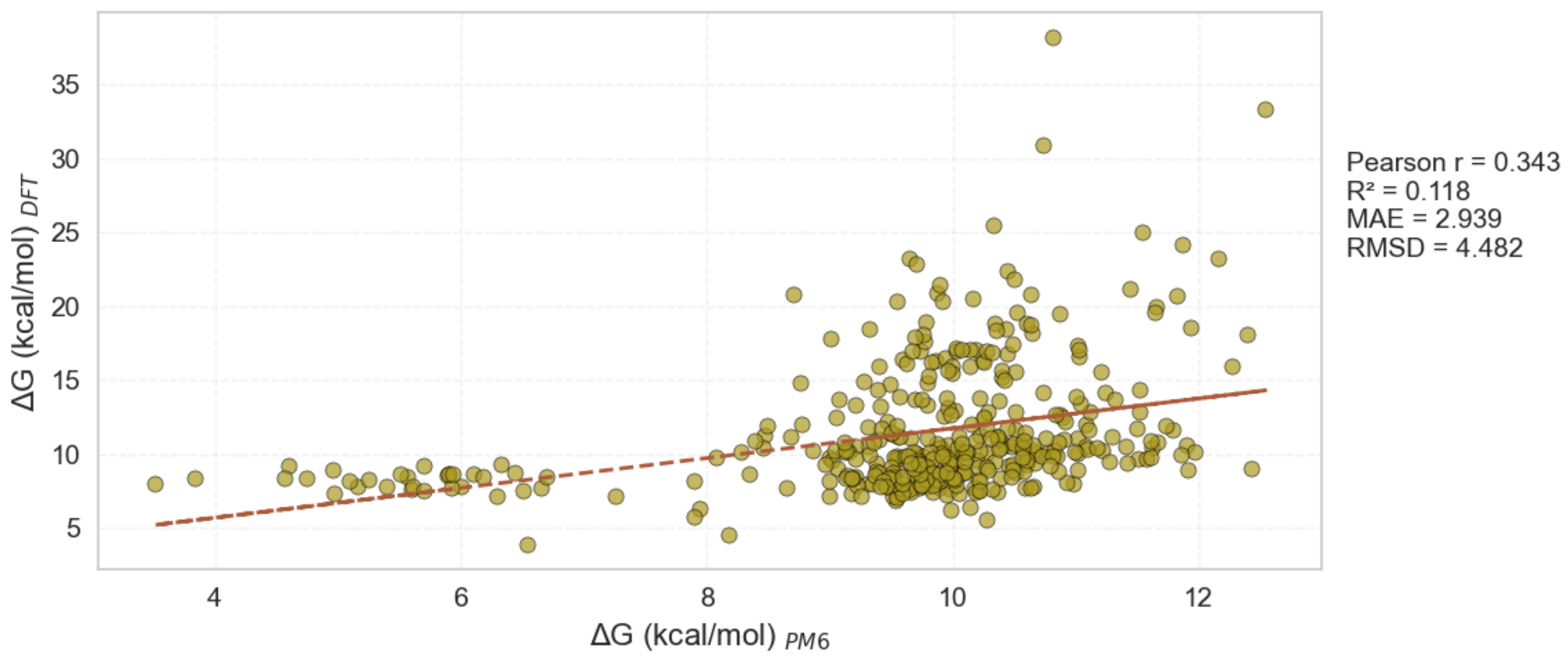

Figure S114: Correlation plot of the the Gibbs free energy (ΔG) between values computed with the PM6 method and those obtained at the DFT CAM-B3LYP/6-311++G(d,p) level of theory.

S4. 9 Correlations between the PM7 method and the CAM-B3LYP/6-311++G(d,p) level of theory

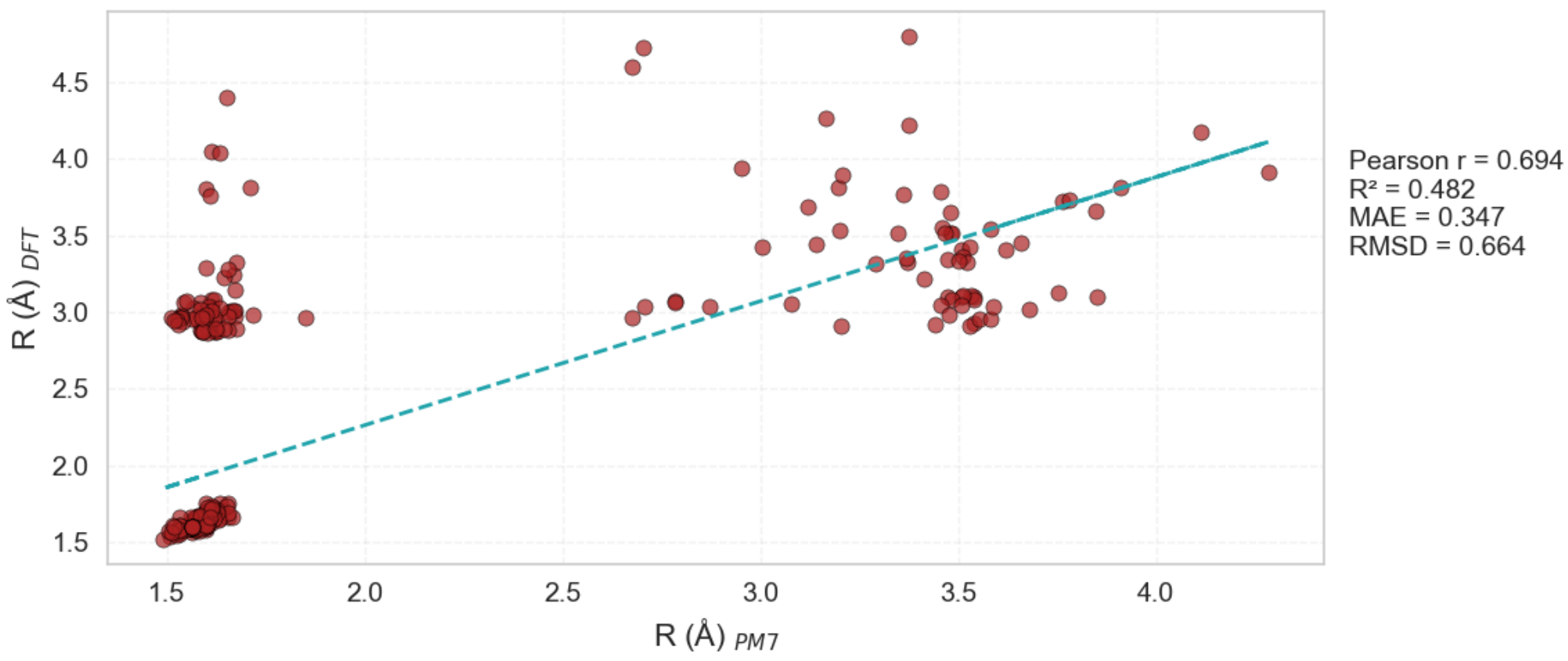

Figure S115: Correlation plot of the bond length R (Å) between values computed with the PM7 method and those obtained at the DFT CAM-B3LYP/6-311++G(d,p) level of theory.

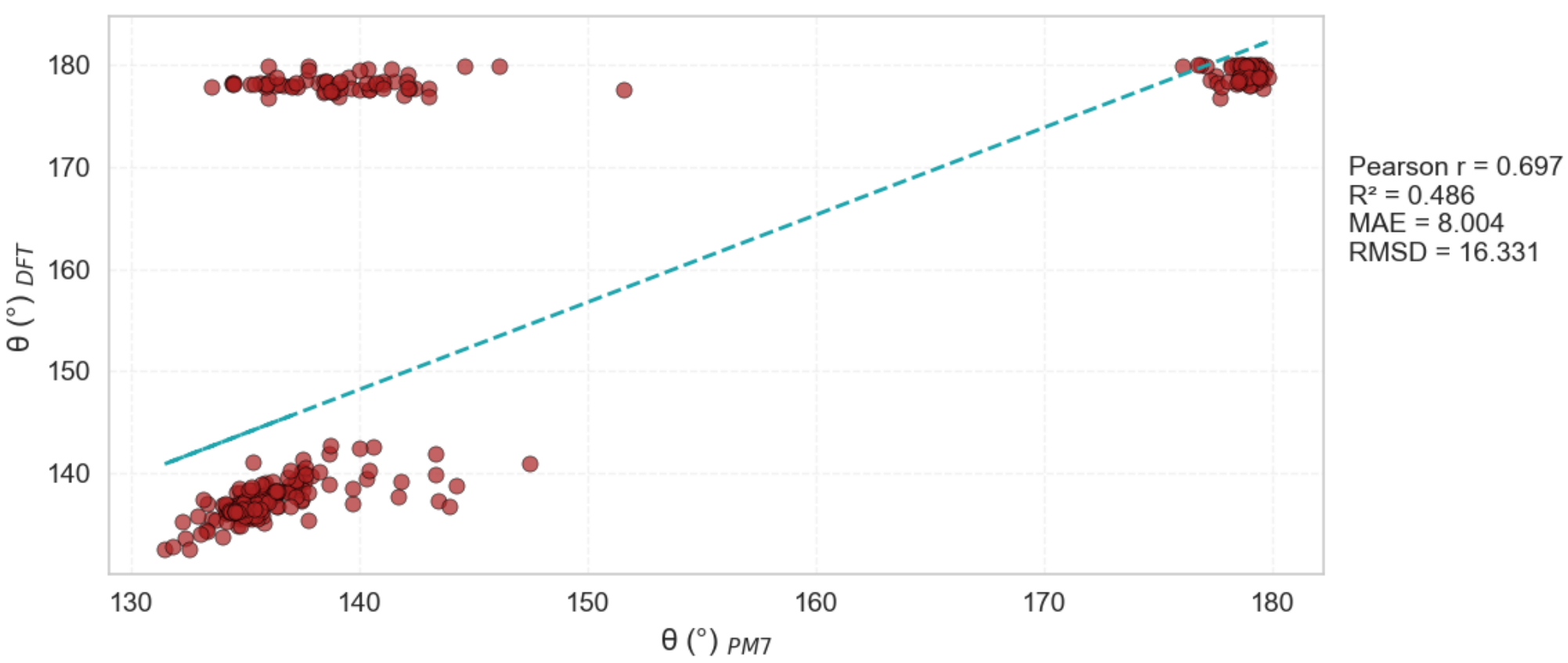

Figure S116: Correlation plot of the bond angle (θ) of the O=C=O between values computed with the PM7 method and those obtained at the DFT CAM-B3LYP/6-311++G(d,p) level of theory.

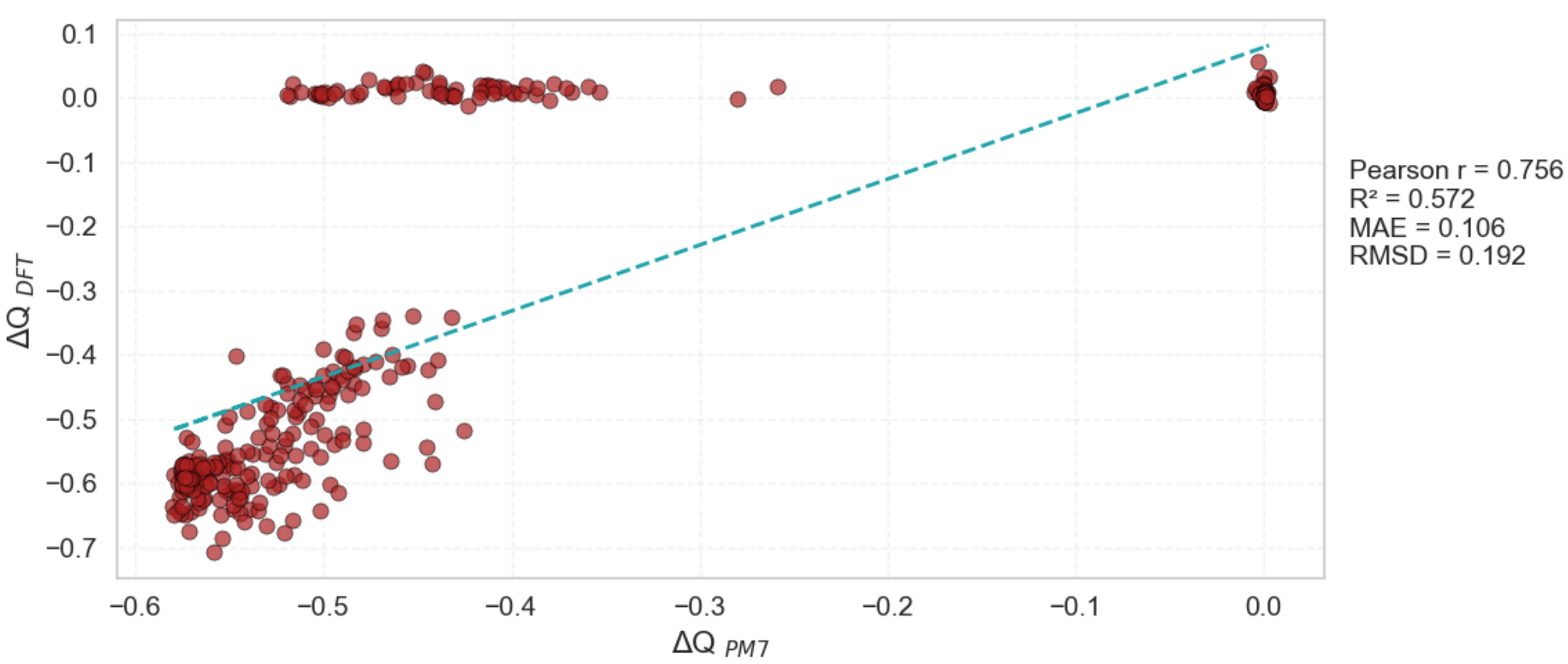

Figure S117: Correlation plot of the charge transfer (ΔQ) between values computed with the PM7 method and those obtained at the DFT CAM-B3LYP/6-311++G(d,p) level of theory.

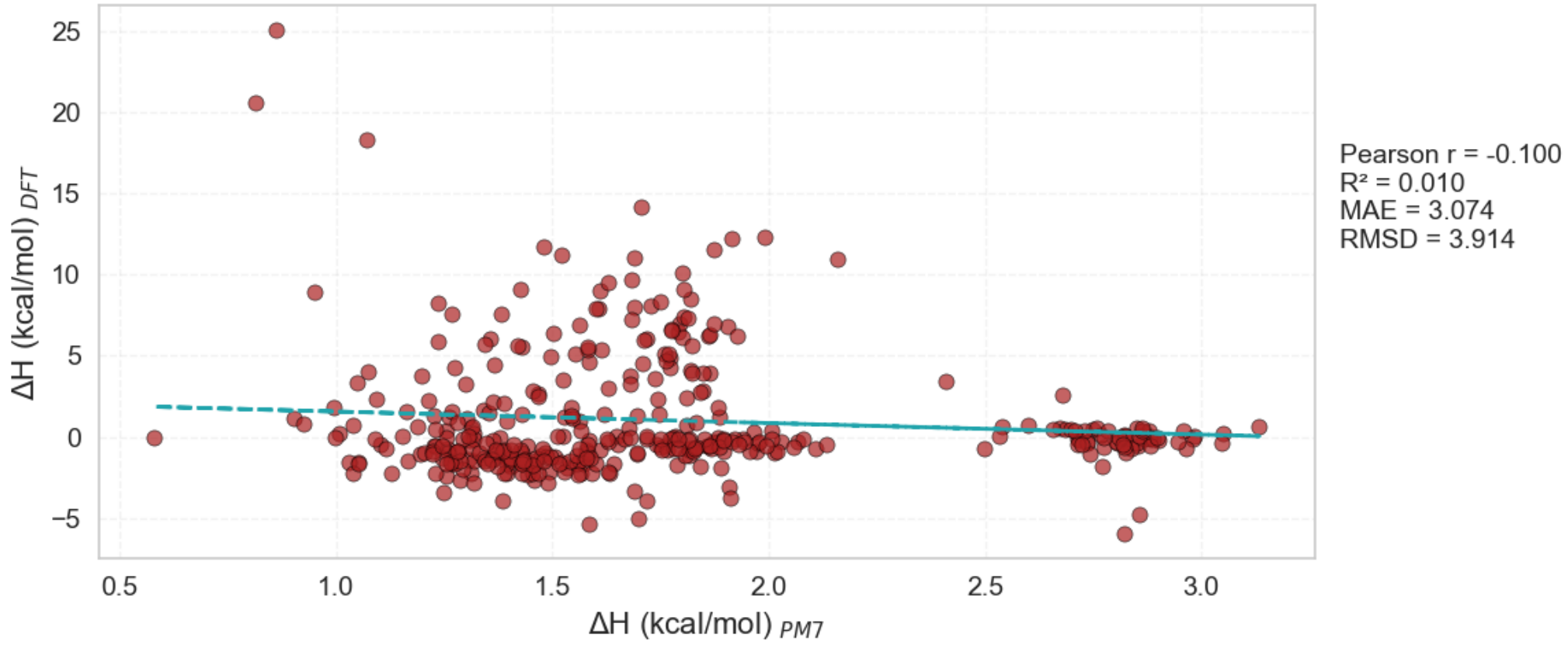

Figure S118: Correlation plot of the enthalpy (ΔH) between values computed with the PM7 method and those obtained at the DFT CAM-B3LYP/6-311++G(d,p) level of theory.

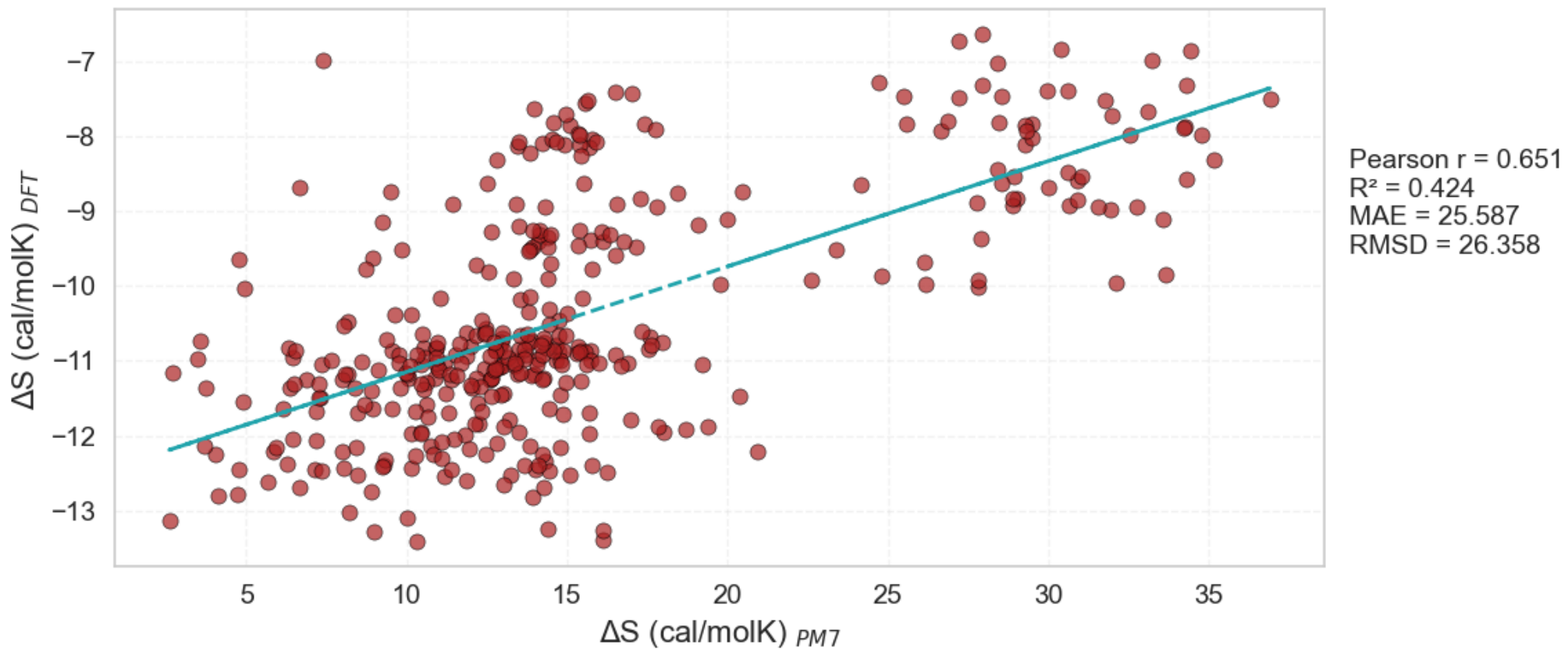

Figure S119: Correlation plot of the the entropy (ΔS) between values computed with the PM7 method and those obtained at the DFT CAM-B3LYP/6-311++G(d,p) level of theory.

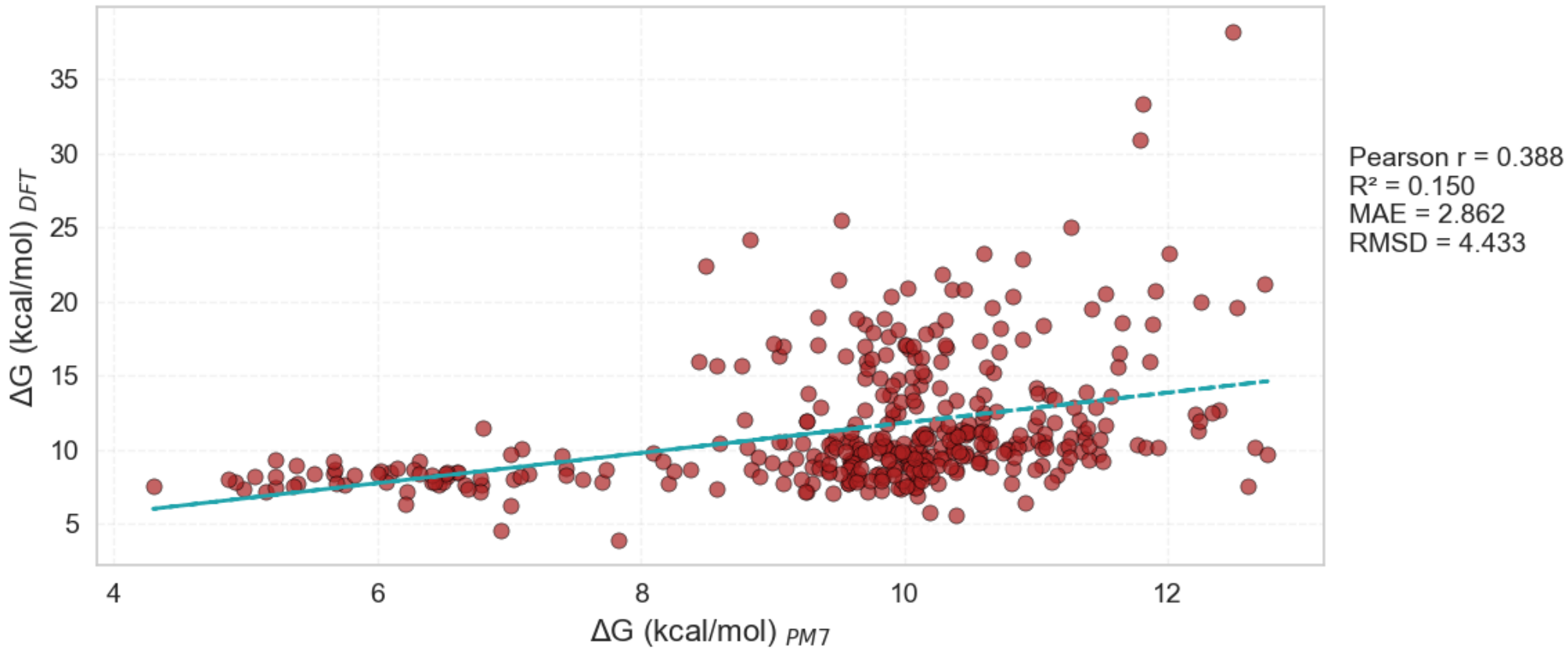

Figure S120: Correlation plot of the the Gibbs free energy (ΔG) between values computed with the PM7 method and those obtained at the DFT CAM-B3LYP/6-311++G(d,p) level of theory.

S4. 10 Correlations between the RM1 method and the CAM-B3LYP/6-311++G(d,p) level of theory

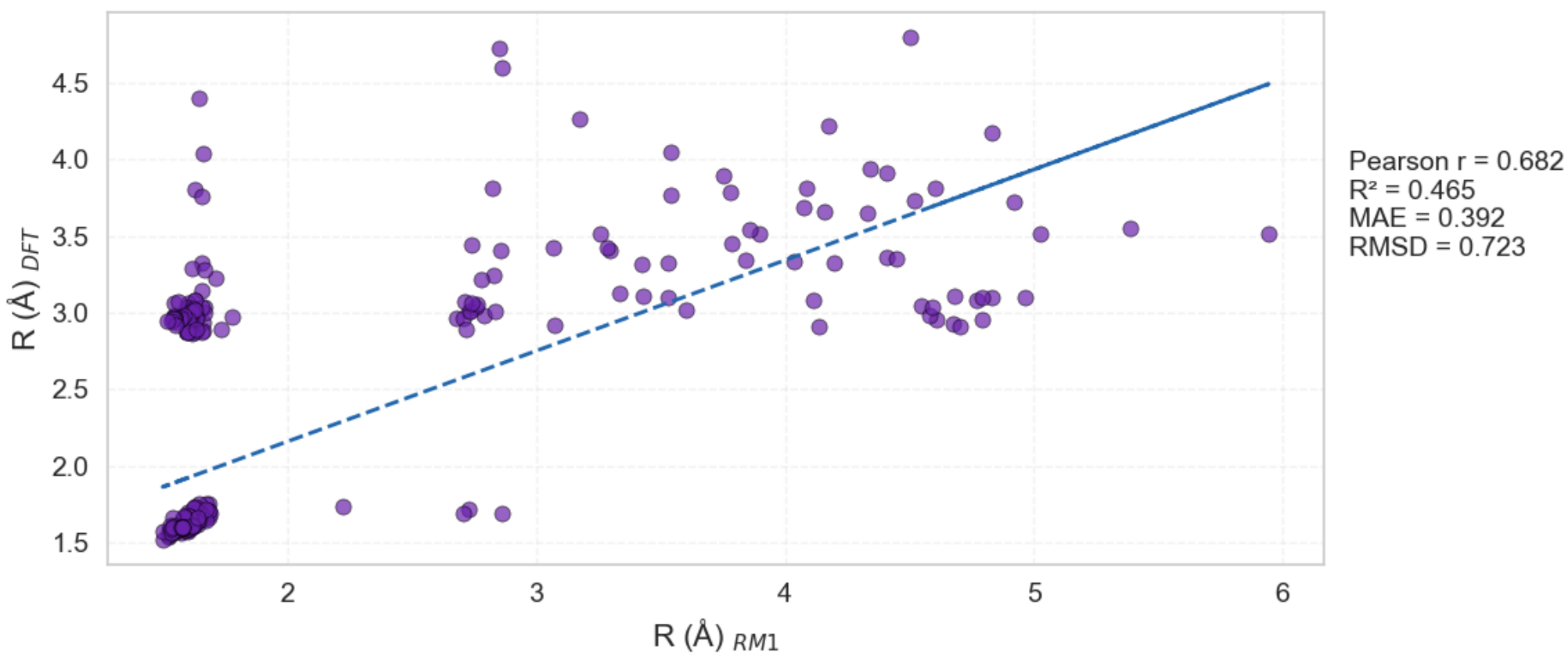

Figure S121: Correlation plot of the bond length R (Å) between values computed with the RM1 method and those obtained at the DFT CAM-B3LYP/6-311++G(d,p) level of theory.

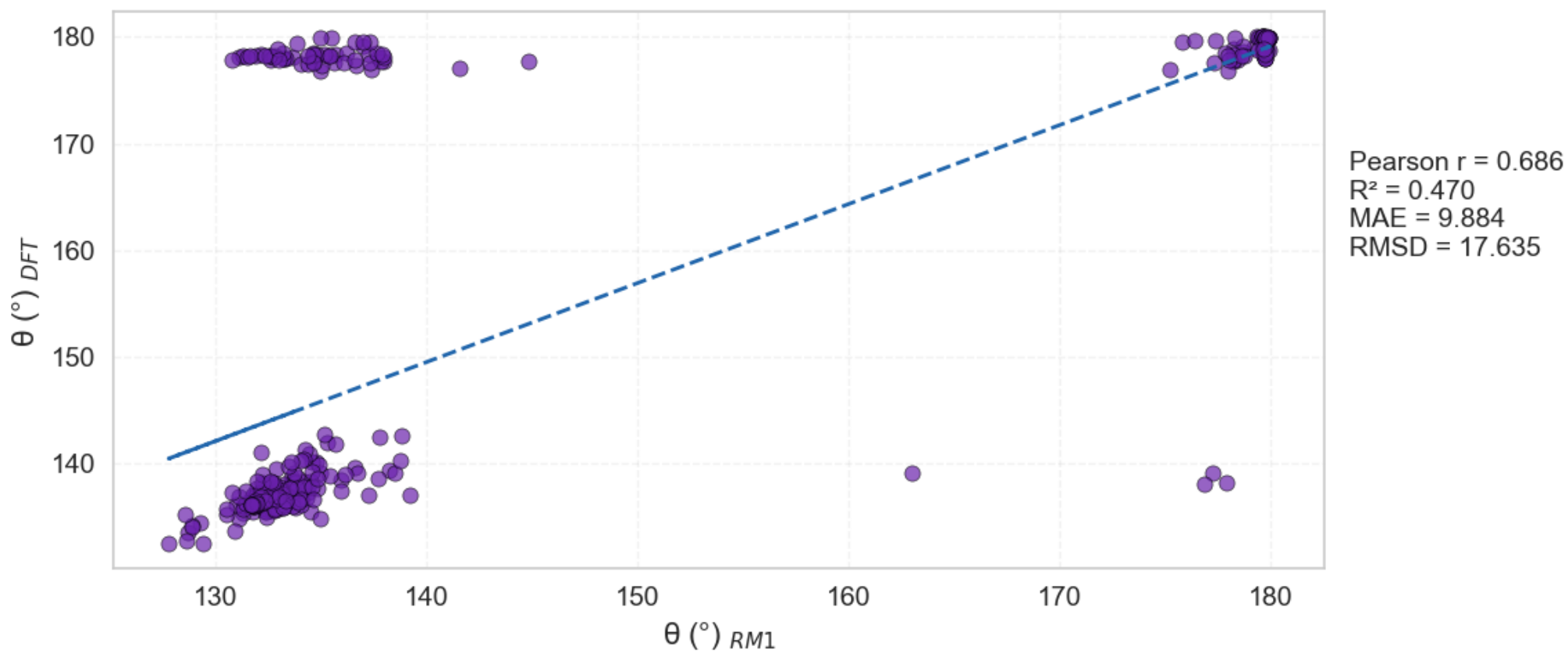

Figure S122: Correlation plot of the bond angle (θ) of the O=C=O between values computed with the RM1 method and those obtained at the DFT CAM-B3LYP/6-311++G(d,p) level of theory.

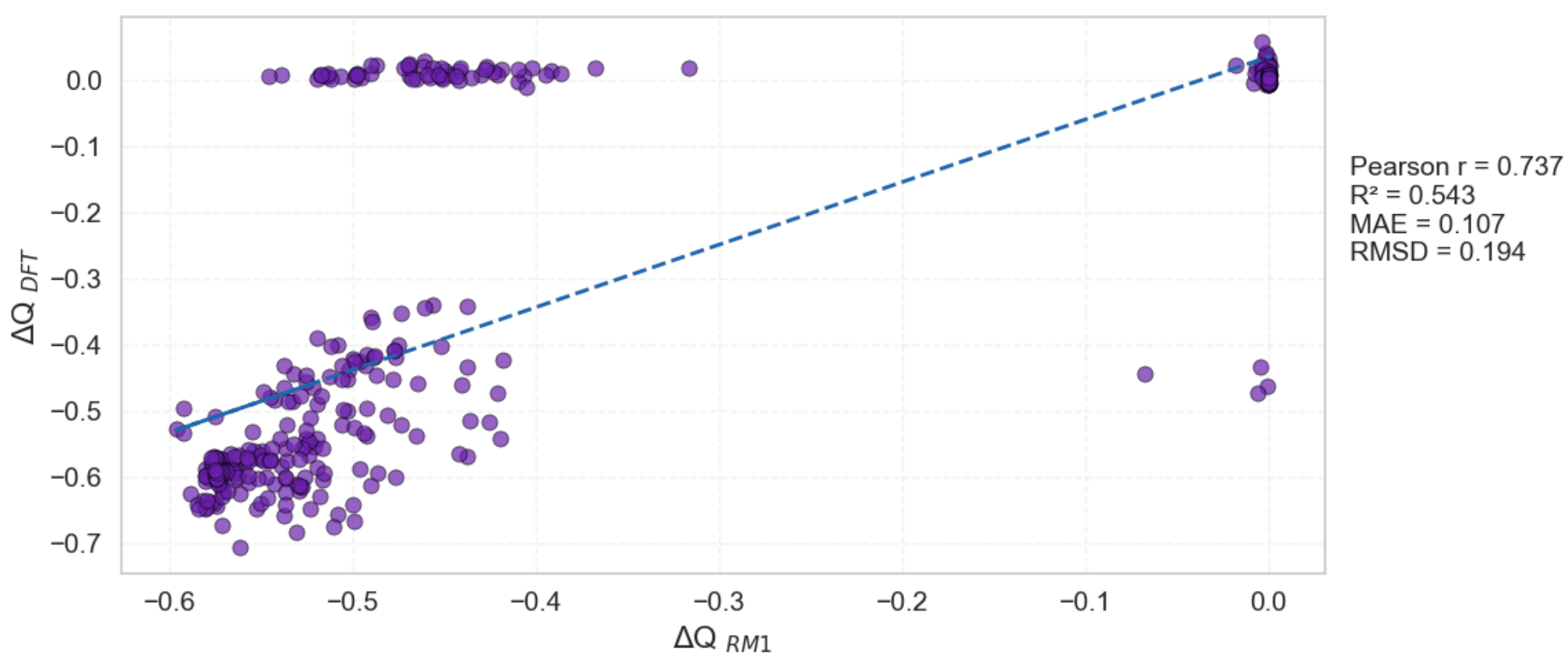

Figure S123: Correlation plot of the charge transfer (ΔQ) between values computed with the RM1 method and those obtained at the DFT CAM-B3LYP/6-311++G(d,p) level of theory.

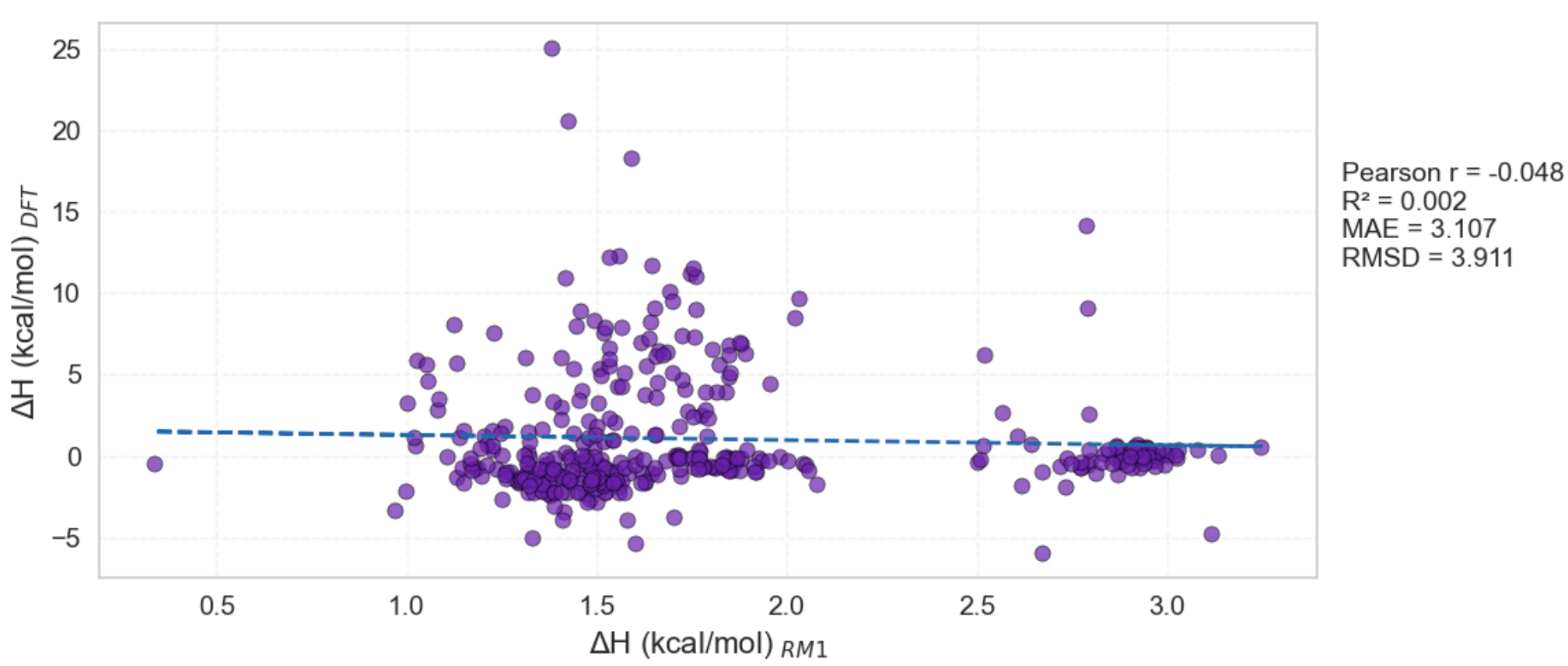

Figure S124: Correlation plot of the enthalpy (ΔH) between values computed with the RM1 method and those obtained at the DFT CAM-B3LYP/6-311++G(d,p) level of theory.

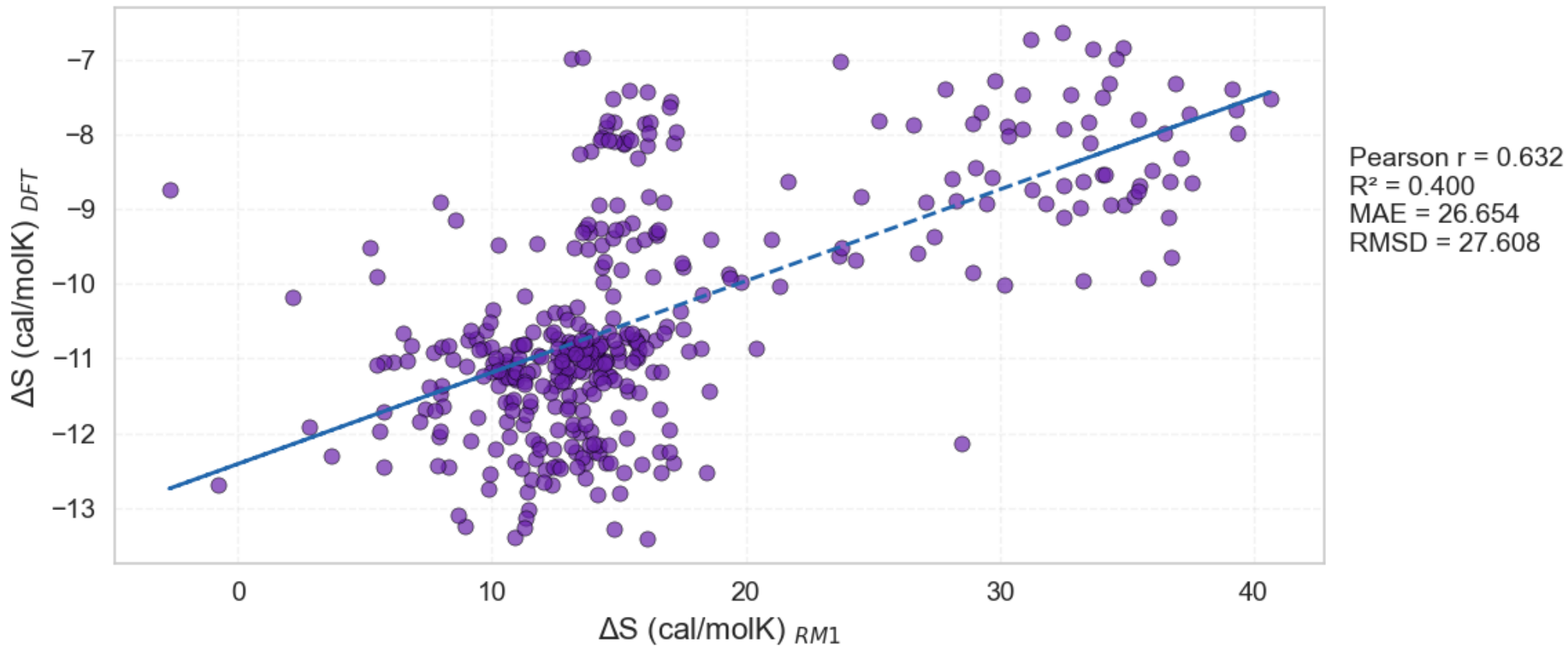

Figure S125: Correlation plot of the the entropy (ΔS) between values computed with the RM1 method and those obtained at the DFT CAM-B3LYP/6-311++G(d,p) level of theory.

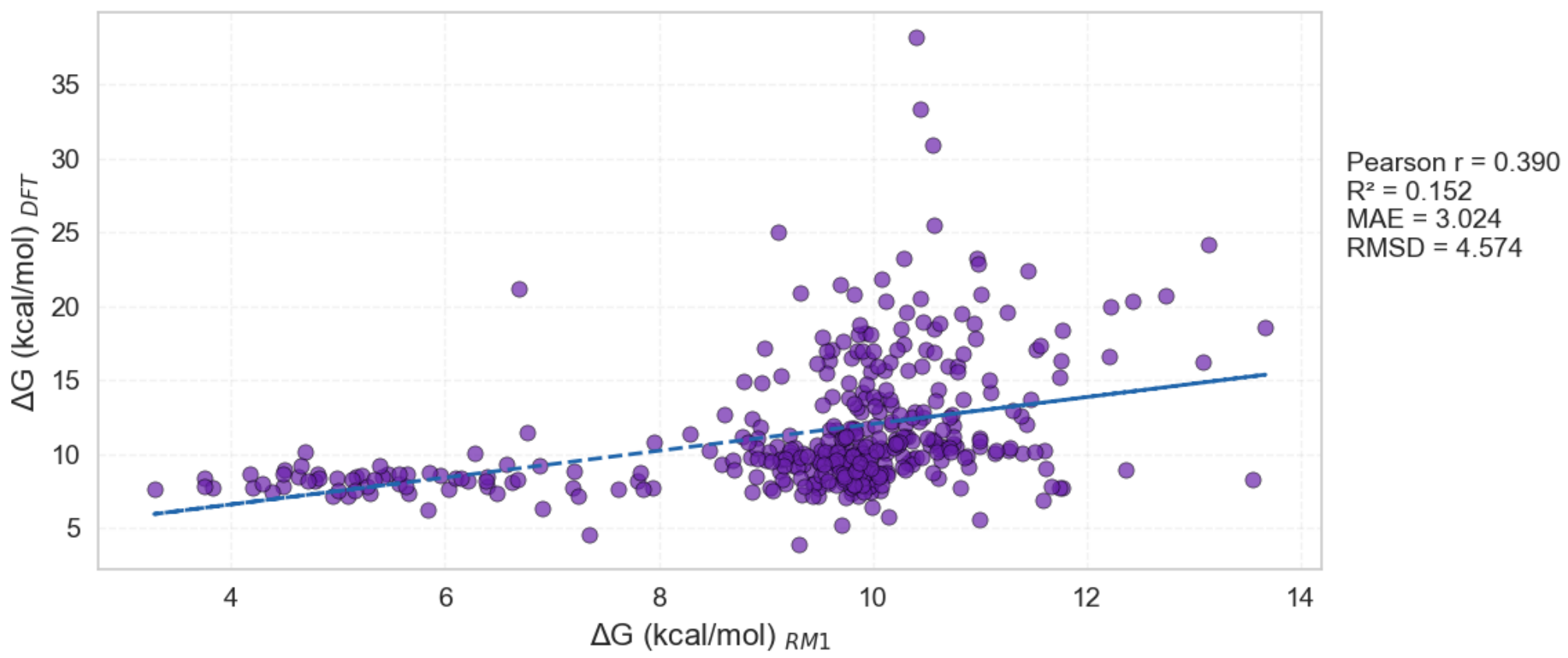

Figure S126: Correlation plot of the the Gibbs free energy (ΔG) between values computed with the RM1 method and those obtained at the DFT CAM-B3LYP/6-311++G(d,p) level of theory.

S5. Residual distributions and parity plots in Ensemble ML Regressor Methods.

S5.1 Randon Forest Regressor

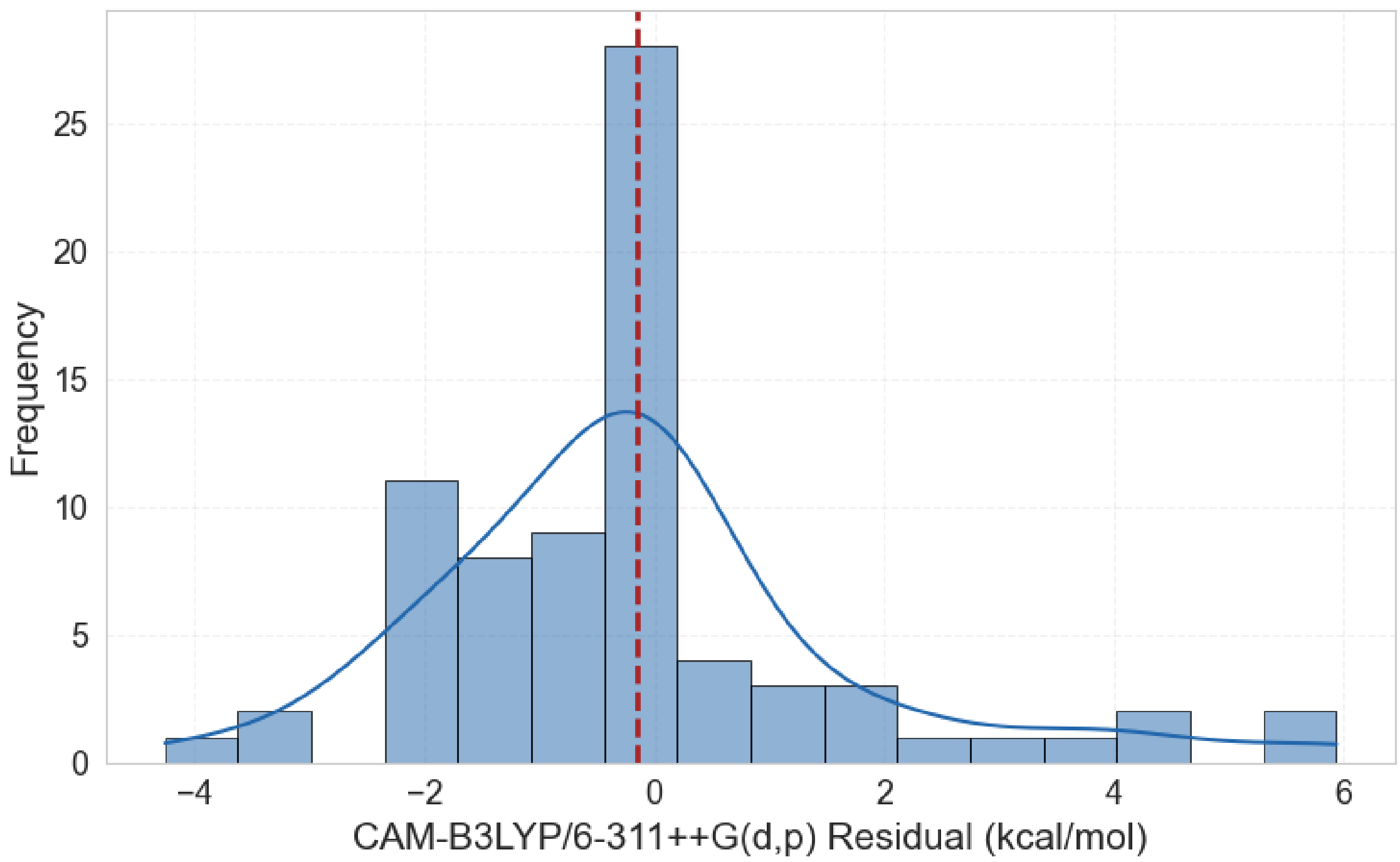

Figure S127: Residual distribution of the predicted enthalpy values obtained using the Random Forest model with features derived from the CAM-B3LYP/6-311++G(d,p) method, in comparison with the reference values.

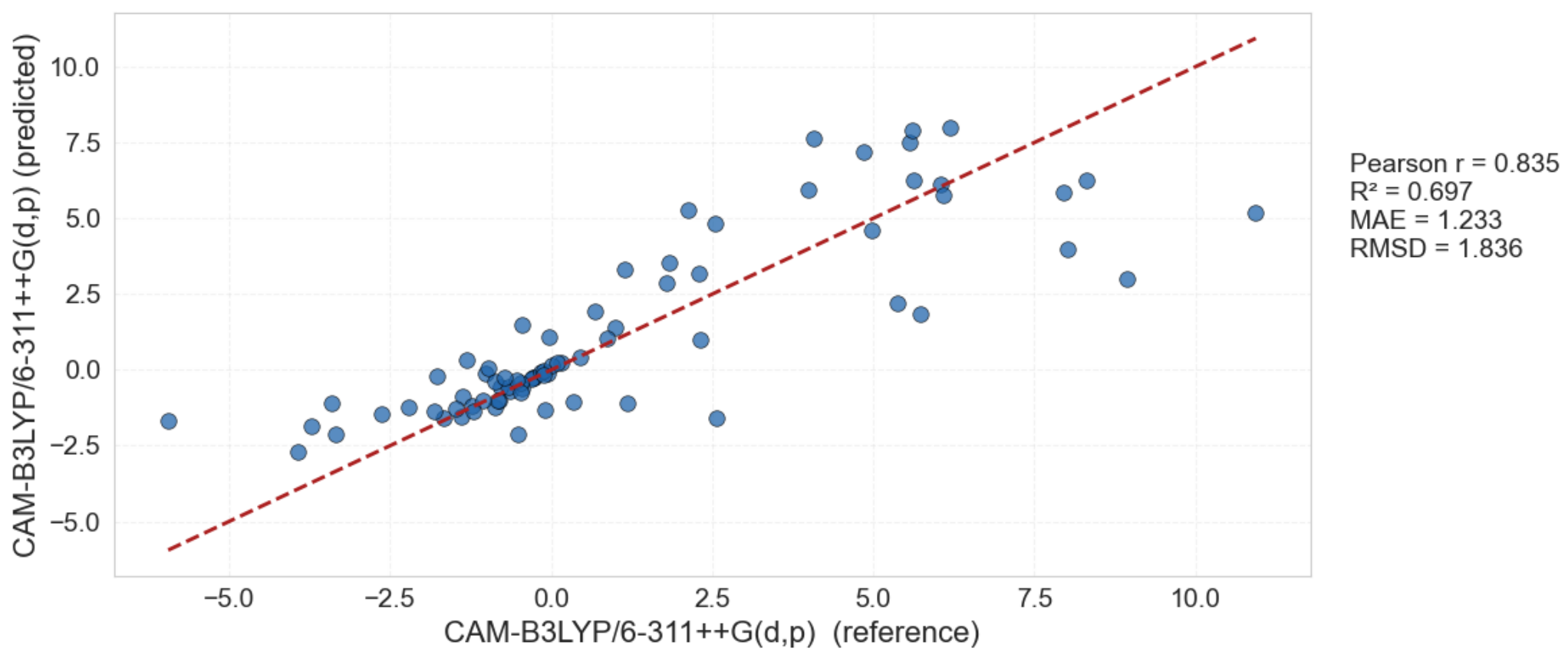

Figure S128: Parity plots of enthalpy values predicted using the Random Forest model with features derived from the CAM-B3LYP/6-311++G(d,p) method and the corresponding reference values.

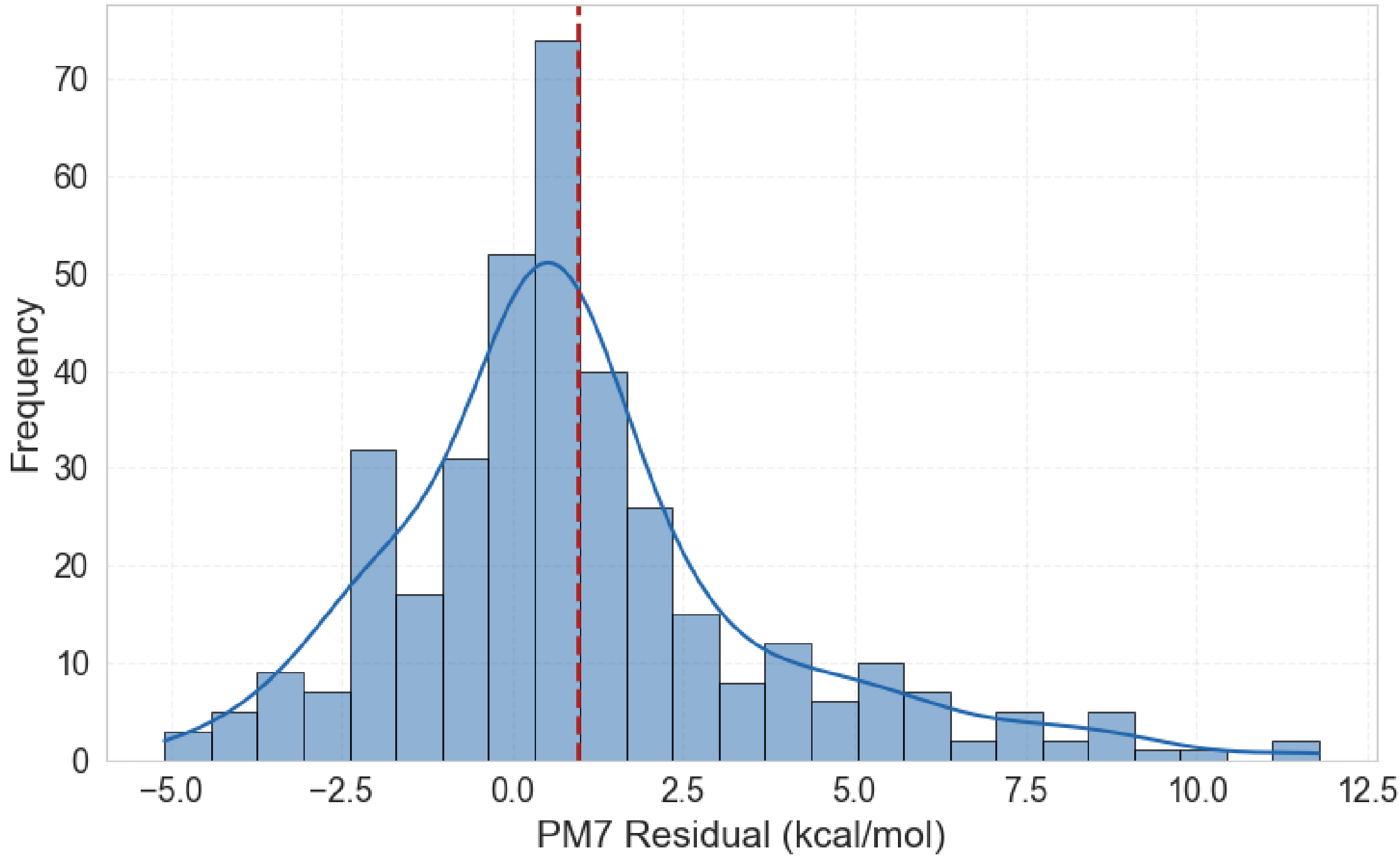

Figure S129: Residual distribution of the predicted enthalpy values obtained using the Random Forest model with features derived from the PM7 method, in comparison with the reference values.

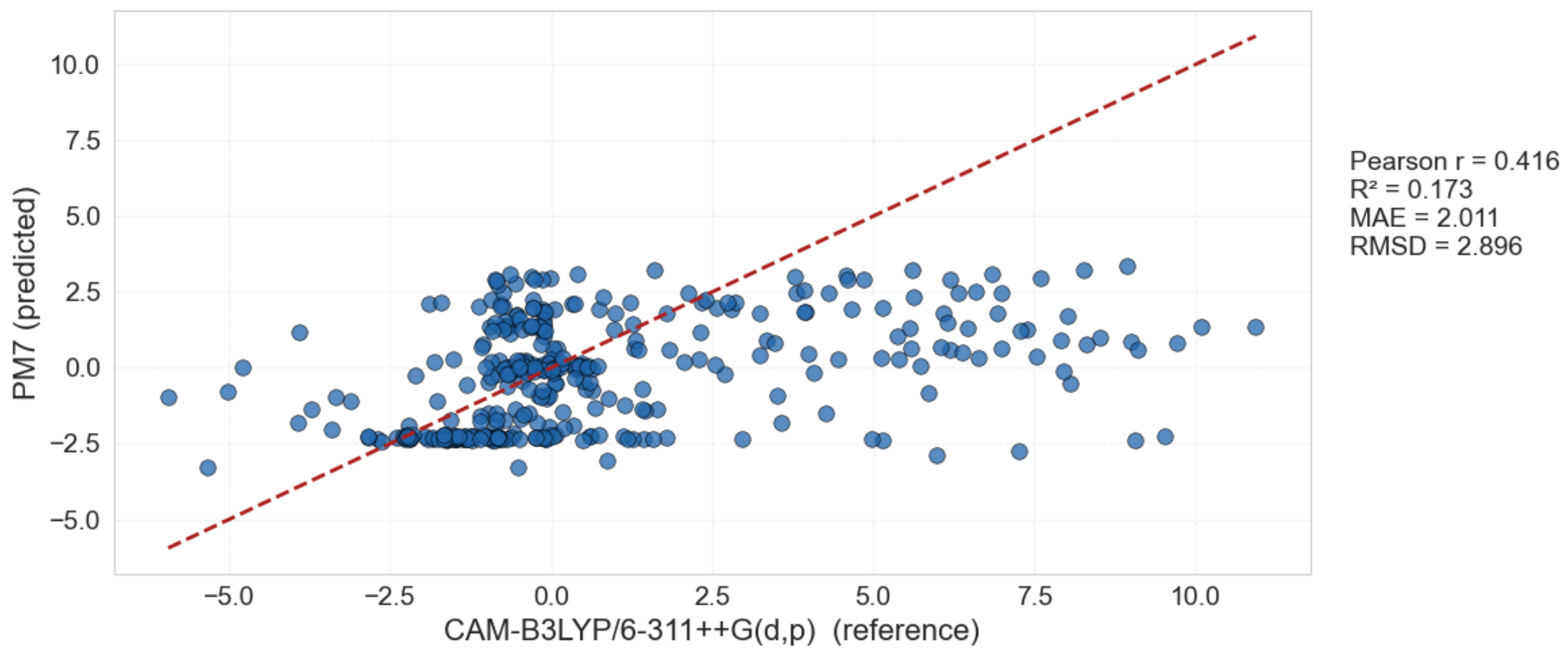

Figure S130: Parity plots of enthalpy values predicted using the Random Forest model with features derived from the PM7 method and the corresponding reference values.

S5.2 Extra Trees Regressor

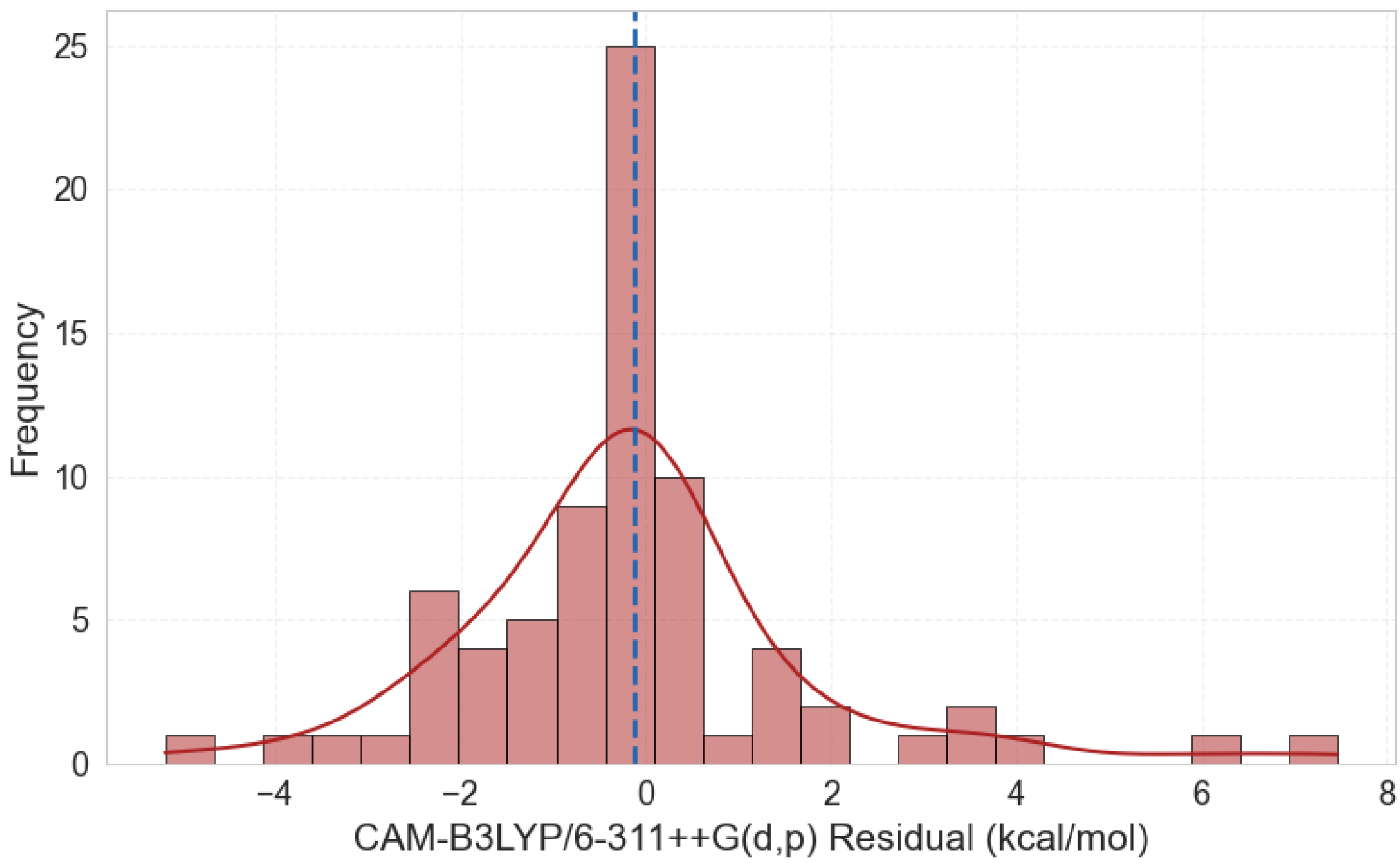

Figure S131: Residual distribution of the predicted enthalpy values obtained using the Extra Trees Regressor model with features derived from the CAM-B3LYP/6-311++G(d,p) method, in comparison with the reference values.

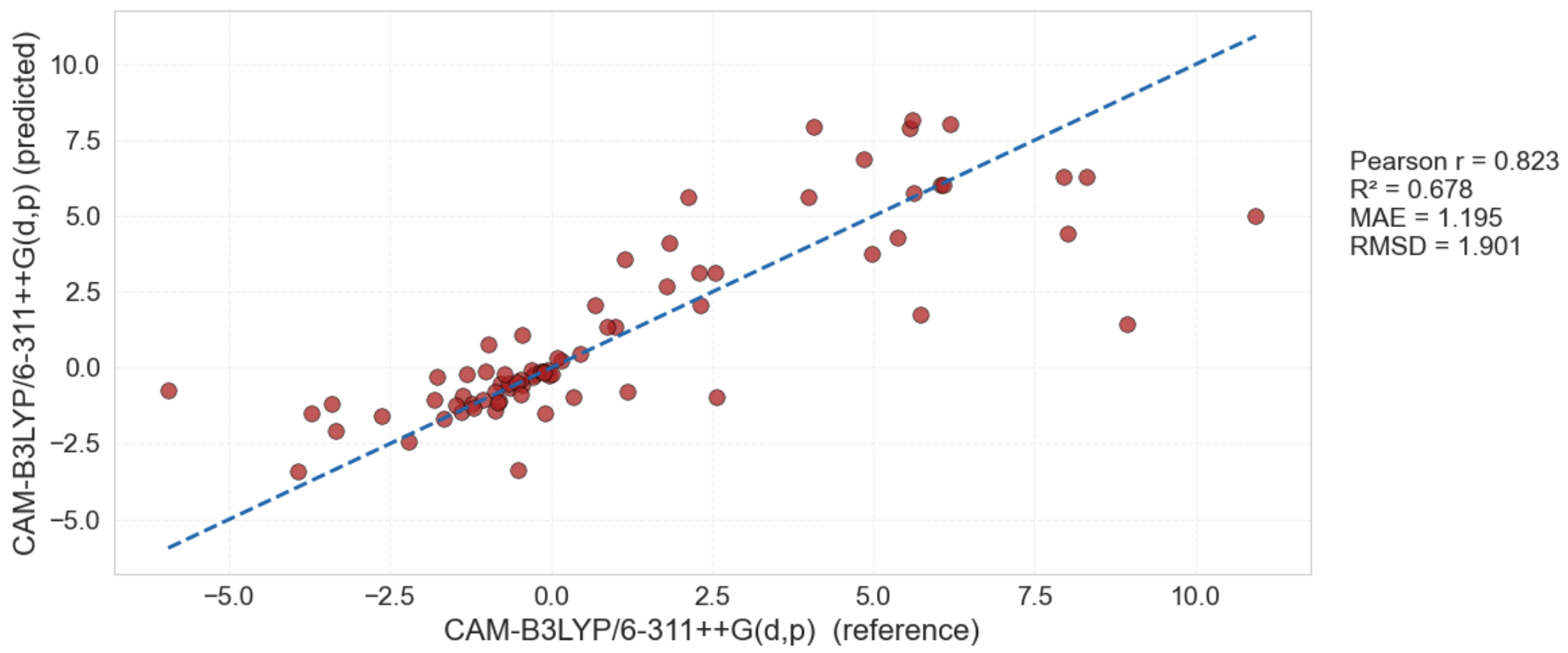

Figure S132: Parity plots of enthalpy values predicted using the Extra Trees Regressor model with features derived from the CAM-B3LYP/6-311++G(d,p) method and the corresponding reference values.

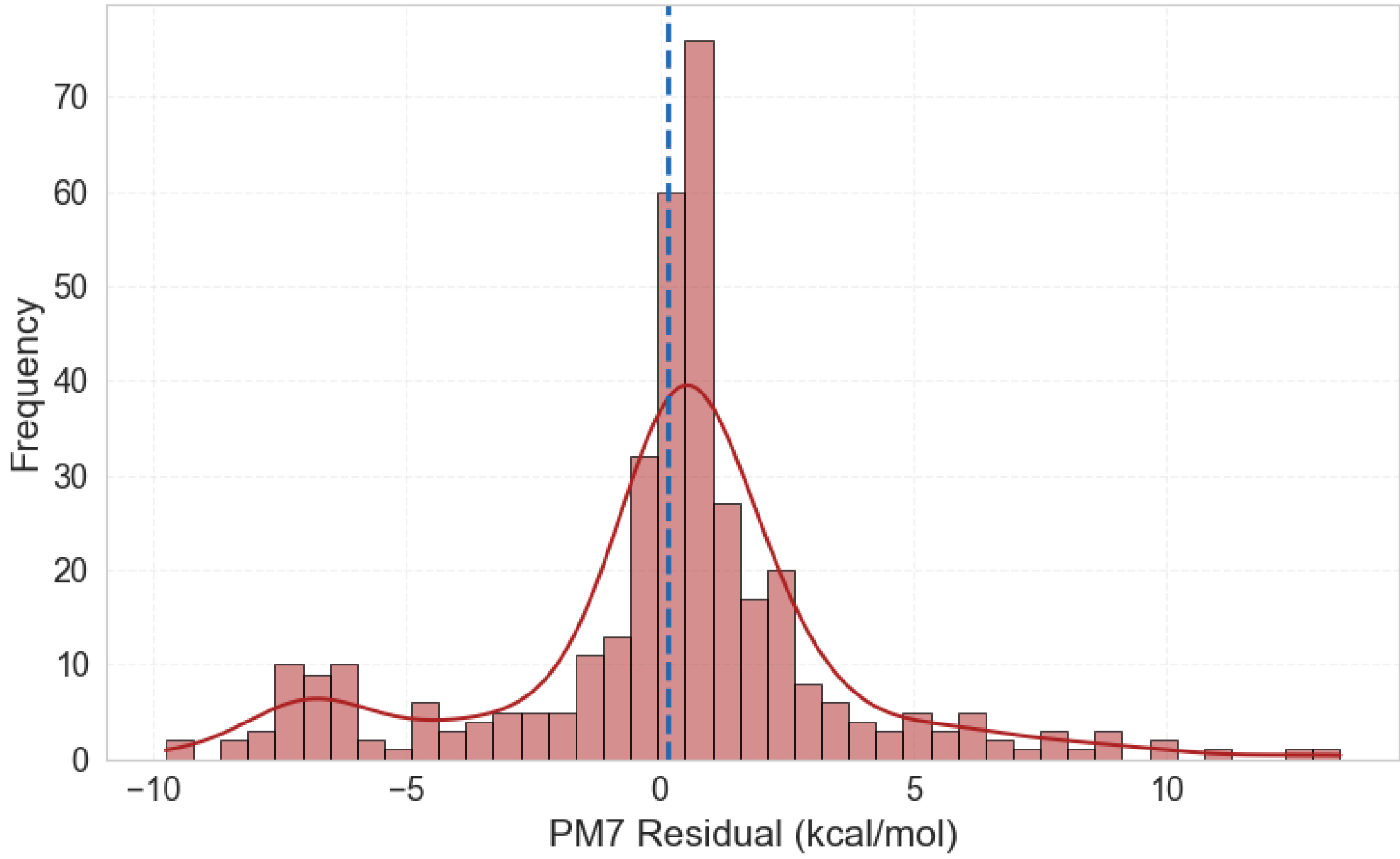

Figure S133: Residual distribution of the predicted enthalpy values obtained using the Extra Trees Regressor model with features derived from the PM7 method, in comparison with the reference values.

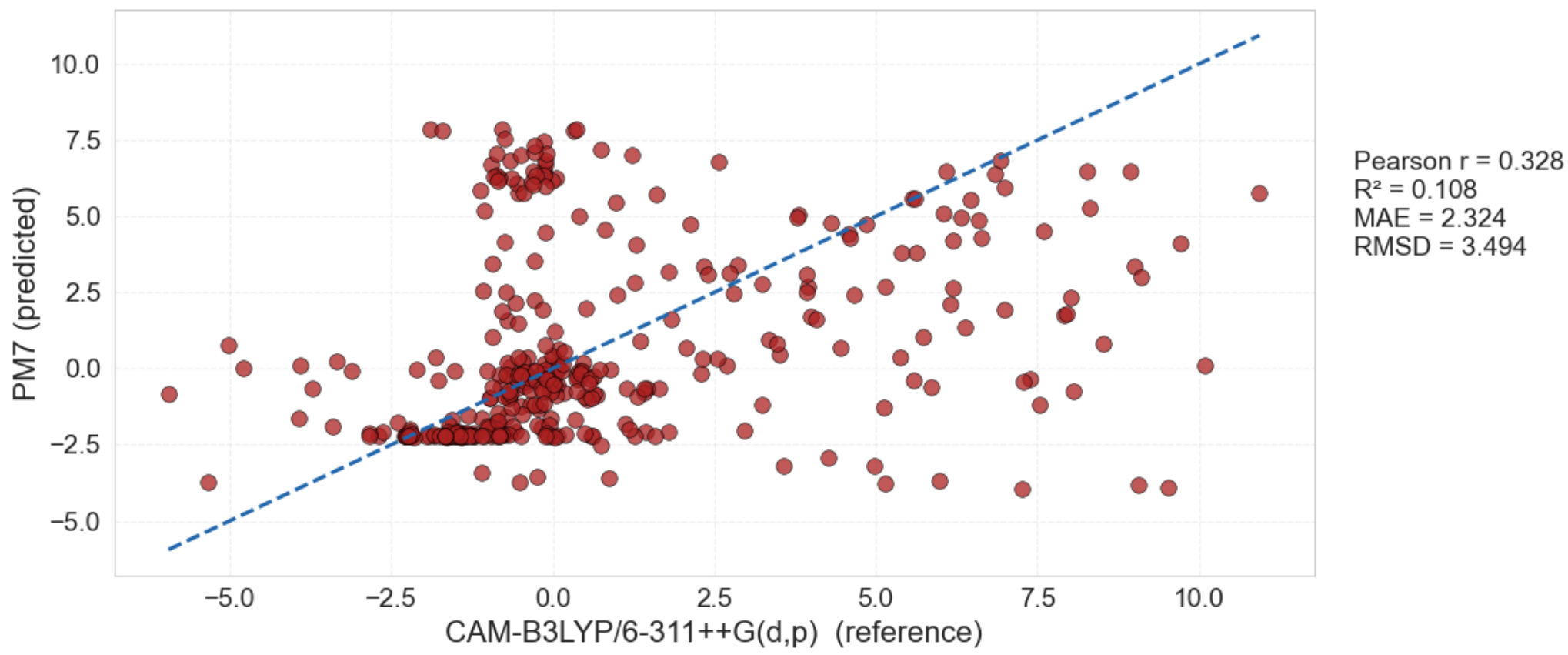

Figure S134: Parity plots of enthalpy values predicted using the Extra Trees Regressor model with features derived from the PM7 method and the corresponding reference values.

S5.3 Gradient Boosting Regressor

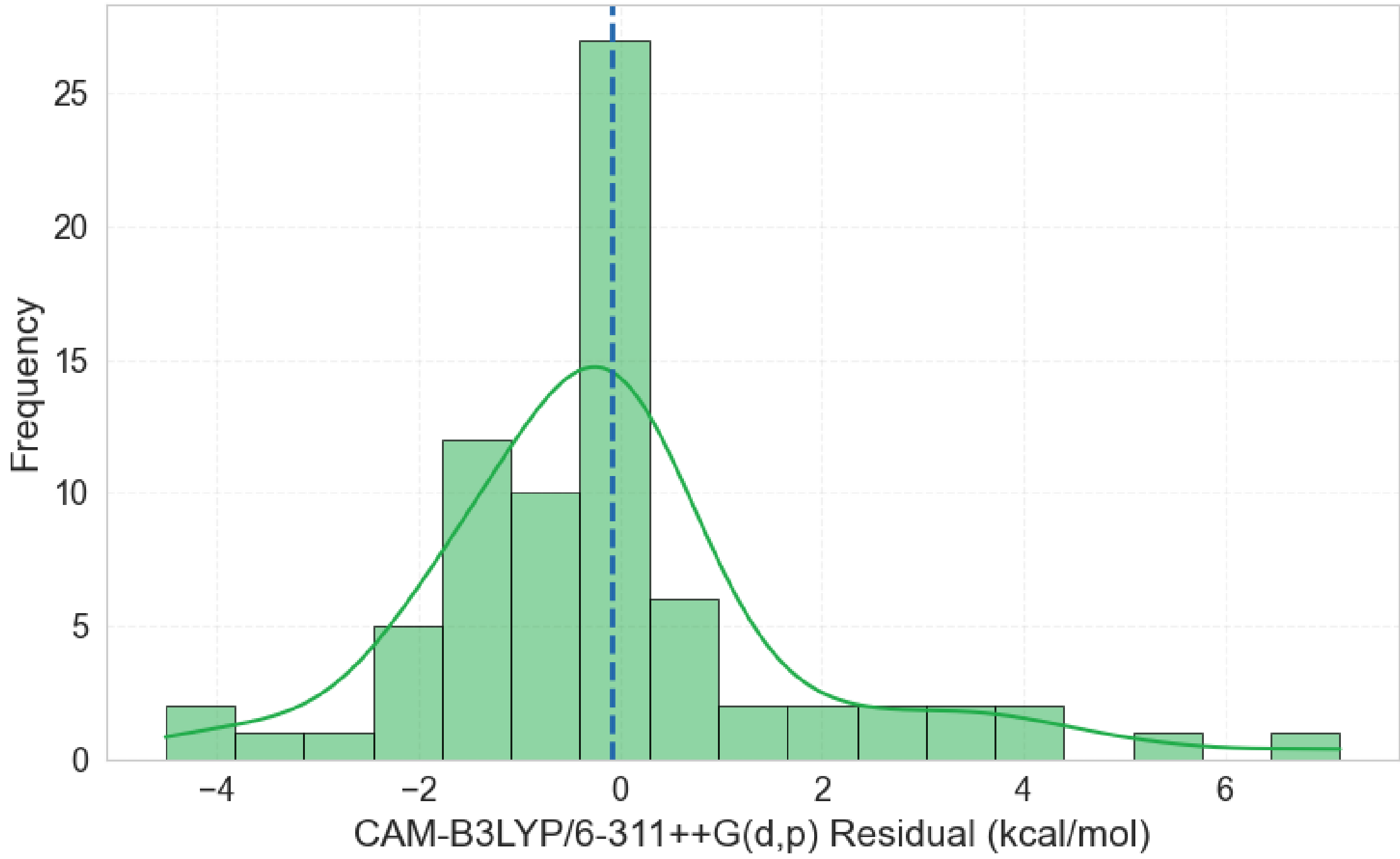

Figure S135: Residual distribution of the predicted enthalpy values obtained using the Gradient Boosting Regressor model with features derived from the CAM-B3LYP/6-311++G(d,p) method, in comparison with the reference values.

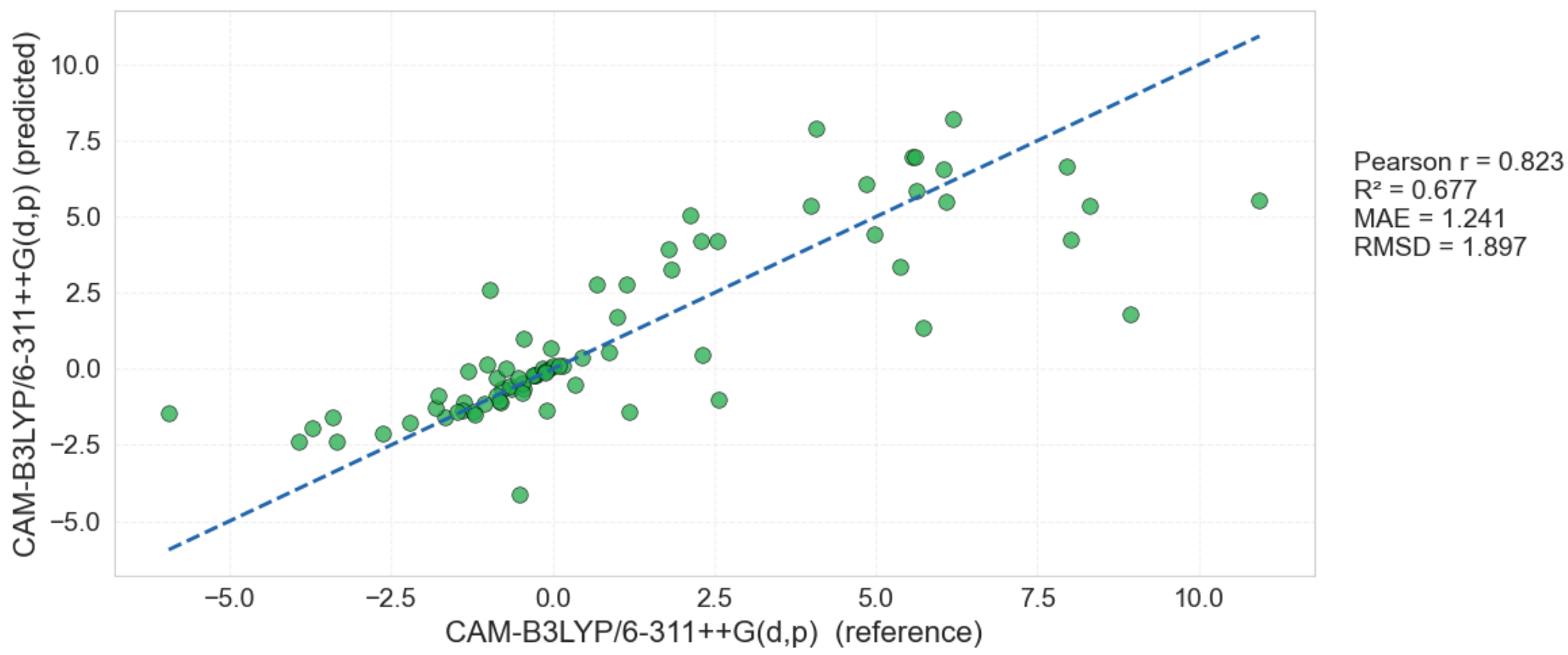

Figure S136: Parity plots of enthalpy values predicted using the Gradient Boosting Regressor model with features derived from the CAM-B3LYP/6-311++G(d,p) method and the corresponding reference values.

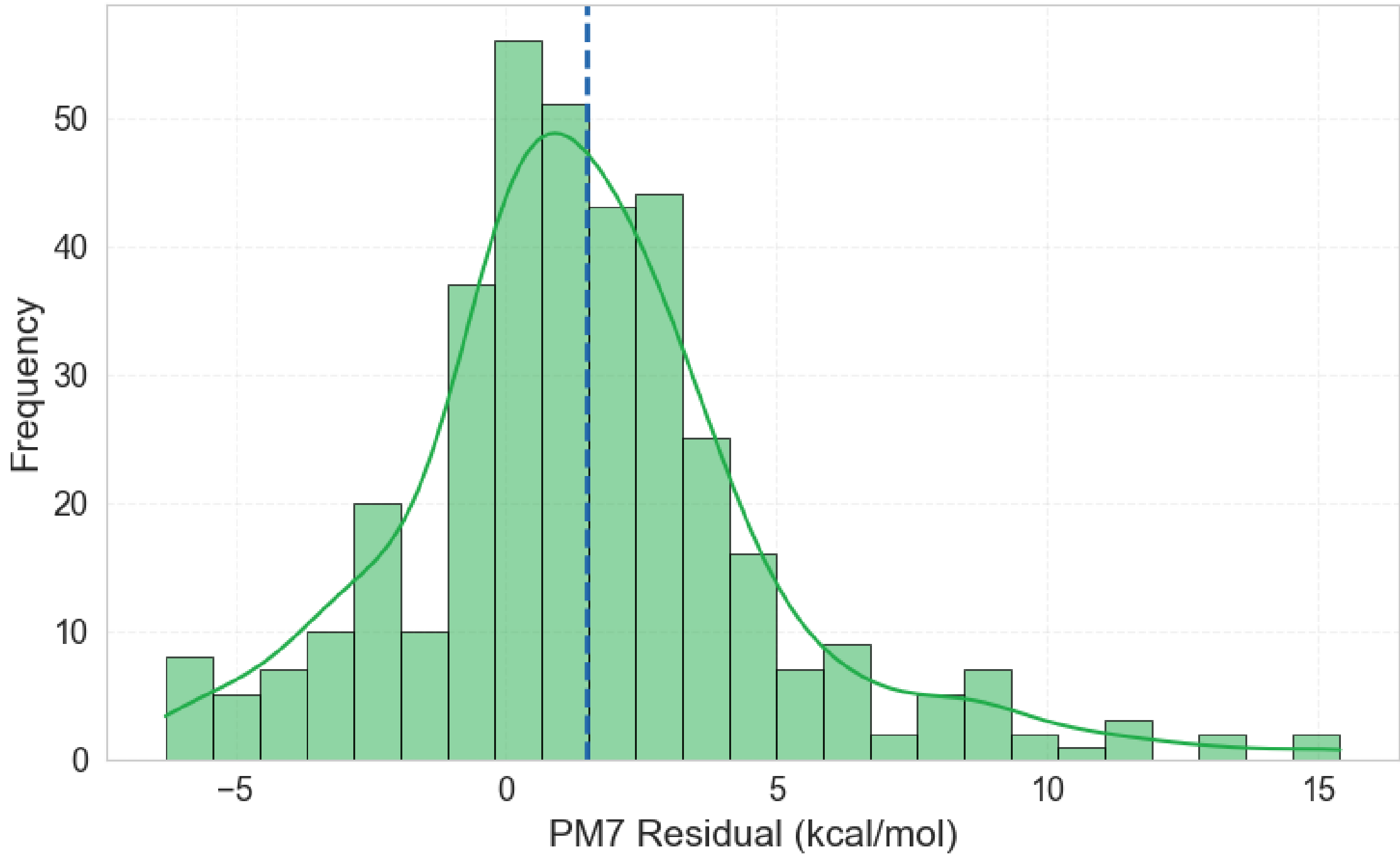

Figure S137: Residual distribution of the predicted enthalpy values obtained using the Random Forest model with features derived from the PM7 method, in comparison with the reference values.

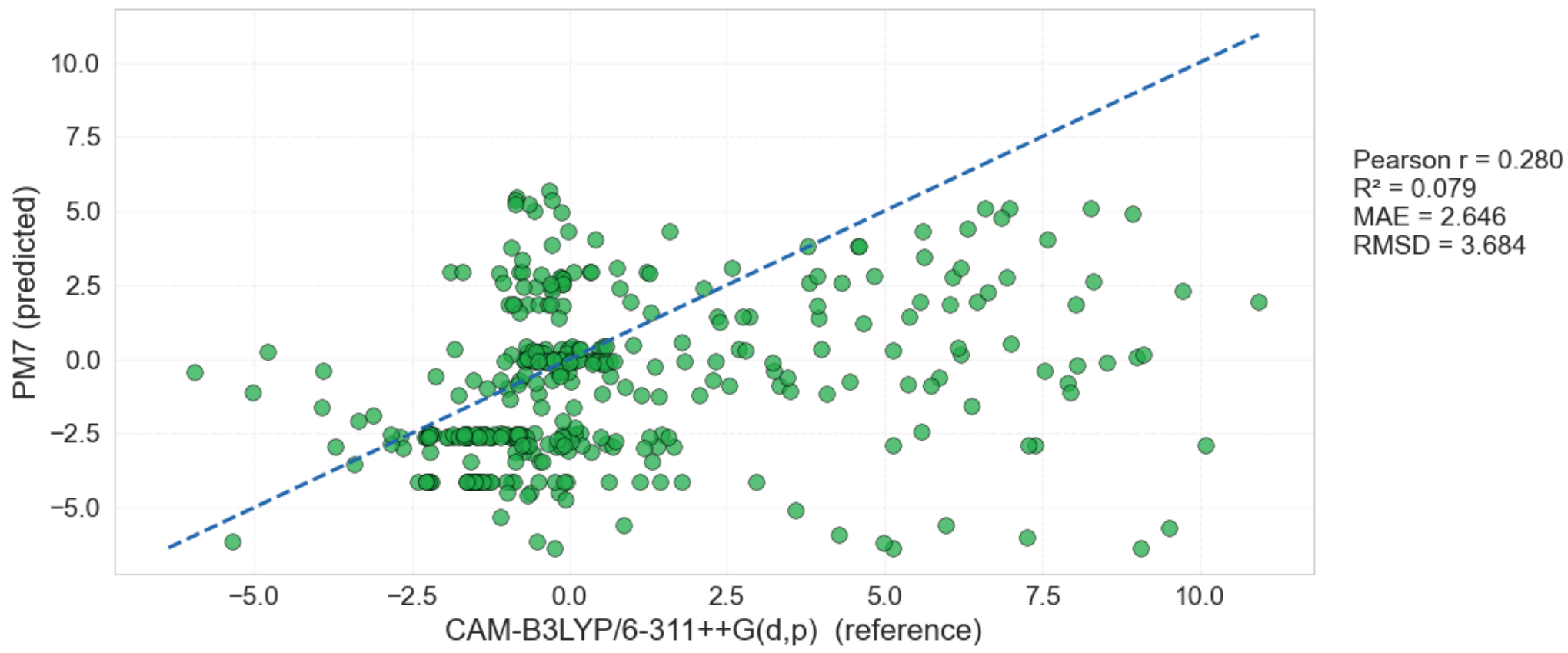

Figure S138: Parity plots of enthalpy values predicted using the Gradient Boosting Regressor model with features derived from the PM7 method and the corresponding reference values.

S5.4 Ada Boost Regressor

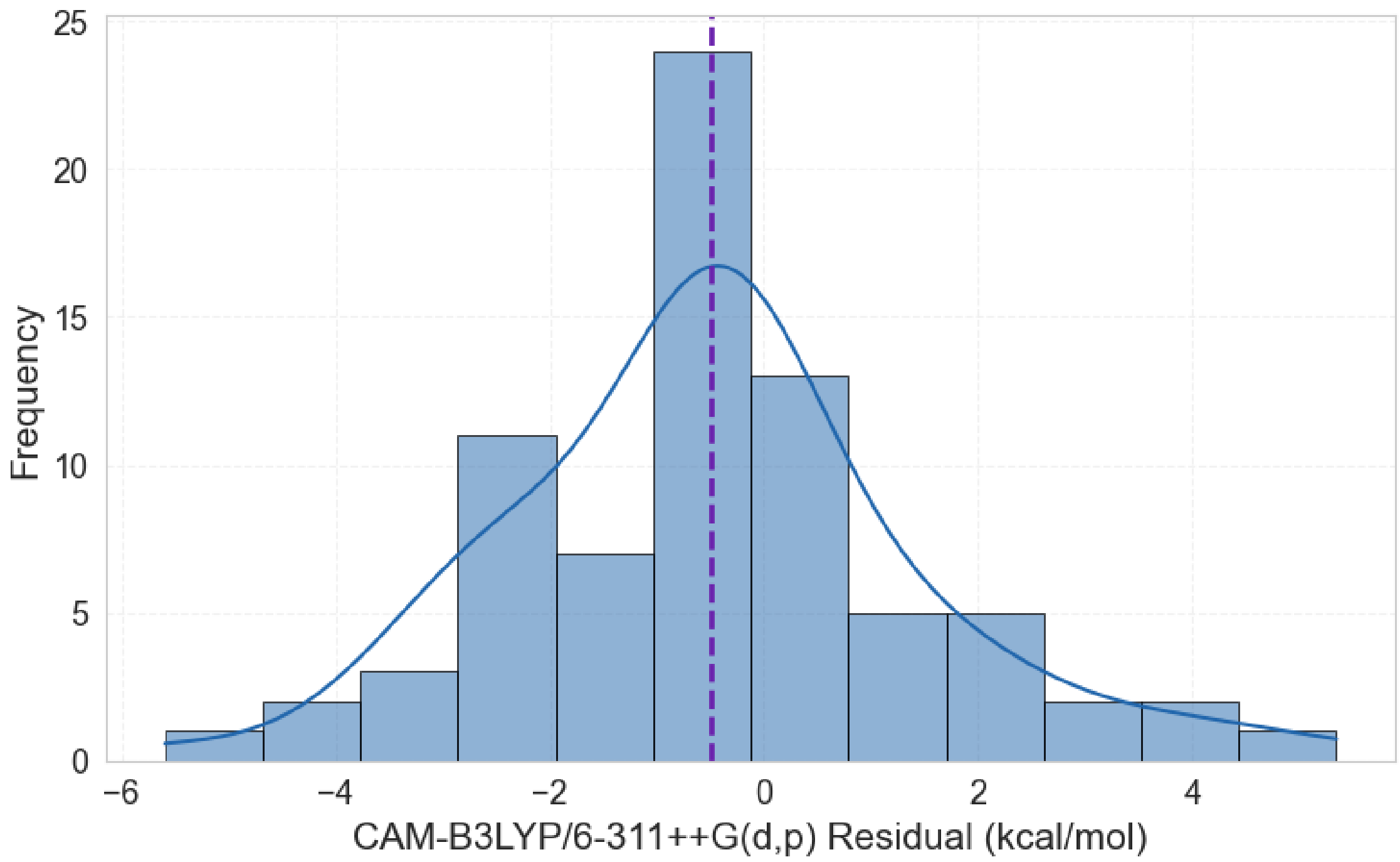

Figure S139: Residual distribution of the predicted enthalpy values obtained using the Ada Boost Regressor model with features derived from the CAM-B3LYP/6-311++G(d,p) method, in comparison with the reference values.

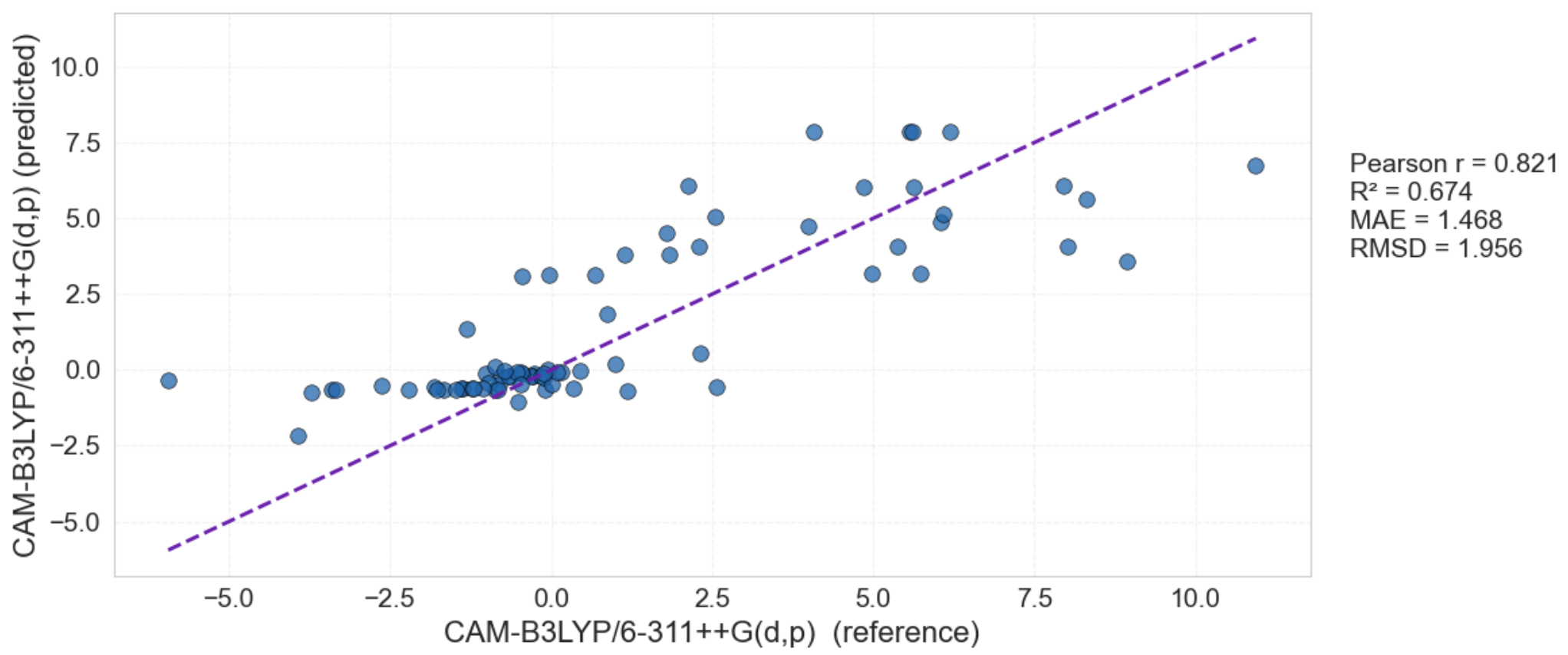

Figure S140: Parity plots of enthalpy values predicted using the Ada Boost Regressor model with features derived from the CAM-B3LYP/6-311++G(d,p) method and the corresponding reference values.

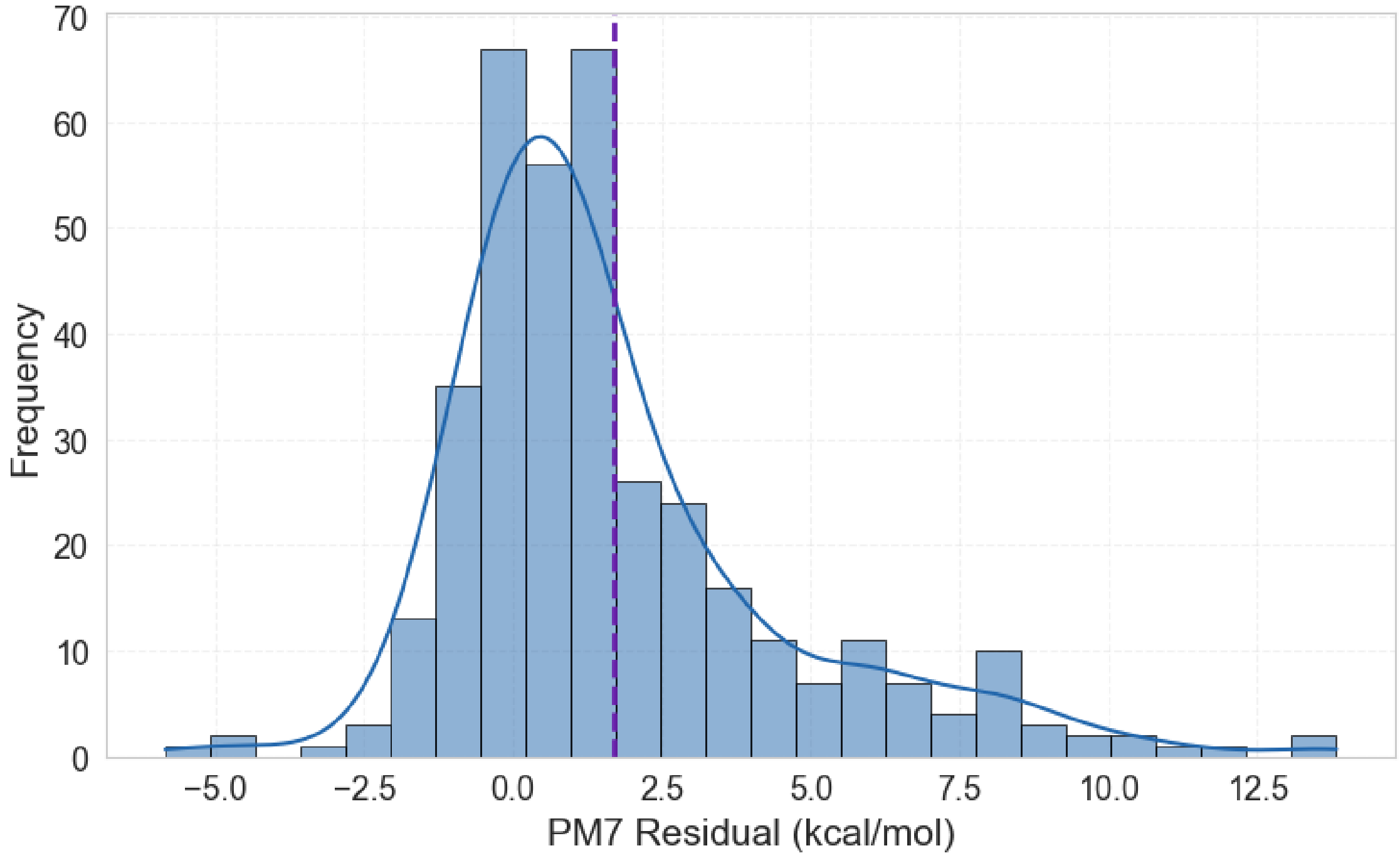

Figure S141: Residual distribution of the predicted enthalpy values obtained using the Ada Boost Regressor model with features derived from the PM7 method, in comparison with the reference values.

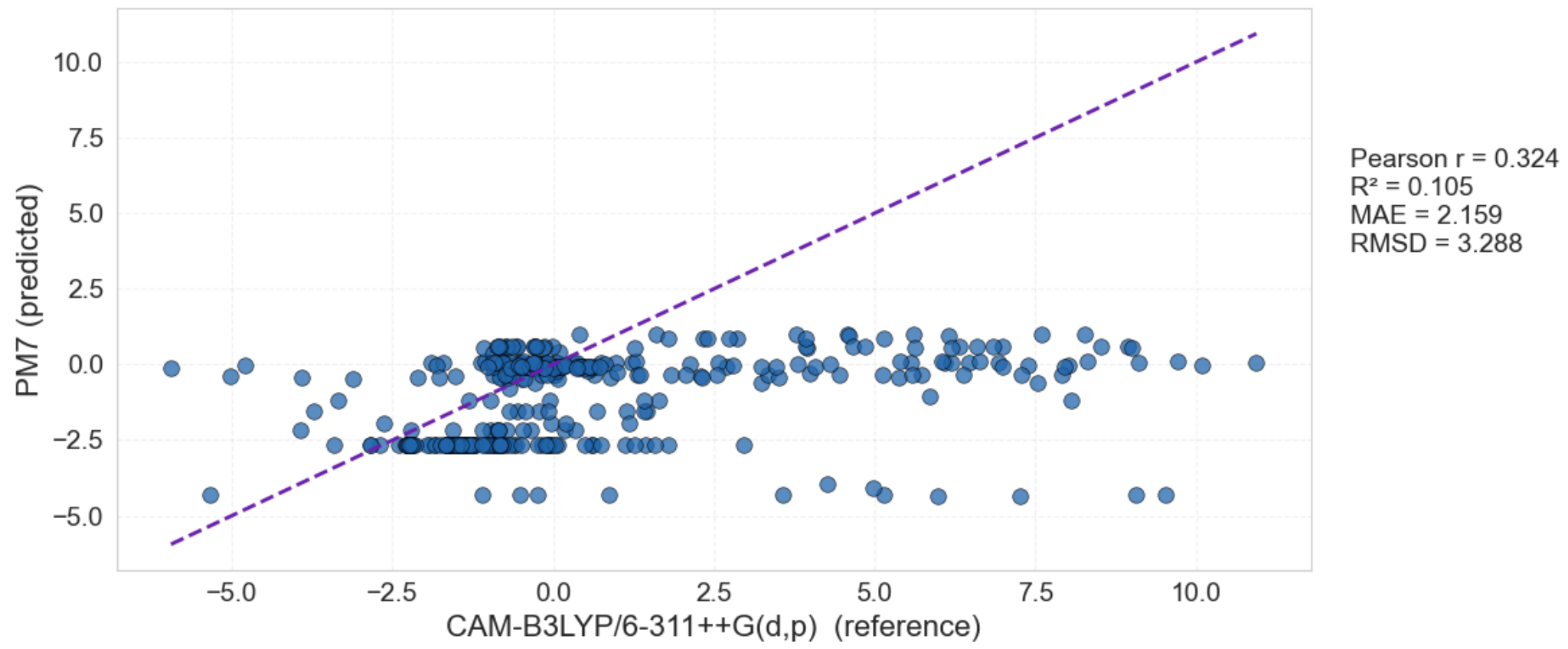

Figure S142: Parity plots of enthalpy values predicted using the Ada Boost Regressor model with features derived from the PM7 method and the corresponding reference values.

S5.5 Hist Gradient Boosting Regressor

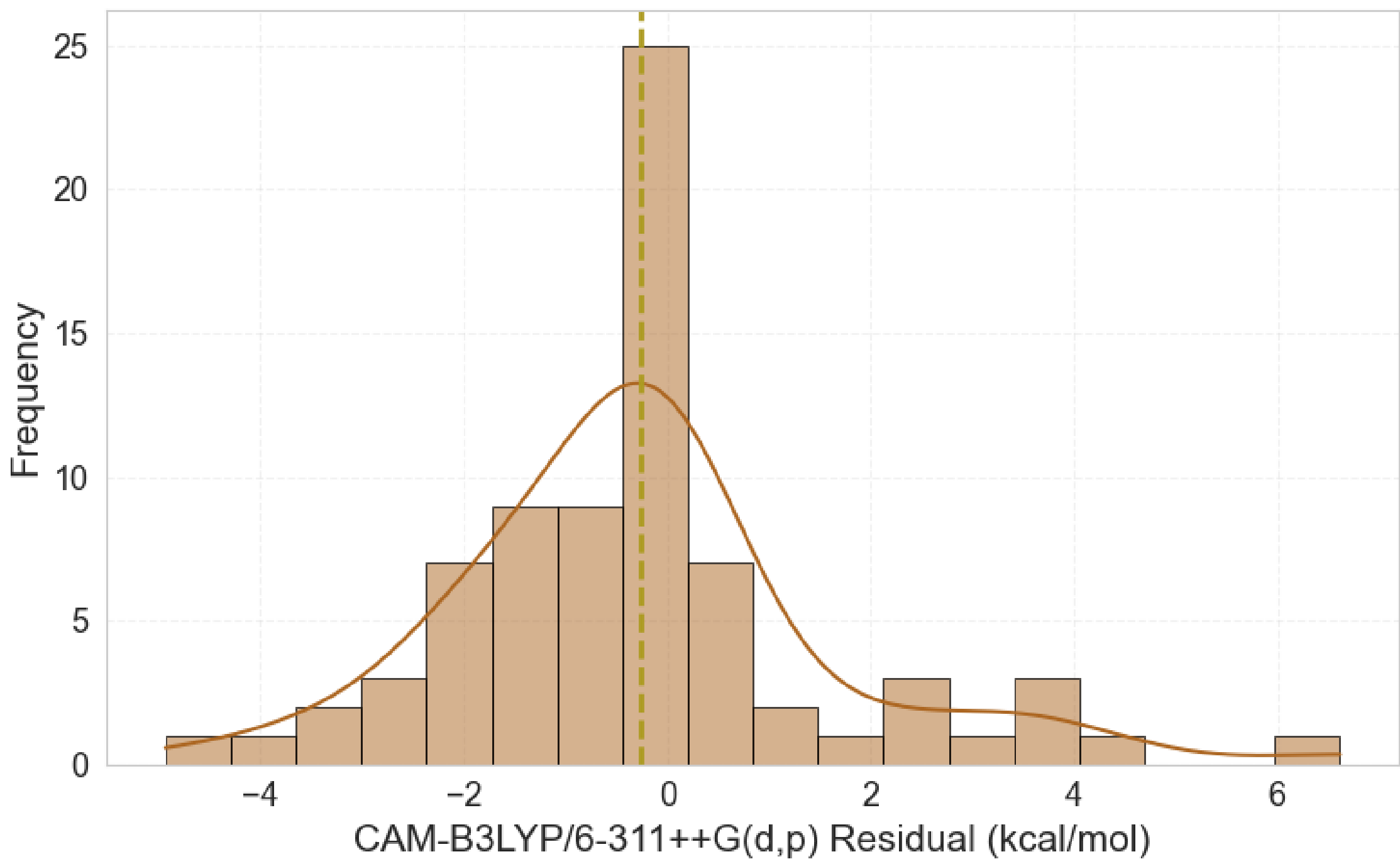

Figure S143: Residual distribution of the predicted enthalpy values obtained using the Hist Gradient Boosting Regressor model with features derived from the CAM-B3LYP/6-311++G(d,p) method, in comparison with the reference values.

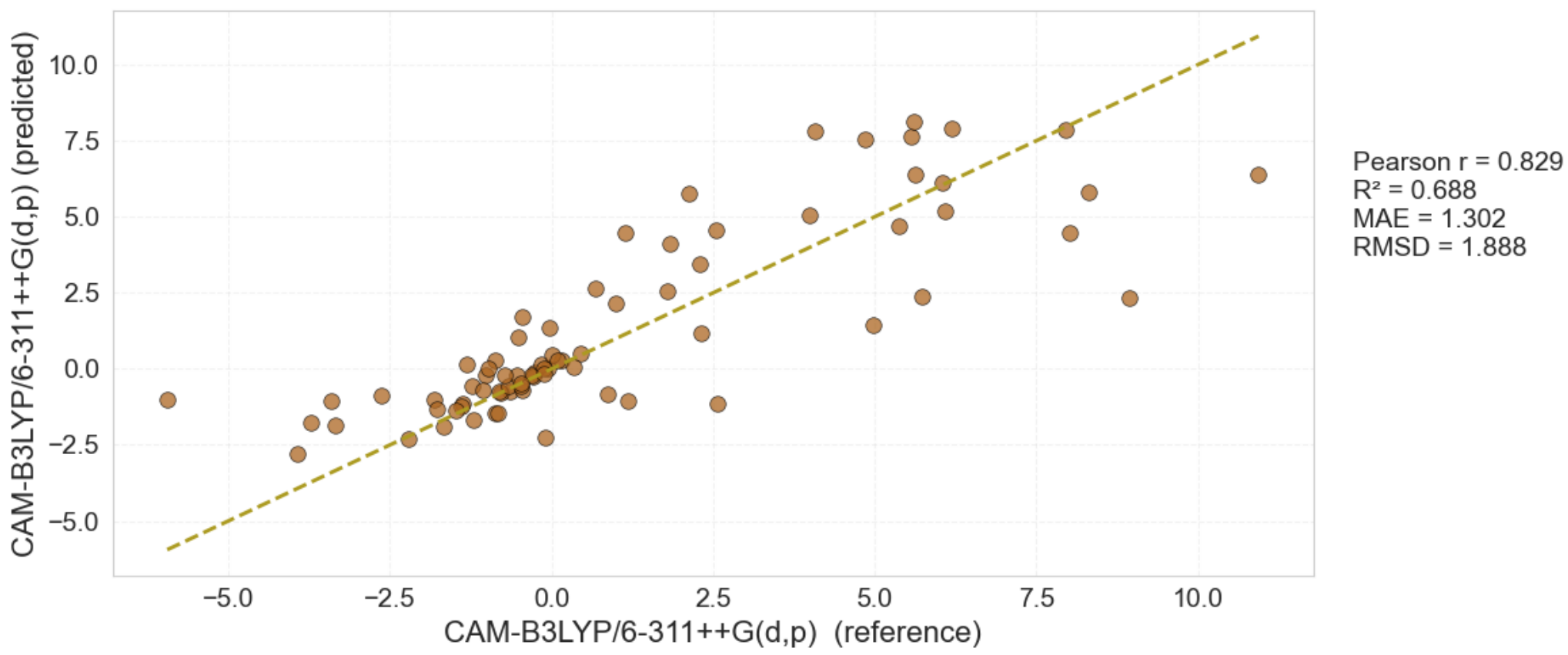

Figure S144: Parity plots of enthalpy values predicted using the Hist Gradient Boosting Regressor model with features derived from the CAM-B3LYP/6-311++G(d,p) method and the corresponding reference values.

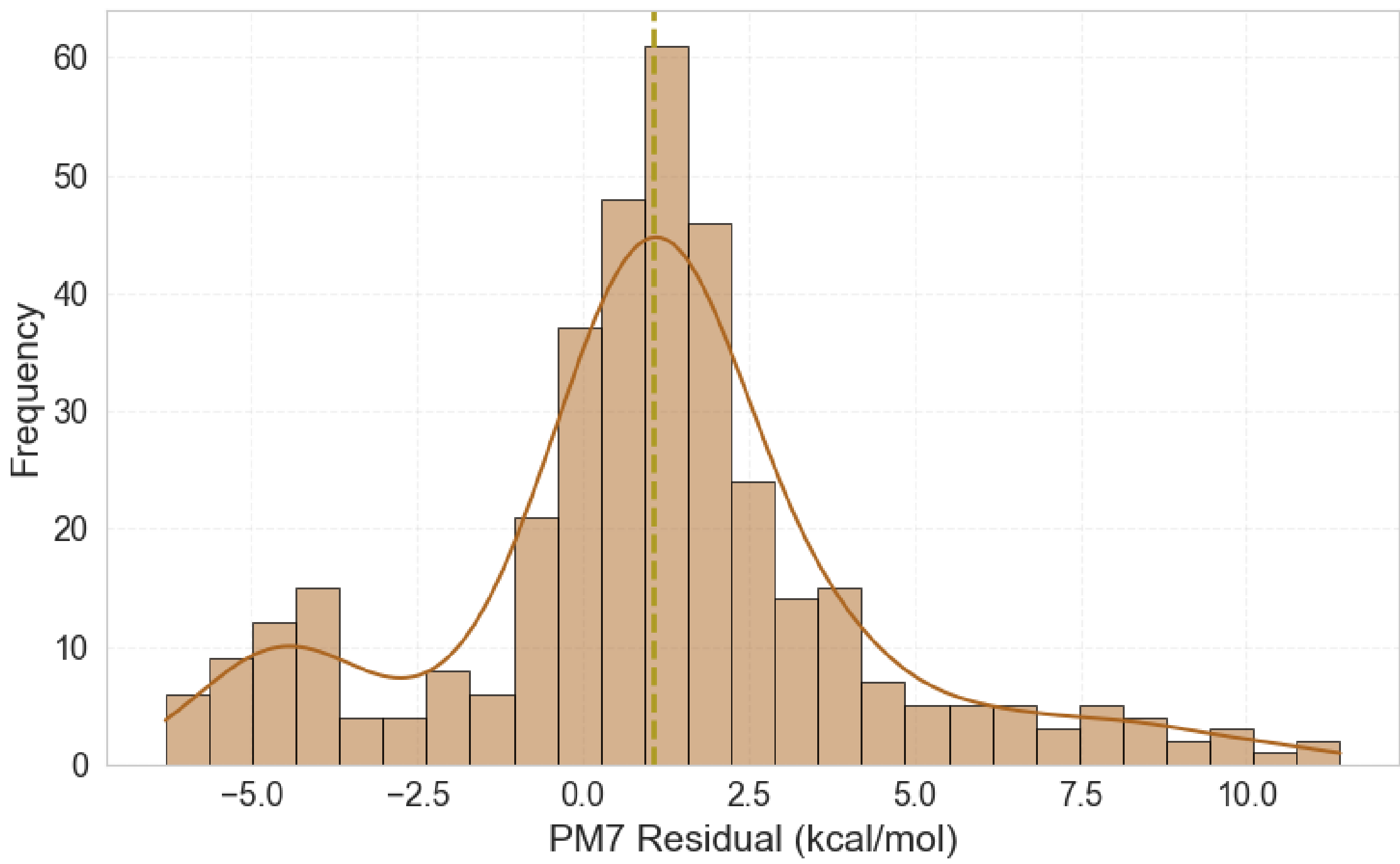

Figure S145: Residual distribution of the predicted enthalpy values obtained using the Hist Gradient Boosting Regressor model with features derived from the PM7 method, in comparison with the reference values.

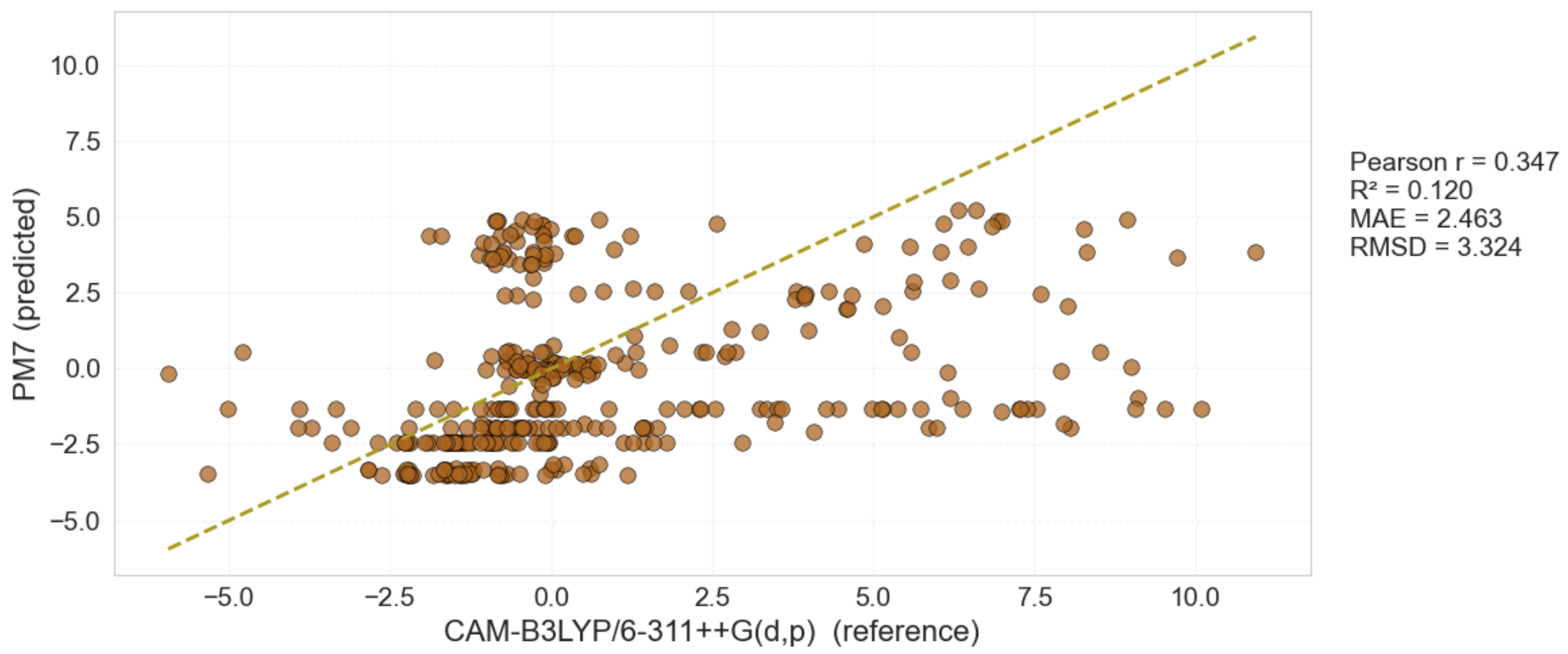

Figure S146: Parity plots of enthalpy values predicted using the Hist Gradient Boosting Regressor model with features derived from the PM7 method and the corresponding reference values.

S5.6 Bagging Regressor

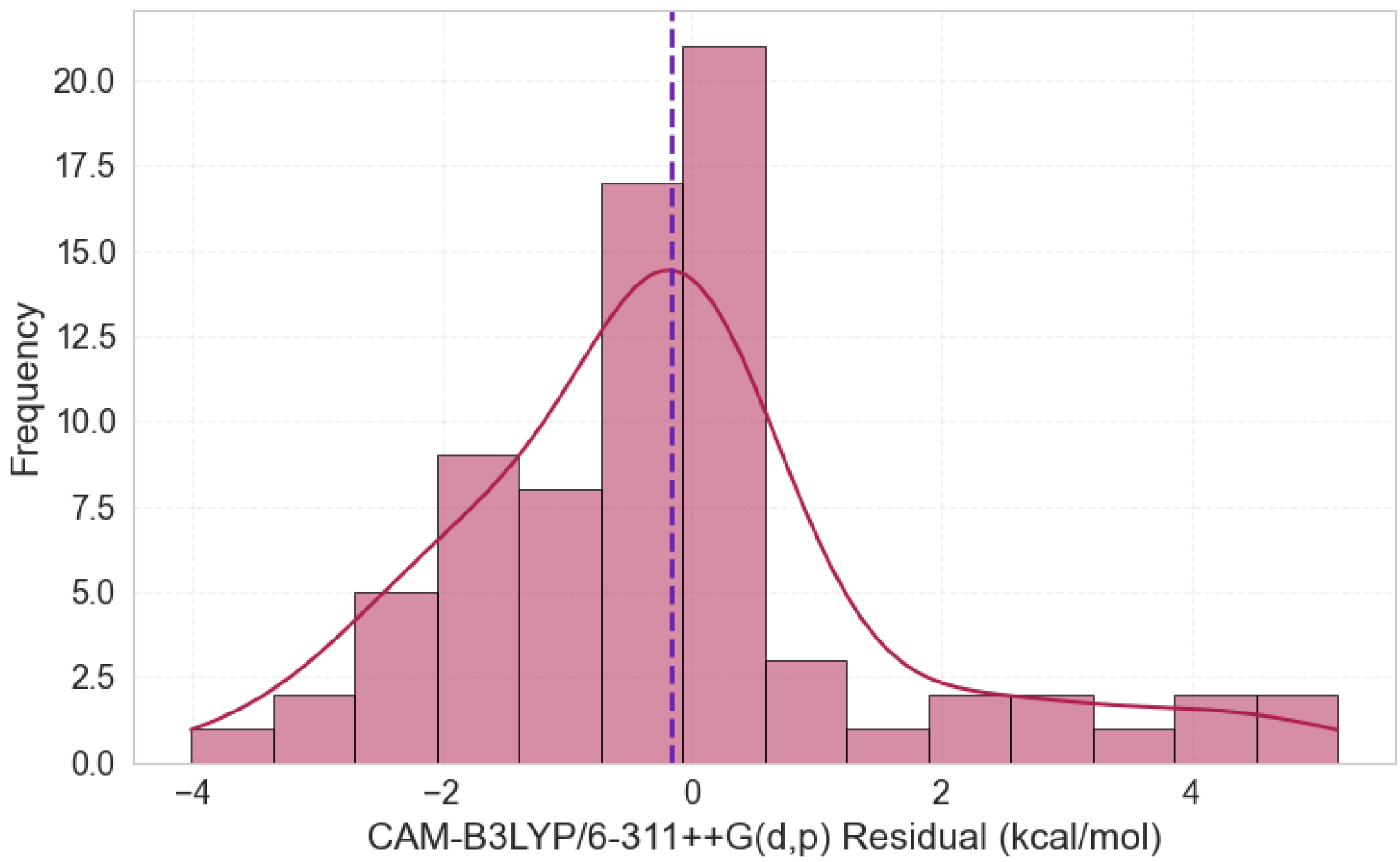

Figure S147: Residual distribution of the predicted enthalpy values obtained using the Bagging Regressor model with features derived from the CAM-B3LYP/6-311++G(d,p) method, in comparison with the reference values.

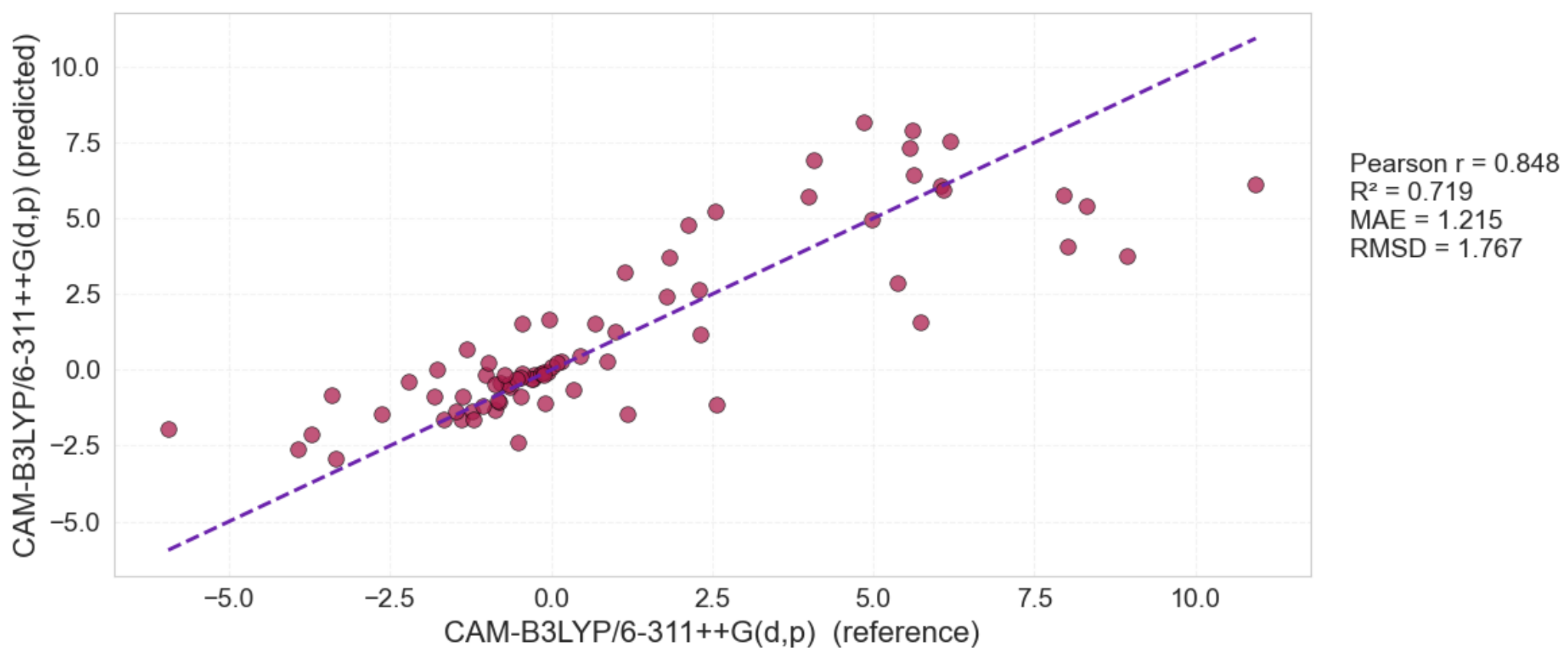

Figure S148: Parity plots of enthalpy values predicted using the Bagging Regressor model with features derived from the CAM-B3LYP/6-311++G(d,p) method and the corresponding reference values.

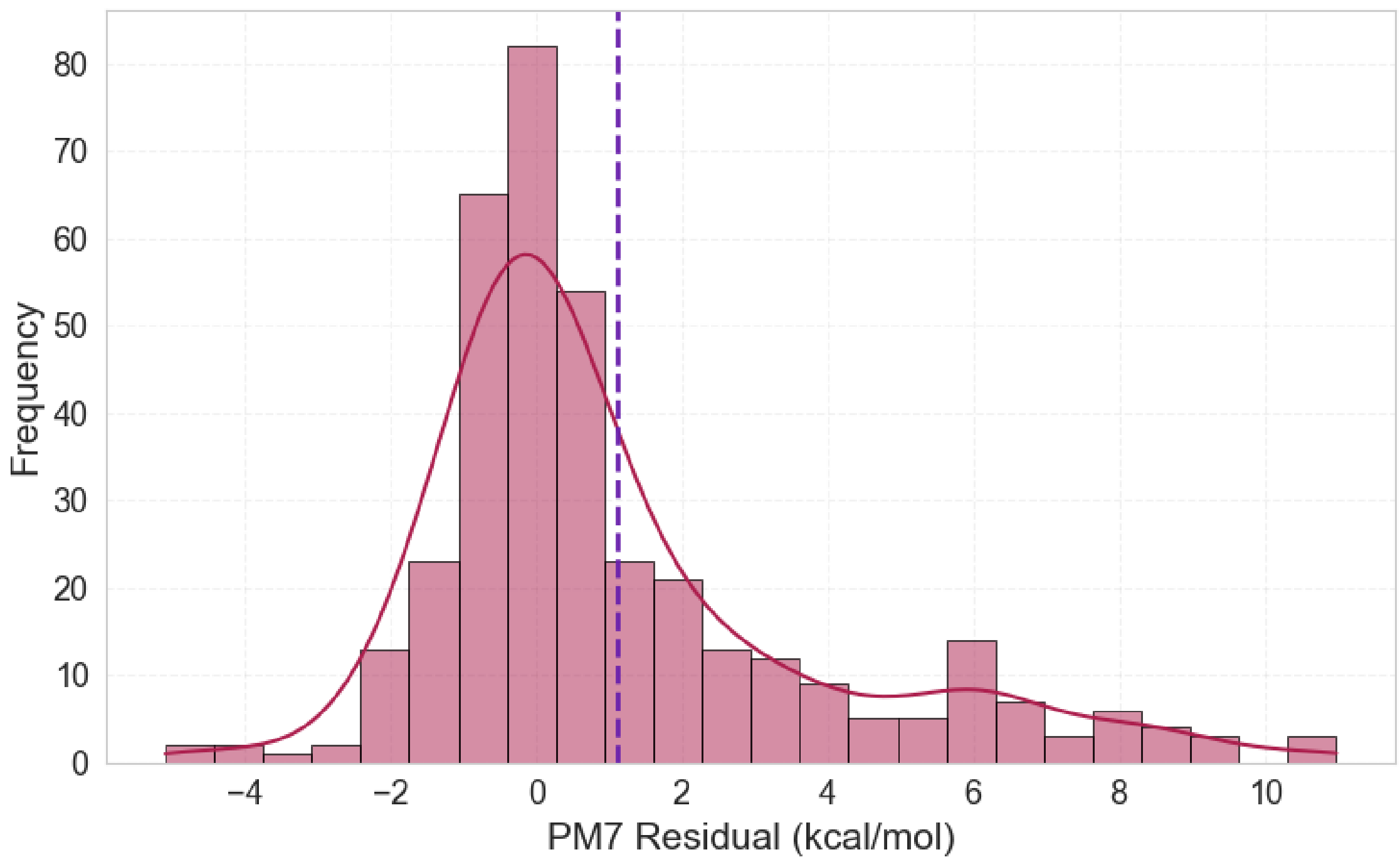

Figure S149: Residual distribution of the predicted enthalpy values obtained using the Bagging Regressor model with features derived from the PM7 method, in comparison with the reference values.

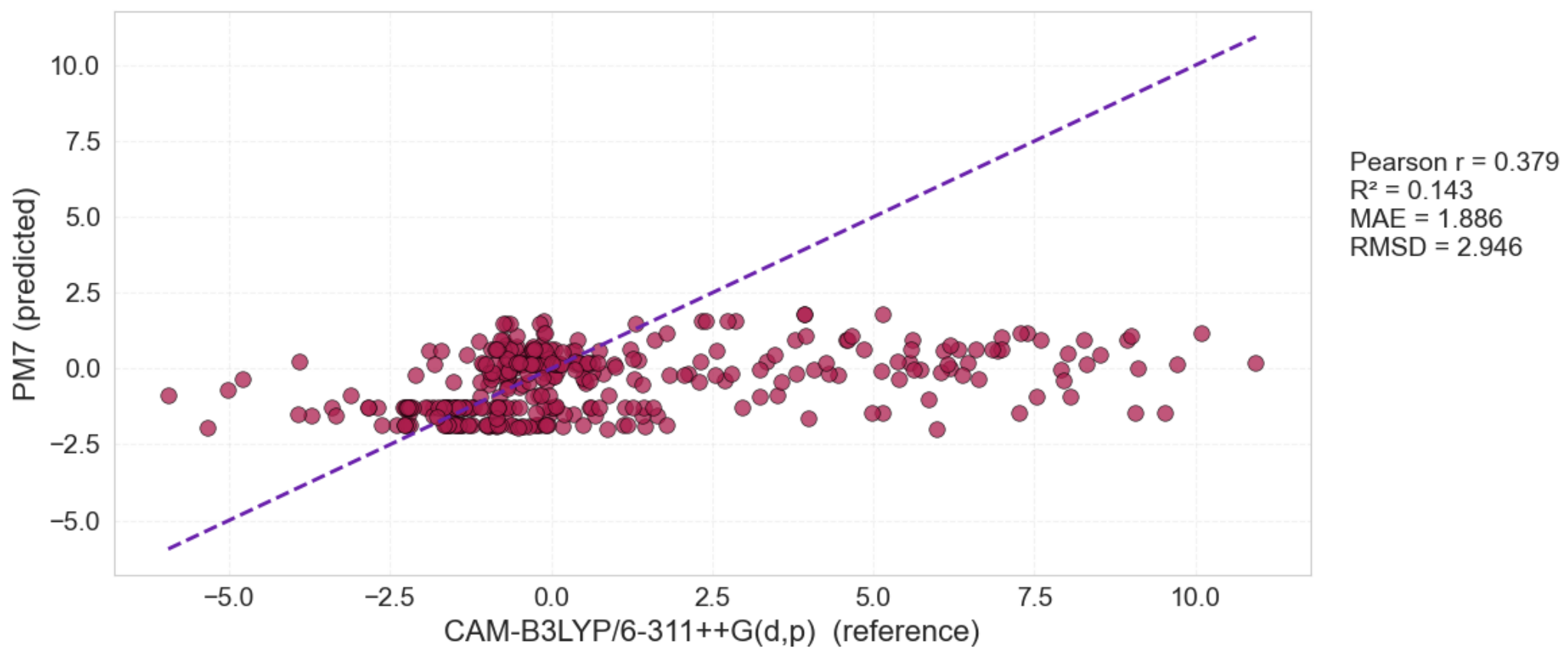

Figure S150: Parity plots of enthalpy values predicted using the Bagging Regressor model with features derived from the PM7 method and the corresponding reference values.

S5.7 Decision Tree Regressor

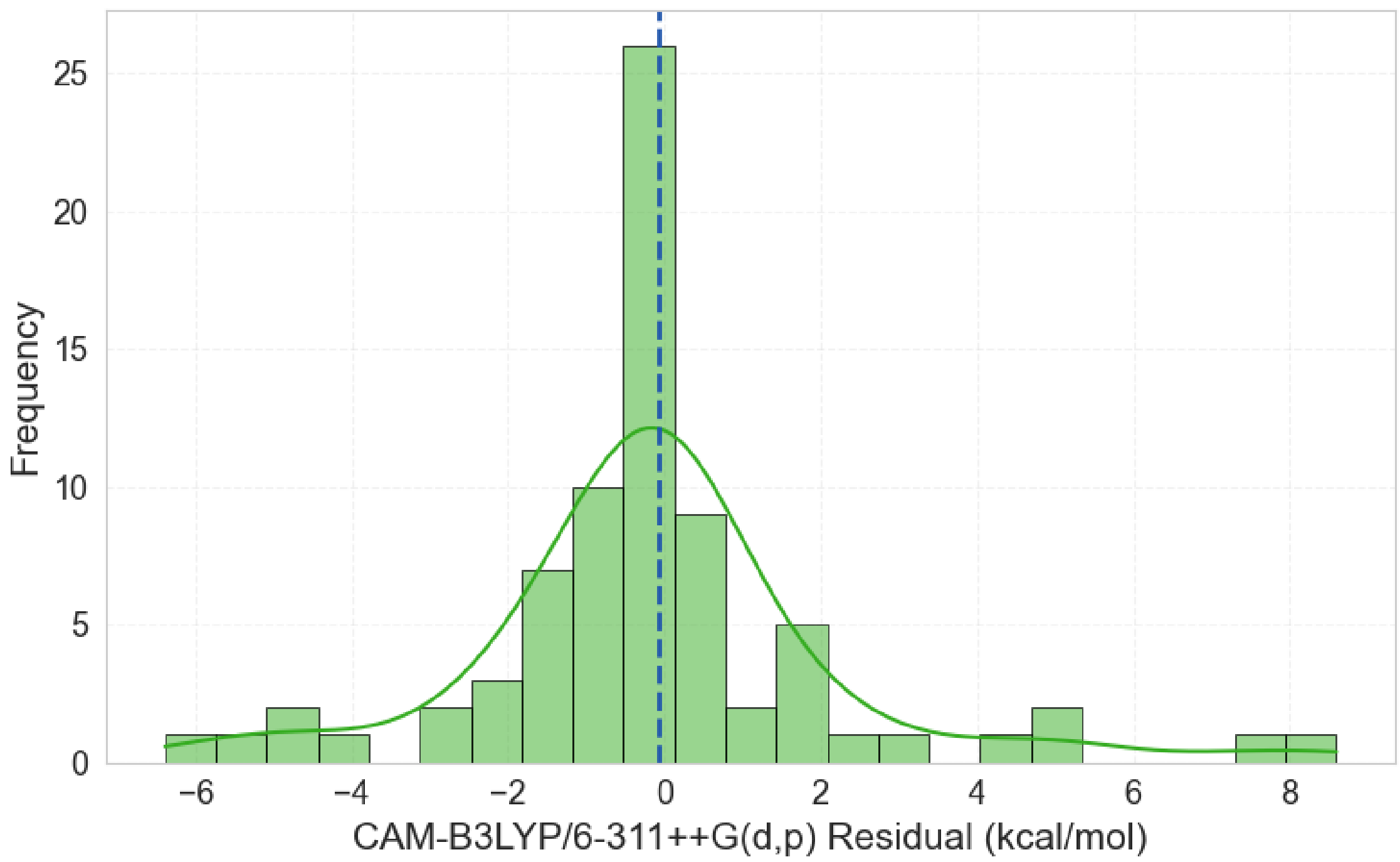

Figure S151: Residual distribution of the predicted enthalpy values obtained using the Decision Tree Regressor model with features derived from the CAM-B3LYP/6-311++G(d,p) method, in comparison with the reference values.

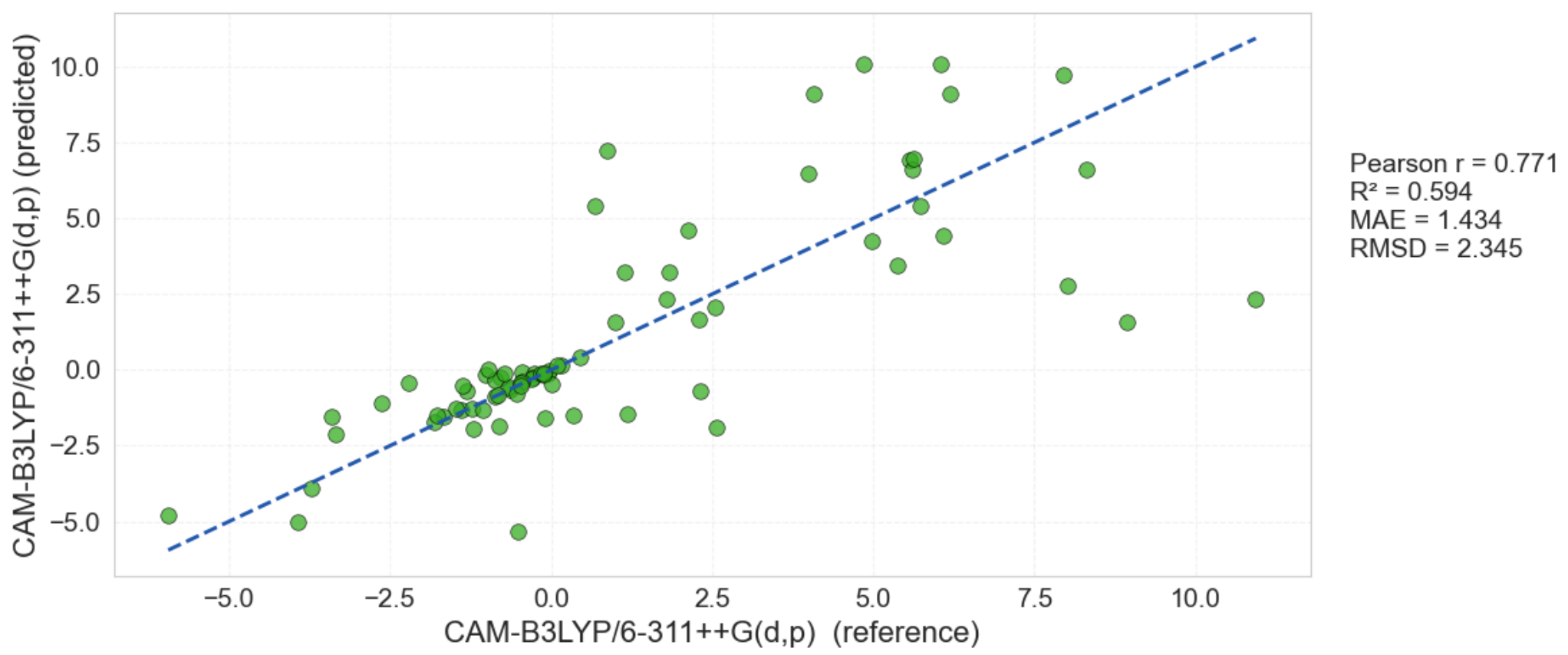

Figure S152: Parity plots of enthalpy values predicted using the Decision Tree Regressor model with features derived from the CAM-B3LYP/6-311++G(d,p) method and the corresponding reference values.

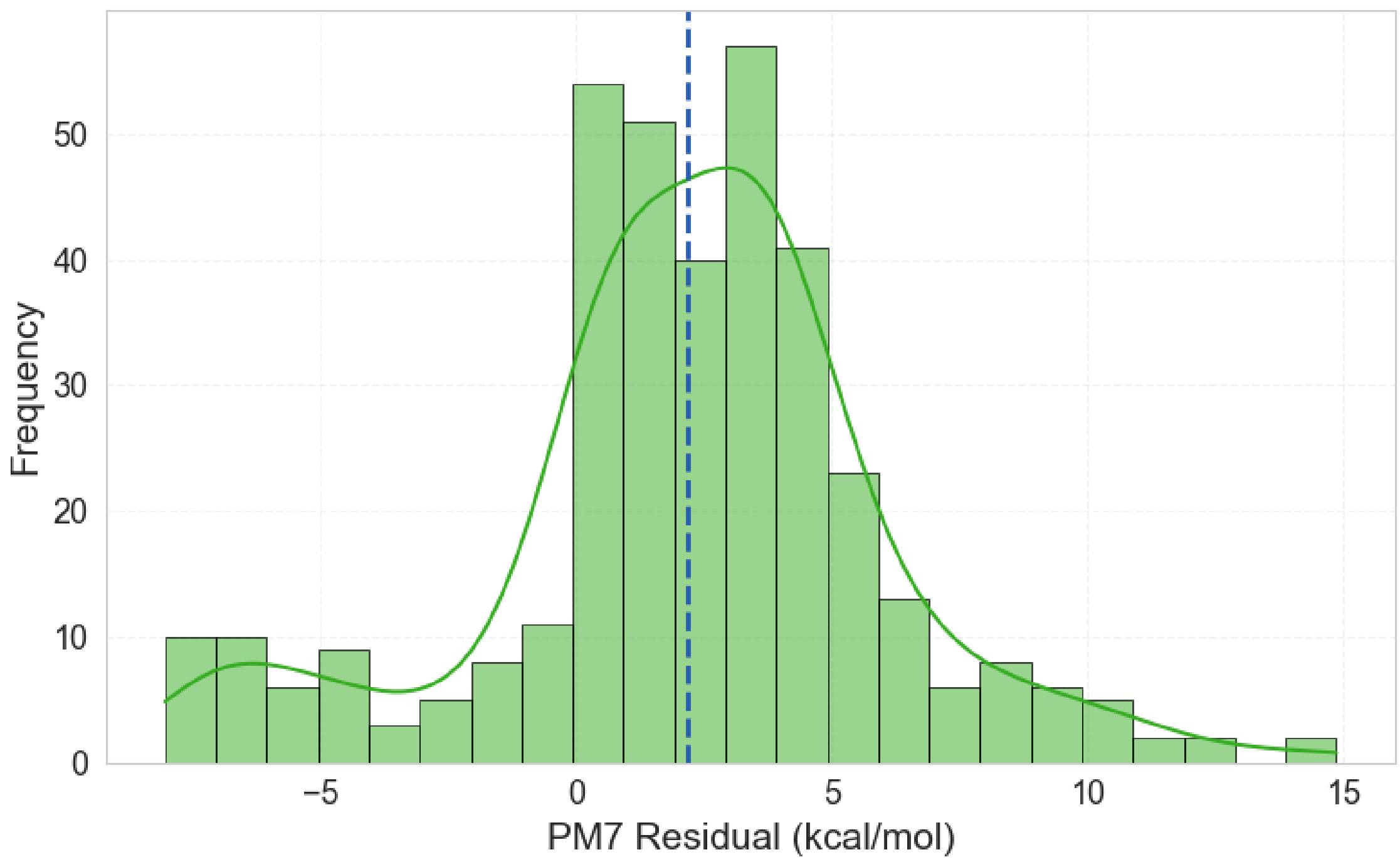

Figure S153: Residual distribution of the predicted enthalpy values obtained using the Decision Tree Regressor with features derived from the PM7 method, in comparison with the reference values.

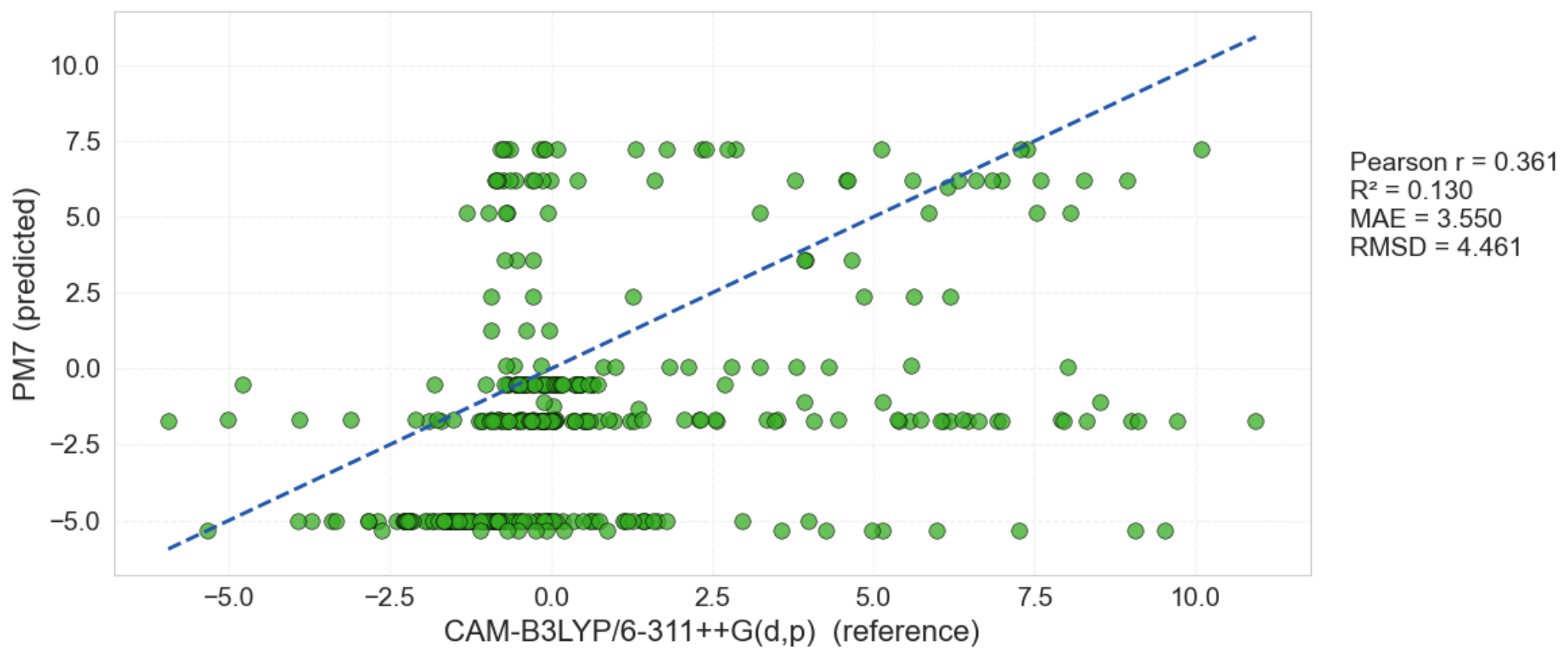

Figure S154: Parity plots of enthalpy values predicted using the Decision Tree Regressor model with features derived from the PM7 method and the corresponding reference values.

S6. Outlier treatment using Robust Covariance, One-Class SVM, Isolation Forest, Local Outlier Factor (LOF), and One-Class SVM within ensemble frameworks. Correlation plots as a function of contamination level and correlation plots with performance metrics (Pearson's r, MAE, RMSD).

S6.1 One-Class SVM (RBF)

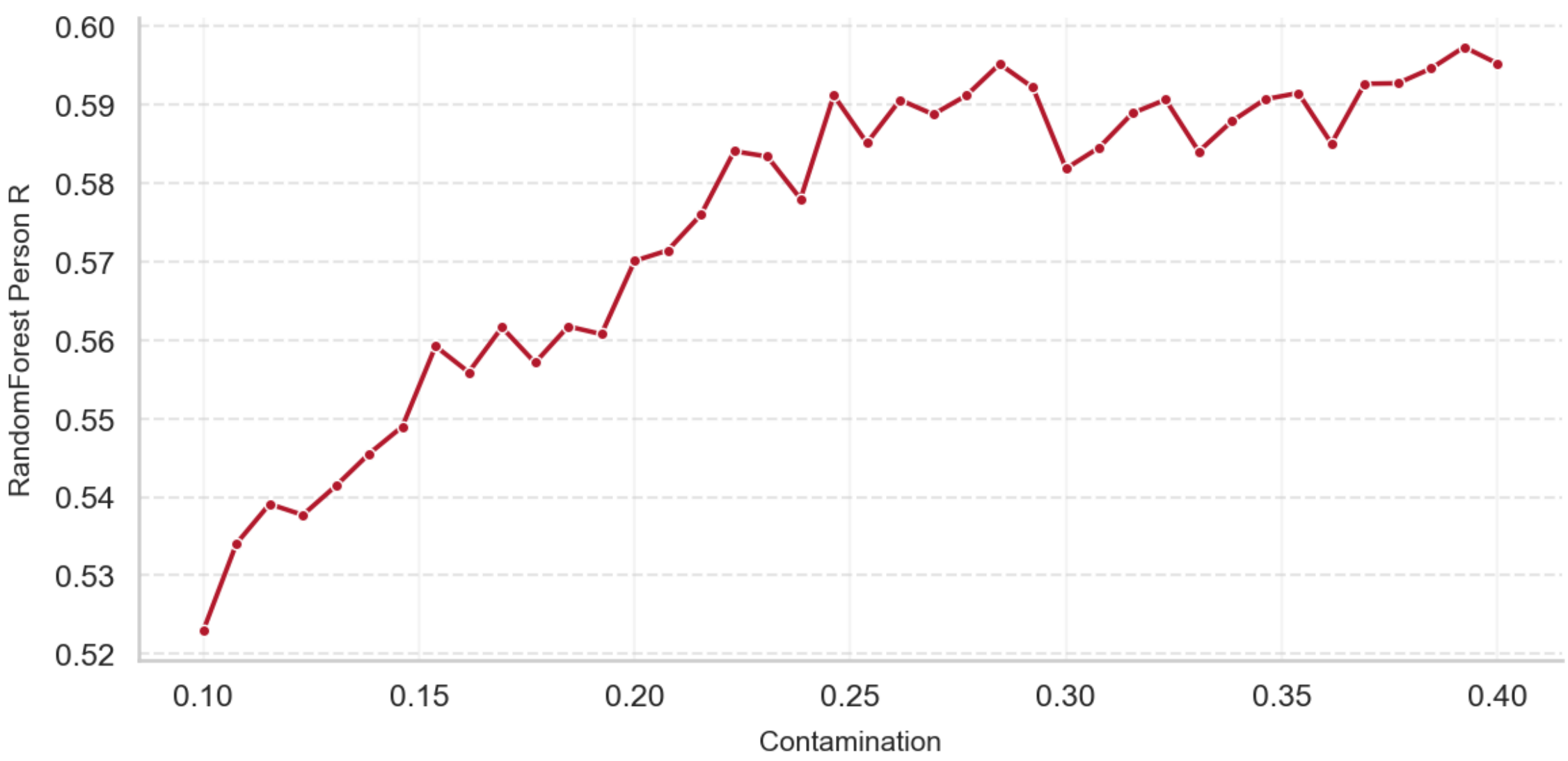

Figure S155: Correlation plot obtained between the data generated by the Random Forest model trained with PM7-derived features and the reference values as a function of data contamination, using One-Class SVM (RBF) for outlier treatment.

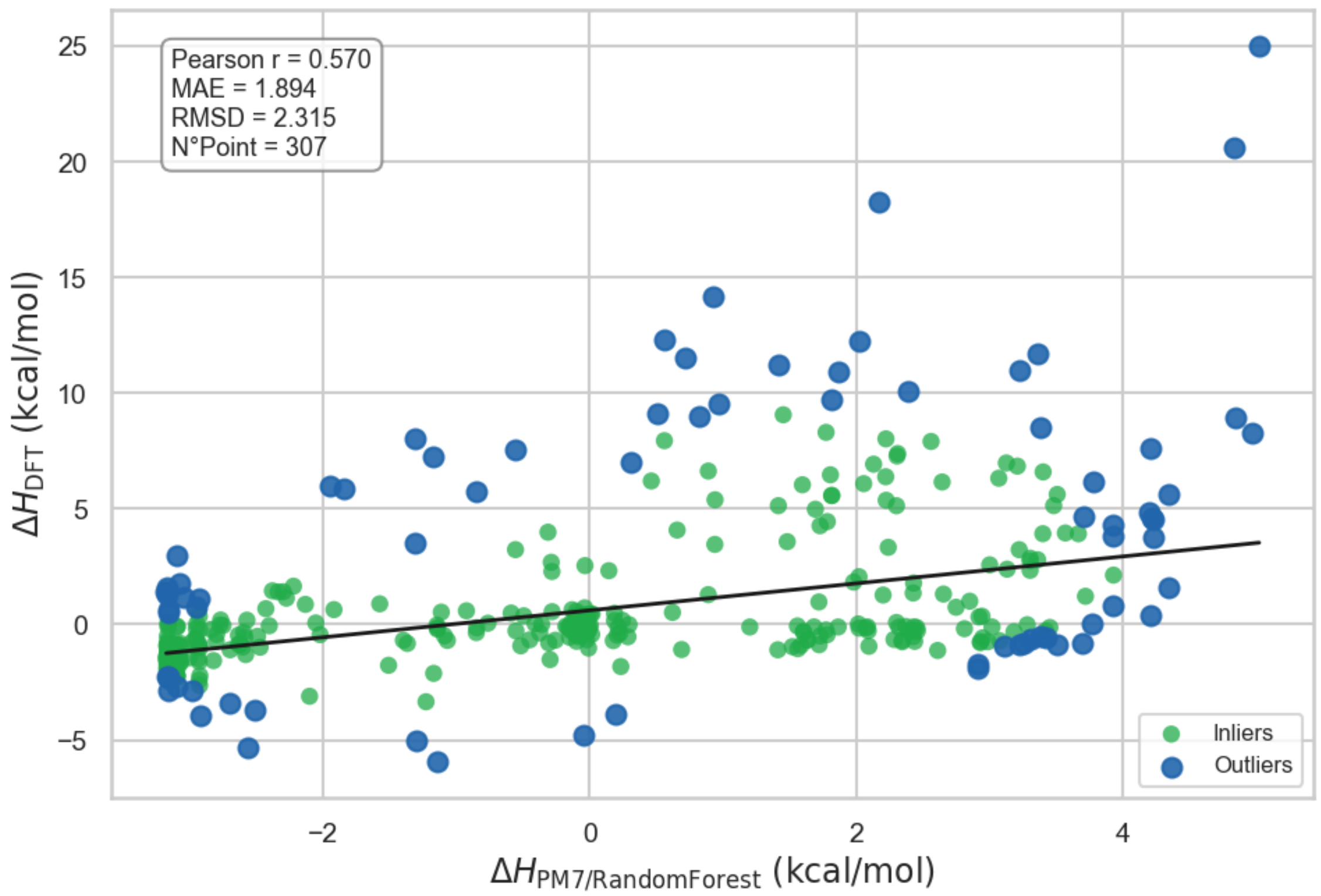

Figure S156: Scatter plots between reference DFT values and the values generated by the Random Forest model trained with PM7-derived features, applying One-Class SVM (RBF) outlier treatment with a contamination level of 0.20.

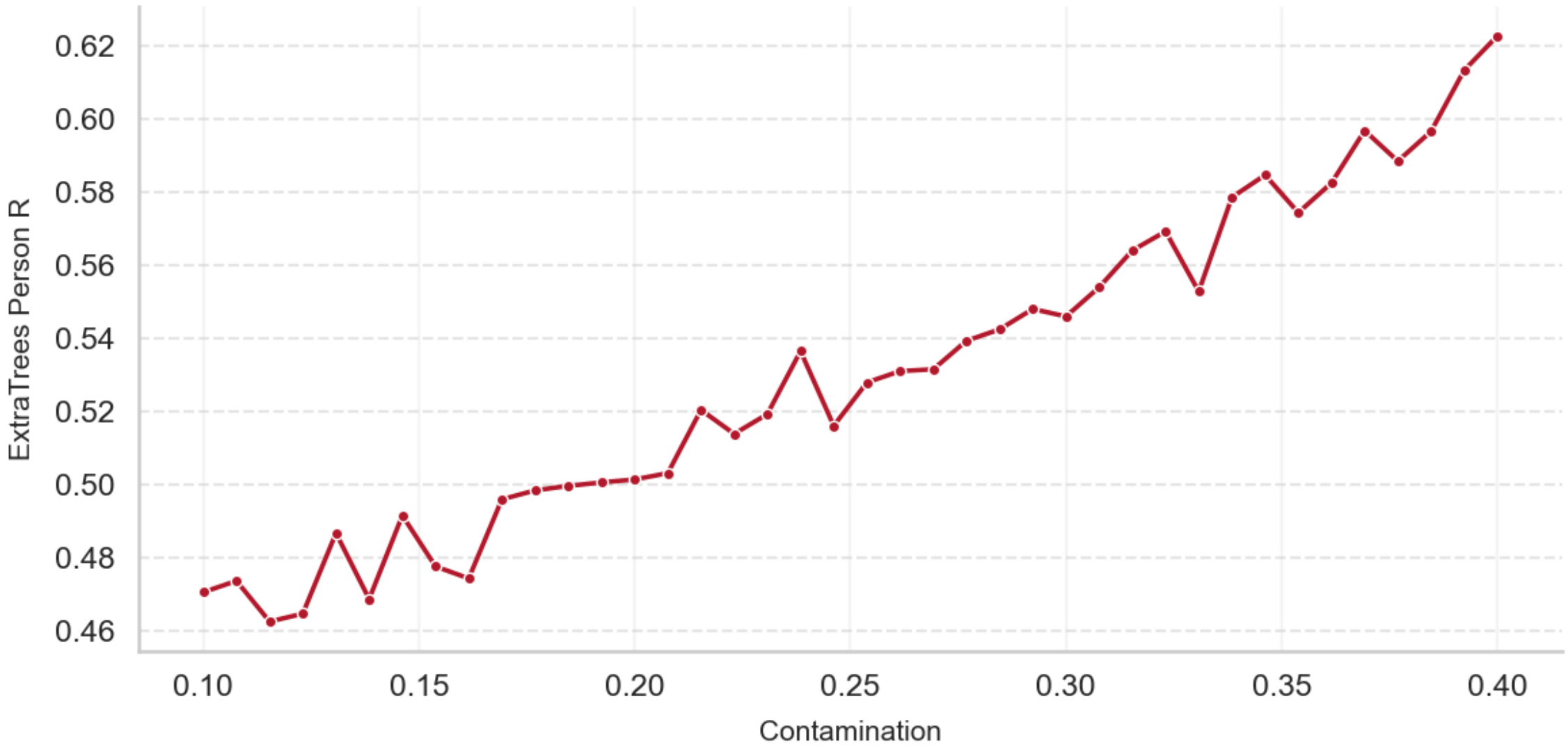

Figure S157: Correlation plot obtained between the data generated by the Extra Trees Regressor model trained with PM7-derived features and the reference values as a function of data contamination, using One-Class SVM (RBF) for outlier treatment.

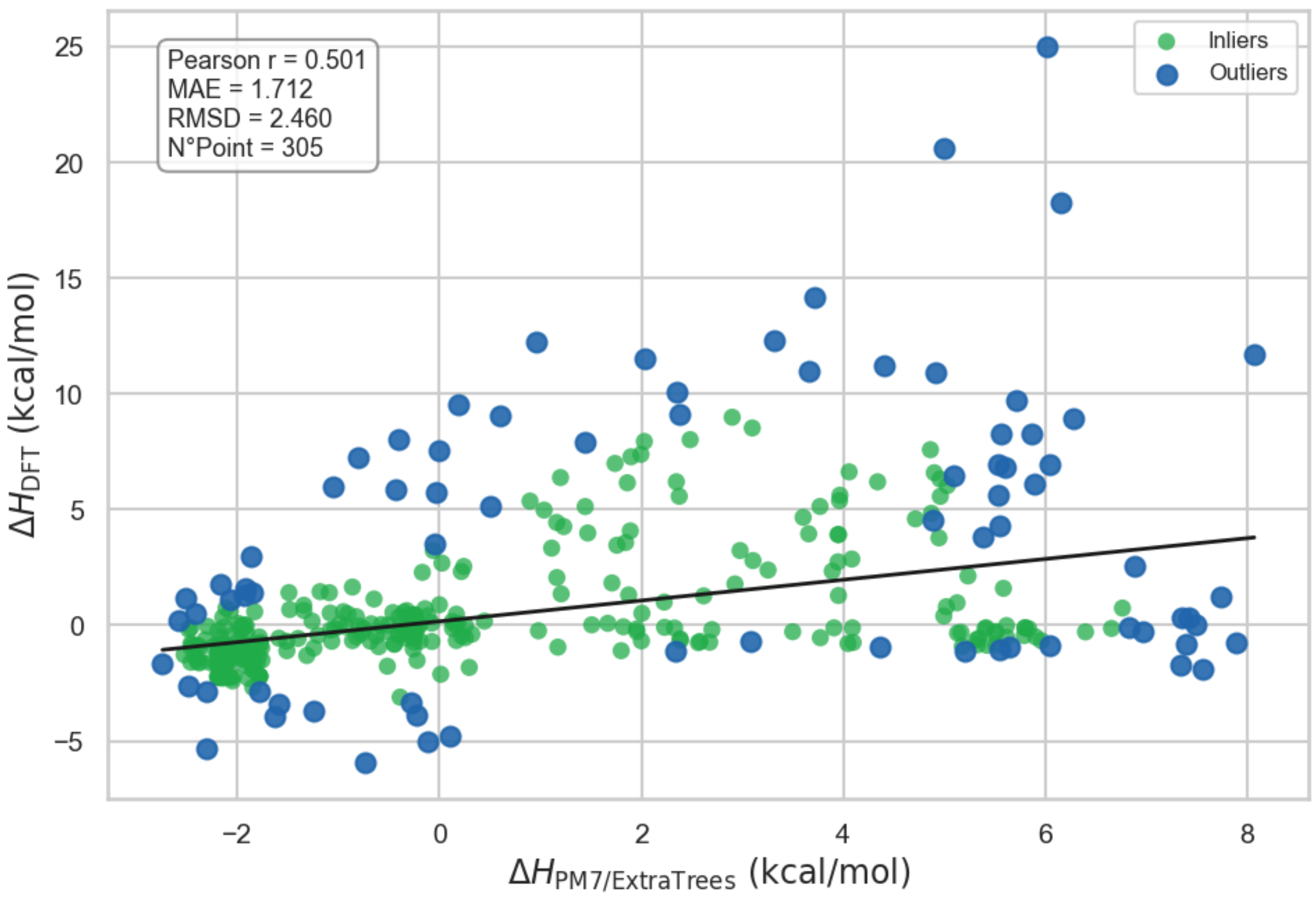

Figure S158: Scatter plots between reference DFT values and the values generated by the Extra Trees Regressor model trained with PM7-derived features, applying One-Class SVM (RBF) outlier treatment with a contamination level of 0.20.

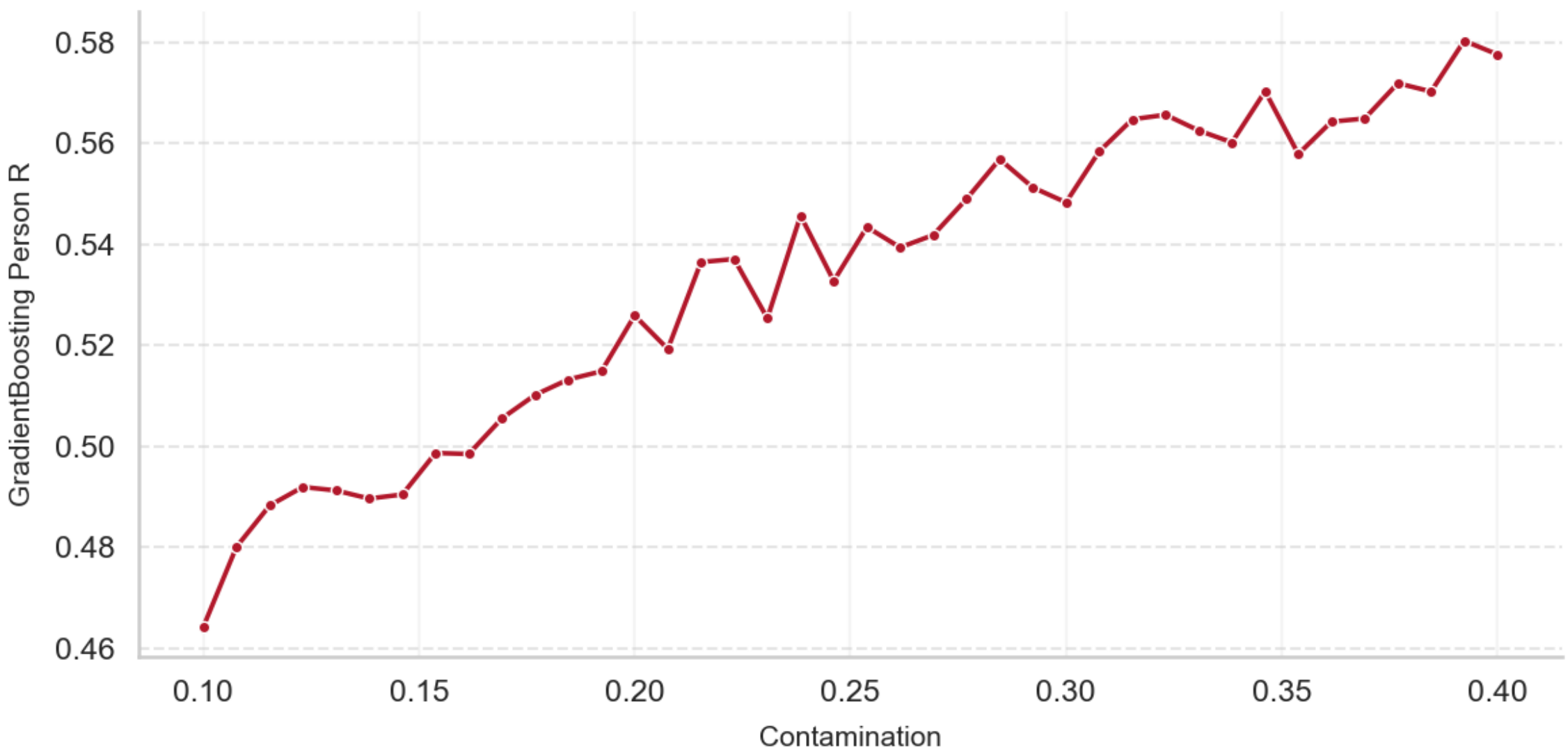

Figure S159: Correlation plot obtained between the data generated by the Gradient Boosting Regressor model trained with PM7-derived features and the reference values as a function of data contamination, using One-Class SVM (RBF) for outlier treatment.

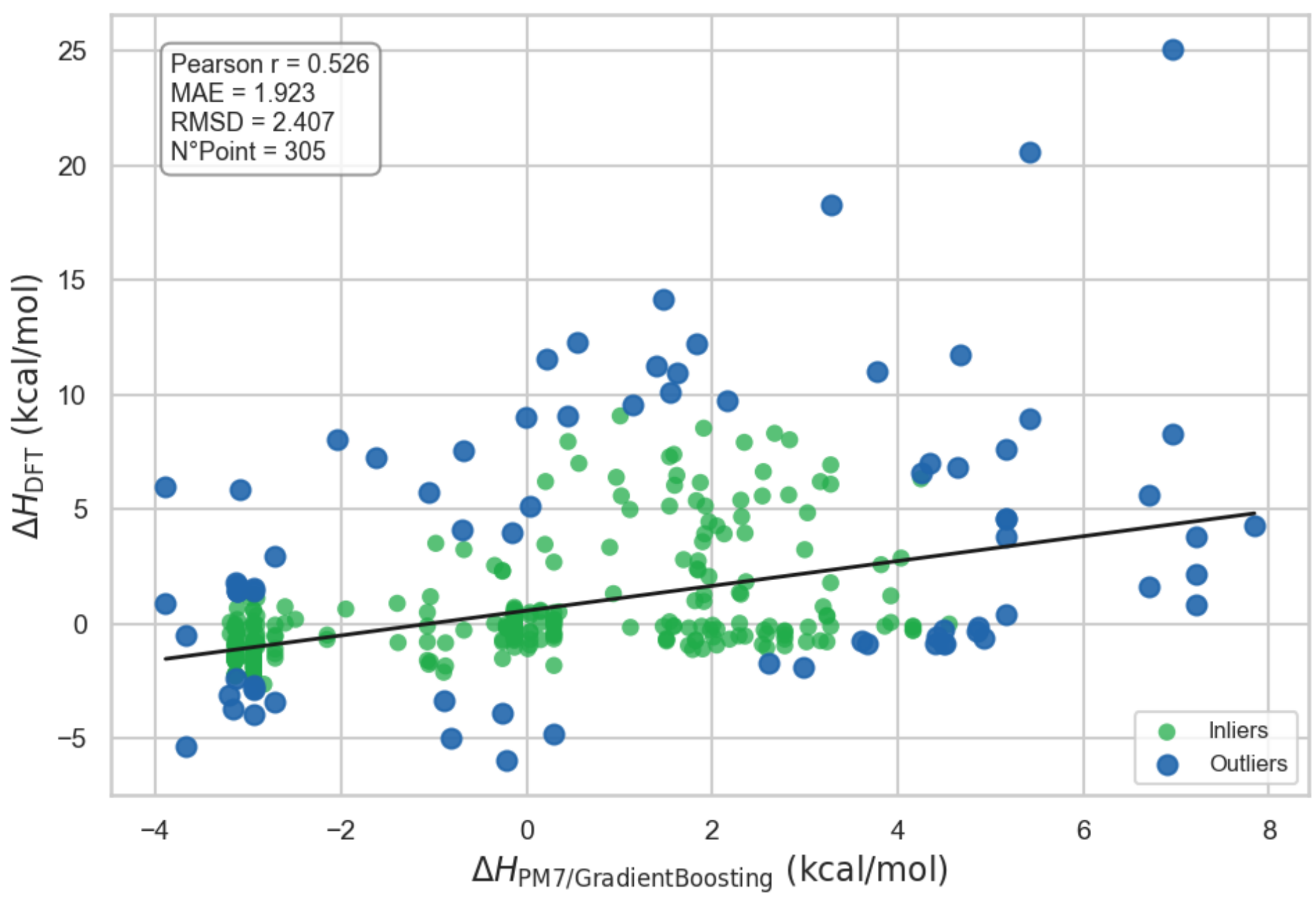

Figure S160: Scatter plots between reference DFT values and the values generated by the Boosting Regressor model trained with PM7-derived features, applying One-Class SVM (RBF) outlier treatment with a contamination level of 0.20.

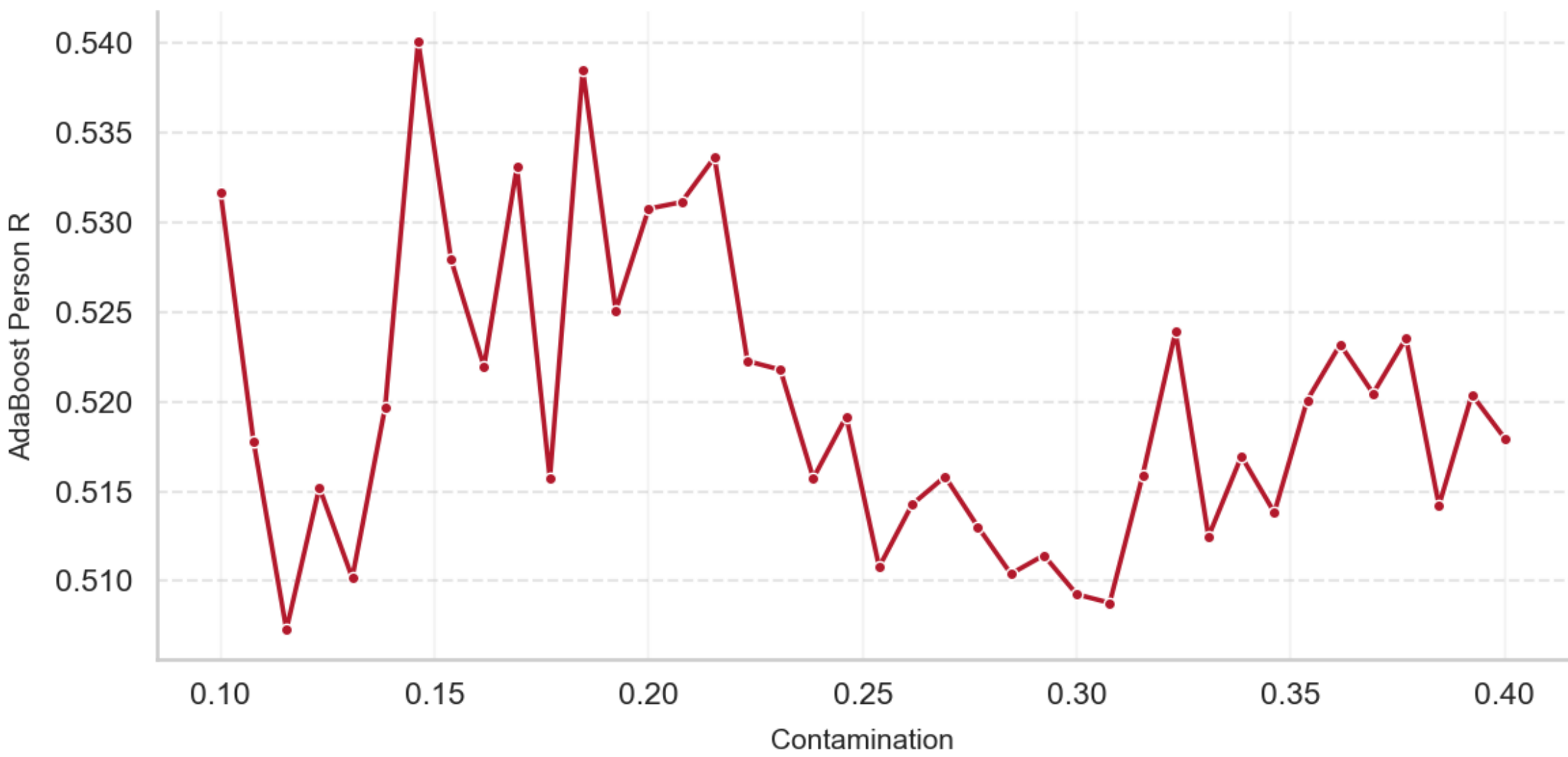

Figure S161: Correlation plot obtained between the data generated by the Ada Boost Regressor model trained with PM7-derived features and the reference values as a function of data contamination, using One-Class SVM (RBF) for outlier treatment.

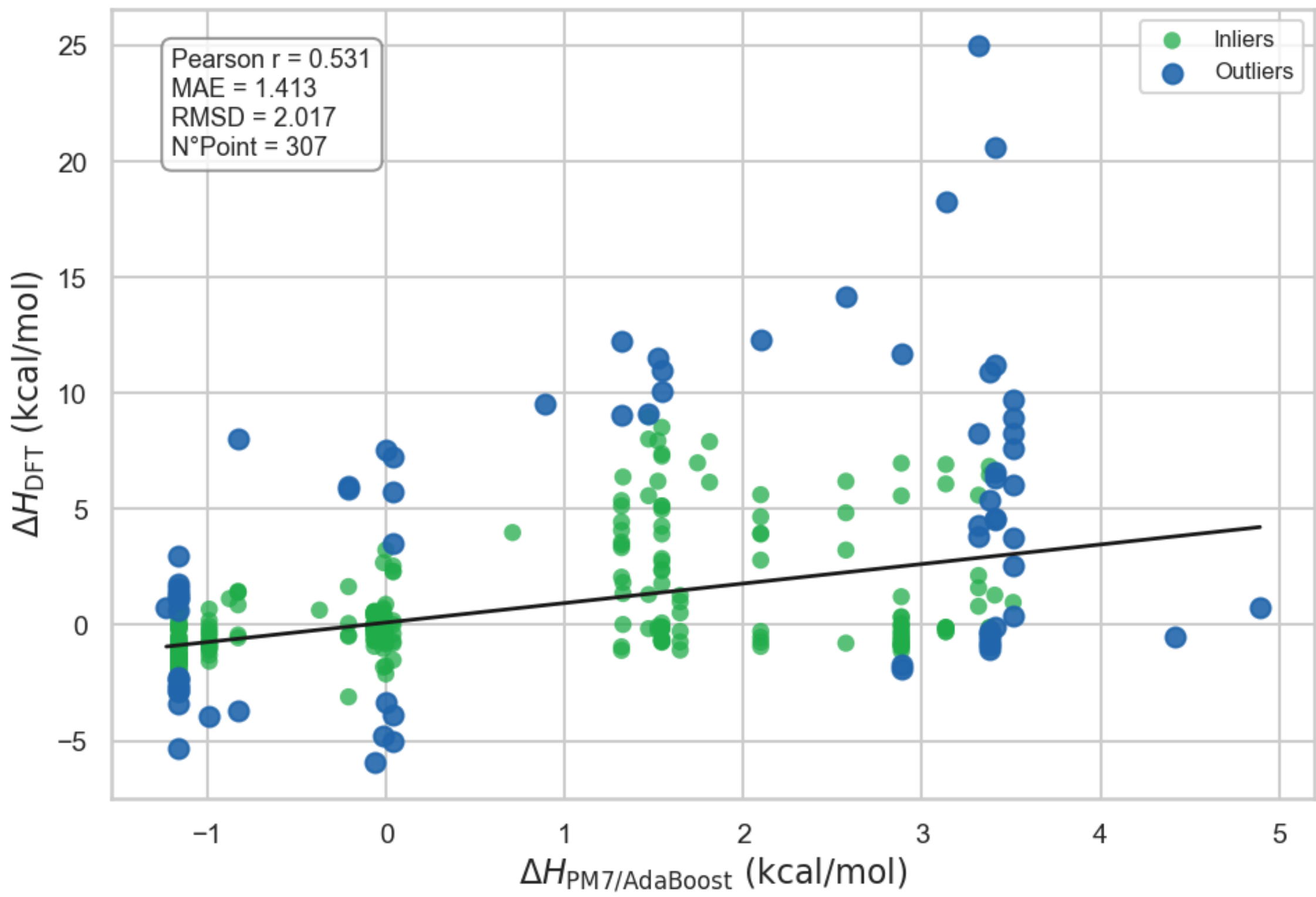

Figure S162: Scatter plots between reference DFT values and the values generated by the Ada Boost Regressor model trained with PM7-derived features, applying One-Class SVM (RBF) outlier treatment with a contamination level of 0.20.

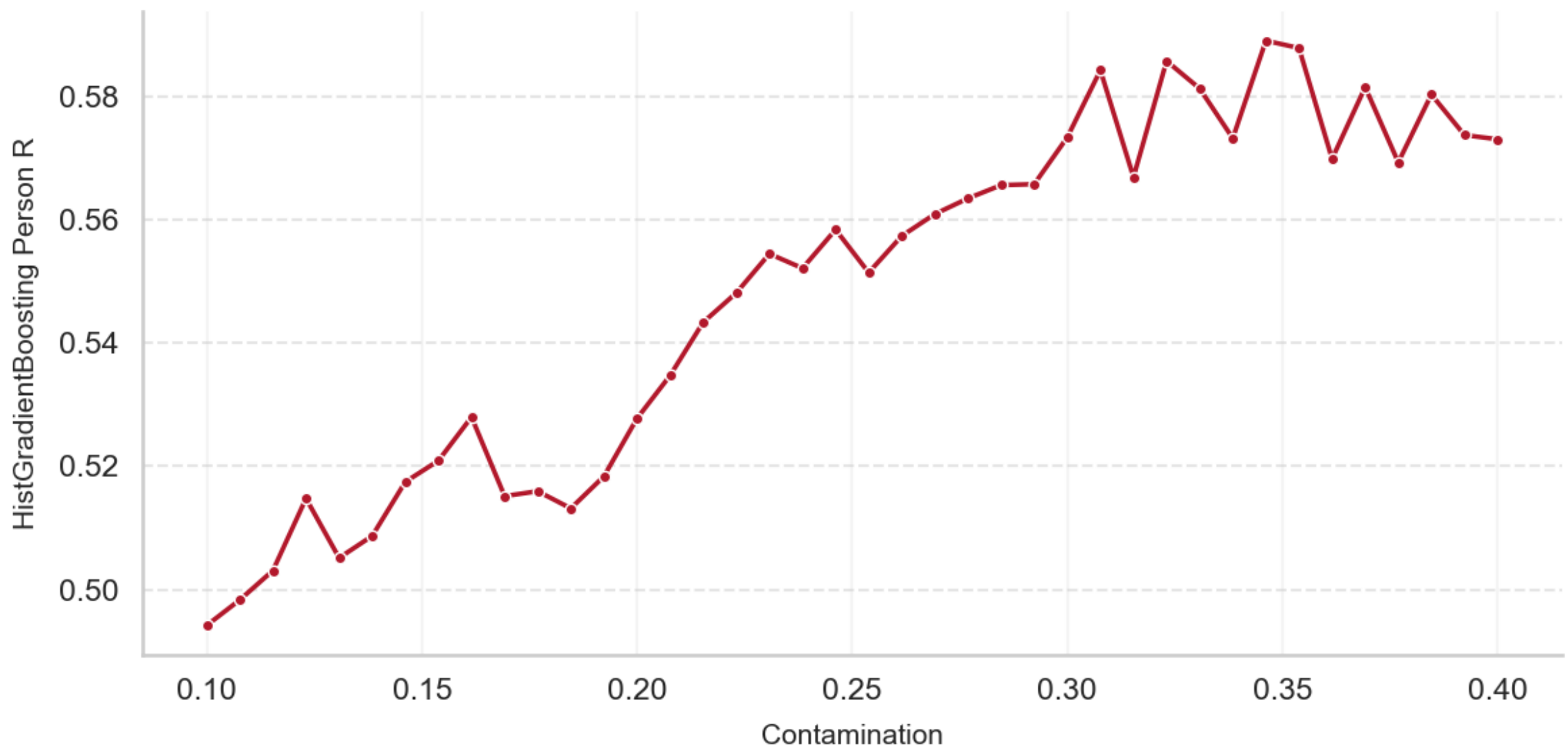

Figure S163: Correlation plot obtained between the data generated by the Hist Gradient Boosting model trained with PM7-derived features and the reference values as a function of data contamination, using One-Class SVM (RBF) for outlier treatment.

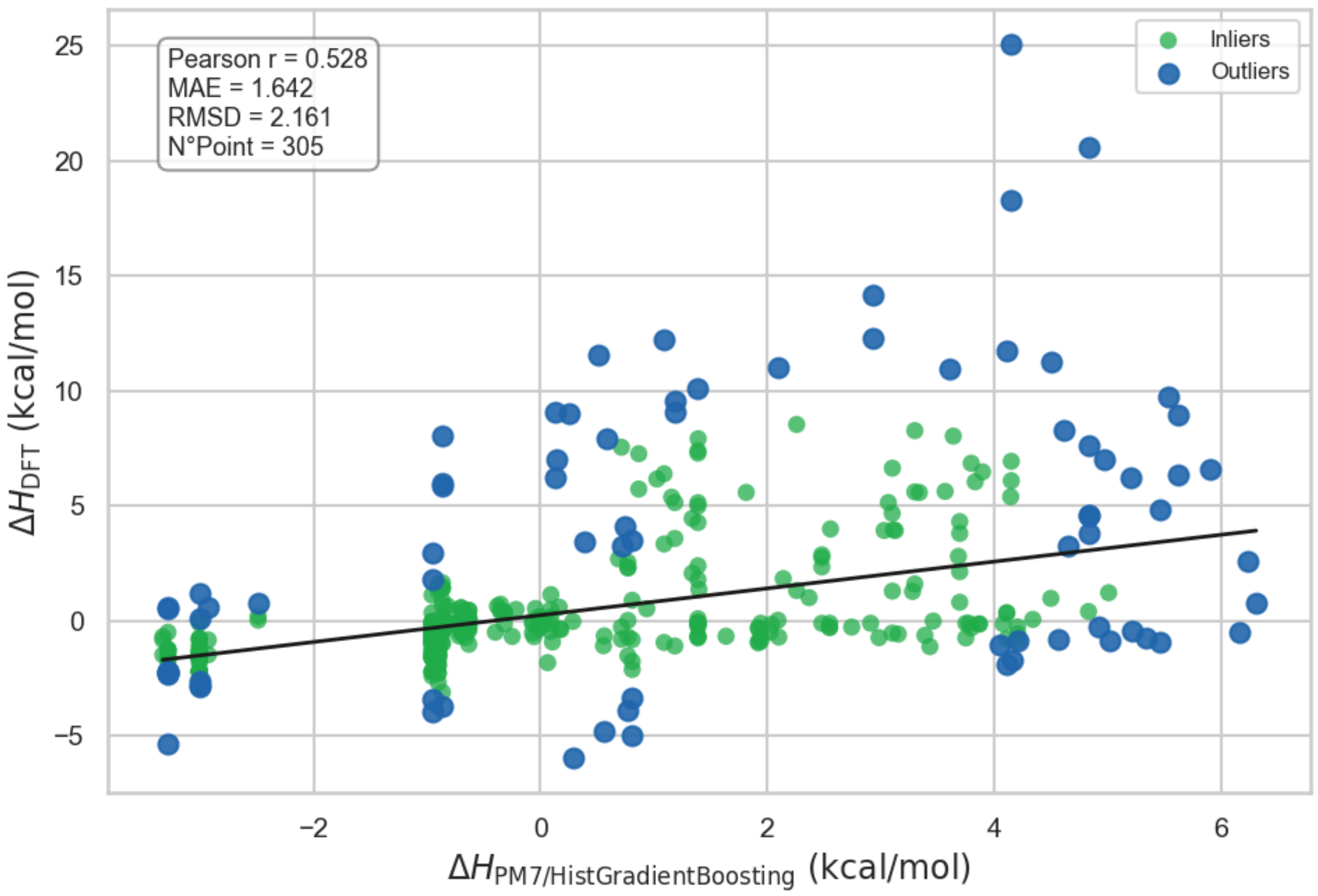

Figure S164: Scatter plots between reference DFT values and the values generated by the Hist Gradient Boosting model trained with PM7-derived features, applying One-Class SVM (RBF) outlier treatment with a contamination level of 0.20.

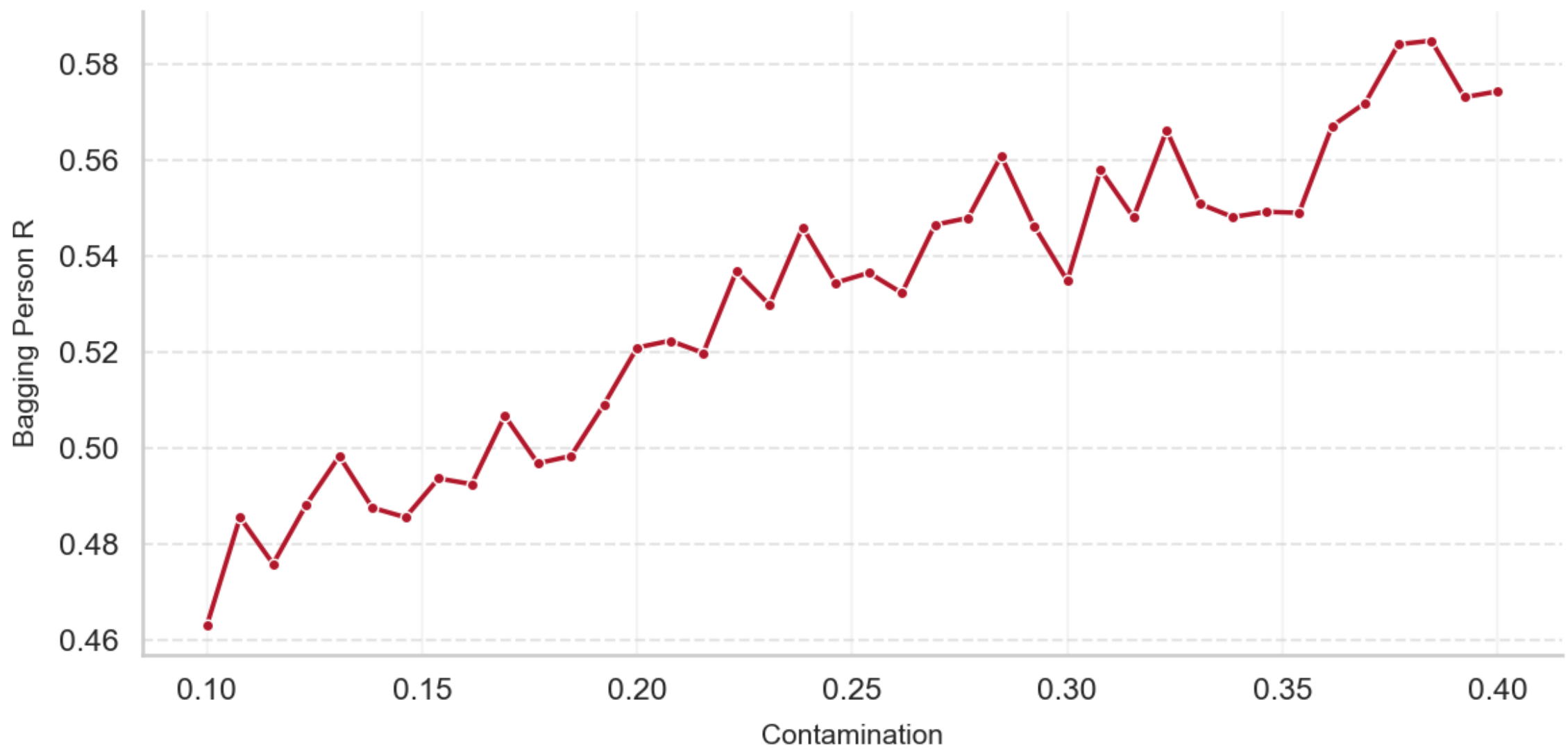

Figure S165: Correlation plot obtained between the data generated by the Bagging Regressor model trained with PM7-derived features and the reference values as a function of data contamination, using One-Class SVM (RBF) for outlier treatment.

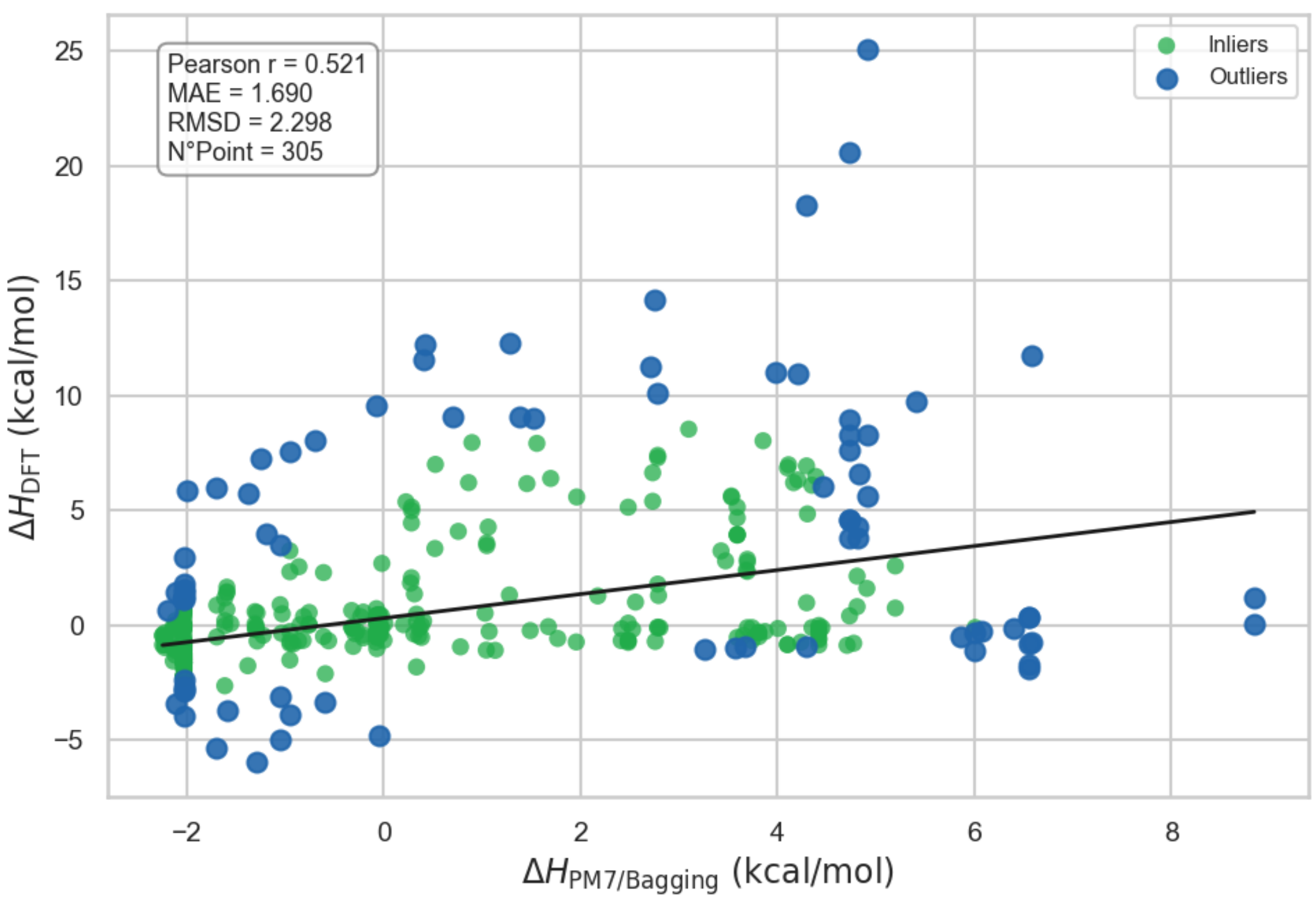

Figure S166: Scatter plots between reference DFT values and the values generated by the Bagging Regressor model trained with PM7-derived features, applying One-Class SVM (RBF) outlier treatment with a contamination level of 0.20.

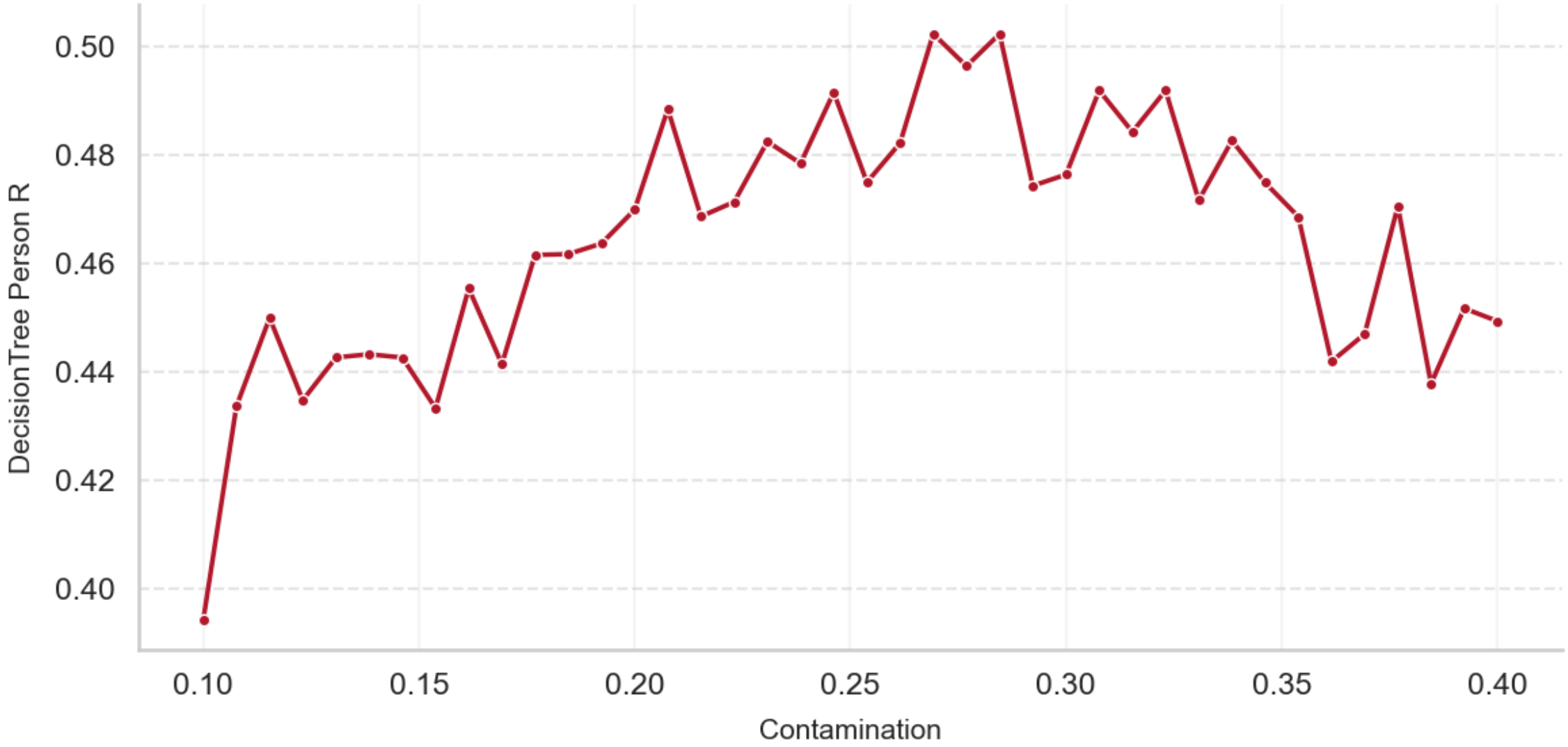

Figure S167: Correlation plot obtained between the data generated by the Decision Tree Regressor model trained with PM7-derived features and the reference values as a function of data contamination, using One-Class SVM (RBF) for outlier treatment.

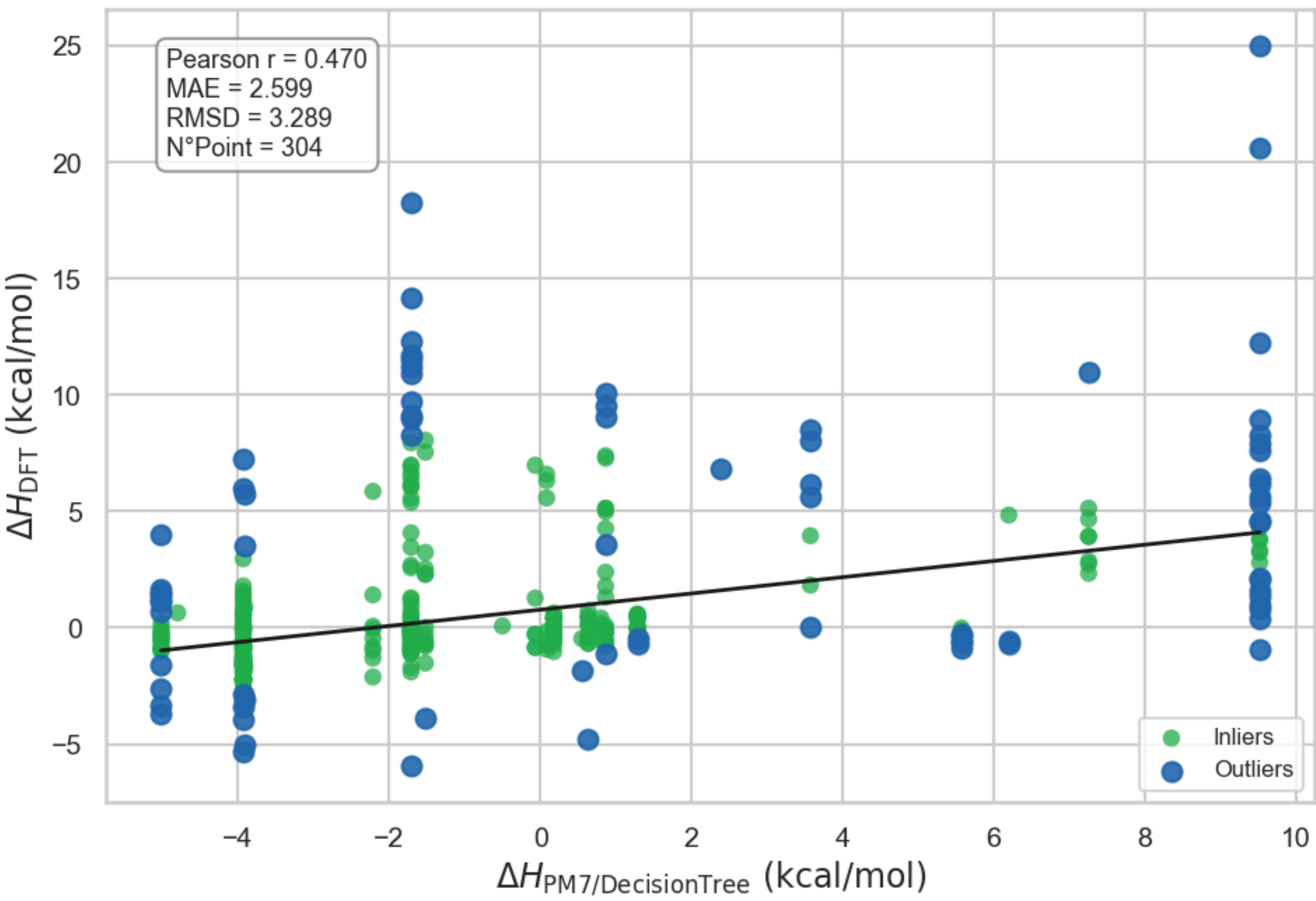

Figure S168: Scatter plots between reference DFT values and the values generated by the the Decision Tree Regressor model trained with PM7-derived features, applying One-Class SVM (RBF) outlier treatment with a contamination level of 0.20.

S6.2 One-Class SVM (SGD)

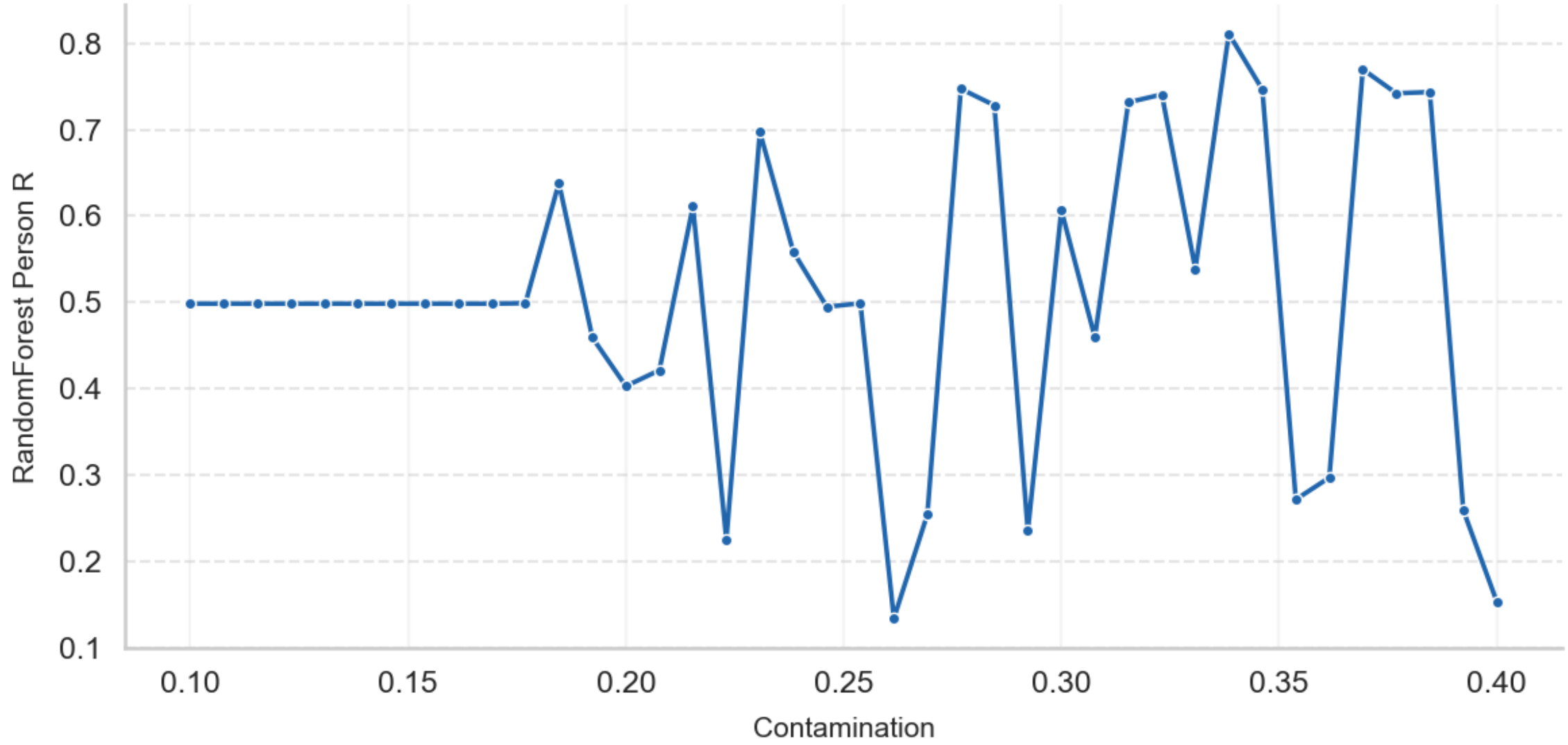

Figure S169: Correlation plot obtained between the data generated by the Random Forest model trained with PM7-derived features and the reference values as a function of data contamination, using One-Class SVM (SGD)for outlier treatment.

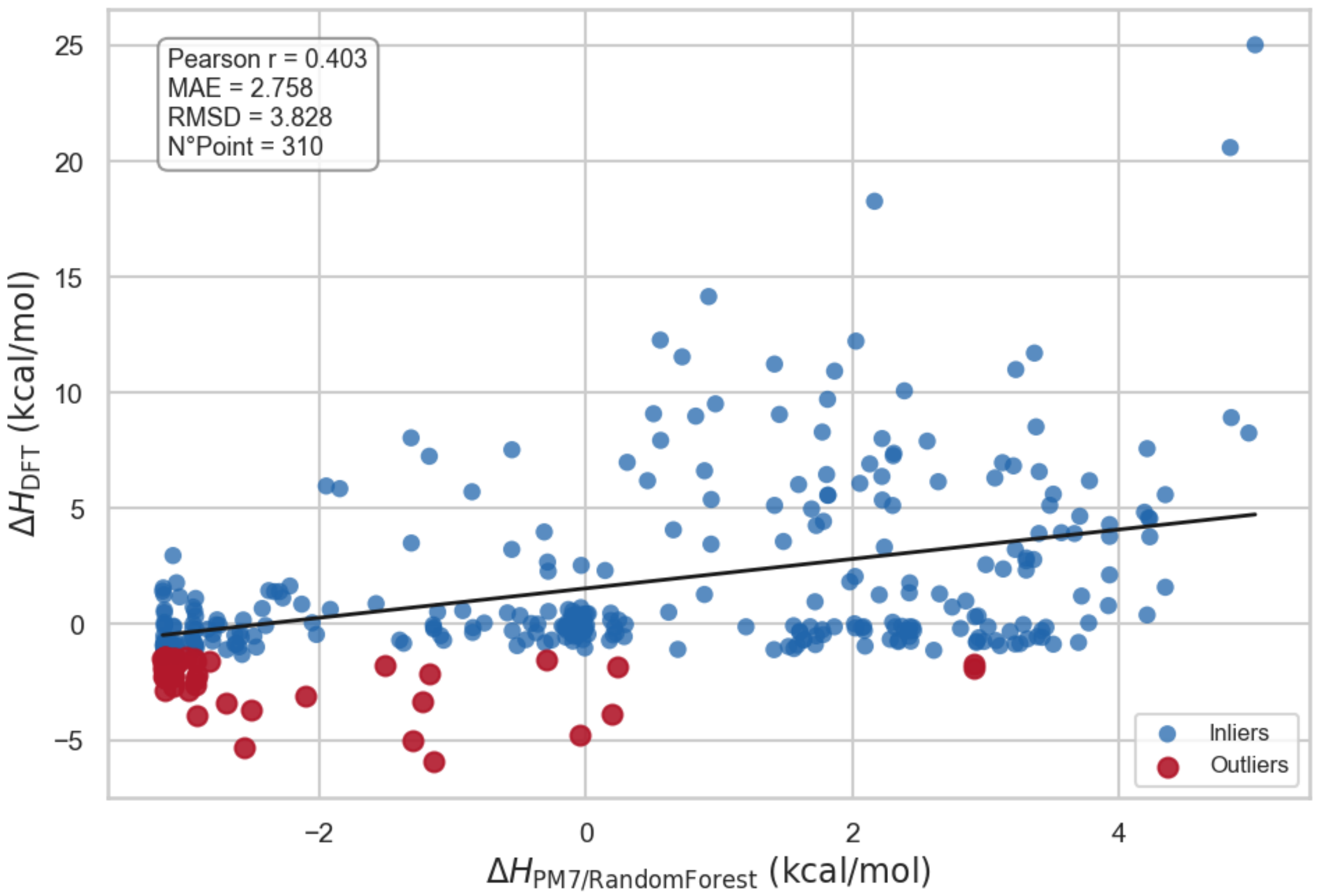

Figure S170: Scatter plots between reference DFT values and the values generated by the Random Forest model trained with PM7-derived features, applying One-Class SVM (SGD) outlier treatment with a contamination level of 0.20.

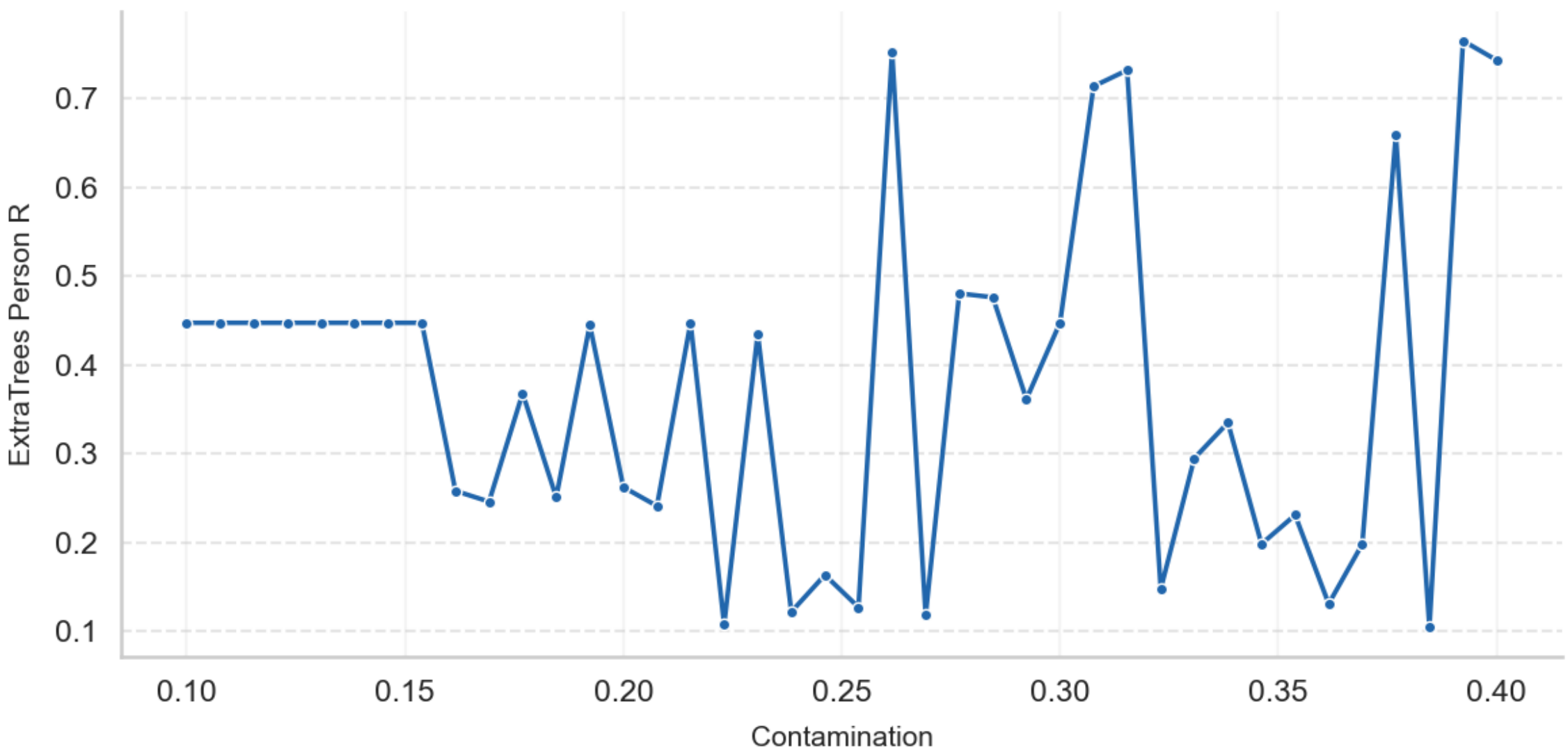

Figure S171: Correlation plot obtained between the data generated by the Extra Trees Regressor model trained with PM7-derived features and the reference values as a function of data contamination, using One-Class SVM (SGD)for outlier treatment.

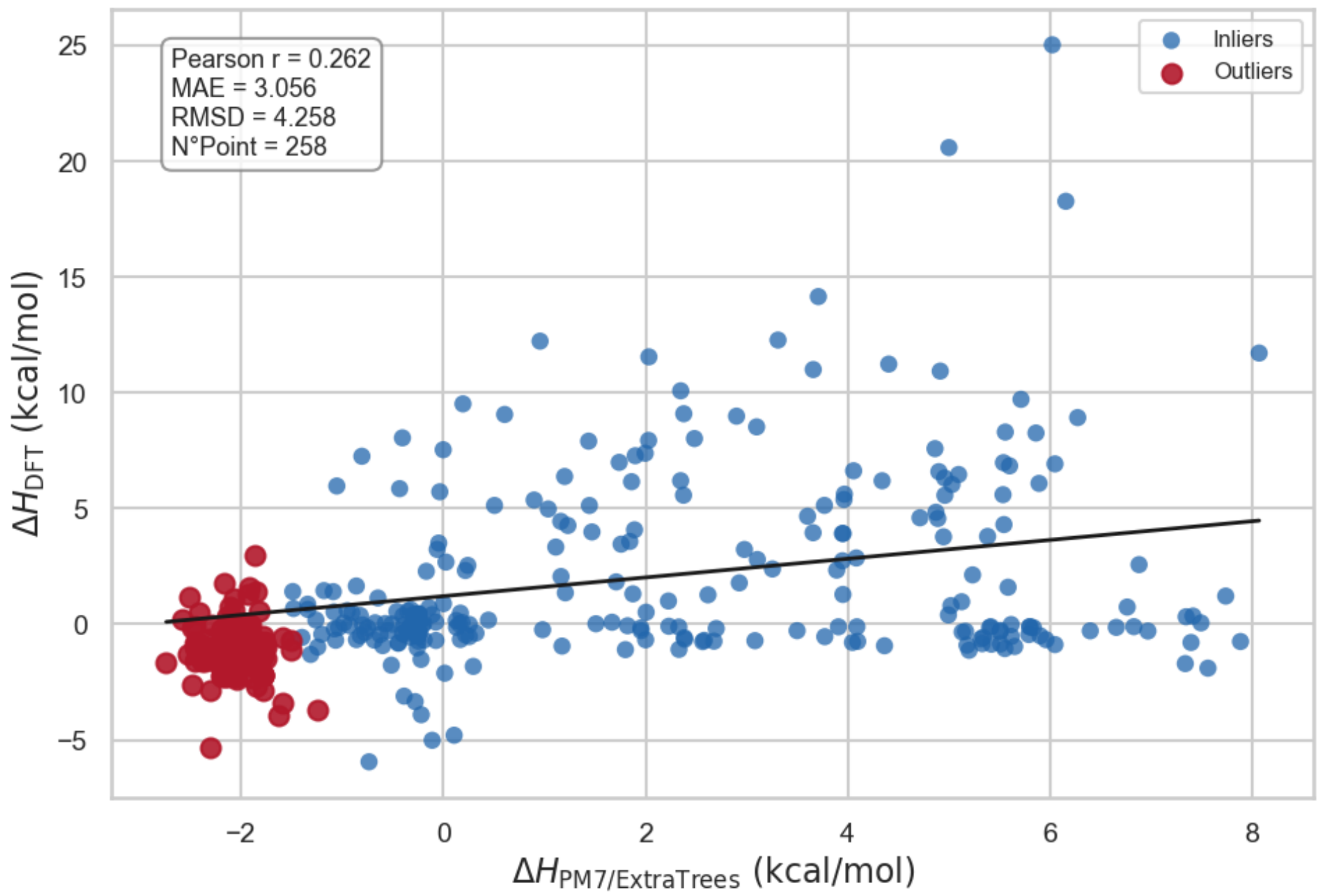

Figure S172: Scatter plots between reference DFT values and the values generated by the Extra Trees Regressor model trained with PM7-derived features, applying One-Class SVM (SGD) outlier treatment with a contamination level of 0.20.

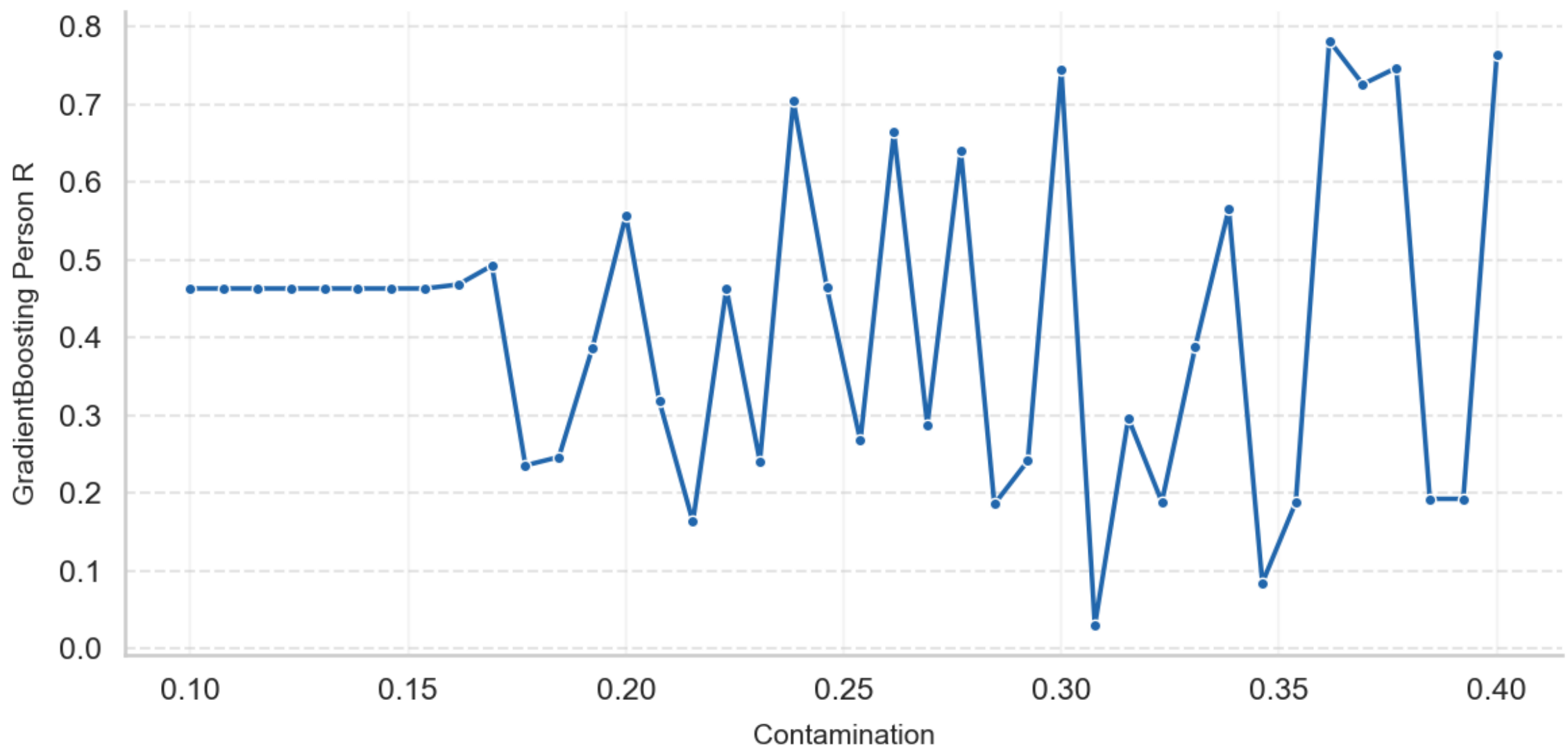

Figure S173: Correlation plot obtained between the data generated by the Gradient Boosting Regressor model trained with PM7-derived features and the reference values as a function of data contamination, using One-Class SVM (SGD)for outlier treatment.

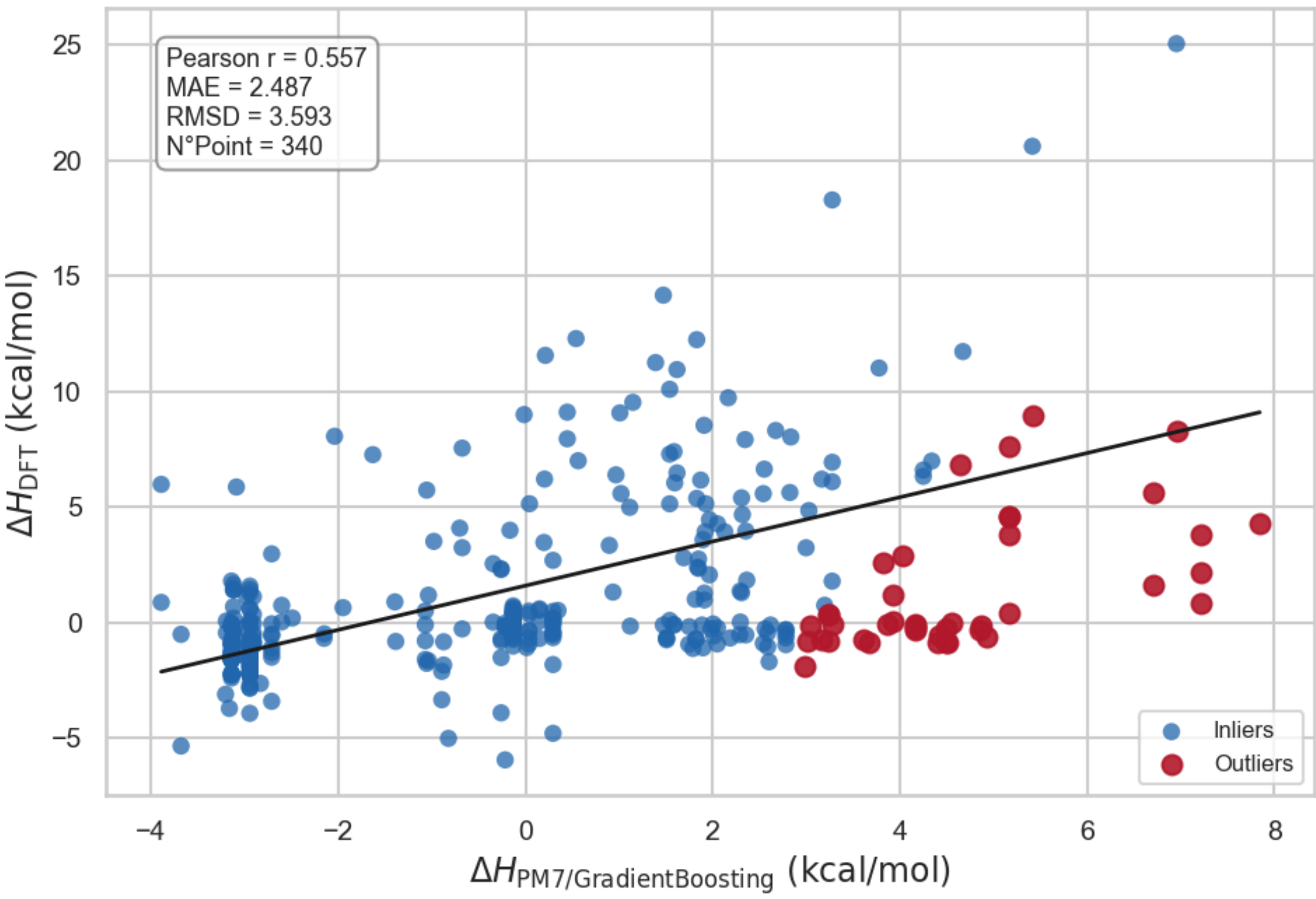

Figure S174: Scatter plots between reference DFT values and the values generated by the Boosting Regressor model trained with PM7-derived features, applying One-Class SVM (SGD) outlier treatment with a contamination level of 0.20.

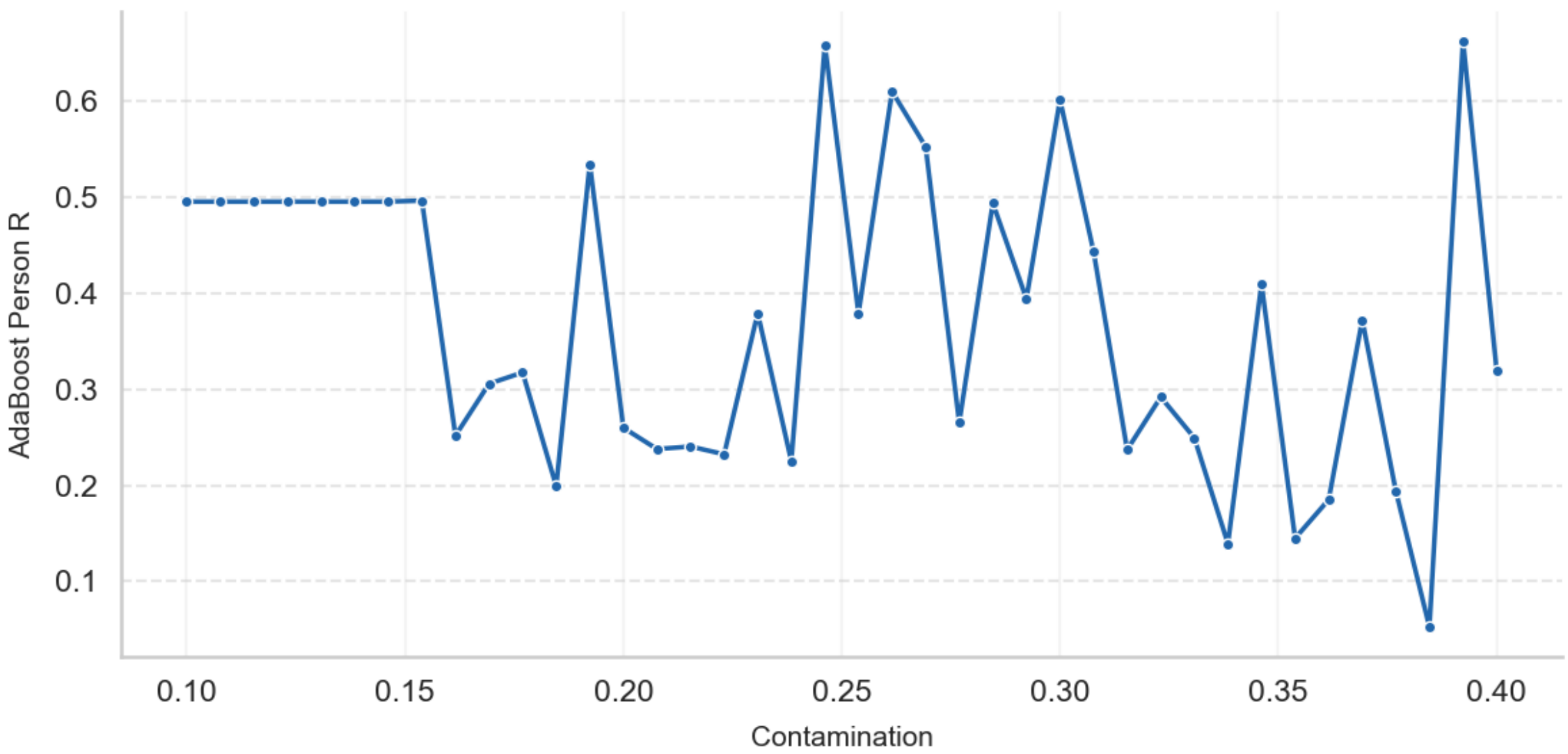

Figure S175: Correlation plot obtained between the data generated by the Ada Boost Regressor model trained with PM7-derived features and the reference values as a function of data contamination, using One-Class SVM (SGD)for outlier treatment.

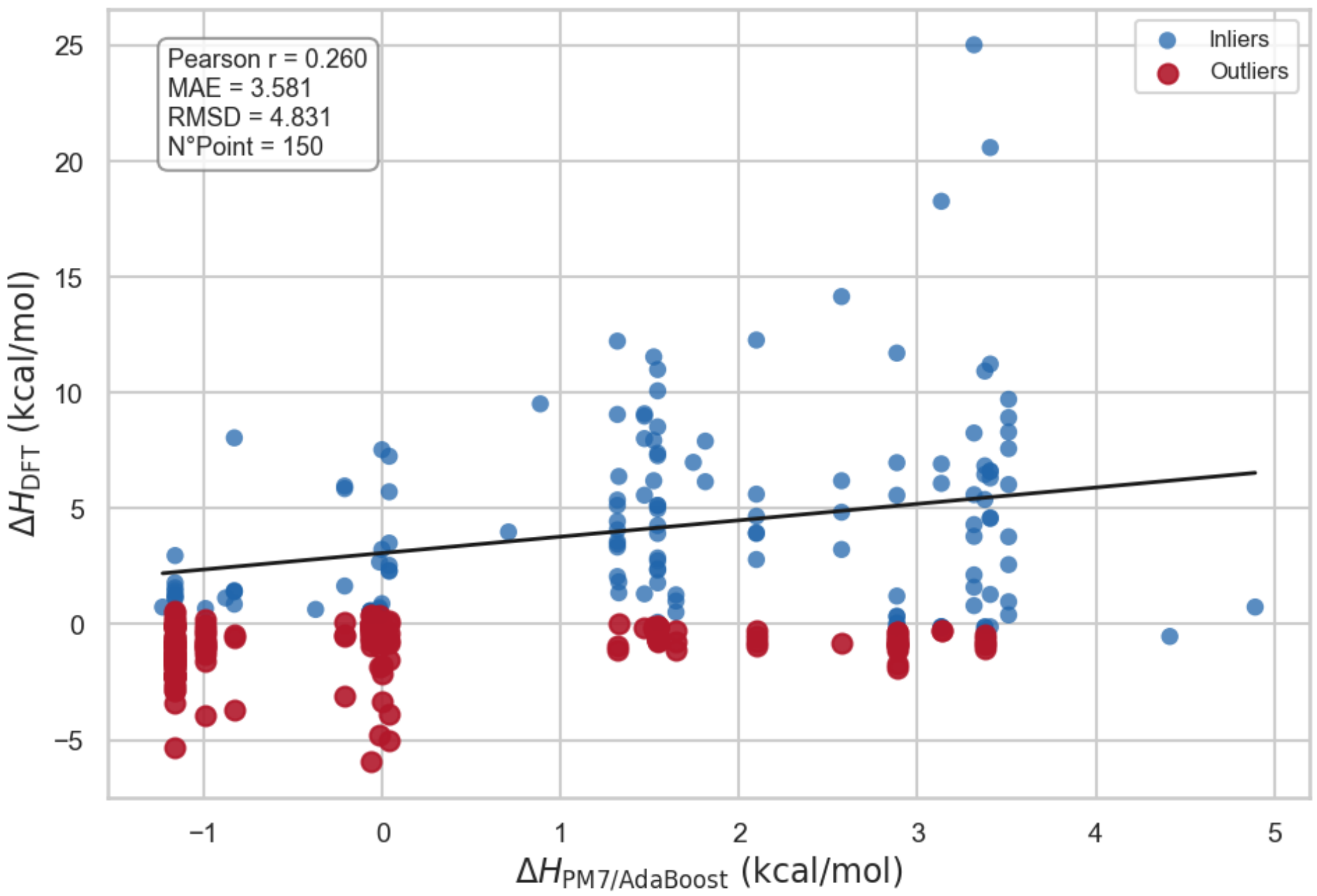

Figure S176: Scatter plots between reference DFT values and the values generated by the Ada Boost Regressor model trained with PM7-derived features, applying One-Class SVM (SGD) outlier treatment with a contamination level of 0.20.

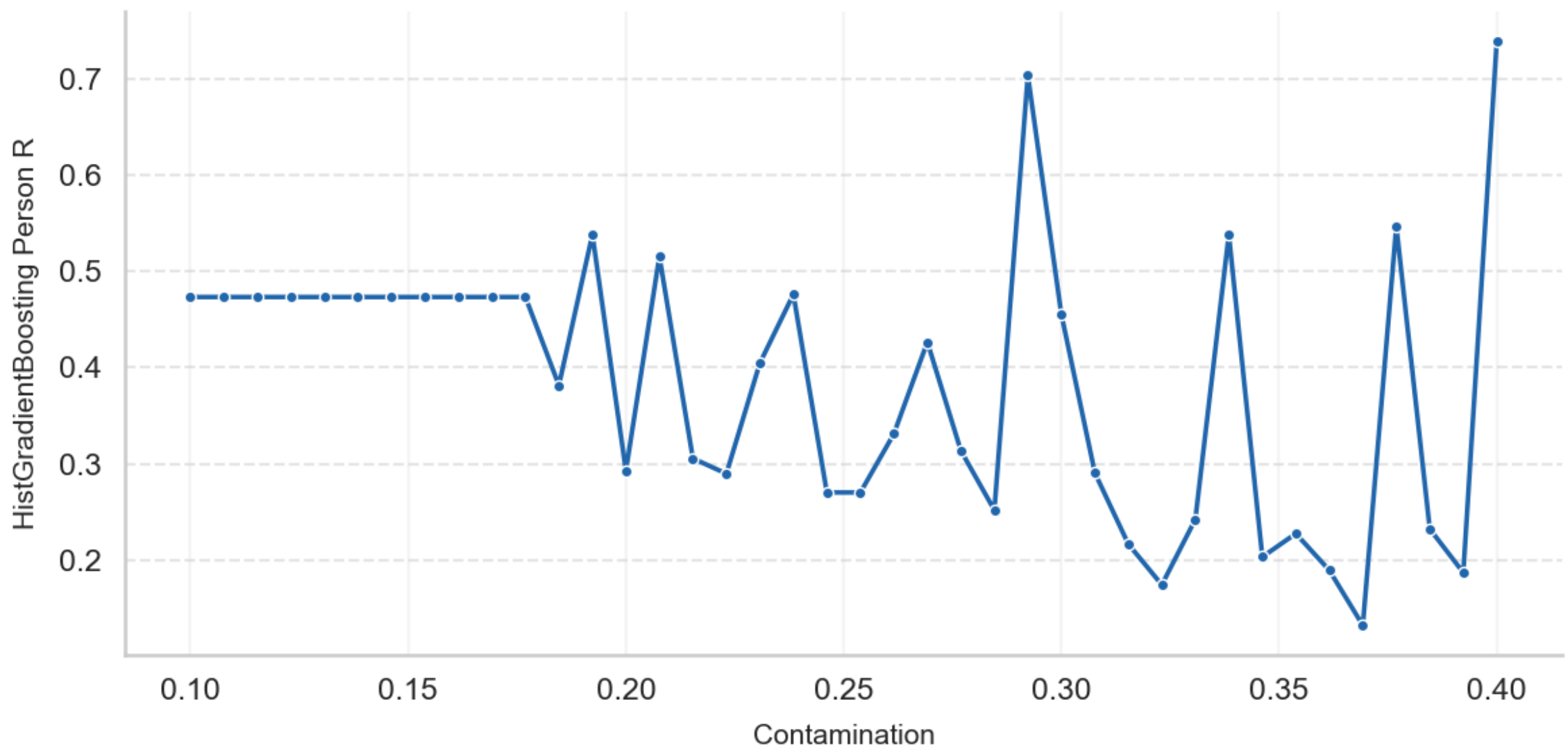

Figure S177: Correlation plot obtained between the data generated by the Hist Gradient Boosting model trained with PM7-derived features and the reference values as a function of data contamination, using One-Class SVM (SGD)for outlier treatment.

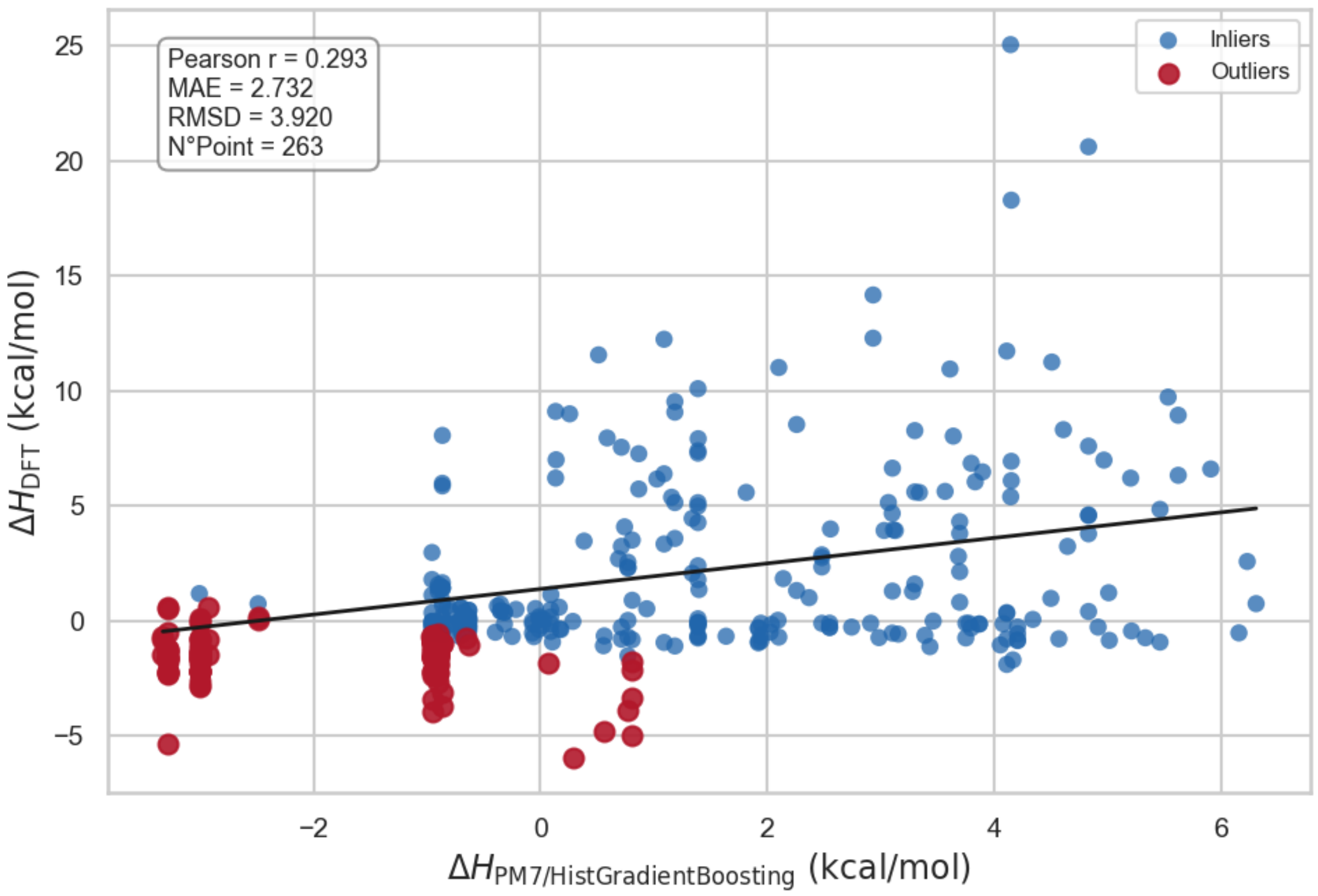

Figure S178: Scatter plots between reference DFT values and the values generated by the Hist Gradient Boosting model trained with PM7-derived features, applying One-Class SVM (SGD) outlier treatment with a contamination level of 0.20.

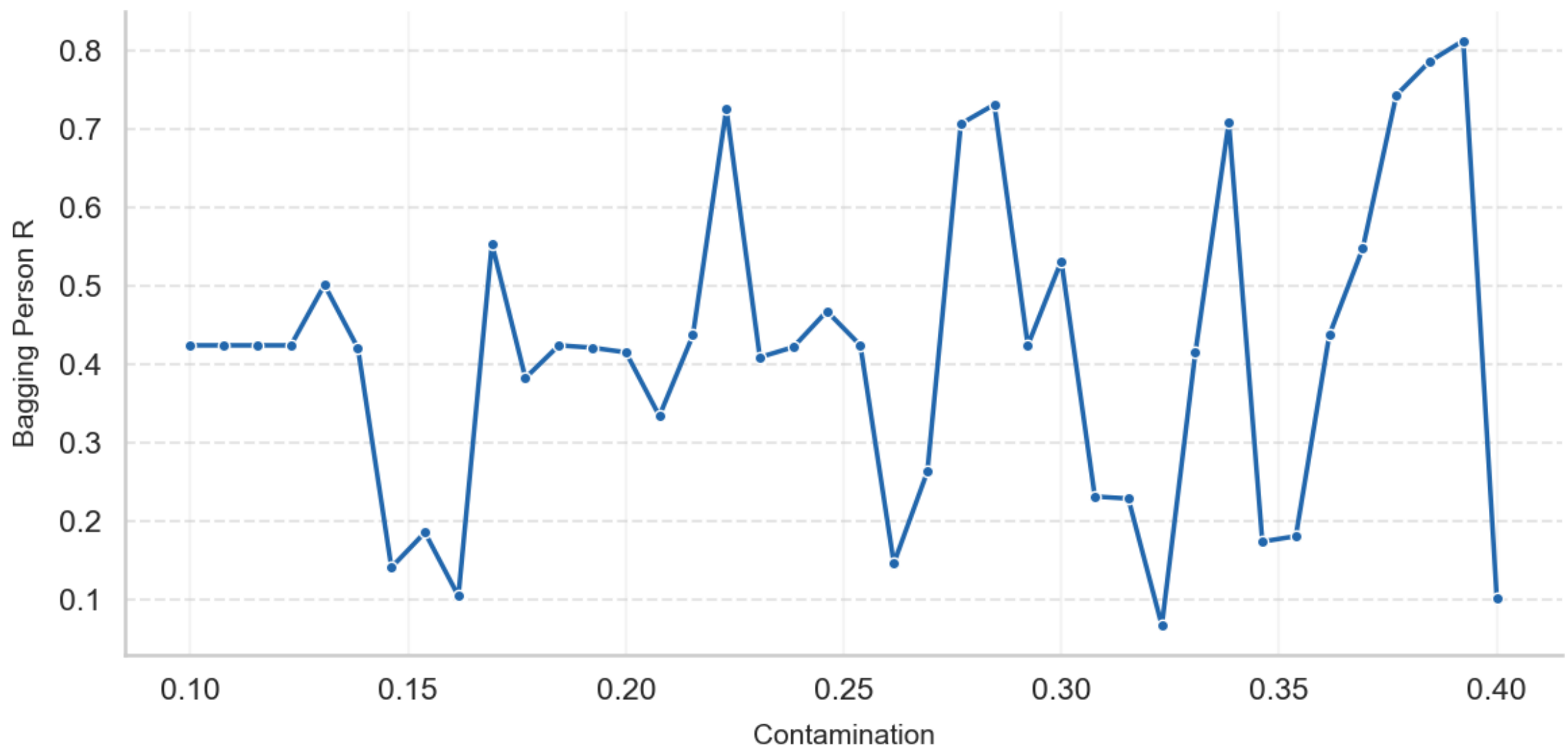

Figure S179: Correlation plot obtained between the data generated by the Bagging Regressor model trained with PM7-derived features and the reference values as a function of data contamination, using One-Class SVM (SGD)for outlier treatment.

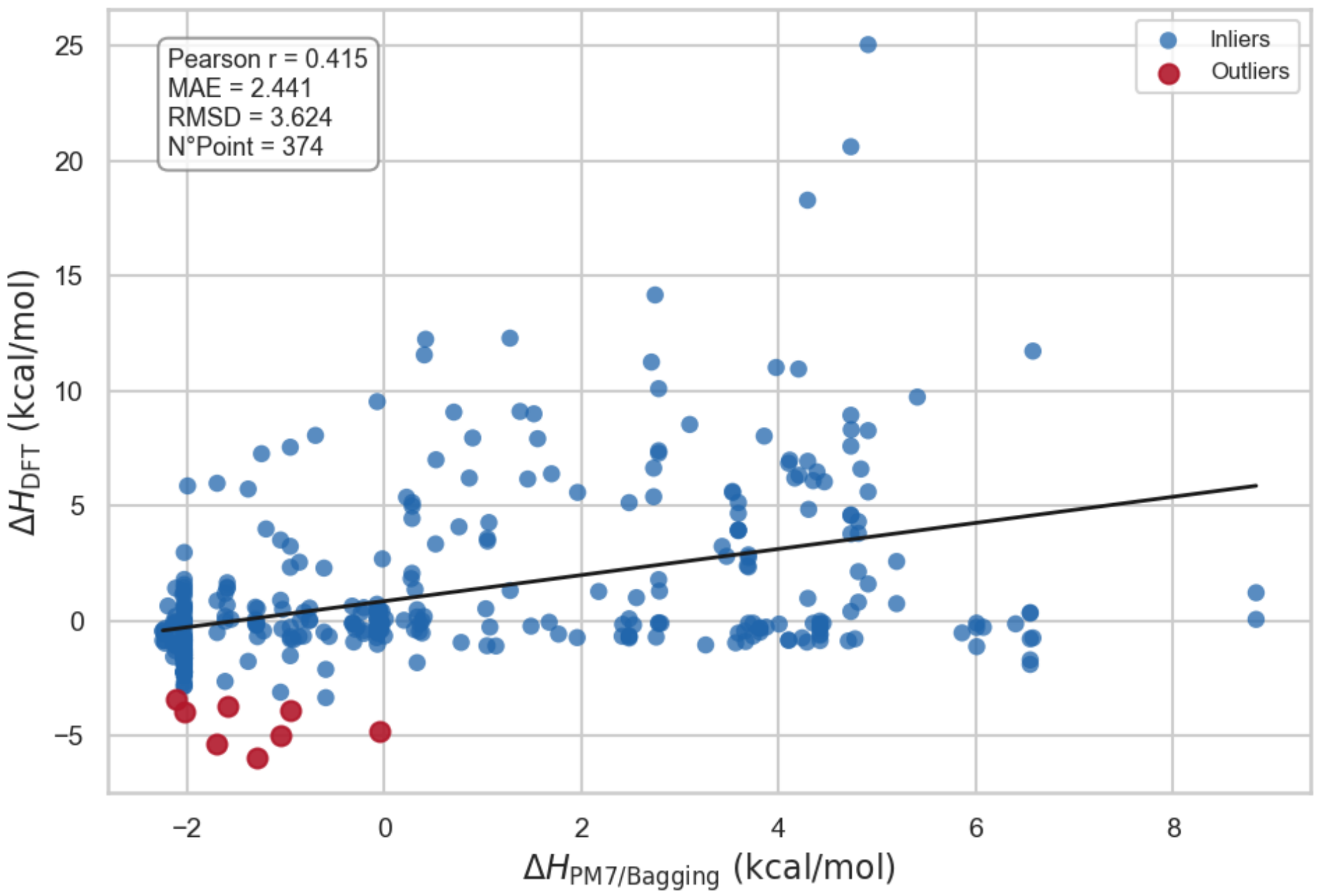

Figure S180: Scatter plots between reference DFT values and the values generated by the Bagging Regressor model trained with PM7-derived features, applying One-Class SVM (SGD) outlier treatment with a contamination level of 0.20.

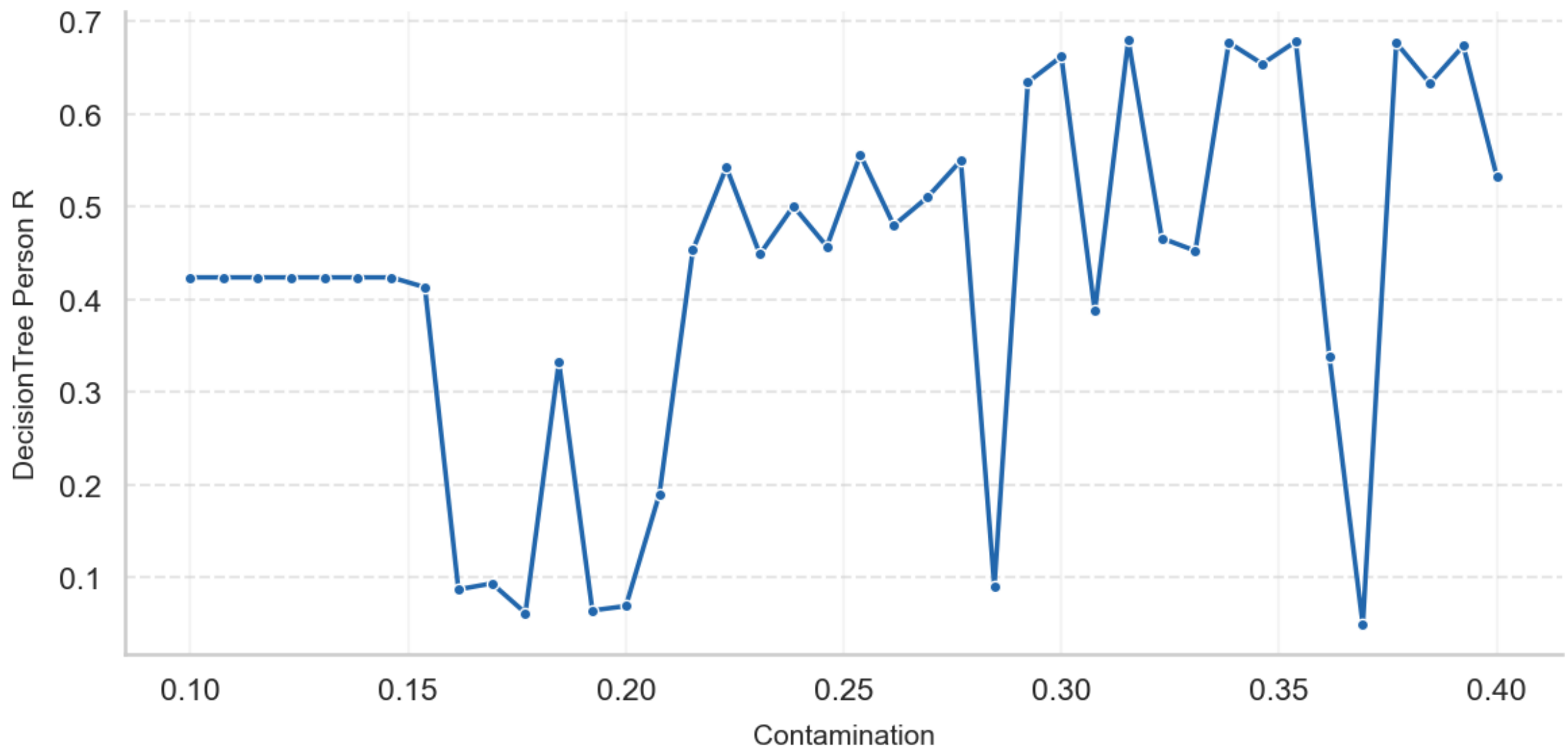

Figure S181: Correlation plot obtained between the data generated by the Decision Tree Regressor model trained with PM7-derived features and the reference values as a function of data contamination, using One-Class SVM (SGD)for outlier treatment.

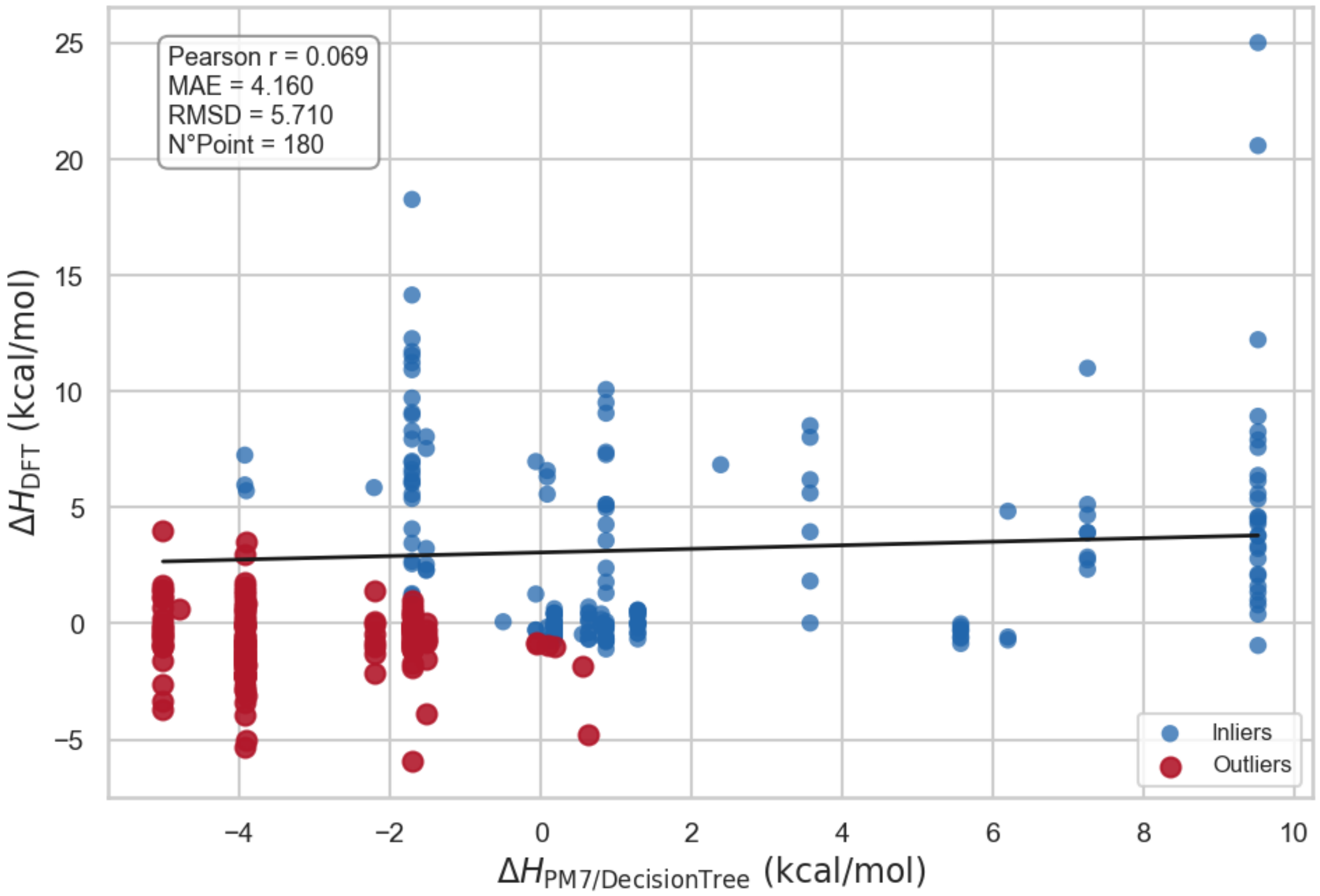

Figure S182: Scatter plots between reference DFT values and the values generated by the the Decision Tree Regressor model trained with PM7-derived features, applying One-Class SVM (SGD)outlier treatment with a contamination level of 0.20.

S6.3 Robust Covariance

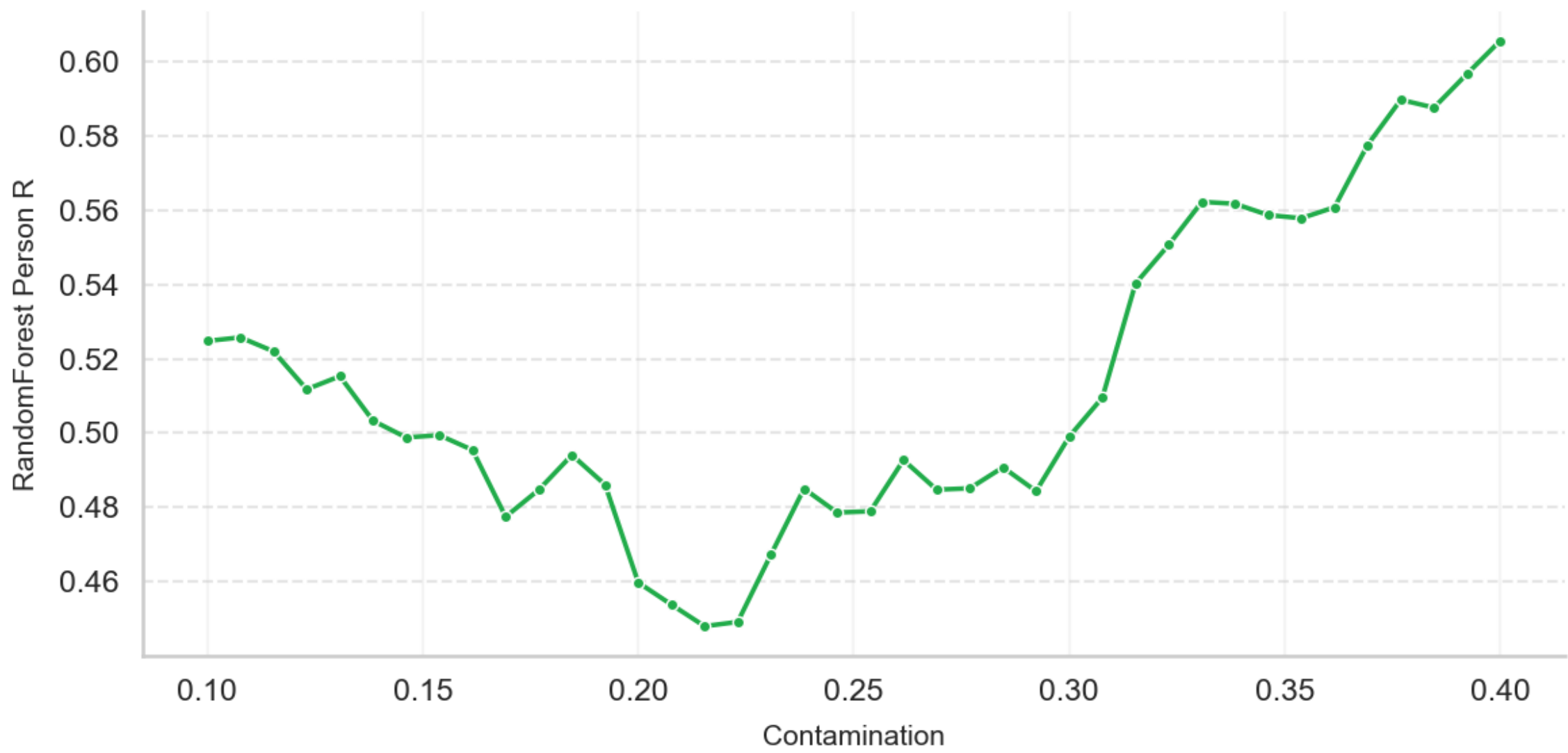

Figure S183: Correlation plot obtained between the data generated by the Random Forest model trained with PM7-derived features and the reference values as a function of data contamination, using Robust Covariance for outlier treatment.

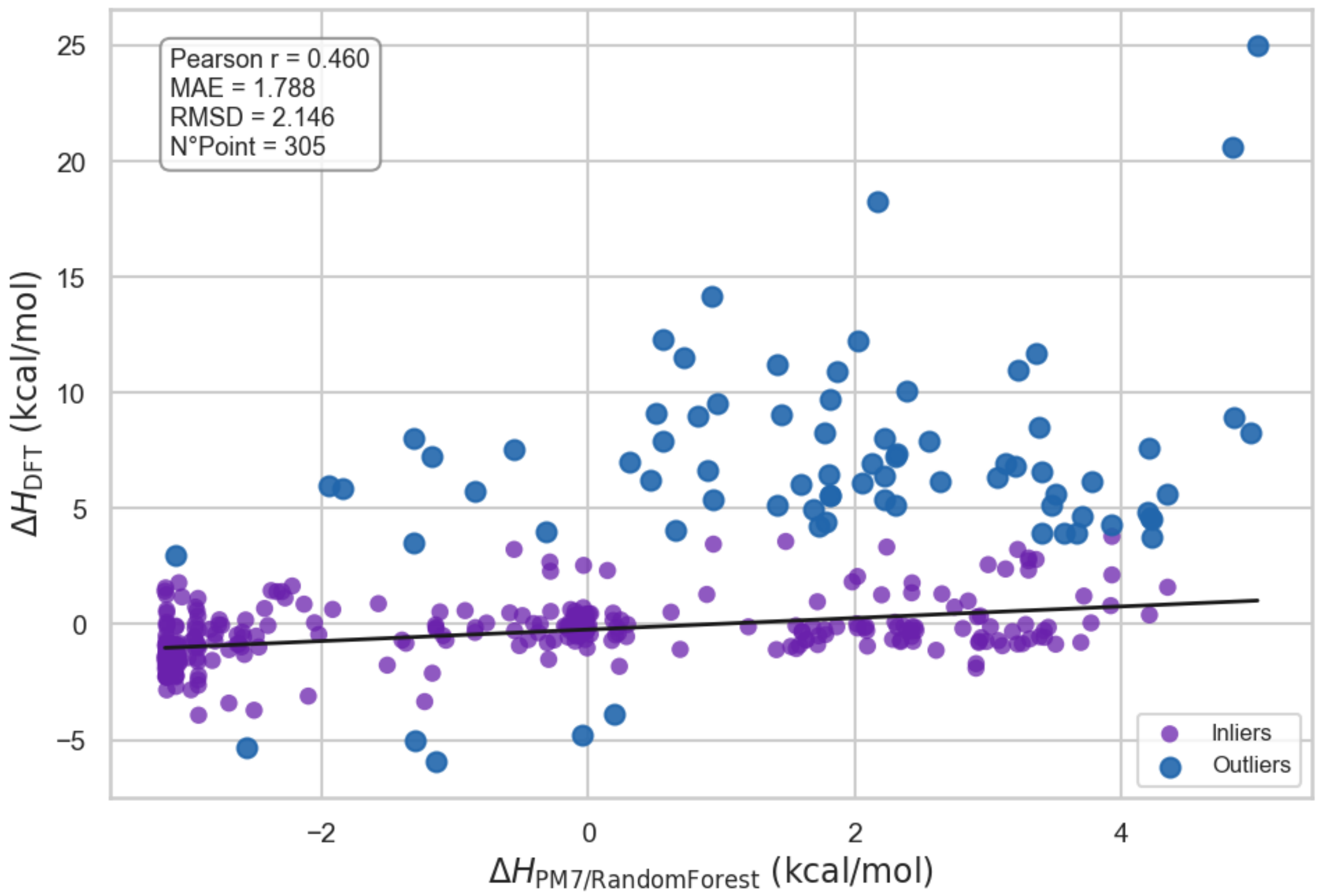

Figure S184: Scatter plots between reference DFT values and the values generated by the Random Forest model trained with PM7-derived features, applying Robust Covariance outlier treatment with a contamination level of 0.20.

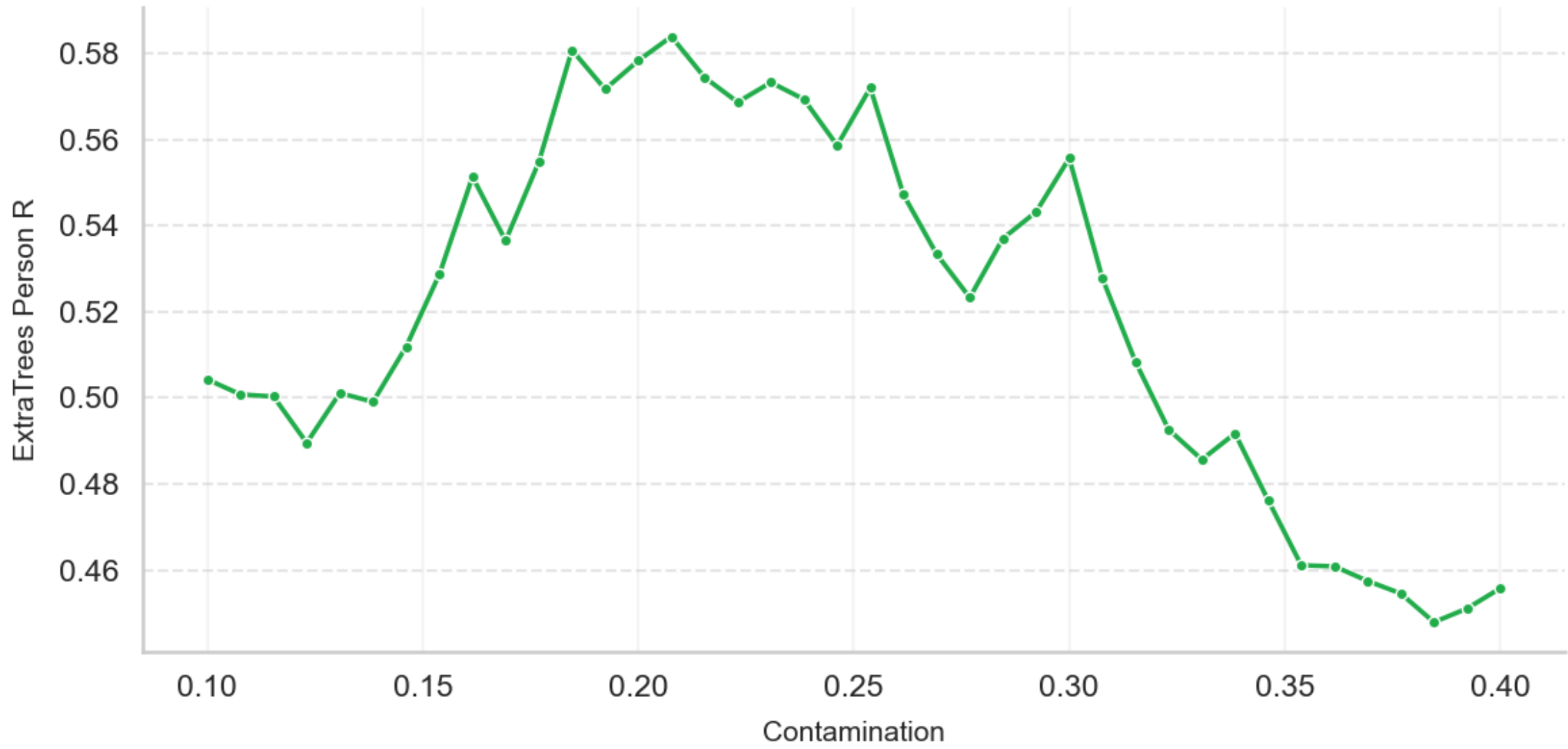

Figure S185: Correlation plot obtained between the data generated by the Extra Trees Regressor model trained with PM7-derived features and the reference values as a function of data contamination, using Robust Covariance for outlier treatment.

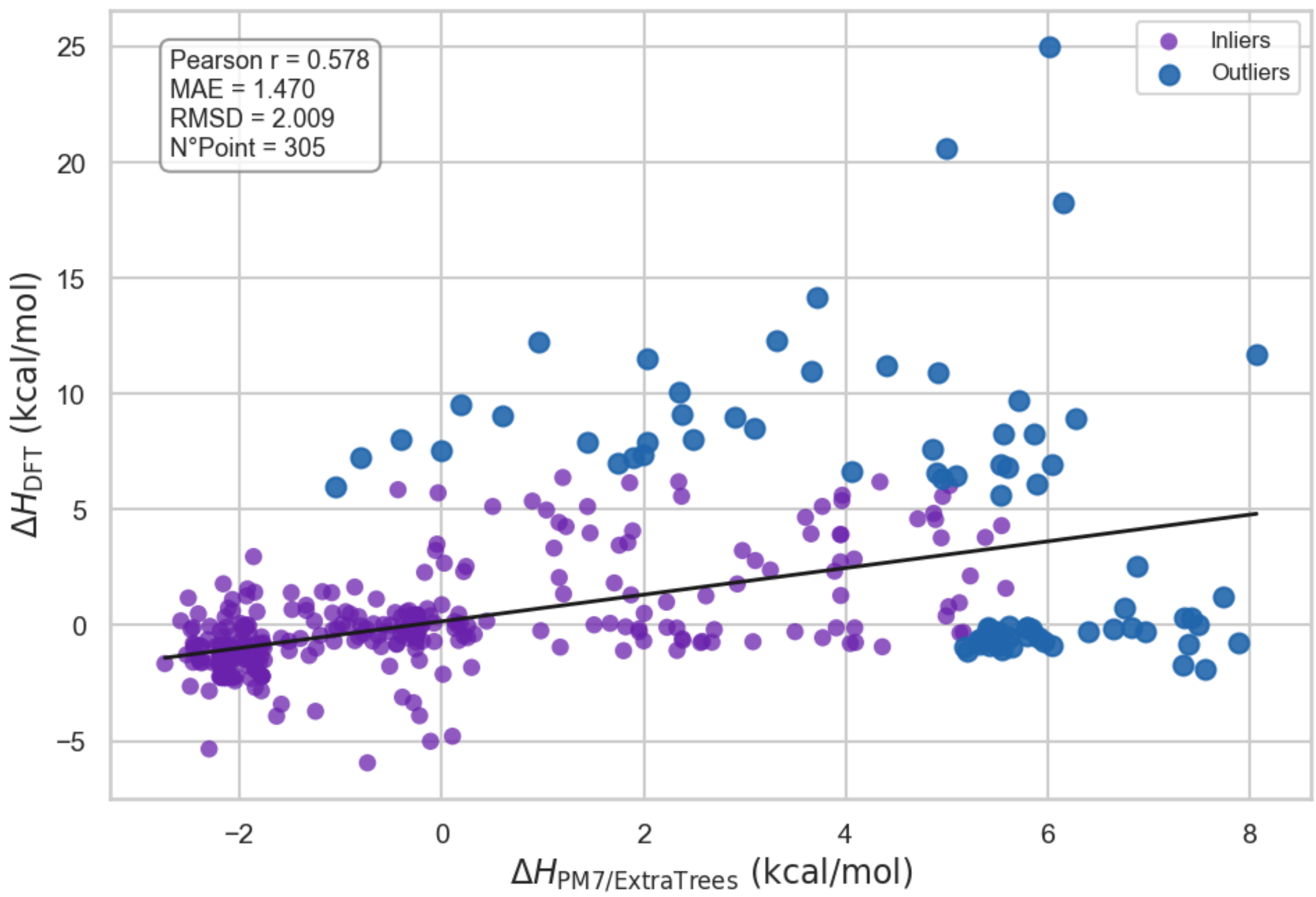

Figure S186: Scatter plots between reference DFT values and the values generated by the Extra Trees Regressor model trained with PM7-derived features, applying Robust Covariance outlier treatment with a contamination level of 0.20.

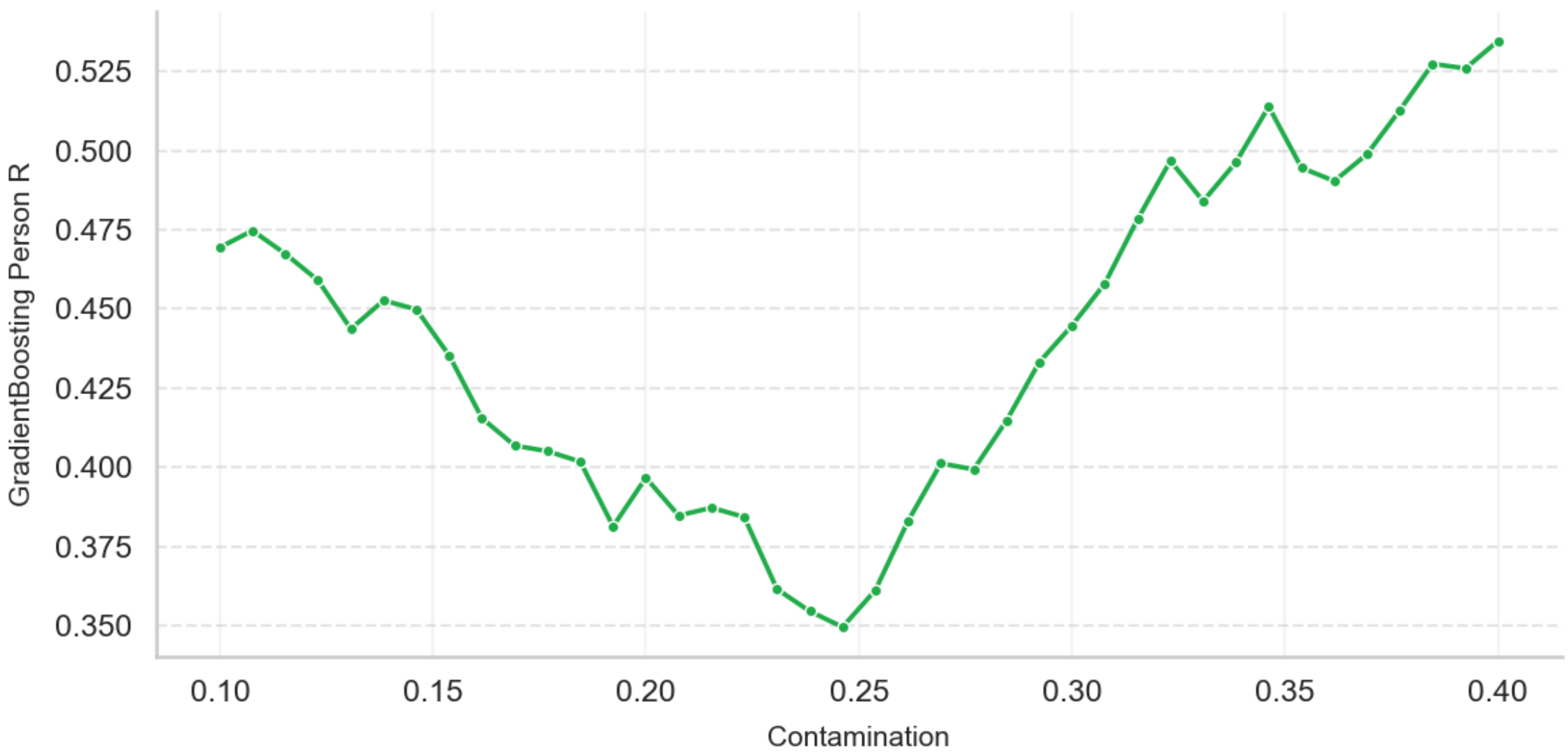

Figure S187: Correlation plot obtained between the data generated by the Gradient Boosting Regressor model trained with PM7-derived features and the reference values as a function of data contamination, using Robust Covariance for outlier treatment.

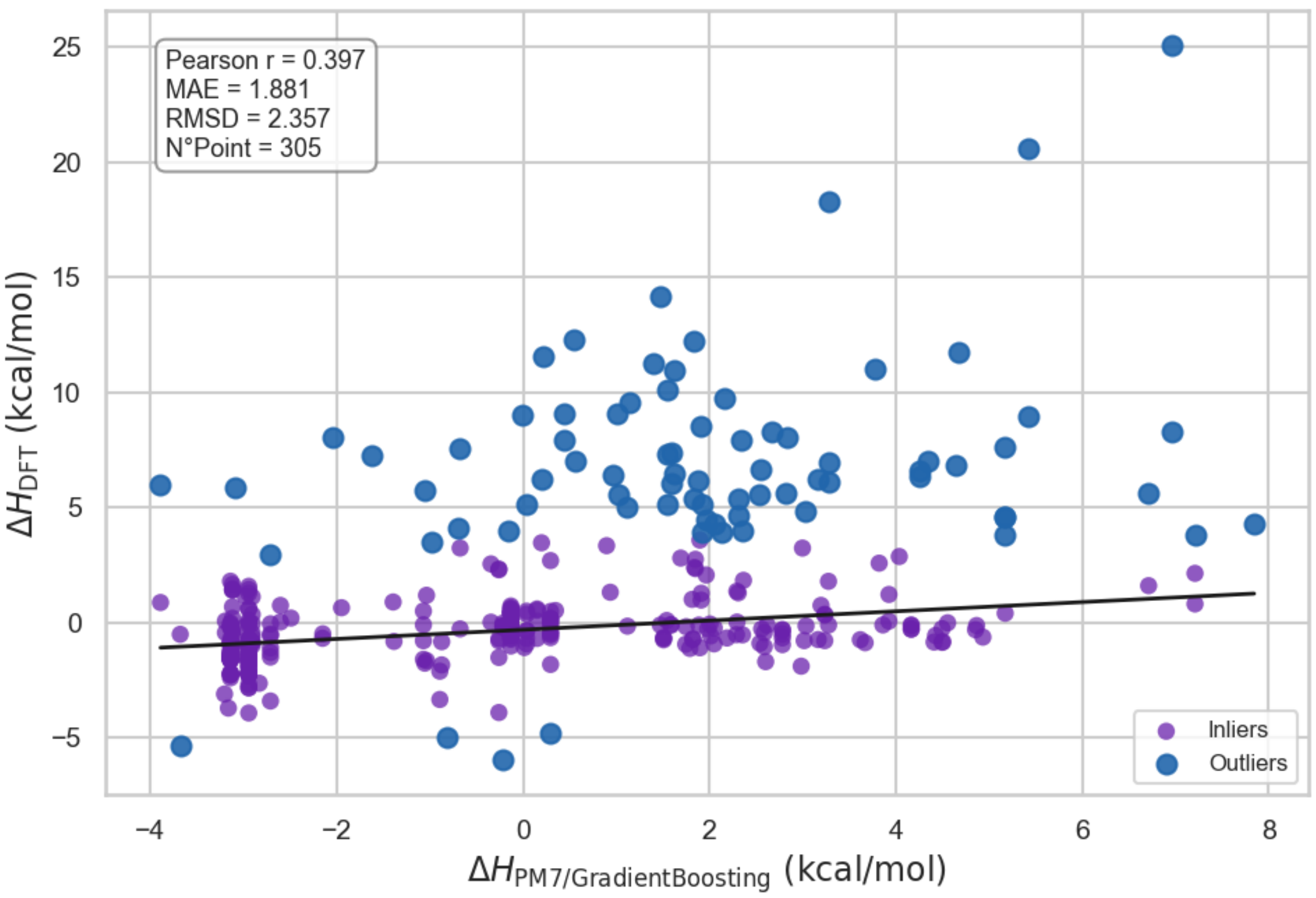

Figure S188: Scatter plots between reference DFT values and the values generated by the Boosting Regressor model trained with PM7-derived features, applying Robust Covariance outlier treatment with a contamination level of 0.20.

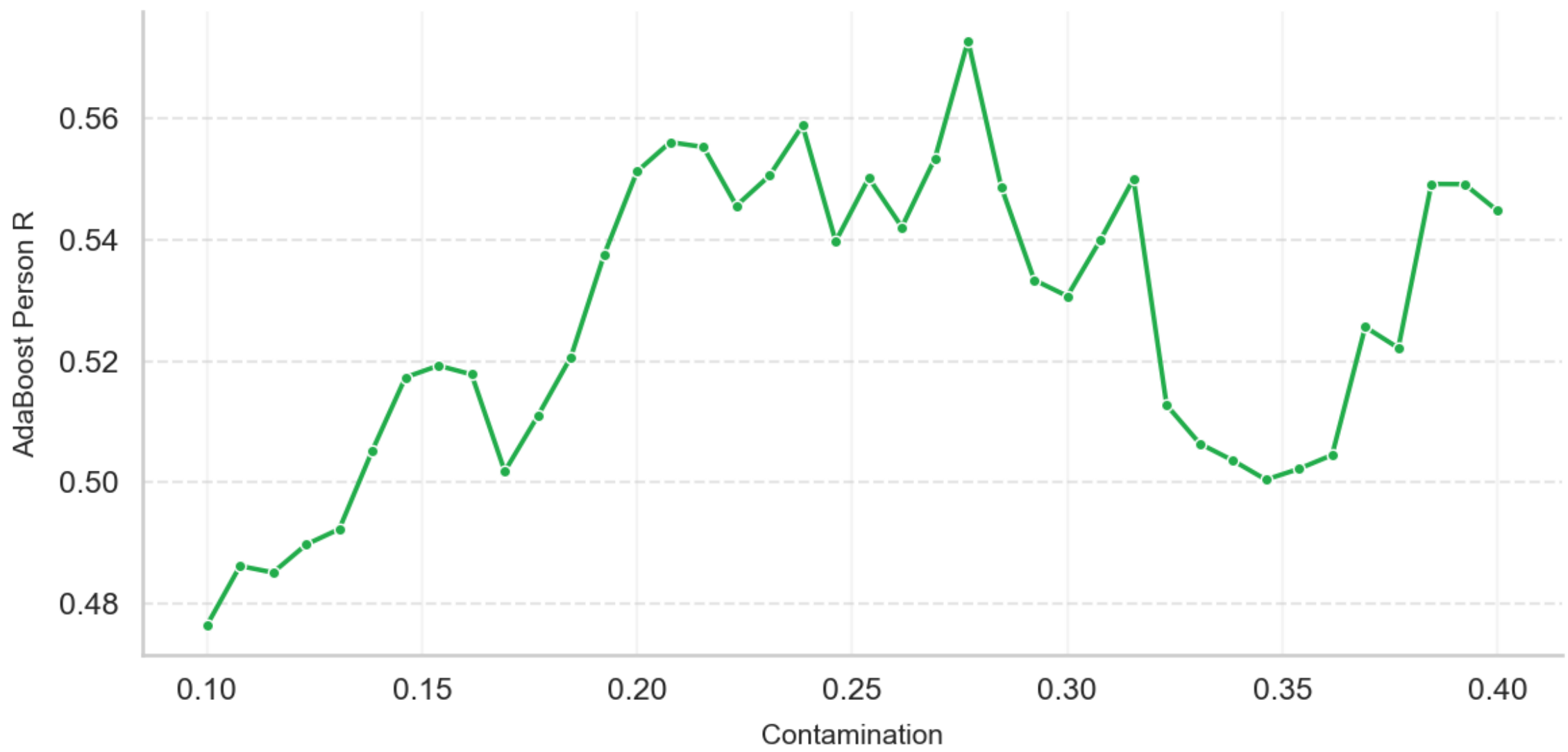

Figure S189: Correlation plot obtained between the data generated by the Ada Boost Regressor model trained with PM7-derived features and the reference values as a function of data contamination, using Robust Covariance for outlier treatment.

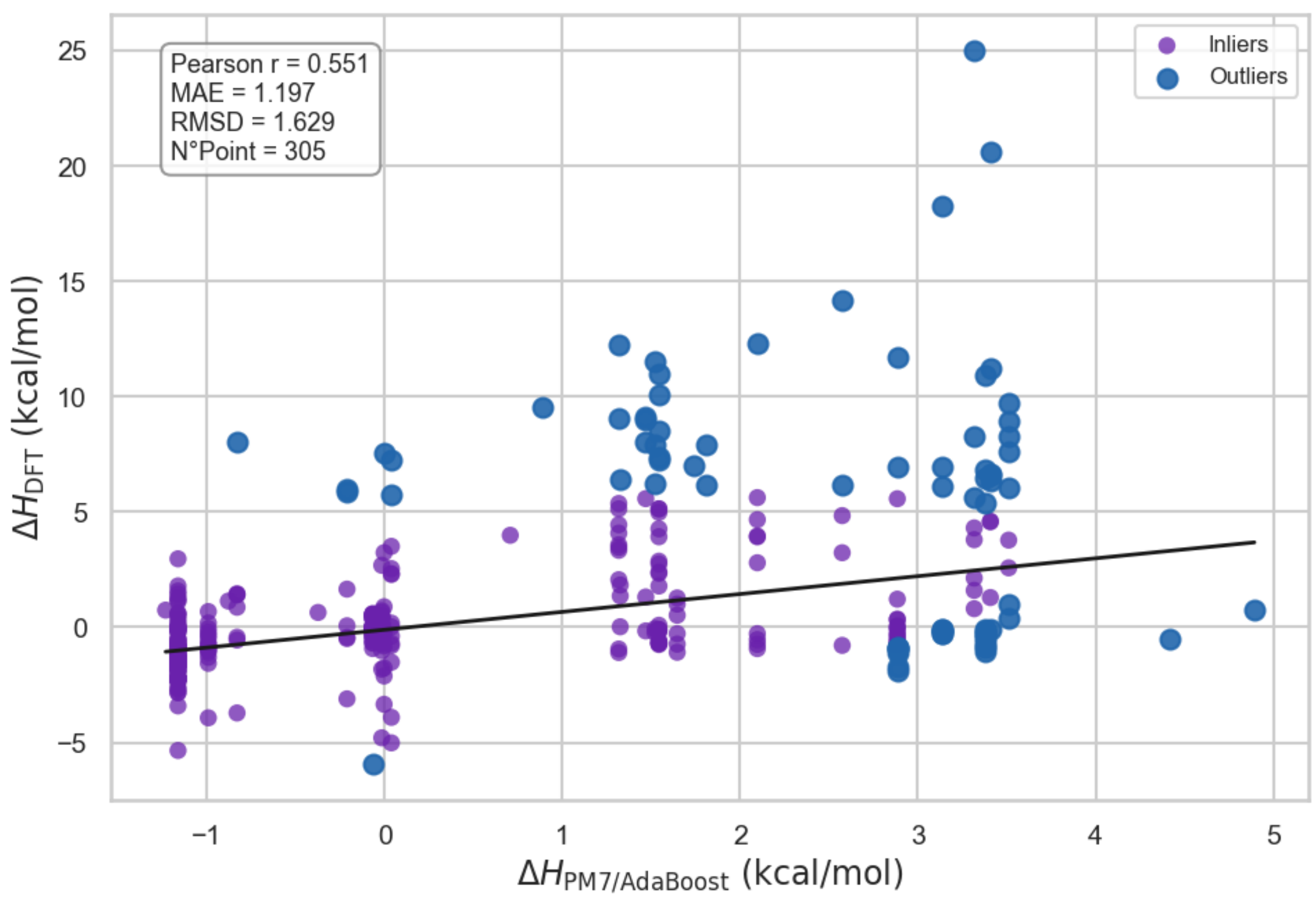

Figure S190: Scatter plots between reference DFT values and the values generated by the Ada Boost Regressor model trained with PM7-derived features, applying Robust Covariance outlier treatment with a contamination level of 0.20.

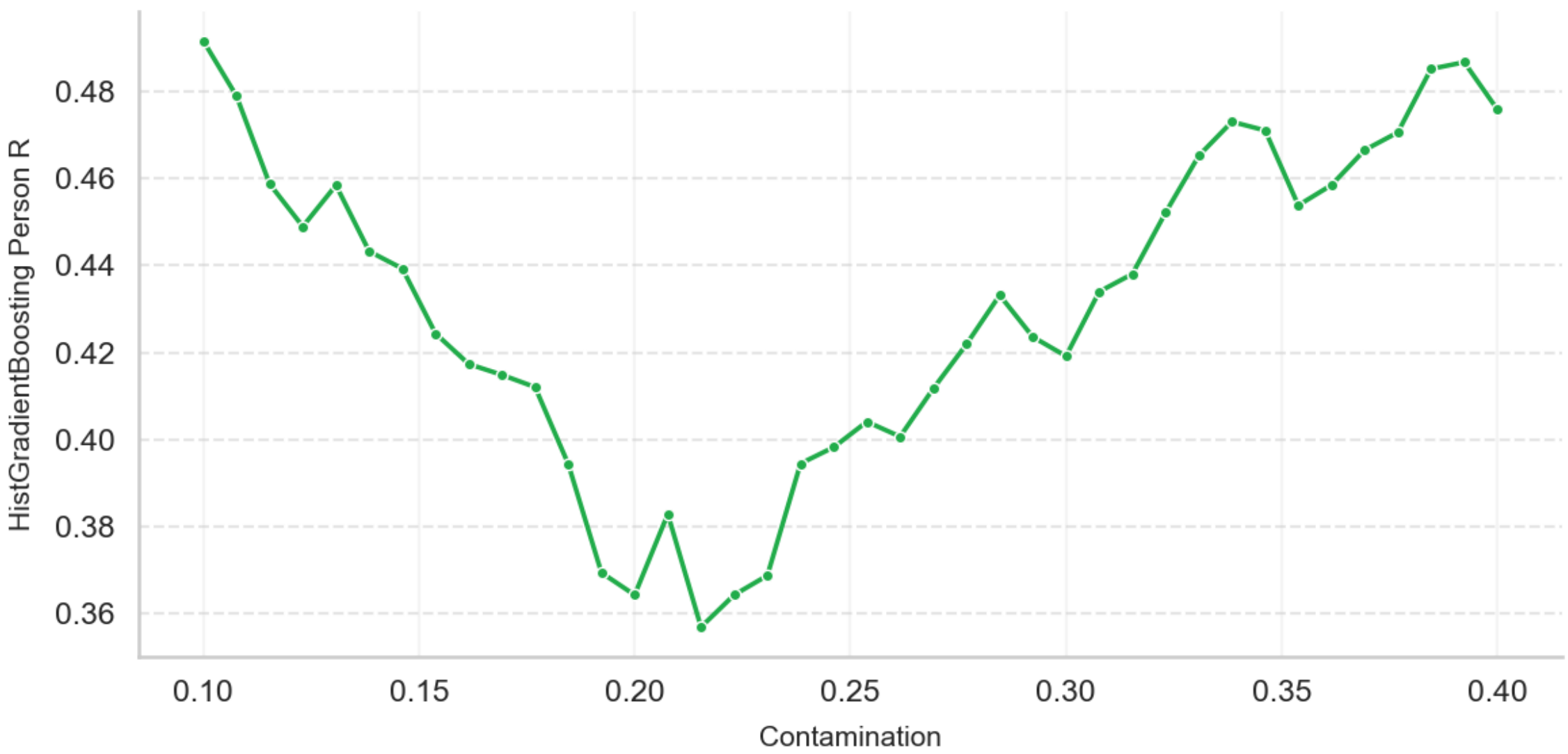

Figure S191: Correlation plot obtained between the data generated by the Hist Gradient Boosting model trained with PM7-derived features and the reference values as a function of data contamination, using Robust Covariance for outlier treatment.

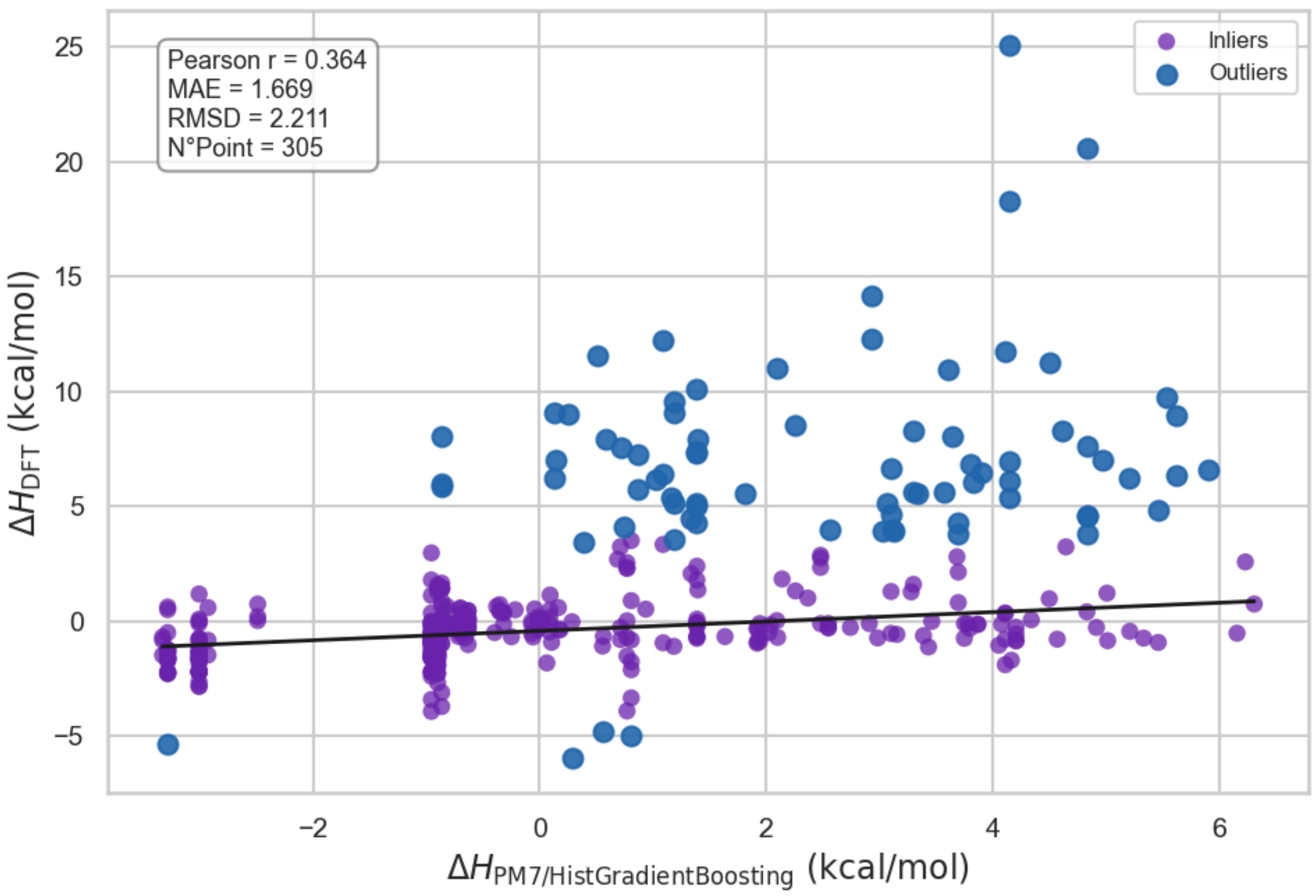

Figure S192: Scatter plots between reference DFT values and the values generated by the Hist Gradient Boosting model trained with PM7-derived features, applying Robust Covariance outlier treatment with a contamination level of 0.20.

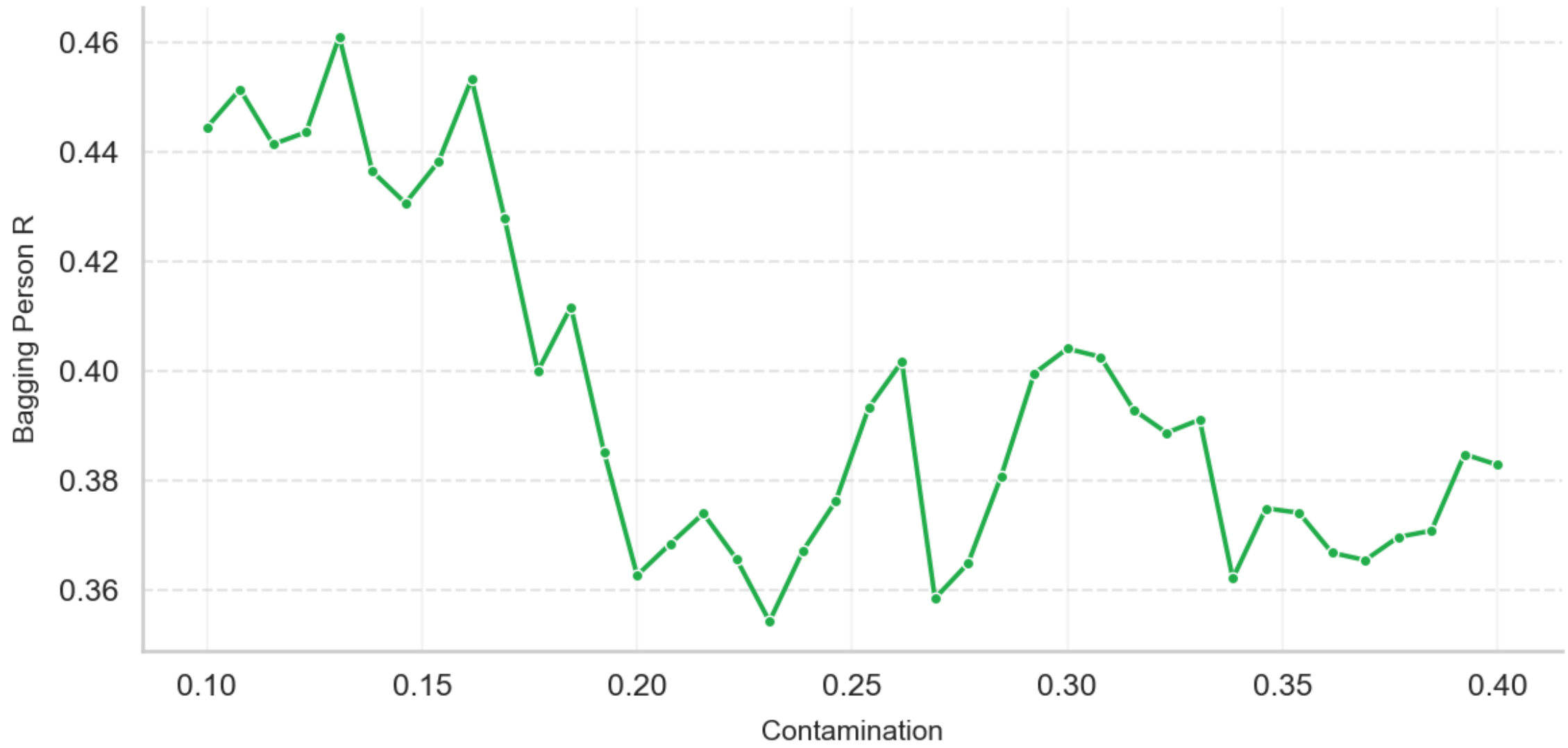

Figure S193: Correlation plot obtained between the data generated by the Bagging Regressor model trained with PM7-derived features and the reference values as a function of data contamination, using Robust Covariance for outlier treatment.

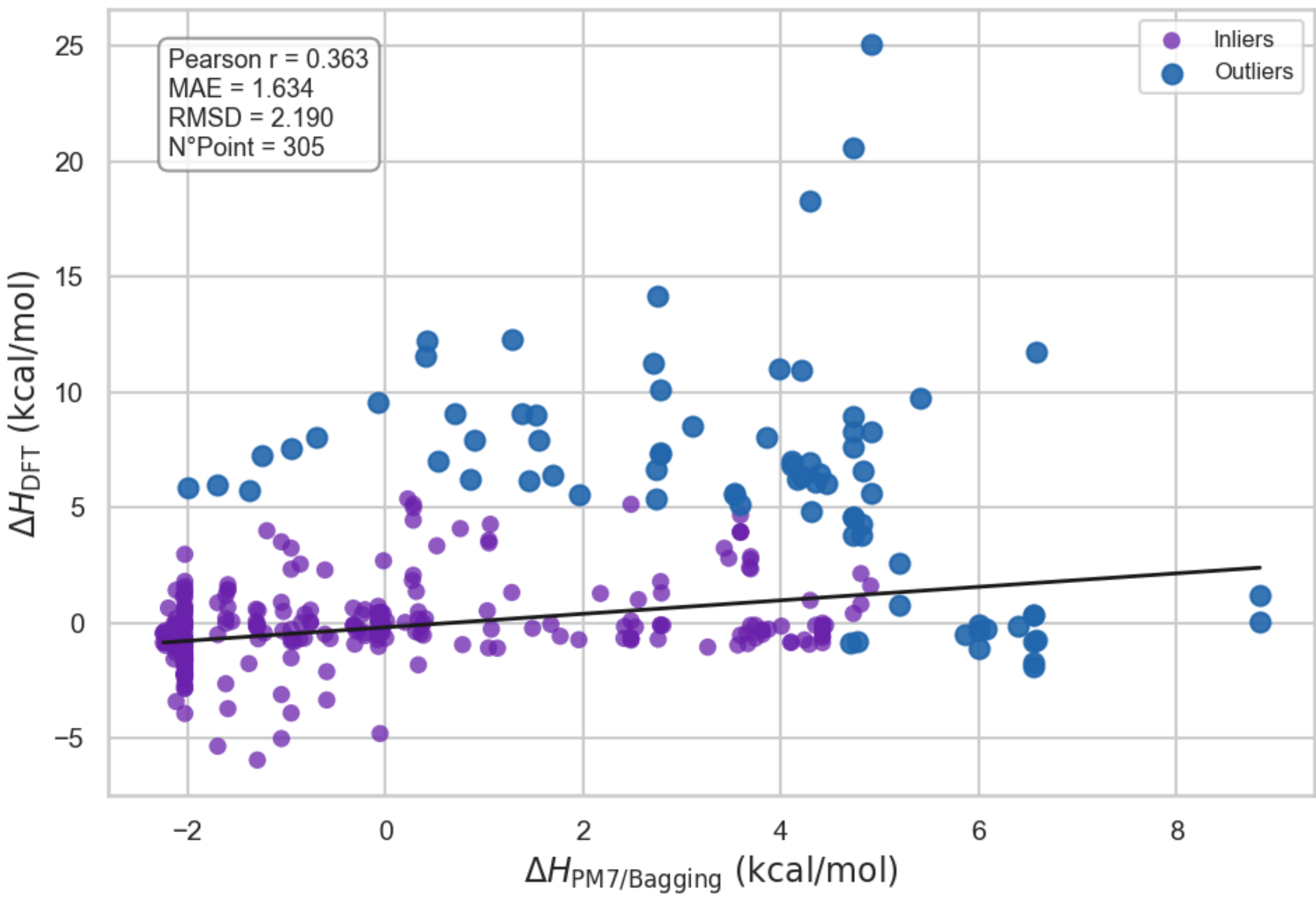

Figure S194: Scatter plots between reference DFT values and the values generated by the Bagging Regressor model trained with PM7-derived features, applying Robust Covariance with a contamination level of 0.20.

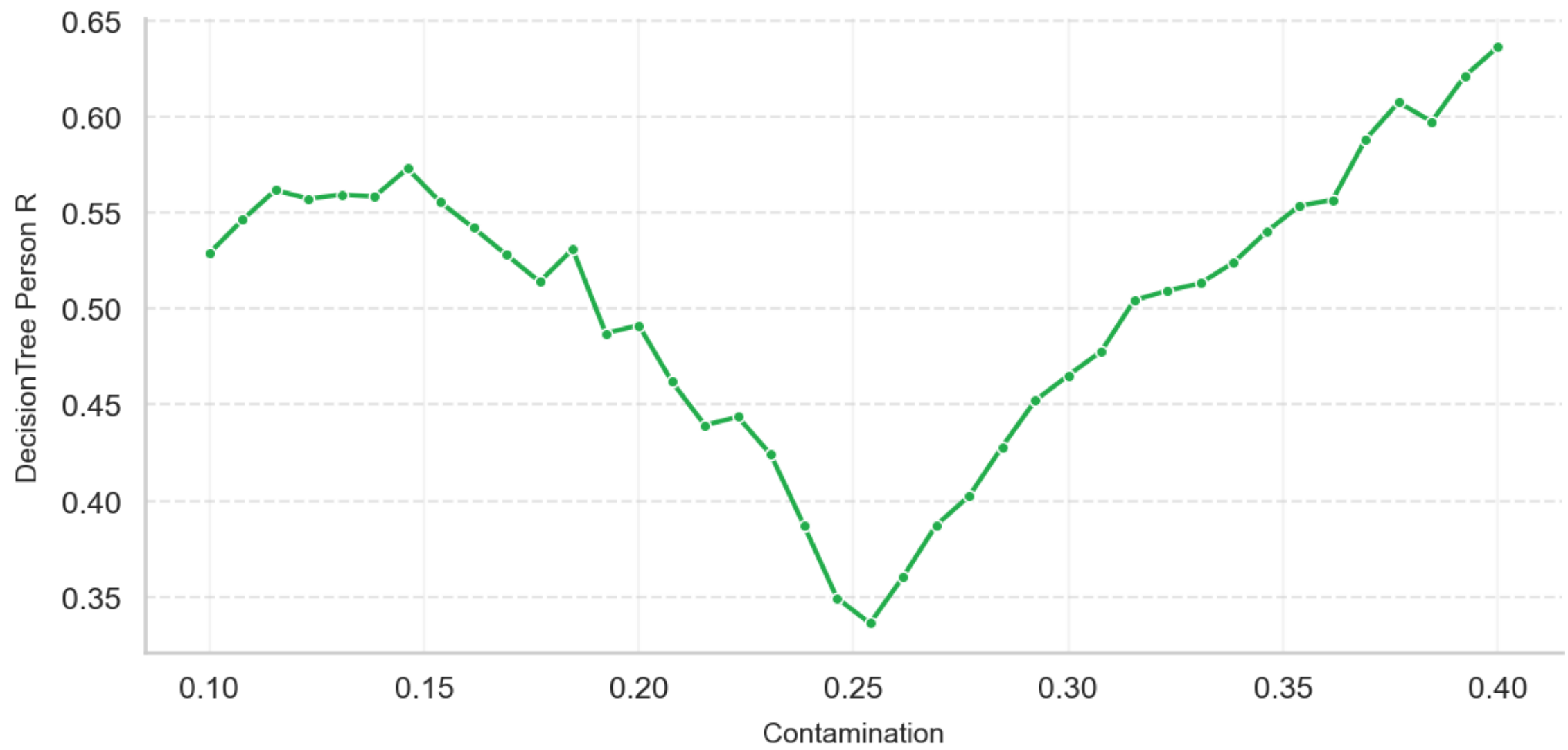

Figure S195: Correlation plot obtained between the data generated by the Decision Tree Regressor model trained with PM7-derived features and the reference values as a function of data contamination, using Robust Covariance for outlier treatment.

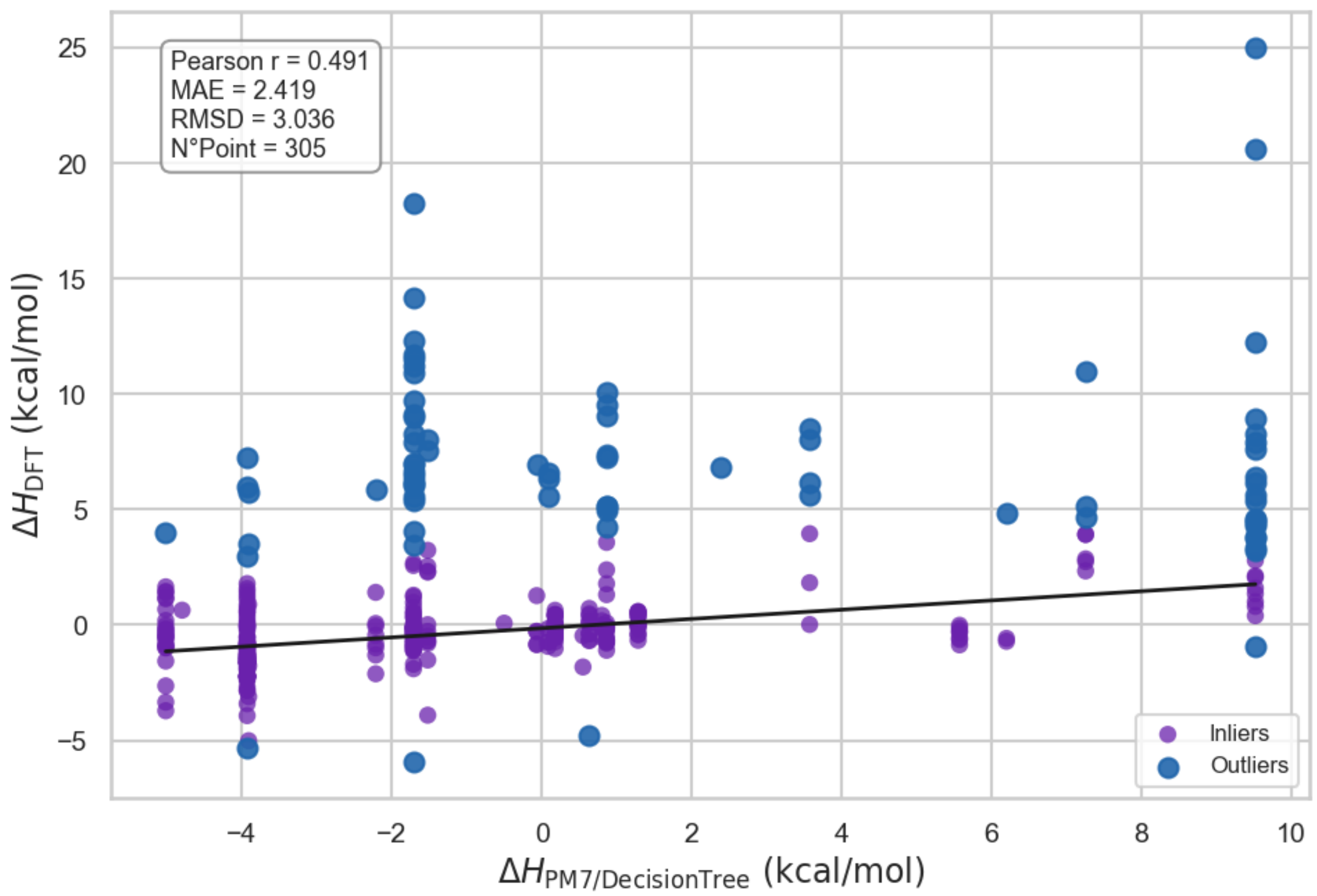

Figure S196: Scatter plots between reference DFT values and the values generated by the the Decision Tree Regressor model trained with PM7-derived features, applying Robust Covariance outlier treatment with a contamination level of 0.20.

S6.4 Isolation Forest

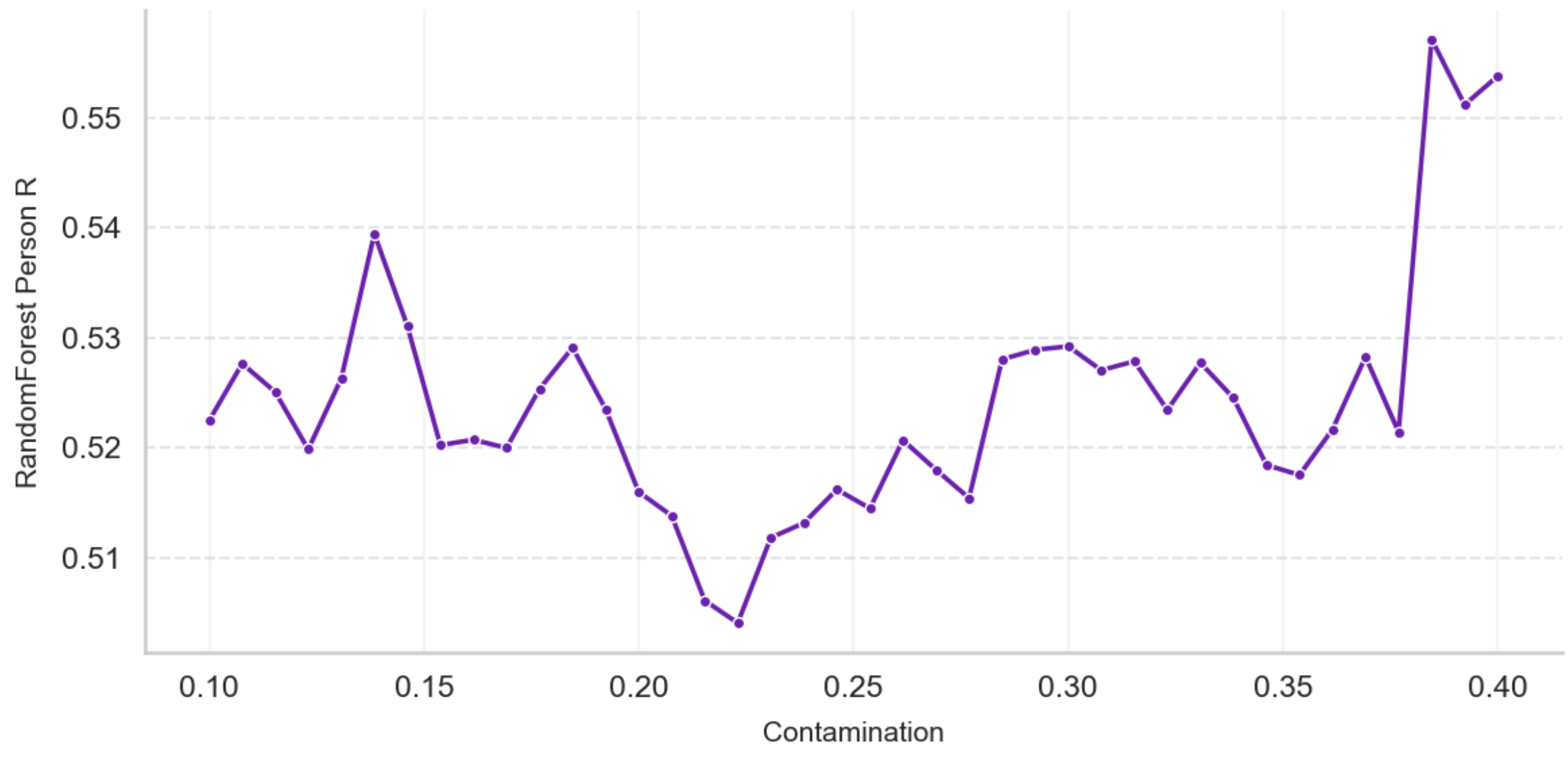

Figure S197: Correlation plot obtained between the data generated by the Random Forest model trained with PM7-derived features and the reference values as a function of data contamination, using Isolation Forest for outlier treatment.

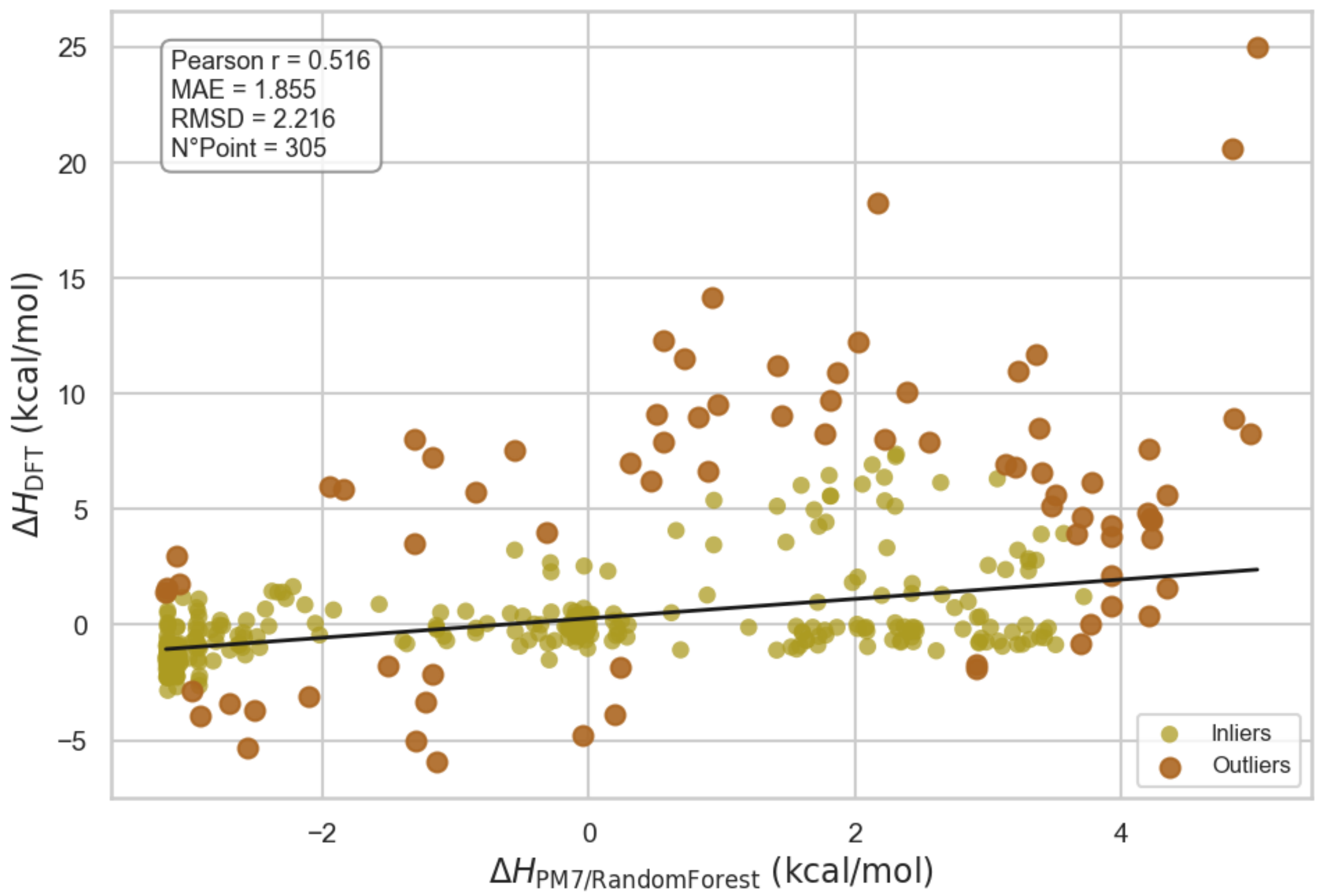

Figure S198: Scatter plots between reference DFT values and the values generated by the Random Forest model trained with PM7-derived features, applying Isolation Forest outlier treatment with a contamination level of 0.20.

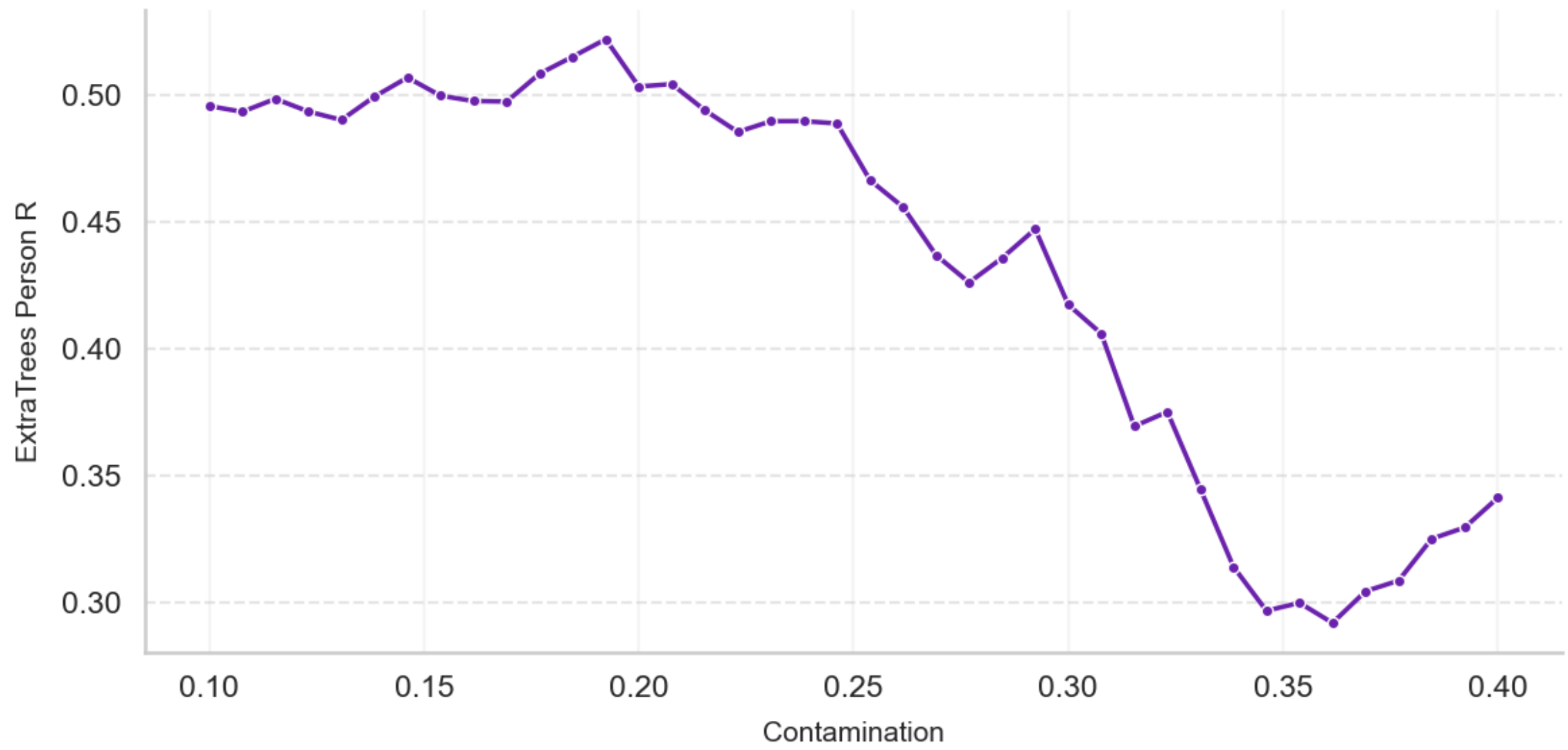

Figure S199: Correlation plot obtained between the data generated by the Extra Trees Regressor model trained with PM7-derived features and the reference values as a function of data contamination, using Isolation Forest for outlier treatment.

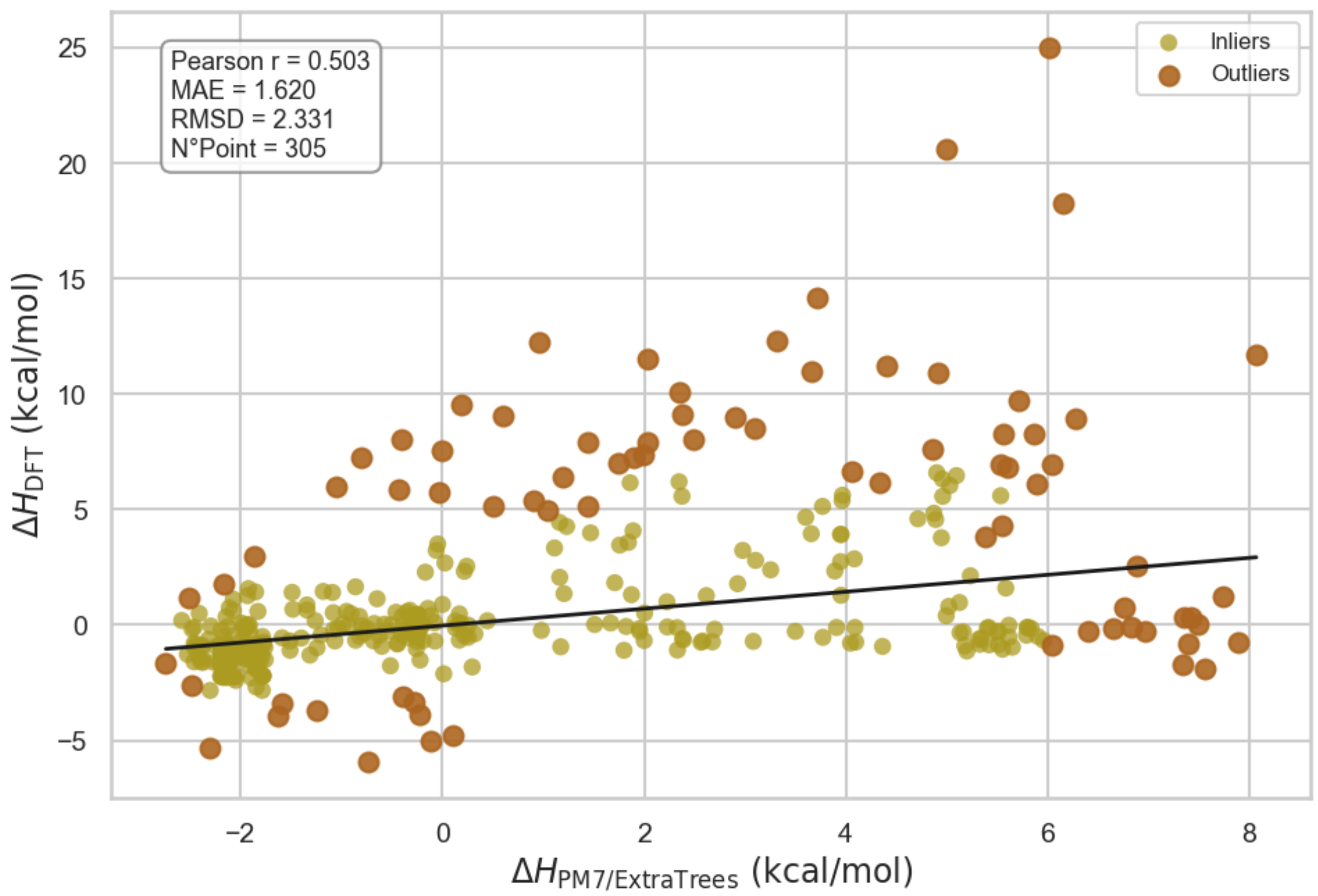

Figure S200: Scatter plots between reference DFT values and the values generated by the Extra Trees Regressor model trained with PM7-derived features, applying Isolation Forest) outlier treatment with a contamination level of 0.20.

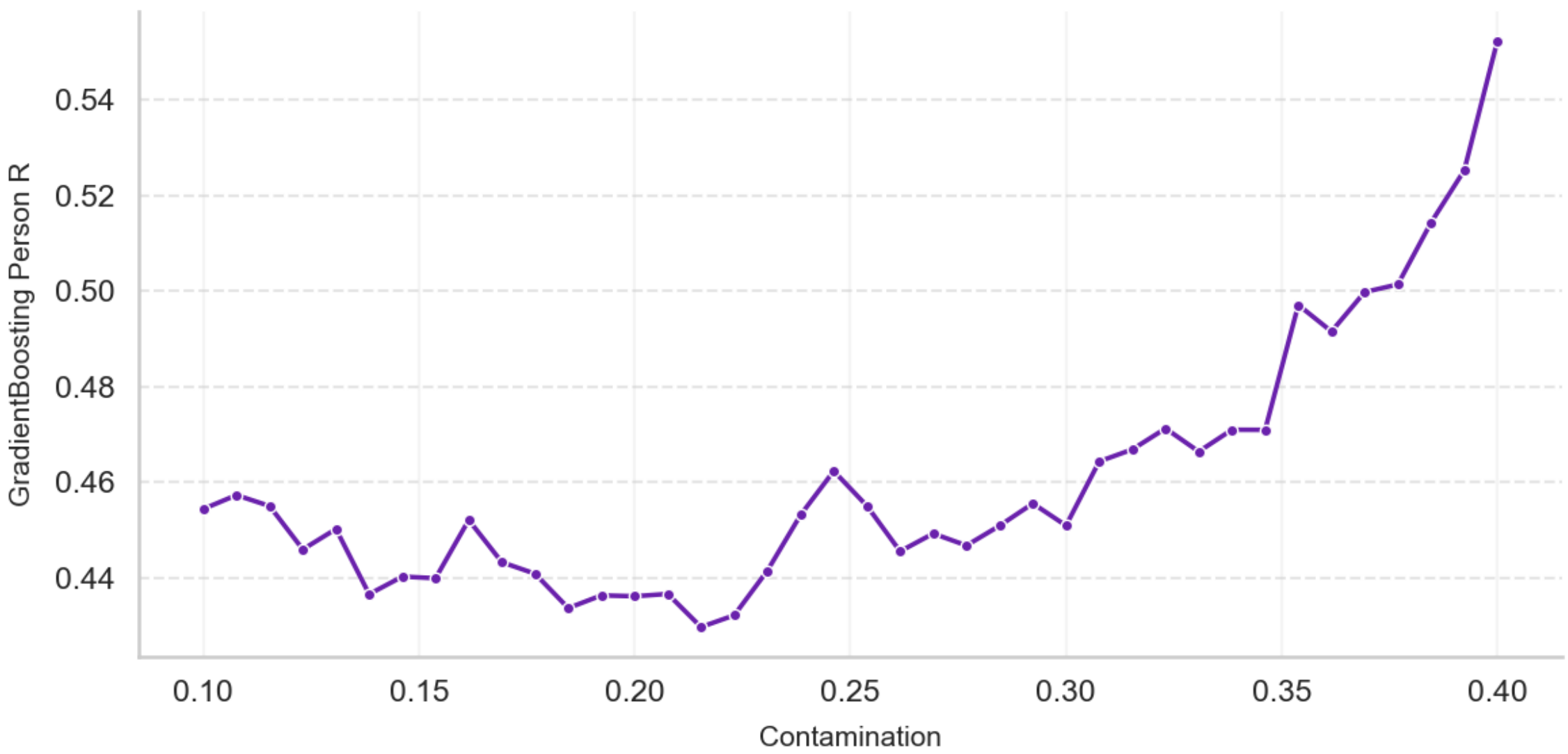

Figure S201: Correlation plot obtained between the data generated by the Gradient Boosting Regressor model trained with PM7-derived features and the reference values as a function of data contamination, using Isolation Forest for outlier treatment.

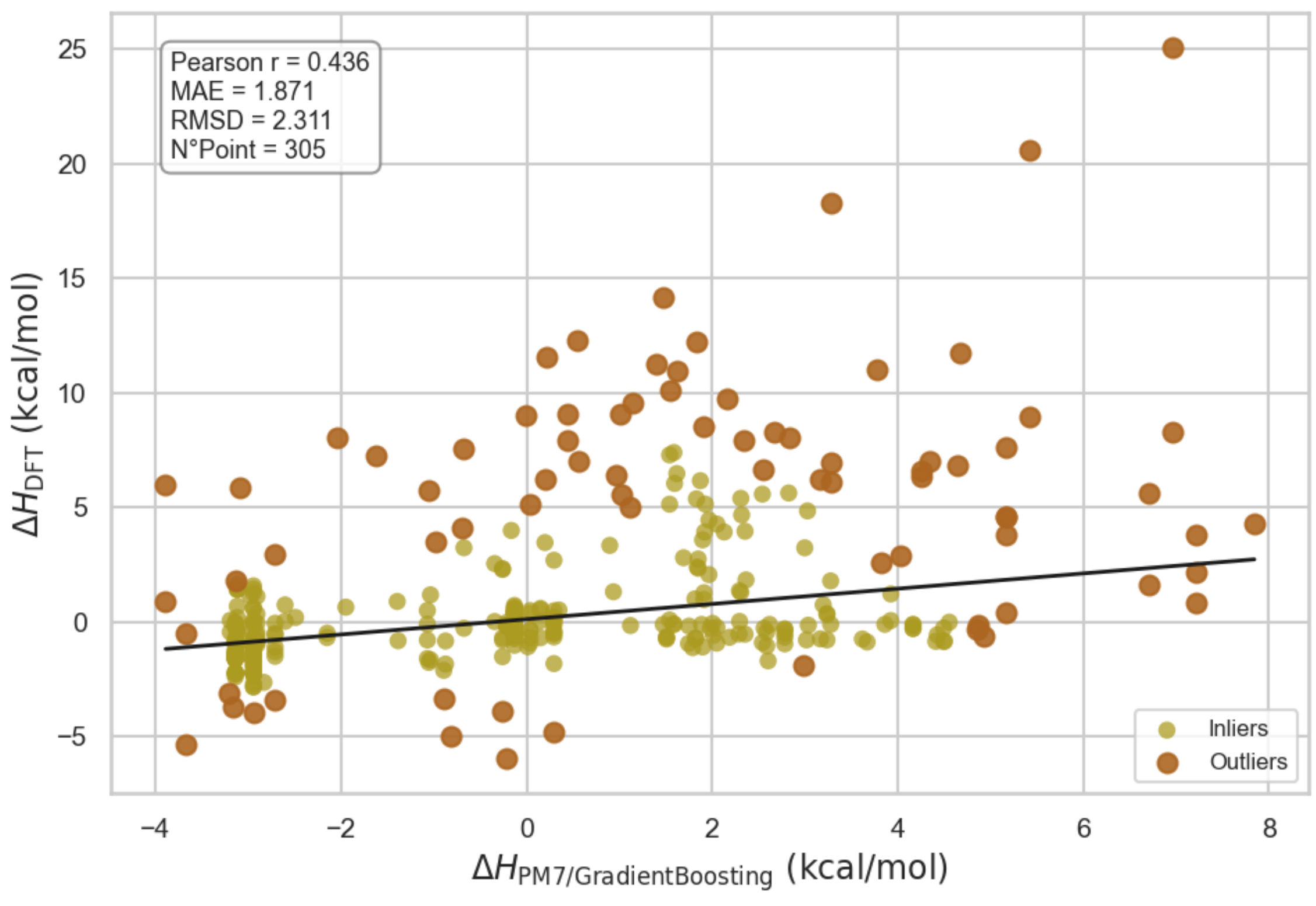

Figure S202: Scatter plots between reference DFT values and the values generated by the Boosting Regressor model trained with PM7-derived features, applying Isolation Forest outlier treatment with a contamination level of 0.20.

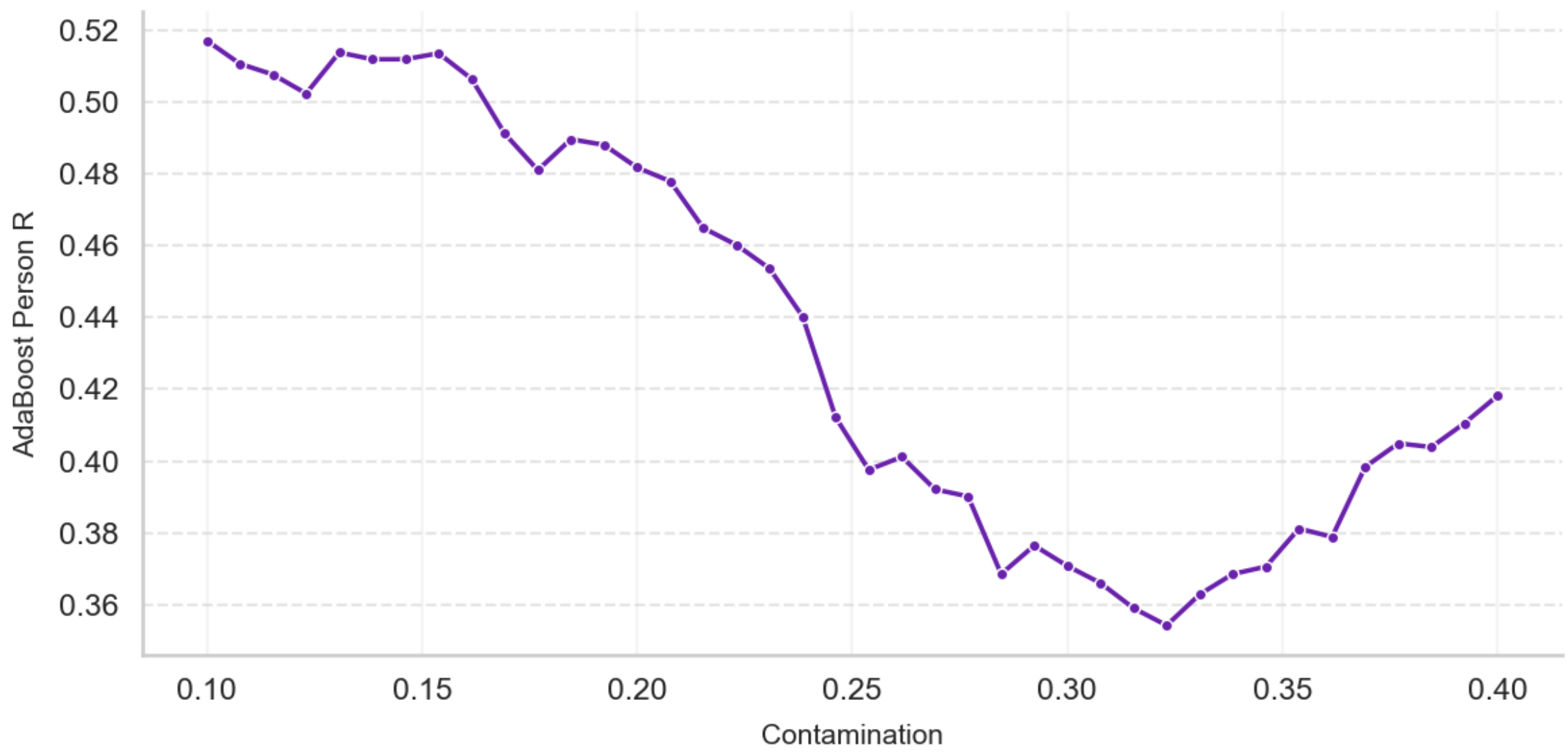

Figure S203: Correlation plot obtained between the data generated by the Ada Boost Regressor model trained with PM7-derived features and the reference values as a function of data contamination, using Isolation Forest for outlier treatment.

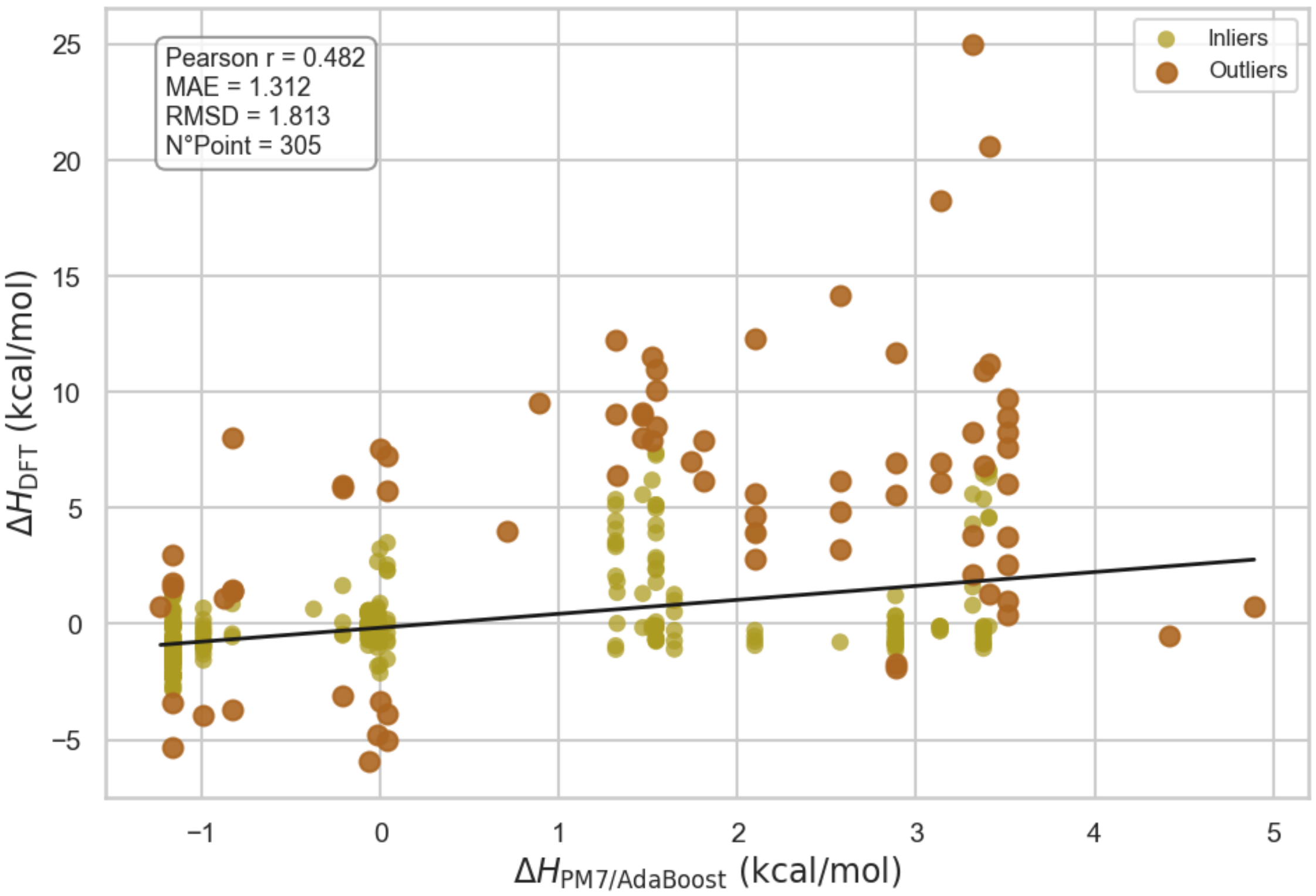

Figure S204: Scatter plots between reference DFT values and the values generated by the Ada Boost Regressor model trained with PM7-derived features, applying Isolation Forest outlier treatment with a contamination level of 0.20.

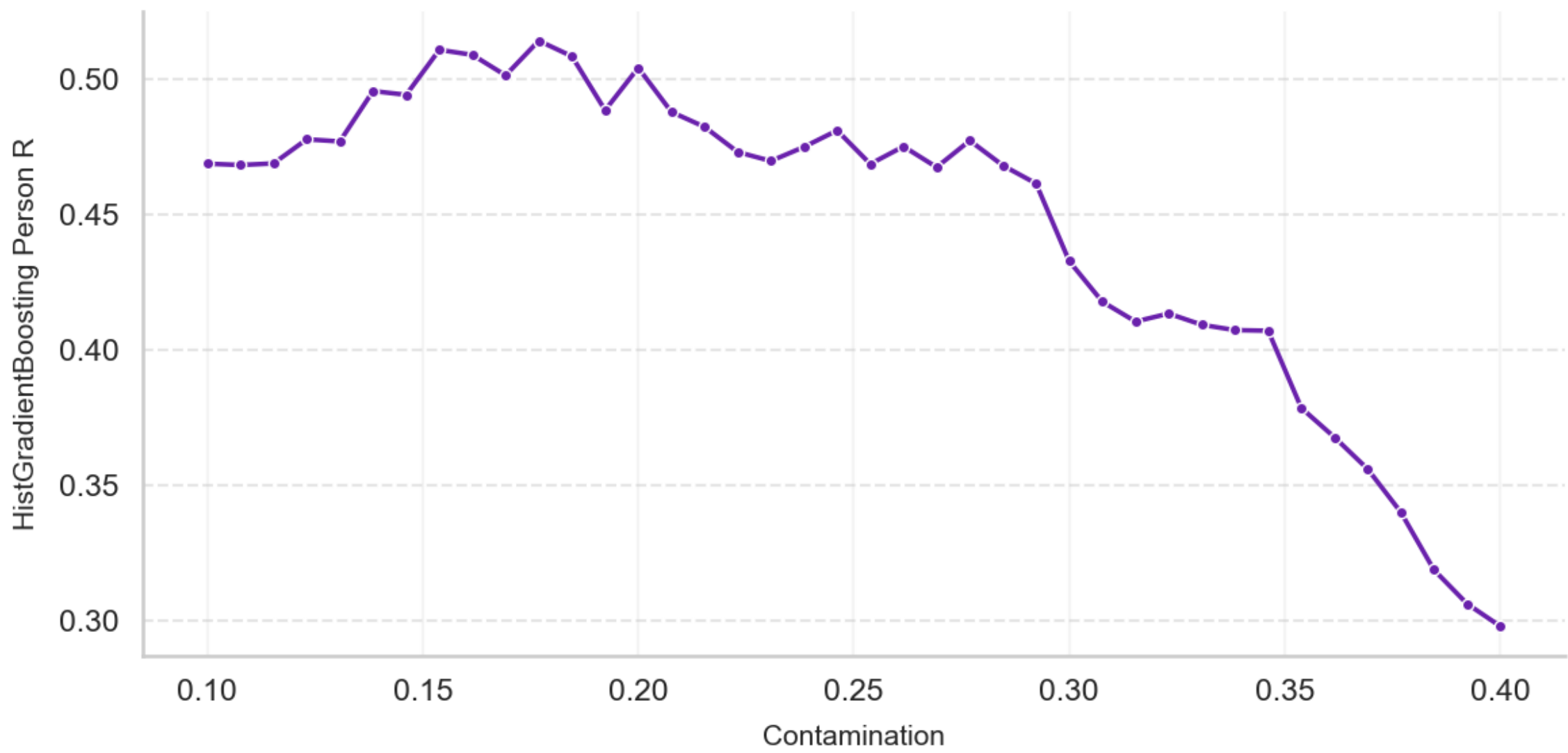

Figure S205: Correlation plot obtained between the data generated by the Hist Gradient Boosting model trained with PM7-derived features and the reference values as a function of data contamination, using Isolation Forest for outlier treatment.

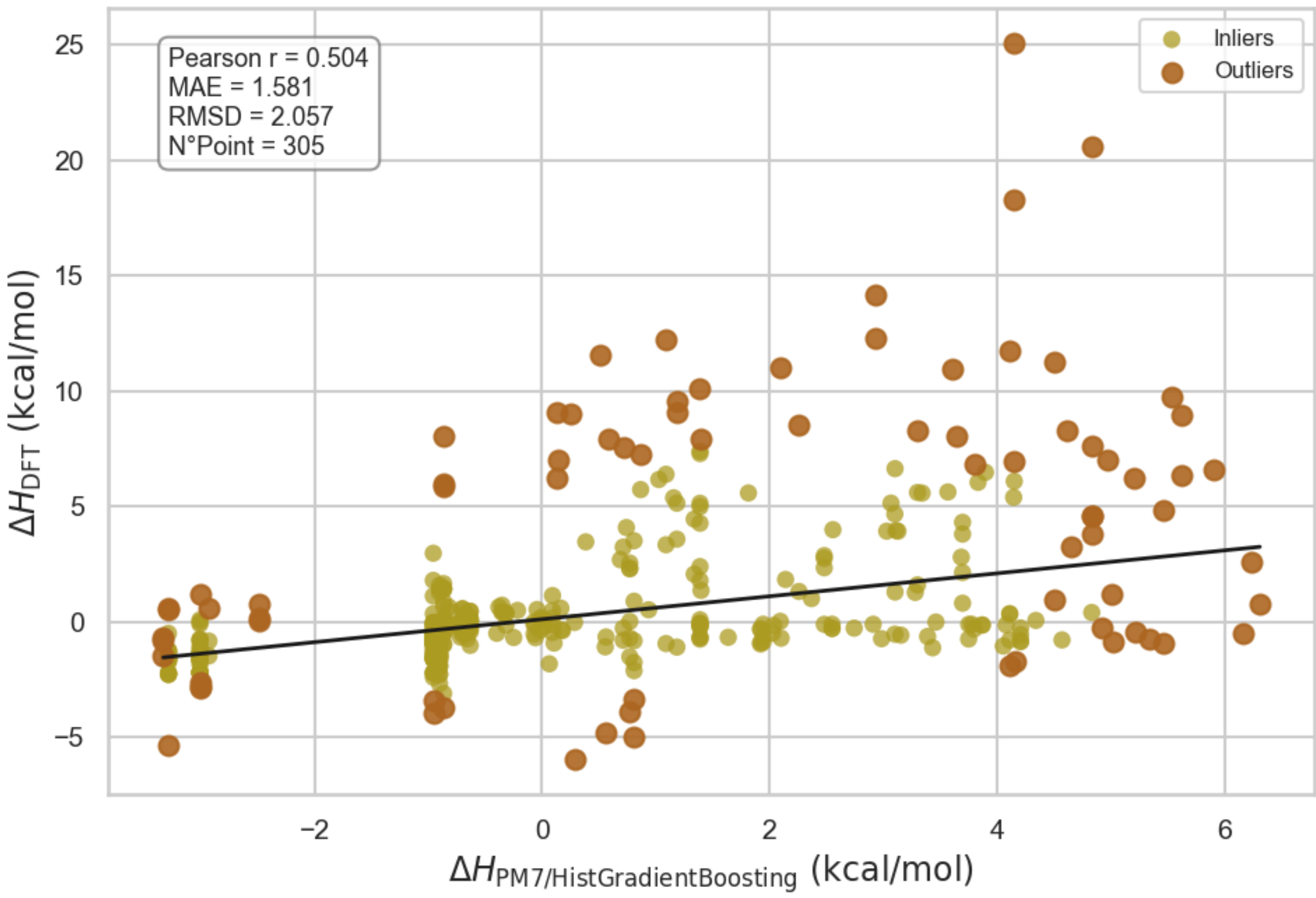

Figure S206: Scatter plots between reference DFT values and the values generated by the Hist Gradient Boosting model trained with PM7-derived features, applying Isolation Forest outlier treatment with a contamination level of 0.20.

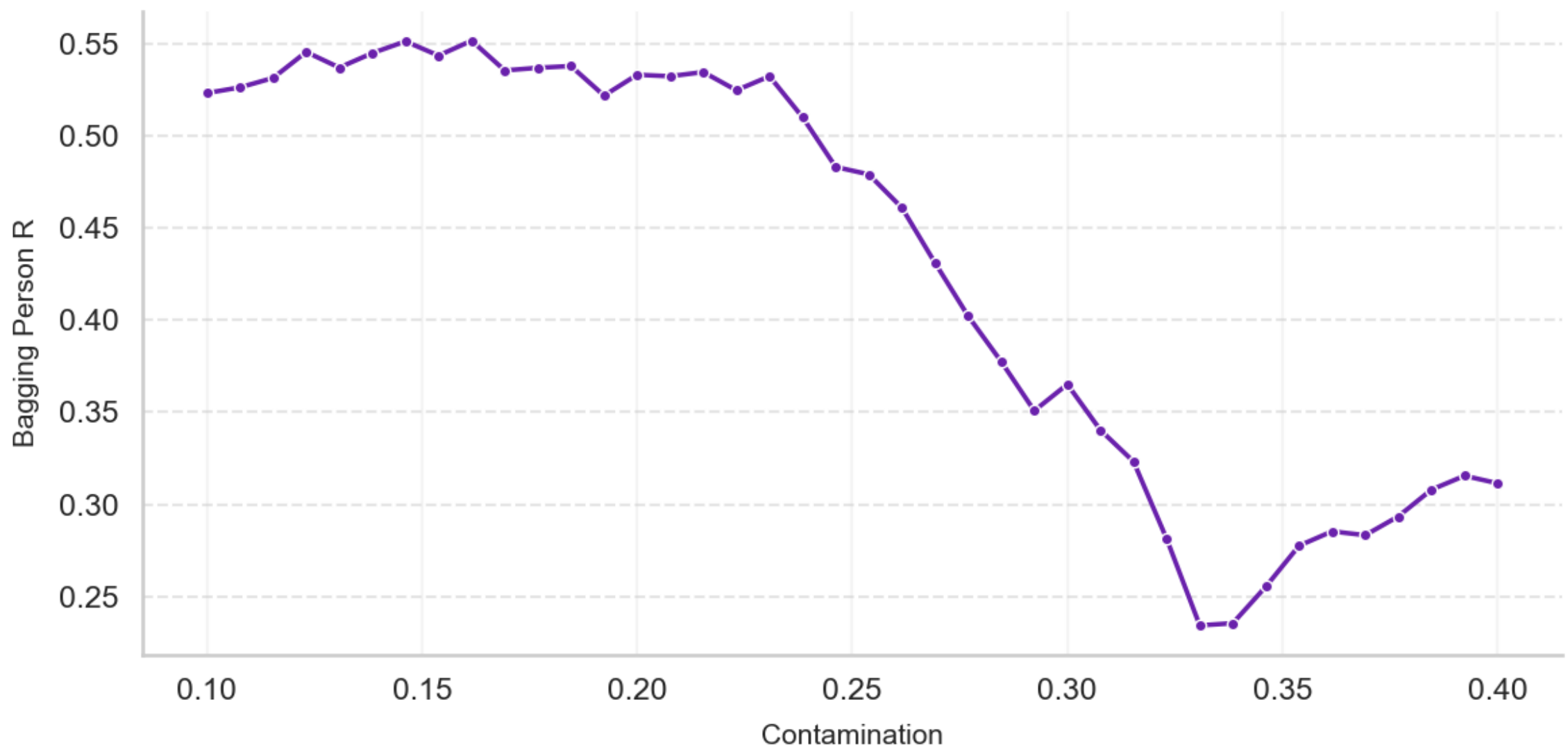

Figure S207: Correlation plot obtained between the data generated by the Bagging Regressor model trained with PM7-derived features and the reference values as a function of data contamination, using Isolation Forest for outlier treatment.

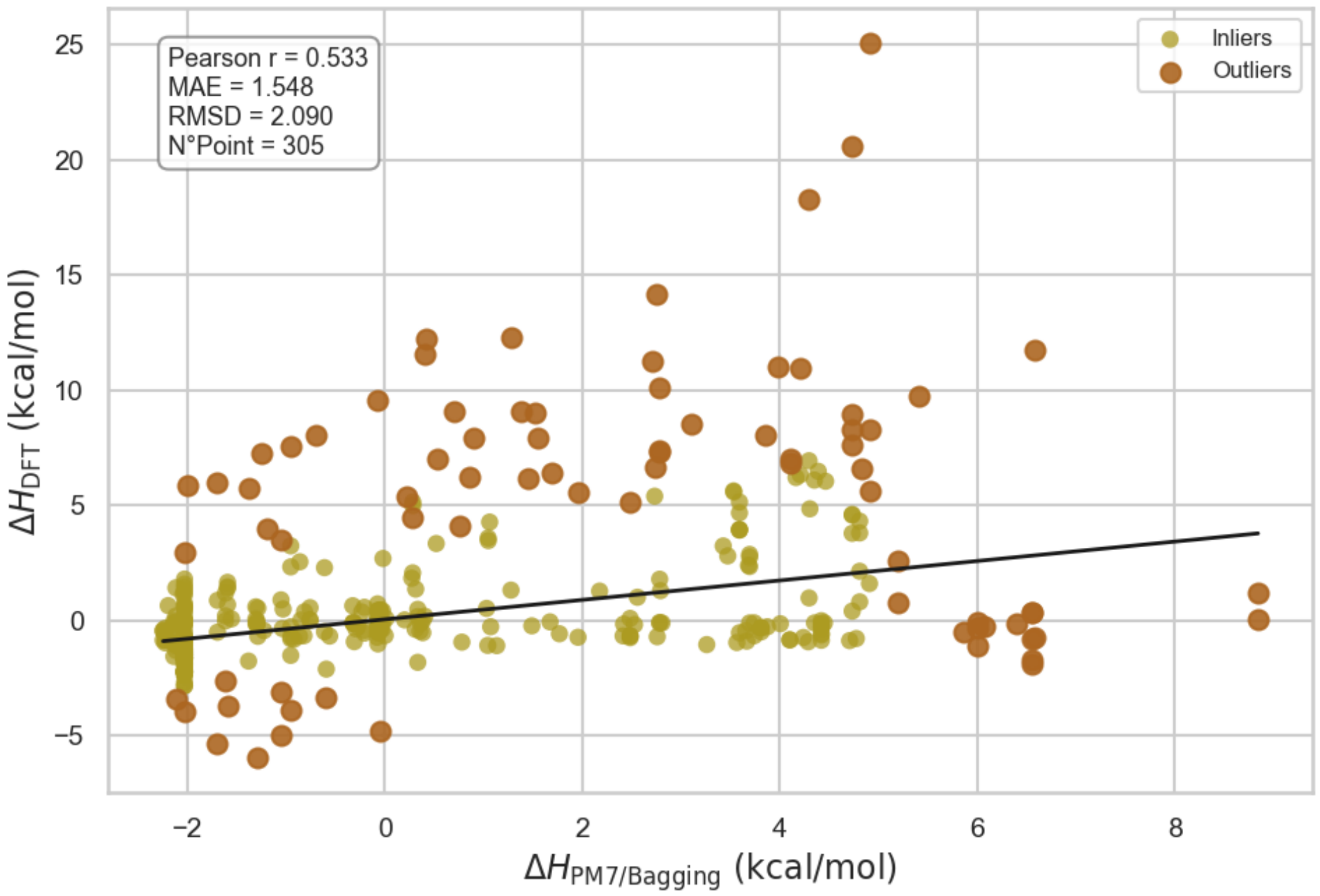

Figure S208: Scatter plots between reference DFT values and the values generated by the Bagging Regressor model trained with PM7-derived features, applying Isolation Forest outlier treatment with a contamination level of 0.20.

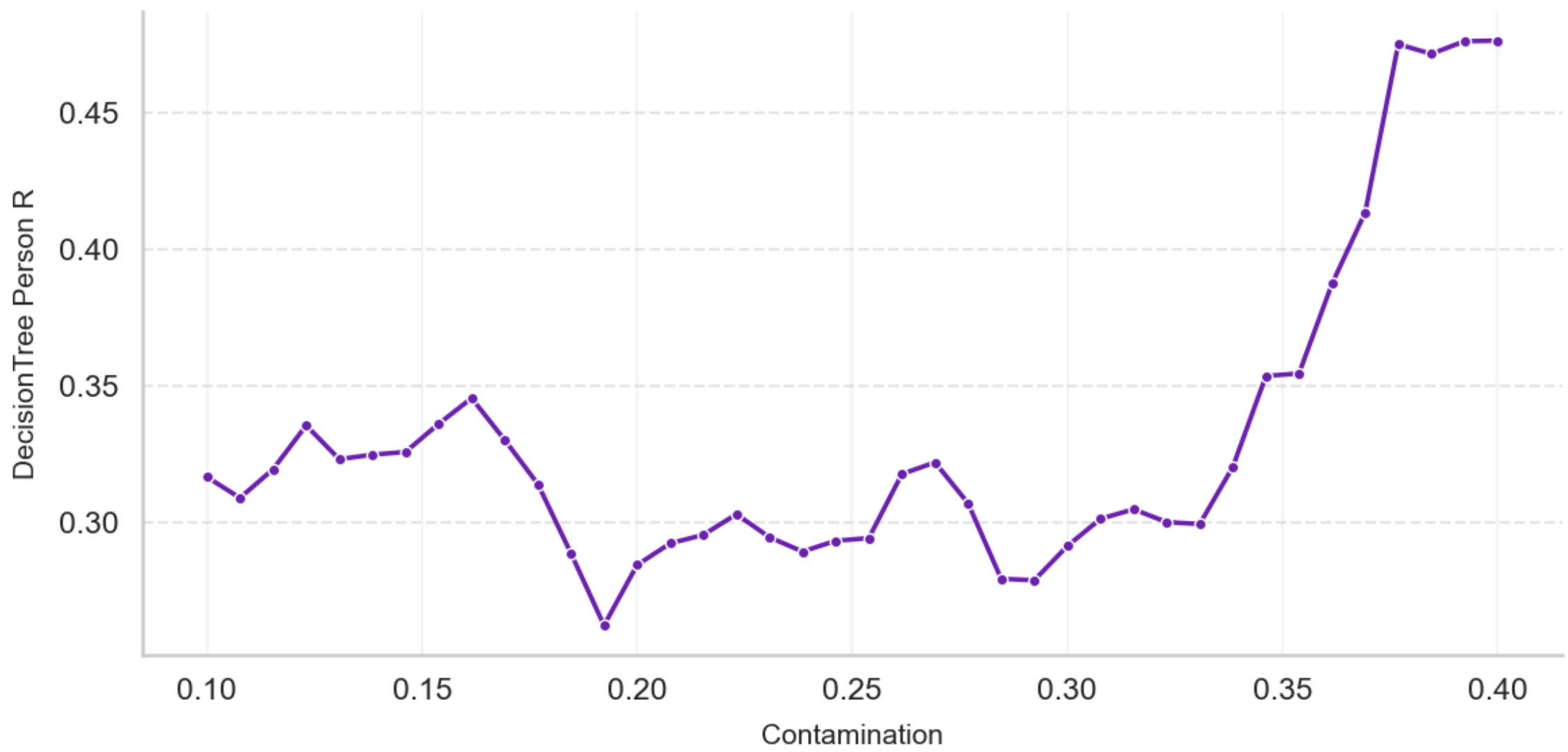

Figure S209: Correlation plot obtained between the data generated by the Decision Tree Regressor model trained with PM7-derived features and the reference values as a function of data contamination, using Isolation Forest for outlier treatment.

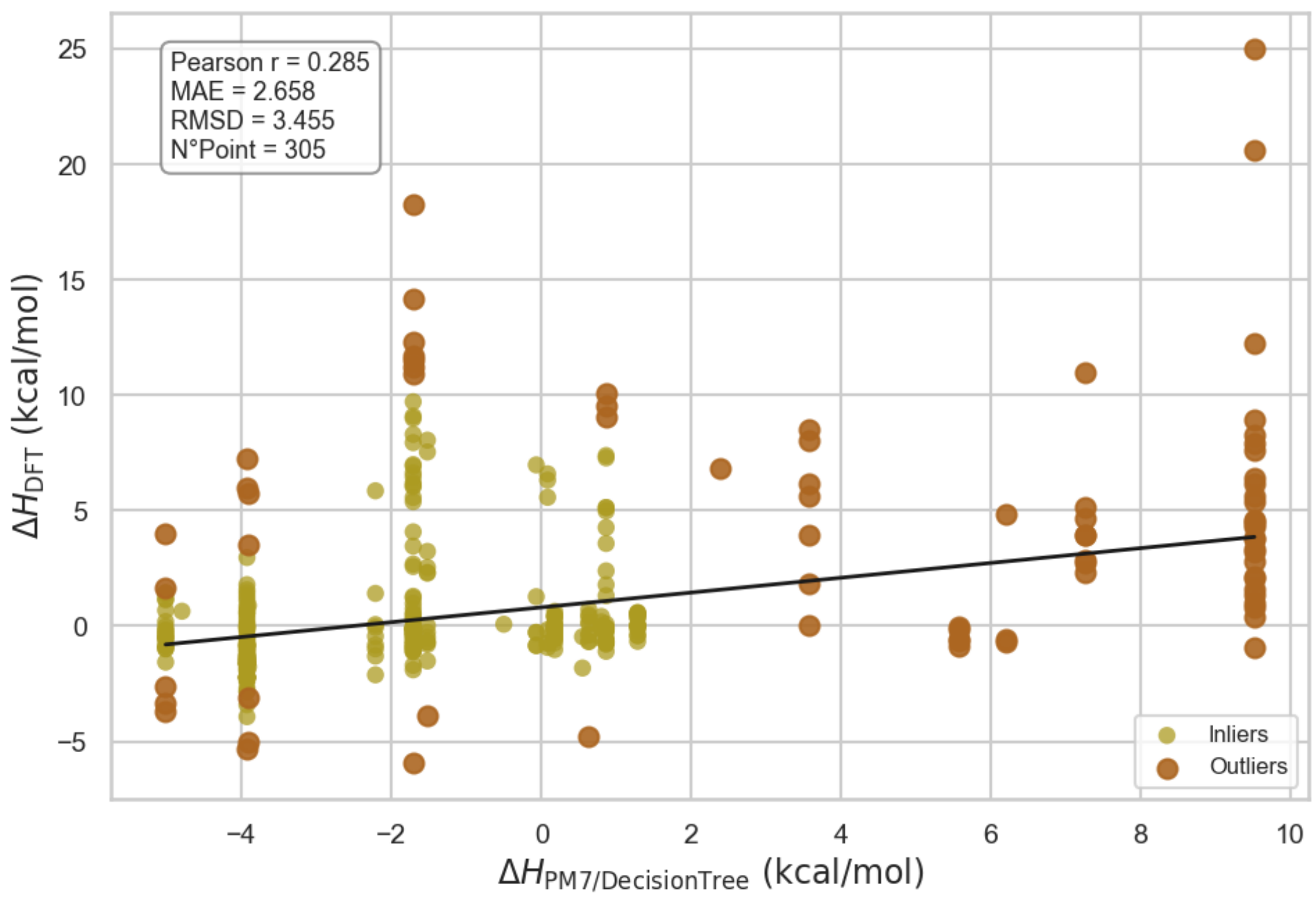

Figure S210: Scatter plots between reference DFT values and the values generated by the the Decision Tree Regressor model trained with PM7-derived features, applying Isolation Forest outlier treatment with a contamination level of 0.20.

S6.5 Local Outlier Factor

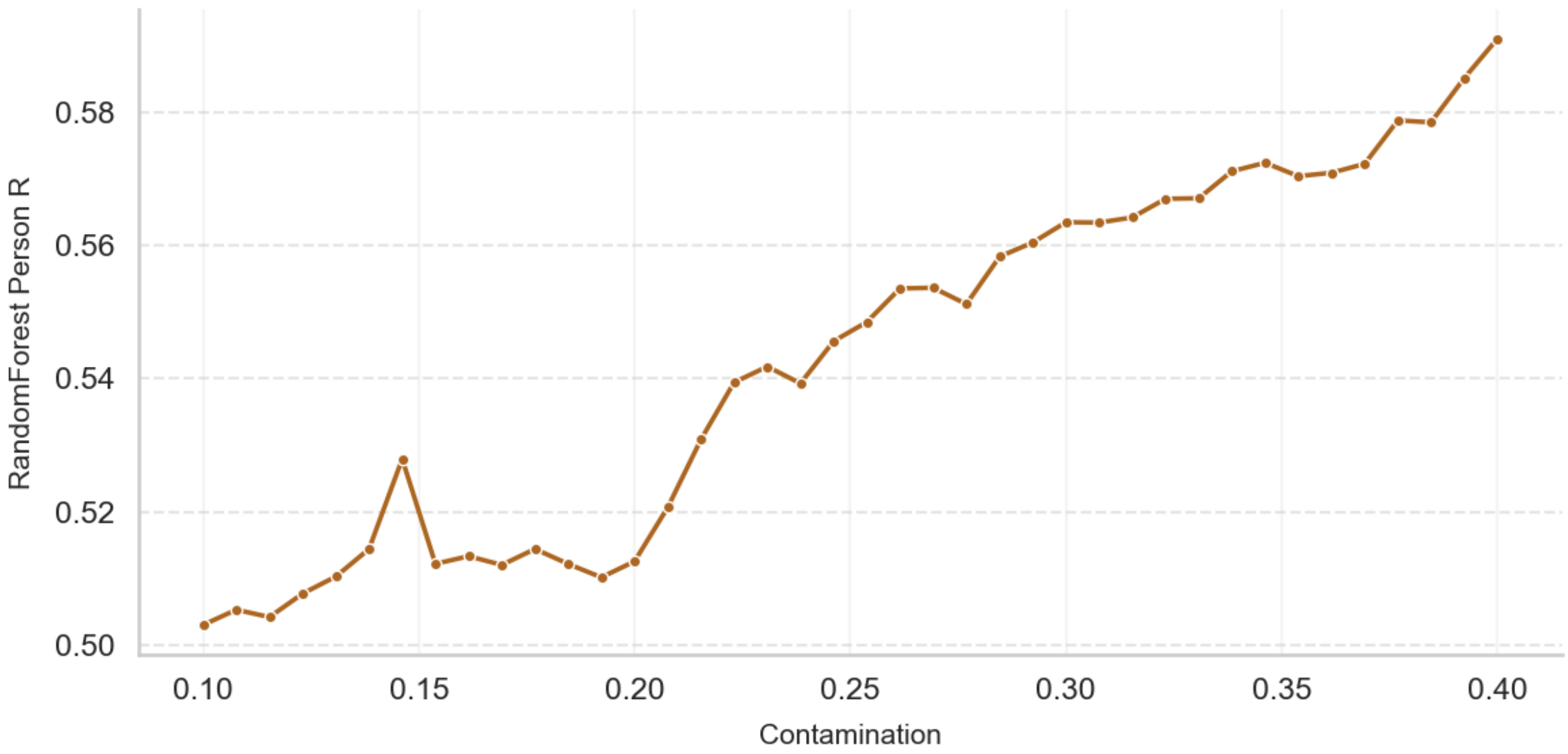

Figure S211: Correlation plot obtained between the data generated by the Random Forest model trained with PM7-derived features and the reference values as a function of data contamination, using Local Outlier Factor for outlier treatment.

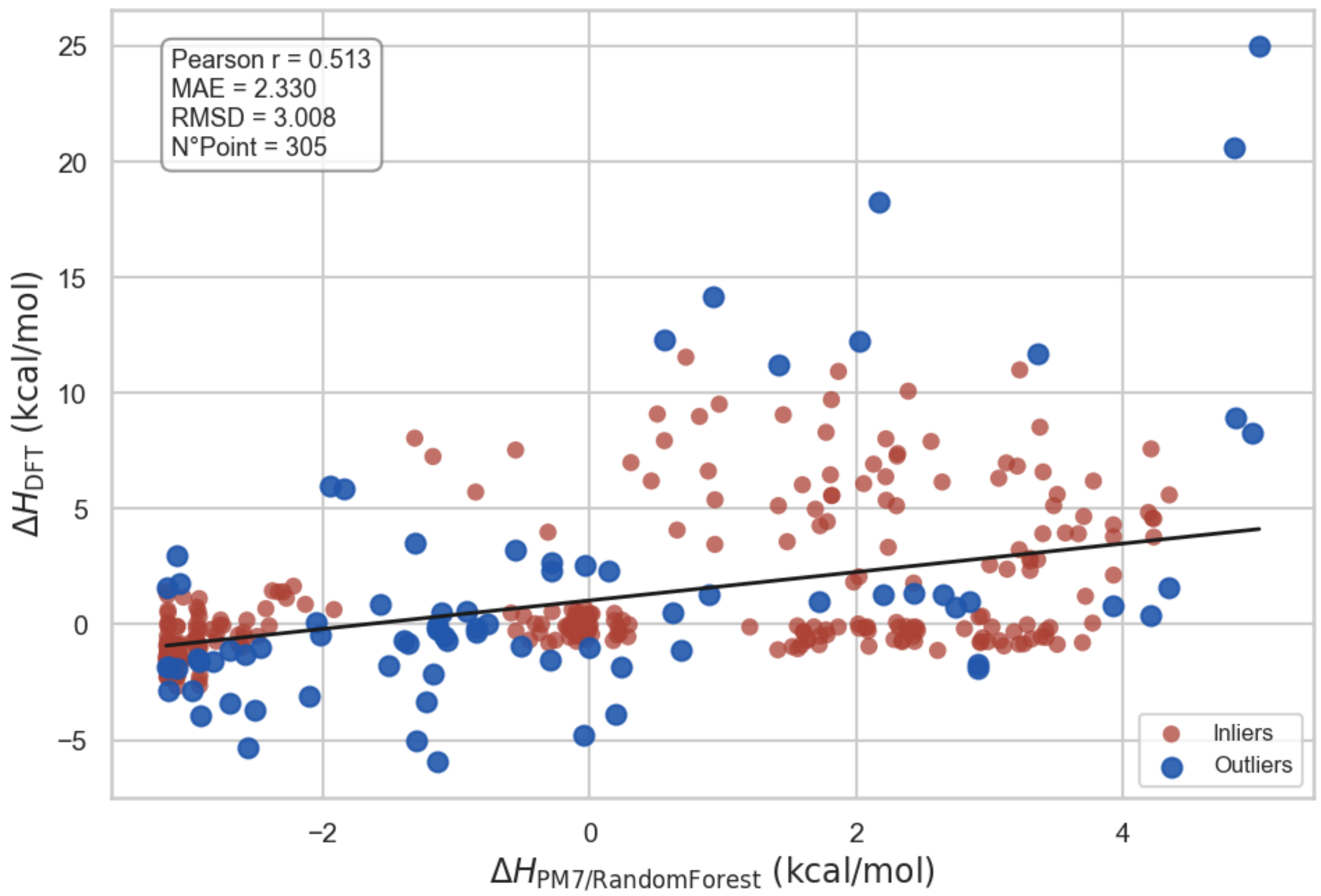

Figure S212: Scatter plots between reference DFT values and the values generated by the Random Forest model trained with PM7-derived features, applying Local Outlier Factor outlier treatment with a contamination level of 0.20.

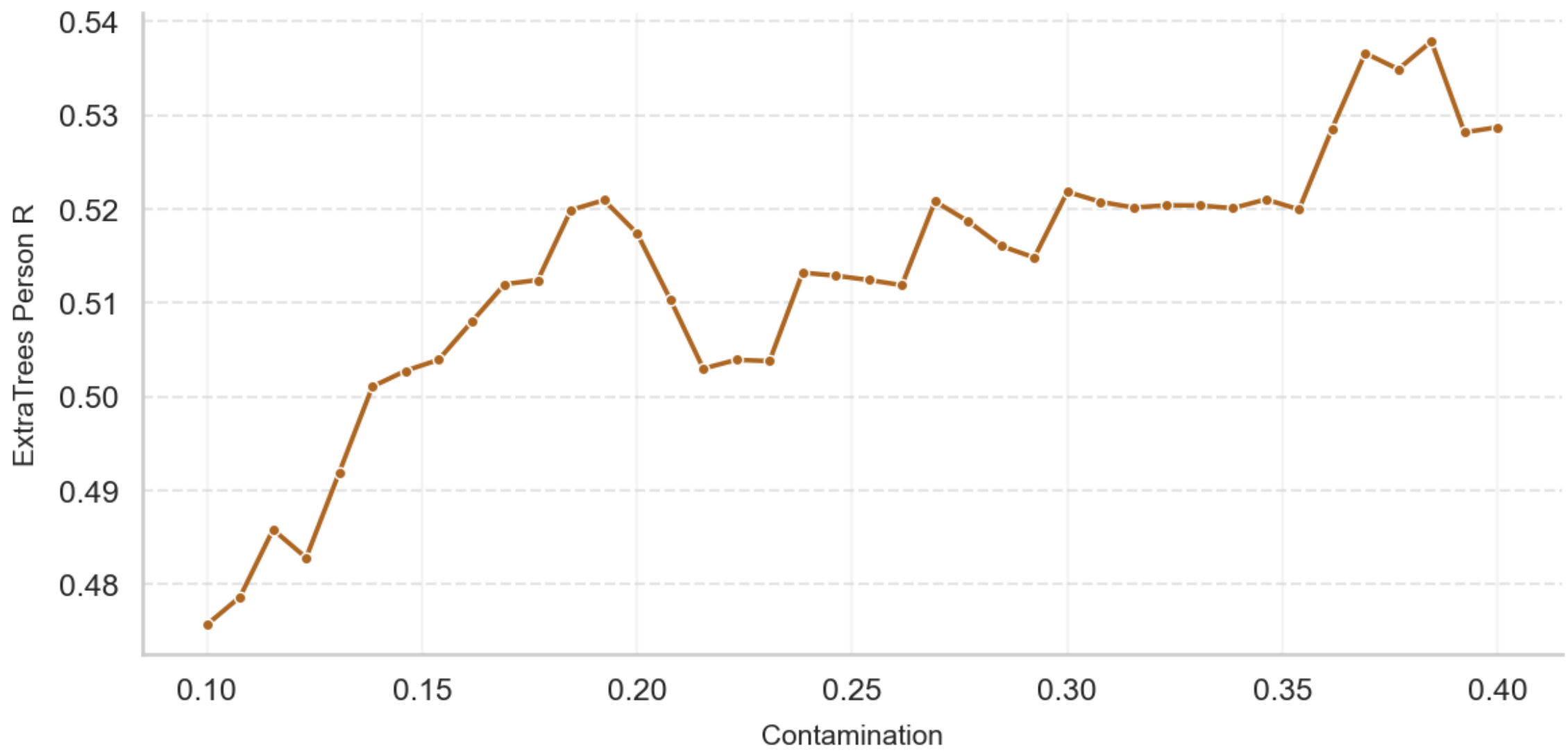

Figure S213: Correlation plot obtained between the data generated by the Extra Trees Regressor model trained with PM7-derived features and the reference values as a function of data contamination, using Local Outlier Factor for outlier treatment.

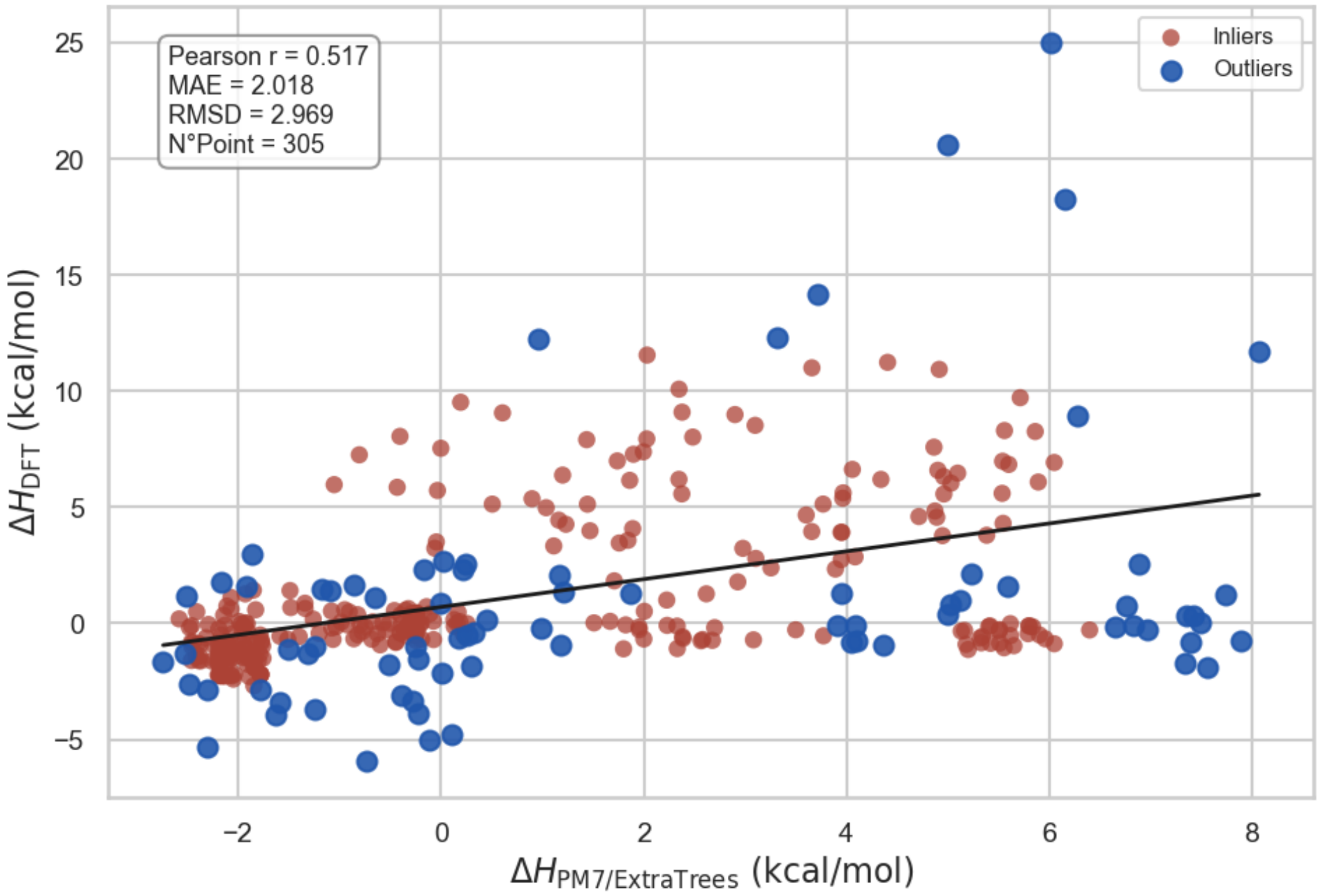

Figure S214: Scatter plots between reference DFT values and the values generated by the Extra Trees Regressor model trained with PM7-derived features, applying Local Outlier Factor outlier treatment with a contamination level of 0.20.

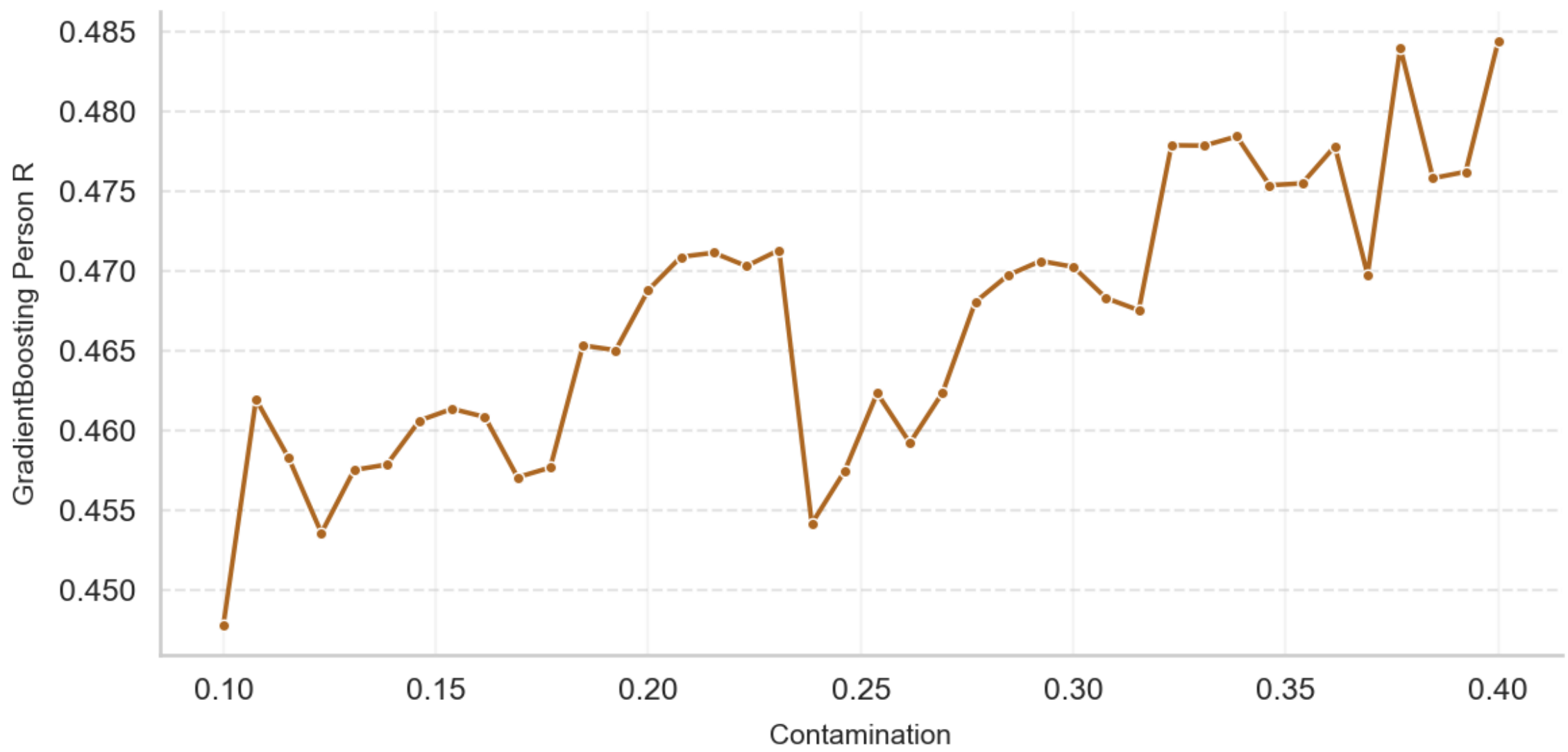

Figure S215: Correlation plot obtained between the data generated by the Gradient Boosting Regressor model trained with PM7-derived features and the reference values as a function of data contamination, using Local Outlier Factor for outlier treatment.

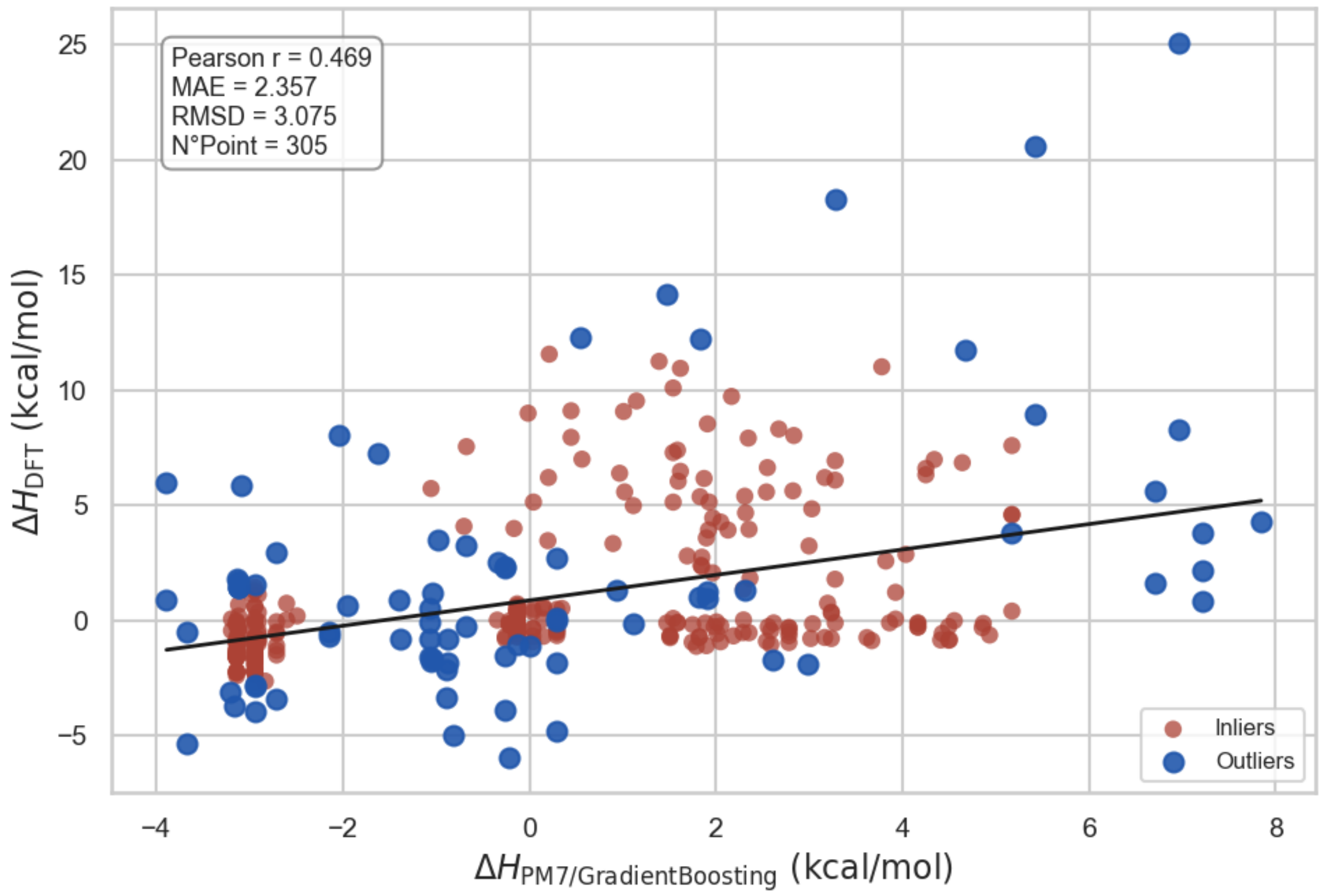

Figure S216: Scatter plots between reference DFT values and the values generated by the Boosting Regressor model trained with PM7-derived features, applying Local Outlier Factor outlier treatment with a contamination level of 0.20.

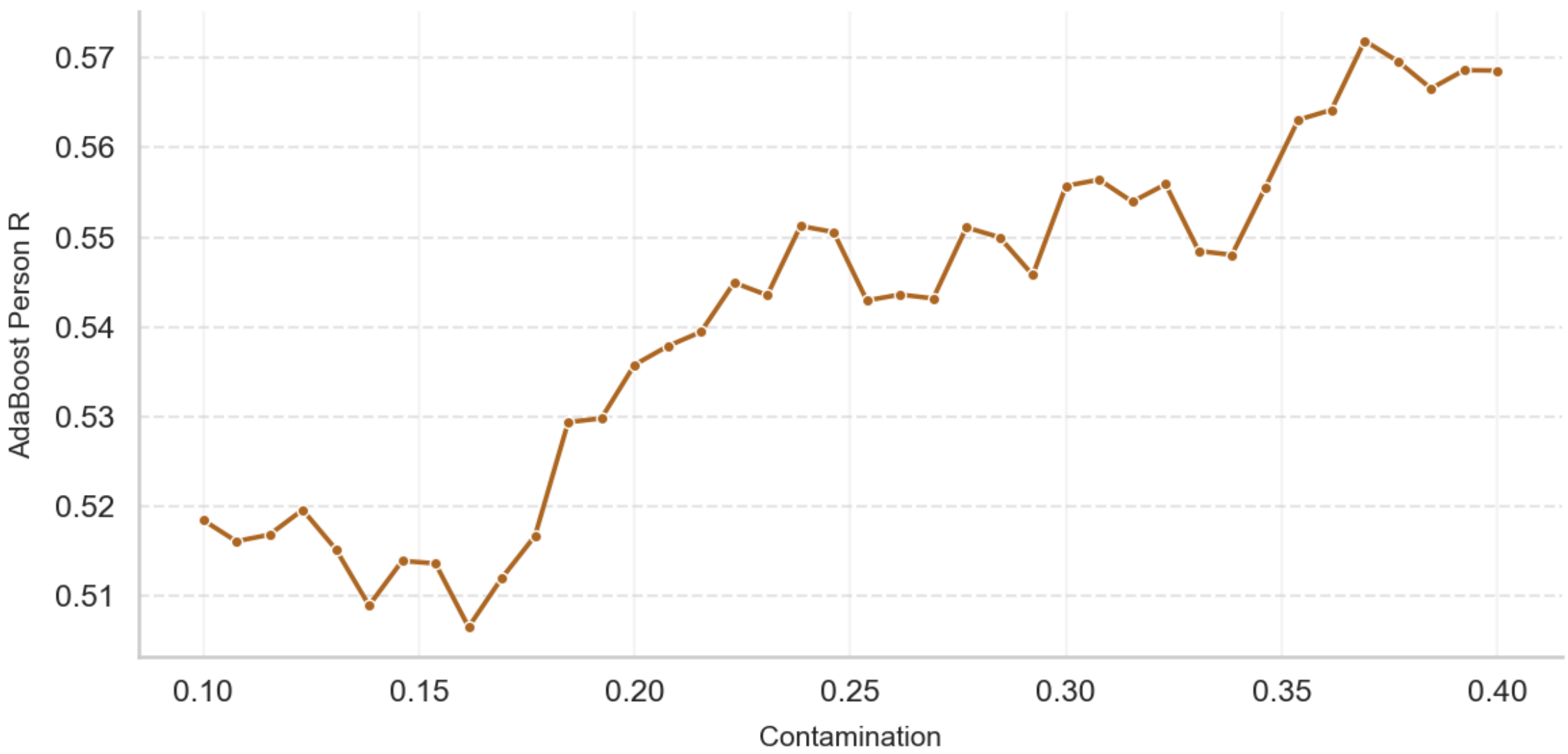

Figure S217: Correlation plot obtained between the data generated by the Ada Boost Regressor model trained with PM7-derived features and the reference values as a function of data contamination, using Local Outlier Factor for outlier treatment.

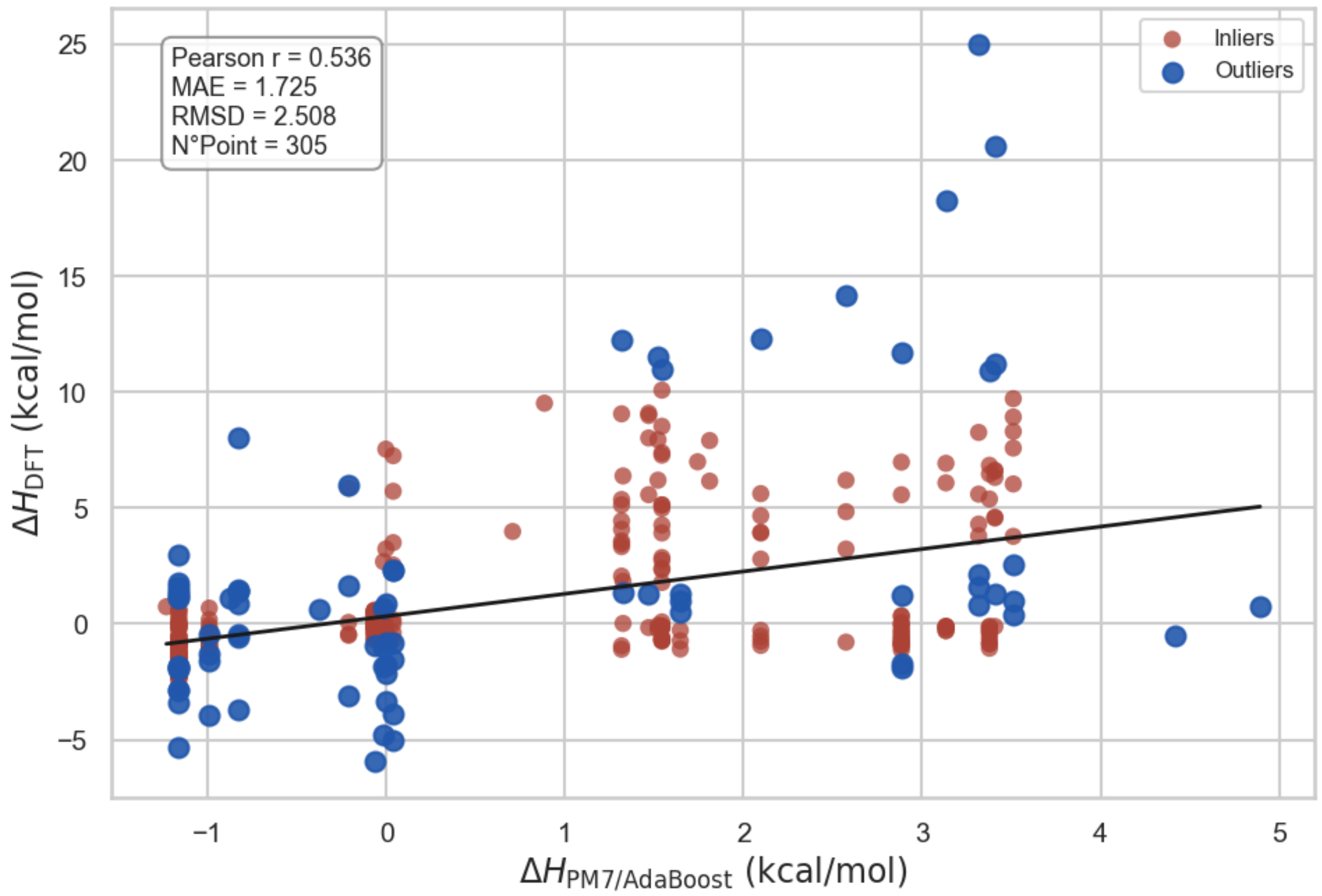

Figure S218: Scatter plots between reference DFT values and the values generated by the Ada Boost Regressor model trained with PM7-derived features, applying Local Outlier Factor outlier treatment with a contamination level of 0.20.

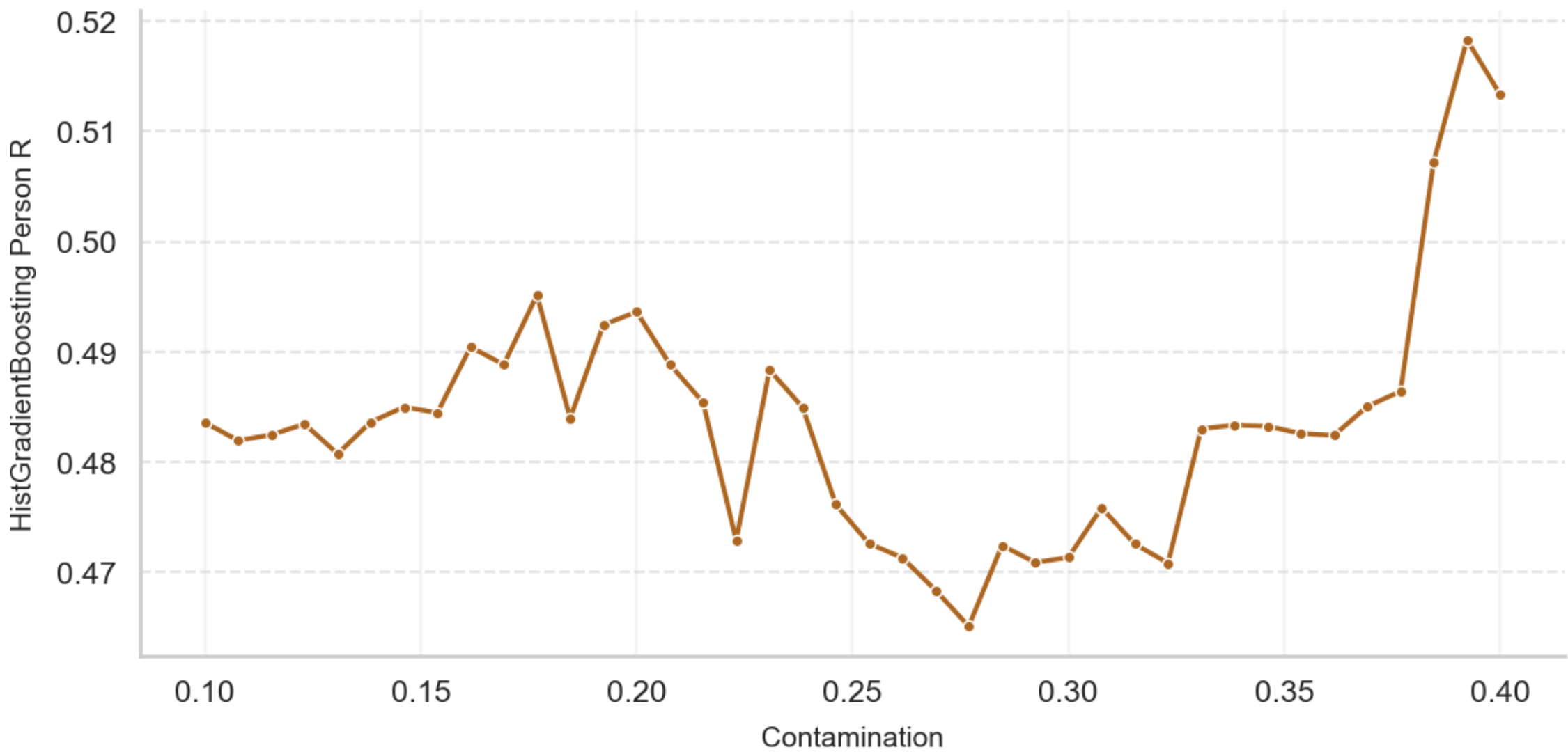

Figure S219: Correlation plot obtained between the data generated by the Hist Gradient Boosting model trained with PM7-derived features and the reference values as a function of data contamination, using Local Outlier Factor for outlier treatment.

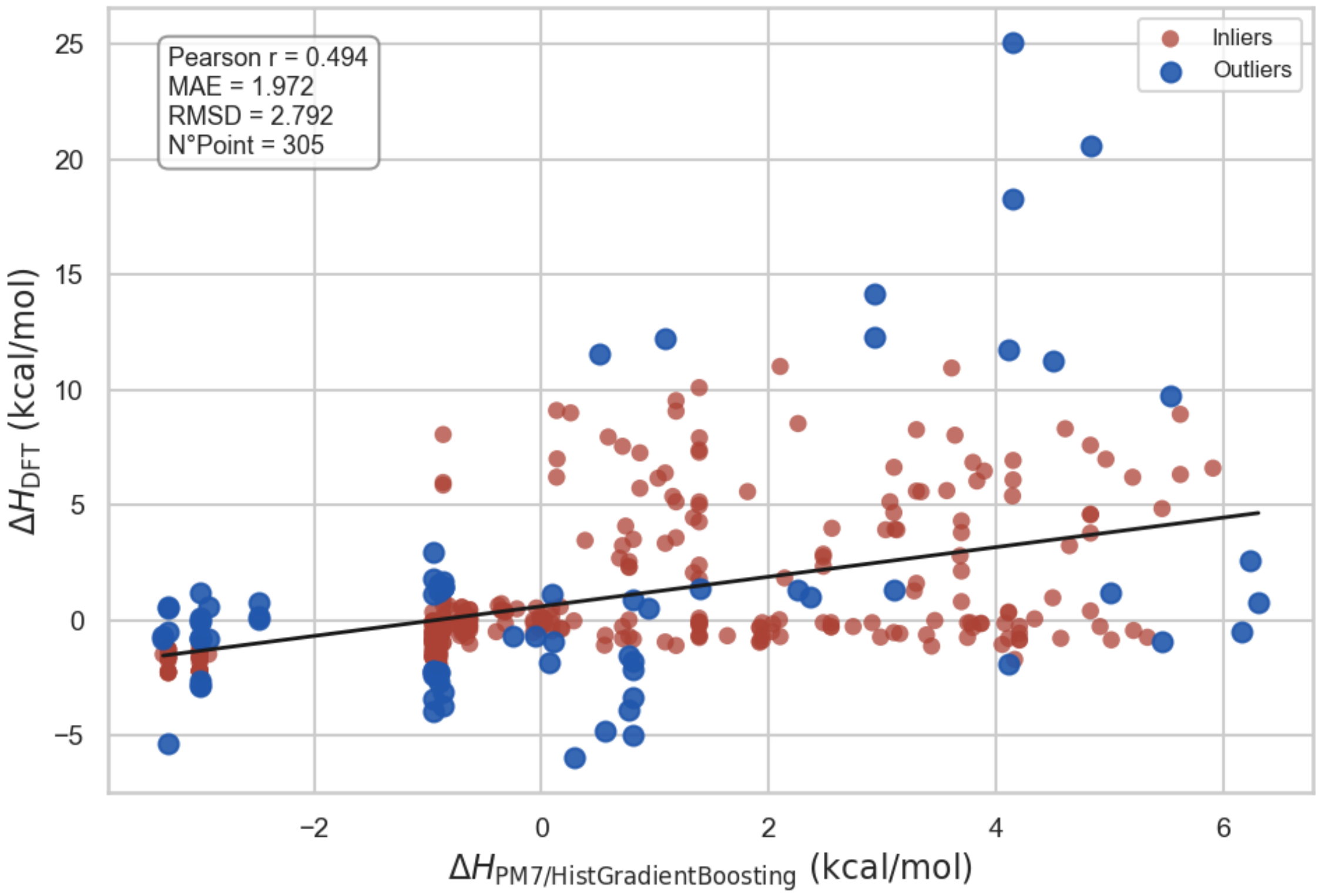

Figure S220: Scatter plots between reference DFT values and the values generated by the Hist Gradient Boosting model trained with PM7-derived features, applying Local Outlier Factor outlier treatment with a contamination level of 0.20.

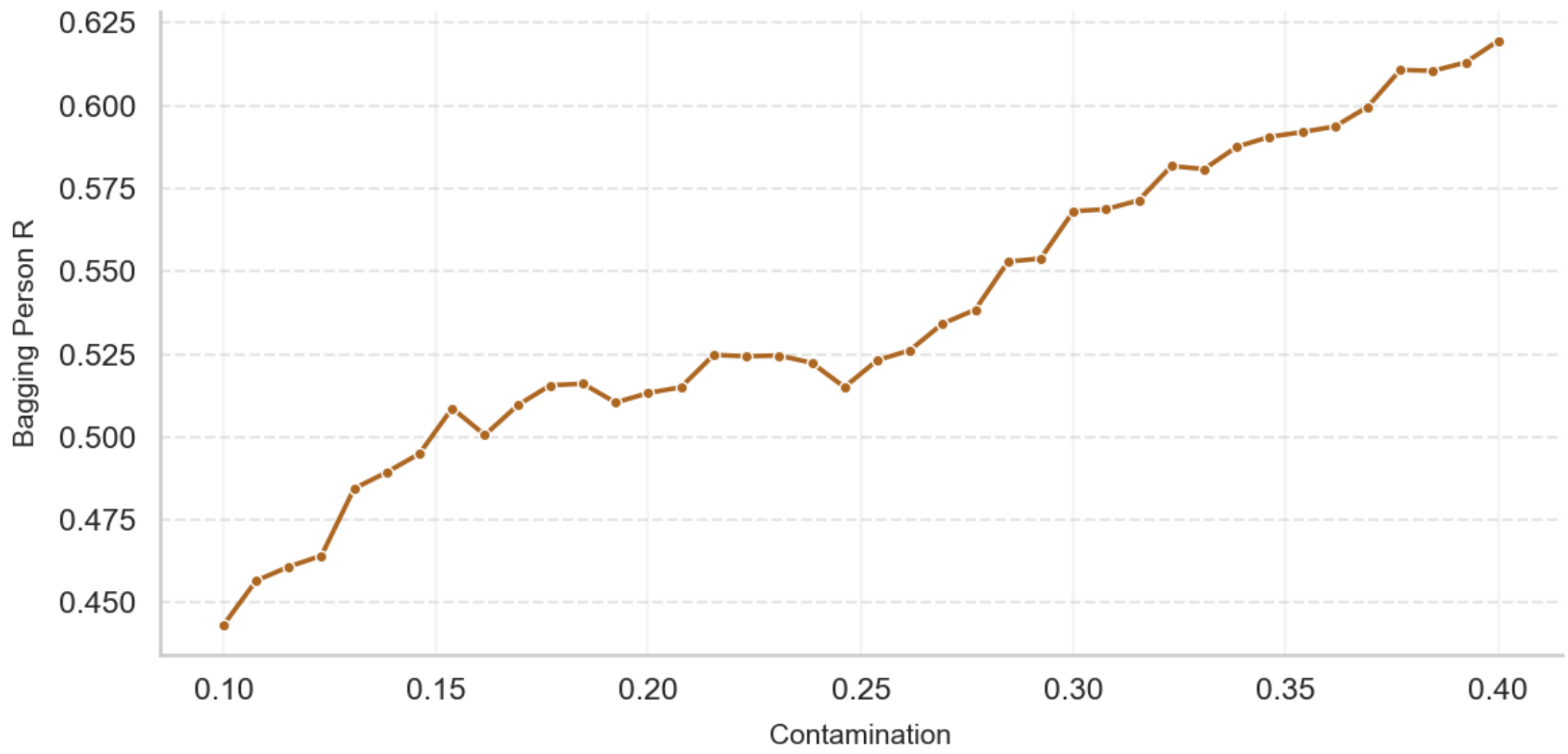

Figure S221: Correlation plot obtained between the data generated by the Bagging Regressor model trained with PM7-derived features and the reference values as a function of data contamination, using Local Outlier Factor for outlier treatment.

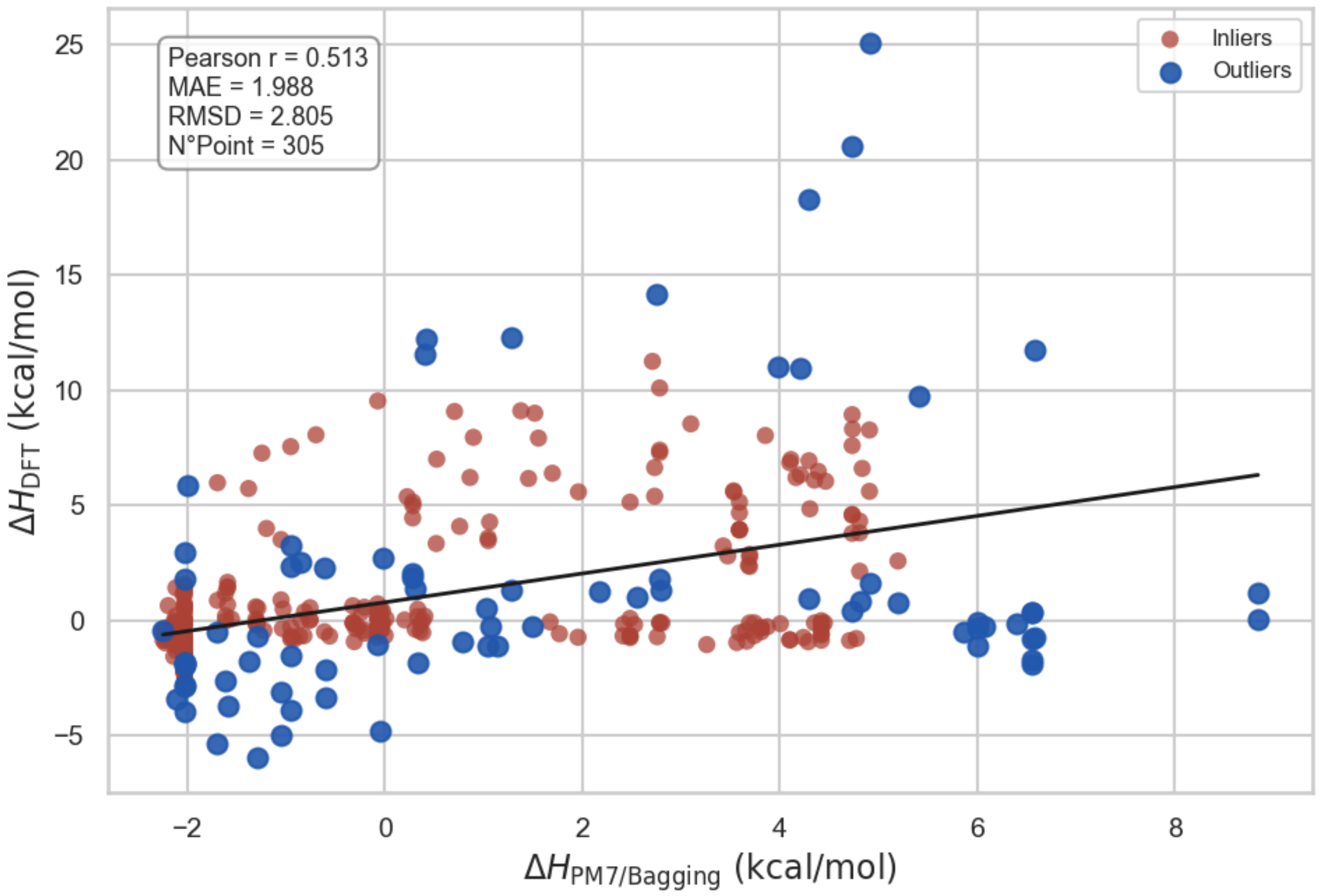

Figure S222: Scatter plots between reference DFT values and the values generated by the Bagging Regressor model trained with PM7-derived features, applying Local Outlier Factor outlier treatment with a contamination level of 0.20.

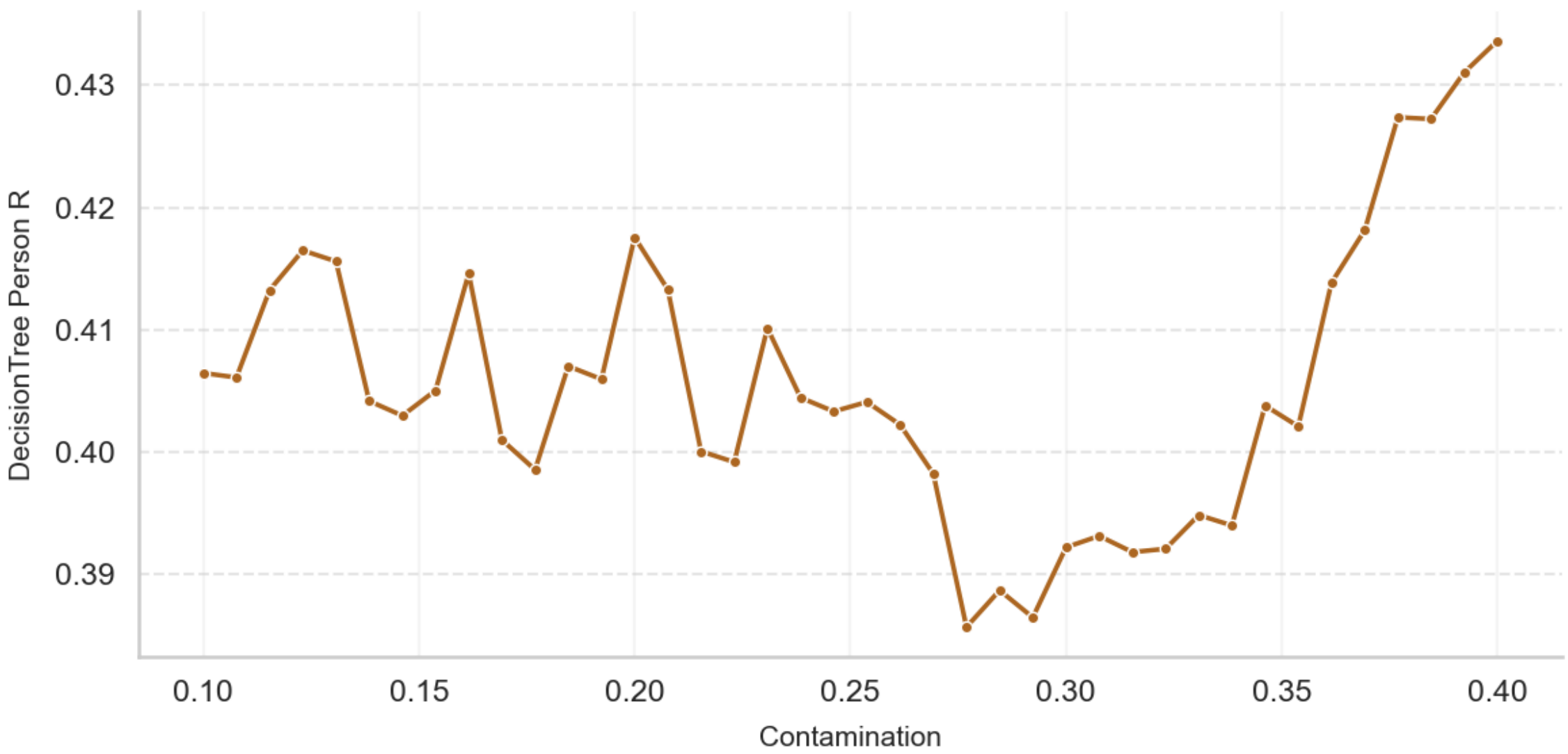

Figure S223: Correlation plot obtained between the data generated by the Decision Tree Regressor model trained with PM7-derived features and the reference values as a function of data contamination, using Local Outlier Factor for outlier treatment.

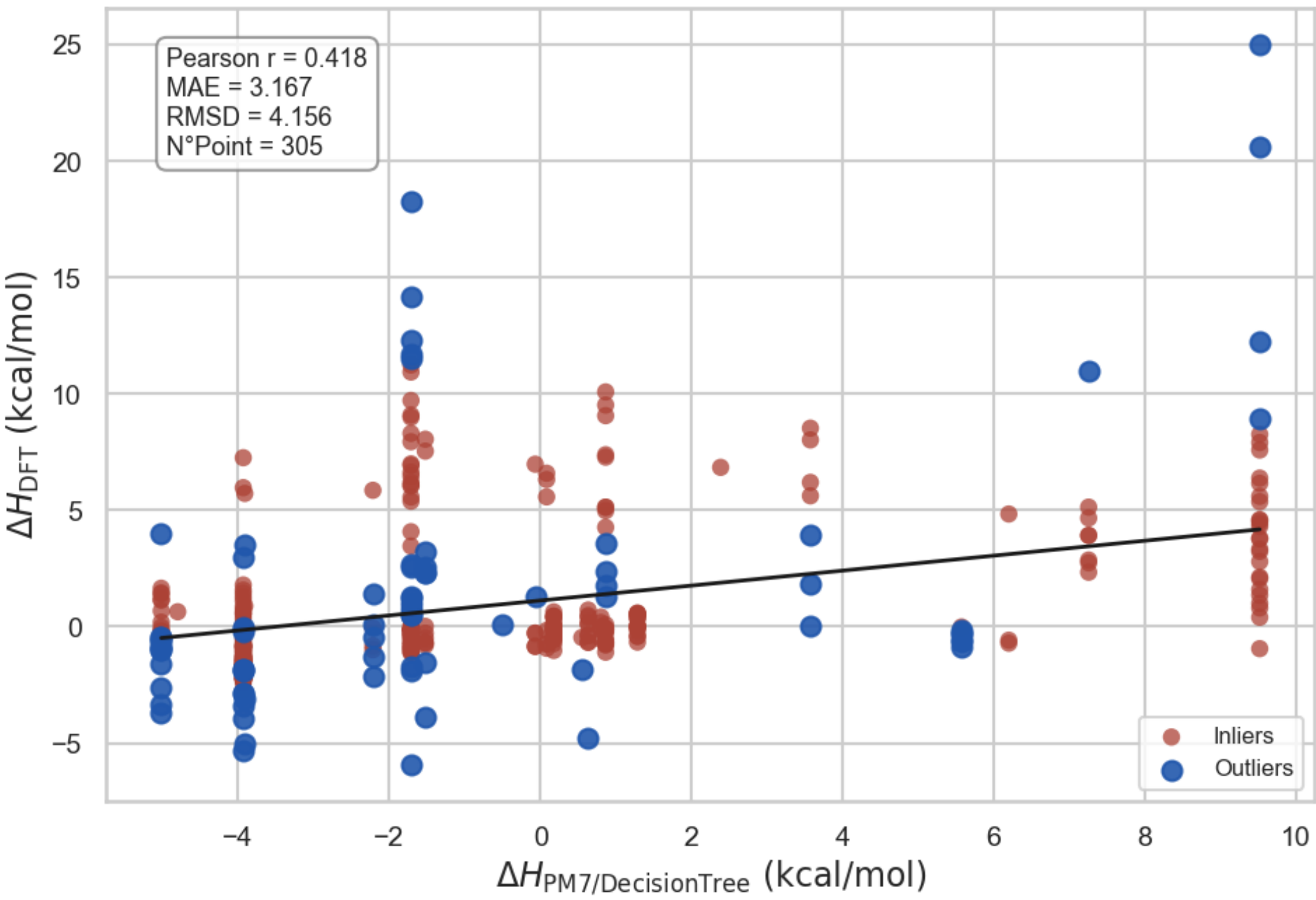

Figure S224: Scatter plots between reference DFT values and the values generated by the the Decision Tree Regressor model trained with PM7-derived features, applying Local Outlier Factor outlier treatment with a contamination level of 0.20.
